# Risk loci involved in giant cell arteritis susceptibility: a genome-wide association study

**DOI:** 10.1016/S2665-9913(24)00064-X

**Published:** 2024-05-08

**Authors:** Gonzalo Borrego-Yaniz, Lourdes Ortiz-Fernández, Adela Madrid-Paredes, Martin Kerick, José Hernández-Rodríguez, Sarah L Mackie, Augusto Vaglio, Santos Castañeda, Roser Solans, Jaume Mestre-Torres, Nader Khalidi, Carol A Langford, Steven Ytterberg, Lorenzo Beretta, Marcello Govoni, Giacomo Emmi, Marco A Cimmino, Torsten Witte, Thomas Neumann, Julia Holle, Verena Schönau, Gregory Pugnet, Thomas Papo, Julien Haroche, Alfred Mahr, Luc Mouthon, Øyvind Molberg, Andreas P Diamantopoulos, Alexandre Voskuyl, Thomas Daikeler, Christoph T Berger, Eamonn S Molloy, Daniel Blockmans, Yannick van Sleen, Mark Iles, Louise Sorensen, Raashid Luqmani, Gary Reynolds, Marwan Bukhari, Shweta Bhagat, Norberto Ortego-Centeno, Elisabeth Brouwer, Peter Lamprecht, Sebastian Klapa, Carlo Salvarani, Peter A Merkel, María C Cid, Miguel A González-Gay, Ann W Morgan, Javier Martin, Ana Márquez, Spanish GCA Group, José Luis Callejas, Luis Caminal-Montero, Marc Corbera-Bellalta, Eugenio de Miguel, J. Bernardino Díaz-López, María Jesús García-Villanueva, Carmen Gómez-Vaquero, Mercedes Guijarro-Rojas, Ana Hidalgo-Conde, Begoña Marí-Alfonso, Agustín Martínez-Berriochoa, Inmaculada C. Morado, Javier Narváez, Marc Ramentol-Sintas, Aleida Martínez-Zapico, Víctor Manuel Martínez-Taboada, José A. Miranda-Filloy, Jordi Monfort, Mercedes Pérez-Conesa, Sergio Prieto-González, Enrique Raya, Raquel Ríos-Fenández, Julio Sánchez-Martín, Bernardo Sopeña, Laura Tío, Ainhoa Unzurrunzaga, UK GCA Consortium, Oliver Wordsworth, Isobel Whitwell, Jessica Brock, Victoria Douglas, Chamila Hettiarachchi, Jacqui Bartholomew, Stephen Jarrett, Gayle Smithson, Michael Green, Pearl Clark Brown, Cathy Lawson, Esther Gordon, Suzanne Lane, Rebecca Francis, Bhaskar Dasgupta, Bridgett Masunda, Jo Calver, Yusuf Patel, Charlotte Thompson, Louise Gregory, Sarah Levy, Ajit Menon, Amy Thompson, Lisa Dyche, Michael Martin, Charles Li, Ramasharan Laxminarayan, Louise Wilcox, Ralph de Guzman, John Isaacs, Alice Lorenzi, Ross Farley, Helain Hinchcliffe-Hume, Victoria Bejarano, Susan Hope, Pradip Nandi, Lynne Stockham, Catherine Wilde, Donna Durrant, Mark Lloyd, Chee-Seng Ye, Rob Stevens, Amjad Jilani, David Collins, Suzannah Pegler, Ali Rivett, Liz Price, Neil McHugh, Sarah Skeoch, Diana O'Kane, Sue Kirkwood, Saravanan Vadivelu, Susan Pugmire, Shabina Sultan, Emma Dooks, Lisa Armstrong, Hala Sadik, Anupama Nandagudi, Tolu Abioye, Angelo Ramos, Steph Gumus, Nidhi Sofat, Abiola Harrison, Abi Seward, Susan Mollan, Ray Rahan, Helen Hawkins, Hedley Emsley, Anna Bhargava, Vicki Fleming, Marianne Hare, Sonia Raj, Emmanuel George, Nicola Allen, Karl Hunter, Eoin O'Sullivan, Georgina Bird, Malgorzata Magliano, Katarina Manzo, Bobbie Sanghera, David Hutchinson, Fiona Hammonds, Poonam Sharma, Richard Cooper, Graeme McLintock, Zaid S. Al-Saffar, Mike Green, Kerry Elliott, Tania Neale, Janine Mallinson, Peter Lanyon, Marie-Josephe Pradere, Natasha Jordan, Ei Phyu Htut, Thelma Mushapaidzi, Donna Abercrombie, Sam Wright, Jane Rowlands, Chetan Mukhtyar, James Kennedy, Damodar Makkuni, Elva Wilhelmsen, Michael Kouroupis, Lily John, Rod Hughes, Margaret Walsh, Marie Buckley, Kirsten Mackay, Tracey Camden-Woodley, Joan Redome, Kirsty Pearce, Thiraupathy Marianayagam, Carina Cruz, Elizabeth Warner, Ishmael Atchia, Claire Walker, Karen Black, Stacey Duffy, Lynda Fothergill, Rebecca Jefferey, Jackie Toomey, Ceril Rhys-Dillon, Carla Pothecary, Lauren Green, Tracey Toms, Linda Maher, Diana Davis, Amrinder Sayan, Mini Thankachen, Mahdi Abusalameh, Jessica Record, Asad Khan, Sam Stafford, Azza Hussein, Clare Williams, Alison Fletcher, Laura Johson, Richard Burnett, Robert Moots, Helen Frankland, James Dale, Karen Black, Kirsten Moar, Carol Hollas, Ben Parker, Derek Ridings, Sandhya Eapen, Sindhu John, Jo Robson, Lucy Belle Guthrie, Rose Fyfe, Moira Tait, Jonathan Marks, Emma Gunter, Rochelle Hernandez, Smita Bhat, Paul Johnston, Muhammad Khurshid, Charlotte Barclay, Deepti Kapur, Helen Jeffrey, Anna Hughes, Lauren Slack, Eleri Thomas, Anna Royon, Angela Hall, Jon King, Sindi Nyathi, Vanessa Morris, Madhura Castelino, Ellie Hawkins, Linda Tomson, Animesh Singh, Annalyn Nunag, Stella O'Connor, Nathan Rushby, Nicola Hewitson, Kenny O'Sunmboye, Adam Lewszuk, Louise Boyles, Martin Perry, Emma Williams, Christine Graver, Emmanuel Defever, Sanjeet Kamanth, Dominic Kay, Joe Ogor, Louise Winter, Sarah Horton, Gillian Welch, Kath Hollinshead, James Peters, Julius Labao, Andrea Dmello, Julie Dawson, Denise Graham, Denise De Lord, Jo Deery, Tracy Hazelton, Vasculitis Clinical Research Consortium, Simon Carette, Sharon Chung, David Cuthbertson, Lindsy J. Forbess, Ora Gewurz-Singer, Gary S. Hoffman, Curry L. Koening, Kathleen M. Maksimowicz-McKinnon, Carol A. McAlear, Larry W. Moreland, Christian Pagnoux, Philip Seo, Ulrich Specks, Robert F. Spiera, Antoine Sreih, Kenneth J. Warrington, Paul A. Monach, Michael Weisman

**Affiliations:** a Institute of Parasitology and Biomedicine López-Neyra, Consejo Superior de Investigaciones Científicas (CSIC), Granada, Spain; b Department of Clinical Pharmacy, San Cecilio University Hospital, Instituto de Investigación Biosanitaria de Granada (ibs.Granada), Granada, Spain; c Vasculitis Research Unit, Department of Autoimmune Diseases, Hospital Clinic of Barcelona, Institut d'Investigacions Biomèdiques August Pi i Sunyer (IDIBAPS), University of Barcelona, Barcelona, Spain; d School of Medicine, University of Leeds, Leeds, UK; e Leeds Institute for Data Analytics, University of Leeds, Leeds, UK; f NIHR Leeds Biomedical Research Centre, Leeds Teaching Hospitals NHS Trust, Leeds, UK; g NIHR Leeds Medtech and In Vitro Diagnostics Co-Operative, Leeds Teaching Hospitals NHS Trust, Leeds, UK; h Department of Biomedical Experimental and Clinical Sciences “Mario Serio”, University of Florence, Florence, Italy; i Meyer Children's Hospital, Nephrology and Dialysis Unit, Florence, Italy; j Department of Rheumatology, Hospital de la Princesa, IIS-IP, Madrid, Spain; k Autoimmune Systemic Diseases Unit, Department of Internal Medicine, Hospital Vall d'Hebron, Autonomous University of Barcelona, Barcelona, Spain; l Division of Rheumatology, McMaster University, Hamilton, ON, Canada; m Department of Rheumatic and Immunologic Diseases, Cleveland Clinic, Cleveland, OH, USA; n Division of Rheumatology, Mayo Clinic, Rochester, NY, USA; o Referral Center for Systemic Autoimmune Diseases, Fondazione IRCCS Ca' Granda Ospedale Maggiore Policlinico di Milano, Milan, Italy; p Department of Rheumatology, Azienda Ospedaliero Universitaria S Anna, University of Ferrara, Ferrara, Italy; q Department of Experimental and Clinical Medicine, University of Firenze, Florence, Italy; r Centre for Inflammatory Diseases, Department of Medicine, Monash Medical Centre, Monash University, Clayton, VIC, Australia; s Research Laboratory and Academic Division of Clinical Rheumatology, Department of Internal Medicine, University of Genova, Genova, Italy; t Hannover Medical School, Hannover, Germany; u Klinik für Innere Medizin III, University-Hospital Jena, Jena, Germany; v Department of Rheumatology, Cantonal Hospital St Gallen, St Gallen, Switzerland; w Vasculitis Clinic, Klinikum Bad Bramstedt and University Hospital of Schleswig Holstein, Bad Bramstedt, Germany; x Department of Rheumatology and Immunology, Universitätsklinikum Erlangen, Erlangen, Germany; y Department of Internal Medicine, Toulouse University Hospital Center, Toulouse, France; z Hôpital Bichat, Université Paris-Cité, Service de Médecine Interne, Paris, France; aa Department of Internal Medicine and French Reference Center for Rare Auto-immune & Systemic Diseases, Pitié-Salpêtrière Hospital, Assistance Publique–Hôpitaux de Paris, Paris, France; ab ECSTRRA Research Unit, Centre of Research in Epidemiology and Statistics, Sorbonne Paris Cité Research Center UMR 1153, Inserm, Paris, France; ac Cochin Hospital, National Referral Center for Rare Autoimmune and Systemic Diseases, Université Paris Descartes, Department of Internal Medicine, Assistance Publique–Hôpitaux de Paris, Paris, France; ad Department of Rheumatology, Oslo University Hospital, Oslo, Norway; ae Department of Rheumatology, Hospital of Southern Norway Trust, Kristiansand, Norway; af Department of Rheumatology and Clinical Immunology, Amsterdam University Medical Centre, Amsterdam, Netherlands; ag Department of Rheumatology, University Hospital Basel and Department of Clinical Research, University of Basel, Basel, Switzerland; ah Department of Biomedicine and Department of Internal Medicine, Translational Immunology and Medical Outpatient Clinic, University Hospital Basel, Basel, Switzerland; ai Department of Rheumatology, Centre for Arthritis and Rheumatic Diseases, St Vincent's University Hospital, Dublin Academic Medical Centre, Dublin, Ireland; aj Department of General Internal Medicine, University Hospital Gasthuisberg, Leuven, Belgium; ak Department of Rheumatology and Clinical Immunology, University of Groningen, University Medical Center Groningen, Groningen, Netherlands; al Nuffield Department of Orthopaedics Rheumatology and Musculoskeletal Sciences, Oxford NIHR Biomedical Research Centre, University of Oxford, Oxford, UK; am Center for Immunology and Inflammatory Diseases, Massachusetts General Hospital, Boston, MA, USA; an Rheumatology Department, University Hospitals of Morecambe Bay NHS Foundation Trust, Royal Lancaster Infirmary, Lancaster, UK; ao Faculty of Health and Medicine, Lancaster University, Lancaster, UK; ap West Suffolk NHS Foundation Trust, Bury Saint Edmunds, Bury St Edmunds, UK; aq Department of Medicine, University of Granada, Instituto de Investigación Biosanitaria de Granada ibs GRANADA, Granada, Spain; ar Department of Rheumatology and Clinical Immunology, University of Lübeck, Lübeck, Germany; as Azienda USL-IRCCS di Reggio Emilia and Università di Modena e Reggio Emilia, Reggio Emilia, Italy; at Division of Rheumatology, Department of Medicine, and Division of Epidemiology, Department of Biostatistics, Epidemiology, and Informatics, University of Pennsylvania, Philadelphia, PA, USA; au Division of Rheumatology, IIS-Fundación Jiménez Díaz, Madrid, Spain; av Department of Medicine, University of Cantabria, Santander, Spain

## Abstract

**Background:**

Giant cell arteritis is an age-related vasculitis that mainly affects the aorta and its branches in individuals aged 50 years and older. Current options for diagnosis and treatment are scarce, highlighting the need to better understand its underlying pathogenesis. Genome-wide association studies (GWAS) have emerged as a powerful tool for unravelling the pathogenic mechanisms involved in complex diseases. We aimed to characterise the genetic basis of giant cell arteritis by performing the largest GWAS of this vasculitis to date and to assess the functional consequences and clinical implications of identified risk loci.

**Methods:**

We collected and meta-analysed genomic data from patients with giant cell arteritis and healthy controls of European ancestry from ten cohorts across Europe and North America. Eligible patients required confirmation of giant cell arteritis diagnosis by positive temporal artery biopsy, positive temporal artery doppler ultrasonography, or imaging techniques confirming large-vessel vasculitis. We assessed the functional consequences of loci associated with giant cell arteritis using cell enrichment analysis, fine-mapping, and causal gene prioritisation. We also performed a drug repurposing analysis and developed a polygenic risk score to explore the clinical implications of our findings.

**Findings:**

We included a total of 3498 patients with giant cell arteritis and 15 550 controls. We identified three novel loci associated with risk of giant cell arteritis. Two loci, *MFGE8* (rs8029053; p=4·96 × 10^–8^; OR 1·19 [95% CI 1·12–1·26]) and *VTN* (rs704; p=2·75 × 10^–9^; OR 0·84 [0·79–0·89]), were related to angiogenesis pathways and the third locus, *CCDC25* (rs11782624; p=1·28 × 10^–8^; OR 1·18 [1·12–1·25]), was related to neutrophil extracellular traps (NETs). We also found an association between this vasculitis and HLA region and *PLG*. Variants associated with giant cell arteritis seemed to fulfil a specific regulatory role in crucial immune cell types. Furthermore, we identified several drugs that could represent promising candidates for treatment of this disease. The polygenic risk score model was able to identify individuals at increased risk of developing giant cell arteritis (90th percentile OR 2·87 [95% CI 2·15–3·82]; p=1·73 × 10^–13^).

**Interpretation:**

We have found several additional loci associated with giant cell arteritis, highlighting the crucial role of angiogenesis in disease susceptibility. Our study represents a step forward in the translation of genomic findings to clinical practice in giant cell arteritis, proposing new treatments and a method to measure genetic predisposition to this vasculitis.

**Funding:**

Institute of Health Carlos III, Spanish Ministry of Science and Innovation, UK Medical Research Council, and National Institute for Health and Care Research

## Introduction

Giant cell arteritis represents the most common form of vasculitis in Europe and North America, and mainly affects individuals aged 50 years and older.^1^ This type of large-vessel vasculitis primarily affects the aorta and its branches, resulting in ischaemic complications if not promptly treated.^2^ Giant cell arteritis not only reduces the quality of life for affected individuals, but also imposes a considerable socioeconomic burden on health-care systems given the high health-care costs associated with disease complications (eg, blindness, cerebrovascular accident, and aortic aneurysms) and glucocorticoid toxicity.

The cause of giant cell arteritis is complex, involving both genetic and environmental factors.^1^ Previous attempts to uncover the genetic basis of giant cell arteritis through large-scale genetic approaches led to the identification of two loci associated with the disease, *P4HA2* and *PLG*, and substantiated the crucial involvement of the HLA region in disease susceptibility.^3^, ^4^ The identified signals indicated a genetic contribution of the angiogenesis process in disease pathogenesis, further supporting the proposed role of this mechanism in giant cell arteritis.

**Research in context**

**Evidence before this study**

Giant cell arteritis is an immune-mediated inflammatory disease with a strong genetic component that remains poorly understood. Our incomplete understanding of the pathogenesis of this chronic large-vessel vasculitis limits our ability to identify new therapeutic targets, biomarkers, and preventive strategies. Previous research has shown that genome-wide association studies (GWAS) have great potential to discover the genetic factors that contribute to the development of complex diseases, such as giant cell arteritis. We searched PubMed, with no language restrictions, for articles published from database inception to Aug 31, 2023, using the search terms “giant cell arteritis”, “temporal arteritis” and “genome wide”. We found only one GWAS on giant cell arteritis reporting three risk loci associated with susceptibility to this vasculitis; these data were included in the present study.

**Added value of this study**

In this GWAS and meta-analysis of the largest cohort of patients with giant cell arteritis studied to date, we identified three novel loci associated with giant cell arteritis susceptibility. Through a comprehensive functional analysis, we identified relevant cell types and potential causal variants, and prioritised genes involved in angiogenesis and neutrophil extracellular traps. Notably, we pinpointed several potentially repositionable drugs for giant cell arteritis treatment and developed a genetic risk model capable of identifying individuals at high risk of developing giant cell arteritis.

**Implications of all the available evidence**

Enhancing our understanding of giant cell arteritis opens up opportunities for uncovering underlying disease mechanisms, thereby potentially advancing the clinical management of individuals with this type of vasculitis.

Despite the research efforts invested in the past decade to elucidate the genetic basis of giant cell arteritis, the cause of the condition remains largely unknown, with fewer identified loci associated with disease risk compared with other immune-mediated inflammatory diseases with low prevalence, such as systemic sclerosis, Sjögren's disease, or even other vasculitides.^5^, ^6^ The low prevalence of giant cell arteritis and the high statistical power necessary for genomic studies have imposed limitations on previous research, hindering substantial discoveries. Furthermore, the functional consequences of risk alleles associated with giant cell arteritis, as well as the potential causal genes and cell types implicated, have not yet been investigated. Additionally, previous studies have not explored the clinical potential of genetic findings in this type of vasculitis.

Herein, we report the largest genome-wide association study (GWAS) in giant cell arteritis to date. We aimed to characterise the genetic factors contributing to disease susceptibility; assess the functional consequences of loci associated with giant cell arteritis, prioritising potential causal genes and identifying new potential therapeutic options; and evaluate the ability of genetic findings to predict the risk of giant cell arteritis.

## Methods

### Study design and participants

Patients with giant cell arteritis and healthy controls of European ancestry were included in this GWAS, from ten different cohorts across France, Germany, Ireland, Italy, Norway, the Netherlands, Spain, Switzerland, the UK, and North America (appendix p 11). A proportion of the study population were included in a previous GWAS,^3^ and the remaining participants were newly recruited for this study. After quality controls of the genomic data, a total of 3498 patients and 15 550 controls were finally included.

Patients were eligible for inclusion if they met the 1990 American College of Rheumatology classification criteria for giant cell arteritis;^7^ however, a confirmation of giant cell arteritis diagnosis was required for the inclusion of patients in the study. Confirmation was provided by positive temporal artery biopsy, positive temporal artery doppler ultrasonography, or imaging techniques confirming large-vessel vasculitis.

All patients and controls provided written informed consent in accordance with the tenets of the Declaration of Helsinki. The protocol adhered to all ethical regulations and the study was approved by the Ethics Committee of the Spanish National Research Council and the Ethic Committee of Research of the Granada Province, as well as by all participating institutions. A favourable ethical opinion was granted for participants of the UK Giant Cell Arteritis Consortium by the Yorkshire and the Humber Leeds West Research Ethics Committee (05/Q1108/28).

### Genotyping and quality control

We performed genome-wide genotyping using the arrays specified in the appendix (p 11). All genotype quality controls were performed with PLINK (version 1.90). Strict quality control parameters were applied to all cohorts, including both patients and controls, to filter rare single-nucleotide polymorphisms (SNPs; minor allele frequency <0·01), SNPs with call rates lower than 0·98 and samples with call rates lower than 0·95, and SNPs that deviated from Hardy–Weinberg equilibrium (p<0·001). To eliminate duplicates and relatives, one sample from every pair of duplicated or related samples estimated by identity by descent (Pi_Hat >0·99 for duplicates and Pi_Hat >0·45 for relatives) was discarded.

### Imputation

We performed whole-genome imputation on the filtered genotype data using the TOPMed Imputation server), including the TOPMed reference data as a reference panel. After imputation, a probability threshold of 0·9 was established to merge genotypes using GTOOL), otherwise the genotype was set as missing. Additional quality controls of each imputed dataset were performed with PLINK (version 1.90), removing SNPs with call rates lower than 0·98, SNPs that deviated from the Hardy–Weinberg equilibrium (p<0·001), or SNPs with a minor allele frequency lower than 0·01. In addition, singleton SNPs and variants that showed genetic inconsistency between patients and controls were removed using an in-house *perl* script.

### Principal component analysis

To estimate ancestry outliers, we selected around 100 000 quality-filtered independent SNPs to calculate ten principal components for each independent cohort. The principal component analysis was performed with PLINK, GCTA64 (and R-based software under the GNU operating system (public licence version 2). Outliers showing more than four standard deviations from the cluster centroids when plotted by principal components were removed from further analyses. Variance explained by principal components is shown in the appendix (p 12).

### Association analysis

The statistical power of the study, calculated with the GAS Power Calculator, is shown in the appendix (p 13). Each cohort was individually analysed with PLINK, whereby a logistic regression model of additive effects, including sex and ten principal components as covariates, was performed. To test if the population stratification was correctly addressed, we calculated the genomic inflation factor λ for every cohort and λ_1000_ for cohorts with more than 1000 patients and 1000 controls in total (appendix p 11). We then conducted a fixed-effect, inverse variance-weighted meta-analysis to combine the odd ratios (ORs) obtained in each independent regression. The heterogeneity of ORs across cohorts was assessed with *I2* and Cochran's Q tests. SNPs with p values less than and including 5 × 10^–8^ were considered to be statistically significant and SNPs with p values less than and including 5 × 10^–5^ were considered to be suggestive SNPs. After these analyses, we used a variant effect prediction analysis to annotate associated SNPs.

Details of fine-mapping and the functional annotation of associated variants, as well as drug repositioning and the genetic risk analysis are provided in the appendix (pp 4–7).

### Stepwise conditional analysis in loci associated with giant cell arteritis

To identify independent signals in loci associated with giant cell arteritis, we performed a joint conditional analysis using GCTA. This method corrects summary-level statistics considering the linkage disequilibrium between SNPs, estimated from a reference sample set. Associated regions were analysed, considering the most significant SNP (lead SNP) as a covariate and a window of 1·5 Mb around it. Any SNP with a p value less than 1 × 10^–6^ after conditioning, alongside an r^2^ value less than 0·2 and D' value less than 0·5 with the lead SNP, was considered to be independent and was included as a covariate in a new round of conditional analysis.

### HLA imputation

We used a reliable imputation method to investigate the complex associations within the extended HLA region located on chromosome 6. Specifically, we used the SNP2HLA method in conjunction with the Beagle software package and a reference panel collected by the Type 1 Diabetes Genetics Consortium, comprising 5225 individuals of European ancestry and 8961 polymorphisms (including HLA SNPs, classical alleles, and amino acid variants across the extended MHC region)^8^ Each cohort was imputed separately and the results were subsequently meta-analysed with the same methods and quality controls described above. To identify secondary signals, we conducted a conditional analysis. Any variant with a p value less than 1 × 10^–6^ was deemed to be independent if it continued to show a p value less than 1 × 10^–6^ after conditioning, along with having an r^2^ value less than 0·2 and a D' value less than 0·5 with all previously defined independent variants. The R packages ggplot2, ggbreak, and MetBrewer were used for plotting these results.

### Role of the funding source

The funder of the study had no role in study design, data collection, data analysis, data interpretation, or writing of the report.

## Results

A total of 3901 patients with giant cell arteritis and 17 475 healthy controls of European ancestry were included in this GWAS, from ten different cohorts across Europe and North America (appendix p 11). 2138 (54·8%) patients with giant cell arteritis and 4997 (28·6%) controls were included in a previous GWAS,^3^ the remaining 1763 (45·2%) patients and 12 478 (71·4%) controls were newly recruited for this study. After quality controls of the genomic data and outlier removal, a total of 3498 patients with giant cell arteritis and 15 550 controls were finally included. Excluding the HLA region, all cohorts showed a λ (or λ_1000_ if applicable) below 1·03 (appendix p 11). All SNPs that were not present in at least two datasets were removed from the analysis, resulting in 6 691 295 SNPs that were meta-analysed. Considering all variants, giant cell arteritis heritability explained by SNPs was estimated to be 15·1% (SD 0·8). Excluding the HLA region, this value was reduced to 13·4% (0·8), implying a contribution of both HLA and non-HLA loci in giant cell arteritis.

After the meta-analysis, 2955 SNPs were found to be significantly associated with giant cell arteritis (appendix pp 8, 16–80). These signals belonged to five different loci (table 1), two of which are established risk loci for giant cell arteritis, 6p21.32 (*HLA-DQA1*, rs41269974; p=1·60 × 10^–87^; OR 2·03 [95% CI 1·90–2·17]) and 6q26 (*PLG*, rs4252114; p=1·38 × 10^–13^; OR 1·25 [1·18–1·32]). Additionally, three new genomic associations with giant cell arteritis were identified at the 8p21.1 (*CCDC25*, rs11782624; p=1·28 × 10^–8^; OR 1·18 [95% CI 1·12–1·25]), 15q26.1 (*MFGE8*, rs8029053; p=4·96 × 10^–8^; OR 1·19 [1·12–1·26]) and 17q11.2 (*VTN*, rs704; p=2·75 × 10^–9^; OR 0·84 [0·79–0·89]) regions. Associations between the lead SNPs of these loci and giant cell arteritis are reported by cohort in the appendix (p 81). The conditional analysis of non-HLA signals showed no additional associations within these loci.

**Table 1:** Lead significant genetic variants by genomic region, credible sets of non-HLA signals, and potential mapped genes proposed by gene prioritisation

	**Base pair (hg38)**	**rs identification**	**Nearest gene**	**Effect allele**	**p value**	**OR (95% CI)**	**Number of SNPs in LD block**	**Number of SNPs in credible set (ΣPP >0·95)**	**Credible set of SNPs**	**Candidate genes**
6p21.32	32652425	rs41269974	HLA-DQA1*	A	1·60 × 10^−87^	2·03 (1·90–2·17)	..	..	..	..
6q26	160722158	rs4252114	*PLG*	C	1·38 × 10^−13^	1·25 (1·18–1·32)	347	3	rs4252114, rs1897108, rs1321197	*PLG*
8p21.1	27755870	rs11782624	*CCDC25*	T	1·28 × 10^−8^	1·18 (1·12–1·25)	81	1	rs9644049	*CCDC25, CLU, ELP3, ESCO2, LEPROTL1, PBK, SCARA3, SCARA5, TMEM66*
15q26.1	88906856	rs8029053	*MFGE8*	T	4·96 × 10^−8^	1·19 (1·12–1·26)	20	1	rs11073821	*ACAN, HAPLN3, MFGE8*
17q11.2	28367840	rs704	*VTN*	A	2·75 × 10^−9^	0·84 (0·79–0·89)	55	13	rs704, rs3093680, rs1007398, rs4795435, rs1128162, rs1128161, rs2227736, rs2227735, rs10853128, rs2239908, rs6505077, rs8081240, rs8079943	*FOXN1, IFT20, LGALS9, NUFIP2, POLDIP2, SARM1, SEBOX, SLC13A2, SLC46A1, TMEM199, TMEM97, TNFAIP1, VTN*

Independent genetic variants that reached genome-wide significance in the meta-analysis are shown. OR=odds ratio. SNP=single nucleotide polymorphism. LD=linkage disequilibrium. ΣPP=sum of posterior probability.

* Fine mapping of the HLA region was extended (see table 2).

To refine the association between HLA region and giant cell arteritis, 8643 polymorphisms (including SNPs, HLA classical alleles, and amino acidic variants) were meta-analysed within the extended MHC. 800 (9·3%) polymorphisms showed a genome-wide level of significance (appendix pp 82–96). The most significant signal corresponded to a SNP (rs17882084) within the *HLA-DRB1* gene (p=4·34 × 10^–87^; OR 2·02; table 2). This polymorphism showed strong linkage disequilibrium with the HLA-DRB1*04 classical allele (r^2^ 0·98) and the presence of histidine at the amino acid position 13 of the DRβ1 molecule (r^2^ 0·94).

**Table 2:** Independent giant cell arteritis-associated HLA variants after conditional analysis

	**Base pair (hg38)**	**Nearest gene**	**Amino acid variant in highest LD (r2)**	**Classical HLA allele in highest LD (r2)**	**Effect allele**	**Patient minor allele frequency**	**Control minor allele frequency**	p value*	**Conditional p value**†	**OR (95% CI)**
rs17882084	32581836	*HLA-DRB1*	DRB1-180-Leu (0·999); DRB1-96-Tyr (0·999)	HLA-DRB1*04 (0·984)	A	0·26	0·16	4·34 × 10^−87^	..	2·02 (1·89–2·15)
rs1049087	32662112	*HLA-DQB1*	DQB1-57-Ala (0·726)	HLA-DQA1*01 (0·424); HLA-DQB1*02 (0·422)	A	0·51	0·41	7·70 × 10^−44^	1·07 × 10^−21^	1·49 (1·42–1·58)
rs2856726	32698944	Intergenic	DQB1-167-His (0·443); DQB1-13-Gly (0·443)	HLA-DQB1*0301 (0·419)	A	0·41	0·36	1·35 × 10^−7^	3·75 × 10^−8^	1·17 (1·10–1·24)
rs2596501	31353434	*HLA-B*	B-97-Ser/Asn/Val (0·499); B-45-Thr/Lys (0·384)	HLA-C*07 (0·171)	G	0·50	0·48	2·79 × 10^−10^	1·16 × 10^−7^	1·20 (1·14–1·27)
HLA-DPB1*03	33081591	*HLA-DPB1*	DPB1-57-Asp (0·593); DPB1-65-Leu (0·570)	..	..	0·08	0·11	3·60 × 10^−7^	4·37 × 10^−7^	0·77 (0·70–0·85)

LD=linkage disequilibrium. OR=odds ratio.

* p values of the meta-analysis.

† p values of the meta-analysis after stepwise conditional analysis, including previous associated signals.

The conditional analysis identified four additional independent variants that, together with the *HLA-DRB1* SNP, encompassed the association with HLA region (table 2; appendix p 9). Notably, three of these variants were located in the HLA class II region: within *HLA-DQB1* (rs1049087), the HLA-DPB1*03 classical allele, and in an intergenic region between *HLA-DQB1* and *HLA-DQA2* (rs2856726). The other variant (rs2596501) was located in the HLA class I region, downstream of *HLA-B*. The *HLA-DQB1* polymorphism (rs1049087) showed considerable linkage disequilibrium with amino acid changes in both DRβ1 and DQβ1 molecules (r^2^ 0·6), as well as moderate linkage disequilibrium with the HLA-DQA1*01 allele (r^2^ 0·42). Additionally, the SNP at *HLA-B* showed moderate linkage disequilibrium with amino acid changes on positions 97 and 45 of the HLA-B protein. It should be noted that the presence of threonine at position 45 of HLA-B was previously identified as an independent HLA signal in giant cell arteritis;^4^ however, the linkage disequilibrium between threonine at position 45 of HLA-B and the SNP identified in the present study was low (r^2^ 0·15).

Subsequently, we searched the Open Targets Genetics tool to explore the potential regulatory role of the four HLA SNPs independently associated with giant cell arteritis. All SNPs, except for rs17882084, showed a role in regulating protein concentrations, gene expression, or alternative splicing in tissues or cell types related to giant cell arteritis (appendix p 97).

Regarding non-HLA signals, 1·7% of the identified suggestive and significant variants were detected within coding regions, suggesting a predominantly regulatory role. Thus, to explore this potential regulatory function of the associated loci, we examined all significant and suggestive associations for enrichment in nine histone marks previously characterised for immune and vascular tissues. These findings showed a significant enrichment in various immune cell types, including CD4^+^ and CD8^+^ T cells, B cells, neutrophils, and monocytes, with a notably strong representation of natural killer (NK) cell regulation (appendix p 10). By contrast, we detected no significant enrichment in vascular tissues or for most of the histone marks representative of promoter regions (ie, H3K4me2, H3K4me3, H3K9ac). These findings imply that variants associated with giant cell arteritis primarily affect immune cells and that these genetic variations predominantly coincide with actively transcribed regions and enhancers.

To identify potential causal SNPs outside the HLA region, we defined 95% credible sets through Bayesian fine-mapping. Using this strategy, we were able to effectively identify concise credible sets in three of four non-HLA significant signals (table 1; appendix pp 98–108). For two of these loci, 8p21.1 and 15q26.1, the credible set comprised a single variant (rs9644049 at 8p21.1 and rs11073821 at 15q26.1). In the case of the 6q26 locus, this analysis prioritised three SNPs as probably causal of the association identified in this region (rs4252114 [posterior probability 0·46], rs1897108 [0·26], and rs1321197 [0·25]). By contrast, the fine-mapping of the signal on chr17q11.2 yielded few results. The location of this signal in a remarkably dense genetic region is likely to render the functional annotations not informative enough to single out variants with more prominent functional consequences. Within this signal, the credible set comprised the 13 most significantly associated variants, showing inferior prioritisation ability for this locus.

**Figure 1:**
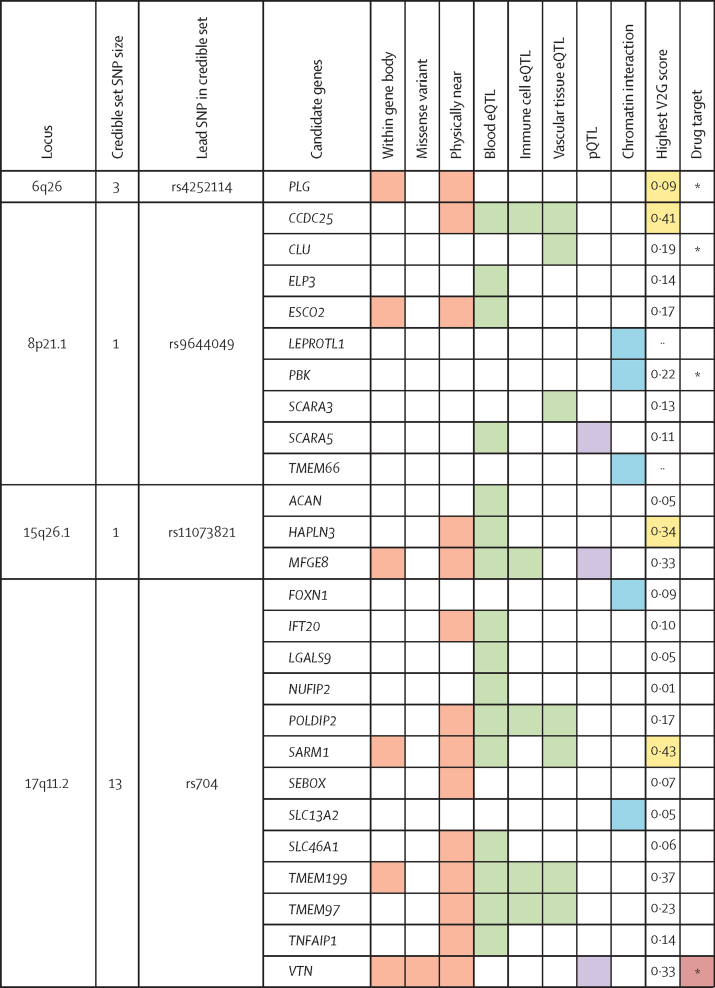
Gene prioritisation for loci associated with giant cell arteritis Colours indicate the SNPs or genes associated with giant cell arteritis that overlap with the considered functional annotations. Different colours indicate different categories of annotations. The items evaluated comprise information about the genomic location of the genetic variants, their correlation with gene and protein expression, evidence of chromatin interaction, and their V2G score from Open Target Genetics (appendix p 15). The last column shows if the prioritised causal genes are targets for approved drugs, according to our drug repurposing analysis. The drug target item was not considered for gene prioritisation. pQTL=protein quantitative trait loci. eQTL=expression quantitative trait loci. SNP=single nucleotide polymorphism. V2G=Variant-to-Gene. *The gene is in high-confidence protein–protein interaction with an identified drug target.

Subsequently, considering the SNPs that comprise the credible set of each non-HLA signal, we explored the genes that could be affected by them, considering FUMA gene mapping and additional data from Open Targets Genetics. A total of 26 candidate genes were identified (figure 1). The most supported candidates for the associated loci considering different parameters were *PLG* (chromosome 6), *CCDC25* (chromosome 8), *MFGE8* (chromosome 15), and *VTN* (chromosome 17). *SARM1* also emerged as a potential candidate gene at chromosome 17. However, the lead SNP rs704 is a missense variant of *VTN*, which strongly supports its role as a credible causal factor for this association. This observation is further substantiated by the fact that rs704 acts as a protein quantitative trait locus, influencing the concentrations of protein encoded by *VTN*.

To identify potential new treatment options for giant cell arteritis, we considered all significant non-HLA signals and defined a set of 136 proteins, comprising both proteins encoded by the suggested causal genes and their high confidence interacting proteins (appendix pp 109–111). Through an extensive search on the DrugBank database, we found 181 different drugs targeting these proteins (appendix pp 112–136). Focusing on plausible and approved drugs, we identified different promising candidates on the basis of their mechanism of action for further investigation in giant cell arteritis treatment (table 3). Notably, the glycoprotein IIb/IIIa inhibitors abciximab, tirofiban, and eptifibatide are approved drugs for treating ischaemic complications in patients at high risk undergoing coronary intervention or prevention of myocardial infarction. Additionally, fostamatinib, a spleen tyrosine kinase inhibitor that is licensed in chronic immune thrombocytopenia and has successfully completed phase 3 clinical trials for treating rheumatoid arthritis, is among the repurposed candidate drugs.

**Table 3:** Selected repurposed drug candidates for giant cell arteritis treatment

	**Target**	**Licensed indication**
Abciximab	ITGB2B, ITGB3, VTN	Ischaemic cardiovascular events
Eptifibatide	ITGB3	Acute coronary syndrome
Tirofiban	ITGA2B, ITGB3	Acute coronary syndrome
Fostamatinib	CDK1, MELK, TTK	Chronic immune thrombocytopenia
Human C1 esterase inhibitor	KLKB1	Acute attacks of hereditary angioedema
Lanadelumab	KLKB1	Acute attacks of hereditary angioedema
Alteplase	FGA, PLG, PLAUR, SERPINE1	Lysis of acute massive pulmonary embolism, acute ischaemic stroke, and acute myocardial infarction
Anistreplase	FGA, PLG, SERPINE1	Lysis of acute pulmonary emboli, intracoronary emboli, and management of myocardial infarction
Tenecteplase	FGA, PLG, PLAUR, SERPINE1	Myocardial infarction and lysis of intracoronary emboli
Urokinase	FGA, PLG, SERPINE1	Lysis of acute massive pulmonary emboli, acute thrombi obstructing coronary arteries, occlusive thromboemboli in peripheral arteries and grafts, and restoration of patency to intravenous catheters
Reteplase	FGA, PLG, SERPINE1	Lysis of acute pulmonary emboli, intracoronary emboli, and management of myocardial infarction
Streptokinase	PLG	Acute evolving transmural myocardial infarction, pulmonary embolism, deep vein thrombosis, arterial thrombosis, or embolism and occlusion of arteriovenous cannulae

Only proposed candidate drugs are shown, the complete report of the drug repurposing analysis is provided in the appendix (pp 112–136).

Furthermore, we assessed the ability of associated variants to predict risk of giant cell arteritis. First, we developed a polygenic risk score model considering the five independent HLA variants identified in our analysis and a p value thresholding approach. Including only these HLA variants, the best polygenic risk score showed a prediction ability of area under the curve (AUC) 0·610. A clumping and p value thresholding approach including only independent non-HLA SNPs resulted in a model comprising 39 SNPs with poorer prediction (AUC 0·560). Nevertheless, applying this approach considering both HLA and non-HLA SNPs resulted in a model comprising 11 SNPs with the best predictive score (AUC 0·617). Using sex as a covariate for this model showed a minor decrease in prediction ability (AUC 0·616); therefore, sex was not included in the final model. The different polygenic risk score models and their predictive ability, as well as the final model with each variant weight are described in the appendix (pp 137–138).

**Figure 2:**
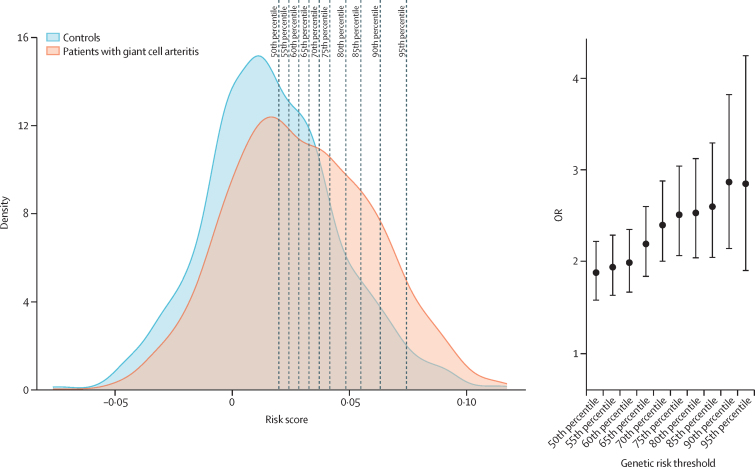
Genetic risk distribution Predictive ability of the polygenic risk score model, including 11 SNPs (HLA and non-HLA). (A) Density plot of the distribution of genetic risk between patients with giant cell arteritis and controls. Dashed lines represent the percentile thresholds used to calculate the OR of each division. (B) ORs associated with the group at high risk of giant cell arteritis when segregating the test sample by the same percentile thresholds. Error bars depict 95% CIs. OR=odds ratio.

After establishing the best polygenic risk score, we assessed its effectiveness in identifying individuals at high risk of giant cell arteritis. We categorised the test dataset into two groups, namely low risk and high risk, on the basis of different risk percentile thresholds. The results showed significant differences in risk score between groups for all studied divisions. Notably, the top 10% individuals at risk had an OR of 2·87 (95% CI 2·15–3·82) for the disease (figure 2). Giant cell arteritis risk stratification based on results is provided in the appendix (p 139).

## Discussion

In this study, we explored the genetic basis of giant cell arteritis through the largest GWAS conducted for this type of vasculitis to date. Our findings substantially increase the understanding of the genetic components contributing to the pathogenesis of giant cell arteritis. We identified three novel genetic risk factors and, through an extensive functional analysis, elucidated their potential biological consequences in giant cell arteritis pathogenesis. Furthermore, our results have clarified the association between HLA and this form of vasculitis, and substantiated the role of another established giant cell arteritis-associated locus, the *PLG* gene.

To overcome the challenge of identifying the mechanisms underlying loci associated with giant cell arteritis, we did a comprehensive functional analysis of the results from the GWAS. This approach led to a considerable reduction in the number of variants that could reliably explain these associations, thus improving our ability to interpret their biological involvement. In the case of the HLA region, we have defined a set of five variants that adequately encompass the observed association within this highly complex region. For non-HLA loci, we identified the most credible causal variants with biological relevance, facilitating a focused analysis of the potential causal genes of these signals.

The association observed within the HLA region in this study supports previous findings that identify giant cell arteritis as a disorder predominantly related to HLA class II. Nevertheless, in line with the outcomes of a previous giant cell arteritis ImmunoChip study,^4^ we have also verified the involvement of HLA class I in the manifestation of this disease. This finding is consistent with the associations observed in the only other form of large-vessel vasculitis, Takayasu's arteritis, which similarly shows a contribution of both HLA classes I and II in its pathogenesis.^9^

Our findings highlight a prominent role of the genetic component of giant cell arteritis in the process of arterial tissue destruction and neovascularisation, in which three of the four loci associated with giant cell arteritis, *PLG, VTN*, and *MFGE8*, are involved. The suggested role of *PLG* in the development of giant cell arteritis arises from its connection with the remodelling of damaged arterial tissue. This gene encodes plasminogen, a precursor to plasmin and angiostatin, which has a crucial role in various processes relevant to giant cell arteritis pathogenesis, including angiogenesis, inflammation, wound healing, and lymphocyte recruitment.^10^ Additionally, it has been shown that plasminogen functions as a regulator of macrophage reprogramming and neutrophil apoptosis in mouse models.^11^ Furthermore, in the present study, the scope of evidence indicating the participation of this pathway in giant cell arteritis has been expanded through the observed association between *VTN* and *MFGE8* and this type of vasculitis. *MFGE8* encodes a pre-proprotein that mainly gives rise to lactadherin, a glycoprotein found in the cell membrane that enhances the phagocytosis of apoptotic cells^12^ and promotes neovascularisation dependent on VEGF.^13^ In mouse models, *MFGE8* failure has been linked to the incidence of autoimmune diseases.^14^ Notably, the only genetic variant prioritised within this region was found to act as an expression quantitative trait locus according to our functional annotation analysis, with its risk allele correlating with decreased concentrations of *MFGE8* in the blood and with increased amounts of this gene in T cells. Vitronectin, encoded by *VTN*, is a glycoprotein that binds to plasminogen and various other molecules, and serves to stabilise the inhibitory conformation of PAI-1. Interestingly, PAI-1 has been shown to regulate VEGF signalling in a vitronectin-dependent manner, thus inhibiting angiogenesis.^15^ Taken together, our results depict an important contribution of an altered artery remodelling mechanism in giant cell arteritis susceptibility, which could be one of the key agents of the long-term damage occurring in the pathological process of this vasculitis.

It should be noted that the associated locus 17q11.2 constitutes a highly complex genetic region, representing a challenge in identifying the most likely causal gene. There is notable evidence that this association is caused by *VTN*, given that the lead SNP, rs704, is a missense variant and a protein quantitative trait locus for this gene. Specifically, it has been shown that the minor allele of rs704 results in higher vitronectin protein concentrations, stronger binding of vitronectin to PAI-1, and less cell-surface binding than the major allele.^16^, ^17^ However, evidence also supports the involvement of *SARM1*. *SARM1*, a member of the TIR adaptor family, has been linked to the regulation of essential inflammatory pathways and implicated as a regulatory element in the interleukin-1 pathway in rheumatoid arthritis monocytes.^18^ Therefore, considering their reasonable potential role in giant cell arteritis pathogenesis, either gene (*VTN* or *SARM1*) or a more intricate mechanism influencing both could underlie the observed association within this genomic locus.

In the past decade, understanding around the diverse roles that neutrophil extracellular traps (NETs) have in inflammation has increased.^19^ Interestingly, NETs have been associated with giant cell arteritis^20^ and their presence in the affected temporal artery of patients with giant cell arteritis has been reported.^21^ Although the specific involvement of NETs in giant cell arteritis requires further investigation, our findings contribute to reinforcing the important role of this mechanism in the pathogenesis of the disease. In this study, we identified an intronic variant of *CCDC25* that constitutes a risk factor for giant cell arteritis. Specifically, the risk allele of this SNP correlates with an increased expression of *CCDC25* in immune cells. This gene encodes a transmembrane receptor for NETs, which triggers the activation of the ILK-β–parvin pathway and enhances cell motility.^22^ Although this pathway has been associated with metastasis in cancer, in the context of giant cell arteritis, it could potentially serve as a signalling mechanism that enhances the recruitment of immune cells to pathological artery tissue. Of note, in granulomatosis with polyangiitis, another vasculitis mainly affecting small vessels, NETs have been observed to induce the expression of MMP-9 in monocytes, obtaining the capability of tissue invasion.^23^ In the context of giant cell arteritis, MMP-9 is the main effector for the loss of immune privilege in the arterial wall, a crucial event in disease initiation.^1^

Our results on cell enrichment showed that the genetic component of giant cell arteritis affects gene regulation in both adaptive and innate immune cells, including B cells, different subtypes of T cells, and monocytes. Additionally, we identified an enrichment in neutrophil gene regulation, coherent with the involvement of NETs in the pathogenic process of this type of vasculitis. Furthermore, this finding supports the previously suggested role of neutrophils in the pathogenesis of giant cell arteritis. Previous research has proposed the involvement of immature neutrophils in giant cell arteritis as promoters of vascular lesions^24^ and the participation of peripheral neutrophils in effector T-cell proliferation.^25^

Unexpectedly, the strongest enrichment was observed in NK cells. Although the number of NK cells is known to be decreased in patients with giant cell arteritis,^26^ the role of this cell type in the context of this vasculitis remains underexplored. Nevertheless, this same NK cell enrichment was reported in a previous cross-disease association study that investigated the shared genetic component across systemic vasculitides, including giant cell arteritis.^27^ These findings strongly suggest the potential role of NK cells as contributors to the development of giant cell arteritis.

Unlike previous findings, our study did not observe the reported association between *P4HA2* and giant cell arteritis, a gene that was suggested to be related to the artery remodelling process through the plasmin pathway.^3^ The potential involvement of this gene in the pathogenesis of giant cell arteritis has also been highlighted in a 2022 transcriptomic and methylomic profiling study of giant cell arteritis monocytes.^28^ We believe that this observed disparity could be attributed to the inherent high clinical heterogeneity among patients with giant cell arteritis. It is plausible that the influence of the *P4HA2* gene is specific to a particular subtype of patients with giant cell arteritis, who are potentially under-represented in our study.

Through an intense effort to translate these novel genomic results into tangible clinical applications, we have been able to prioritise several drugs that could be repurposed for patients with giant cell arteritis. Importantly, abciximab directly targets vitronectin, whereas fostamatinib has drawn attention for its reported safety and efficacy in phase 3 clinical trials for the treatment of patients with rheumatoid arthritis.^29^ Interestingly, fostamatinib was also suggested to be potentially repositionable in patients with vasculitides in a previous study.^27^ We have also introduced the first polygenic risk score aimed at detecting the genetic predisposition of giant cell arteritis. The best model, combining both HLA and non-HLA variants, enabled the identification of individuals at high risk with a level of risk similar to that observed in patients with monogenic diseases.^30^ Our results indicate that genetic profiling could be useful for predicting risk of giant cell arteritis and represent a further step towards the implementation of personalised medicine for patients with this type of vasculitis.

Although our GWAS has provided valuable insights into the genetic basis of giant cell arteritis, it is essential to acknowledge this study's limitations. In our analysis, we addressed potential confounding factors, including sex and population stratification, to ensure robust and reliable results. However, it is crucial to recognise that other confounding variables, not explicitly addressed in this study, might contribute to the observed associations. Additionally, to further elucidate the functional importance of variants associated with giant cell arteritis, we acknowledge the need to complement our findings from this GWAS with experimental validations, which will allow us to substantiate the causal roles of these variants and provide a more comprehensive understanding of their effect on giant cell arteritis.

In conclusion, this study has notably expanded our understanding of the genetic architecture of giant cell arteritis, leading to the identification of cell types and genes that have a crucial role in the pathogenesis of this form of vasculitis. Specifically, our findings show a relevant involvement of angiogenesis and NET signalling in the development of giant cell arteritis. Furthermore, our study represents a step forward in translating genomic findings into clinical practice in this disease, proposing new treatments and a method to assess genetic predisposition to develop this vasculitis. These discoveries hold substantial clinical importance, offering avenues for more specific patient care and potentially influencing healthcare policies related to giant cell arteritis treatment.

## Data sharing

Summary statistic data are available under reasonable request to the corresponding author.

## Declaration of interests

MCC reports support from the Spanish Ministry of Science and Innovation (PID2020-114909RB-I00), Vasculitis Foundation, Agency for the Management of University and Research Grants (2021 SGR 01561), and Kiniksa Pharmaceuticals; consulting fees or honoraria from GSK, CSL Vifor, AbbVie, and AstraZeneca; support for attending meetings from Kiniksa Pharmaceuticals; and participation on a data safety monitoring board or advisory board for GSK, CSL Vifor, and AstraZeneca. GE has acted as a consultant for GSK, AstraZeneca, Sobi, Novartis, Boehringer, and CSL Vifor. AWM reports support from the UK Medical Research Council (MRC), National Institute for Health and Care Research (NIHR), Leeds Care, and Roche Products; and consulting fees or honoraria from CSL Vifor and AstraZeneca. PL reports grants or contracts from the Federal Ministry of Education and Research, German Research Society, German Society for Rheumatology, John Grube Foundation, and CSL Vifor; consulting fees or honoraria from GSK, CSL Vifor, AstraZeneca, Bristol Myers Squibb, Boehringer Ingelheim, Forum für medizinische Fortbildung, Janssen, Rheumaakademie, and UCB; support for attending meetings from CSL Vifor; and participation on a data safety monitoring board or advisory board for GSK, CSL Vifor, AbbVie, and Novartis. TW reports consulting fees or honoraria from AbbVie, AstraZeneca, Lilly, UCB, and Novartis; and participation on a data safety monitoring board or advisory board for AbbVie, AstraZeneca, Lilly, UCB, Novartis, and Fresenius. MAG-G reports honoraria from GSK. NK reports grants or contracts from Bristol Myers Squibb, AbbVie, and Sanofi; and consulting fees or honoraria from Roche, Otsuka, GSK, and Mallinckrodt. CAL reports grants or contracts from Bristol Myers Squibb and support from the National Institutes of Health. PAM reports grants, contracts, or consulting fees from AbbVie, Amgen, AstraZeneca, ArGenx, Boehringer Ingelheim, Bristol Myers Squibb, Cabaletta, CSL Behring, Eicos, Electra, Forbius, Genentech–Roche, GSK, HiBio, InflaRx, Janssen, Jubilant, Kyverna, MiroBio, Neutrolis, Novartis, NS Pharma, Q32, Regeneron, Sanofi, Sparrow, Takeda, and Vistera; royalties or licenses from UpToDate; and stock or stock options from Kyverna, Q32, and Sparrow. SLM reports grants or contracts from MRC, NIHR, and CSL Vifor; consulting fees from Roche, Sanofi, AbbVie, AstraZeneca, and Pfizer; payment or honoraria for lectures or educational events from Roche, Pfizer, UCB, CSL Vifor, Fresenius Kabi, and Novartis; support for attending meetings from Pfizer; participation on a data safety monitoring board or advisory board for Collaboration for Leadership in Applied Health Research and Care, Haywood Foundation, and GC-SheaLD; a leadership or fiduciary role in the British Society for Rheumatology Clinical Affairs Committee; participation as an investigator on industry-sponsored clinical trials for Sanofi; and infrastructure support from MRC. LB reports payment or honoraria for lectures, presentations, speakers bureaus, manuscript writing, or educational events from Instrumentation Laboratory SPA and AbbVie; and support for attending meetings from AbbVie and Novartis. EB reports payments or honoraria from EULAR and received grants from the Dutch Arthritis Society DAS and the EU/EFPIA/Innovative Medicines Initiative 2 Joint Undertaking Immune-Image grant no 831514. EB is member of the board of the non-profit organisation, Auto-immune Research Hub, in the Netherlands. All other authors declare no competing interests.
