# Supplementary material for: Risk loci involved in giant cell arteritis susceptibility: a genome-wide association study
Source: Lancet Rheumatol. 2024 May 8;6(6):e374–83. doi: 10.1016/S2665-9913(24)00064-X (PMC11108802; doi:10.1016/S2665-9913(24)00064-X)
Supplement: Supplementary appendix [file mmc1.pdf]

SUPPLEMENTARY APPENDIX

Angiogenesis- and NETosis-related risk loci involved in giant cell arteritis susceptibility by a genome-wide association study

Gonzalo Borrego-Yaniz, Lourdes Ortiz-Fernández, Adela Madrid-Paredes, Martin Kerick, José Hernández-Rodríguez, Sarah L. Mackie, Augusto Vaglio, Santos Castañeda, Roser Solans, Jaume Mestre-Torres, Nader Khalidi, Carol A. Langford, Steven Ytterberg, Lorenzo Beretta, Marcello Govoni, Giacomo Emmi, Marco A. Cimmino, Torsten Witte, Thomas Neumann, Julia Holle, Verena Schönau, Gregory Puignet, Thomas Papo, Julien Haroche, Alfred Mahr, Luc Mouthon, Øyvind Molberg, Andreas P. Diamantopoulos, Alexandre Voskuyl, Thomas Daikeler, Christoph T. Berger, Eamonn S. Molloy, Daniel Blockmans, Yannick van Sleen, Mark Iles, Louise Sorensen, Raashid Luqmani, Gary Reynolds, Marwan Bukhari, Shweta Bhagat, Spanish GCA Group, UK GCA Consortium, Vasculitis Clinical Research Consortium, Norberto Ortego-Centeno, Elisabeth Brouwer, Peter Lamprecht, Sebastian Klapa, Carlo Salvarani, Peter A. Merkel, María C. Cid, Miguel A. González-Gay, Ann W. Morgan, Javier Martin and Ana Márquez.

SUPPLEMENTARY APPENDIX

Supplementary methods

Estimation of explained heritability 4

Enrichment in epigenetic marks 4

Fine-mapping of associated loci ………………………………………………… 4

Functional annotation and gene prioritisation …………………..……………..… 5

Drug repurposing ………………..…………………………………………….… 5

Genetic risk prediction …..……..……………………………………….…….. 5

References ……………..…...……...……………………………………………….. 6

Supplementary figures

Supp Figure 1 8

Supp Figure 2 9

Supp Figure 3 10

Supplementary tables

Supp Table 1 11

Supp Table 2 12

Supp Table 3 13

Supp Table 4 14

Supp Table 5 15

Supp Table 6 16

Supp Table 7 81

Supp Table 8 82

Supp Table 9 97

Supp Table 10 98

Supp Table 11 109

Supp Table 12 112

Supp Table 13 137

Supp Table 14 138

Supp Table 15 139

Members of the GCA consortia

Spanish GCA Group ……………..……………………………………………… 140

UK GCA Consortium ……………..……………………………………….…… 141

Vasculitis Clinical Research Consortium ….……………………………..……. 146

Supplementary methods

Estimation of explained heritability. Restricted maximum likelihood (REML) analysis using Fisher algorithm was implemented in GCTA64 to estimate the proportion of phenotypic variance explained by studied SNPs. Principal components (PCs) and country of origin were included as covariates to adjust for population structure and potential environmental confounders. This analysis was repeated excluding variants within the HLA region to explore the specific contribution of HLA and non-HLA loci to disease heritability.

Enrichment in epigenetic marks. To infer the possible biological function of the associated loci, we performed regulatory enrichment analysis including both the suggestive and significant SNPs. We used the tool GoShifter[1], which allows us to calculate the statistical enrichment in a set of SNPs given a functional annotation. Specifically, we tested functional annotations from the Roadmap Epigenomics Consortium[2], which includes a variety of histone marks related to active promoter regions (H3K9ac, H3K4me2, H3K4me3, H3K4ac), accessible or active genes (H3K79me1, H2BK15ac) and active enhancers (H3K27ac, H3K3me1, H2BK20ac). Only functional annotations from vascular and immune tissues or cell types were included.

Fine-mapping of associated loci. All non-HLA significant signals were fine-mapped using PAINTOR[3] to perform an exhaustive analysis of the potential causality of associated SNPs. PAINTOR performs probabilistic inference and computes posterior probabilities (PP) for SNPs to be causal considering the strength of association (Z score) and the LD pattern across genomic regions. Additionally, PAINTOR leverages functional annotation data as a prior probability to improve SNP prioritisation. Finally, the method uses Bayes theorem to obtain PP for SNPs to be causal, which in turn are used to generate 95% credible sets (the smallest list of variants that jointly have a probability of including the causal variant ≥95%). For the PAINTOR fine-mapping we included any histone mark annotation that showed significant enrichment in the GoShifter analysis, as well as expression quantitative trait loci (eQTLs) and splicing quantitative trait loci (sQTL) data from immune and arterial tissues from GTEx[4].

Functional annotation and gene prioritisation. To prioritise potential genes affected by the associated SNPs, we conducted gene mapping of the significant associations, using the SNP2GENE function from FUMA[5]. Only those SNPs included in the previously established credible sets were considered. This tool uses a collection of epigenomic and transcriptomic databases to evaluate the implication of the SNPs on the regulation of potential causal genes, or their physical chromatin interaction with a specific gene. FUMA parameters are shown in the appendix (p 14). We also evaluated complementary information from Open Target Genetics[6]. We considered items from three main categories: the physical position of the variant regarding the proposed gene, reported effects of the variant over the expression of the proposed gene or protein, and reported chromatin interaction between the SNP and the proposed gene. These considered items are displayed in the appendix (p 15). Only coding genes and those genes scoring at least once in any category were considered candidate genes.

Drug repurposing. Drug repurposing was conducted to propose potential new treatment options for GCA. For this analysis, we included those candidate genes prioritised from the gene mapping analysis. Moreover, to identify genes interacting with the proposed genes, we calculated the protein-protein interaction (PPi) network using STRING[7], with a minimum required interaction score of 0.70 (high confidence) and excluding "text mining" as a source of interaction prediction. We then searched the DrugBank[8] database for therapies targeting any of these genes. To propose potential treatments for GCA, we focused on the mechanism of action of the different drugs as reported in DrugBank and we also performed a manual literature search.

Genetic risk prediction. We defined a polygenic risk score (PRS) to predict the genetic predisposition of an individual to develop GCA. First, we split the genomic data into a training set (9 cohorts; 2,464 cases and 14,256 controls), which was used to develop the prediction model, and a test set (Spanish cohort; 1,034 cases and 1,294 controls) to evaluate the prediction ability of the model. Based on the association results considering the training set, we extracted the suggestive and significant SNPs to develop a PRS. First, we conducted a clumping analysis on these variants to identify groups of linked SNPs (r^2>0.2). Subsequently, only independent SNPs were included in the models. We used the score function of PLINK v1.90 to calculate the number of

effect alleles per individual, each weighted by the logarithm of the OR. To avoid an overestimation of the weight of the HLA association, only the HLA variants identified as independent in our conditional analysis were included in the model. Additionally, we tested sex as a possible covariate of the model. After developing the PRS using the data of the training set, we calculated the prediction ability of the model on the test set, defining the predictor area-under-the-curve (AUC) using the pROC R package[9]. Furthermore, we assessed the capability of the model to identify high-risk individuals by setting various risk percentile thresholds and examining the statistical significance of each division through a χ^2 test.

References

1. Trynka G, Westra HJ, Slowikowski K, Hu X, Xu H, et al. Disentangling the Effects of Colocalizing Genomic Annotations to Functionally Prioritize Non-coding Variants within Complex-Trait Loci. Am J Hum Genet. 2015 Jul 2;97(1):139–52.
2. Kundaje A, Meuleman W, Ernst J, Bilenky M, Yen A, Heravi-Moussavi A, et al. Integrative analysis of 111 reference human epigenomes. Nature. 2015 Feb 18;518(7539):317–30.
3. Kichaev G, Yang WY, Lindstrom S, Hormozdiari F, Eskin E, Price AL, et al. Integrating functional data to prioritize causal variants in statistical fine-mapping studies. PLoS Genet. 2014 Oct;10(10):e1004722.
4. Lonsdale J, Thomas J, Salvatore M, Phillips R, Lo E, Shad S, et al. The Genotype-Tissue Expression (GTEx) project. Nat Genet. 2013 May 29;45(6):580–5.
5. Watanabe K, Taskesen E, van Bochoven A, Posthuma D. Functional mapping and annotation of genetic associations with FUMA. Nat Commun. 2017 Nov 28;8(1):1826.
6. Ghoussaini M, Mountjoy E, Carmona M, Peat G, Schmidt EM, Hercules A, et al. Open Targets Genetics: systematic identification of trait-associated genes using large-scale genetics and functional genomics. Nucleic Acids Res. 2021 Jan 8;49(D1):D1311–20.
7. Szklarczyk D, Gable AL, Lyon D, Junge A, Wyder S, Huerta-Cepas J, et al. STRING v11: protein-protein association networks with increased coverage, supporting functional discovery in genome-wide experimental datasets. Nucleic

Acids Res. 2019 Jan 8;47(D1):D607–13.
8. Wishart DS, Feunang YD, Guo AC, Lo EJ, Marcu A, Grant JR, et al. DrugBank 5.0: a major update to the DrugBank database for 2018. Nucleic Acids Res. 2018 Jan 4;46(D1):D1074–82.
9. Robin X, Turck N, Hainard A, Tiberti N, Lisacek F, Sanchez JC, et al. pROC: an open-source package for R and S+ to analyze and compare ROC curves. BMC Bioinformatics. 2011 Mar 17;12:77.

Supplementary figures

Supplementary Figure 1

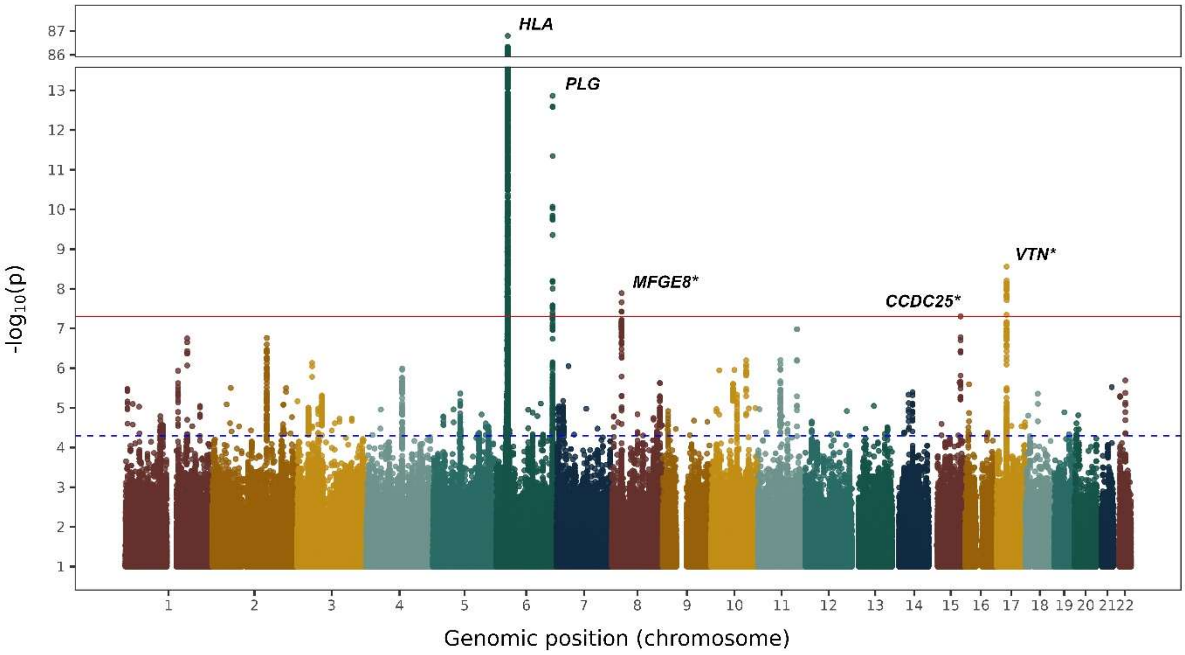

Manhattan plot of the GWAS results. The -log10 of the inverse variance weighted meta-analyses p values are plotted against their physical chromosomal position. The red line represents the significant level of association (p<5x10-8) while the blue line represents the suggestive level (p<5x10-5). *Associations not previously described.

Supplementary Figure 2

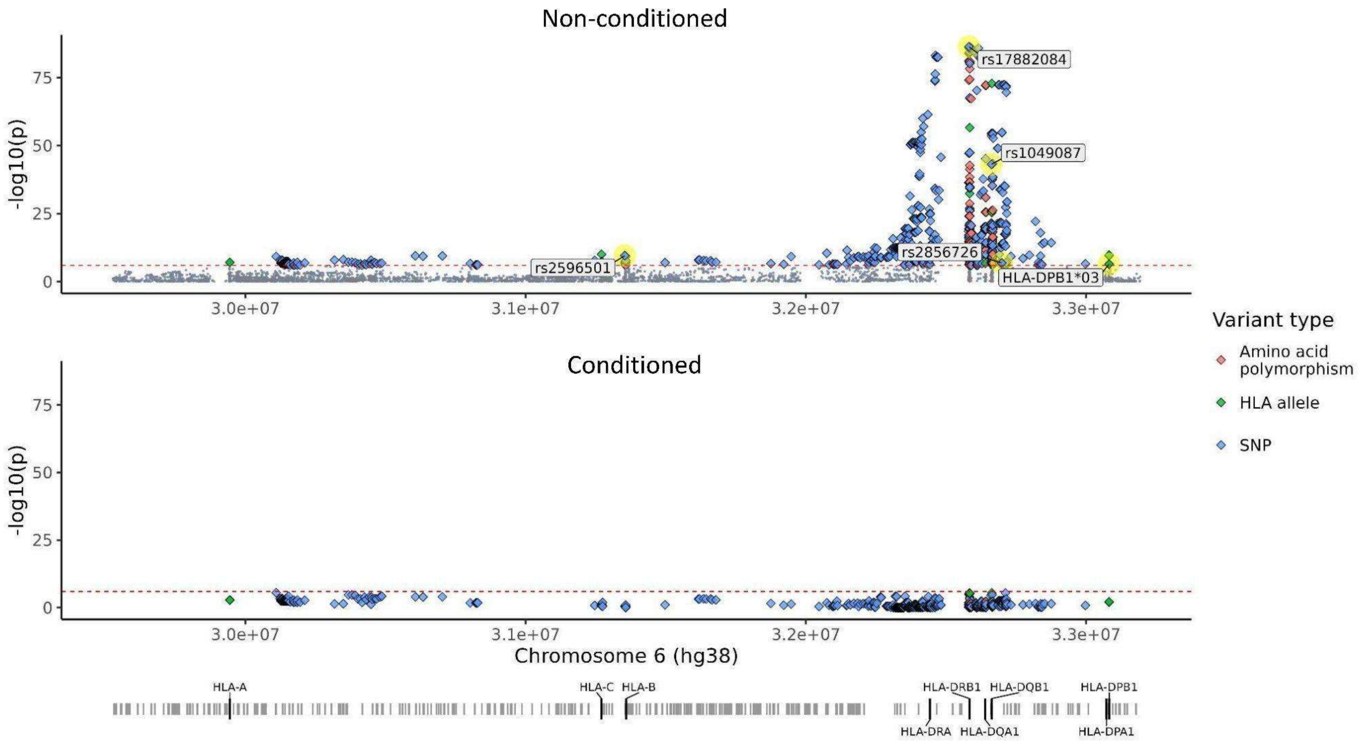

Regional plot of the imputed HLA variants. On the top, the association results before conditional analysis are shown. The five independent variants, determined by the conditional analysis, are highlighted. On the bottom, the association results after conditioning for the five independent variants. The variant type is marked by colour and the red line marks the established significant threshold ($p<1x10^{-6}$).

Supplementary Figure 3

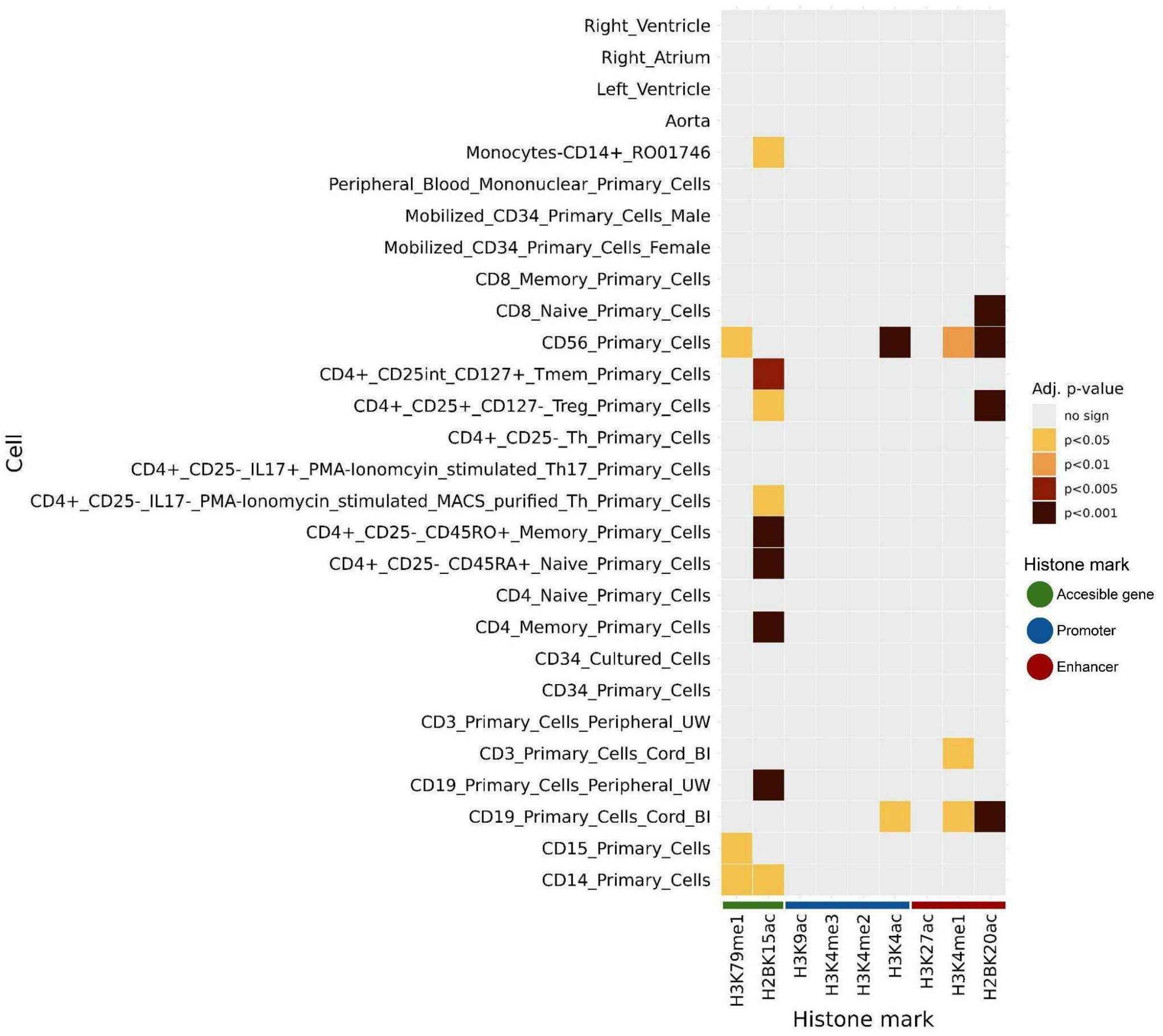

Tile plot of the histone mark enrichment analysis for immune cell types and vascular tissues from the ROADMAP database. The significance of the enrichment (adjusted p-value) is represented by the tile colour, while the colour on the horizontal axis indicates histone marks associated with accessible genes (green), promoters (blue), and enhancers (red).

Supplementary Tables

Supplementary Table 1. Summary of the cohorts included in the study.

Cohort	Cases			Controls			Total size (cases/controls)	Sex Cases		Sex Controls		SNPs after imputation	λ	λ_1000
	Genotype Platform	N before QC	N final	Genotype Platform	N before QC	N final		N male (%)	N female (%)	N male (%)	N female (%)			
United Kingdom	Illumina Infinium Global Screening Array	996	962	Affymetrix 6.0; Illumina 1.2 M SNP	5272	5153	1,306 / 5,153	416 (31.9%)	890 (68.1%)	2600 (50.5%)	2553 (49.5%)	6,086,676	1.020	1.010
	Illumina HumanCore	355*	344											
Spain	Illumina Infinium Global Screening Array	284	259	Illumina HumanCore	1326*	1294	1,034 / 1,294	316 (30.6%)	718 (69.4%)	413 (31.9%)	881 (68.1%)	4,749,490	1.032	1.028
	Illumina HumanCore	819*	775											
Italy	Illumina HumanCore	283*	266	Illumina HumanHap550K	960*	955	266 / 955	56 (21.1%)	210 (78.9%)	325 (34%)	630 (66%)	4,086,853	1.026	-
Germany	Illumina Infinium Global Screening Array	90	87	Illumina HumanOmniExpressExome-8 v1.2	1269	1128	251 / 1,790	53 (21.1%)	198 (78.9%)	720 (40.2%)	1070 (59.8%)	5,250,480	0.976	0.945
	Illumina HumanCore	167*	164	Illumina HumanHap550K	670*	662								
North America	Illumina HumanCore	336*	213	Illumina HumanHap550K	4977	3483	213 / 3,483	58 (27.2%)	155 (72.8%)	419 (12%)	3064 (88%)	6,450,152	0.988	0.971
The Netherlands	Illumina Infinium Global Screening Array	82	76	Illumina HumanHap550K	960	846	138 / 1,484	45 (32.6%)	93 (67.4%)	811 (54.6%)	673 (45.4%)	5,257,574	0.993	-
	Illumina HumanCore	84*	62	Illumina HumanHap550K	643*	638								
France	Illumina HumanCore	118*	106	Illumina HumanHap610K	488*	488	106 / 488	36 (34.0%)	70 (66.0%)	91 (18.6%)	397 (81.4%)	5,295,396	0.959	-
Norway	Illumina HumanCore	116*	104	Illumina HumanHap550K	122*	121	104 / 121	23 (22.1%)	81 (77.9%)	53 (43.8%)	68 (56.2%)	5,475,558	0.981	-
Switzerland	Illumina HumanCore	59*	44	Illumina HumanHap550K	500*	500	44 / 500	19 (43.2%)	25 (56.8%)	208 (41.6%)	292 (58.4%)	5,216,959	0.974	-
Ireland	Illumina HumanCore	84*	36	Illumina HumanCore	288*	282	36 / 282	19 (43.2%)	25 (56.8%)	208 (41.6%)	292 (58.4%)	6,444,129	0.974	-

* Dataset included in previous GWAS in GCA
λ and λ-1000 values were calculated excluding the HLA region

Supplementary Table 2. Eigenvalues of the principal components calculated for each cohort.

	Spain	Germany	Netherlands	North America	Italy	United Kingdom	Norway	France	Ireland	Switzerland
**PC1**	1.880	2.115	1.678	10.539	2.247	2.414	1.242	2.364	2.286	2.691
**PC2**	1.660	1.549	1.455	2.981	1.440	2.351	1.120	1.731	1.458	1.356
**PC3**	1.506	1.498	1.412	2.952	1.347	1.687	1.113	1.340	1.131	1.264
**PC4**	1.429	1.359	1.384	2.688	1.314	1.660	1.109	1.240	1.120	1.243
**PC5**	1.421	1.348	1.375	2.023	1.302	1.638	1.103	1.216	1.114	1.192
**PC6**	1.415	1.345	1.352	1.710	1.296	1.627	1.099	1.212	1.109	1.185
**PC7**	1.405	1.343	1.350	1.454	1.295	1.619	1.097	1.206	1.106	1.181
**PC8**	1.402	1.341	1.345	1.424	1.292	1.617	1.096	1.198	1.105	1.178
**PC9**	1.401	1.339	1.338	1.407	1.287	1.610	1.094	1.193	1.104	1.176
**PC10**	1.397	1.338	1.335	1.395	1.287	1.608	1.091	1.190	1.103	1.176

PC, Principal component

Supplementary Table 3. Statistical power of the study.

MAF	OR=1.2	OR=1.3	OR=1.4	OR=1.5	OR=1.6	OR=1.7	OR=1.8	OR=1.9	OR=2
**0.05**	0.008	0.138	0.562	0.907	0.992	1	1	1	1
**0.1**	0.091	0.677	0.983	1	1	1	1	1	1
**0.15**	0.272	0.93	1	1	1	1	1	1	1
**0.2**	0.468	0.986	1	1	1	1	1	1	1
**0.3**	0.718	0.999	1	1	1	1	1	1	1
**0.4**	0.806	1	1	1	1	1	1	1	1
**0.5**	0.806	1	1	1	1	1	1	1	1

MAF, minor allele frequency; OR, odds ratio

Supplementary Table 4. FUMA SNP2GENE parameters

Field	Parameter	Value
Versions	FUMA	v1.5.1
	MAGMA	v1.08
	GWAScatalog	e0_r2022-11-29
	ANNOVAR	2017-07-17
General parameters	N	19048
	Ncol	NA
	exMHC	1
	MHCopt	annot
	extMHC	NA
	ensembl	v92
	genetype	all
	leadP	1.00E-05
	gwasP	0.05
	r2	0.8
	r2_2	0.1
	refpanel	1KG/Phase3
	pop	EUR
	MAF	0.01
	refSNPs	0
	mergeDist	250
Positional mapping	posMap	1
	posMapWindowSize	10
	posMapAnnot	NA
	posMapCADDth	0
	posMapRDBth	NA
	posMapChr15	NA
	posMapChr15Max	NA
	posMapChr15Meth	NA
	posMapAnnoDs	NA
	posMapAnnoMeth	NA
eQTL mapping	eqtlMap	1
	eqtlMaptss	eQTLcatalogue/BLUEPRINT_ge_monocyte.txt.gz:eQTLcatalogue/BLUEPRINT_ge_neutrophil.txt.gz:eQTLcatalogue/BLUEPRINT_ge_T-cell.txt.gz:eQTLcatalogue/CEDAR_monocyte_CD14.txt.gz:eQTLcatalogue/CEDAR_neutrophil_CD15.txt.gz:eQTLcatalogue/CEDAR_T-cell_CD4.txt.gz:eQTLcatalogue/CEDAR_T-cell_CD8.txt.gz:scRNA_eQTLs/B_cell.txt.gz:scRNA_eQTLs/DC.txt.gz:scRNA_eQTLs/NK.txt.gz:scRNA_eQTLs/Monocyte.txt.gz:scRNA_eQTLs/Classical_Monocyte.txt.gz:scRNA_eQTLs/Non_classical_Monocyte.txt.gz:scRNA_eQTLs/T_CD4.txt.gz:scRNA_eQTLs/T_CD8.txt.gz:scRNA_eQTLs/PBMC.txt.gz:DICE/B_cell_naive.txt.gz:DICE/T_CD4_naive.txt.gz:DICE/T_CD4_naive_activated.txt.gz:DICE/T_CD8_naive.txt.gz:DICE/T_CD8_naive_activated.txt.gz:DICE/Monocyte_classical.txt.gz:DICE/Monocyte_non_classical.txt.gz:DICE/NK.txt.gz:DICE/T_CD4_TFH.txt.gz:DICE/T_CD4_TH1.txt.gz:DICE/T_CD4_TH17.txt.gz:DICE/T_CD4_TH1_17.txt.gz:DICE/T_CD4_TH2.txt.gz:DICE/T_CD4_memory_TREG.txt.gz:DICE/T_CD4_naive_TREG.txt.gz:eQTLGen/eQTLGen_cis_eQTLs.txt.gz:eQTLGen/eQTLGen_trans_eQTLs.txt.gz:BIOSQTL/BIOS_eQTL_geneLevel.txt.gz:GTEx/v8/Cells_EBV-transformed_lymphocytes.txt.gz:GTEx/v8/Whole_Blood.txt.gz:GTEx/v8/Artery_Aorta.txt.gz:GTEx/v8/Artery_Coronary.txt.gz
	eqtlMapSig	1
	eqtlMapP	1
	eqtlMapCADDth	0
	eqtlMapRDBth	NA
	eqtlMapChr15	NA
	eqtlMapChr15Max	NA
	eqtlMapChr15Meth	NA
	eqtlMapAnnoDs	NA
	eqtlMapAnnoMeth	NA
Chromatin interaction mapping	ciMap	1
	ciMapBuiltin	HiC/GSE87112/Aorta.txt.gz:HiC/GSE87112/GM12878.txt.gz
	ciMapFileN	0
	ciMapFiles	NA
	ciMapFDR	1.00E-06
	ciMapPromWindow	250-500
	ciMapRoadmap	E029:E030:E031:E032:E033:E034:E035:E036:E037:E038:E039:E040:E041:E042:E043:E044:E045:E046:E047:E048:E050:E051:E062:E065
	ciMapEnhFilt	0
	ciMapPromFilt	0
	ciMapCADDth	0
	ciMapRDBth	NA
	ciMapChr15	NA
	ciMapChr15Max	NA
	ciMapChr15Meth	NA
	ciMapAnnoDs	NA
	ciMapAnnoMeth	NA

Supplementary Table 5. Items considered for causal gene prioritisation

Item name	Disclosed definition	Data source
Within gene body	Any credible set SNP is located within the genomic position of the proposed gene	FUMA
Missense variant	Any credible set SNP is a missense variant for the proposed gene	FUMA
Physically near	The lead SNP is physically close (10kb max) to the proposed gene	FUMA
Blood eQTL	Any credible set SNP is a reported eQTL for any whole-blood sample	FUMA
Immune cell eQTL	Any credible set SNP is a reported eQTL for any immune cell-specific annotation	FUMA
Vascular tissue eQTL	Any credible set SNP is a reported eQTL for any vascular tissue annotation	FUMA
pQTL	The lead SNP is a reported pQTL for the proposed gene	Open Targets Genetics
Chromatin interaction	Any credible set SNP has chromatin interaction evidence for 3D physical interaction between the SNP region and the proposed gene region	FUMA
Highest V2G Score	Highest Variant-to-Gene score from Open Targets Genetics for the lead SNP-proposed gene pairs. All values are displayed when available	Open Targets Genetics

Supplementary Table 6. Genetic variants showing genome-wide significant association with GCA after meta-analysis.

CHR	BP (hg38)	SNP	Nearest gene	Variant type	ID	Change	Meta-analysis				
							N	P	OR	Q	I
6	32444703	chr6:32444703:G:A	HLA-DRA	3' UTR	rs71941	G>A	10	5.57x10-14	0.79 [0.75-0.84]	0.777	0
6	32444762	chr6:32444762:A:G	HLA-DRA	3' UTR	rs7195	A>G	10	5.57x10-14	0.79 [0.75-0.84]	0.777	0
6	32444794	chr6:32444794:A:T	HLA-DRA	3' UTR	rs7196	A>T	10	2.28x10-12	0.76 [0.71-0.82]	0.452	0
6	32444803	chr6:32444803:T:C	HLA-DRA	3' UTR	rs7197	T>C	10	1.07x10-11	0.74 [0.69-0.81]	0.17	29.97
6	32643170	chr6:32643170:A:C	HLA-DQA1	3' UTR	rs1130153	A>C	9	1.30x10-08	0.61 [0.51-0.72]	0.808	0
6	32643393	chr6:32643393:C:A	HLA-DQA1	3' UTR	rs9272958	C>A	10	8.10x10-20	0.57 [0.51-0.64]	0.006	61.15
6	32643452	chr6:32643452:G:T	HLA-DQA1	3' UTR	rs9272962	G>T	10	8.10x10-20	0.57 [0.51-0.64]	0.006	61.15
6	32643457	chr6:32643457:C:T	HLA-DQA1	3' UTR	rs1065043	C>T	10	6.42x10-83	1.78 [1.69-1.88]	0.171	29.78
6	32643466	chr6:32643466:C:T	HLA-DQA1	3' UTR	rs1065044	C>T	10	6.42x10-83	1.78 [1.69-1.88]	0.171	29.78
6	32643488	chr6:32643488:C:T	HLA-DQA1	3' UTR	rs1065048	C>T	10	6.42x10-83	1.78 [1.69-1.88]	0.171	29.78
6	32643673	chr6:32643673:G:T	HLA-DQA1	3' UTR	rs7757696	G>T	10	8.55x10-20	0.57 [0.51-0.64]	0.006	61.23
6	32659602	chr6:32659602:T:C	HLA-DQB1	3' UTR	rs1130456	T>C	10	3.38x10-26	0.67 [0.63-0.72]	0.574	0
6	32659613	chr6:32659613:T:C	HLA-DQB1	3' UTR	rs9273423	T>C	10	1.18x10-26	0.67 [0.62-0.71]	0.574	0
6	32659784	chr6:32659784:T:C	HLA-DQB1	3' UTR	rs9273440	T>C	10	1.17x10-26	0.67 [0.62-0.71]	0.572	0
6	32659796	chr6:32659796:C:G	HLA-DQB1	3' UTR	rs9273441	C>G	10	7.61x10-20	0.57 [0.51-0.64]	0.006	61.07
6	32659805	chr6:32659805:T:G	HLA-DQB1	3' UTR	rs9273442	T>G	10	1.21x10-26	0.67 [0.62-0.71]	0.573	0
6	32659823	chr6:32659823:G:T	HLA-DQB1	3' UTR	rs9273443	G>T	10	7.61x10-20	0.57 [0.51-0.64]	0.006	61.07
6	32659858	chr6:32659858:T:G	HLA-DQB1	3' UTR	rs9273444	T>G	10	7.83x10-20	0.57 [0.51-0.64]	0.006	61.09
6	32659869	chr6:32659869:A:G	HLA-DQB1	3' UTR	rs9273445	A>G	10	7.83x10-20	0.57 [0.51-0.64]	0.006	61.09
6	32659875	chr6:32659875:C:G	HLA-DQB1	3' UTR	rs4993986	C>G	10	2.02x10-14	1.59 [1.42-1.78]	0.002	65.04
6	32659923	chr6:32659923:A:G	HLA-DQB1	3' UTR	rs6689	A>G	9	1.38x10-10	0.55 [0.46-0.66]	0.001	68.37
6	32659937	chr6:32659937:T:G	HLA-DQB1	3' UTR	rs1063355	T>G	10	7.61x10-20	0.57 [0.51-0.64]	0.006	61.07
6	32659970	chr6:32659970:A:G	HLA-DQB1	3' UTR	rs1049225	A>G	10	1.21x10-26	0.67 [0.62-0.71]	0.573	0
6	32659996	chr6:32659996:A:G	HLA-DQB1	3' UTR	rs1049213	A>G	10	8.15x10-26	0.68 [0.63-0.73]	0.776	0
6	32660000	chr6:32660000:G:C	HLA-DQB1	3' UTR	rs1762	G>C	10	5.12x10-55	1.73 [1.62-1.84]	0.361	8.83
6	32813180	chr6:32813180:C:T	HLA-DOB	3' UTR	rs41258084	C>T	10	3.93x10-09	1.47 [1.30-1.66]	0.311	14.37
6	32828533	chr6:32828533:A:G	TAP2	3' UTR	rs115360810	A>G	6	1.45x10-22	2.28 [1.94-2.67]	0.911	0
6	32371771	chr6:32371771:G:A	TSBP1	5' UTR	rs2073045	G>A	9	1.87x10-24	1.48 [1.38-1.60]	0.085	42.43
6	32628195	chr6:32628195:G:A	HLA-DQA1	5' UTR	rs9271898	G>A	10	1.72x10-33	1.42 [1.34-1.50]	0.859	0
6	32628230	chr6:32628230:A:G	HLA-DQA1	5' UTR	rs9271899	A>G	10	1.68x10-33	1.42 [1.34-1.50]	0.859	0
6	32628236	chr6:32628236:A:G	HLA-DQA1	5' UTR	rs9271900	A>G	10	4.87x10-09	0.84 [0.79-0.89]	0.165	30.48
6	32628250	chr6:32628250:C:T	HLA-DQA1	5' UTR	rs9271901	C>T	10	1.68x10-33	1.42 [1.34-1.50]	0.859	0
6	32632085	chr6:32632085:T:G	HLA-DQA1	5' UTR	rs11751908	T>G	10	3.53x10-36	0.63 [0.59-0.68]	0.243	21.7
6	32666610	chr6:32666610:C:T	HLA-DQB1	5' UTR	rs1049055	C>T	10	1.21x10-26	0.67 [0.62-0.71]	0.573	0
6	32666648	chr6:32666648:C:T	HLA-DQB1	5' UTR	rs1049070	C>T	10	3.18x10-55	1.73 [1.62-1.84]	0.325	12.86
6	32853670	chr6:32853670:C:T	TAP1	5' UTR	rs2071536	C>T	10	1.34x10-21	1.49 [1.38-1.61]	0.056	45.62
6	31281490	chr6:31281490:G:C	RPL3P2	Exonic	rs35840219	G>C	9	1.52x10-10	1.42 [1.28-1.58]	0.629	0
6	31468553	chr6:31468553:T:C	HCP5	Exonic	rs62395310	T>C	9	8.60x10-13	1.67 [1.46-1.91]	0.486	0
6	32264632	chr6:32264632:C:T	TSBP1-AS1	Exonic	rs9268071	C>T	8	2.84x10-08	0.59 [0.50-0.71]	0.176	31.54
6	32265684	chr6:32265684:C:G	TSBP1-AS1	Exonic	rs2395113	C>G	8	2.65x10-08	0.60 [0.50-0.71]	0.253	22.18
6	32396039	chr6:32396039:T:C	BTNL2	Exonic	rs2076530	T>C	10	1.04x10-22	1.33 [1.26-1.40]	0.085	40.91
6	32396067	chr6:32396067:C:T	BTNL2	Exonic	rs9268480	C>T	9	1.84x10-51	1.61 [1.52-1.70]	0.154	33.03
6	32396178	chr6:32396178:T:C	BTNL2	Exonic	rs2076529	T>C	10	1.11x10-23	1.34 [1.27-1.41]	0.106	37.94
6	32459971	chr6:32459971:C:T	HLA-DRB9	Exonic	rs9268831	C>T	10	1.25x10-10	0.72 [0.65-0.79]	0.023	53.27
6	32460012	chr6:32460012:T:C	HLA-DRB9	Exonic	rs9268832	T>C	10	6.16x10-18	0.77 [0.72-0.81]	0.508	0

6	32552906	chr6:32552906:G:A	HLA-DRB6	Exonic	rs66935282	G>A	10	1.51x10-15	0.69 [0.63-0.76]	0.206	25.8
6	32552930	chr6:32552930:G:C	HLA-DRB6	Exonic	rs66723041	G>C	10	1.28x10-15	0.69 [0.63-0.75]	0.202	26.28
6	32552952	chr6:32552952:G:A	HLA-DRB6	Exonic	rs66758173	G>A	10	2.06x10-13	0.70 [0.64-0.77]	0.432	0.6
6	32553008	chr6:32553008:T:G	HLA-DRB6	Exonic	rs61615404	T>G	10	1.77x10-13	0.70 [0.64-0.77]	0.423	1.72
6	32553039	chr6:32553039:G:C	HLA-DRB6	Exonic	rs58495148	G>C	10	2.12x10-13	0.70 [0.64-0.77]	0.413	2.88
6	32554793	chr6:32554793:G:A	HLA-DRB6	Exonic	rs35923382	G>A	10	4.60x10-17	0.78 [0.73-0.82]	0.425	1.48
6	32706143	chr6:32706143:C:T	MTCO3P1	Exonic	rs9275501	C>T	10	7.06x10-15	1.29 [1.21-1.37]	0.497	0
6	32706144	chr6:32706144:A:G	MTCO3P1	Exonic	rs9275502	A>G	10	7.06x10-15	1.29 [1.21-1.37]	0.497	0
6	32706154	chr6:32706154:A:G	MTCO3P1	Exonic	rs9275503	A>G	9	1.17x10-65	2.07 [1.92-2.24]	0.953	0
6	32706209	chr6:32706209:C:T	MTCO3P1	Exonic	rs9275504	C>T	10	7.06x10-15	1.29 [1.21-1.37]	0.497	0
6	32706250	chr6:32706250:C:G	MTCO3P1	Exonic	rs3134969	C>G	10	1.66x10-19	0.69 [0.64-0.75]	0.746	0
6	32706281	chr6:32706281:A:G	MTCO3P1	Exonic	rs9275505	A>G	9	2.22x10-18	1.35 [1.27-1.44]	0.709	0
6	32706282	chr6:32706282:A:G	MTCO3P1	Exonic	rs3134968	A>G	10	1.66x10-19	0.69 [0.64-0.75]	0.746	0
6	32706288	chr6:32706288:T:C	MTCO3P1	Exonic	rs1794268	T>C	5	1.06x10-10	0.41 [0.31-0.53]	0.904	0
6	32706306	chr6:32706306:G:A	MTCO3P1	Exonic	rs9275506	G>A	9	1.70x10-18	1.35 [1.27-1.44]	0.715	0
6	32706353	chr6:32706353:G:A	MTCO3P1	Exonic	rs9275507	G>A	10	8.83x10-15	1.29 [1.21-1.37]	0.485	0
6	32706382	chr6:32706382:C:G	MTCO3P1	Exonic	rs9275508	C>G	10	7.06x10-15	1.29 [1.21-1.37]	0.497	0
6	32706510	chr6:32706510:A:G	MTCO3P1	Exonic	rs9275510	A>G	9	1.83x10-18	1.35 [1.27-1.44]	0.712	0
6	32706521	chr6:32706521:T:C	MTCO3P1	Exonic	rs3134966	T>C	10	2.28x10-26	0.66 [0.62-0.71]	0.462	0
6	32706552	chr6:32706552:G:A	MTCO3P1	Exonic	rs9275511	G>A	10	8.46x10-25	0.74 [0.70-0.78]	0.051	46.65
6	32706619	chr6:32706619:A:G	MTCO3P1	Exonic	rs9275512	A>G	9	2.02x10-18	1.35 [1.27-1.44]	0.725	0
6	32706787	chr6:32706787:A:T	MTCO3P1	Exonic	rs9275513	A>T	10	6.50x10-36	0.68 [0.64-0.72]	0.151	32.13
6	32706789	chr6:32706789:T:A	MTCO3P1	Exonic	rs9275514	T>A	10	1.68x10-35	0.68 [0.64-0.72]	0.168	30.14
6	32706796	chr6:32706796:T:C	MTCO3P1	Exonic	rs9275515	T>C	10	5.15x10-36	0.68 [0.64-0.72]	0.148	32.49
6	32706866	chr6:32706866:A:G	MTCO3P1	Exonic	rs9275516	A>G	10	6.50x10-36	0.68 [0.64-0.72]	0.151	32.13
6	32706872	chr6:32706872:A:G	MTCO3P1	Exonic	rs9275517	A>G	10	6.50x10-36	0.68 [0.64-0.72]	0.151	32.13
6	32706923	chr6:32706923:G:A	MTCO3P1	Exonic	rs9275518	G>A	10	3.94x10-37	0.67 [0.64-0.71]	0.141	33.39
6	31679224	chr6:31679224:A:G	LY6G5C	xonic (missens	rs11575852	A>G	6	2.01x10-08	1.71 [1.42-2.06]	0.43	0
6	32058330	chr6:32058330:C:T	TNXB	xonic (missens	rs1009382	C>T	9	9.76x10-15	0.76 [0.71-0.81]	0.444	0
6	32222613	chr6:32222613:T:G	NOTCH4	xonic (missens	rs915894	T>G	10	3.47x10-10	1.20 [1.14-1.27]	0.421	1.92
6	32330595	chr6:32330595:A:G	TSBP1	xonic (missens	rs9405090	A>G	10	4.00x10-13	1.24 [1.17-1.31]	0.276	18.16
6	32339605	chr6:32339605:G:A	TSBP1	xonic (missens	rs1033500	G>A	10	3.36x10-13	1.24 [1.17-1.31]	0.261	19.82
6	32366178	chr6:32366178:T:C	TSBP1	xonic (missens	rs9268368	T>C	10	3.29x10-13	1.24 [1.17-1.31]	0.26	19.86
6	32368809	chr6:32368809:A:G	TSBP1	xonic (missens	rs9268384	A>G	10	3.29x10-13	1.24 [1.17-1.31]	0.26	19.86
6	32443869	chr6:32443869:T:G	HLA-DRA	xonic (missens	rs7192	T>G	10	5.31x10-14	0.79 [0.75-0.84]	0.776	0
6	32061449	chr6:32061449:A:G	TNXB	nic (synonymo	rs204887	A>G	10	8.76x10-10	0.82 [0.77-0.87]	0.55	0
6	32064966	chr6:32064966:G:A	TNXB	nic (synonymo	rs204883	G>A	10	1.11x10-10	1.21 [1.14-1.27]	0.799	0
6	31084584	chr6:31084584:G:A	RNU6-1133P	Intergenic	rs34507986	G>A	9	1.17x10-09	1.42 [1.27-1.59]	0.939	0
6	31210307	chr6:31210307:C:G	-	Intergenic	rs3130513	C>G	10	1.54x10-08	0.85 [0.81-0.90]	0.723	0
6	31217172	chr6:31217172:G:A	-	Intergenic	rs35990442	G>A	9	5.32x10-09	1.39 [1.24-1.54]	0.936	0
6	31240250	chr6:31240250:C:T	-	Intergenic	rs3132498	C>T	10	1.66x10-08	1.18 [1.11-1.24]	0.991	0
6	31241011	chr6:31241011:A:T	-	Intergenic	rs3130533	A>T	10	2.55x10-09	1.19 [1.12-1.26]	0.991	0
6	31241612	chr6:31241612:G:A	-	Intergenic	rs3095247	G>A	10	7.62x10-09	1.18 [1.12-1.25]	0.878	0
6	31242083	chr6:31242083:T:C	-	Intergenic	rs3134767	T>C	10	1.56x10-08	1.18 [1.11-1.25]	0.991	0
6	31242824	chr6:31242824:C:T	-	Intergenic	rs3130689	C>T	10	1.56x10-08	1.18 [1.11-1.25]	0.991	0
6	31243720	chr6:31243720:C:T	-	Intergenic	rs3130698	C>T	10	1.66x10-08	1.18 [1.11-1.24]	0.985	0

6	31246034	chr6:31246034:G:A	-	Intergenic	rs3130409	G>A	10	1.56x10-08	1.18 [1.11-1.25]	0.991	0
6	31246125	chr6:31246125:T:C	-	Intergenic	rs3095245	T>C	10	1.56x10-08	1.18 [1.11-1.25]	0.991	0
6	31246780	chr6:31246780:C:T	-	Intergenic	rs2394902	C>T	10	1.56x10-08	1.18 [1.11-1.25]	0.991	0
6	31248523	chr6:31248523:C:T	-	Intergenic	rs3134755	C>T	10	2.55x10-09	1.19 [1.12-1.26]	0.991	0
6	31248552	chr6:31248552:A:G	-	Intergenic	rs3134754	A>G	10	1.52x10-08	1.18 [1.11-1.25]	0.991	0
6	31249163	chr6:31249163:C:A	-	Intergenic	rs3130417	C>A	10	1.56x10-08	1.18 [1.11-1.25]	0.991	0
6	31251580	chr6:31251580:G:A	-	Intergenic	rs3130432	G>A	10	1.56x10-08	1.18 [1.11-1.25]	0.991	0
6	31251692	chr6:31251692:C:T	-	Intergenic	rs3134749	C>T	10	1.56x10-08	1.18 [1.11-1.25]	0.991	0
6	31252145	chr6:31252145:C:T	-	Intergenic	rs9264181	C>T	10	5.97x10-09	1.18 [1.12-1.25]	0.965	0
6	31252157	chr6:31252157:A:G	-	Intergenic	rs9264183	A>G	10	5.97x10-09	1.18 [1.12-1.25]	0.965	0
6	31252168	chr6:31252168:G:A	-	Intergenic	rs9264184	G>A	10	6.05x10-09	1.18 [1.12-1.25]	0.964	0
6	31268564	chr6:31268564:C:T	HLA-C	Intergenic	rs34247821	C>T	10	4.94x10-08	1.26 [1.16-1.36]	0.374	7.4
6	31268623	chr6:31268623:G:A	HLA-C	Intergenic	rs34634498	G>A	10	4.69x10-08	1.26 [1.16-1.36]	0.375	7.24
6	31268633	chr6:31268633:C:T	HLA-C	Intergenic	rs34131062	C>T	10	4.69x10-08	1.26 [1.16-1.36]	0.375	7.24
6	31272654	chr6:31272654:G:T	HLA-C	Intergenic	rs2074488	G>T	10	4.98x10-08	1.26 [1.16-1.36]	0.374	7.39
6	31273037	chr6:31273037:C:T	HLA-C	Intergenic	rs34622008	C>T	10	4.90x10-08	1.26 [1.16-1.36]	0.372	7.59
6	31273300	chr6:31273300:T:C	HLA-C	Intergenic	rs35976302	T>C	10	4.98x10-08	1.26 [1.16-1.36]	0.374	7.39
6	31273576	chr6:31273576:G:T	HLA-C	Intergenic	rs34090104	G>T	10	4.98x10-08	1.26 [1.16-1.36]	0.374	7.39
6	31273823	chr6:31273823:G:A	HLA-C	Intergenic	rs34255701	G>A	10	4.98x10-08	1.26 [1.16-1.36]	0.374	7.39
6	31273944	chr6:31273944:A:G	HLA-C	Intergenic	rs35028179	A>G	10	4.98x10-08	1.26 [1.16-1.36]	0.374	7.39
6	31281834	chr6:31281834:G:A	RPL3P2	Intergenic	rs35193884	G>A	9	2.00x10-10	1.42 [1.28-1.57]	0.606	0
6	31303926	chr6:31303926:A:T	-	Intergenic	rs11962586	A>T	9	1.92x10-10	1.42 [1.28-1.58]	0.606	0
6	31332181	chr6:31332181:A:G	-	Intergenic	rs34972021	A>G	8	9.45x10-10	1.42 [1.27-1.58]	0.88	0
6	31353434	chr6:31353434:C:T	HLA-B	Intergenic	rs2596501	C>T	10	1.12x10-10	1.21 [1.14-1.28]	0.982	0
6	31353774	chr6:31353774:A:G	HLA-B	Intergenic	rs75456009	A>G	10	3.92x10-08	0.80 [0.75-0.87]	0.815	0
6	31353782	chr6:31353782:C:A	HLA-B	Intergenic	rs74615740	C>A	10	3.92x10-08	0.80 [0.75-0.87]	0.815	0
6	31353786	chr6:31353786:A:C	HLA-B	Intergenic	rs75256987	A>C	10	4.13x10-08	0.81 [0.75-0.87]	0.816	0
6	31353800	chr6:31353800:C:T	HLA-B	Intergenic	rs114322933	C>T	10	3.75x10-08	0.80 [0.75-0.87]	0.814	0
6	31358533	chr6:31358533:A:G	HLA-B	Intergenic	rs142279800	A>G	4	2.26x10-11	2.28 [1.80-2.88]	0.594	0
6	31479228	chr6:31479228:C:G	HCP5	Intergenic	rs2248477	C>G	7	4.67x10-08	1.69 [1.40-2.03]	0.411	1.75
6	31550392	chr6:31550392:A:G	ATP6V1G2	Intergenic	rs2844492	A>G	7	3.05x10-08	1.70 [1.42-2.05]	0.433	0
6	31563096	chr6:31563096:G:A	NFKBIL1	Intergenic	rs138240887	G>A	9	2.00x10-16	1.93 [1.66-2.25]	0.274	19.02
6	31605800	chr6:31605800:T:C	-	Intergenic	rs144360625	T>C	10	3.79x10-08	1.26 [1.16-1.37]	0.841	0
6	31617847	chr6:31617847:C:T	AIF1	Intergenic	rs6927872	C>T	10	9.35x10-09	1.27 [1.17-1.38]	0.629	0
6	31620161	chr6:31620161:A:G	AIF1	Intergenic	rs3763295	A>G	10	8.37x10-09	1.27 [1.17-1.38]	0.655	0
6	31634712	chr6:31634712:T:C	BAG6	Intergenic	rs2242657	T>C	10	1.55x10-08	1.26 [1.17-1.37]	0.61	0
6	31659672	chr6:31659672:C:G	Y_RNA	Intergenic	rs2242655	C>G	10	1.87x10-08	1.26 [1.17-1.37]	0.62	0
6	31667421	chr6:31667421:T:C	Y_RNA	Intergenic	rs35149168	T>C	10	8.13x10-14	1.53 [1.37-1.70]	0.672	0
6	31710914	chr6:31710914:C:T	LY6G6D	Intergenic	rs17207370	C>T	6	1.03x10-08	1.73 [1.44-2.09]	0.453	0
6	31879641	chr6:31879641:G:C	SLC44A4	Intergenic	rs116369005	G>C	6	7.34x10-23	2.37 [2.01-2.80]	0.749	0
6	31896761	chr6:31896761:G:A	ZBTB12	Intergenic	rs115884658	G>A	6	3.88x10-08	1.73 [1.43-2.10]	0.131	41.16
6	31905515	chr6:31905515:C:T	ZBTB12	Intergenic	rs2844454	C>T	8	4.98x10-08	1.47 [1.28-1.69]	0.36	9.07
6	31947158	chr6:31947158:A:G	C2	Intergenic	rs1048709	A>G	9	1.47x10-11	0.73 [0.67-0.80]	0.733	0
6	31955454	chr6:31955454:T:A	SKIV2L	Intergenic	rs372313241	T>A	9	2.39x10-28	2.21 [1.93-2.53]	0.808	0
6	32146738	chr6:32146738:A:T	PRRT1	Intergenic	rs9296009	A>T	10	2.93x10-12	1.28 [1.20-1.37]	0.32	13.33
6	32152121	chr6:32152121:T:C	PRRT1	Intergenic	rs3131283	T>C	7	2.77x10-08	0.73 [0.66-0.82]	0.795	0

6	32167360	chr6:32167360:G:A	PPT2	Intergenic	rs41268924	G>A	8	4.11x10-08	1.48 [1.29-1.70]	0.387	5.66
6	32170768	chr6:32170768:A:C	EGFL8	Intergenic	rs3130283	A>C	7	1.71x10-08	0.73 [0.66-0.82]	0.767	0
6	32184217	chr6:32184217:A:T	PBX2	Intergenic	rs1800684	A>T	7	1.37x10-08	0.73 [0.65-0.81]	0.785	0
6	32187804	chr6:32187804:A:G	GPSM3	Intergenic	rs204993	A>G	10	4.56x10-10	1.22 [1.15-1.30]	0.263	19.52
6	32228158	chr6:32228158:T:C	NOTCH4	Intergenic	rs3132949	T>C	9	4.04x10-10	0.82 [0.77-0.87]	0.359	9.15
6	32239616	chr6:32239616:G:T	-	Intergenic	rs416352	G>T	10	4.81x10-08	0.85 [0.80-0.90]	0.619	0
6	32240182	chr6:32240182:T:C	-	Intergenic	rs393481	T>C	10	3.68x10-08	0.84 [0.80-0.90]	0.715	0
6	32242084	chr6:32242084:C:T	-	Intergenic	rs507778	C>T	10	4.22x10-09	0.84 [0.79-0.89]	0.518	0
6	32243308	chr6:32243308:T:G	-	Intergenic	rs412657	T>G	10	3.65x10-12	0.81 [0.77-0.86]	0.872	0
6	32244878	chr6:32244878:C:T	-	Intergenic	rs9267951	C>T	9	1.92x10-10	1.32 [1.21-1.44]	0.078	43.5
6	32251543	chr6:32251543:G:T	TSBP1-AS1	Intergenic	rs9267989	G>T	10	1.66x10-12	1.55 [1.38-1.75]	0.034	50.32
6	32266037	chr6:32266037:C:T	TSBP1-AS1	Intergenic	rs3115562	C>T	8	3.07x10-08	0.60 [0.50-0.72]	0.285	18.36
6	32267607	chr6:32267607:C:G	TSBP1-AS1	Intergenic	rs2114436	C>G	8	2.67x10-08	0.60 [0.50-0.71]	0.252	22.3
6	32268277	chr6:32268277:G:T	TSBP1-AS1	Intergenic	rs3132933	G>T	8	2.93x10-08	0.59 [0.50-0.71]	0.176	31.61
6	32269868	chr6:32269868:G:C	TSBP1-AS1	Intergenic	rs2023223	G>C	8	2.73x10-08	0.60 [0.50-0.71]	0.252	22.27
6	32285767	chr6:32285767:T:C	TSBP1	Intergenic	rs6902465	T>C	8	1.83x10-08	0.59 [0.50-0.71]	0.269	20.24
6	32287217	chr6:32287217:G:A	TSBP1	Intergenic	rs9268133	G>A	8	1.94x10-08	0.59 [0.49-0.71]	0.19	29.85
6	32289507	chr6:32289507:T:G	TSBP1	Intergenic	rs9268145	T>G	10	4.94x10-44	1.61 [1.51-1.72]	0.177	29.1
6	32292493	chr6:32292493:G:C	TSBP1	Intergenic	rs565448	G>C	8	1.83x10-08	0.59 [0.50-0.71]	0.269	20.24
6	32372120	chr6:32372120:C:T	TSBP1	Intergenic	rs2050188	C>T	10	5.53x10-24	0.73 [0.69-0.77]	0.066	43.85
6	32372291	chr6:32372291:C:T	TSBP1	Intergenic	rs1967688	C>T	10	2.60x10-15	1.50 [1.36-1.66]	0.022	53.5
6	32372293	chr6:32372293:C:T	TSBP1	Intergenic	rs1980496	C>T	10	1.51x10-48	1.54 [1.45-1.62]	0.059	45.11
6	32372329	chr6:32372329:A:G	TSBP1	Intergenic	rs2281276	A>G	10	1.44x10-25	1.35 [1.28-1.43]	0.246	21.37
6	32372459	chr6:32372459:G:A	TSBP1	Intergenic	rs9268399	G>A	10	5.61x10-19	1.54 [1.41-1.69]	0.048	47.29
6	32372877	chr6:32372877:G:A	TSBP1	Intergenic	rs9268400	G>A	9	5.70x10-51	1.60 [1.51-1.70]	0.112	38.38
6	32373541	chr6:32373541:A:G	TSBP1	Intergenic	rs9268401	A>G	9	4.13x10-51	1.60 [1.51-1.70]	0.119	37.47
6	32373576	chr6:32373576:G:A	TSBP1	Intergenic	rs9268402	G>A	10	1.48x10-27	1.37 [1.30-1.45]	0.204	26.08
6	32373696	chr6:32373696:T:C	TSBP1	Intergenic	rs9268403	T>C	9	4.22x10-51	1.60 [1.51-1.70]	0.12	37.4
6	32373942	chr6:32373942:C:T	TSBP1	Intergenic	rs9268404	C>T	9	4.13x10-51	1.60 [1.51-1.70]	0.119	37.47
6	32374310	chr6:32374310:C:T	TSBP1	Intergenic	rs9268405	C>T	9	4.04x10-51	1.61 [1.51-1.70]	0.119	37.54
6	32374352	chr6:32374352:C:T	TSBP1	Intergenic	rs9268406	C>T	9	4.13x10-51	1.60 [1.51-1.70]	0.119	37.47
6	32374389	chr6:32374389:T:C	TSBP1	Intergenic	rs9268407	T>C	9	4.13x10-51	1.60 [1.51-1.70]	0.119	37.47
6	32374862	chr6:32374862:A:C	TSBP1	Intergenic	rs9268409	A>C	9	4.04x10-51	1.61 [1.51-1.70]	0.119	37.54
6	32374914	chr6:32374914:C:A	TSBP1	Intergenic	rs7746327	C>A	10	2.78x10-27	1.37 [1.30-1.45]	0.176	29.26
6	32375045	chr6:32375045:C:G	TSBP1	Intergenic	rs3117108	C>G	10	7.71x10-19	0.75 [0.70-0.80]	0.055	45.92
6	32375241	chr6:32375241:T:A	TSBP1	Intergenic	rs9268412	T>A	9	3.74x10-51	1.61 [1.51-1.70]	0.118	37.65
6	32375318	chr6:32375318:T:C	TSBP1	Intergenic	rs9268413	T>C	9	4.55x10-51	1.60 [1.51-1.70]	0.121	37.29
6	32375319	chr6:32375319:G:A	TSBP1	Intergenic	rs9268414	G>A	9	4.13x10-51	1.60 [1.51-1.70]	0.119	37.47
6	32375490	chr6:32375490:G:C	TSBP1	Intergenic	rs9268415	G>C	9	4.13x10-51	1.60 [1.51-1.70]	0.119	37.47
6	32375607	chr6:32375607:A:G	TSBP1	Intergenic	rs9268416	A>G	9	4.22x10-51	1.60 [1.51-1.70]	0.12	37.4
6	32375839	chr6:32375839:A:G	TSBP1	Intergenic	rs9268417	A>G	9	4.17x10-51	1.60 [1.51-1.70]	0.118	37.62
6	32375909	chr6:32375909:T:C	TSBP1	Intergenic	rs9268418	T>C	9	4.36x10-51	1.60 [1.51-1.70]	0.119	37.48
6	32375937	chr6:32375937:T:C	TSBP1	Intergenic	rs3117105	T>C	10	8.45x10-19	0.75 [0.70-0.80]	0.055	45.78
6	32376019	chr6:32376019:C:T	TSBP1	Intergenic	rs9268420	C>T	9	4.22x10-51	1.60 [1.51-1.70]	0.12	37.4
6	32376092	chr6:32376092:A:G	TSBP1	Intergenic	rs9268421	A>G	9	4.22x10-51	1.60 [1.51-1.70]	0.12	37.4
6	32376096	chr6:32376096:G:A	TSBP1	Intergenic	rs9268422	G>A	9	4.22x10-51	1.60 [1.51-1.70]	0.12	37.4

6	32376105	chr6:32376105:C:T	TSBP1	Intergenic	rs9268423	C>T	9	4.22x10-51	1.60 [1.51-1.70]	0.12	37.4
6	32376208	chr6:32376208:T:C	TSBP1	Intergenic	rs9268424	T>C	9	4.68x10-51	1.60 [1.51-1.70]	0.119	37.49
6	32376599	chr6:32376599:C:T	TSBP1	Intergenic	rs9268425	C>T	9	9.22x10-52	1.61 [1.52-1.71]	0.124	36.87
6	32386651	chr6:32386651:A:G	TSBP1-AS1	Intergenic	rs4424066	A>G	10	7.84x10-24	1.34 [1.27-1.41]	0.104	38.15
6	32386867	chr6:32386867:C:A	TSBP1-AS1	Intergenic	rs3129948	C>A	10	8.19x10-19	0.75 [0.70-0.80]	0.051	46.54
6	32387828	chr6:32387828:G:A	TSBP1-AS1	Intergenic	rs9268472	G>A	10	7.95x10-24	1.34 [1.27-1.41]	0.104	38.15
6	32387906	chr6:32387906:A:G	TSBP1-AS1	Intergenic	rs9268473	A>G	10	7.84x10-24	1.34 [1.27-1.41]	0.104	38.15
6	32389388	chr6:32389388:T:C	TSBP1-AS1	Intergenic	rs9268474	T>C	9	8.09x10-52	1.61 [1.52-1.71]	0.159	32.32
6	32390454	chr6:32390454:C:T	BTNL2	Intergenic	rs9268475	C>T	9	8.09x10-52	1.61 [1.52-1.71]	0.159	32.32
6	32390509	chr6:32390509:T:C	BTNL2	Intergenic	rs3129951	T>C	10	6.80x10-19	0.75 [0.70-0.80]	0.056	45.74
6	32390736	chr6:32390736:G:A	BTNL2	Intergenic	rs3117098	G>A	10	6.76x10-19	0.75 [0.70-0.80]	0.057	45.49
6	32391165	chr6:32391165:T:A	BTNL2	Intergenic	rs9268476	T>A	10	5.02x10-24	1.34 [1.27-1.42]	0.125	35.4
6	32391344	chr6:32391344:G:A	BTNL2	Intergenic	rs9268477	G>A	9	7.33x10-52	1.61 [1.52-1.71]	0.158	32.52
6	32391986	chr6:32391986:G:C	BTNL2	Intergenic	rs3129952	G>C	10	6.76x10-19	0.75 [0.70-0.80]	0.057	45.49
6	32392481	chr6:32392481:G:A	BTNL2	Intergenic	rs9268478	G>A	9	9.79x10-52	1.61 [1.52-1.71]	0.152	33.21
6	32393334	chr6:32393334:C:T	BTNL2	Intergenic	rs3817973	C>T	10	6.74x10-24	1.34 [1.27-1.41]	0.111	37.17
6	32394639	chr6:32394639:T:G	BTNL2	Intergenic	rs2076534	T>G	10	1.77x10-14	0.74 [0.69-0.80]	0.865	0
6	32407503	chr6:32407503:C:G	BTNL2	Intergenic	rs9268492	C>G	10	3.06x10-14	1.48 [1.34-1.64]	0.031	50.97
6	32407553	chr6:32407553:G:A	BTNL2	Intergenic	rs9268493	G>A	10	3.06x10-14	1.48 [1.34-1.64]	0.031	50.97
6	32407575	chr6:32407575:A:C	BTNL2	Intergenic	rs9268494	A>C	9	1.43x10-11	1.46 [1.32-1.63]	0.018	56.57
6	32407594	chr6:32407594:A:T	BTNL2	Intergenic	rs9268495	A>T	9	1.43x10-11	1.46 [1.32-1.63]	0.018	56.57
6	32407597	chr6:32407597:G:A	BTNL2	Intergenic	rs9268496	G>A	9	1.43x10-11	1.46 [1.32-1.63]	0.018	56.57
6	32407647	chr6:32407647:G:A	BTNL2	Intergenic	rs9268497	G>A	9	1.43x10-11	1.46 [1.32-1.63]	0.018	56.57
6	32407918	chr6:32407918:G:A	BTNL2	Intergenic	rs9268499	G>A	10	7.90x10-17	1.52 [1.38-1.67]	0.048	47.21
6	32407968	chr6:32407968:G:A	BTNL2	Intergenic	rs6926737	G>A	10	2.10x10-24	0.74 [0.70-0.78]	0.209	25.51
6	32408196	chr6:32408196:C:A	BTNL2	Intergenic	rs3763309	C>A	10	1.30x10-52	1.67 [1.57-1.77]	0.242	21.87
6	32408399	chr6:32408399:C:T	BTNL2	Intergenic	rs3763311	C>T	10	2.25x10-17	1.53 [1.39-1.68]	0.049	46.98
6	32408571	chr6:32408571:G:A	BTNL2	Intergenic	rs3763312	G>A	10	2.62x10-52	1.66 [1.56-1.77]	0.234	22.67
6	32408740	chr6:32408740:C:T	BTNL2	Intergenic	rs9268500	C>T	8	6.16x10-27	1.87 [1.68-2.09]	0.519	0
6	32408841	chr6:32408841:C:T	BTNL2	Intergenic	rs3763314	C>T	10	2.71x10-24	0.74 [0.70-0.78]	0.216	24.73
6	32408969	chr6:32408969:C:T	BTNL2	Intergenic	rs3763316	C>T	9	1.86x10-51	1.61 [1.52-1.70]	0.165	31.64
6	32409011	chr6:32409011:C:T	BTNL2	Intergenic	rs3763317	C>T	10	5.45x10-19	1.30 [1.23-1.37]	0.288	16.85
6	32409105	chr6:32409105:T:G	BTNL2	Intergenic	rs9268501	T>G	10	2.41x10-24	0.74 [0.70-0.78]	0.204	26.04
6	32409155	chr6:32409155:C:T	BTNL2	Intergenic	rs9268502	C>T	10	1.69x10-24	0.74 [0.70-0.78]	0.208	25.58
6	32409284	chr6:32409284:C:T	BTNL2	Intergenic	rs9268503	C>T	10	2.20x10-24	0.74 [0.70-0.78]	0.212	25.14
6	32409339	chr6:32409339:C:G	BTNL2	Intergenic	rs9268504	C>G	10	2.02x10-24	0.74 [0.70-0.78]	0.213	25.05
6	32409693	chr6:32409693:C:T	BTNL2	Intergenic	rs9268506	C>T	10	2.32x10-24	0.74 [0.70-0.78]	0.209	25.52
6	32409762	chr6:32409762:A:G	BTNL2	Intergenic	rs9268507	A>G	10	2.32x10-24	0.74 [0.70-0.78]	0.209	25.52
6	32409811	chr6:32409811:T:A	BTNL2	Intergenic	rs9268508	T>A	10	2.32x10-24	0.74 [0.70-0.78]	0.209	25.52
6	32411010	chr6:32411010:T:C	BTNL2	Intergenic	rs9268513	T>C	10	2.30x10-24	0.74 [0.70-0.78]	0.21	25.42
6	32411057	chr6:32411057:T:C	BTNL2	Intergenic	rs5007266	T>C	10	2.35x10-24	0.74 [0.70-0.78]	0.21	25.37
6	32411089	chr6:32411089:T:G	BTNL2	Intergenic	rs5007265	T>G	10	2.30x10-24	0.74 [0.70-0.78]	0.21	25.42
6	32411100	chr6:32411100:C:T	BTNL2	Intergenic	rs5007264	C>T	10	2.30x10-24	0.74 [0.70-0.78]	0.21	25.42
6	32411168	chr6:32411168:T:A	BTNL2	Intergenic	rs9268514	T>A	9	2.81x10-51	1.61 [1.51-1.70]	0.132	35.79
6	32411205	chr6:32411205:A:G	BTNL2	Intergenic	rs5007263	A>G	10	2.30x10-24	0.74 [0.70-0.78]	0.21	25.42
6	32411234	chr6:32411234:A:G	BTNL2	Intergenic	rs5007262	A>G	10	2.30x10-24	0.74 [0.70-0.78]	0.21	25.42

6	32411254	chr6:32411254:G:A	BTNL2	Intergenic	rs5007261	G>A	10	2.30x10-24	0.74 [0.70-0.78]	0.21	25.42
6	32411270	chr6:32411270:G:A	BTNL2	Intergenic	rs5007260	G>A	10	1.90x10-24	0.74 [0.70-0.78]	0.205	25.96
6	32411324	chr6:32411324:T:C	BTNL2	Intergenic	rs5007259	T>C	10	2.30x10-24	0.74 [0.70-0.78]	0.21	25.42
6	32411462	chr6:32411462:A:G	BTNL2	Intergenic	rs5007258	A>G	10	2.79x10-24	0.74 [0.70-0.78]	0.215	24.79
6	32411518	chr6:32411518:G:C	BTNL2	Intergenic	rs9268515	G>C	10	1.15x10-55	1.76 [1.65-1.89]	0.275	18.24
6	32411668	chr6:32411668:T:C	BTNL2	Intergenic	rs6906730	T>C	10	3.60x10-24	0.74 [0.70-0.79]	0.231	23.09
6	32411712	chr6:32411712:C:T	BTNL2	Intergenic	rs9268516	C>T	9	3.81x10-51	1.60 [1.51-1.70]	0.137	35.11
6	32411905	chr6:32411905:T:C	BTNL2	Intergenic	rs6911383	T>C	10	2.79x10-24	0.74 [0.70-0.78]	0.215	24.79
6	32412413	chr6:32412413:G:A	TSBP1-AS1	Intergenic	rs6932810	G>A	10	2.79x10-24	0.74 [0.70-0.78]	0.215	24.79
6	32412485	chr6:32412485:A:G	TSBP1-AS1	Intergenic	rs6932542	A>G	10	2.01x10-24	0.74 [0.70-0.78]	0.208	25.6
6	32412938	chr6:32412938:C:T	-	Intergenic	rs968155	C>T	10	4.20x10-17	1.28 [1.21-1.35]	0.075	42.49
6	32413005	chr6:32413005:T:A	-	Intergenic	rs4502931	T>A	10	2.01x10-24	0.74 [0.70-0.78]	0.208	25.6
6	32413008	chr6:32413008:G:A	-	Intergenic	rs968154	G>A	10	2.01x10-24	0.74 [0.70-0.78]	0.208	25.6
6	32413041	chr6:32413041:A:G	-	Intergenic	rs9268518	A>G	10	2.01x10-24	0.74 [0.70-0.78]	0.208	25.6
6	32413049	chr6:32413049:G:A	-	Intergenic	rs9268519	G>A	10	2.01x10-24	0.74 [0.70-0.78]	0.208	25.6
6	32413083	chr6:32413083:C:A	-	Intergenic	rs9268520	C>A	10	2.01x10-24	0.74 [0.70-0.78]	0.208	25.6
6	32413264	chr6:32413264:C:G	-	Intergenic	rs6938337	C>G	10	2.40x10-24	0.74 [0.70-0.78]	0.204	26.07
6	32413433	chr6:32413433:T:C	-	Intergenic	rs6918317	T>C	10	3.27x10-22	0.75 [0.71-0.79]	0.168	30.11
6	32413597	chr6:32413597:G:C	-	Intergenic	rs9268521	G>C	10	3.69x10-53	1.66 [1.56-1.77]	0.254	20.57
6	32413666	chr6:32413666:A:T	-	Intergenic	rs9268522	A>T	10	4.57x10-53	1.66 [1.56-1.76]	0.248	21.16
6	32413684	chr6:32413684:C:T	-	Intergenic	rs3129967	C>T	10	4.49x10-08	0.84 [0.80-0.90]	0.247	21.33
6	32413959	chr6:32413959:T:A	-	Intergenic	rs7759742	T>A	10	5.47x10-23	0.75 [0.71-0.79]	0.156	31.61
6	32417024	chr6:32417024:A:T	-	Intergenic	rs9268543	A>T	10	9.90x10-61	1.84 [1.72-1.97]	0.508	0
6	32420032	chr6:32420032:T:C	-	Intergenic	rs2395163	T>C	10	9.15x10-58	1.71 [1.61-1.82]	0.435	0.32
6	32425129	chr6:32425129:C:A	-	Intergenic	rs3135352	C>A	9	6.27x10-12	0.69 [0.62-0.76]	0.586	0
6	32425204	chr6:32425204:C:T	-	Intergenic	rs3135350	C>T	9	8.10x10-12	0.69 [0.62-0.76]	0.592	0
6	32425458	chr6:32425458:G:C	-	Intergenic	rs3129971	G>C	9	6.27x10-12	0.69 [0.62-0.76]	0.586	0
6	32427259	chr6:32427259:C:T	-	Intergenic	rs3135344	C>T	10	5.69x10-17	0.75 [0.71-0.80]	0.06	45.04
6	32427864	chr6:32427864:T:C	-	Intergenic	rs9268576	T>C	8	7.78x10-12	0.63 [0.55-0.72]	0.758	0
6	32428058	chr6:32428058:A:G	-	Intergenic	rs3129844	A>G	10	1.86x10-11	0.80 [0.75-0.85]	0.838	0
6	32428500	chr6:32428500:A:G	-	Intergenic	rs3129845	A>G	10	4.76x10-12	0.79 [0.75-0.85]	0.884	0
6	32428536	chr6:32428536:C:T	-	Intergenic	rs3135343	C>T	10	4.76x10-12	0.79 [0.75-0.85]	0.884	0
6	32428698	chr6:32428698:A:G	-	Intergenic	rs3129846	A>G	10	4.76x10-12	0.79 [0.75-0.85]	0.884	0
6	32428729	chr6:32428729:A:G	-	Intergenic	rs3129847	A>G	10	1.78x10-11	0.80 [0.75-0.85]	0.838	0
6	32428838	chr6:32428838:G:T	-	Intergenic	rs3135342	G>T	10	1.86x10-11	0.80 [0.75-0.85]	0.838	0
6	32429153	chr6:32429153:G:A	-	Intergenic	rs9268581	G>A	10	6.16x10-62	1.74 [1.63-1.85]	0.5	0
6	32429272	chr6:32429272:G:A	-	Intergenic	rs3129848	G>A	10	1.78x10-11	0.80 [0.75-0.85]	0.834	0
6	32429935	chr6:32429935:A:C	-	Intergenic	rs3129851	A>C	10	1.78x10-11	0.80 [0.75-0.85]	0.834	0
6	32430871	chr6:32430871:G:A	-	Intergenic	rs3129853	G>A	10	1.78x10-11	0.80 [0.75-0.85]	0.834	0
6	32431095	chr6:32431095:T:G	-	Intergenic	rs3135340	T>G	10	2.18x10-11	0.80 [0.75-0.85]	0.818	0
6	32431198	chr6:32431198:C:T	-	Intergenic	rs4988822	C>T	10	2.05x10-11	0.80 [0.75-0.85]	0.834	0
6	32431245	chr6:32431245:A:G	-	Intergenic	rs4988821	A>G	10	1.78x10-11	0.80 [0.75-0.85]	0.834	0
6	32431381	chr6:32431381:C:T	-	Intergenic	rs4988820	C>T	10	1.87x10-11	0.80 [0.75-0.85]	0.836	0
6	32431410	chr6:32431410:C:A	-	Intergenic	rs4321864	C>A	10	2.37x10-38	1.48 [1.40-1.56]	0.067	43.79
6	32431484	chr6:32431484:C:G	-	Intergenic	rs3135339	C>G	10	1.80x10-11	0.80 [0.75-0.85]	0.836	0
6	32432008	chr6:32432008:A:G	-	Intergenic	rs3129857	A>G	10	1.77x10-11	0.80 [0.75-0.85]	0.833	0

6	32432065	chr6:32432065:T:C	-	Intergenic	rs2395172	T>C	10	1.73x10-11	0.80 [0.75-0.85]	0.831	0
6	32432743	chr6:32432743:G:A	-	Intergenic	rs3129858	G>A	10	1.77x10-11	0.80 [0.75-0.85]	0.833	0
6	32433162	chr6:32433162:G:C	-	Intergenic	rs3129859	G>C	10	7.00x10-09	0.83 [0.78-0.88]	0.464	0
6	32433302	chr6:32433302:A:G	-	Intergenic	rs3129860	A>G	10	1.78x10-12	0.71 [0.65-0.78]	0.555	0
6	32433757	chr6:32433757:C:T	-	Intergenic	rs3135337	C>T	10	1.77x10-11	0.80 [0.75-0.85]	0.833	0
6	32435001	chr6:32435001:T:G	HLA-DRA	Intergenic	rs9268614	T>G	10	2.87x10-62	1.74 [1.64-1.85]	0.511	0
6	32435878	chr6:32435878:T:G	HLA-DRA	Intergenic	rs983561	T>G	10	2.04x10-11	0.80 [0.75-0.85]	0.827	0
6	32436157	chr6:32436157:C:T	HLA-DRA	Intergenic	rs3135398	C>T	10	1.77x10-11	0.80 [0.75-0.85]	0.833	0
6	32436266	chr6:32436266:A:G	HLA-DRA	Intergenic	rs3129864	A>G	10	1.77x10-11	0.80 [0.75-0.85]	0.833	0
6	32436271	chr6:32436271:G:C	HLA-DRA	Intergenic	rs3129865	G>C	10	1.87x10-12	0.71 [0.65-0.78]	0.557	0
6	32436358	chr6:32436358:A:G	HLA-DRA	Intergenic	rs5000563	A>G	10	1.77x10-11	0.80 [0.75-0.85]	0.833	0
6	32436436	chr6:32436436:C:T	HLA-DRA	Intergenic	rs3135396	C>T	10	1.77x10-11	0.80 [0.75-0.85]	0.833	0
6	32436600	chr6:32436600:A:C	HLA-DRA	Intergenic	rs3129868	A>C	10	1.87x10-12	0.71 [0.65-0.78]	0.557	0
6	32437101	chr6:32437101:T:G	HLA-DRA	Intergenic	rs2395174	T>G	10	1.77x10-11	0.80 [0.75-0.85]	0.833	0
6	32437249	chr6:32437249:G:A	HLA-DRA	Intergenic	rs2395175	G>A	10	6.31x10-64	1.86 [1.74-2.00]	0.529	0
6	32437267	chr6:32437267:G:C	HLA-DRA	Intergenic	rs9268626	G>C	7	2.59x10-08	0.65 [0.56-0.76]	0.734	0
6	32437285	chr6:32437285:G:T	HLA-DRA	Intergenic	rs2395176	G>T	10	2.14x10-11	0.80 [0.75-0.85]	0.825	0
6	32437299	chr6:32437299:G:C	HLA-DRA	Intergenic	rs2395177	G>C	10	2.14x10-11	0.80 [0.75-0.85]	0.825	0
6	32438044	chr6:32438044:T:C	HLA-DRA	Intergenic	rs9268627	T>C	10	2.87x10-62	1.74 [1.64-1.85]	0.511	0
6	32438088	chr6:32438088:A:C	HLA-DRA	Intergenic	rs9268628	A>C	10	2.08x10-11	0.77 [0.71-0.83]	0.274	18.33
6	32438308	chr6:32438308:G:T	HLA-DRA	Intergenic	rs9268629	G>T	8	1.04x10-11	0.63 [0.56-0.72]	0.76	0
6	32438323	chr6:32438323:C:T	HLA-DRA	Intergenic	rs3129870	C>T	10	2.27x10-11	0.80 [0.75-0.85]	0.825	0
6	32438635	chr6:32438635:C:G	HLA-DRA	Intergenic	rs9268632	C>G	10	2.77x10-11	0.80 [0.75-0.85]	0.829	0
6	32438802	chr6:32438802:T:C	HLA-DRA	Intergenic	rs9268635	T>C	9	4.69x10-12	0.68 [0.62-0.76]	0.625	0
6	32439110	chr6:32439110:C:T	HLA-DRA	Intergenic	rs9268641	C>T	10	1.94x10-11	0.80 [0.75-0.85]	0.806	0
6	32439376	chr6:32439376:A:T	HLA-DRA	Intergenic	rs3129872	A>T	10	2.51x10-11	0.80 [0.75-0.85]	0.828	0
6	32439525	chr6:32439525:A:G	HLA-DRA	Intergenic	rs2395179	A>G	10	6.45x10-12	0.80 [0.75-0.85]	0.882	0
6	32439533	chr6:32439533:T:G	HLA-DRA	Intergenic	rs2395180	T>G	10	6.45x10-12	0.80 [0.75-0.85]	0.882	0
6	32439627	chr6:32439627:G:C	HLA-DRA	Intergenic	rs2395181	G>C	10	6.45x10-12	0.80 [0.75-0.85]	0.882	0
6	32439656	chr6:32439656:G:C	HLA-DRA	Intergenic	rs3129873	G>C	10	2.51x10-11	0.80 [0.75-0.85]	0.828	0
6	32439663	chr6:32439663:T:C	HLA-DRA	Intergenic	rs3129874	T>C	10	2.51x10-11	0.80 [0.75-0.85]	0.828	0
6	32439691	chr6:32439691:T:C	HLA-DRA	Intergenic	rs3129875	T>C	10	6.45x10-12	0.80 [0.75-0.85]	0.882	0
6	32445274	chr6:32445274:A:G	HLA-DRA	Intergenic	rs3135388	A>G	9	3.10x10-12	0.68 [0.61-0.76]	0.639	0
6	32445373	chr6:32445373:G:A	HLA-DRA	Intergenic	rs2213585	G>A	10	4.76x10-14	0.79 [0.75-0.84]	0.778	0
6	32445482	chr6:32445482:A:G	HLA-DRA	Intergenic	rs2213584	A>G	10	4.76x10-14	0.79 [0.75-0.84]	0.778	0
6	32445540	chr6:32445540:G:T	HLA-DRA	Intergenic	rs2395182	G>T	10	2.12x10-12	0.76 [0.71-0.82]	0.447	0
6	32445682	chr6:32445682:G:A	HLA-DRA	Intergenic	rs2227139	G>A	10	4.20x10-14	0.79 [0.75-0.84]	0.774	0
6	32445768	chr6:32445768:G:A	HLA-DRA	Intergenic	rs3129889	G>A	9	2.67x10-12	0.68 [0.61-0.76]	0.635	0
6	32446053	chr6:32446053:G:C	HLA-DRA	Intergenic	rs3763327	G>C	10	3.63x10-14	0.79 [0.75-0.84]	0.759	0
6	32446112	chr6:32446112:C:A	HLA-DRA	Intergenic	rs9268668	C>A	10	4.89x10-15	0.78 [0.74-0.83]	0.825	0
6	32446510	chr6:32446510:T:C	HLA-DRA	Intergenic	rs9268670	T>C	10	1.88x10-16	0.78 [0.73-0.82]	0.659	0
6	32446643	chr6:32446643:G:A	HLA-DRA	Intergenic	rs7382085	G>A	10	3.69x10-12	0.76 [0.71-0.82]	0.345	10.66
6	32446656	chr6:32446656:C:T	HLA-DRA	Intergenic	rs9268673	C>T	10	7.31x10-29	0.72 [0.69-0.76]	0.065	44.15
6	32446862	chr6:32446862:G:A	HLA-DRA	Intergenic	rs7382354	G>A	10	3.01x10-12	0.76 [0.71-0.82]	0.356	9.4
6	32446891	chr6:32446891:G:A	HLA-DRA	Intergenic	rs9268677	G>A	10	7.85x10-29	0.72 [0.69-0.76]	0.065	44.14
6	32447332	chr6:32447332:T:G	HLA-DRA	Intergenic	rs3135387	T>G	10	3.13x10-10	0.78 [0.72-0.84]	0.267	19.18

6	32447609	chr6:32447609:G:A	HLA-DRA	Intergenic	rs9268681	G>A	10	4.26x10-29	0.72 [0.68-0.76]	0.055	45.77
6	32447645	chr6:32447645:G:T	HLA-DRA	Intergenic	rs9268682	G>T	10	4.26x10-29	0.72 [0.68-0.76]	0.055	45.77
6	32447680	chr6:32447680:G:A	HLA-DRA	Intergenic	rs9268684	G>A	10	5.41x10-29	0.72 [0.68-0.76]	0.059	45.21
6	32447687	chr6:32447687:A:T	HLA-DRA	Intergenic	rs9268685	A>T	10	4.94x10-29	0.72 [0.68-0.76]	0.059	45.19
6	32447745	chr6:32447745:C:T	HLA-DRA	Intergenic	rs9268686	C>T	10	1.69x10-16	0.78 [0.73-0.82]	0.617	0
6	32447763	chr6:32447763:G:T	HLA-DRA	Intergenic	rs9268687	G>T	10	4.74x10-29	0.72 [0.68-0.76]	0.058	45.28
6	32447808	chr6:32447808:C:A	HLA-DRA	Intergenic	rs2894256	C>A	10	4.34x10-29	0.72 [0.68-0.76]	0.055	45.79
6	32448589	chr6:32448589:A:G	HLA-DRA	Intergenic	rs3135385	A>G	10	3.57x10-12	0.76 [0.71-0.82]	0.335	11.77
6	32449049	chr6:32449049:C:T	HLA-DRA	Intergenic	rs9268693	C>T	10	2.74x10-29	0.72 [0.68-0.76]	0.053	46.31
6	32449501	chr6:32449501:G:A	HLA-DRA	Intergenic	rs9268744	G>A	10	3.14x10-29	0.72 [0.68-0.76]	0.057	45.57
6	32449927	chr6:32449927:C:T	HLA-DRA	Intergenic	rs9268759	C>T	10	2.69x10-29	0.72 [0.68-0.76]	0.053	46.28
6	32450241	chr6:32450241:C:T	-	Intergenic	rs6937696	C>T	10	1.38x10-14	0.78 [0.73-0.83]	0.292	16.43
6	32450254	chr6:32450254:A:C	-	Intergenic	rs6937545	A>C	10	1.38x10-14	0.78 [0.73-0.83]	0.292	16.43
6	32450380	chr6:32450380:G:T	-	Intergenic	rs9268762	G>T	10	2.68x10-29	0.72 [0.68-0.76]	0.051	46.56
6	32450784	chr6:32450784:T:C	-	Intergenic	rs9268764	T>C	10	3.09x10-29	0.72 [0.68-0.76]	0.051	46.63
6	32450802	chr6:32450802:A:C	-	Intergenic	rs9268765	A>C	10	1.47x10-16	0.78 [0.73-0.82]	0.581	0
6	32450933	chr6:32450933:C:A	-	Intergenic	rs9268766	C>A	10	1.34x10-14	0.78 [0.73-0.83]	0.292	16.43
6	32451245	chr6:32451245:T:C	-	Intergenic	rs9268767	T>C	10	3.09x10-29	0.72 [0.68-0.76]	0.051	46.63
6	32451514	chr6:32451514:C:T	-	Intergenic	rs9268768	C>T	10	3.09x10-29	0.72 [0.68-0.76]	0.051	46.63
6	32451770	chr6:32451770:G:T	-	Intergenic	rs6912728	G>T	10	3.09x10-29	0.72 [0.68-0.76]	0.051	46.63
6	32452131	chr6:32452131:A:C	-	Intergenic	rs6457588	A>C	10	3.09x10-29	0.72 [0.68-0.76]	0.051	46.63
6	32452182	chr6:32452182:A:G	-	Intergenic	rs3135384	A>G	9	2.60x10-12	0.68 [0.61-0.76]	0.648	0
6	32452184	chr6:32452184:T:G	-	Intergenic	rs6457589	T>G	10	1.47x10-16	0.78 [0.73-0.82]	0.581	0
6	32452402	chr6:32452402:C:T	-	Intergenic	rs7754768	C>T	10	1.59x10-16	0.78 [0.73-0.82]	0.582	0
6	32452422	chr6:32452422:C:T	-	Intergenic	rs9268775	C>T	7	4.46x10-09	0.63 [0.55-0.74]	0.751	0
6	32452461	chr6:32452461:T:C	-	Intergenic	rs7775108	T>C	10	1.47x10-16	0.78 [0.73-0.82]	0.581	0
6	32452595	chr6:32452595:A:T	-	Intergenic	rs9268778	A>T	10	3.09x10-29	0.72 [0.68-0.76]	0.051	46.63
6	32452712	chr6:32452712:G:A	-	Intergenic	rs9268780	G>A	10	3.09x10-29	0.72 [0.68-0.76]	0.051	46.63
6	32452720	chr6:32452720:T:C	-	Intergenic	rs9268781	T>C	10	1.34x10-14	0.78 [0.73-0.83]	0.292	16.43
6	32452735	chr6:32452735:C:T	-	Intergenic	rs7755283	C>T	10	1.34x10-14	0.78 [0.73-0.83]	0.292	16.43
6	32452938	chr6:32452938:T:C	-	Intergenic	rs9268783	T>C	10	1.47x10-16	0.78 [0.73-0.82]	0.581	0
6	32453015	chr6:32453015:G:A	-	Intergenic	rs9268784	G>A	10	3.09x10-29	0.72 [0.68-0.76]	0.051	46.63
6	32453296	chr6:32453296:T:C	-	Intergenic	rs9268791	T>C	10	4.85x10-15	0.78 [0.73-0.83]	0.293	16.36
6	32453377	chr6:32453377:A:G	-	Intergenic	rs9268792	A>G	10	3.09x10-29	0.72 [0.68-0.76]	0.051	46.63
6	32453450	chr6:32453450:A:G	-	Intergenic	rs3129895	A>G	10	2.71x10-13	0.70 [0.64-0.77]	0.546	0
6	32453551	chr6:32453551:G:A	-	Intergenic	rs9268795	G>A	10	3.24x10-29	0.72 [0.68-0.76]	0.054	46.12
6	32453552	chr6:32453552:T:G	-	Intergenic	rs9268796	T>G	10	1.47x10-16	0.78 [0.73-0.82]	0.586	0
6	32453958	chr6:32453958:T:C	-	Intergenic	rs9268798	T>C	10	3.09x10-29	0.72 [0.68-0.76]	0.054	46.12
6	32454348	chr6:32454348:G:A	-	Intergenic	rs3129898	G>A	10	2.66x10-13	0.70 [0.64-0.77]	0.545	0
6	32454482	chr6:32454482:A:C	-	Intergenic	rs2097441	A>C	10	5.12x10-12	0.72 [0.65-0.79]	0.049	47.03
6	32454945	chr6:32454945:T:C	HLA-DRB9	Intergenic	rs4577793	T>C	10	6.80x10-12	0.72 [0.65-0.79]	0.045	47.78
6	32455622	chr6:32455622:A:G	HLA-DRB9	Intergenic	rs9268805	A>G	10	9.39x10-11	0.82 [0.78-0.87]	0.585	0
6	32455832	chr6:32455832:C:A	HLA-DRB9	Intergenic	rs9268806	C>A	10	4.66x10-12	0.72 [0.65-0.79]	0.048	47.15
6	32456138	chr6:32456138:C:G	HLA-DRB9	Intergenic	rs9268807	C>G	10	2.76x10-13	0.70 [0.64-0.77]	0.545	0
6	32456399	chr6:32456399:G:T	HLA-DRB9	Intergenic	rs9268808	G>T	10	6.97x10-12	0.72 [0.65-0.79]	0.045	47.88
6	32456488	chr6:32456488:T:C	HLA-DRB9	Intergenic	rs9268809	T>C	10	6.97x10-12	0.72 [0.65-0.79]	0.045	47.88

6	32456519	chr6:32456519:C:T	HLA-DRB9	Intergenic	rs9268810	C>T	10	6.97x10-12	0.72 [0.65-0.79]	0.045	47.88
6	32456593	chr6:32456593:G:T	HLA-DRB9	Intergenic	rs9268811	G>T	10	6.97x10-12	0.72 [0.65-0.79]	0.045	47.88
6	32456791	chr6:32456791:A:G	HLA-DRB9	Intergenic	rs9268812	A>G	10	1.30x10-14	0.78 [0.73-0.83]	0.277	18.09
6	32456839	chr6:32456839:C:G	HLA-DRB9	Intergenic	rs9268814	C>G	10	7.03x10-12	0.72 [0.65-0.79]	0.045	47.9
6	32456900	chr6:32456900:A:T	HLA-DRB9	Intergenic	rs9268815	A>T	10	1.10x10-16	0.78 [0.73-0.82]	0.559	0
6	32457105	chr6:32457105:T:C	HLA-DRB9	Intergenic	rs7763262	T>C	10	1.46x10-14	0.78 [0.73-0.83]	0.281	17.58
6	32457272	chr6:32457272:G:A	HLA-DRB9	Intergenic	rs7743662	G>A	10	6.92x10-12	0.72 [0.65-0.79]	0.045	47.83
6	32457358	chr6:32457358:C:T	HLA-DRB9	Intergenic	rs7743415	C>T	10	1.38x10-16	0.78 [0.73-0.82]	0.59	0
6	32457620	chr6:32457620:C:A	HLA-DRB9	Intergenic	rs9268820	C>A	10	6.92x10-12	0.72 [0.65-0.79]	0.045	47.83
6	32457710	chr6:32457710:G:T	HLA-DRB9	Intergenic	rs7744304	G>T	10	1.46x10-14	0.78 [0.73-0.83]	0.281	17.58
6	32457828	chr6:32457828:G:A	HLA-DRB9	Intergenic	rs7748310	G>A	10	6.92x10-12	0.72 [0.65-0.79]	0.045	47.83
6	32457965	chr6:32457965:A:G	HLA-DRB9	Intergenic	rs7748085	A>G	10	6.92x10-12	0.72 [0.65-0.79]	0.045	47.83
6	32458137	chr6:32458137:G:C	HLA-DRB9	Intergenic	rs7748825	G>C	10	6.92x10-12	0.72 [0.65-0.79]	0.045	47.83
6	32458206	chr6:32458206:T:C	HLA-DRB9	Intergenic	rs6457592	T>C	10	2.51x10-25	0.73 [0.69-0.78]	0.104	38.23
6	32458681	chr6:32458681:T:G	HLA-DRB9	Intergenic	rs7769693	T>G	10	5.92x10-17	0.77 [0.73-0.82]	0.622	0
6	32458763	chr6:32458763:A:G	HLA-DRB9	Intergenic	rs9268828	A>G	10	8.89x10-30	0.72 [0.68-0.76]	0.052	46.34
6	32458825	chr6:32458825:A:G	HLA-DRB9	Intergenic	rs9268829	A>G	10	8.89x10-30	0.72 [0.68-0.76]	0.052	46.34
6	32459402	chr6:32459402:A:T	HLA-DRB9	Intergenic	rs1548306	A>T	10	1.24x10-14	0.77 [0.73-0.82]	0.295	16.12
6	32459795	chr6:32459795:C:G	HLA-DRB9	Intergenic	rs9268830	C>G	10	2.21x10-09	0.80 [0.74-0.86]	0.259	20
6	32473615	chr6:32473615:A:T	HLA-DRB9	Intergenic	rs17209747	A>T	10	4.38x10-83	1.77 [1.68-1.87]	0.265	19.3
6	32473619	chr6:32473619:G:A	HLA-DRB9	Intergenic	rs7773030	G>A	10	7.37x10-16	0.61 [0.54-0.69]	0.004	63.32
6	32474614	chr6:32474614:A:C	HLA-DRB9	Intergenic	rs61550563	A>C	10	1.08x10-09	0.60 [0.51-0.71]	0.582	0
6	32475059	chr6:32475059:T:G	HLA-DRB9	Intergenic	rs13211921	T>G	10	3.07x10-46	1.52 [1.44-1.60]	0.506	0
6	32475254	chr6:32475254:C:T	HLA-DRB9	Intergenic	rs17203430	C>T	10	1.01x10-09	0.60 [0.51-0.70]	0.587	0
6	32475256	chr6:32475256:C:G	HLA-DRB9	Intergenic	rs17203444	C>G	10	1.01x10-09	0.60 [0.51-0.70]	0.587	0
6	32475284	chr6:32475284:C:A	HLA-DRB9	Intergenic	rs17209803	C>A	10	1.03x10-09	0.60 [0.51-0.70]	0.586	0
6	32475395	chr6:32475395:C:T	HLA-DRB9	Intergenic	rs9391879	C>T	10	3.19x10-46	1.52 [1.44-1.60]	0.506	0
6	32475398	chr6:32475398:G:A	HLA-DRB9	Intergenic	rs17203458	G>A	10	1.01x10-09	0.60 [0.51-0.70]	0.587	0
6	32475446	chr6:32475446:G:A	HLA-DRB9	Intergenic	rs9269108	G>A	10	2.64x10-13	0.80 [0.75-0.85]	0.795	0
6	32475489	chr6:32475489:T:C	HLA-DRB9	Intergenic	rs9269109	T>C	10	2.59x10-37	0.66 [0.62-0.70]	0.061	44.77
6	32475492	chr6:32475492:A:C	HLA-DRB9	Intergenic	rs9269110	A>C	10	3.48x10-34	0.67 [0.63-0.71]	0.07	43.18
6	32475648	chr6:32475648:A:G	HLA-DRB9	Intergenic	rs9269114	A>G	10	6.50x10-34	0.67 [0.63-0.71]	0.074	42.51
6	32475655	chr6:32475655:C:G	HLA-DRB9	Intergenic	rs9391881	C>G	10	3.07x10-46	1.52 [1.44-1.60]	0.506	0
6	32475674	chr6:32475674:G:A	HLA-DRB9	Intergenic	rs9378264	G>A	10	3.07x10-46	1.52 [1.44-1.60]	0.506	0
6	32475889	chr6:32475889:G:C	HLA-DRB9	Intergenic	rs28895242	G>C	10	3.07x10-46	1.52 [1.44-1.60]	0.506	0
6	32476244	chr6:32476244:C:T	HLA-DRB9	Intergenic	rs28895249	C>T	10	5.24x10-46	1.51 [1.43-1.60]	0.515	0
6	32476553	chr6:32476553:A:C	HLA-DRB9	Intergenic	rs12207473	A>C	10	3.07x10-46	1.52 [1.44-1.60]	0.506	0
6	32476698	chr6:32476698:T:C	HLA-DRB9	Intergenic	rs6457598	T>C	10	1.22x10-10	0.70 [0.63-0.78]	0.046	47.67
6	32476767	chr6:32476767:C:T	HLA-DRB9	Intergenic	rs12195582	C>T	10	3.15x10-46	1.52 [1.44-1.60]	0.505	0
6	32476807	chr6:32476807:C:T	HLA-DRB9	Intergenic	rs12195589	C>T	10	3.07x10-46	1.52 [1.44-1.60]	0.506	0
6	32476931	chr6:32476931:T:A	HLA-DRB9	Intergenic	rs28895251	T>A	10	3.07x10-46	1.52 [1.44-1.60]	0.506	0
6	32477119	chr6:32477119:C:T	HLA-DRB9	Intergenic	rs7776032	C>T	10	5.35x10-34	0.67 [0.63-0.71]	0.075	42.49
6	32477131	chr6:32477131:G:C	HLA-DRB9	Intergenic	rs9469130	G>C	10	1.01x10-09	0.60 [0.51-0.70]	0.587	0
6	32477133	chr6:32477133:G:A	HLA-DRB9	Intergenic	rs28895253	G>A	10	3.07x10-46	1.52 [1.44-1.60]	0.506	0
6	32477138	chr6:32477138:C:G	HLA-DRB9	Intergenic	rs9269126	C>G	10	2.67x10-34	0.67 [0.63-0.71]	0.066	43.96
6	32477156	chr6:32477156:T:C	HLA-DRB9	Intergenic	rs9269128	T>C	10	3.27x10-34	0.67 [0.63-0.71]	0.072	42.87

6	32477253	chr6:32477253:T:C	HLA-DRB9	Intergenic	rs9269135	T>C	10	2.28x10-34	0.67 [0.63-0.71]	0.07	43.16
6	32477302	chr6:32477302:C:T	HLA-DRB9	Intergenic	rs28895255	C>T	10	2.65x10-46	1.52 [1.44-1.60]	0.503	0
6	32477337	chr6:32477337:A:G	HLA-DRB9	Intergenic	rs28895257	A>G	10	3.39x10-46	1.52 [1.44-1.60]	0.512	0
6	32477340	chr6:32477340:C:T	HLA-DRB9	Intergenic	rs28895258	C>T	10	2.71x10-46	1.52 [1.44-1.60]	0.506	0
6	32477370	chr6:32477370:C:T	HLA-DRB9	Intergenic	rs9269137	C>T	10	4.89x10-34	0.67 [0.63-0.71]	0.065	44.09
6	32477378	chr6:32477378:A:C	HLA-DRB9	Intergenic	rs9269138	A>C	10	4.33x10-34	0.67 [0.63-0.71]	0.067	43.71
6	32477379	chr6:32477379:G:A	HLA-DRB9	Intergenic	rs9269139	G>A	10	4.33x10-34	0.67 [0.63-0.71]	0.067	43.71
6	32477481	chr6:32477481:A:G	HLA-DRB9	Intergenic	rs28895259	A>G	10	3.57x10-46	1.51 [1.43-1.60]	0.518	0
6	32477484	chr6:32477484:T:C	HLA-DRB9	Intergenic	rs28895260	T>C	10	3.25x10-46	1.52 [1.44-1.60]	0.505	0
6	32477493	chr6:32477493:C:T	HLA-DRB9	Intergenic	rs9269141	C>T	10	3.40x10-34	0.67 [0.63-0.71]	0.071	43.08
6	32477507	chr6:32477507:G:T	HLA-DRB9	Intergenic	rs148948227	G>T	10	1.01x10-09	0.60 [0.51-0.70]	0.587	0
6	32477529	chr6:32477529:G:A	HLA-DRB9	Intergenic	rs28895261	G>A	10	2.62x10-46	1.52 [1.44-1.60]	0.516	0
6	32477646	chr6:32477646:G:A	HLA-DRB9	Intergenic	rs9269142	G>A	10	3.48x10-34	0.67 [0.63-0.71]	0.07	43.18
6	32477823	chr6:32477823:G:A	HLA-DRB9	Intergenic	rs9405112	G>A	10	2.85x10-83	1.77 [1.68-1.87]	0.259	19.98
6	32478706	chr6:32478706:T:C	-	Intergenic	rs9269157	T>C	10	3.48x10-34	0.67 [0.63-0.71]	0.07	43.18
6	32479145	chr6:32479145:T:C	-	Intergenic	rs17203619	T>C	10	3.07x10-46	1.52 [1.44-1.60]	0.506	0
6	32479216	chr6:32479216:T:C	-	Intergenic	rs4640926	T>C	9	1.16x10-13	0.66 [0.59-0.74]	0.644	0
6	32479304	chr6:32479304:C:G	-	Intergenic	rs9469133	C>G	10	1.01x10-09	0.60 [0.51-0.70]	0.587	0
6	32479308	chr6:32479308:T:C	-	Intergenic	rs2395191	T>C	10	4.93x10-47	0.63 [0.59-0.67]	0.139	33.59
6	32479334	chr6:32479334:A:G	-	Intergenic	rs29001652	A>G	10	3.07x10-46	1.52 [1.44-1.60]	0.506	0
6	32479342	chr6:32479342:C:T	-	Intergenic	rs9469134	C>T	10	9.03x10-10	0.60 [0.51-0.70]	0.587	0
6	32479362	chr6:32479362:C:A	-	Intergenic	rs9461758	C>A	10	1.01x10-09	0.60 [0.51-0.70]	0.587	0
6	32479377	chr6:32479377:C:G	-	Intergenic	rs9269172	C>G	10	2.72x10-34	0.67 [0.63-0.71]	0.066	43.93
6	32479385	chr6:32479385:G:A	-	Intergenic	rs29001478	G>A	10	3.49x10-46	1.51 [1.44-1.60]	0.501	0
6	32479439	chr6:32479439:A:T	-	Intergenic	rs29001620	A>T	10	3.07x10-46	1.52 [1.44-1.60]	0.506	0
6	32479442	chr6:32479442:G:A	-	Intergenic	rs28732246	G>A	10	3.07x10-46	1.52 [1.44-1.60]	0.506	0
6	32479564	chr6:32479564:G:A	-	Intergenic	rs29001568	G>A	10	3.07x10-46	1.52 [1.44-1.60]	0.506	0
6	32479587	chr6:32479587:T:C	-	Intergenic	rs9269174	T>C	10	4.01x10-34	0.67 [0.63-0.71]	0.071	43.11
6	32479674	chr6:32479674:G:A	-	Intergenic	rs9269175	G>A	10	3.48x10-34	0.67 [0.63-0.71]	0.07	43.18
6	32479697	chr6:32479697:A:G	-	Intergenic	rs28732247	A>G	10	2.71x10-46	1.52 [1.44-1.60]	0.503	0
6	32479741	chr6:32479741:T:A	-	Intergenic	rs35007143	T>A	10	3.07x10-46	1.52 [1.44-1.60]	0.506	0
6	32479743	chr6:32479743:G:A	-	Intergenic	rs11754562	G>A	10	1.01x10-09	0.60 [0.51-0.70]	0.587	0
6	32479768	chr6:32479768:T:G	-	Intergenic	rs9269176	T>G	10	4.13x10-34	0.67 [0.63-0.71]	0.069	43.36
6	32481383	chr6:32481383:G:T	-	Intergenic	rs9394099	G>T	10	2.00x10-46	1.52 [1.44-1.60]	0.499	0
6	32484509	chr6:32484509:T:G	-	Intergenic	rs1557548	T>G	10	7.19x10-11	0.65 [0.58-0.74]	0.42	2.04
6	32485559	chr6:32485559:G:T	-	Intergenic	rs34036879	G>T	10	1.88x10-83	1.77 [1.68-1.87]	0.249	21.12
6	32486018	chr6:32486018:A:C	-	Intergenic	rs9269243	A>C	9	1.16x10-13	0.66 [0.59-0.74]	0.644	0
6	32486624	chr6:32486624:A:T	-	Intergenic	rs9269256	A>T	10	7.54x10-16	0.69 [0.63-0.75]	0.224	23.87
6	32486804	chr6:32486804:C:T	-	Intergenic	rs9269263	C>T	10	4.87x10-16	0.69 [0.63-0.75]	0.234	22.74
6	32533771	chr6:32533771:G:A	HLA-DRB5	Intergenic	rs71549250	G>A	10	7.13x10-16	0.69 [0.63-0.75]	0.244	21.64
6	32535551	chr6:32535551:G:A	-	Intergenic	rs116132127	G>A	10	4.44x10-17	0.78 [0.73-0.82]	0.493	0
6	32535558	chr6:32535558:G:A	-	Intergenic	rs115878723	G>A	10	4.97x10-17	0.78 [0.73-0.82]	0.493	0
6	32535575	chr6:32535575:A:T	-	Intergenic	rs114961271	A>T	10	4.38x10-17	0.78 [0.73-0.82]	0.494	0
6	32535994	chr6:32535994:G:C	-	Intergenic	rs35874713	G>C	10	7.79x10-16	0.69 [0.63-0.75]	0.252	20.8
6	32536048	chr6:32536048:T:C	-	Intergenic	rs75802100	T>C	10	1.22x10-13	0.72 [0.67-0.79]	0.107	37.71
6	32536103	chr6:32536103:G:A	-	Intergenic	rs76662624	G>A	10	3.10x10-17	0.78 [0.73-0.82]	0.535	0

6	32536238	chr6:32536238:A:G	-	Intergenic	rs67894469	A>G	10	7.83x10-16	0.69 [0.63-0.75]	0.246	21.46
6	32539975	chr6:32539975:T:G	-	Intergenic	rs67382147	T>G	10	7.22x10-16	0.69 [0.63-0.75]	0.243	21.77
6	32540782	chr6:32540782:G:T	-	Intergenic	rs62405590	G>T	10	6.76x10-16	0.69 [0.63-0.75]	0.249	21.13
6	32541411	chr6:32541411:A:T	-	Intergenic	rs67111333	A>T	10	7.59x10-16	0.69 [0.63-0.75]	0.241	21.97
6	32541517	chr6:32541517:G:A	-	Intergenic	rs79207037	G>A	10	1.82x10-78	1.75 [1.66-1.85]	0.246	21.4
6	32541543	chr6:32541543:C:T	-	Intergenic	rs66678933	C>T	10	4.20x10-17	0.78 [0.73-0.82]	0.483	0
6	32541634	chr6:32541634:C:T	-	Intergenic	rs66502326	C>T	10	5.91x10-17	0.78 [0.74-0.82]	0.434	0.43
6	32541640	chr6:32541640:A:T	-	Intergenic	rs28776176	A>T	10	1.04x10-15	0.69 [0.63-0.75]	0.247	21.3
6	32542120	chr6:32542120:G:T	-	Intergenic	rs78942674	G>T	9	1.16x10-14	0.79 [0.75-0.84]	0.207	26.68
6	32542158	chr6:32542158:C:T	-	Intergenic	rs59350153	C>T	10	1.63x10-81	1.77 [1.68-1.88]	0.259	20.05
6	32542235	chr6:32542235:G:A	-	Intergenic	rs57588792	G>A	10	5.22x10-81	1.77 [1.67-1.87]	0.252	20.77
6	32542822	chr6:32542822:C:T	-	Intergenic	rs114015220	C>T	10	4.47x10-17	0.78 [0.73-0.82]	0.506	0
6	32543825	chr6:32543825:A:G	-	Intergenic	rs62405593	A>G	10	9.27x10-16	0.69 [0.63-0.75]	0.258	20.11
6	32544210	chr6:32544210:C:T	-	Intergenic	rs72492319	C>T	10	4.18x10-17	0.78 [0.73-0.82]	0.495	0
6	32544230	chr6:32544230:T:C	-	Intergenic	rs79202843	T>C	10	8.96x10-16	0.69 [0.63-0.75]	0.257	20.21
6	32545937	chr6:32545937:A:C	RNU1-61P	Intergenic	rs34791134	A>C	8	6.50x10-14	0.65 [0.59-0.73]	0.954	0
6	32546189	chr6:32546189:A:T	RNU1-61P	Intergenic	rs34072909	A>T	10	5.45x10-16	0.69 [0.63-0.75]	0.264	19.51
6	32546723	chr6:32546723:T:C	RNU1-61P	Intergenic	rs62405631	T>C	10	8.87x10-82	1.77 [1.68-1.87]	0.21	25.32
6	32546762	chr6:32546762:C:T	RNU1-61P	Intergenic	rs72844118	C>T	10	4.08x10-17	0.78 [0.73-0.82]	0.506	0
6	32546763	chr6:32546763:T:A	RNU1-61P	Intergenic	rs72844120	T>A	10	5.96x10-09	0.84 [0.79-0.89]	0.197	26.79
6	32548590	chr6:32548590:G:A	HLA-DRB6	Intergenic	rs71534589	G>A	10	9.09x10-16	0.69 [0.63-0.75]	0.214	24.9
6	32548928	chr6:32548928:C:T	HLA-DRB6	Intergenic	rs201639066	C>T	10	1.98x10-81	1.77 [1.68-1.87]	0.173	29.6
6	32549094	chr6:32549094:G:T	HLA-DRB6	Intergenic	rs12664542	G>T	10	8.04x10-10	0.60 [0.51-0.70]	0.519	0
6	32549621	chr6:32549621:G:A	HLA-DRB6	Intergenic	rs28880026	G>A	8	4.60x10-14	0.65 [0.59-0.73]	0.946	0
6	32551542	chr6:32551542:T:A	HLA-DRB6	Intergenic	rs66532036	T>A	10	7.39x10-17	0.78 [0.74-0.82]	0.476	0
6	32552051	chr6:32552051:T:G	HLA-DRB6	Intergenic	rs71536517	T>G	10	7.86x10-16	0.69 [0.63-0.75]	0.239	22.15
6	32572202	chr6:32572202:G:A	-	Intergenic	rs9269325	G>A	10	6.96x10-16	0.69 [0.63-0.75]	0.246	21.47
6	32574091	chr6:32574091:C:A	HLA-DRB1	Intergenic	rs9269437	C>A	10	3.02x10-15	0.69 [0.64-0.76]	0.173	29.54
6	32574104	chr6:32574104:C:T	HLA-DRB1	Intergenic	rs9269438	C>T	10	2.78x10-15	0.69 [0.64-0.76]	0.196	26.97
6	32574197	chr6:32574197:C:T	HLA-DRB1	Intergenic	rs9269444	C>T	10	1.30x10-15	0.69 [0.63-0.75]	0.171	29.78
6	32574234	chr6:32574234:C:T	HLA-DRB1	Intergenic	rs9269448	C>T	10	1.30x10-15	0.69 [0.63-0.75]	0.171	29.78
6	32574513	chr6:32574513:A:G	HLA-DRB1	Intergenic	rs9269467	A>G	10	1.48x10-15	0.69 [0.63-0.75]	0.169	30.05
6	32575521	chr6:32575521:C:A	HLA-DRB1	Intergenic	rs9269511	C>A	10	9.65x10-16	0.69 [0.63-0.75]	0.15	32.3
6	32577784	chr6:32577784:T:C	HLA-DRB1	Intergenic	rs9269626	T>C	10	1.35x10-15	0.69 [0.63-0.75]	0.175	29.36
6	32577827	chr6:32577827:C:T	HLA-DRB1	Intergenic	rs9269627	C>T	10	1.30x10-15	0.69 [0.63-0.75]	0.171	29.78
6	32578085	chr6:32578085:C:T	HLA-DRB1	Intergenic	rs9269640	C>T	10	1.30x10-15	0.69 [0.63-0.75]	0.171	29.78
6	32578656	chr6:32578656:C:T	HLA-DRB1	Intergenic	rs9269672	C>T	10	1.09x10-15	0.69 [0.63-0.75]	0.199	26.63
6	32591226	chr6:32591226:A:G	HLA-DRB1	Intergenic	rs9270474	A>G	10	3.09x10-21	0.76 [0.71-0.80]	0.489	0
6	32591242	chr6:32591242:G:A	HLA-DRB1	Intergenic	rs77122291	G>A	10	3.29x10-84	1.79 [1.69-1.89]	0.218	24.49
6	32591268	chr6:32591268:T:C	HLA-DRB1	Intergenic	rs9270481	T>C	10	3.09x10-21	0.76 [0.71-0.80]	0.489	0
6	32591287	chr6:32591287:T:C	HLA-DRB1	Intergenic	rs9270485	T>C	10	3.09x10-21	0.76 [0.71-0.80]	0.489	0
6	32591354	chr6:32591354:G:A	HLA-DRB1	Intergenic	rs34127991	G>A	10	3.29x10-84	1.79 [1.69-1.89]	0.218	24.49
6	32591398	chr6:32591398:C:T	HLA-DRB1	Intergenic	rs9270502	C>T	10	1.26x10-15	0.69 [0.63-0.75]	0.173	29.62
6	32591400	chr6:32591400:G:C	HLA-DRB1	Intergenic	rs561760011	G>C	10	6.33x10-87	2.03 [1.90-2.17]	0.901	0
6	32591401	chr6:32591401:G:A	HLA-DRB1	Intergenic	rs529034563	G>A	10	6.33x10-87	2.03 [1.90-2.17]	0.901	0
6	32591404	chr6:32591404:C:G	HLA-DRB1	Intergenic	rs9270503	C>G	10	3.25x10-21	0.76 [0.71-0.80]	0.488	0

6	32591407	chr6:32591407:C:A	HLA-DRB1	Intergenic	rs9270504	C>A	10	2.88x10-21	0.76 [0.71-0.80]	0.48	0
6	32591414	chr6:32591414:A:G	HLA-DRB1	Intergenic	rs114335056	A>G	10	8.61x10-87	2.03 [1.90-2.16]	0.928	0
6	32591435	chr6:32591435:A:G	HLA-DRB1	Intergenic	rs75928817	A>G	10	2.48x10-84	1.79 [1.70-1.89]	0.225	23.72
6	32591436	chr6:32591436:A:T	HLA-DRB1	Intergenic	rs115879259	A>T	10	5.80x10-87	2.03 [1.90-2.17]	0.93	0
6	32591507	chr6:32591507:A:G	HLA-DRB1	Intergenic	rs114258010	A>G	9	5.21x10-24	0.51 [0.45-0.58]	1	0
6	32591881	chr6:32591881:C:T	HLA-DRB1	Intergenic	rs9270529	C>T	10	3.71x10-21	0.76 [0.71-0.80]	0.486	0
6	32591896	chr6:32591896:T:G	HLA-DRB1	Intergenic	rs34434863	T>G	10	3.50x10-37	1.49 [1.41-1.58]	0.237	22.44
6	32591909	chr6:32591909:G:A	HLA-DRB1	Intergenic	rs9270530	G>A	10	3.25x10-21	0.76 [0.71-0.80]	0.488	0
6	32591932	chr6:32591932:C:T	HLA-DRB1	Intergenic	rs9270533	C>T	10	3.25x10-21	0.76 [0.71-0.80]	0.488	0
6	32591937	chr6:32591937:G:A	HLA-DRB1	Intergenic	rs9270535	G>A	10	3.25x10-21	0.76 [0.71-0.80]	0.488	0
6	32592127	chr6:32592127:C:T	HLA-DRB1	Intergenic	rs35743245	C>T	10	6.31x10-87	2.03 [1.90-2.17]	0.931	0
6	32592152	chr6:32592152:A:G	HLA-DRB1	Intergenic	rs11756331	A>G	9	5.21x10-24	0.51 [0.45-0.58]	1	0
6	32592157	chr6:32592157:A:G	HLA-DRB1	Intergenic	rs28366270	A>G	10	2.97x10-84	1.79 [1.70-1.89]	0.216	24.67
6	32592174	chr6:32592174:A:G	HLA-DRB1	Intergenic	rs9270547	A>G	10	3.77x10-21	0.76 [0.71-0.80]	0.488	0
6	32592593	chr6:32592593:G:T	HLA-DRB1	Intergenic	rs35472547	G>T	10	6.01x10-87	2.03 [1.90-2.17]	0.93	0
6	32592608	chr6:32592608:A:T	HLA-DRB1	Intergenic	rs34291045	A>T	10	6.01x10-87	2.03 [1.90-2.17]	0.93	0
6	32592655	chr6:32592655:C:T	HLA-DRB1	Intergenic	rs28366288	C>T	10	3.29x10-84	1.79 [1.69-1.89]	0.218	24.49
6	32592663	chr6:32592663:G:A	HLA-DRB1	Intergenic	rs9270549	G>A	10	3.25x10-21	0.76 [0.71-0.80]	0.488	0
6	32592664	chr6:32592664:A:G	HLA-DRB1	Intergenic	rs9270550	A>G	10	3.84x10-21	0.76 [0.71-0.80]	0.5	0
6	32592669	chr6:32592669:C:T	HLA-DRB1	Intergenic	rs34924558	C>T	10	6.01x10-87	2.03 [1.90-2.17]	0.93	0
6	32592678	chr6:32592678:C:T	HLA-DRB1	Intergenic	rs34496598	C>T	10	6.01x10-87	2.03 [1.90-2.17]	0.93	0
6	32592700	chr6:32592700:A:G	HLA-DRB1	Intergenic	rs34415150	A>G	10	7.53x10-87	2.03 [1.90-2.16]	0.944	0
6	32592701	chr6:32592701:C:T	HLA-DRB1	Intergenic	rs11752229	C>T	9	6.23x10-24	0.51 [0.45-0.58]	1	0
6	32592706	chr6:32592706:T:C	HLA-DRB1	Intergenic	rs34212923	T>C	10	6.01x10-87	2.03 [1.90-2.17]	0.93	0
6	32592757	chr6:32592757:A:T	HLA-DRB1	Intergenic	rs11756836	A>T	9	5.21x10-24	0.51 [0.45-0.58]	1	0
6	32592829	chr6:32592829:G:A	HLA-DRB1	Intergenic	rs9270556	G>A	10	3.19x10-21	0.76 [0.71-0.80]	0.488	0
6	32592854	chr6:32592854:C:T	HLA-DRB1	Intergenic	rs35118762	C>T	10	5.72x10-87	2.03 [1.90-2.17]	0.93	0
6	32592890	chr6:32592890:G:A	HLA-DRB1	Intergenic	rs9270558	G>A	10	3.06x10-21	0.76 [0.71-0.80]	0.489	0
6	32592918	chr6:32592918:G:C	HLA-DRB1	Intergenic	rs34928543	G>C	10	9.47x10-87	2.03 [1.90-2.16]	0.932	0
6	32592921	chr6:32592921:T:G	HLA-DRB1	Intergenic	rs9270559	T>G	10	2.72x10-24	0.74 [0.70-0.78]	0.555	0
6	32592938	chr6:32592938:G:A	HLA-DRB1	Intergenic	rs28366294	G>A	10	5.27x10-84	1.79 [1.69-1.89]	0.224	23.83
6	32592950	chr6:32592950:C:A	HLA-DRB1	Intergenic	rs28366296	C>A	10	5.21x10-84	1.79 [1.69-1.89]	0.222	24
6	32592964	chr6:32592964:T:C	HLA-DRB1	Intergenic	rs9270560	T>C	10	4.98x10-09	1.19 [1.12-1.26]	0.169	30.05
6	32592965	chr6:32592965:G:A	HLA-DRB1	Intergenic	rs34752364	G>A	10	1.08x10-86	2.03 [1.90-2.16]	0.933	0
6	32593024	chr6:32593024:T:G	HLA-DRB1	Intergenic	rs9270567	T>G	10	3.16x10-21	0.76 [0.71-0.80]	0.488	0
6	32593052	chr6:32593052:G:C	HLA-DRB1	Intergenic	rs28366297	G>C	10	5.40x10-84	1.79 [1.69-1.89]	0.225	23.74
6	32593082	chr6:32593082:A:C	HLA-DRB1	Intergenic	rs28366298	A>C	10	3.05x10-84	1.79 [1.69-1.89]	0.218	24.48
6	32593093	chr6:32593093:T:A	HLA-DRB1	Intergenic	rs28366299	T>A	10	6.64x10-84	1.79 [1.69-1.89]	0.228	23.38
6	32593098	chr6:32593098:C:T	HLA-DRB1	Intergenic	rs28366300	C>T	10	6.64x10-84	1.79 [1.69-1.89]	0.228	23.38
6	32593106	chr6:32593106:G:A	HLA-DRB1	Intergenic	rs28366301	G>A	10	6.64x10-84	1.79 [1.69-1.89]	0.228	23.38
6	32593157	chr6:32593157:G:C	HLA-DRB1	Intergenic	rs28366302	G>C	10	3.29x10-84	1.79 [1.69-1.89]	0.218	24.49
6	32593186	chr6:32593186:C:G	HLA-DRB1	Intergenic	rs28366303	C>G	10	3.29x10-84	1.79 [1.69-1.89]	0.218	24.49
6	32593201	chr6:32593201:A:G	HLA-DRB1	Intergenic	rs9270570	A>G	10	2.92x10-21	0.76 [0.71-0.80]	0.489	0
6	32593204	chr6:32593204:A:G	HLA-DRB1	Intergenic	rs28366304	A>G	10	3.72x10-84	1.79 [1.69-1.89]	0.216	24.71
6	32593205	chr6:32593205:C:T	HLA-DRB1	Intergenic	rs28366305	C>T	10	3.72x10-84	1.79 [1.69-1.89]	0.216	24.71
6	32593233	chr6:32593233:G:T	HLA-DRB1	Intergenic	rs9270572	G>T	10	2.92x10-21	0.76 [0.71-0.80]	0.489	0

6	32593271	chr6:32593271:G:T	HLA-DRB1	Intergenic	rs28366309	G>T	10	3.29x10-84	1.79 [1.69-1.89]	0.218	24.49
6	32593351	chr6:32593351:A:C	HLA-DRB1	Intergenic	rs28366310	A>C	10	3.29x10-84	1.79 [1.69-1.89]	0.218	24.49
6	32593360	chr6:32593360:C:G	HLA-DRB1	Intergenic	rs28366311	C>G	10	3.29x10-84	1.79 [1.69-1.89]	0.218	24.49
6	32593398	chr6:32593398:T:C	HLA-DRB1	Intergenic	rs28366312	T>C	10	3.29x10-84	1.79 [1.69-1.89]	0.218	24.49
6	32593409	chr6:32593409:G:A	HLA-DRB1	Intergenic	rs28366313	G>A	10	3.29x10-84	1.79 [1.69-1.89]	0.218	24.49
6	32593424	chr6:32593424:A:G	HLA-DRB1	Intergenic	rs28366314	A>G	10	3.29x10-84	1.79 [1.69-1.89]	0.218	24.49
6	32593430	chr6:32593430:C:T	HLA-DRB1	Intergenic	rs28366315	C>T	10	3.29x10-84	1.79 [1.69-1.89]	0.218	24.49
6	32593452	chr6:32593452:A:G	HLA-DRB1	Intergenic	rs9270583	A>G	10	3.22x10-21	0.76 [0.71-0.80]	0.488	0
6	32593469	chr6:32593469:T:C	HLA-DRB1	Intergenic	rs28366316	T>C	10	3.29x10-84	1.79 [1.69-1.89]	0.218	24.49
6	32593473	chr6:32593473:A:G	HLA-DRB1	Intergenic	rs7749350	A>G	9	5.21x10-24	0.51 [0.45-0.58]	1	0
6	32593506	chr6:32593506:C:A	HLA-DRB1	Intergenic	rs35525122	C>A	10	6.45x10-87	2.03 [1.90-2.17]	0.931	0
6	32593523	chr6:32593523:C:T	HLA-DRB1	Intergenic	rs9270585	C>T	10	1.46x10-09	1.19 [1.13-1.26]	0.092	39.88
6	32593550	chr6:32593550:C:T	HLA-DRB1	Intergenic	rs9270588	C>T	10	1.45x10-09	1.19 [1.13-1.26]	0.092	39.9
6	32593557	chr6:32593557:C:G	HLA-DRB1	Intergenic	rs35265698	C>G	10	6.45x10-87	2.03 [1.90-2.17]	0.931	0
6	32593593	chr6:32593593:C:T	HLA-DRB1	Intergenic	rs35139284	C>T	10	3.70x10-32	1.44 [1.36-1.53]	0.083	41.18
6	32593613	chr6:32593613:C:T	HLA-DRB1	Intergenic	rs9270589	C>T	10	3.11x10-21	0.76 [0.71-0.80]	0.489	0
6	32593615	chr6:32593615:G:A	HLA-DRB1	Intergenic	rs9270590	G>A	10	2.44x10-21	0.75 [0.71-0.80]	0.493	0
6	32593627	chr6:32593627:A:C	HLA-DRB1	Intergenic	rs7753335	A>C	10	3.04x10-10	0.59 [0.50-0.69]	0.615	0
6	32593634	chr6:32593634:C:T	HLA-DRB1	Intergenic	rs28366317	C>T	10	3.29x10-84	1.79 [1.69-1.89]	0.218	24.49
6	32593647	chr6:32593647:C:T	HLA-DRB1	Intergenic	rs9270591	C>T	10	1.46x10-09	1.19 [1.13-1.26]	0.092	39.88
6	32593652	chr6:32593652:A:T	HLA-DRB1	Intergenic	rs9270592	A>T	10	3.25x10-21	0.76 [0.71-0.80]	0.488	0
6	32593668	chr6:32593668:A:G	HLA-DRB1	Intergenic	rs9270593	A>G	10	3.25x10-21	0.76 [0.71-0.80]	0.488	0
6	32593688	chr6:32593688:C:T	HLA-DRB1	Intergenic	rs34350244	C>T	10	6.45x10-87	2.03 [1.90-2.17]	0.931	0
6	32593689	chr6:32593689:A:G	HLA-DRB1	Intergenic	rs35294087	A>G	10	6.45x10-87	2.03 [1.90-2.17]	0.931	0
6	32593718	chr6:32593718:G:A	HLA-DRB1	Intergenic	rs28366319	G>A	10	2.97x10-84	1.79 [1.70-1.89]	0.216	24.67
6	32593750	chr6:32593750:T:C	HLA-DRB1	Intergenic	rs34535888	T>C	10	4.60x10-87	2.03 [1.90-2.17]	0.931	0
6	32593774	chr6:32593774:G:A	HLA-DRB1	Intergenic	rs28366320	G>A	10	2.14x10-45	1.51 [1.43-1.59]	0.453	0
6	32593788	chr6:32593788:T:C	HLA-DRB1	Intergenic	rs34553045	T>C	10	9.92x10-87	2.03 [1.90-2.16]	0.9	0
6	32593805	chr6:32593805:T:A	HLA-DRB1	Intergenic	rs9270596	T>A	8	2.49x10-14	0.65 [0.58-0.72]	0.93	0
6	32593821	chr6:32593821:T:G	HLA-DRB1	Intergenic	rs9270597	T>G	10	3.07x10-21	0.76 [0.71-0.80]	0.494	0
6	32593832	chr6:32593832:A:G	HLA-DRB1	Intergenic	rs28366323	A>G	10	8.29x10-84	1.79 [1.69-1.89]	0.223	23.89
6	32593841	chr6:32593841:T:C	HLA-DRB1	Intergenic	rs9270598	T>C	10	3.25x10-21	0.76 [0.71-0.80]	0.488	0
6	32593861	chr6:32593861:C:T	HLA-DRB1	Intergenic	rs35371668	C>T	10	5.44x10-87	2.03 [1.90-2.17]	0.934	0
6	32593882	chr6:32593882:T:C	HLA-DRB1	Intergenic	rs28366325	T>C	10	2.94x10-84	1.79 [1.70-1.89]	0.219	24.36
6	32593901	chr6:32593901:G:A	HLA-DRB1	Intergenic	rs28366327	G>A	10	2.94x10-84	1.79 [1.70-1.89]	0.219	24.36
6	32593904	chr6:32593904:G:A	HLA-DRB1	Intergenic	rs34647096	G>A	10	5.44x10-87	2.03 [1.90-2.17]	0.934	0
6	32593989	chr6:32593989:A:G	HLA-DRB1	Intergenic	rs28366328	A>G	10	3.07x10-84	1.79 [1.70-1.89]	0.221	24.21
6	32594017	chr6:32594017:C:G	HLA-DRB1	Intergenic	rs9270602	C>G	10	1.26x10-15	0.69 [0.63-0.75]	0.173	29.62
6	32594042	chr6:32594042:G:A	HLA-DRB1	Intergenic	rs28366329	G>A	10	3.07x10-84	1.79 [1.70-1.89]	0.221	24.21
6	32594069	chr6:32594069:G:C	HLA-DRB1	Intergenic	rs28366330	G>C	10	3.07x10-84	1.79 [1.70-1.89]	0.221	24.21
6	32594103	chr6:32594103:T:G	HLA-DRB1	Intergenic	rs28366331	T>G	10	3.07x10-84	1.79 [1.70-1.89]	0.221	24.21
6	32594108	chr6:32594108:C:A	HLA-DRB1	Intergenic	rs28366332	C>A	10	3.07x10-84	1.79 [1.70-1.89]	0.221	24.21
6	32594110	chr6:32594110:G:C	HLA-DRB1	Intergenic	rs28366333	G>C	10	3.07x10-84	1.79 [1.70-1.89]	0.221	24.21
6	32594118	chr6:32594118:A:C	HLA-DRB1	Intergenic	rs28366334	A>C	10	3.07x10-84	1.79 [1.70-1.89]	0.221	24.21
6	32594134	chr6:32594134:A:G	HLA-DRB1	Intergenic	rs28366335	A>G	10	2.99x10-84	1.79 [1.70-1.89]	0.22	24.29
6	32596904	chr6:32596904:A:C	-	Intergenic	rs17191234	A>C	10	4.41x10-24	1.66 [1.51-1.82]	0.034	50.35

6	32596922	chr6:32596922:A:G	-	Intergenic	rs28366337	A>G	10	4.41x10-24	1.66 [1.51-1.82]	0.034	50.35
6	32596933	chr6:32596933:T:C	-	Intergenic	rs28366338	T>C	10	4.41x10-24	1.66 [1.51-1.82]	0.034	50.35
6	32596948	chr6:32596948:A:G	-	Intergenic	rs28366339	A>G	10	4.41x10-24	1.66 [1.51-1.82]	0.034	50.35
6	32596950	chr6:32596950:A:G	-	Intergenic	rs28366340	A>G	10	4.41x10-24	1.66 [1.51-1.82]	0.034	50.35
6	32597007	chr6:32597007:G:C	-	Intergenic	rs28366341	G>C	10	4.41x10-24	1.66 [1.51-1.82]	0.034	50.35
6	32597023	chr6:32597023:T:C	-	Intergenic	rs9270604	T>C	10	6.88x10-17	0.77 [0.73-0.82]	0.78	0
6	32597026	chr6:32597026:T:C	-	Intergenic	rs28366343	T>C	10	5.76x10-24	1.66 [1.51-1.82]	0.033	50.58
6	32597036	chr6:32597036:T:C	-	Intergenic	rs28366344	T>C	10	5.76x10-24	1.66 [1.51-1.82]	0.033	50.58
6	32597092	chr6:32597092:C:T	-	Intergenic	rs9270606	C>T	10	6.88x10-17	0.77 [0.73-0.82]	0.78	0
6	32597208	chr6:32597208:C:T	-	Intergenic	rs35372932	C>T	9	5.21x10-24	0.51 [0.45-0.58]	1	0
6	32597213	chr6:32597213:G:A	-	Intergenic	rs28366351	G>A	10	9.09x10-24	1.65 [1.51-1.82]	0.032	50.83
6	32597216	chr6:32597216:T:C	-	Intergenic	rs28366352	T>C	10	9.09x10-24	1.65 [1.51-1.82]	0.032	50.83
6	32597227	chr6:32597227:A:G	-	Intergenic	rs28366353	A>G	10	5.76x10-24	1.66 [1.51-1.82]	0.033	50.58
6	32597267	chr6:32597267:T:G	-	Intergenic	rs28366354	T>G	10	5.98x10-24	1.66 [1.51-1.82]	0.033	50.6
6	32597279	chr6:32597279:T:G	-	Intergenic	rs28366355	T>G	10	5.76x10-24	1.66 [1.51-1.82]	0.033	50.58
6	32597314	chr6:32597314:G:T	-	Intergenic	rs28366356	G>T	10	5.76x10-24	1.66 [1.51-1.82]	0.033	50.58
6	32597377	chr6:32597377:C:G	-	Intergenic	rs9270616	C>G	10	6.88x10-17	0.77 [0.73-0.82]	0.78	0
6	32597387	chr6:32597387:A:G	-	Intergenic	rs28366358	A>G	10	6.48x10-24	1.66 [1.51-1.82]	0.032	50.71
6	32597392	chr6:32597392:C:T	-	Intergenic	rs28366359	C>T	10	5.90x10-24	1.66 [1.51-1.82]	0.033	50.63
6	32597399	chr6:32597399:G:C	-	Intergenic	rs28366360	G>C	10	5.76x10-24	1.66 [1.51-1.82]	0.033	50.58
6	32597420	chr6:32597420:G:A	-	Intergenic	rs28366361	G>A	10	5.76x10-24	1.66 [1.51-1.82]	0.033	50.58
6	32597424	chr6:32597424:G:A	-	Intergenic	rs2760980	G>A	10	6.45x10-87	2.03 [1.90-2.17]	0.931	0
6	32597436	chr6:32597436:T:C	-	Intergenic	rs28366362	T>C	10	5.76x10-24	1.66 [1.51-1.82]	0.033	50.58
6	32597459	chr6:32597459:C:T	-	Intergenic	rs9270618	C>T	10	6.88x10-17	0.77 [0.73-0.82]	0.78	0
6	32597506	chr6:32597506:G:A	-	Intergenic	rs9270621	G>A	10	6.88x10-17	0.77 [0.73-0.82]	0.78	0
6	32597688	chr6:32597688:G:A	-	Intergenic	rs2760981	G>A	10	1.42x10-09	1.19 [1.13-1.26]	0.092	39.92
6	32597958	chr6:32597958:C:T	-	Intergenic	rs28383171	C>T	10	1.58x10-23	1.65 [1.50-1.82]	0.03	51.35
6	32598028	chr6:32598028:A:C	-	Intergenic	rs9270647	A>C	10	6.88x10-17	0.77 [0.73-0.82]	0.78	0
6	32598107	chr6:32598107:C:T	-	Intergenic	rs9270651	C>T	10	6.88x10-17	0.77 [0.73-0.82]	0.779	0
6	32598128	chr6:32598128:C:T	-	Intergenic	rs9270652	C>T	10	6.65x10-17	0.77 [0.73-0.82]	0.779	0
6	32598163	chr6:32598163:G:A	-	Intergenic	rs9270653	G>A	10	6.65x10-17	0.77 [0.73-0.82]	0.779	0
6	32598169	chr6:32598169:T:G	-	Intergenic	rs9270654	T>G	10	6.65x10-17	0.77 [0.73-0.82]	0.779	0
6	32598189	chr6:32598189:A:T	-	Intergenic	rs9270655	A>T	10	6.65x10-17	0.77 [0.73-0.82]	0.779	0
6	32598234	chr6:32598234:C:A	-	Intergenic	rs9270656	C>A	10	1.26x10-15	0.69 [0.63-0.75]	0.173	29.62
6	32598244	chr6:32598244:T:G	-	Intergenic	rs9270657	T>G	10	5.48x10-16	0.78 [0.74-0.83]	0.899	0
6	32598295	chr6:32598295:T:C	-	Intergenic	rs9270658	T>C	10	6.65x10-17	0.77 [0.73-0.82]	0.779	0
6	32598299	chr6:32598299:C:T	-	Intergenic	rs9270659	C>T	10	6.65x10-17	0.77 [0.73-0.82]	0.779	0
6	32598300	chr6:32598300:A:G	-	Intergenic	rs9270660	A>G	10	6.65x10-17	0.77 [0.73-0.82]	0.779	0
6	32598310	chr6:32598310:A:G	-	Intergenic	rs9270661	A>G	10	6.65x10-17	0.77 [0.73-0.82]	0.779	0
6	32598324	chr6:32598324:A:G	-	Intergenic	rs9270663	A>G	10	6.65x10-17	0.77 [0.73-0.82]	0.779	0
6	32598372	chr6:32598372:G:A	-	Intergenic	rs9270664	G>A	10	4.82x10-15	0.56 [0.49-0.65]	0	71.67
6	32598379	chr6:32598379:G:A	-	Intergenic	rs11754183	G>A	9	5.21x10-24	0.51 [0.45-0.58]	1	0
6	32598380	chr6:32598380:T:A	-	Intergenic	rs11756633	T>A	10	3.04x10-10	0.59 [0.50-0.69]	0.615	0
6	32598510	chr6:32598510:G:C	-	Intergenic	rs28383173	G>C	10	5.76x10-24	1.66 [1.51-1.82]	0.033	50.58
6	32598511	chr6:32598511:G:A	-	Intergenic	rs28383174	G>A	10	5.76x10-24	1.66 [1.51-1.82]	0.033	50.58
6	32598529	chr6:32598529:T:C	-	Intergenic	rs9270667	T>C	10	6.65x10-17	0.77 [0.73-0.82]	0.779	0

6	32598621	chr6:32598621:G:A	-	Intergenic	rs2760985	G>A	10	2.09x10-85	2.02 [1.89-2.16]	0.922	0
6	32598724	chr6:32598724:C:T	-	Intergenic	rs74729707	C>T	10	9.81x10-27	0.55 [0.50-0.61]	0.581	0
6	32598800	chr6:32598800:C:T	-	Intergenic	rs4329147	C>T	10	1.26x10-15	0.69 [0.63-0.75]	0.173	29.62
6	32598858	chr6:32598858:G:C	-	Intergenic	rs2454139	G>C	10	6.45x10-87	2.03 [1.90-2.17]	0.931	0
6	32598872	chr6:32598872:T:C	-	Intergenic	rs2858875	T>C	10	6.65x10-17	0.77 [0.73-0.82]	0.779	0
6	32598935	chr6:32598935:T:C	-	Intergenic	rs2858874	T>C	10	5.76x10-24	1.66 [1.51-1.82]	0.033	50.58
6	32598992	chr6:32598992:G:C	-	Intergenic	rs2858873	G>C	10	7.56x10-17	0.77 [0.73-0.82]	0.783	0
6	32599032	chr6:32599032:T:G	-	Intergenic	rs35820711	T>G	10	3.04x10-10	0.59 [0.50-0.69]	0.615	0
6	32599115	chr6:32599115:A:G	-	Intergenic	rs71542417	A>G	10	9.58x10-27	0.55 [0.50-0.61]	0.579	0
6	32599714	chr6:32599714:G:T	-	Intergenic	rs9270715	G>T	10	1.64x10-15	0.69 [0.63-0.76]	0.185	28.16
6	32599729	chr6:32599729:C:G	-	Intergenic	rs9270717	C>G	10	1.30x10-15	0.69 [0.63-0.75]	0.171	29.78
6	32599826	chr6:32599826:A:T	-	Intergenic	rs9689836	A>T	10	9.58x10-27	0.55 [0.50-0.61]	0.579	0
6	32599873	chr6:32599873:T:C	-	Intergenic	rs9689437	T>C	10	9.58x10-27	0.55 [0.50-0.61]	0.579	0
6	32599980	chr6:32599980:G:A	-	Intergenic	rs591973	G>A	10	5.80x10-17	0.77 [0.73-0.82]	0.509	0
6	32600120	chr6:32600120:G:A	-	Intergenic	rs28752490	G>A	10	1.36x10-68	1.67 [1.58-1.76]	0.073	42.81
6	32600179	chr6:32600179:T:C	-	Intergenic	rs9270728	T>C	10	5.80x10-17	0.77 [0.73-0.82]	0.509	0
6	32600895	chr6:32600895:G:A	-	Intergenic	rs11752186	G>A	9	5.17x10-24	0.51 [0.45-0.58]	1	0
6	32601130	chr6:32601130:T:A	-	Intergenic	rs9270769	T>A	10	5.61x10-17	0.77 [0.73-0.82]	0.509	0
6	32601133	chr6:32601133:T:A	-	Intergenic	rs9270770	T>A	10	5.61x10-17	0.77 [0.73-0.82]	0.509	0
6	32601160	chr6:32601160:T:C	-	Intergenic	rs10947315	T>C	10	9.58x10-27	0.55 [0.50-0.61]	0.579	0
6	32601187	chr6:32601187:G:T	-	Intergenic	rs9270771	G>T	10	4.57x10-17	0.77 [0.73-0.82]	0.536	0
6	32601190	chr6:32601190:T:C	-	Intergenic	rs9270772	T>C	10	1.70x10-14	0.79 [0.74-0.84]	0.517	0
6	32601213	chr6:32601213:A:G	-	Intergenic	rs9270773	A>G	10	6.18x10-17	0.77 [0.73-0.82]	0.511	0
6	32601218	chr6:32601218:G:A	-	Intergenic	rs28752501	G>A	10	1.36x10-68	1.67 [1.58-1.76]	0.073	42.81
6	32601638	chr6:32601638:G:A	-	Intergenic	rs9270803	G>A	10	6.93x10-17	0.77 [0.73-0.82]	0.457	0
6	32601723	chr6:32601723:C:T	-	Intergenic	rs11752754	C>T	9	5.21x10-24	0.51 [0.45-0.58]	1	0
6	32601786	chr6:32601786:C:T	-	Intergenic	rs9270806	C>T	10	6.46x10-22	0.75 [0.71-0.79]	0.184	28.3
6	32601795	chr6:32601795:G:A	-	Intergenic	rs9270807	G>A	10	6.46x10-22	0.75 [0.71-0.79]	0.184	28.3
6	32601860	chr6:32601860:A:C	-	Intergenic	rs11757382	A>C	10	7.96x10-28	0.62 [0.57-0.67]	0.591	0
6	32601914	chr6:32601914:G:A	-	Intergenic	rs477515	G>A	10	3.38x10-85	1.80 [1.70-1.90]	0.278	17.95
6	32601934	chr6:32601934:T:C	-	Intergenic	rs6938928	T>C	10	7.97x10-28	0.62 [0.57-0.67]	0.591	0
6	32601935	chr6:32601935:T:A	-	Intergenic	rs6938929	T>A	10	7.68x10-28	0.62 [0.57-0.67]	0.593	0
6	32601945	chr6:32601945:G:T	-	Intergenic	rs476775	G>T	10	6.46x10-22	0.75 [0.71-0.79]	0.184	28.3
6	32602015	chr6:32602015:T:C	-	Intergenic	rs476602	T>C	10	6.46x10-22	0.75 [0.71-0.79]	0.184	28.3
6	32602075	chr6:32602075:G:A	-	Intergenic	rs622137	G>A	10	3.55x10-85	1.80 [1.70-1.90]	0.279	17.83
6	32602082	chr6:32602082:A:G	-	Intergenic	rs9270815	A>G	10	1.30x10-15	0.69 [0.63-0.75]	0.171	29.78
6	32602333	chr6:32602333:T:A	-	Intergenic	rs13191764	T>A	8	1.62x10-14	0.45 [0.37-0.55]	0.885	0
6	32602370	chr6:32602370:A:G	-	Intergenic	rs2516052	A>G	10	3.55x10-85	1.80 [1.70-1.90]	0.279	17.83
6	32602407	chr6:32602407:C:T	-	Intergenic	rs2516051	C>T	10	3.55x10-85	1.80 [1.70-1.90]	0.279	17.83
6	32602624	chr6:32602624:G:A	-	Intergenic	rs2454138	G>A	10	3.55x10-85	1.80 [1.70-1.90]	0.279	17.83
6	32602640	chr6:32602640:A:C	-	Intergenic	rs2647062	A>C	10	4.90x10-87	2.03 [1.90-2.17]	0.931	0
6	32602796	chr6:32602796:C:T	-	Intergenic	rs558721	C>T	10	1.15x10-86	2.03 [1.90-2.16]	0.932	0
6	32603181	chr6:32603181:G:T	-	Intergenic	rs679242	G>T	10	9.59x10-87	2.03 [1.90-2.17]	0.932	0
6	32603182	chr6:32603182:C:T	-	Intergenic	rs679243	C>T	10	6.80x10-22	0.75 [0.71-0.79]	0.184	28.27
6	32603202	chr6:32603202:G:A	-	Intergenic	rs9270861	G>A	10	6.46x10-22	0.75 [0.71-0.79]	0.184	28.3
6	32603204	chr6:32603204:T:C	-	Intergenic	rs9270862	T>C	10	6.46x10-22	0.75 [0.71-0.79]	0.184	28.3

6	32603205	chr6:32603205:G:A	-	Intergenic	rs9270863	G>A	10	6.46x10-22	0.75 [0.71-0.79]	0.184	28.3
6	32603217	chr6:32603217:C:T	-	Intergenic	rs10947317	C>T	10	3.11x10-10	0.59 [0.50-0.69]	0.617	0
6	32603321	chr6:32603321:A:G	-	Intergenic	rs680061	A>G	10	6.16x10-22	0.75 [0.71-0.79]	0.193	27.26
6	32603333	chr6:32603333:G:A	-	Intergenic	rs2760990	G>A	10	1.13x10-86	2.03 [1.90-2.17]	0.933	0
6	32603345	chr6:32603345:C:T	-	Intergenic	rs2647066	C>T	10	9.59x10-87	2.03 [1.90-2.17]	0.932	0
6	32603378	chr6:32603378:T:C	-	Intergenic	rs9270869	T>C	10	4.78x10-22	0.75 [0.71-0.79]	0.177	29.15
6	32603383	chr6:32603383:A:G	-	Intergenic	rs9270871	A>G	10	5.84x10-22	0.75 [0.71-0.79]	0.178	29.02
6	32603389	chr6:32603389:A:G	-	Intergenic	rs680151	A>G	10	5.80x10-17	0.77 [0.73-0.82]	0.509	0
6	32604152	chr6:32604152:C:T	-	Intergenic	rs4367411	C>T	10	1.30x10-15	0.69 [0.63-0.75]	0.171	29.78
6	32604184	chr6:32604184:T:C	-	Intergenic	rs17425622	T>C	10	8.80x10-87	2.03 [1.90-2.17]	0.931	0
6	32604253	chr6:32604253:T:A	-	Intergenic	rs526784	T>A	10	3.55x10-85	1.80 [1.70-1.90]	0.279	17.83
6	32604271	chr6:32604271:G:T	-	Intergenic	rs585305	G>T	10	3.55x10-85	1.80 [1.70-1.90]	0.279	17.83
6	32604329	chr6:32604329:G:A	-	Intergenic	rs34102154	G>A	10	7.68x10-28	0.62 [0.57-0.67]	0.593	0
6	32604403	chr6:32604403:G:C	-	Intergenic	rs9270910	G>C	10	4.77x10-20	0.56 [0.50-0.64]	0.003	63.79
6	32604425	chr6:32604425:C:T	-	Intergenic	rs9270911	C>T	10	5.17x10-20	0.56 [0.50-0.63]	0.003	63.92
6	32604517	chr6:32604517:G:T	-	Intergenic	rs9270917	G>T	10	1.30x10-15	0.69 [0.63-0.75]	0.171	29.78
6	32604534	chr6:32604534:T:C	-	Intergenic	rs586610	T>C	10	5.53x10-85	1.80 [1.70-1.90]	0.33	12.31
6	32604628	chr6:32604628:C:T	-	Intergenic	rs9270923	C>T	10	1.30x10-15	0.69 [0.63-0.75]	0.171	29.78
6	32604684	chr6:32604684:G:T	-	Intergenic	rs9270928	G>T	10	1.30x10-15	0.69 [0.63-0.75]	0.171	29.78
6	32604965	chr6:32604965:C:G	-	Intergenic	rs13214858	C>G	8	1.62x10-14	0.45 [0.37-0.55]	0.885	0
6	32605127	chr6:32605127:T:A	-	Intergenic	rs574170	T>A	10	1.49x10-68	1.67 [1.58-1.76]	0.074	42.61
6	32605248	chr6:32605248:A:G	-	Intergenic	rs13215135	A>G	8	1.62x10-14	0.45 [0.37-0.55]	0.885	0
6	32605638	chr6:32605638:A:G	-	Intergenic	rs601945	A>G	10	5.30x10-85	2.02 [1.89-2.16]	0.924	0
6	32605785	chr6:32605785:T:C	-	Intergenic	rs602457	T>C	10	9.39x10-78	1.94 [1.81-2.07]	0.951	0
6	32606100	chr6:32606100:T:C	-	Intergenic	rs614348	T>C	10	2.43x10-68	1.67 [1.58-1.76]	0.073	42.72
6	32606132	chr6:32606132:C:A	-	Intergenic	rs9270980	C>A	10	1.45x10-15	0.69 [0.63-0.75]	0.174	29.48
6	32606157	chr6:32606157:C:T	-	Intergenic	rs543713	C>T	10	2.43x10-68	1.67 [1.58-1.76]	0.073	42.72
6	32606164	chr6:32606164:A:G	-	Intergenic	rs614437	A>G	10	4.76x10-17	0.77 [0.73-0.82]	0.501	0
6	32606214	chr6:32606214:T:G	-	Intergenic	rs9270984	T>G	10	1.30x10-15	0.69 [0.63-0.75]	0.171	29.78
6	32606283	chr6:32606283:A:C	-	Intergenic	rs9270986	A>C	10	1.30x10-15	0.69 [0.63-0.75]	0.171	29.78
6	32606331	chr6:32606331:G:A	-	Intergenic	rs34107399	G>A	8	1.62x10-14	0.45 [0.37-0.55]	0.885	0
6	32606394	chr6:32606394:G:C	-	Intergenic	rs615672	G>C	10	5.67x10-17	0.77 [0.73-0.82]	0.506	0
6	32606413	chr6:32606413:A:G	-	Intergenic	rs615698	A>G	10	5.67x10-17	0.77 [0.73-0.82]	0.506	0
6	32606427	chr6:32606427:G:A	-	Intergenic	rs615719	G>A	10	5.67x10-17	0.77 [0.73-0.82]	0.506	0
6	32606473	chr6:32606473:T:C	-	Intergenic	rs9270994	T>C	10	1.30x10-15	0.69 [0.63-0.75]	0.171	29.78
6	32606528	chr6:32606528:G:A	-	Intergenic	rs616187	G>A	10	9.81x10-16	0.78 [0.74-0.83]	0.684	0
6	32606597	chr6:32606597:G:A	-	Intergenic	rs9270997	G>A	10	1.30x10-15	0.69 [0.63-0.75]	0.171	29.78
6	32607179	chr6:32607179:A:G	-	Intergenic	rs145945003	A>G	9	1.67x10-08	0.61 [0.52-0.72]	0.778	0
6	32607548	chr6:32607548:A:G	-	Intergenic	rs2858867	A>G	10	5.67x10-17	0.77 [0.73-0.82]	0.506	0
6	32607592	chr6:32607592:G:T	-	Intergenic	rs9271055	G>T	10	2.19x10-15	0.69 [0.63-0.76]	0.19	27.64
6	32607601	chr6:32607601:T:C	-	Intergenic	rs9271056	T>C	10	2.19x10-15	0.69 [0.63-0.76]	0.19	27.64
6	32607614	chr6:32607614:G:C	-	Intergenic	rs2858866	G>C	10	5.67x10-17	0.77 [0.73-0.82]	0.506	0
6	32607655	chr6:32607655:C:T	-	Intergenic	rs113742050	C>T	9	2.14x10-08	1.42 [1.26-1.59]	0.555	0
6	32607716	chr6:32607716:C:G	-	Intergenic	rs486912	C>G	10	5.67x10-17	0.77 [0.73-0.82]	0.506	0
6	32607767	chr6:32607767:A:T	-	Intergenic	rs9271061	A>T	10	1.30x10-15	0.69 [0.63-0.75]	0.171	29.78
6	32607798	chr6:32607798:T:A	-	Intergenic	rs9271062	T>A	10	1.30x10-15	0.69 [0.63-0.75]	0.171	29.78

6	32607842	chr6:32607842:C:G	-	Intergenic	rs9271065	C>G	8	2.49x10-14	0.65 [0.58-0.72]	0.93	0
6	32607881	chr6:32607881:G:C	-	Intergenic	rs3021304	G>C	10	6.21x10-32	0.71 [0.67-0.75]	0.539	0
6	32607923	chr6:32607923:A:G	-	Intergenic	rs9271069	A>G	10	1.81x10-13	0.70 [0.64-0.77]	0.381	6.58
6	32607969	chr6:32607969:G:T	-	Intergenic	rs3021303	G>T	10	5.67x10-17	0.77 [0.73-0.82]	0.506	0
6	32608141	chr6:32608141:A:T	-	Intergenic	rs643889	A>T	10	1.52x10-68	1.67 [1.58-1.76]	0.079	41.86
6	32608206	chr6:32608206:A:G	-	Intergenic	rs12660719	A>G	9	1.67x10-08	0.61 [0.52-0.72]	0.778	0
6	32608232	chr6:32608232:T:G	-	Intergenic	rs482205	T>G	10	1.52x10-68	1.67 [1.58-1.76]	0.079	41.86
6	32608242	chr6:32608242:T:C	-	Intergenic	rs482162	T>C	10	1.52x10-68	1.67 [1.58-1.76]	0.079	41.86
6	32608287	chr6:32608287:G:C	-	Intergenic	rs482044	G>C	10	6.29x10-14	1.48 [1.34-1.64]	0.015	56
6	32608299	chr6:32608299:C:T	-	Intergenic	rs9271080	C>T	10	1.30x10-15	0.69 [0.63-0.75]	0.171	29.78
6	32608309	chr6:32608309:T:C	-	Intergenic	rs9271082	T>C	10	1.30x10-15	0.69 [0.63-0.75]	0.171	29.78
6	32608337	chr6:32608337:A:C	-	Intergenic	rs481245	A>C	10	5.67x10-17	0.77 [0.73-0.82]	0.506	0
6	32608368	chr6:32608368:C:A	-	Intergenic	rs481139	C>A	10	1.52x10-68	1.67 [1.58-1.76]	0.079	41.86
6	32608375	chr6:32608375:T:C	-	Intergenic	rs9271085	T>C	10	1.30x10-15	0.69 [0.63-0.75]	0.171	29.78
6	32608564	chr6:32608564:G:A	-	Intergenic	rs9271093	G>A	10	1.30x10-15	0.69 [0.63-0.75]	0.171	29.78
6	32608669	chr6:32608669:C:T	-	Intergenic	rs646159	C>T	10	5.55x10-17	0.77 [0.73-0.82]	0.51	0
6	32608761	chr6:32608761:T:C	-	Intergenic	rs566861	T>C	10	2.52x10-14	0.79 [0.74-0.84]	0.495	0
6	32608815	chr6:32608815:A:C	-	Intergenic	rs646984	A>C	10	2.40x10-61	1.62 [1.53-1.71]	0.072	42.84
6	32608849	chr6:32608849:G:A	-	Intergenic	rs647035	G>A	10	2.40x10-61	1.62 [1.53-1.71]	0.072	42.84
6	32608944	chr6:32608944:G:A	-	Intergenic	rs647455	G>A	10	2.53x10-14	0.79 [0.74-0.84]	0.495	0
6	32608949	chr6:32608949:C:T	-	Intergenic	rs647467	C>T	10	2.53x10-14	0.79 [0.74-0.84]	0.495	0
6	32608987	chr6:32608987:T:G	-	Intergenic	rs9271116	T>G	10	1.30x10-15	0.69 [0.63-0.75]	0.171	29.78
6	32609445	chr6:32609445:G:A	-	Intergenic	rs560530	G>A	10	5.23x10-71	1.84 [1.73-1.96]	0.632	0
6	32609446	chr6:32609446:G:T	-	Intergenic	rs542179215	G>T	9	1.67x10-08	0.61 [0.52-0.72]	0.778	0
6	32609603	chr6:32609603:A:G	-	Intergenic	rs660895	A>G	10	5.92x10-71	1.84 [1.73-1.96]	0.634	0
6	32609608	chr6:32609608:T:C	-	Intergenic	rs9271147	T>C	9	1.42x10-15	0.65 [0.59-0.72]	0.455	0
6	32609695	chr6:32609695:T:C	-	Intergenic	rs661330	T>C	10	5.92x10-71	1.84 [1.73-1.96]	0.634	0
6	32609720	chr6:32609720:C:T	-	Intergenic	rs536810	C>T	10	2.40x10-61	1.62 [1.53-1.71]	0.072	42.84
6	32609754	chr6:32609754:T:G	-	Intergenic	rs9271152	T>G	10	1.30x10-15	0.69 [0.63-0.75]	0.171	29.78
6	32609809	chr6:32609809:G:A	-	Intergenic	rs9271155	G>A	10	1.30x10-15	0.69 [0.63-0.75]	0.171	29.78
6	32609827	chr6:32609827:C:T	-	Intergenic	rs535852	C>T	10	2.40x10-61	1.62 [1.53-1.71]	0.072	42.84
6	32609869	chr6:32609869:A:G	-	Intergenic	rs9271160	A>G	10	1.30x10-15	0.69 [0.63-0.75]	0.171	29.78
6	32609936	chr6:32609936:T:C	-	Intergenic	rs9271161	T>C	10	1.30x10-15	0.69 [0.63-0.75]	0.171	29.78
6	32610081	chr6:32610081:G:A	-	Intergenic	rs673336	G>A	10	2.86x10-13	0.80 [0.75-0.85]	0.649	0
6	32610126	chr6:32610126:A:C	-	Intergenic	rs73728204	A>C	9	1.67x10-08	0.61 [0.52-0.72]	0.778	0
6	32610196	chr6:32610196:T:G	-	Intergenic	rs532965	T>G	10	1.57x10-86	2.02 [1.89-2.16]	0.94	0
6	32610275	chr6:32610275:G:A	-	Intergenic	rs532098	G>A	10	4.31x10-14	1.48 [1.34-1.64]	0.018	54.95
6	32610282	chr6:32610282:C:G	-	Intergenic	rs36233206	C>G	9	1.67x10-08	0.61 [0.52-0.72]	0.778	0
6	32610350	chr6:32610350:A:G	-	Intergenic	rs9271176	A>G	10	5.53x10-09	0.66 [0.57-0.75]	0.001	69.16
6	32610508	chr6:32610508:A:C	-	Intergenic	rs9271184	A>C	10	1.20x10-15	0.69 [0.63-0.75]	0.169	30
6	32610546	chr6:32610546:A:G	-	Intergenic	rs17211342	A>G	10	4.31x10-14	1.48 [1.34-1.64]	0.018	54.95
6	32610551	chr6:32610551:G:A	-	Intergenic	rs17204995	G>A	10	4.31x10-14	1.48 [1.34-1.64]	0.018	54.95
6	32610672	chr6:32610672:T:C	-	Intergenic	rs9271191	T>C	10	1.30x10-15	0.69 [0.63-0.75]	0.171	29.78
6	32610704	chr6:32610704:T:C	-	Intergenic	rs5021726	T>C	10	4.31x10-14	1.48 [1.34-1.64]	0.018	54.95
6	32610813	chr6:32610813:A:G	-	Intergenic	rs3997868	A>G	10	5.92x10-71	1.84 [1.73-1.96]	0.634	0
6	32610855	chr6:32610855:C:T	-	Intergenic	rs3997869	C>T	10	1.08x10-48	1.59 [1.50-1.69]	0.139	33.65

6	32610856	chr6:32610856:A:G	-	Intergenic	rs5021727	A>G	10	4.31x10-14	1.48 [1.34-1.64]	0.018	54.95
6	32610995	chr6:32610995:C:A	-	Intergenic	rs504594	C>A	10	3.55x10-85	2.02 [1.89-2.15]	0.935	0
6	32611108	chr6:32611108:T:C	-	Intergenic	rs9271203	T>C	10	1.30x10-15	0.69 [0.63-0.75]	0.171	29.78
6	32611181	chr6:32611181:G:A	-	Intergenic	rs502803	G>A	10	4.31x10-14	1.48 [1.34-1.64]	0.018	54.95
6	32611846	chr6:32611846:C:T	-	Intergenic	rs28752509	C>T	10	4.31x10-14	1.48 [1.34-1.64]	0.018	54.95
6	32611849	chr6:32611849:G:A	-	Intergenic	rs28752510	G>A	10	4.31x10-14	1.48 [1.34-1.64]	0.018	54.95
6	32611866	chr6:32611866:T:A	-	Intergenic	rs6917729	T>A	10	1.34x10-86	2.02 [1.90-2.16]	0.939	0
6	32611876	chr6:32611876:A:G	-	Intergenic	rs13191862	A>G	10	4.31x10-14	1.48 [1.34-1.64]	0.018	54.95
6	32611894	chr6:32611894:C:T	-	Intergenic	rs13191975	C>T	10	4.31x10-14	1.48 [1.34-1.64]	0.018	54.95
6	32611931	chr6:32611931:T:C	-	Intergenic	rs13207945	T>C	10	4.31x10-14	1.48 [1.34-1.64]	0.018	54.95
6	32611966	chr6:32611966:T:A	-	Intergenic	rs13208027	T>A	10	4.31x10-14	1.48 [1.34-1.64]	0.018	54.95
6	32611998	chr6:32611998:A:T	-	Intergenic	rs12523808	A>T	10	4.31x10-14	1.48 [1.34-1.64]	0.018	54.95
6	32612091	chr6:32612091:G:T	-	Intergenic	rs12527255	G>T	10	4.31x10-14	1.48 [1.34-1.64]	0.018	54.95
6	32612242	chr6:32612242:C:T	-	Intergenic	rs116714077	C>T	9	1.67x10-08	0.61 [0.52-0.72]	0.778	0
6	32612255	chr6:32612255:C:T	-	Intergenic	rs115035141	C>T	9	1.67x10-08	0.61 [0.52-0.72]	0.778	0
6	32612359	chr6:32612359:C:T	-	Intergenic	rs3104419	C>T	10	4.31x10-14	1.48 [1.34-1.64]	0.018	54.95
6	32612491	chr6:32612491:G:A	-	Intergenic	rs80343249	G>A	9	1.67x10-08	0.61 [0.52-0.72]	0.778	0
6	32612554	chr6:32612554:C:T	-	Intergenic	rs2858861	C>T	10	4.31x10-14	1.48 [1.34-1.64]	0.018	54.95
6	32612589	chr6:32612589:G:T	-	Intergenic	rs2858860	G>T	10	4.31x10-14	1.48 [1.34-1.64]	0.018	54.95
6	32612634	chr6:32612634:A:G	-	Intergenic	rs9271256	A>G	10	1.31x10-15	0.69 [0.63-0.75]	0.172	29.74
6	32612840	chr6:32612840:T:A	-	Intergenic	rs3997872	T>A	10	3.55x10-85	2.02 [1.89-2.15]	0.935	0
6	32612860	chr6:32612860:C:T	-	Intergenic	rs2395515	C>T	10	1.30x10-15	0.69 [0.63-0.75]	0.171	29.78
6	32612880	chr6:32612880:T:C	-	Intergenic	rs2395516	T>C	10	6.74x10-30	1.42 [1.34-1.51]	0.084	41.01
6	32612915	chr6:32612915:T:G	-	Intergenic	rs7451882	T>G	9	1.16x10-08	0.61 [0.51-0.72]	0.776	0
6	32612946	chr6:32612946:G:T	-	Intergenic	rs3997873	G>T	10	1.81x10-13	0.70 [0.64-0.77]	0.381	6.58
6	32612959	chr6:32612959:G:A	-	Intergenic	rs7449587	G>A	9	1.16x10-08	0.61 [0.51-0.72]	0.776	0
6	32613134	chr6:32613134:T:C	-	Intergenic	rs2894379	T>C	10	8.70x10-49	1.60 [1.50-1.69]	0.143	33.14
6	32613231	chr6:32613231:T:C	-	Intergenic	rs3129747	T>C	10	6.74x10-30	1.42 [1.34-1.51]	0.084	41.01
6	32613238	chr6:32613238:T:A	-	Intergenic	rs3129748	T>A	10	6.74x10-30	1.42 [1.34-1.51]	0.084	41.01
6	32613244	chr6:32613244:G:A	-	Intergenic	rs3104418	G>A	10	6.74x10-30	1.42 [1.34-1.51]	0.084	41.01
6	32613319	chr6:32613319:T:C	-	Intergenic	rs35759989	T>C	9	1.67x10-08	0.61 [0.52-0.72]	0.778	0
6	32613380	chr6:32613380:G:A	-	Intergenic	rs75765164	G>A	8	1.62x10-14	0.45 [0.37-0.55]	0.885	0
6	32613556	chr6:32613556:G:A	-	Intergenic	rs3129749	G>A	10	1.30x10-15	0.69 [0.63-0.75]	0.171	29.78
6	32613738	chr6:32613738:G:A	-	Intergenic	rs3129750	G>A	10	1.77x10-15	0.69 [0.63-0.76]	0.154	31.75
6	32613851	chr6:32613851:C:T	-	Intergenic	rs73728237	C>T	9	1.67x10-08	0.61 [0.52-0.72]	0.778	0
6	32613907	chr6:32613907:T:G	-	Intergenic	rs1966002	T>G	10	1.30x10-15	0.69 [0.63-0.75]	0.171	29.78
6	32614005	chr6:32614005:C:T	-	Intergenic	rs1966001	C>T	10	1.30x10-15	0.69 [0.63-0.75]	0.171	29.78
6	32614145	chr6:32614145:C:T	-	Intergenic	rs522308	C>T	10	1.06x10-83	1.79 [1.69-1.89]	0.189	27.71
6	32614152	chr6:32614152:A:G	-	Intergenic	rs34704034	A>G	9	1.67x10-08	0.61 [0.52-0.72]	0.778	0
6	32614196	chr6:32614196:G:A	-	Intergenic	rs521539	G>A	10	5.92x10-71	1.84 [1.73-1.96]	0.634	0
6	32614219	chr6:32614219:C:A	-	Intergenic	rs56361357	C>A	9	2.88x10-08	0.62 [0.52-0.73]	0.797	0
6	32614412	chr6:32614412:A:C	-	Intergenic	rs3129751	A>C	10	3.55x10-85	2.02 [1.89-2.15]	0.935	0
6	32614543	chr6:32614543:A:T	-	Intergenic	rs73728240	A>T	9	1.67x10-08	0.61 [0.52-0.72]	0.778	0
6	32614657	chr6:32614657:C:T	-	Intergenic	rs28614783	C>T	9	1.67x10-08	0.61 [0.52-0.72]	0.778	0
6	32614835	chr6:32614835:G:A	-	Intergenic	rs3104414	G>A	10	2.40x10-26	1.38 [1.31-1.46]	0.088	40.51
6	32614873	chr6:32614873:C:G	-	Intergenic	rs3104413	C>G	10	1.57x10-86	2.02 [1.89-2.16]	0.94	0

6	32615162	chr6:32615162:G:T	-	Intergenic	rs201424733	G>T	9	1.67x10-08	0.61 [0.52-0.72]	0.778	0
6	32615163	chr6:32615163:G:T	-	Intergenic	rs199640993	G>T	9	1.67x10-08	0.61 [0.52-0.72]	0.778	0
6	32615250	chr6:32615250:G:C	-	Intergenic	rs3129753	G>C	10	1.57x10-86	2.02 [1.89-2.16]	0.94	0
6	32615269	chr6:32615269:A:G	-	Intergenic	rs3129754	A>G	10	1.56x10-11	1.22 [1.15-1.29]	0.27	18.83
6	32615274	chr6:32615274:C:T	-	Intergenic	rs35484705	C>T	10	1.56x10-11	1.22 [1.15-1.29]	0.27	18.83
6	32615279	chr6:32615279:C:T	-	Intergenic	rs35558953	C>T	10	1.56x10-11	1.22 [1.15-1.29]	0.27	18.83
6	32615280	chr6:32615280:A:G	-	Intergenic	rs3129755	A>G	10	8.76x10-25	0.74 [0.70-0.78]	0.277	18.07
6	32615286	chr6:32615286:A:G	-	Intergenic	rs3129756	A>G	10	1.56x10-11	1.22 [1.15-1.29]	0.27	18.83
6	32615322	chr6:32615322:A:G	-	Intergenic	rs6605556	A>G	10	1.57x10-86	2.02 [1.89-2.16]	0.94	0
6	32615348	chr6:32615348:T:C	-	Intergenic	rs4959103	T>C	10	2.29x10-09	1.19 [1.13-1.26]	0.192	27.41
6	32615352	chr6:32615352:T:C	-	Intergenic	rs4959104	T>C	10	2.29x10-09	1.19 [1.13-1.26]	0.192	27.41
6	32615369	chr6:32615369:C:T	-	Intergenic	rs4959105	C>T	10	3.17x10-08	1.32 [1.20-1.46]	0.046	47.63
6	32615382	chr6:32615382:T:C	-	Intergenic	rs4959106	T>C	10	2.69x10-09	1.19 [1.13-1.26]	0.197	26.85
6	32615417	chr6:32615417:G:T	-	Intergenic	rs6931044	G>T	10	1.56x10-11	1.22 [1.15-1.29]	0.27	18.83
6	32615423	chr6:32615423:G:A	-	Intergenic	rs6941393	G>A	10	5.92x10-71	1.84 [1.73-1.96]	0.634	0
6	32615429	chr6:32615429:G:A	-	Intergenic	rs6941395	G>A	10	1.57x10-86	2.02 [1.89-2.16]	0.94	0
6	32615435	chr6:32615435:A:G	-	Intergenic	rs28383221	A>G	10	8.05x10-22	0.75 [0.71-0.79]	0.323	13.02
6	32615522	chr6:32615522:C:T	-	Intergenic	rs34850435	C>T	10	2.25x10-09	1.19 [1.13-1.26]	0.194	27.22
6	32615551	chr6:32615551:T:C	-	Intergenic	rs9271344	T>C	10	3.87x10-08	0.85 [0.80-0.90]	0.18	28.8
6	32615580	chr6:32615580:A:T	-	Intergenic	rs6931277	A>T	10	1.57x10-86	2.02 [1.89-2.16]	0.94	0
6	32615626	chr6:32615626:A:G	-	Intergenic	rs35128369	A>G	10	2.29x10-09	1.19 [1.13-1.26]	0.192	27.41
6	32615649	chr6:32615649:T:G	-	Intergenic	rs35029150	T>G	10	2.29x10-09	1.19 [1.13-1.26]	0.192	27.41
6	32615684	chr6:32615684:C:T	-	Intergenic	rs35928237	C>T	10	2.29x10-09	1.19 [1.13-1.26]	0.192	27.41
6	32615691	chr6:32615691:C:T	-	Intergenic	rs9271346	C>T	10	2.74x10-27	0.62 [0.57-0.67]	0.54	0
6	32615700	chr6:32615700:C:T	-	Intergenic	rs28383223	C>T	10	7.44x10-22	0.75 [0.71-0.79]	0.319	13.46
6	32615713	chr6:32615713:A:G	-	Intergenic	rs35534739	A>G	10	2.29x10-09	1.19 [1.13-1.26]	0.192	27.41
6	32615719	chr6:32615719:G:A	-	Intergenic	rs34341844	G>A	10	2.29x10-09	1.19 [1.13-1.26]	0.192	27.41
6	32615752	chr6:32615752:G:A	-	Intergenic	rs6941972	G>A	10	5.92x10-71	1.84 [1.73-1.96]	0.634	0
6	32615766	chr6:32615766:A:G	-	Intergenic	rs9271347	A>G	10	1.81x10-13	0.70 [0.64-0.77]	0.381	6.58
6	32615780	chr6:32615780:C:G	-	Intergenic	rs34599306	C>G	10	2.29x10-09	1.19 [1.13-1.26]	0.192	27.41
6	32615799	chr6:32615799:T:C	-	Intergenic	rs28752514	T>C	10	7.44x10-22	0.75 [0.71-0.79]	0.319	13.46
6	32615807	chr6:32615807:A:G	-	Intergenic	rs115625939	A>G	9	8.61x10-14	0.68 [0.61-0.75]	0.947	0
6	32615826	chr6:32615826:G:A	-	Intergenic	rs28752515	G>A	10	7.44x10-22	0.75 [0.71-0.79]	0.319	13.46
6	32615828	chr6:32615828:A:G	-	Intergenic	rs34341095	A>G	10	2.29x10-09	1.19 [1.13-1.26]	0.192	27.41
6	32615833	chr6:32615833:C:A	-	Intergenic	rs35656642	C>A	10	1.54x10-11	1.22 [1.15-1.29]	0.27	18.77
6	32615834	chr6:32615834:A:G	-	Intergenic	rs28752516	A>G	10	8.62x10-25	0.74 [0.70-0.78]	0.278	17.91
6	32615876	chr6:32615876:A:G	-	Intergenic	rs28383224	A>G	10	7.44x10-22	0.75 [0.71-0.79]	0.319	13.46
6	32615900	chr6:32615900:T:C	-	Intergenic	rs36124427	T>C	10	2.29x10-09	1.19 [1.13-1.26]	0.192	27.41
6	32615905	chr6:32615905:A:C	-	Intergenic	rs34136174	A>C	10	2.29x10-09	1.19 [1.13-1.26]	0.192	27.41
6	32616006	chr6:32616006:A:G	-	Intergenic	rs77015620	A>G	9	1.67x10-08	0.61 [0.52-0.72]	0.778	0
6	32616036	chr6:32616036:G:A	-	Intergenic	rs1846190	G>A	10	6.34x10-10	1.38 [1.25-1.52]	0.046	47.57
6	32616043	chr6:32616043:G:T	-	Intergenic	rs1281935	G>T	10	4.64x10-17	1.57 [1.42-1.73]	0.214	24.95
6	32616053	chr6:32616053:A:G	-	Intergenic	rs9271349	A>G	10	2.73x10-27	0.62 [0.57-0.67]	0.539	0
6	32616083	chr6:32616083:C:G	-	Intergenic	rs9271351	C>G	10	1.81x10-13	0.70 [0.64-0.77]	0.381	6.58
6	32616142	chr6:32616142:T:C	-	Intergenic	rs33964890	T>C	10	1.37x10-13	1.48 [1.34-1.63]	0.041	48.6
6	32616153	chr6:32616153:T:C	-	Intergenic	rs35542934	T>C	10	2.29x10-09	1.19 [1.13-1.26]	0.192	27.41

6	32616202	chr6:32616202:A:G	-	Intergenic	rs33915496	A>G	10	3.38x10-08	1.33 [1.20-1.46]	0.045	47.84
6	32616237	chr6:32616237:T:C	-	Intergenic	rs9271353	T>C	10	1.98x10-13	0.70 [0.64-0.77]	0.385	6.13
6	32616238	chr6:32616238:A:G	-	Intergenic	rs34985232	A>G	10	2.29x10-09	1.19 [1.13-1.26]	0.192	27.41
6	32616289	chr6:32616289:C:T	-	Intergenic	rs9271354	C>T	10	1.76x10-13	0.70 [0.64-0.77]	0.393	5.16
6	32616311	chr6:32616311:T:C	-	Intergenic	rs28383230	T>C	10	8.96x10-22	0.75 [0.71-0.80]	0.327	12.62
6	32616322	chr6:32616322:C:T	-	Intergenic	rs28383231	C>T	10	8.40x10-22	0.75 [0.71-0.79]	0.324	12.91
6	32616356	chr6:32616356:T:C	-	Intergenic	rs73730369	T>C	9	1.67x10-08	0.61 [0.52-0.72]	0.778	0
6	32616376	chr6:32616376:G:A	-	Intergenic	rs28383233	G>A	10	8.40x10-22	0.75 [0.71-0.79]	0.324	12.91
6	32616411	chr6:32616411:T:G	-	Intergenic	rs28383236	T>G	10	8.33x10-22	0.75 [0.71-0.79]	0.325	12.87
6	32616414	chr6:32616414:T:C	-	Intergenic	rs34077986	T>C	10	2.29x10-09	1.19 [1.13-1.26]	0.192	27.41
6	32616460	chr6:32616460:C:T	-	Intergenic	rs6930731	C>T	8	2.12x10-14	0.45 [0.37-0.55]	0.869	0
6	32616518	chr6:32616518:A:G	-	Intergenic	rs34537691	A>G	10	2.29x10-09	1.19 [1.13-1.26]	0.192	27.41
6	32616536	chr6:32616536:C:G	-	Intergenic	rs33932178	C>G	10	3.59x10-08	1.33 [1.20-1.46]	0.044	48.03
6	32616542	chr6:32616542:C:A	-	Intergenic	rs28383241	C>A	10	8.40x10-22	0.75 [0.71-0.79]	0.324	12.91
6	32616548	chr6:32616548:A:G	-	Intergenic	rs28383242	A>G	10	8.27x10-22	0.75 [0.71-0.79]	0.325	12.79
6	32616553	chr6:32616553:C:A	-	Intergenic	rs28359884	C>A	10	2.29x10-09	1.19 [1.13-1.26]	0.192	27.41
6	32616569	chr6:32616569:C:A	-	Intergenic	rs34028938	C>A	10	2.34x10-09	1.19 [1.13-1.26]	0.193	27.31
6	32616578	chr6:32616578:A:G	-	Intergenic	rs35940802	A>G	10	2.29x10-09	1.19 [1.13-1.26]	0.192	27.41
6	32616804	chr6:32616804:C:T	-	Intergenic	rs73730372	C>T	9	8.61x10-14	0.68 [0.61-0.75]	0.947	0
6	32616832	chr6:32616832:A:C	-	Intergenic	rs3129757	A>C	10	1.88x10-13	0.70 [0.64-0.77]	0.383	6.39
6	32616848	chr6:32616848:A:G	-	Intergenic	rs3129758	A>G	10	3.20x10-13	0.62 [0.55-0.71]	0.003	64.41
6	32616916	chr6:32616916:C:G	-	Intergenic	rs510205	C>G	10	1.13x10-72	1.86 [1.74-1.98]	0.628	0
6	32616931	chr6:32616931:A:G	-	Intergenic	rs73730375	A>G	9	1.67x10-08	0.61 [0.52-0.72]	0.778	0
6	32616992	chr6:32616992:A:G	-	Intergenic	rs685244	A>G	10	8.40x10-22	0.75 [0.71-0.79]	0.324	12.91
6	32617103	chr6:32617103:A:G	-	Intergenic	rs73730377	A>G	9	8.61x10-14	0.68 [0.61-0.75]	0.947	0
6	32617141	chr6:32617141:A:G	-	Intergenic	rs28752526	A>G	10	8.00x10-76	1.73 [1.63-1.82]	0.253	20.61
6	32617149	chr6:32617149:A:G	-	Intergenic	rs34278657	A>G	9	1.67x10-08	0.61 [0.52-0.72]	0.778	0
6	32617151	chr6:32617151:C:T	-	Intergenic	rs28752527	C>T	10	8.29x10-22	0.75 [0.71-0.79]	0.324	12.96
6	32617175	chr6:32617175:A:G	-	Intergenic	rs685810	A>G	10	4.57x10-75	1.72 [1.63-1.82]	0.26	19.87
6	32617192	chr6:32617192:A:G	-	Intergenic	rs73730378	A>G	9	1.67x10-08	0.61 [0.52-0.72]	0.778	0
6	32617200	chr6:32617200:T:G	-	Intergenic	rs113016235	T>G	9	8.61x10-14	0.68 [0.61-0.75]	0.947	0
6	32617275	chr6:32617275:C:A	-	Intergenic	rs13213778	C>A	8	2.12x10-14	0.45 [0.37-0.55]	0.869	0
6	32617278	chr6:32617278:A:G	-	Intergenic	rs686250	A>G	10	4.19x10-11	0.82 [0.77-0.87]	0.076	42.31
6	32617294	chr6:32617294:G:C	-	Intergenic	rs113243185	G>C	9	8.61x10-14	0.68 [0.61-0.75]	0.947	0
6	32617725	chr6:32617725:C:T	-	Intergenic	rs76509496	C>T	9	1.67x10-08	0.61 [0.52-0.72]	0.778	0
6	32617727	chr6:32617727:G:C	-	Intergenic	rs3129761	G>C	10	3.30x10-11	1.47 [1.32-1.64]	0.004	63.28
6	32617904	chr6:32617904:A:T	-	Intergenic	rs13217507	A>T	8	2.12x10-14	0.45 [0.37-0.55]	0.869	0
6	32617919	chr6:32617919:A:C	-	Intergenic	rs13217512	A>C	8	2.81x10-14	0.46 [0.37-0.55]	0.866	0
6	32617972	chr6:32617972:G:A	-	Intergenic	rs75483390	G>A	9	1.67x10-08	0.61 [0.52-0.72]	0.778	0
6	32617978	chr6:32617978:T:A	-	Intergenic	rs3129762	T>A	10	2.06x10-13	0.70 [0.64-0.77]	0.384	6.21
6	32618190	chr6:32618190:A:G	-	Intergenic	rs3104412	A>G	10	3.30x10-11	1.47 [1.32-1.64]	0.004	63.28
6	32618242	chr6:32618242:A:G	-	Intergenic	rs3135005	A>G	10	2.06x10-13	0.70 [0.64-0.77]	0.384	6.21
6	32618445	chr6:32618445:G:A	-	Intergenic	rs11759846	G>A	10	1.73x10-12	0.60 [0.52-0.69]	0.889	0
6	32618459	chr6:32618459:C:A	-	Intergenic	rs11751024	C>A	10	8.19x10-16	0.79 [0.74-0.83]	0.334	11.81
6	32618898	chr6:32618898:T:C	-	Intergenic	rs35805541	T>C	9	1.67x10-08	0.61 [0.52-0.72]	0.778	0
6	32618955	chr6:32618955:G:C	-	Intergenic	rs9271363	G>C	10	6.59x10-16	0.70 [0.64-0.76]	0.253	20.65

6	32619017	chr6:32619017:T:G	-	Intergenic	rs9271365	T>G	10	1.43x10-11	1.51 [1.34-1.70]	0.001	66.6
6	32619077	chr6:32619077:G:A	-	Intergenic	rs9271366	G>A	10	1.61x10-13	0.70 [0.64-0.77]	0.405	3.85
6	32619145	chr6:32619145:T:C	-	Intergenic	rs13207893	T>C	9	3.38x10-21	0.58 [0.52-0.65]	0.44	0
6	32619157	chr6:32619157:G:A	-	Intergenic	rs9271368	G>A	10	1.45x10-12	0.58 [0.50-0.67]	0	74.53
6	32619184	chr6:32619184:A:T	-	Intergenic	rs114812135	A>T	8	2.12x10-14	0.45 [0.37-0.55]	0.869	0
6	32619221	chr6:32619221:C:G	-	Intergenic	rs607929	C>G	10	8.45x10-34	1.42 [1.35-1.50]	0.602	0
6	32619264	chr6:32619264:G:A	-	Intergenic	rs9271374	G>A	10	1.09x10-25	0.65 [0.60-0.70]	0.278	17.93
6	32619273	chr6:32619273:T:C	-	Intergenic	rs28464365	T>C	10	3.25x10-12	0.60 [0.52-0.69]	0.882	0
6	32619290	chr6:32619290:G:A	-	Intergenic	rs9271375	G>A	10	6.62x10-10	0.69 [0.62-0.77]	0.002	65.42
6	32619313	chr6:32619313:C:T	-	Intergenic	rs28383312	C>T	10	8.50x10-23	1.65 [1.50-1.82]	0.027	52.14
6	32619336	chr6:32619336:G:A	-	Intergenic	rs9271376	G>A	10	1.85x10-29	0.66 [0.62-0.71]	0.434	0.4
6	32619340	chr6:32619340:C:G	-	Intergenic	rs28383313	C>G	10	1.46x10-83	1.78 [1.69-1.88]	0.158	31.32
6	32619380	chr6:32619380:A:G	-	Intergenic	rs28688825	A>G	10	1.04x10-25	0.61 [0.56-0.67]	0.971	0
6	32619388	chr6:32619388:T:G	-	Intergenic	rs9271377	T>G	10	4.01x10-10	0.83 [0.78-0.88]	0.168	30.19
6	32619436	chr6:32619436:T:C	-	Intergenic	rs28383314	T>C	10	5.01x10-11	0.82 [0.77-0.87]	0.099	38.83
6	32619458	chr6:32619458:C:T	-	Intergenic	rs28533694	C>T	10	1.25x10-16	0.62 [0.56-0.69]	0.032	50.71
6	32619613	chr6:32619613:T:C	-	Intergenic	rs28391139	T>C	8	2.12x10-14	0.45 [0.37-0.55]	0.869	0
6	32619759	chr6:32619759:A:G	-	Intergenic	rs9271403	A>G	10	8.50x10-23	1.65 [1.50-1.82]	0.027	52.14
6	32619763	chr6:32619763:A:T	-	Intergenic	rs80195381	A>T	9	1.67x10-08	0.61 [0.52-0.72]	0.778	0
6	32619776	chr6:32619776:T:A	-	Intergenic	rs9271404	T>A	10	8.50x10-23	1.65 [1.50-1.82]	0.027	52.14
6	32619811	chr6:32619811:A:G	-	Intergenic	rs9271406	A>G	10	2.02x10-39	1.46 [1.39-1.55]	0.511	0
6	32619836	chr6:32619836:C:T	-	Intergenic	rs9271408	C>T	10	6.63x10-83	1.78 [1.69-1.88]	0.178	29.01
6	32619851	chr6:32619851:G:C	-	Intergenic	rs9271409	G>C	10	6.63x10-83	1.78 [1.69-1.88]	0.178	29.01
6	32619884	chr6:32619884:G:A	-	Intergenic	rs9271411	G>A	10	6.63x10-83	1.78 [1.69-1.88]	0.178	29.01
6	32619991	chr6:32619991:G:A	-	Intergenic	rs9271413	G>A	10	1.61x10-13	0.70 [0.64-0.77]	0.405	3.85
6	32620005	chr6:32620005:C:T	-	Intergenic	rs9271414	C>T	10	1.03x10-09	0.60 [0.51-0.71]	0	77.37
6	32620009	chr6:32620009:C:T	-	Intergenic	rs9271415	C>T	10	1.03x10-09	0.60 [0.51-0.71]	0	77.37
6	32620035	chr6:32620035:G:A	-	Intergenic	rs9271416	G>A	10	6.63x10-83	1.78 [1.69-1.88]	0.178	29.01
6	32620058	chr6:32620058:C:A	-	Intergenic	rs71542419	C>A	10	3.51x10-12	0.70 [0.64-0.78]	0.891	0
6	32620069	chr6:32620069:C:T	-	Intergenic	rs9271419	C>T	10	5.37x10-13	1.49 [1.34-1.65]	0.007	60.21
6	32620075	chr6:32620075:A:G	-	Intergenic	rs9271420	A>G	10	9.83x10-13	0.60 [0.53-0.69]	0	71.35
6	32620082	chr6:32620082:T:C	-	Intergenic	rs9271421	T>C	8	2.93x10-14	0.65 [0.58-0.72]	0.929	0
6	32620092	chr6:32620092:A:G	-	Intergenic	rs34976781	A>G	10	3.51x10-12	0.70 [0.64-0.78]	0.891	0
6	32620118	chr6:32620118:A:G	-	Intergenic	rs11756432	A>G	9	1.67x10-08	0.61 [0.52-0.72]	0.778	0
6	32620120	chr6:32620120:G:T	-	Intergenic	rs9271423	G>T	10	1.61x10-13	0.70 [0.64-0.77]	0.405	3.85
6	32620172	chr6:32620172:C:T	-	Intergenic	rs11751846	C>T	9	1.67x10-08	0.61 [0.52-0.72]	0.778	0
6	32620189	chr6:32620189:T:C	-	Intergenic	rs1281931	T>C	10	4.47x10-17	1.57 [1.42-1.73]	0.216	24.69
6	32620196	chr6:32620196:G:A	-	Intergenic	rs9271426	G>A	10	6.63x10-83	1.78 [1.69-1.88]	0.178	29.01
6	32620208	chr6:32620208:A:G	-	Intergenic	rs9271429	A>G	10	5.87x10-18	0.69 [0.64-0.75]	0.191	27.45
6	32620217	chr6:32620217:A:G	-	Intergenic	rs34405843	A>G	10	3.51x10-12	0.70 [0.64-0.78]	0.891	0
6	32620225	chr6:32620225:T:C	-	Intergenic	rs9271430	T>C	10	9.82x10-83	1.78 [1.68-1.88]	0.182	28.51
6	32620230	chr6:32620230:T:C	-	Intergenic	rs34304006	T>C	10	3.51x10-12	0.70 [0.64-0.78]	0.891	0
6	32620254	chr6:32620254:G:A	-	Intergenic	rs9271431	G>A	10	5.31x10-13	1.49 [1.34-1.65]	0.007	60.17
6	32620292	chr6:32620292:C:T	-	Intergenic	rs9271435	C>T	10	5.37x10-13	1.49 [1.34-1.65]	0.007	60.21
6	32620303	chr6:32620303:T:C	-	Intergenic	rs35372933	T>C	8	2.12x10-14	0.45 [0.37-0.55]	0.869	0
6	32620333	chr6:32620333:C:A	-	Intergenic	rs9271436	C>A	10	1.02x10-12	0.60 [0.53-0.69]	0	71.44

6	32620416	chr6:32620416:A:G	-	Intergenic	rs9271438	A>G	10	6.63x10-83	1.78 [1.69-1.88]	0.178	29.01
6	32620424	chr6:32620424:A:G	-	Intergenic	rs35945734	A>G	9	1.87x10-09	0.63 [0.54-0.73]	0.716	0
6	32620439	chr6:32620439:A:G	-	Intergenic	rs71542420	A>G	8	2.12x10-14	0.45 [0.37-0.55]	0.869	0
6	32620463	chr6:32620463:C:T	-	Intergenic	rs9271440	C>T	10	1.03x10-09	0.60 [0.51-0.71]	0	77.37
6	32620468	chr6:32620468:T:C	-	Intergenic	rs9271441	T>C	10	1.03x10-12	0.60 [0.53-0.69]	0	71.39
6	32620567	chr6:32620567:A:G	-	Intergenic	rs9271446	A>G	10	9.27x10-13	0.60 [0.53-0.69]	0	71.25
6	32620580	chr6:32620580:G:A	-	Intergenic	rs9271447	G>A	10	1.06x10-22	1.65 [1.50-1.82]	0.026	52.39
6	32620591	chr6:32620591:G:A	-	Intergenic	rs9271448	G>A	10	9.24x10-23	1.65 [1.50-1.81]	0.028	51.97
6	32620608	chr6:32620608:T:A	-	Intergenic	rs9271452	T>A	10	4.58x10-19	1.58 [1.43-1.74]	0.027	52.05
6	32620645	chr6:32620645:C:T	-	Intergenic	rs9271454	C>T	10	8.50x10-23	1.65 [1.50-1.82]	0.027	52.14
6	32620646	chr6:32620646:T:G	-	Intergenic	rs9271455	T>G	10	9.29x10-23	1.65 [1.50-1.82]	0.027	52.26
6	32620655	chr6:32620655:A:T	-	Intergenic	rs34697429	A>T	9	1.87x10-09	0.63 [0.54-0.73]	0.716	0
6	32620658	chr6:32620658:C:T	-	Intergenic	rs9271456	C>T	10	8.50x10-23	1.65 [1.50-1.82]	0.027	52.14
6	32620669	chr6:32620669:C:T	-	Intergenic	rs9271457	C>T	10	1.03x10-09	0.60 [0.51-0.71]	0	77.37
6	32620672	chr6:32620672:G:T	-	Intergenic	rs28456694	G>T	9	1.87x10-09	0.63 [0.54-0.73]	0.716	0
6	32620676	chr6:32620676:C:A	-	Intergenic	rs9271458	C>A	10	8.50x10-23	1.65 [1.50-1.82]	0.027	52.14
6	32620680	chr6:32620680:T:C	-	Intergenic	rs9271459	T>C	10	2.55x10-30	0.72 [0.68-0.76]	0.598	0
6	32620683	chr6:32620683:G:A	-	Intergenic	rs28592295	G>A	9	1.87x10-09	0.63 [0.54-0.73]	0.716	0
6	32620723	chr6:32620723:G:A	-	Intergenic	rs9271461	G>A	10	8.50x10-23	1.65 [1.50-1.82]	0.027	52.14
6	32620758	chr6:32620758:A:T	-	Intergenic	rs9271463	A>T	10	1.03x10-09	0.60 [0.51-0.71]	0	77.37
6	32620767	chr6:32620767:A:T	-	Intergenic	rs9271464	A>T	10	6.63x10-83	1.78 [1.69-1.88]	0.178	29.01
6	32620777	chr6:32620777:C:T	-	Intergenic	rs9271465	C>T	10	6.63x10-83	1.78 [1.69-1.88]	0.178	29.01
6	32620829	chr6:32620829:C:G	-	Intergenic	rs9271466	C>G	10	2.24x10-15	0.69 [0.63-0.76]	0.174	29.4
6	32620836	chr6:32620836:T:C	-	Intergenic	rs9271467	T>C	10	1.08x10-09	0.60 [0.51-0.71]	0	77.21
6	32620839	chr6:32620839:G:A	-	Intergenic	rs9271468	G>A	10	1.08x10-09	0.60 [0.51-0.71]	0	77.21
6	32620853	chr6:32620853:T:C	-	Intergenic	rs9271469	T>C	10	6.63x10-83	1.78 [1.69-1.88]	0.178	29.01
6	32620885	chr6:32620885:C:T	-	Intergenic	rs9271470	C>T	10	6.63x10-83	1.78 [1.69-1.88]	0.178	29.01
6	32620923	chr6:32620923:C:T	-	Intergenic	rs9271472	C>T	10	6.63x10-83	1.78 [1.69-1.88]	0.178	29.01
6	32620930	chr6:32620930:G:A	-	Intergenic	rs9271474	G>A	10	6.63x10-83	1.78 [1.69-1.88]	0.178	29.01
6	32620981	chr6:32620981:T:C	-	Intergenic	rs11751439	T>C	9	1.67x10-08	0.61 [0.52-0.72]	0.778	0
6	32621031	chr6:32621031:A:T	-	Intergenic	rs9271475	A>T	10	1.03x10-09	0.60 [0.51-0.71]	0	77.37
6	32621038	chr6:32621038:C:T	-	Intergenic	rs9271477	C>T	10	1.03x10-09	0.60 [0.51-0.71]	0	77.37
6	32621044	chr6:32621044:A:G	-	Intergenic	rs9271478	A>G	10	1.03x10-09	0.60 [0.51-0.71]	0	77.37
6	32621053	chr6:32621053:G:A	-	Intergenic	rs9271479	G>A	10	1.03x10-09	0.60 [0.51-0.71]	0	77.37
6	32621054	chr6:32621054:G:A	-	Intergenic	rs9271480	G>A	10	7.51x10-83	1.78 [1.69-1.88]	0.174	29.43
6	32621100	chr6:32621100:A:G	-	Intergenic	rs9271481	A>G	10	1.61x10-13	0.70 [0.64-0.77]	0.405	3.85
6	32621103	chr6:32621103:C:T	-	Intergenic	rs9271482	C>T	10	1.03x10-09	0.60 [0.51-0.71]	0	77.37
6	32621105	chr6:32621105:G:T	-	Intergenic	rs9271483	G>T	10	1.03x10-09	0.60 [0.51-0.71]	0	77.37
6	32621144	chr6:32621144:G:T	-	Intergenic	rs11758307	G>T	9	1.72x10-08	0.61 [0.52-0.72]	0.779	0
6	32621162	chr6:32621162:A:T	-	Intergenic	rs28699237	A>T	8	2.12x10-14	0.45 [0.37-0.55]	0.869	0
6	32621176	chr6:32621176:A:G	-	Intergenic	rs9271485	A>G	10	6.42x10-83	1.78 [1.69-1.88]	0.178	28.97
6	32621203	chr6:32621203:C:T	-	Intergenic	rs9271486	C>T	10	6.63x10-83	1.78 [1.69-1.88]	0.178	29.01
6	32621208	chr6:32621208:G:T	-	Intergenic	rs9271487	G>T	10	1.03x10-09	0.60 [0.51-0.71]	0	77.37
6	32621223	chr6:32621223:G:T	-	Intergenic	rs9271488	G>T	10	6.63x10-83	1.78 [1.69-1.88]	0.178	29.01
6	32621274	chr6:32621274:C:A	-	Intergenic	rs9271489	C>A	10	6.50x10-09	0.84 [0.79-0.89]	0.188	27.8
6	32621283	chr6:32621283:A:G	-	Intergenic	rs9271490	A>G	10	1.03x10-09	0.60 [0.51-0.71]	0	77.37

6	32621284	chr6:32621284:A:G	-	Intergenic	rs9271491	A>G	10	1.03x10-09	0.60 [0.51-0.71]	0	77.37
6	32621318	chr6:32621318:A:G	-	Intergenic	rs28669773	A>G	9	1.87x10-09	0.63 [0.54-0.73]	0.716	0
6	32621324	chr6:32621324:T:G	-	Intergenic	rs34846487	T>G	8	2.12x10-14	0.45 [0.37-0.55]	0.869	0
6	32621345	chr6:32621345:A:C	-	Intergenic	rs9271492	A>C	10	1.00x10-18	0.77 [0.72-0.81]	0.433	0.47
6	32621381	chr6:32621381:A:G	-	Intergenic	rs9271493	A>G	10	1.03x10-09	0.60 [0.51-0.71]	0	77.37
6	32621440	chr6:32621440:A:T	-	Intergenic	rs34104805	A>T	9	1.26x10-23	0.51 [0.45-0.58]	1	0
6	32621465	chr6:32621465:C:T	-	Intergenic	rs9271495	C>T	10	1.05x10-09	0.60 [0.51-0.71]	0	77.41
6	32621467	chr6:32621467:C:T	-	Intergenic	rs9271496	C>T	10	1.05x10-09	0.60 [0.51-0.71]	0	77.41
6	32621468	chr6:32621468:A:T	-	Intergenic	rs9271497	A>T	10	6.63x10-83	1.78 [1.69-1.88]	0.178	29.01
6	32621489	chr6:32621489:G:A	-	Intergenic	rs35597309	G>A	8	2.16x10-14	0.45 [0.37-0.55]	0.867	0
6	32621505	chr6:32621505:G:A	-	Intergenic	rs9271498	G>A	10	6.63x10-83	1.78 [1.69-1.88]	0.178	29.01
6	32621514	chr6:32621514:C:T	-	Intergenic	rs9271499	C>T	10	6.89x10-14	1.52 [1.37-1.69]	0.007	60.41
6	32621523	chr6:32621523:C:T	-	Intergenic	rs9271500	C>T	10	2.93x10-08	1.38 [1.23-1.54]	0.003	64.14
6	32621524	chr6:32621524:A:G	-	Intergenic	rs9271501	A>G	10	2.59x10-08	1.38 [1.24-1.54]	0.003	63.9
6	32621528	chr6:32621528:G:A	-	Intergenic	rs9271502	G>A	10	5.44x10-14	1.52 [1.37-1.69]	0.007	60.12
6	32621549	chr6:32621549:C:A	-	Intergenic	rs9271503	C>A	10	5.98x10-83	1.78 [1.69-1.88]	0.176	29.17
6	32621586	chr6:32621586:A:G	-	Intergenic	rs73728611	A>G	9	1.67x10-08	0.61 [0.52-0.72]	0.778	0
6	32621591	chr6:32621591:C:A	-	Intergenic	rs9271504	C>A	10	6.63x10-83	1.78 [1.69-1.88]	0.178	29.01
6	32621595	chr6:32621595:T:A	-	Intergenic	rs28664035	T>A	9	1.87x10-09	0.63 [0.54-0.73]	0.716	0
6	32621598	chr6:32621598:T:G	-	Intergenic	rs9271505	T>G	10	1.18x10-09	0.60 [0.51-0.71]	0	77.54
6	32621603	chr6:32621603:C:T	-	Intergenic	rs9271506	C>T	10	1.03x10-09	0.60 [0.51-0.71]	0	77.37
6	32621617	chr6:32621617:C:A	-	Intergenic	rs9271507	C>A	10	1.03x10-09	0.60 [0.51-0.71]	0	77.37
6	32621633	chr6:32621633:C:T	-	Intergenic	rs9271508	C>T	10	6.74x10-09	0.84 [0.79-0.89]	0.188	27.79
6	32621638	chr6:32621638:G:A	-	Intergenic	rs9271509	G>A	10	2.19x10-82	1.96 [1.84-2.09]	0.826	0
6	32621690	chr6:32621690:T:C	-	Intergenic	rs9271510	T>C	10	1.16x10-09	0.60 [0.51-0.71]	0	77.65
6	32621706	chr6:32621706:G:A	-	Intergenic	rs9271511	G>A	10	6.63x10-83	1.78 [1.69-1.88]	0.178	29.01
6	32621718	chr6:32621718:G:A	-	Intergenic	rs35990832	G>A	8	2.12x10-14	0.45 [0.37-0.55]	0.869	0
6	32621733	chr6:32621733:A:G	-	Intergenic	rs9271512	A>G	10	1.02x10-09	0.60 [0.51-0.71]	0	77.38
6	32621746	chr6:32621746:C:G	-	Intergenic	rs9271513	C>G	10	4.48x10-18	0.69 [0.64-0.75]	0.183	28.41
6	32621836	chr6:32621836:A:G	-	Intergenic	rs9271514	A>G	10	1.03x10-09	0.60 [0.51-0.71]	0	77.37
6	32621868	chr6:32621868:A:G	-	Intergenic	rs9271515	A>G	10	1.03x10-09	0.60 [0.51-0.71]	0	77.37
6	32621891	chr6:32621891:C:A	-	Intergenic	rs9271516	C>A	10	6.26x10-83	1.78 [1.69-1.88]	0.175	29.29
6	32621923	chr6:32621923:C:T	-	Intergenic	rs77875016	C>T	8	2.12x10-14	0.45 [0.37-0.55]	0.869	0
6	32621963	chr6:32621963:A:G	-	Intergenic	rs9271517	A>G	10	6.63x10-83	1.78 [1.69-1.88]	0.178	29.01
6	32621968	chr6:32621968:C:T	-	Intergenic	rs9271518	C>T	10	1.03x10-09	0.60 [0.51-0.71]	0	77.37
6	32621974	chr6:32621974:T:C	-	Intergenic	rs9271519	T>C	10	6.63x10-83	1.78 [1.69-1.88]	0.178	29.01
6	32621994	chr6:32621994:A:G	-	Intergenic	rs9271520	A>G	10	6.63x10-83	1.78 [1.69-1.88]	0.178	29.01
6	32622006	chr6:32622006:C:T	-	Intergenic	rs9271521	C>T	10	6.63x10-83	1.78 [1.69-1.88]	0.178	29.01
6	32622014	chr6:32622014:T:C	-	Intergenic	rs9271522	T>C	10	6.50x10-09	0.84 [0.79-0.89]	0.188	27.8
6	32622029	chr6:32622029:A:G	-	Intergenic	rs9271523	A>G	10	6.63x10-83	1.78 [1.69-1.88]	0.178	29.01
6	32622065	chr6:32622065:G:A	-	Intergenic	rs9271525	G>A	10	1.73x10-15	0.69 [0.63-0.76]	0.164	30.55
6	32622090	chr6:32622090:C:T	-	Intergenic	rs9271527	C>T	10	5.75x10-09	0.84 [0.79-0.89]	0.192	27.44
6	32622160	chr6:32622160:G:A	-	Intergenic	rs9271529	G>A	10	4.55x10-18	0.69 [0.64-0.75]	0.184	28.32
6	32622161	chr6:32622161:A:G	-	Intergenic	rs9271530	A>G	10	1.04x10-09	0.60 [0.51-0.71]	0	77.2
6	32622162	chr6:32622162:A:C	-	Intergenic	rs9271531	A>C	10	1.04x10-09	0.60 [0.51-0.71]	0	77.2
6	32622168	chr6:32622168:T:C	-	Intergenic	rs9271532	T>C	10	5.91x10-83	1.78 [1.69-1.88]	0.18	28.81

6	32622175	chr6:32622175:G:T	-	Intergenic	rs9271533	G>T	10	6.50x10-09	0.84 [0.79-0.89]	0.188	27.8
6	32622176	chr6:32622176:C:A	-	Intergenic	rs9271534	C>A	10	9.92x10-10	0.60 [0.51-0.71]	0	77.35
6	32622182	chr6:32622182:C:T	-	Intergenic	rs9271535	C>T	10	6.50x10-09	0.84 [0.79-0.89]	0.188	27.8
6	32622201	chr6:32622201:A:T	-	Intergenic	rs9271536	A>T	10	4.48x10-18	0.69 [0.64-0.75]	0.183	28.41
6	32622205	chr6:32622205:C:T	-	Intergenic	rs9271537	C>T	10	2.59x10-26	0.73 [0.69-0.77]	0.687	0
6	32622251	chr6:32622251:G:A	-	Intergenic	rs9271539	G>A	10	4.97x10-09	0.84 [0.79-0.89]	0.186	28.08
6	32622252	chr6:32622252:T:C	-	Intergenic	rs9271540	T>C	10	6.63x10-83	1.78 [1.69-1.88]	0.178	29.01
6	32622314	chr6:32622314:T:G	-	Intergenic	rs9271542	T>G	10	6.46x10-83	1.78 [1.69-1.88]	0.177	29.08
6	32622320	chr6:32622320:A:T	-	Intergenic	rs9271543	A>T	10	1.03x10-09	0.60 [0.51-0.71]	0	77.37
6	32622343	chr6:32622343:C:A	-	Intergenic	rs9271544	C>A	10	6.63x10-83	1.78 [1.69-1.88]	0.178	29.01
6	32622377	chr6:32622377:T:C	-	Intergenic	rs9271546	T>C	10	3.51x10-21	0.76 [0.72-0.80]	0.101	38.52
6	32622382	chr6:32622382:C:A	-	Intergenic	rs9271547	C>A	10	4.33x10-21	0.76 [0.72-0.80]	0.102	38.49
6	32622457	chr6:32622457:A:T	-	Intergenic	rs9271548	A>T	10	1.03x10-09	0.60 [0.51-0.71]	0	77.37
6	32622471	chr6:32622471:T:C	-	Intergenic	rs9271549	T>C	10	6.63x10-83	1.78 [1.69-1.88]	0.178	29.01
6	32622521	chr6:32622521:A:G	-	Intergenic	rs9271551	A>G	10	1.03x10-09	0.60 [0.51-0.71]	0	77.37
6	32622522	chr6:32622522:T:C	-	Intergenic	rs9271553	T>C	10	1.18x10-09	0.60 [0.51-0.71]	0	77.46
6	32622538	chr6:32622538:C:T	-	Intergenic	rs71542421	C>T	8	2.12x10-14	0.45 [0.37-0.55]	0.869	0
6	32622542	chr6:32622542:T:G	-	Intergenic	rs9271555	T>G	10	1.03x10-09	0.60 [0.51-0.71]	0	77.38
6	32622547	chr6:32622547:T:G	-	Intergenic	rs9271556	T>G	10	1.03x10-09	0.60 [0.51-0.71]	0	77.38
6	32622554	chr6:32622554:T:C	-	Intergenic	rs9271557	T>C	10	1.03x10-09	0.60 [0.51-0.71]	0	77.38
6	32622571	chr6:32622571:A:G	-	Intergenic	rs9271558	A>G	10	6.50x10-09	0.84 [0.79-0.89]	0.188	27.8
6	32622651	chr6:32622651:T:C	-	Intergenic	rs9271563	T>C	10	6.30x10-83	1.78 [1.69-1.88]	0.176	29.16
6	32622666	chr6:32622666:A:G	-	Intergenic	rs9271566	A>G	10	5.44x10-14	1.52 [1.37-1.69]	0.007	60.12
6	32622676	chr6:32622676:C:G	-	Intergenic	rs113705304	C>G	10	9.60x10-12	0.70 [0.63-0.77]	0.931	0
6	32622679	chr6:32622679:C:T	-	Intergenic	rs9271567	C>T	10	5.44x10-14	1.52 [1.37-1.69]	0.007	60.12
6	32622686	chr6:32622686:G:A	-	Intergenic	rs9271568	G>A	10	6.63x10-83	1.78 [1.69-1.88]	0.178	29.01
6	32622696	chr6:32622696:T:C	-	Intergenic	rs2105899	T>C	10	4.48x10-18	0.69 [0.64-0.75]	0.183	28.41
6	32622706	chr6:32622706:C:T	-	Intergenic	rs9271571	C>T	10	5.44x10-83	1.78 [1.69-1.88]	0.184	28.34
6	32622721	chr6:32622721:T:G	-	Intergenic	rs2105898	T>G	10	4.48x10-18	0.69 [0.64-0.75]	0.183	28.41
6	32622724	chr6:32622724:A:C	-	Intergenic	rs9271573	A>C	10	8.81x10-16	0.79 [0.74-0.83]	0.16	31.09
6	32622752	chr6:32622752:A:G	-	Intergenic	rs35407883	A>G	8	2.12x10-14	0.45 [0.37-0.55]	0.869	0
6	32622813	chr6:32622813:T:G	-	Intergenic	rs9271574	T>G	10	6.63x10-83	1.78 [1.69-1.88]	0.178	29.01
6	32622825	chr6:32622825:T:C	-	Intergenic	rs570194309	T>C	10	6.63x10-83	1.78 [1.69-1.88]	0.178	29.01
6	32622835	chr6:32622835:T:C	-	Intergenic	rs9271576	T>C	10	6.63x10-83	1.78 [1.69-1.88]	0.178	29.01
6	32622847	chr6:32622847:T:C	-	Intergenic	rs2395225	T>C	10	6.26x10-09	0.84 [0.79-0.89]	0.185	28.24
6	32622858	chr6:32622858:A:T	-	Intergenic	rs9271578	A>T	10	1.28x10-32	1.41 [1.34-1.49]	0.191	27.5
6	32622860	chr6:32622860:T:A	-	Intergenic	rs9271579	T>A	10	2.19x10-82	1.96 [1.84-2.09]	0.826	0
6	32622864	chr6:32622864:T:G	-	Intergenic	rs9271580	T>G	10	2.19x10-82	1.96 [1.84-2.09]	0.826	0
6	32622991	chr6:32622991:C:A	-	Intergenic	rs34831921	C>A	10	9.60x10-12	0.70 [0.63-0.77]	0.931	0
6	32622997	chr6:32622997:T:C	-	Intergenic	rs9271582	T>C	10	6.63x10-83	1.78 [1.69-1.88]	0.178	29.01
6	32623003	chr6:32623003:T:C	-	Intergenic	rs9271583	T>C	10	6.63x10-83	1.78 [1.69-1.88]	0.178	29.01
6	32623093	chr6:32623093:T:C	-	Intergenic	rs9271584	T>C	10	6.63x10-83	1.78 [1.69-1.88]	0.178	29.01
6	32623102	chr6:32623102:C:A	-	Intergenic	rs9271585	C>A	10	6.63x10-83	1.78 [1.69-1.88]	0.178	29.01
6	32623122	chr6:32623122:G:T	-	Intergenic	rs9271586	G>T	10	1.28x10-32	1.41 [1.34-1.49]	0.191	27.5
6	32623139	chr6:32623139:A:G	-	Intergenic	rs9271587	A>G	10	1.28x10-32	1.41 [1.34-1.49]	0.191	27.5
6	32623176	chr6:32623176:T:C	-	Intergenic	rs9271588	T>C	10	1.28x10-32	1.41 [1.34-1.49]	0.191	27.5

6	32623224	chr6:32623224:A:G	HLA-DQA1	Intergenic	rs34136716	A>G	9	1.67x10-08	0.61 [0.52-0.72]	0.778	0
6	32623351	chr6:32623351:G:A	HLA-DQA1	Intergenic	rs9271589	G>A	10	9.65x10-82	1.77 [1.68-1.88]	0.145	32.82
6	32623361	chr6:32623361:G:A	HLA-DQA1	Intergenic	rs9271590	G>A	10	1.28x10-32	1.41 [1.34-1.49]	0.191	27.5
6	32623364	chr6:32623364:G:A	HLA-DQA1	Intergenic	rs9271591	G>A	10	1.28x10-32	1.41 [1.34-1.49]	0.191	27.5
6	32623374	chr6:32623374:C:T	HLA-DQA1	Intergenic	rs35765065	C>T	8	2.12x10-14	0.45 [0.37-0.55]	0.869	0
6	32623396	chr6:32623396:C:T	HLA-DQA1	Intergenic	rs9271592	C>T	10	6.91x10-83	1.78 [1.69-1.88]	0.178	29
6	32623421	chr6:32623421:C:T	HLA-DQA1	Intergenic	rs9271593	C>T	10	7.77x10-16	0.79 [0.74-0.83]	0.164	30.61
6	32623436	chr6:32623436:A:G	HLA-DQA1	Intergenic	rs9271594	A>G	10	2.73x10-86	2.02 [1.89-2.16]	0.938	0
6	32623445	chr6:32623445:A:G	HLA-DQA1	Intergenic	rs34898108	A>G	9	1.67x10-08	0.61 [0.52-0.72]	0.778	0
6	32623449	chr6:32623449:A:G	HLA-DQA1	Intergenic	rs35407265	A>G	10	9.81x10-12	0.70 [0.64-0.77]	0.931	0
6	32623455	chr6:32623455:C:T	HLA-DQA1	Intergenic	rs35316732	C>T	10	9.81x10-12	0.70 [0.64-0.77]	0.931	0
6	32623458	chr6:32623458:A:G	HLA-DQA1	Intergenic	rs9271595	A>G	10	8.63x10-83	1.78 [1.69-1.88]	0.181	28.61
6	32623460	chr6:32623460:A:G	HLA-DQA1	Intergenic	rs9271596	A>G	10	8.63x10-83	1.78 [1.69-1.88]	0.181	28.61
6	32623461	chr6:32623461:A:G	HLA-DQA1	Intergenic	rs34376179	A>G	10	9.81x10-12	0.70 [0.64-0.77]	0.931	0
6	32623471	chr6:32623471:C:T	HLA-DQA1	Intergenic	rs34967069	C>T	10	9.81x10-12	0.70 [0.64-0.77]	0.931	0
6	32623503	chr6:32623503:T:C	HLA-DQA1	Intergenic	rs35326082	T>C	9	1.67x10-08	0.61 [0.52-0.72]	0.778	0
6	32623514	chr6:32623514:T:A	HLA-DQA1	Intergenic	rs9271597	T>A	10	5.13x10-14	1.52 [1.37-1.69]	0.007	60.01
6	32623523	chr6:32623523:G:A	HLA-DQA1	Intergenic	rs3129764	G>A	10	9.43x10-18	0.69 [0.64-0.75]	0.202	26.23
6	32623538	chr6:32623538:A:C	HLA-DQA1	Intergenic	rs9271599	A>C	10	8.81x10-16	0.79 [0.74-0.83]	0.16	31.09
6	32623555	chr6:32623555:T:G	HLA-DQA1	Intergenic	rs9271600	T>G	10	4.99x10-14	1.52 [1.37-1.69]	0.007	60.01
6	32623560	chr6:32623560:A:T	HLA-DQA1	Intergenic	rs9271601	A>T	10	5.13x10-14	1.52 [1.37-1.69]	0.007	60.01
6	32623583	chr6:32623583:G:C	HLA-DQA1	Intergenic	rs114480004	G>C	8	2.12x10-14	0.45 [0.37-0.55]	0.869	0
6	32623606	chr6:32623606:T:G	HLA-DQA1	Intergenic	rs9271602	T>G	10	7.39x10-23	1.68 [1.52-1.86]	0.019	54.65
6	32623645	chr6:32623645:A:G	HLA-DQA1	Intergenic	rs34915133	A>G	10	9.63x10-12	0.70 [0.64-0.77]	0.926	0
6	32623651	chr6:32623651:A:G	HLA-DQA1	Intergenic	rs3129765	A>G	10	1.25x10-17	0.69 [0.64-0.75]	0.193	27.29
6	32623653	chr6:32623653:A:G	HLA-DQA1	Intergenic	rs9271604	A>G	10	7.68x10-83	1.78 [1.69-1.88]	0.175	29.28
6	32623671	chr6:32623671:G:A	HLA-DQA1	Intergenic	rs9271605	G>A	10	6.91x10-83	1.78 [1.69-1.88]	0.178	29
6	32623789	chr6:32623789:A:G	HLA-DQA1	Intergenic	rs9271607	A>G	10	6.91x10-83	1.78 [1.69-1.88]	0.178	29
6	32623790	chr6:32623790:T:A	HLA-DQA1	Intergenic	rs73728618	T>A	10	9.60x10-12	0.70 [0.63-0.77]	0.931	0
6	32623811	chr6:32623811:A:G	HLA-DQA1	Intergenic	rs9271608	A>G	10	1.47x10-86	2.02 [1.89-2.16]	0.942	0
6	32623820	chr6:32623820:T:C	HLA-DQA1	Intergenic	rs9271609	T>C	10	9.47x10-83	1.78 [1.69-1.88]	0.176	29.16
6	32623824	chr6:32623824:C:T	HLA-DQA1	Intergenic	rs9271610	C>T	10	2.87x10-16	0.78 [0.74-0.83]	0.365	8.4
6	32623832	chr6:32623832:A:G	HLA-DQA1	Intergenic	rs9271611	A>G	10	2.87x10-16	0.78 [0.74-0.83]	0.365	8.4
6	32623852	chr6:32623852:C:T	HLA-DQA1	Intergenic	rs73728619	C>T	9	1.67x10-08	0.61 [0.52-0.72]	0.778	0
6	32623887	chr6:32623887:G:A	HLA-DQA1	Intergenic	rs35001273	G>A	10	9.60x10-12	0.70 [0.63-0.77]	0.931	0
6	32623909	chr6:32623909:A:G	HLA-DQA1	Intergenic	rs9271615	A>G	10	2.01x10-33	1.42 [1.34-1.50]	0.865	0
6	32623913	chr6:32623913:C:T	HLA-DQA1	Intergenic	rs4959107	C>T	10	3.17x10-66	1.66 [1.57-1.75]	0.102	38.42
6	32623974	chr6:32623974:A:G	HLA-DQA1	Intergenic	rs4959030	A>G	8	4.50x10-14	0.65 [0.58-0.73]	0.936	0
6	32623975	chr6:32623975:G:A	HLA-DQA1	Intergenic	rs9271618	G>A	10	5.50x10-09	0.84 [0.79-0.89]	0.172	29.67
6	32624000	chr6:32624000:G:T	HLA-DQA1	Intergenic	rs9271620	G>T	10	1.28x10-32	1.41 [1.34-1.49]	0.191	27.5
6	32624068	chr6:32624068:G:A	HLA-DQA1	Intergenic	rs9271622	G>A	10	6.95x10-18	0.69 [0.64-0.75]	0.215	24.84
6	32624137	chr6:32624137:G:C	HLA-DQA1	Intergenic	rs9271623	G>C	10	5.50x10-09	0.84 [0.79-0.89]	0.172	29.67
6	32624144	chr6:32624144:G:T	HLA-DQA1	Intergenic	rs9271624	G>T	10	6.91x10-83	1.78 [1.69-1.88]	0.178	29
6	32624145	chr6:32624145:G:A	HLA-DQA1	Intergenic	rs4959108	G>A	10	7.56x10-13	0.71 [0.64-0.77]	0.277	18.07
6	32624194	chr6:32624194:T:G	HLA-DQA1	Intergenic	rs9271628	T>G	10	6.91x10-83	1.78 [1.69-1.88]	0.178	29
6	32624199	chr6:32624199:C:A	HLA-DQA1	Intergenic	rs9271629	C>A	10	6.95x10-18	0.69 [0.64-0.75]	0.215	24.84

6	32624212	chr6:32624212:T:G	HLA-DQA1	Intergenic	rs9271630	T>G	10	5.65x10-09	0.84 [0.79-0.89]	0.174	29.43
6	32624219	chr6:32624219:A:G	HLA-DQA1	Intergenic	rs9271631	A>G	10	6.95x10-18	0.69 [0.64-0.75]	0.215	24.84
6	32624232	chr6:32624232:A:G	HLA-DQA1	Intergenic	rs1281947	A>G	10	3.44x10-85	2.02 [1.89-2.16]	0.921	0
6	32624291	chr6:32624291:T:C	HLA-DQA1	Intergenic	rs35867827	T>C	10	9.60x10-12	0.70 [0.63-0.77]	0.931	0
6	32624335	chr6:32624335:A:G	HLA-DQA1	Intergenic	rs9271634	A>G	10	6.95x10-18	0.69 [0.64-0.75]	0.215	24.84
6	32624339	chr6:32624339:C:G	HLA-DQA1	Intergenic	rs9271635	C>G	10	5.13x10-18	0.69 [0.64-0.75]	0.221	24.19
6	32624347	chr6:32624347:A:G	HLA-DQA1	Intergenic	rs9271637	A>G	10	6.91x10-83	1.78 [1.69-1.88]	0.178	29
6	32624365	chr6:32624365:C:T	HLA-DQA1	Intergenic	rs9271638	C>T	10	8.21x10-10	0.60 [0.51-0.71]	0	77.01
6	32624366	chr6:32624366:A:G	HLA-DQA1	Intergenic	rs9271639	A>G	10	5.13x10-18	0.69 [0.64-0.75]	0.221	24.19
6	32624423	chr6:32624423:T:C	HLA-DQA1	Intergenic	rs9271640	T>C	10	7.88x10-18	0.69 [0.64-0.75]	0.209	25.51
6	32624432	chr6:32624432:A:G	HLA-DQA1	Intergenic	rs9271641	A>G	10	7.88x10-18	0.69 [0.64-0.75]	0.209	25.51
6	32624442	chr6:32624442:A:G	HLA-DQA1	Intergenic	rs9271642	A>G	10	9.45x10-16	0.79 [0.74-0.83]	0.159	31.18
6	32624452	chr6:32624452:G:A	HLA-DQA1	Intergenic	rs9271643	G>A	10	8.33x10-83	1.78 [1.69-1.88]	0.173	29.54
6	32624536	chr6:32624536:T:C	HLA-DQA1	Intergenic	rs35224566	T>C	10	9.60x10-12	0.70 [0.63-0.77]	0.931	0
6	32624560	chr6:32624560:G:A	HLA-DQA1	Intergenic	rs9271644	G>A	10	8.33x10-83	1.78 [1.69-1.88]	0.173	29.54
6	32624568	chr6:32624568:C:G	HLA-DQA1	Intergenic	rs9271645	C>G	10	7.88x10-18	0.69 [0.64-0.75]	0.209	25.51
6	32624583	chr6:32624583:C:T	HLA-DQA1	Intergenic	rs4246055	C>T	8	2.73x10-14	0.57 [0.50-0.66]	0.29	17.73
6	32624790	chr6:32624790:G:A	HLA-DQA1	Intergenic	rs9271655	G>A	10	7.63x10-18	0.69 [0.64-0.75]	0.207	25.69
6	32624811	chr6:32624811:T:C	HLA-DQA1	Intergenic	rs9271658	T>C	10	7.85x10-18	0.69 [0.64-0.75]	0.209	25.54
6	32624825	chr6:32624825:T:G	HLA-DQA1	Intergenic	rs9271660	T>G	10	5.14x10-83	1.78 [1.69-1.88]	0.183	28.44
6	32624828	chr6:32624828:T:A	HLA-DQA1	Intergenic	rs9271661	T>A	10	5.14x10-83	1.78 [1.69-1.88]	0.183	28.44
6	32624851	chr6:32624851:G:A	HLA-DQA1	Intergenic	rs9271662	G>A	10	4.76x10-09	0.84 [0.79-0.89]	0.186	28.02
6	32624859	chr6:32624859:G:A	HLA-DQA1	Intergenic	rs9271663	G>A	10	1.34x10-33	1.42 [1.34-1.50]	0.871	0
6	32624879	chr6:32624879:T:C	HLA-DQA1	Intergenic	rs9271664	T>C	10	7.88x10-18	0.69 [0.64-0.75]	0.209	25.51
6	32624899	chr6:32624899:G:A	HLA-DQA1	Intergenic	rs9271665	G>A	10	1.31x10-09	0.60 [0.51-0.71]	0	77.56
6	32624933	chr6:32624933:T:C	HLA-DQA1	Intergenic	rs9271667	T>C	10	5.80x10-09	0.84 [0.79-0.89]	0.167	30.26
6	32625013	chr6:32625013:G:A	HLA-DQA1	Intergenic	rs114683059	G>A	9	1.62x10-08	0.61 [0.52-0.72]	0.78	0
6	32625056	chr6:32625056:C:G	HLA-DQA1	Intergenic	rs9271673	C>G	10	7.88x10-18	0.69 [0.64-0.75]	0.209	25.51
6	32625068	chr6:32625068:T:C	HLA-DQA1	Intergenic	rs9271674	T>C	10	8.33x10-83	1.78 [1.69-1.88]	0.173	29.54
6	32625173	chr6:32625173:C:T	HLA-DQA1	Intergenic	rs9271683	C>T	10	7.88x10-18	0.69 [0.64-0.75]	0.209	25.51
6	32625176	chr6:32625176:C:T	HLA-DQA1	Intergenic	rs9271684	C>T	10	5.50x10-09	0.84 [0.79-0.89]	0.172	29.67
6	32625196	chr6:32625196:T:C	HLA-DQA1	Intergenic	rs9271686	T>C	10	7.88x10-18	0.69 [0.64-0.75]	0.209	25.51
6	32625208	chr6:32625208:A:G	HLA-DQA1	Intergenic	rs9271687	A>G	10	7.43x10-13	0.71 [0.64-0.77]	0.275	18.27
6	32625215	chr6:32625215:A:G	HLA-DQA1	Intergenic	rs9271688	A>G	10	5.50x10-09	0.84 [0.79-0.89]	0.172	29.67
6	32625222	chr6:32625222:C:G	HLA-DQA1	Intergenic	rs9271689	C>G	10	7.88x10-18	0.69 [0.64-0.75]	0.209	25.51
6	32625229	chr6:32625229:A:G	HLA-DQA1	Intergenic	rs9271691	A>G	10	1.79x10-33	1.42 [1.34-1.50]	0.863	0
6	32625233	chr6:32625233:C:A	HLA-DQA1	Intergenic	rs9271692	C>A	10	1.79x10-33	1.42 [1.34-1.50]	0.863	0
6	32625256	chr6:32625256:C:T	HLA-DQA1	Intergenic	rs9271693	C>T	10	8.33x10-83	1.78 [1.69-1.88]	0.173	29.54
6	32625274	chr6:32625274:T:C	HLA-DQA1	Intergenic	rs9271694	T>C	10	5.50x10-09	0.84 [0.79-0.89]	0.172	29.67
6	32625303	chr6:32625303:A:G	HLA-DQA1	Intergenic	rs9271695	A>G	10	7.88x10-18	0.69 [0.64-0.75]	0.209	25.51
6	32625304	chr6:32625304:G:C	HLA-DQA1	Intergenic	rs796340315	G>C	8	2.12x10-14	0.45 [0.37-0.55]	0.869	0
6	32625308	chr6:32625308:T:C	HLA-DQA1	Intergenic	rs9271696	T>C	10	2.87x10-16	0.78 [0.74-0.83]	0.365	8.4
6	32625318	chr6:32625318:G:A	HLA-DQA1	Intergenic	rs9271697	G>A	10	9.86x10-18	0.69 [0.64-0.75]	0.195	27.06
6	32625367	chr6:32625367:A:G	HLA-DQA1	Intergenic	rs35508382	A>G	10	9.60x10-12	0.70 [0.63-0.77]	0.931	0
6	32625390	chr6:32625390:C:T	HLA-DQA1	Intergenic	rs4587163	C>T	10	5.40x10-21	1.61 [1.47-1.78]	0.033	50.64
6	32625402	chr6:32625402:A:G	HLA-DQA1	Intergenic	rs9271699	A>G	10	1.79x10-33	1.42 [1.34-1.50]	0.863	0

6	32625421	chr6:32625421:G:C	HLA-DQA1	Intergenic	rs9271700	G>C	10	7.88x10-18	0.69 [0.64-0.75]	0.209	25.51
6	32625423	chr6:32625423:A:G	HLA-DQA1	Intergenic	rs199507083	A>G	9	1.67x10-08	0.61 [0.52-0.72]	0.778	0
6	32625443	chr6:32625443:T:G	HLA-DQA1	Intergenic	rs9271702	T>G	10	8.66x10-18	0.69 [0.64-0.75]	0.213	24.99
6	32625493	chr6:32625493:G:C	HLA-DQA1	Intergenic	rs34720986	G>C	10	9.60x10-12	0.70 [0.63-0.77]	0.931	0
6	32625503	chr6:32625503:C:T	HLA-DQA1	Intergenic	rs9271703	C>T	10	5.50x10-09	0.84 [0.79-0.89]	0.172	29.67
6	32625539	chr6:32625539:C:A	HLA-DQA1	Intergenic	rs560607175	C>A	10	9.60x10-12	0.70 [0.63-0.77]	0.931	0
6	32625540	chr6:32625540:T:G	HLA-DQA1	Intergenic	rs9271704	T>G	10	5.50x10-09	0.84 [0.79-0.89]	0.172	29.67
6	32625544	chr6:32625544:G:T	HLA-DQA1	Intergenic	rs9271705	G>T	10	5.50x10-09	0.84 [0.79-0.89]	0.172	29.67
6	32625579	chr6:32625579:C:T	HLA-DQA1	Intergenic	rs9271707	C>T	10	1.51x10-37	1.45 [1.37-1.53]	0.725	0
6	32625609	chr6:32625609:C:A	HLA-DQA1	Intergenic	rs9271708	C>A	10	5.50x10-09	0.84 [0.79-0.89]	0.172	29.67
6	32625615	chr6:32625615:G:A	HLA-DQA1	Intergenic	rs9271709	G>A	10	5.30x10-09	0.84 [0.79-0.89]	0.172	29.7
6	32625702	chr6:32625702:C:T	HLA-DQA1	Intergenic	rs9271719	C>T	10	7.88x10-18	0.69 [0.64-0.75]	0.209	25.51
6	32625742	chr6:32625742:G:A	HLA-DQA1	Intergenic	rs9271721	G>A	10	8.41x10-18	0.69 [0.64-0.75]	0.197	26.86
6	32625808	chr6:32625808:G:A	HLA-DQA1	Intergenic	rs9271727	G>A	10	5.50x10-09	0.84 [0.79-0.89]	0.172	29.67
6	32625815	chr6:32625815:T:C	HLA-DQA1	Intergenic	rs9271728	T>C	10	5.50x10-09	0.84 [0.79-0.89]	0.172	29.67
6	32625823	chr6:32625823:G:A	HLA-DQA1	Intergenic	rs9271729	G>A	10	5.50x10-09	0.84 [0.79-0.89]	0.172	29.67
6	32625831	chr6:32625831:G:A	HLA-DQA1	Intergenic	rs9271730	G>A	10	1.06x10-09	0.60 [0.51-0.71]	0	77.29
6	32625835	chr6:32625835:A:G	HLA-DQA1	Intergenic	rs9271731	A>G	10	7.88x10-18	0.69 [0.64-0.75]	0.209	25.51
6	32625850	chr6:32625850:G:A	HLA-DQA1	Intergenic	rs9271732	G>A	10	3.24x10-28	0.65 [0.60-0.70]	0.267	19.12
6	32625856	chr6:32625856:G:A	HLA-DQA1	Intergenic	rs9271733	G>A	10	5.50x10-09	0.84 [0.79-0.89]	0.172	29.67
6	32625872	chr6:32625872:G:A	HLA-DQA1	Intergenic	rs9271735	G>A	10	9.82x10-83	1.78 [1.68-1.88]	0.174	29.39
6	32626284	chr6:32626284:A:G	HLA-DQA1	Intergenic	rs9271763	A>G	10	1.39x10-32	1.41 [1.34-1.49]	0.191	27.48
6	32626296	chr6:32626296:G:T	HLA-DQA1	Intergenic	rs72848204	G>T	10	5.40x10-09	0.84 [0.79-0.89]	0.172	29.69
6	32626326	chr6:32626326:G:A	HLA-DQA1	Intergenic	rs34518860	G>A	10	9.60x10-12	0.70 [0.63-0.77]	0.931	0
6	32626382	chr6:32626382:G:A	HLA-DQA1	Intergenic	rs9271764	G>A	10	8.33x10-83	1.78 [1.69-1.88]	0.173	29.54
6	32626389	chr6:32626389:A:G	HLA-DQA1	Intergenic	rs9271765	A>G	10	8.33x10-83	1.78 [1.69-1.88]	0.173	29.54
6	32626392	chr6:32626392:A:G	HLA-DQA1	Intergenic	rs9271766	A>G	10	7.88x10-18	0.69 [0.64-0.75]	0.209	25.51
6	32626399	chr6:32626399:G:T	HLA-DQA1	Intergenic	rs9271767	G>T	10	8.33x10-83	1.78 [1.69-1.88]	0.173	29.54
6	32626422	chr6:32626422:A:G	HLA-DQA1	Intergenic	rs35130701	A>G	10	5.50x10-09	0.84 [0.79-0.89]	0.172	29.67
6	32626440	chr6:32626440:G:T	HLA-DQA1	Intergenic	rs9271769	G>T	10	8.33x10-83	1.78 [1.69-1.88]	0.173	29.54
6	32626471	chr6:32626471:G:A	HLA-DQA1	Intergenic	rs9271770	G>A	10	7.88x10-18	0.69 [0.64-0.75]	0.209	25.51
6	32626497	chr6:32626497:T:C	HLA-DQA1	Intergenic	rs9271771	T>C	10	7.88x10-18	0.69 [0.64-0.75]	0.209	25.51
6	32626498	chr6:32626498:G:A	HLA-DQA1	Intergenic	rs9271772	G>A	10	7.88x10-18	0.69 [0.64-0.75]	0.209	25.51
6	32626518	chr6:32626518:G:T	HLA-DQA1	Intergenic	rs75237145	G>T	10	1.79x10-33	1.42 [1.34-1.50]	0.863	0
6	32626522	chr6:32626522:T:A	HLA-DQA1	Intergenic	rs114969562	T>A	10	5.50x10-09	0.84 [0.79-0.89]	0.172	29.67
6	32626527	chr6:32626527:T:C	HLA-DQA1	Intergenic	rs9271773	T>C	10	8.33x10-83	1.78 [1.69-1.88]	0.173	29.54
6	32626532	chr6:32626532:A:C	HLA-DQA1	Intergenic	rs9271774	A>C	10	7.88x10-18	0.69 [0.64-0.75]	0.209	25.51
6	32626551	chr6:32626551:C:T	HLA-DQA1	Intergenic	rs9271775	C>T	10	1.73x10-15	0.70 [0.65-0.76]	0.183	28.36
6	32626564	chr6:32626564:T:C	HLA-DQA1	Intergenic	rs9271776	T>C	10	8.33x10-83	1.78 [1.69-1.88]	0.173	29.54
6	32626577	chr6:32626577:T:C	HLA-DQA1	Intergenic	rs35366682	T>C	10	5.50x10-09	0.84 [0.79-0.89]	0.172	29.67
6	32626611	chr6:32626611:C:T	HLA-DQA1	Intergenic	rs568805507	C>T	10	9.98x10-12	0.70 [0.64-0.77]	0.931	0
6	32626618	chr6:32626618:T:G	HLA-DQA1	Intergenic	rs116093550	T>G	10	2.09x10-33	1.42 [1.34-1.50]	0.859	0
6	32626619	chr6:32626619:G:T	HLA-DQA1	Intergenic	rs116361489	G>T	10	1.79x10-33	1.42 [1.34-1.50]	0.863	0
6	32626622	chr6:32626622:A:G	HLA-DQA1	Intergenic	rs539509361	A>G	10	5.21x10-09	0.84 [0.79-0.89]	0.199	26.64
6	32626626	chr6:32626626:C:T	HLA-DQA1	Intergenic	rs3104393	C>T	10	8.33x10-83	1.78 [1.69-1.88]	0.173	29.54
6	32626648	chr6:32626648:C:T	HLA-DQA1	Intergenic	rs3104392	C>T	10	7.74x10-13	0.71 [0.64-0.78]	0.276	18.12

6	32626664	chr6:32626664:A:G	HLA-DQA1	Intergenic	rs3129766	A>G	10	3.24x10-75	1.73 [1.63-1.82]	0.1	38.75
6	32626770	chr6:32626770:G:A	HLA-DQA1	Intergenic	rs9271781	G>A	10	2.07x10-33	1.42 [1.34-1.50]	0.854	0
6	32626785	chr6:32626785:G:A	HLA-DQA1	Intergenic	rs9271782	G>A	10	2.36x10-33	1.42 [1.34-1.50]	0.867	0
6	32626835	chr6:32626835:G:A	HLA-DQA1	Intergenic	rs116571774	G>A	9	1.67x10-08	0.61 [0.52-0.72]	0.778	0
6	32626867	chr6:32626867:T:C	HLA-DQA1	Intergenic	rs35456124	T>C	10	1.49x10-33	1.42 [1.34-1.50]	0.857	0
6	32626881	chr6:32626881:T:A	HLA-DQA1	Intergenic	rs551560868	T>A	9	1.71x10-08	0.61 [0.52-0.72]	0.778	0
6	32626884	chr6:32626884:A:G	HLA-DQA1	Intergenic	rs116352702	A>G	10	9.89x10-12	0.70 [0.64-0.77]	0.931	0
6	32626927	chr6:32626927:T:A	HLA-DQA1	Intergenic	rs9271784	T>A	10	1.79x10-33	1.42 [1.34-1.50]	0.863	0
6	32626931	chr6:32626931:G:T	HLA-DQA1	Intergenic	rs9271785	G>T	10	1.81x10-33	1.42 [1.34-1.50]	0.863	0
6	32626932	chr6:32626932:A:T	HLA-DQA1	Intergenic	rs9271786	A>T	10	1.81x10-33	1.42 [1.34-1.50]	0.863	0
6	32626941	chr6:32626941:T:C	HLA-DQA1	Intergenic	rs9271787	T>C	10	1.81x10-33	1.42 [1.34-1.50]	0.863	0
6	32626957	chr6:32626957:A:G	HLA-DQA1	Intergenic	rs9271788	A>G	10	1.79x10-33	1.42 [1.34-1.50]	0.863	0
6	32626961	chr6:32626961:A:T	HLA-DQA1	Intergenic	rs116747180	A>T	9	1.72x10-08	0.61 [0.52-0.72]	0.779	0
6	32626991	chr6:32626991:G:A	HLA-DQA1	Intergenic	rs9271789	G>A	10	1.79x10-33	1.42 [1.34-1.50]	0.863	0
6	32627009	chr6:32627009:C:T	HLA-DQA1	Intergenic	rs73728638	C>T	9	1.67x10-08	0.61 [0.52-0.72]	0.778	0
6	32627033	chr6:32627033:G:A	HLA-DQA1	Intergenic	rs9271791	G>A	10	1.79x10-33	1.42 [1.34-1.50]	0.863	0
6	32627042	chr6:32627042:G:T	HLA-DQA1	Intergenic	rs201407766	G>T	10	1.57x10-86	2.02 [1.89-2.16]	0.941	0
6	32627078	chr6:32627078:G:A	HLA-DQA1	Intergenic	rs9271794	G>A	10	1.79x10-33	1.42 [1.34-1.50]	0.863	0
6	32627121	chr6:32627121:C:T	HLA-DQA1	Intergenic	rs72848212	C>T	10	1.79x10-33	1.42 [1.34-1.50]	0.863	0
6	32627124	chr6:32627124:T:C	HLA-DQA1	Intergenic	rs3104391	T>C	10	7.78x10-13	0.71 [0.64-0.78]	0.277	18.02
6	32627138	chr6:32627138:A:G	HLA-DQA1	Intergenic	rs9271823	A>G	10	1.79x10-33	1.42 [1.34-1.50]	0.863	0
6	32627139	chr6:32627139:A:G	HLA-DQA1	Intergenic	rs9271824	A>G	10	1.79x10-33	1.42 [1.34-1.50]	0.863	0
6	32627176	chr6:32627176:C:A	HLA-DQA1	Intergenic	rs9271842	C>A	10	1.79x10-33	1.42 [1.34-1.50]	0.863	0
6	32627180	chr6:32627180:G:T	HLA-DQA1	Intergenic	rs9271843	G>T	10	1.68x10-33	1.42 [1.34-1.50]	0.859	0
6	32627203	chr6:32627203:T:A	HLA-DQA1	Intergenic	rs9271846	T>A	10	1.68x10-33	1.42 [1.34-1.50]	0.859	0
6	32627216	chr6:32627216:A:G	HLA-DQA1	Intergenic	rs9271847	A>G	10	1.68x10-33	1.42 [1.34-1.50]	0.859	0
6	32627221	chr6:32627221:C:T	HLA-DQA1	Intergenic	rs116518618	C>T	10	8.22x10-12	0.70 [0.63-0.77]	0.924	0
6	32627250	chr6:32627250:A:G	HLA-DQA1	Intergenic	rs9271848	A>G	10	1.68x10-33	1.42 [1.34-1.50]	0.859	0
6	32627254	chr6:32627254:A:T	HLA-DQA1	Intergenic	rs3104390	A>T	10	1.02x10-17	0.69 [0.64-0.75]	0.199	26.57
6	32627283	chr6:32627283:A:G	HLA-DQA1	Intergenic	rs9271850	A>G	10	4.87x10-09	0.84 [0.79-0.89]	0.165	30.48
6	32627306	chr6:32627306:G:T	HLA-DQA1	Intergenic	rs3129768	G>T	10	8.40x10-18	0.69 [0.64-0.75]	0.212	25.17
6	32627340	chr6:32627340:C:T	HLA-DQA1	Intergenic	rs9271853	C>T	10	8.33x10-83	1.78 [1.69-1.88]	0.173	29.54
6	32627341	chr6:32627341:G:A	HLA-DQA1	Intergenic	rs9271854	G>A	10	1.68x10-33	1.42 [1.34-1.50]	0.859	0
6	32627342	chr6:32627342:T:C	HLA-DQA1	Intergenic	rs9271855	T>C	10	1.68x10-33	1.42 [1.34-1.50]	0.859	0
6	32627343	chr6:32627343:G:A	HLA-DQA1	Intergenic	rs9271856	G>A	10	1.68x10-33	1.42 [1.34-1.50]	0.859	0
6	32627370	chr6:32627370:C:T	HLA-DQA1	Intergenic	rs9271857	C>T	10	1.68x10-33	1.42 [1.34-1.50]	0.859	0
6	32627416	chr6:32627416:T:G	HLA-DQA1	Intergenic	rs1334766346	T>G	9	1.67x10-08	0.61 [0.52-0.72]	0.778	0
6	32627417	chr6:32627417:C:T	HLA-DQA1	Intergenic	rs116603449	C>T	10	1.68x10-33	1.42 [1.34-1.50]	0.859	0
6	32627446	chr6:32627446:A:G	HLA-DQA1	Intergenic	rs9271858	A>G	10	1.68x10-33	1.42 [1.34-1.50]	0.859	0
6	32627454	chr6:32627454:T:G	HLA-DQA1	Intergenic	rs9271859	T>G	10	1.68x10-33	1.42 [1.34-1.50]	0.859	0
6	32627819	chr6:32627819:G:C	HLA-DQA1	Intergenic	rs9271880	G>C	10	5.06x10-09	0.84 [0.79-0.89]	0.165	30.46
6	32627827	chr6:32627827:G:A	HLA-DQA1	Intergenic	rs9271881	G>A	10	1.89x10-33	1.42 [1.34-1.50]	0.861	0
6	32627828	chr6:32627828:C:T	HLA-DQA1	Intergenic	rs9271882	C>T	10	1.68x10-33	1.42 [1.34-1.50]	0.859	0
6	32627833	chr6:32627833:G:A	HLA-DQA1	Intergenic	rs9271883	G>A	10	1.68x10-33	1.42 [1.34-1.50]	0.859	0
6	32627851	chr6:32627851:T:C	HLA-DQA1	Intergenic	rs9271884	T>C	10	1.68x10-33	1.42 [1.34-1.50]	0.859	0
6	32627852	chr6:32627852:C:T	HLA-DQA1	Intergenic	rs9271885	C>T	10	1.68x10-33	1.42 [1.34-1.50]	0.859	0

6	32627856	chr6:32627856:C:T	HLA-DQA1	Intergenic	rs9271886	C>T	10	1.68x10-33	1.42 [1.34-1.50]	0.859	0
6	32627902	chr6:32627902:G:A	HLA-DQA1	Intergenic	rs9271887	G>A	10	4.87x10-09	0.84 [0.79-0.89]	0.165	30.48
6	32627955	chr6:32627955:T:C	HLA-DQA1	Intergenic	rs9271888	T>C	10	1.68x10-33	1.42 [1.34-1.50]	0.859	0
6	32627970	chr6:32627970:G:A	HLA-DQA1	Intergenic	rs9271889	G>A	10	1.68x10-33	1.42 [1.34-1.50]	0.859	0
6	32628002	chr6:32628002:C:A	HLA-DQA1	Intergenic	rs9271890	C>A	10	1.68x10-33	1.42 [1.34-1.50]	0.859	0
6	32628006	chr6:32628006:G:T	HLA-DQA1	Intergenic	rs9271891	G>T	10	1.68x10-33	1.42 [1.34-1.50]	0.859	0
6	32628036	chr6:32628036:G:C	HLA-DQA1	Intergenic	rs9271892	G>C	10	1.68x10-33	1.42 [1.34-1.50]	0.859	0
6	32628082	chr6:32628082:G:T	HLA-DQA1	Intergenic	rs9271893	G>T	10	1.68x10-33	1.42 [1.34-1.50]	0.859	0
6	32628097	chr6:32628097:C:T	HLA-DQA1	Intergenic	rs9271894	C>T	10	4.87x10-09	0.84 [0.79-0.89]	0.165	30.48
6	32628130	chr6:32628130:G:A	HLA-DQA1	Intergenic	rs9271896	G>A	10	1.68x10-33	1.42 [1.34-1.50]	0.859	0
6	32628177	chr6:32628177:G:A	HLA-DQA1	Intergenic	rs9271897	G>A	10	1.68x10-33	1.42 [1.34-1.50]	0.859	0
6	32632434	chr6:32632434:G:A	HLA-DQA1	Intergenic	rs34927613	G>A	10	3.41x10-36	0.63 [0.59-0.68]	0.243	21.74
6	32632451	chr6:32632451:T:C	HLA-DQA1	Intergenic	rs9272108	T>C	10	1.68x10-33	1.42 [1.34-1.50]	0.859	0
6	32632472	chr6:32632472:A:C	HLA-DQA1	Intergenic	rs13211123	A>C	10	3.41x10-36	0.63 [0.59-0.68]	0.243	21.74
6	32632476	chr6:32632476:G:T	HLA-DQA1	Intergenic	rs9272109	G>T	10	1.68x10-33	1.42 [1.34-1.50]	0.859	0
6	32632510	chr6:32632510:T:C	HLA-DQA1	Intergenic	rs9272110	T>C	10	1.29x10-33	1.42 [1.34-1.50]	0.866	0
6	32632514	chr6:32632514:T:C	HLA-DQA1	Intergenic	rs9272113	T>C	10	1.70x10-33	1.42 [1.34-1.50]	0.858	0
6	32632540	chr6:32632540:T:C	HLA-DQA1	Intergenic	rs9272114	T>C	10	1.25x10-38	1.46 [1.38-1.54]	0.776	0
6	32632598	chr6:32632598:T:C	HLA-DQA1	Intergenic	rs3104373	T>C	8	5.58x10-14	0.65 [0.59-0.73]	0.928	0
6	32632627	chr6:32632627:T:C	HLA-DQA1	Intergenic	rs9272116	T>C	10	1.25x10-38	1.46 [1.38-1.54]	0.776	0
6	32632634	chr6:32632634:C:T	HLA-DQA1	Intergenic	rs9272117	C>T	10	1.68x10-33	1.42 [1.34-1.50]	0.859	0
6	32632671	chr6:32632671:C:T	HLA-DQA1	Intergenic	rs9272118	C>T	10	1.25x10-38	1.46 [1.38-1.54]	0.776	0
6	32632691	chr6:32632691:G:A	HLA-DQA1	Intergenic	rs13211715	G>A	8	2.74x10-14	0.46 [0.38-0.56]	0.852	0
6	32632693	chr6:32632693:T:C	HLA-DQA1	Intergenic	rs9272119	T>C	10	1.68x10-33	1.42 [1.34-1.50]	0.859	0
6	32632698	chr6:32632698:G:A	HLA-DQA1	Intergenic	rs114883129	G>A	5	1.61x10-25	2.41 [2.06-2.82]	0.612	0
6	32632724	chr6:32632724:A:G	HLA-DQA1	Intergenic	rs9272120	A>G	10	1.68x10-33	1.42 [1.34-1.50]	0.859	0
6	32632771	chr6:32632771:C:G	HLA-DQA1	Intergenic	rs13211628	C>G	10	1.50x10-11	0.65 [0.57-0.73]	0.045	47.85
6	32632778	chr6:32632778:C:T	HLA-DQA1	Intergenic	rs13211632	C>T	10	1.50x10-11	0.65 [0.57-0.73]	0.045	47.85
6	32632779	chr6:32632779:G:C	HLA-DQA1	Intergenic	rs9272121	G>C	10	1.68x10-33	1.42 [1.34-1.50]	0.859	0
6	32632785	chr6:32632785:C:T	HLA-DQA1	Intergenic	rs9272122	C>T	10	2.45x10-20	1.32 [1.25-1.40]	0.987	0
6	32632804	chr6:32632804:G:A	HLA-DQA1	Intergenic	rs9272124	G>A	10	1.78x10-33	1.42 [1.34-1.50]	0.86	0
6	32632807	chr6:32632807:T:G	HLA-DQA1	Intergenic	rs9272125	T>G	10	1.15x10-38	1.46 [1.38-1.54]	0.773	0
6	32632812	chr6:32632812:G:C	HLA-DQA1	Intergenic	rs9272126	G>C	10	1.15x10-38	1.46 [1.38-1.54]	0.773	0
6	32632818	chr6:32632818:T:C	HLA-DQA1	Intergenic	rs9272127	T>C	10	1.19x10-38	1.46 [1.38-1.54]	0.774	0
6	32632861	chr6:32632861:A:G	HLA-DQA1	Intergenic	rs9272130	A>G	10	1.71x10-33	1.42 [1.34-1.50]	0.859	0
6	32632894	chr6:32632894:C:T	HLA-DQA1	Intergenic	rs9272131	C>T	10	3.57x10-69	2.13 [1.97-2.30]	0.783	0
6	32632900	chr6:32632900:A:G	HLA-DQA1	Intergenic	rs9272132	A>G	10	1.15x10-38	1.46 [1.38-1.54]	0.773	0
6	32632928	chr6:32632928:C:G	HLA-DQA1	Intergenic	rs9272134	C>G	10	1.71x10-33	1.42 [1.34-1.50]	0.859	0
6	32632946	chr6:32632946:G:A	HLA-DQA1	Intergenic	rs9272135	G>A	10	1.26x10-33	1.42 [1.35-1.50]	0.866	0
6	32632953	chr6:32632953:C:T	HLA-DQA1	Intergenic	rs9272136	C>T	10	1.33x10-33	1.42 [1.34-1.50]	0.867	0
6	32632961	chr6:32632961:A:C	HLA-DQA1	Intergenic	rs9272137	A>C	10	1.71x10-33	1.42 [1.34-1.50]	0.859	0
6	32632980	chr6:32632980:G:A	HLA-DQA1	Intergenic	rs9272138	G>A	10	1.88x10-33	1.42 [1.34-1.50]	0.862	0
6	32633020	chr6:32633020:C:A	HLA-DQA1	Intergenic	rs9272141	C>A	10	1.87x10-33	1.42 [1.34-1.50]	0.857	0
6	32633023	chr6:32633023:G:C	HLA-DQA1	Intergenic	rs9272142	G>C	10	1.28x10-38	1.46 [1.38-1.54]	0.767	0
6	32633026	chr6:32633026:T:C	HLA-DQA1	Intergenic	rs9272143	T>C	10	1.87x10-33	1.42 [1.34-1.50]	0.857	0
6	32633137	chr6:32633137:T:C	HLA-DQA1	Intergenic	rs9272146	T>C	10	1.71x10-33	1.42 [1.34-1.50]	0.859	0

6	32633140	chr6:32633140:G:T	HLA-DQA1	Intergenic	rs34153292	G>T	9	1.30x10-08	0.61 [0.51-0.72]	0.808	0
6	32633206	chr6:32633206:C:T	HLA-DQA1	Intergenic	rs3104372	C>T	9	1.45x10-77	1.94 [1.81-2.07]	0.677	0
6	32633277	chr6:32633277:C:A	HLA-DQA1	Intergenic	rs13215343	C>A	7	1.85x10-14	0.45 [0.36-0.54]	0.924	0
6	32633493	chr6:32633493:C:T	HLA-DQA1	Intergenic	rs558837594	C>T	7	1.68x10-14	0.45 [0.36-0.54]	0.923	0
6	32633907	chr6:32633907:T:G	HLA-DQA1	Intergenic	rs2002779	T>G	10	5.79x10-15	0.70 [0.64-0.76]	0.157	31.43
6	32633931	chr6:32633931:G:A	HLA-DQA1	Intergenic	rs114803358	G>A	10	4.22x10-37	0.63 [0.59-0.67]	0.27	18.82
6	32633941	chr6:32633941:C:T	HLA-DQA1	Intergenic	rs115566240	C>T	10	8.40x10-41	0.62 [0.58-0.67]	0.145	32.92
6	32633942	chr6:32633942:G:A	HLA-DQA1	Intergenic	rs114567049	G>A	10	3.93x10-37	0.63 [0.58-0.67]	0.272	18.64
6	32634288	chr6:32634288:T:C	HLA-DQA1	Intergenic	rs72848263	T>C	10	3.93x10-37	0.63 [0.58-0.67]	0.272	18.64
6	32634319	chr6:32634319:G:A	HLA-DQA1	Intergenic	rs9272214	G>A	10	1.15x10-38	1.46 [1.38-1.54]	0.773	0
6	32634347	chr6:32634347:C:T	HLA-DQA1	Intergenic	rs34145408	C>T	10	4.12x10-37	0.63 [0.59-0.67]	0.273	18.51
6	32634360	chr6:32634360:C:G	HLA-DQA1	Intergenic	rs3104371	C>G	10	6.55x10-83	1.97 [1.84-2.10]	0.791	0
6	32634402	chr6:32634402:G:A	HLA-DQA1	Intergenic	rs17211454	G>A	10	3.93x10-37	0.63 [0.58-0.67]	0.272	18.64
6	32634419	chr6:32634419:T:G	HLA-DQA1	Intergenic	rs17205114	T>G	10	3.93x10-37	0.63 [0.58-0.67]	0.272	18.64
6	32634440	chr6:32634440:C:G	HLA-DQA1	Intergenic	rs17205128	C>G	10	3.93x10-37	0.63 [0.58-0.67]	0.272	18.64
6	32634445	chr6:32634445:A:G	HLA-DQA1	Intergenic	rs17205135	A>G	10	4.21x10-37	0.63 [0.59-0.67]	0.273	18.49
6	32634461	chr6:32634461:C:T	HLA-DQA1	Intergenic	rs9272218	C>T	10	1.15x10-38	1.46 [1.38-1.54]	0.773	0
6	32634531	chr6:32634531:T:C	HLA-DQA1	Intergenic	rs183736834	T>C	9	1.30x10-08	0.61 [0.51-0.72]	0.808	0
6	32634557	chr6:32634557:C:A	HLA-DQA1	Intergenic	rs9272221	C>A	10	1.15x10-38	1.46 [1.38-1.54]	0.773	0
6	32634564	chr6:32634564:G:A	HLA-DQA1	Intergenic	rs3104370	G>A	10	5.79x10-15	0.70 [0.64-0.76]	0.157	31.43
6	32634619	chr6:32634619:C:T	HLA-DQA1	Intergenic	rs9272226	C>T	10	1.73x10-25	1.37 [1.30-1.46]	0.922	0
6	32634653	chr6:32634653:C:A	HLA-DQA1	Intergenic	rs17211510	C>A	10	3.66x10-37	0.63 [0.58-0.67]	0.27	18.83
6	32634705	chr6:32634705:T:C	HLA-DQA1	Intergenic	rs3104369	T>C	10	1.75x10-21	0.70 [0.65-0.75]	0.308	14.67
6	32634706	chr6:32634706:G:T	HLA-DQA1	Intergenic	rs17205170	G>T	9	2.62x10-10	0.56 [0.47-0.67]	0.004	64.5
6	32634741	chr6:32634741:A:G	HLA-DQA1	Intergenic	rs17205177	A>G	10	3.61x10-37	0.63 [0.58-0.67]	0.27	18.84
6	32634774	chr6:32634774:G:A	HLA-DQA1	Intergenic	rs17205184	G>A	10	1.52x10-11	0.65 [0.57-0.73]	0.046	47.69
6	32634779	chr6:32634779:G:A	HLA-DQA1	Intergenic	rs17205191	G>A	10	7.75x10-41	0.62 [0.58-0.67]	0.144	32.96
6	32634791	chr6:32634791:C:T	HLA-DQA1	Intergenic	rs9272229	C>T	10	1.10x10-38	1.46 [1.38-1.54]	0.772	0
6	32634831	chr6:32634831:T:C	HLA-DQA1	Intergenic	rs17211545	T>C	10	3.66x10-37	0.63 [0.58-0.67]	0.27	18.83
6	32634840	chr6:32634840:C:T	HLA-DQA1	Intergenic	rs9272231	C>T	10	2.56x10-39	1.46 [1.39-1.55]	0.776	0
6	32634853	chr6:32634853:C:T	HLA-DQA1	Intergenic	rs9272232	C>T	10	1.10x10-38	1.46 [1.38-1.54]	0.772	0
6	32634977	chr6:32634977:G:A	HLA-DQA1	Intergenic	rs17211580	G>A	10	3.66x10-37	0.63 [0.58-0.67]	0.27	18.83
6	32635001	chr6:32635001:A:G	HLA-DQA1	Intergenic	rs9272239	A>G	10	1.13x10-38	1.46 [1.38-1.54]	0.772	0
6	32635013	chr6:32635013:T:C	HLA-DQA1	Intergenic	rs9272242	T>C	10	1.10x10-38	1.46 [1.38-1.54]	0.772	0
6	32635040	chr6:32635040:T:G	HLA-DQA1	Intergenic	rs17211601	T>G	10	2.70x10-37	0.63 [0.58-0.67]	0.261	19.81
6	32635087	chr6:32635087:T:A	HLA-DQA1	Intergenic	rs28533091	T>A	10	3.66x10-37	0.63 [0.58-0.67]	0.27	18.83
6	32635095	chr6:32635095:C:G	HLA-DQA1	Intergenic	rs9272245	C>G	10	2.23x10-25	1.37 [1.30-1.45]	0.927	0
6	32635252	chr6:32635252:T:C	HLA-DQA1	Intergenic	rs28694749	T>C	10	3.66x10-37	0.63 [0.58-0.67]	0.27	18.83
6	32635321	chr6:32635321:C:T	HLA-DQA1	Intergenic	rs9272254	C>T	10	1.10x10-38	1.46 [1.38-1.54]	0.772	0
6	32635500	chr6:32635500:G:T	HLA-DQA1	Intergenic	rs28848172	G>T	9	1.30x10-08	0.61 [0.51-0.72]	0.808	0
6	32635544	chr6:32635544:C:T	HLA-DQA1	Intergenic	rs62404084	C>T	10	1.53x10-11	0.65 [0.57-0.73]	0.046	47.71
6	32635578	chr6:32635578:T:C	HLA-DQA1	Intergenic	rs28407322	T>C	10	2.20x10-37	0.63 [0.58-0.67]	0.301	15.48
6	32635635	chr6:32635635:C:T	HLA-DQA1	Intergenic	rs9272265	C>T	10	1.10x10-38	1.46 [1.38-1.54]	0.772	0
6	32635639	chr6:32635639:G:A	HLA-DQA1	Intergenic	rs9272266	G>A	10	3.66x10-37	0.63 [0.58-0.67]	0.27	18.83
6	32635710	chr6:32635710:T:C	HLA-DQA1	Intergenic	rs3104367	T>C	10	8.10x10-20	0.57 [0.51-0.64]	0.006	61.15
6	32635711	chr6:32635711:A:G	HLA-DQA1	Intergenic	rs28675927	A>G	10	3.66x10-37	0.63 [0.58-0.67]	0.27	18.83

6	32635883	chr6:32635883:T:C	HLA-DQA1	Intergenic	rs1391373	T>C	10	7.69x10-21	1.33 [1.26-1.41]	0.318	13.63
6	32635965	chr6:32635965:A:G	HLA-DQA1	Intergenic	rs9272293	A>G	10	7.02x10-14	0.62 [0.55-0.70]	0.002	65.29
6	32636021	chr6:32636021:A:T	HLA-DQA1	Intergenic	rs1391371	A>T	10	1.11x10-82	1.96 [1.84-2.10]	0.782	0
6	32636048	chr6:32636048:C:T	HLA-DQA1	Intergenic	rs3129774	C>T	10	2.14x10-17	1.56 [1.41-1.72]	0.022	53.62
6	32636119	chr6:32636119:T:C	HLA-DQA1	Intergenic	rs36226286	T>C	9	1.30x10-08	0.61 [0.51-0.72]	0.808	0
6	32636191	chr6:32636191:A:G	HLA-DQA1	Intergenic	rs3104364	A>G	10	8.10x10-20	0.57 [0.51-0.64]	0.006	61.15
6	32636223	chr6:32636223:G:T	HLA-DQA1	Intergenic	rs3104363	G>T	10	8.10x10-20	0.57 [0.51-0.64]	0.006	61.15
6	32636331	chr6:32636331:T:C	HLA-DQA1	Intergenic	rs9272319	T>C	10	6.42x10-83	1.78 [1.69-1.88]	0.171	29.78
6	32636347	chr6:32636347:G:A	HLA-DQA1	Intergenic	rs9272320	G>A	10	7.07x10-21	0.57 [0.50-0.63]	0.005	61.97
6	32636482	chr6:32636482:C:T	HLA-DQA1	Intergenic	rs9272335	C>T	10	6.13x10-83	1.78 [1.69-1.88]	0.163	30.69
6	32636487	chr6:32636487:G:T	HLA-DQA1	Intergenic	rs9272336	G>T	10	7.00x10-15	0.70 [0.64-0.76]	0.166	30.35
6	32636595	chr6:32636595:G:A	HLA-DQA1	Intergenic	rs9272346	G>A	10	8.33x10-20	0.57 [0.51-0.64]	0.006	61.22
6	32636617	chr6:32636617:T:C	HLA-DQA1	Intergenic	rs9272347	T>C	10	5.69x10-83	1.78 [1.69-1.88]	0.166	30.37
6	32636625	chr6:32636625:G:A	HLA-DQA1	Intergenic	rs9272349	G>A	10	6.48x10-15	0.70 [0.64-0.76]	0.159	31.22
6	32636633	chr6:32636633:T:A	HLA-DQA1	Intergenic	rs9272350	T>A	10	8.10x10-20	0.57 [0.51-0.64]	0.006	61.15
6	32636679	chr6:32636679:G:C	HLA-DQA1	Intergenic	rs9272353	G>C	10	5.97x10-83	1.97 [1.84-2.10]	0.789	0
6	32636738	chr6:32636738:G:C	HLA-DQA1	Intergenic	rs9272355	G>C	10	6.68x10-20	0.57 [0.51-0.64]	0.006	60.99
6	32636745	chr6:32636745:C:A	HLA-DQA1	Intergenic	rs9272357	C>A	10	8.10x10-20	0.57 [0.51-0.64]	0.006	61.15
6	32643695	chr6:32643695:A:G	HLA-DQA1	Intergenic	rs5002185	A>G	10	7.70x10-20	0.57 [0.51-0.64]	0.006	61.26
6	32643760	chr6:32643760:C:T	HLA-DQA1	Intergenic	rs9273007	C>T	10	6.42x10-83	1.78 [1.69-1.88]	0.171	29.78
6	32643795	chr6:32643795:T:A	HLA-DQA1	Intergenic	rs9273008	T>A	10	6.42x10-83	1.78 [1.69-1.88]	0.171	29.78
6	32643802	chr6:32643802:A:G	HLA-DQA1	Intergenic	rs9273009	A>G	10	6.42x10-83	1.78 [1.69-1.88]	0.171	29.78
6	32643813	chr6:32643813:G:A	HLA-DQA1	Intergenic	rs5002178	G>A	10	1.22x10-14	0.56 [0.49-0.65]	0	70.12
6	32643870	chr6:32643870:G:C	HLA-DQA1	Intergenic	rs28566533	G>C	8	3.58x10-14	0.46 [0.38-0.56]	0.875	0
6	32643872	chr6:32643872:C:T	HLA-DQA1	Intergenic	rs9273013	C>T	10	8.10x10-20	0.57 [0.51-0.64]	0.006	61.15
6	32643873	chr6:32643873:G:A	HLA-DQA1	Intergenic	rs9273014	G>A	10	7.89x10-83	1.78 [1.69-1.88]	0.174	29.46
6	32643917	chr6:32643917:G:A	HLA-DQA1	Intergenic	rs4526236	G>A	10	4.45x10-10	0.83 [0.78-0.88]	0.363	8.6
6	32643946	chr6:32643946:G:T	HLA-DQA1	Intergenic	rs9273020	G>T	10	8.68x10-20	0.57 [0.51-0.64]	0.006	61.25
6	32643948	chr6:32643948:A:G	HLA-DQA1	Intergenic	rs9273021	A>G	10	8.52x10-20	0.57 [0.51-0.64]	0.006	61.23
6	32643987	chr6:32643987:T:C	HLA-DQA1	Intergenic	rs36214157	T>C	8	3.58x10-14	0.46 [0.38-0.56]	0.875	0
6	32644083	chr6:32644083:A:G	HLA-DQA1	Intergenic	rs9273032	A>G	8	6.66x10-14	0.65 [0.59-0.73]	0.929	0
6	32644096	chr6:32644096:T:C	HLA-DQA1	Intergenic	rs9273033	T>C	10	8.83x10-20	0.57 [0.51-0.64]	0.006	61.28
6	32644097	chr6:32644097:G:T	HLA-DQA1	Intergenic	rs9273034	G>T	10	8.83x10-20	0.57 [0.51-0.64]	0.006	61.28
6	32644121	chr6:32644121:A:G	HLA-DQA1	Intergenic	rs9273036	A>G	10	1.33x10-82	1.78 [1.68-1.88]	0.155	31.67
6	32644154	chr6:32644154:A:T	HLA-DQA1	Intergenic	rs9273038	A>T	10	2.25x10-19	0.57 [0.51-0.64]	0.005	61.87
6	32644191	chr6:32644191:G:T	HLA-DQA1	Intergenic	rs9273043	G>T	10	8.10x10-20	0.57 [0.51-0.64]	0.006	61.15
6	32644226	chr6:32644226:C:T	HLA-DQA1	Intergenic	rs9273046	C>T	10	8.10x10-20	0.57 [0.51-0.64]	0.006	61.15
6	32644231	chr6:32644231:T:C	HLA-DQA1	Intergenic	rs9273048	T>C	10	5.91x10-83	1.78 [1.69-1.88]	0.171	29.77
6	32644257	chr6:32644257:C:T	HLA-DQA1	Intergenic	rs9273049	C>T	10	8.10x10-20	0.57 [0.51-0.64]	0.006	61.15
6	32644264	chr6:32644264:A:G	HLA-DQA1	Intergenic	rs9273052	A>G	10	6.26x10-83	1.78 [1.69-1.88]	0.17	29.85
6	32644273	chr6:32644273:A:T	HLA-DQA1	Intergenic	rs9273053	A>T	10	8.36x10-20	0.57 [0.51-0.64]	0.006	61.2
6	32644320	chr6:32644320:T:G	HLA-DQA1	Intergenic	rs9273056	T>G	10	8.10x10-20	0.57 [0.51-0.64]	0.006	61.15
6	32644327	chr6:32644327:C:T	HLA-DQA1	Intergenic	rs9273057	C>T	10	6.42x10-83	1.78 [1.69-1.88]	0.171	29.78
6	32644333	chr6:32644333:T:C	HLA-DQA1	Intergenic	rs9273058	T>C	10	4.54x10-24	0.74 [0.70-0.79]	0.184	28.3
6	32644334	chr6:32644334:G:A	HLA-DQA1	Intergenic	rs9273059	G>A	10	5.79x10-83	1.78 [1.69-1.88]	0.17	29.94
6	32644384	chr6:32644384:C:A	HLA-DQA1	Intergenic	rs9273062	C>A	10	5.28x10-83	1.78 [1.69-1.88]	0.165	30.49

6	32644389	chr6:32644389:C:T	HLA-DQA1	Intergenic	rs9273063	C>T	10	9.01x10-83	1.78 [1.69-1.88]	0.164	30.6
6	32644428	chr6:32644428:T:G	HLA-DQA1	Intergenic	rs9273065	T>G	10	6.50x10-20	0.57 [0.51-0.64]	0.006	60.85
6	32644431	chr6:32644431:G:A	HLA-DQA1	Intergenic	rs9273066	G>A	10	8.10x10-20	0.57 [0.51-0.64]	0.006	61.15
6	32644436	chr6:32644436:C:A	HLA-DQA1	Intergenic	rs9273067	C>A	10	8.58x10-26	0.64 [0.59-0.70]	0.329	12.4
6	32644456	chr6:32644456:C:T	HLA-DQA1	Intergenic	rs9273069	C>T	10	8.10x10-20	0.57 [0.51-0.64]	0.006	61.15
6	32644464	chr6:32644464:A:T	HLA-DQA1	Intergenic	rs9273070	A>T	10	6.42x10-83	1.78 [1.69-1.88]	0.171	29.78
6	32644474	chr6:32644474:T:G	HLA-DQA1	Intergenic	rs9273071	T>G	10	6.15x10-83	1.78 [1.69-1.88]	0.169	30.04
6	32644502	chr6:32644502:T:C	HLA-DQA1	Intergenic	rs9273073	T>C	10	6.64x10-83	1.78 [1.69-1.88]	0.171	29.82
6	32644510	chr6:32644510:A:G	HLA-DQA1	Intergenic	rs9273074	A>G	10	8.15x10-20	0.57 [0.51-0.64]	0.006	61.16
6	32644511	chr6:32644511:A:G	HLA-DQA1	Intergenic	rs9273075	A>G	10	8.10x10-20	0.57 [0.51-0.64]	0.006	61.15
6	32644524	chr6:32644524:T:C	HLA-DQA1	Intergenic	rs9273076	T>C	8	7.59x10-14	0.65 [0.59-0.73]	0.923	0
6	32644532	chr6:32644532:T:A	HLA-DQA1	Intergenic	rs9273078	T>A	10	7.55x10-20	0.57 [0.51-0.64]	0.006	61.12
6	32644549	chr6:32644549:C:T	HLA-DQA1	Intergenic	rs9273079	C>T	10	6.64x10-83	1.78 [1.69-1.88]	0.171	29.82
6	32644559	chr6:32644559:G:A	HLA-DQA1	Intergenic	rs9273081	G>A	10	7.40x10-19	0.57 [0.51-0.65]	0.004	62.88
6	32644562	chr6:32644562:C:T	HLA-DQA1	Intergenic	rs9273082	C>T	10	8.10x10-20	0.57 [0.51-0.64]	0.006	61.15
6	32644566	chr6:32644566:T:C	HLA-DQA1	Intergenic	rs9273083	T>C	10	8.10x10-20	0.57 [0.51-0.64]	0.006	61.15
6	32644619	chr6:32644619:T:C	HLA-DQA1	Intergenic	rs9273084	T>C	10	8.10x10-20	0.57 [0.51-0.64]	0.006	61.15
6	32644620	chr6:32644620:A:G	HLA-DQA1	Intergenic	rs6927022	A>G	9	4.30x10-15	1.67 [1.48-1.89]	0.001	68.25
6	32644633	chr6:32644633:C:T	HLA-DQA1	Intergenic	rs9273086	C>T	10	8.42x10-20	0.57 [0.51-0.64]	0.006	61.21
6	32645109	chr6:32645109:T:C	HLA-DQA1	Intergenic	rs9273142	T>C	10	7.68x10-20	0.57 [0.51-0.64]	0.006	60.79
6	32645122	chr6:32645122:T:G	HLA-DQA1	Intergenic	rs9273143	T>G	10	8.10x10-20	0.57 [0.51-0.64]	0.006	61.15
6	32645202	chr6:32645202:A:T	HLA-DQA1	Intergenic	rs9273146	A>T	10	8.10x10-20	0.57 [0.51-0.64]	0.006	61.15
6	32645204	chr6:32645204:A:G	HLA-DQA1	Intergenic	rs9273147	A>G	10	8.10x10-20	0.57 [0.51-0.64]	0.006	61.15
6	32645205	chr6:32645205:T:A	HLA-DQA1	Intergenic	rs9273148	T>A	10	6.64x10-83	1.78 [1.69-1.88]	0.171	29.82
6	32645210	chr6:32645210:G:A	HLA-DQA1	Intergenic	rs9273149	G>A	10	6.64x10-83	1.78 [1.69-1.88]	0.171	29.82
6	32645273	chr6:32645273:G:A	HLA-DQA1	Intergenic	rs9273156	G>A	10	7.39x10-20	0.57 [0.51-0.64]	0.006	61.1
6	32645285	chr6:32645285:G:C	HLA-DQA1	Intergenic	rs9273158	G>C	10	5.05x10-20	0.57 [0.51-0.64]	0.007	60.51
6	32645447	chr6:32645447:A:C	HLA-DQA1	Intergenic	rs9273172	A>C	10	8.16x10-20	0.57 [0.51-0.64]	0.006	61.17
6	32645969	chr6:32645969:C:A	HLA-DQA1	Intergenic	rs9273216	C>A	10	8.49x10-20	0.57 [0.51-0.64]	0.006	61.22
6	32645979	chr6:32645979:C:A	HLA-DQA1	Intergenic	rs9273218	C>A	10	8.33x10-20	0.57 [0.51-0.64]	0.006	61.19
6	32646027	chr6:32646027:A:T	HLA-DQA1	Intergenic	rs9273224	A>T	10	8.10x10-20	0.57 [0.51-0.64]	0.006	61.15
6	32646040	chr6:32646040:A:G	HLA-DQA1	Intergenic	rs9273225	A>G	10	8.10x10-20	0.57 [0.51-0.64]	0.006	61.15
6	32646047	chr6:32646047:C:T	HLA-DQA1	Intergenic	rs9273226	C>T	10	7.88x10-20	0.57 [0.51-0.64]	0.006	61.11
6	32646248	chr6:32646248:C:T	HLA-DQA1	Intergenic	rs9273241	C>T	10	8.10x10-20	0.57 [0.51-0.64]	0.006	61.15
6	32646260	chr6:32646260:C:T	HLA-DQA1	Intergenic	rs34276369	C>T	10	2.80x10-83	1.78 [1.69-1.89]	0.171	29.74
6	32646303	chr6:32646303:T:C	HLA-DQA1	Intergenic	rs9273242	T>C	10	8.10x10-20	0.57 [0.51-0.64]	0.006	61.15
6	32646314	chr6:32646314:G:A	HLA-DQA1	Intergenic	rs9273243	G>A	10	8.50x10-20	0.57 [0.51-0.64]	0.006	61.19
6	32646317	chr6:32646317:C:G	HLA-DQA1	Intergenic	rs115210925	C>G	10	2.80x10-83	1.78 [1.69-1.89]	0.171	29.74
6	32646331	chr6:32646331:A:G	HLA-DQA1	Intergenic	rs9273244	A>G	10	4.74x10-20	0.57 [0.51-0.64]	0.007	60.37
6	32646333	chr6:32646333:G:A	HLA-DQA1	Intergenic	rs9273245	G>A	10	4.74x10-20	0.57 [0.51-0.64]	0.007	60.37
6	32646949	chr6:32646949:C:T	HLA-DQA1	Intergenic	rs17612482	C>T	10	8.10x10-20	0.57 [0.51-0.64]	0.006	61.15
6	32646972	chr6:32646972:T:A	HLA-DQA1	Intergenic	rs17612489	T>A	10	7.50x10-20	0.57 [0.51-0.64]	0.006	61.1
6	32646975	chr6:32646975:T:G	HLA-DQA1	Intergenic	rs17612496	T>G	10	7.50x10-20	0.57 [0.51-0.64]	0.006	61.1
6	32646995	chr6:32646995:G:A	HLA-DQA1	Intergenic	rs796601656	G>A	10	7.50x10-20	0.57 [0.51-0.64]	0.006	61.1
6	32646996	chr6:32646996:A:G	HLA-DQA1	Intergenic	rs796291650	A>G	10	7.50x10-20	0.57 [0.51-0.64]	0.006	61.1
6	32646997	chr6:32646997:G:C	HLA-DQA1	Intergenic	rs796457056	G>C	10	7.50x10-20	0.57 [0.51-0.64]	0.006	61.1

6	32647065	chr6:32647065:A:G	HLA-DQA1	Intergenic	rs17843564	A>G	10	6.40x10-20	0.57 [0.51-0.64]	0.006	60.83
6	32647070	chr6:32647070:A:G	HLA-DQA1	Intergenic	rs17843565	A>G	10	8.20x10-20	0.57 [0.51-0.64]	0.006	61.17
6	32647071	chr6:32647071:G:T	HLA-DQA1	Intergenic	rs17843566	G>T	10	8.20x10-20	0.57 [0.51-0.64]	0.006	61.17
6	32647089	chr6:32647089:A:G	HLA-DQA1	Intergenic	rs17843567	A>G	10	8.20x10-20	0.57 [0.51-0.64]	0.006	61.17
6	32647095	chr6:32647095:G:C	HLA-DQA1	Intergenic	rs17612503	G>C	8	7.59x10-14	0.65 [0.59-0.73]	0.923	0
6	32647106	chr6:32647106:C:T	HLA-DQA1	Intergenic	rs17612510	C>T	10	8.10x10-20	0.57 [0.51-0.64]	0.006	61.15
6	32647140	chr6:32647140:C:T	HLA-DQA1	Intergenic	rs28375404	C>T	9	1.24x10-08	0.61 [0.51-0.72]	0.795	0
6	32647569	chr6:32647569:C:G	HLA-DQA1	Intergenic	rs28609302	C>G	8	3.77x10-14	0.46 [0.38-0.56]	0.877	0
6	32647589	chr6:32647589:G:A	HLA-DQA1	Intergenic	rs17612555	G>A	10	8.10x10-20	0.57 [0.51-0.64]	0.006	61.15
6	32647623	chr6:32647623:C:T	HLA-DQA1	Intergenic	rs17843573	C>T	10	7.91x10-20	0.57 [0.51-0.64]	0.006	61.11
6	32647644	chr6:32647644:A:G	HLA-DQA1	Intergenic	rs7745002	A>G	9	2.02x10-15	1.67 [1.48-1.89]	0.002	67.58
6	32647650	chr6:32647650:C:T	HLA-DQA1	Intergenic	rs17843575	C>T	10	8.10x10-20	0.57 [0.51-0.64]	0.006	61.15
6	32647654	chr6:32647654:G:A	HLA-DQA1	Intergenic	rs17843576	G>A	10	8.10x10-20	0.57 [0.51-0.64]	0.006	61.15
6	32647669	chr6:32647669:T:G	HLA-DQA1	Intergenic	rs17612562	T>G	10	8.10x10-20	0.57 [0.51-0.64]	0.006	61.15
6	32647680	chr6:32647680:T:C	HLA-DQA1	Intergenic	rs28479464	T>C	10	7.13x10-11	0.65 [0.57-0.74]	0.029	51.65
6	32647681	chr6:32647681:G:A	HLA-DQA1	Intergenic	rs17612576	G>A	10	8.10x10-20	0.57 [0.51-0.64]	0.006	61.15
6	32647693	chr6:32647693:G:A	HLA-DQA1	Intergenic	rs17612583	G>A	10	8.10x10-20	0.57 [0.51-0.64]	0.006	61.15
6	32647733	chr6:32647733:C:T	HLA-DQA1	Intergenic	rs17843577	C>T	10	8.10x10-20	0.57 [0.51-0.64]	0.006	61.15
6	32647743	chr6:32647743:G:A	HLA-DQA1	Intergenic	rs17843578	G>A	10	8.10x10-20	0.57 [0.51-0.64]	0.006	61.15
6	32647750	chr6:32647750:T:G	HLA-DQA1	Intergenic	rs17843579	T>G	10	8.10x10-20	0.57 [0.51-0.64]	0.006	61.15
6	32647774	chr6:32647774:A:G	HLA-DQA1	Intergenic	rs17843580	A>G	10	8.10x10-20	0.57 [0.51-0.64]	0.006	61.15
6	32647801	chr6:32647801:G:A	HLA-DQA1	Intergenic	rs17612590	G>A	10	7.35x10-20	0.57 [0.51-0.64]	0.006	61.11
6	32647805	chr6:32647805:A:C	HLA-DQA1	Intergenic	rs17612597	A>C	10	7.35x10-20	0.57 [0.51-0.64]	0.006	61.1
6	32647820	chr6:32647820:A:C	HLA-DQA1	Intergenic	rs17612604	A>C	10	8.10x10-20	0.57 [0.51-0.64]	0.006	61.15
6	32647834	chr6:32647834:A:T	HLA-DQA1	Intergenic	rs17612610	A>T	10	8.10x10-20	0.57 [0.51-0.64]	0.006	61.15
6	32647836	chr6:32647836:G:T	HLA-DQA1	Intergenic	rs17612617	G>T	10	8.10x10-20	0.57 [0.51-0.64]	0.006	61.15
6	32647861	chr6:32647861:A:C	HLA-DQA1	Intergenic	rs17843581	A>C	10	8.36x10-20	0.57 [0.51-0.64]	0.006	61.2
6	32647874	chr6:32647874:G:A	HLA-DQA1	Intergenic	rs17843582	G>A	10	8.20x10-20	0.57 [0.51-0.64]	0.006	61.17
6	32647875	chr6:32647875:A:C	HLA-DQA1	Intergenic	rs17843583	A>C	10	6.68x10-20	0.57 [0.51-0.64]	0.006	61.02
6	32647891	chr6:32647891:G:T	HLA-DQA1	Intergenic	rs17843584	G>T	10	8.10x10-20	0.57 [0.51-0.64]	0.006	61.15
6	32647925	chr6:32647925:A:C	HLA-DQA1	Intergenic	rs17612625	A>C	10	8.10x10-20	0.57 [0.51-0.64]	0.006	61.15
6	32647933	chr6:32647933:G:C	HLA-DQA1	Intergenic	rs17612633	G>C	10	8.10x10-20	0.57 [0.51-0.64]	0.006	61.15
6	32647977	chr6:32647977:G:A	HLA-DQA1	Intergenic	rs17612640	G>A	10	8.10x10-20	0.57 [0.51-0.64]	0.006	61.15
6	32652425	chr6:32652425:G:A	-	Intergenic	rs41269974	G>A	10	1.60x10-87	2.03 [1.90-2.17]	0.932	0
6	32652464	chr6:32652464:G:A	-	Intergenic	rs17843603	G>A	10	7.98x10-20	0.57 [0.51-0.64]	0.006	60.95
6	32652478	chr6:32652478:T:C	-	Intergenic	rs9348889	T>C	10	3.83x10-83	1.78 [1.69-1.89]	0.171	29.76
6	32652506	chr6:32652506:C:T	-	Intergenic	rs17843604	C>T	10	7.49x10-21	0.57 [0.50-0.63]	0.005	61.78
6	32652509	chr6:32652509:T:C	-	Intergenic	rs12527758	T>C	10	7.49x10-21	0.57 [0.50-0.63]	0.005	61.78
6	32652534	chr6:32652534:T:C	-	Intergenic	rs17612781	T>C	10	8.33x10-20	0.57 [0.51-0.64]	0.006	61
6	32652567	chr6:32652567:G:A	-	Intergenic	rs17612788	G>A	10	7.32x10-20	0.57 [0.51-0.64]	0.006	61.04
6	32652795	chr6:32652795:G:A	-	Intergenic	rs17612852	G>A	10	3.22x10-29	0.66 [0.62-0.71]	0.374	7.34
6	32652845	chr6:32652845:A:T	-	Intergenic	rs17612858	A>T	10	7.82x10-20	0.57 [0.51-0.64]	0.006	61.1
6	32652879	chr6:32652879:A:T	-	Intergenic	rs71542424	A>T	8	4.32x10-14	0.46 [0.38-0.56]	0.88	0
6	32653050	chr6:32653050:C:T	-	Intergenic	rs17843621	C>T	10	7.76x10-20	0.57 [0.51-0.64]	0.006	61.1
6	32653075	chr6:32653075:G:A	-	Intergenic	rs17612907	G>A	10	7.17x10-20	0.57 [0.51-0.64]	0.006	61.04
6	32653083	chr6:32653083:C:T	-	Intergenic	rs17612914	C>T	10	7.61x10-20	0.57 [0.51-0.64]	0.006	61.07

6	32653092	chr6:32653092:G:A	-	Intergenic	rs17843623	G>A	10	7.61x10-20	0.57 [0.51-0.64]	0.006	61.07
6	32653108	chr6:32653108:A:T	-	Intergenic	rs17843624	A>T	10	9.18x10-20	0.57 [0.51-0.64]	0.006	61.17
6	32653132	chr6:32653132:A:G	-	Intergenic	rs17843625	A>G	9	1.73x10-15	1.67 [1.48-1.89]	0.002	67.37
6	32653166	chr6:32653166:C:A	-	Intergenic	rs17612921	C>A	10	7.61x10-20	0.57 [0.51-0.64]	0.006	61.07
6	32653666	chr6:32653666:A:G	-	Intergenic	rs28590669	A>G	10	8.47x10-12	0.70 [0.63-0.77]	0.945	0
6	32653706	chr6:32653706:G:A	-	Intergenic	rs28704178	G>A	10	5.66x10-12	0.70 [0.63-0.77]	0.929	0
6	32654373	chr6:32654373:G:A	-	Intergenic	rs35932914	G>A	10	2.61x10-12	0.69 [0.63-0.77]	0.95	0
6	32655190	chr6:32655190:G:A	HLA-DQB1	Intergenic	rs9273311	G>A	10	7.39x10-20	0.57 [0.51-0.64]	0.006	61.04
6	32655213	chr6:32655213:C:T	HLA-DQB1	Intergenic	rs9273315	C>T	10	7.39x10-20	0.57 [0.51-0.64]	0.006	61.04
6	32655240	chr6:32655240:T:C	HLA-DQB1	Intergenic	rs28468461	T>C	7	2.08x10-11	0.54 [0.46-0.65]	0.094	44.51
6	32655295	chr6:32655295:A:G	HLA-DQB1	Intergenic	rs9273320	A>G	10	8.64x10-20	0.57 [0.51-0.64]	0.006	61.29
6	32655319	chr6:32655319:A:G	HLA-DQB1	Intergenic	rs9273321	A>G	10	7.61x10-20	0.57 [0.51-0.64]	0.006	61.07
6	32655325	chr6:32655325:G:C	HLA-DQB1	Intergenic	rs9273322	G>C	10	8.50x10-20	0.57 [0.51-0.64]	0.006	61.22
6	32655379	chr6:32655379:C:T	HLA-DQB1	Intergenic	rs9273324	C>T	10	7.73x10-20	0.57 [0.51-0.64]	0.006	61.08
6	32655442	chr6:32655442:T:C	HLA-DQB1	Intergenic	rs9273326	T>C	10	7.61x10-20	0.57 [0.51-0.64]	0.006	61.07
6	32655456	chr6:32655456:G:A	HLA-DQB1	Intergenic	rs9273328	G>A	10	7.61x10-20	0.57 [0.51-0.64]	0.006	61.07
6	32655465	chr6:32655465:G:A	HLA-DQB1	Intergenic	rs9273329	G>A	10	7.83x10-20	0.57 [0.51-0.64]	0.006	61.1
6	32655500	chr6:32655500:C:T	HLA-DQB1	Intergenic	rs2395533	C>T	10	6.09x10-67	1.88 [1.76-2.01]	0.481	0
6	32655523	chr6:32655523:C:T	HLA-DQB1	Intergenic	rs9273330	C>T	10	7.61x10-20	0.57 [0.51-0.64]	0.006	61.07
6	32655530	chr6:32655530:C:T	HLA-DQB1	Intergenic	rs9273331	C>T	10	7.61x10-20	0.57 [0.51-0.64]	0.006	61.07
6	32655540	chr6:32655540:A:T	HLA-DQB1	Intergenic	rs9273332	A>T	10	7.61x10-20	0.57 [0.51-0.64]	0.006	61.07
6	32655548	chr6:32655548:T:C	HLA-DQB1	Intergenic	rs9273333	T>C	10	7.61x10-20	0.57 [0.51-0.64]	0.006	61.07
6	32656938	chr6:32656938:A:C	HLA-DQB1	Intergenic	rs72852265	A>C	10	1.33x10-19	0.57 [0.51-0.64]	0.005	61.48
6	32657051	chr6:32657051:C:T	HLA-DQB1	Intergenic	rs35512810	C>T	10	7.61x10-20	0.57 [0.51-0.64]	0.006	61.07
6	32657052	chr6:32657052:T:C	HLA-DQB1	Intergenic	rs35983205	T>C	10	7.61x10-20	0.57 [0.51-0.64]	0.006	61.07
6	32657070	chr6:32657070:G:T	HLA-DQB1	Intergenic	rs113663338	G>T	10	7.61x10-20	0.57 [0.51-0.64]	0.006	61.07
6	32658183	chr6:32658183:A:G	HLA-DQB1	Intergenic	rs9273355	A>G	10	7.61x10-20	0.57 [0.51-0.64]	0.006	61.07
6	32658212	chr6:32658212:A:G	HLA-DQB1	Intergenic	rs9273356	A>G	10	7.61x10-20	0.57 [0.51-0.64]	0.006	61.07
6	32658230	chr6:32658230:C:T	HLA-DQB1	Intergenic	rs28442287	C>T	10	2.86x10-12	0.69 [0.63-0.77]	0.95	0
6	32658244	chr6:32658244:G:A	HLA-DQB1	Intergenic	rs6905775	G>A	10	6.09x10-67	1.88 [1.76-2.01]	0.481	0
6	32658263	chr6:32658263:C:T	HLA-DQB1	Intergenic	rs4713570	C>T	10	1.66x10-08	0.82 [0.77-0.88]	0.504	0
6	32658342	chr6:32658342:C:T	HLA-DQB1	Intergenic	rs28584179	C>T	10	2.84x10-12	0.69 [0.63-0.77]	0.95	0
6	32658472	chr6:32658472:T:C	HLA-DQB1	Intergenic	rs6928482	T>C	10	7.90x10-24	0.60 [0.54-0.66]	0.03	51.33
6	32658495	chr6:32658495:C:A	HLA-DQB1	Intergenic	rs9273363	C>A	10	2.29x10-42	1.53 [1.44-1.62]	0.068	43.6
6	32658525	chr6:32658525:T:G	HLA-DQB1	Intergenic	rs9273364	T>G	10	2.29x10-42	1.53 [1.44-1.62]	0.068	43.6
6	32658571	chr6:32658571:C:T	HLA-DQB1	Intergenic	rs28407950	C>T	10	4.51x10-40	0.62 [0.58-0.67]	0.05	46.78
6	32658605	chr6:32658605:G:T	HLA-DQB1	Intergenic	rs9273366	G>T	10	6.09x10-67	1.88 [1.76-2.01]	0.481	0
6	32658620	chr6:32658620:C:T	HLA-DQB1	Intergenic	rs34983241	C>T	10	5.18x10-40	0.62 [0.58-0.67]	0.051	46.68
6	32658626	chr6:32658626:A:G	HLA-DQB1	Intergenic	rs34004019	A>G	10	4.51x10-40	0.62 [0.58-0.67]	0.05	46.78
6	32658655	chr6:32658655:A:T	HLA-DQB1	Intergenic	rs28515515	A>T	10	5.93x10-18	0.61 [0.54-0.68]	0.05	46.85
6	32658657	chr6:32658657:C:T	HLA-DQB1	Intergenic	rs28447611	C>T	10	4.58x10-40	0.62 [0.58-0.67]	0.05	46.78
6	32658661	chr6:32658661:A:T	HLA-DQB1	Intergenic	rs9273367	A>T	10	2.04x10-41	1.52 [1.43-1.61]	0.053	46.18
6	32658674	chr6:32658674:G:A	HLA-DQB1	Intergenic	rs28414666	G>A	10	4.47x10-25	0.68 [0.63-0.73]	0.321	13.28
6	32658698	chr6:32658698:G:A	HLA-DQB1	Intergenic	rs9273368	G>A	10	2.29x10-42	1.53 [1.44-1.62]	0.068	43.6
6	32658715	chr6:32658715:G:A	HLA-DQB1	Intergenic	rs9273370	G>A	10	5.33x10-34	0.69 [0.65-0.73]	0.107	37.76
6	32658760	chr6:32658760:G:T	HLA-DQB1	Intergenic	rs28672722	G>T	10	1.02x10-27	0.67 [0.63-0.72]	0.069	43.38

6	32658813	chr6:32658813:C:G	HLA-DQB1	Intergenic	rs9273372	C>G	10	7.61x10-20	0.57 [0.51-0.64]	0.006	61.07
6	32658824	chr6:32658824:A:G	HLA-DQB1	Intergenic	rs9273373	A>G	10	7.61x10-20	0.57 [0.51-0.64]	0.006	61.07
6	32658837	chr6:32658837:A:G	HLA-DQB1	Intergenic	rs9273374	A>G	10	7.61x10-20	0.57 [0.51-0.64]	0.006	61.07
6	32658973	chr6:32658973:A:G	HLA-DQB1	Intergenic	rs28718232	A>G	10	7.07x10-25	0.68 [0.63-0.73]	0.316	13.78
6	32659198	chr6:32659198:A:C	HLA-DQB1	Intergenic	rs28414125	A>C	10	2.10x10-23	0.62 [0.56-0.68]	0.982	0
6	32659200	chr6:32659200:T:C	HLA-DQB1	Intergenic	rs9273386	T>C	10	3.69x10-18	0.57 [0.50-0.64]	0.001	66.96
6	32659207	chr6:32659207:A:C	HLA-DQB1	Intergenic	rs4713573	A>C	10	6.63x10-10	0.81 [0.76-0.87]	0.182	28.5
6	32666708	chr6:32666708:A:C	HLA-DQB1	Intergenic	rs9274526	A>C	10	1.21x10-26	0.67 [0.62-0.71]	0.573	0
6	32666729	chr6:32666729:C:T	HLA-DQB1	Intergenic	rs28746851	C>T	10	2.02x10-53	1.70 [1.60-1.81]	0.288	16.84
6	32666731	chr6:32666731:C:T	HLA-DQB1	Intergenic	rs9274528	C>T	10	1.21x10-26	0.67 [0.62-0.71]	0.573	0
6	32666737	chr6:32666737:A:G	HLA-DQB1	Intergenic	rs9274529	A>G	10	6.29x10-20	0.57 [0.51-0.64]	0.006	60.9
6	32666768	chr6:32666768:C:A	HLA-DQB1	Intergenic	rs11568831	C>A	10	2.55x10-55	1.73 [1.62-1.84]	0.326	12.69
6	32667145	chr6:32667145:A:G	HLA-DQB1	Intergenic	rs4403294	A>G	10	1.21x10-26	0.67 [0.62-0.71]	0.573	0
6	32667176	chr6:32667176:A:G	HLA-DQB1	Intergenic	rs9274561	A>G	10	8.68x10-22	0.57 [0.51-0.64]	0.014	56.45
6	32667190	chr6:32667190:T:A	HLA-DQB1	Intergenic	rs28746857	T>A	10	3.18x10-55	1.73 [1.62-1.84]	0.325	12.86
6	32667223	chr6:32667223:A:G	HLA-DQB1	Intergenic	rs9274562	A>G	10	8.68x10-22	0.57 [0.51-0.64]	0.014	56.45
6	32667271	chr6:32667271:G:A	HLA-DQB1	Intergenic	rs9274564	G>A	10	8.73x10-22	0.57 [0.51-0.64]	0.014	56.49
6	32667290	chr6:32667290:T:A	HLA-DQB1	Intergenic	rs9274565	T>A	10	5.59x10-20	0.57 [0.51-0.64]	0.006	60.81
6	32667293	chr6:32667293:T:A	HLA-DQB1	Intergenic	rs1812014	T>A	10	1.90x10-22	0.58 [0.53-0.65]	0.024	53.12
6	32667314	chr6:32667314:T:G	HLA-DQB1	Intergenic	rs4311549	T>G	10	1.21x10-26	0.67 [0.62-0.71]	0.573	0
6	32667343	chr6:32667343:G:A	HLA-DQB1	Intergenic	rs28746861	G>A	10	3.18x10-55	1.73 [1.62-1.84]	0.325	12.86
6	32667395	chr6:32667395:C:T	HLA-DQB1	Intergenic	rs28746863	C>T	10	3.18x10-55	1.73 [1.62-1.84]	0.325	12.86
6	32667428	chr6:32667428:C:T	HLA-DQB1	Intergenic	rs9274573	C>T	10	1.47x10-21	0.57 [0.51-0.64]	0.013	56.8
6	32667449	chr6:32667449:T:G	HLA-DQB1	Intergenic	rs9274575	T>G	10	8.68x10-22	0.57 [0.51-0.64]	0.014	56.45
6	32667453	chr6:32667453:G:A	HLA-DQB1	Intergenic	rs28746864	G>A	10	3.18x10-55	1.73 [1.62-1.84]	0.325	12.86
6	32667471	chr6:32667471:A:T	HLA-DQB1	Intergenic	rs28746865	A>T	10	3.18x10-55	1.73 [1.62-1.84]	0.325	12.86
6	32667855	chr6:32667855:A:G	HLA-DQB1	Intergenic	rs4516985	A>G	10	1.08x10-26	0.66 [0.62-0.71]	0.578	0
6	32667936	chr6:32667936:T:C	HLA-DQB1	Intergenic	rs9274607	T>C	10	6.29x10-20	0.57 [0.51-0.64]	0.006	60.9
6	32667966	chr6:32667966:T:C	HLA-DQB1	Intergenic	rs3828787	T>C	10	3.18x10-55	1.73 [1.62-1.84]	0.325	12.86
6	32667972	chr6:32667972:A:G	HLA-DQB1	Intergenic	rs3828788	A>G	10	3.18x10-55	1.73 [1.62-1.84]	0.325	12.86
6	32667977	chr6:32667977:G:T	HLA-DQB1	Intergenic	rs3828789	G>T	10	6.29x10-20	0.57 [0.51-0.64]	0.006	60.9
6	32668032	chr6:32668032:T:C	HLA-DQB1	Intergenic	rs3828790	T>C	10	6.29x10-20	0.57 [0.51-0.64]	0.006	60.9
6	32668036	chr6:32668036:A:G	HLA-DQB1	Intergenic	rs3828791	A>G	10	6.29x10-20	0.57 [0.51-0.64]	0.006	60.9
6	32668051	chr6:32668051:G:C	HLA-DQB1	Intergenic	rs3828792	G>C	10	3.18x10-55	1.73 [1.62-1.84]	0.325	12.86
6	32668069	chr6:32668069:C:G	HLA-DQB1	Intergenic	rs9274614	C>G	10	2.18x10-27	0.66 [0.62-0.71]	0.553	0
6	32668172	chr6:32668172:G:A	HLA-DQB1	Intergenic	rs2647025	G>A	10	7.60x10-54	1.60 [1.51-1.69]	0.304	15.16
6	32668177	chr6:32668177:G:A	HLA-DQB1	Intergenic	rs9274619	G>A	9	6.52x10-66	2.08 [1.92-2.25]	0.952	0
6	32668197	chr6:32668197:A:G	HLA-DQB1	Intergenic	rs3828796	A>G	10	3.18x10-55	1.73 [1.62-1.84]	0.325	12.86
6	32668261	chr6:32668261:A:T	HLA-DQB1	Intergenic	rs3828798	A>T	10	6.29x10-20	0.57 [0.51-0.64]	0.006	60.9
6	32668263	chr6:32668263:T:C	HLA-DQB1	Intergenic	rs9274626	T>C	10	2.18x10-27	0.66 [0.62-0.71]	0.553	0
6	32668285	chr6:32668285:G:C	HLA-DQB1	Intergenic	rs3828799	G>C	10	2.02x10-53	1.70 [1.60-1.81]	0.288	16.84
6	32668286	chr6:32668286:G:A	HLA-DQB1	Intergenic	rs3828800	G>A	10	2.02x10-53	1.70 [1.60-1.81]	0.288	16.84
6	32668294	chr6:32668294:G:T	HLA-DQB1	Intergenic	rs3828801	G>T	10	2.02x10-53	1.70 [1.60-1.81]	0.288	16.84
6	32668343	chr6:32668343:T:C	HLA-DQB1	Intergenic	rs3828805	T>C	10	2.18x10-27	0.66 [0.62-0.71]	0.553	0
6	32668360	chr6:32668360:A:G	HLA-DQB1	Intergenic	rs9274637	A>G	10	2.04x10-27	0.66 [0.62-0.71]	0.551	0
6	32668369	chr6:32668369:T:C	HLA-DQB1	Intergenic	rs9274639	T>C	10	6.12x10-43	0.61 [0.57-0.65]	0.656	0

6	32668377	chr6:32668377:C:G	HLA-DQB1	Intergenic	rs9274640	C>G	10	1.58x10-53	1.70 [1.60-1.82]	0.251	20.91
6	32668395	chr6:32668395:C:T	HLA-DQB1	Intergenic	rs3828809	C>T	10	1.56x10-53	1.70 [1.60-1.82]	0.25	20.99
6	32668440	chr6:32668440:G:A	HLA-DQB1	Intergenic	rs9274649	G>A	10	2.00x10-53	1.70 [1.60-1.81]	0.257	20.17
6	32668503	chr6:32668503:G:T	HLA-DQB1	Intergenic	rs9274654	G>T	10	3.18x10-55	1.73 [1.62-1.84]	0.325	12.86
6	32668505	chr6:32668505:G:A	HLA-DQB1	Intergenic	rs36058358	G>A	7	2.37x10-11	0.54 [0.46-0.65]	0.09	45.22
6	32668512	chr6:32668512:T:C	HLA-DQB1	Intergenic	rs9274655	T>C	10	3.18x10-55	1.73 [1.62-1.84]	0.325	12.86
6	32668574	chr6:32668574:T:C	HLA-DQB1	Intergenic	rs9274656	T>C	10	2.02x10-53	1.70 [1.60-1.81]	0.288	16.84
6	32668602	chr6:32668602:C:G	HLA-DQB1	Intergenic	rs34896996	C>G	5	1.02x10-10	0.40 [0.31-0.53]	0.898	0
6	32668657	chr6:32668657:A:G	HLA-DQB1	Intergenic	rs9274660	A>G	10	6.29x10-20	0.57 [0.51-0.64]	0.006	60.9
6	32668666	chr6:32668666:A:C	HLA-DQB1	Intergenic	rs9274661	A>C	10	3.18x10-55	1.73 [1.62-1.84]	0.325	12.86
6	32668700	chr6:32668700:G:A	HLA-DQB1	Intergenic	rs35571040	G>A	7	2.37x10-11	0.54 [0.46-0.65]	0.09	45.22
6	32668717	chr6:32668717:T:C	HLA-DQB1	Intergenic	rs9274662	T>C	10	3.55x10-14	0.62 [0.56-0.70]	0.008	59.41
6	32668744	chr6:32668744:G:A	HLA-DQB1	Intergenic	rs9274663	G>A	9	3.97x10-36	1.65 [1.53-1.78]	0.083	42.74
6	32668980	chr6:32668980:T:C	HLA-DQB1	Intergenic	rs3134998	T>C	10	2.80x10-14	0.62 [0.56-0.70]	0.009	59.11
6	32669003	chr6:32669003:G:A	HLA-DQB1	Intergenic	rs9274683	G>A	10	3.78x10-24	0.60 [0.55-0.66]	0.036	49.93
6	32669008	chr6:32669008:C:T	HLA-DQB1	Intergenic	rs9274684	C>T	10	4.56x10-24	0.60 [0.55-0.66]	0.034	50.19
6	32669067	chr6:32669067:T:C	HLA-DQB1	Intergenic	rs9274685	T>C	10	4.74x10-24	0.60 [0.55-0.66]	0.035	50.11
6	32669089	chr6:32669089:A:T	HLA-DQB1	Intergenic	rs3134996	A>T	10	2.79x10-14	0.62 [0.56-0.70]	0.009	59.11
6	32669105	chr6:32669105:C:T	HLA-DQB1	Intergenic	rs9274687	C>T	10	6.51x10-24	0.60 [0.55-0.66]	0.034	50.37
6	32669116	chr6:32669116:T:G	HLA-DQB1	Intergenic	rs34094687	T>G	5	1.02x10-10	0.40 [0.31-0.53]	0.898	0
6	32669153	chr6:32669153:T:C	HLA-DQB1	Intergenic	rs9274689	T>C	10	6.51x10-24	0.60 [0.55-0.66]	0.034	50.37
6	32669163	chr6:32669163:A:C	HLA-DQB1	Intergenic	rs9274690	A>C	10	6.51x10-24	0.60 [0.55-0.66]	0.034	50.37
6	32669180	chr6:32669180:T:C	HLA-DQB1	Intergenic	rs9274691	T>C	10	6.89x10-24	0.60 [0.55-0.66]	0.033	50.45
6	32669181	chr6:32669181:T:A	HLA-DQB1	Intergenic	rs9274692	T>A	10	7.32x10-24	0.60 [0.55-0.66]	0.033	50.54
6	32669195	chr6:32669195:C:T	HLA-DQB1	Intergenic	rs9274693	C>T	10	6.51x10-24	0.60 [0.55-0.66]	0.034	50.37
6	32669214	chr6:32669214:A:T	HLA-DQB1	Intergenic	rs1626993	A>T	10	4.68x10-15	1.59 [1.42-1.78]	0.003	63.53
6	32669220	chr6:32669220:C:T	HLA-DQB1	Intergenic	rs9274695	C>T	10	9.98x10-15	1.64 [1.45-1.85]	0.001	68.01
6	32669230	chr6:32669230:G:C	HLA-DQB1	Intergenic	rs9274696	G>C	10	6.82x10-24	0.60 [0.55-0.66]	0.033	50.43
6	32669239	chr6:32669239:A:G	HLA-DQB1	Intergenic	rs9274697	A>G	10	6.51x10-24	0.60 [0.55-0.66]	0.034	50.37
6	32669245	chr6:32669245:C:T	HLA-DQB1	Intergenic	rs34876308	C>T	5	1.02x10-10	0.40 [0.31-0.53]	0.898	0
6	32669275	chr6:32669275:A:G	HLA-DQB1	Intergenic	rs34809098	A>G	5	1.02x10-10	0.40 [0.31-0.53]	0.898	0
6	32669318	chr6:32669318:A:C	HLA-DQB1	Intergenic	rs9274698	A>C	10	5.91x10-24	0.60 [0.55-0.66]	0.034	50.25
6	32669319	chr6:32669319:G:A	HLA-DQB1	Intergenic	rs9274699	G>A	10	6.92x10-54	1.60 [1.51-1.69]	0.299	15.67
6	32669403	chr6:32669403:A:G	HLA-DQB1	Intergenic	rs9274700	A>G	10	6.23x10-54	1.60 [1.51-1.69]	0.302	15.38
6	32669407	chr6:32669407:C:A	HLA-DQB1	Intergenic	rs9274702	C>A	10	9.59x10-24	0.60 [0.54-0.66]	0.032	50.88
6	32669436	chr6:32669436:A:G	HLA-DQB1	Intergenic	rs9274704	A>G	10	5.31x10-54	1.60 [1.51-1.69]	0.313	14.15
6	32669468	chr6:32669468:G:A	HLA-DQB1	Intergenic	rs9274705	G>A	10	8.15x10-24	0.60 [0.55-0.66]	0.033	50.54
6	32669489	chr6:32669489:A:G	HLA-DQB1	Intergenic	rs9274707	A>G	10	9.02x10-24	0.60 [0.55-0.66]	0.032	50.68
6	32669490	chr6:32669490:G:A	HLA-DQB1	Intergenic	rs9274708	G>A	10	8.42x10-24	0.60 [0.55-0.66]	0.033	50.58
6	32669498	chr6:32669498:A:G	HLA-DQB1	Intergenic	rs9274709	A>G	10	1.18x10-53	1.59 [1.51-1.69]	0.326	12.68
6	32669510	chr6:32669510:C:G	HLA-DQB1	Intergenic	rs9274710	C>G	10	8.42x10-24	0.60 [0.55-0.66]	0.033	50.58
6	32669512	chr6:32669512:A:T	HLA-DQB1	Intergenic	rs9274711	A>T	10	1.13x10-53	1.59 [1.51-1.69]	0.323	13
6	32669521	chr6:32669521:C:T	HLA-DQB1	Intergenic	rs9274712	C>T	10	9.97x10-48	1.54 [1.46-1.63]	0.342	10.92
6	32669564	chr6:32669564:C:T	HLA-DQB1	Intergenic	rs9274713	C>T	10	8.87x10-22	0.61 [0.56-0.68]	0.035	50.01
6	32669614	chr6:32669614:G:A	HLA-DQB1	Intergenic	rs9274715	G>A	10	9.60x10-24	0.60 [0.55-0.66]	0.033	50.67
6	32669627	chr6:32669627:G:A	HLA-DQB1	Intergenic	rs9274716	G>A	10	4.94x10-54	1.60 [1.51-1.69]	0.304	15.11

6	32669677	chr6:32669677:C:A	HLA-DQB1	Intergenic	rs9274717	C>A	10	8.38x10-24	0.60 [0.55-0.66]	0.033	50.65
6	32669693	chr6:32669693:T:C	HLA-DQB1	Intergenic	rs9274719	T>C	10	8.18x10-24	0.60 [0.55-0.66]	0.033	50.56
6	32669702	chr6:32669702:G:A	HLA-DQB1	Intergenic	rs9274720	G>A	10	7.27x10-24	0.60 [0.55-0.66]	0.034	50.38
6	32669720	chr6:32669720:T:C	HLA-DQB1	Intergenic	rs3997832	T>C	10	2.74x10-56	1.59 [1.51-1.68]	0.151	32.15
6	32669750	chr6:32669750:T:A	HLA-DQB1	Intergenic	rs9274722	T>A	10	1.11x10-23	0.60 [0.54-0.66]	0.031	50.96
6	32669754	chr6:32669754:A:G	HLA-DQB1	Intergenic	rs9274724	A>G	10	3.00x10-24	0.60 [0.55-0.66]	0.038	49.32
6	32669796	chr6:32669796:C:T	HLA-DQB1	Intergenic	rs9274725	C>T	10	4.05x10-24	0.60 [0.54-0.66]	0.033	50.43
6	32669816	chr6:32669816:C:G	HLA-DQB1	Intergenic	rs9274726	C>G	10	8.19x10-24	0.60 [0.55-0.66]	0.033	50.55
6	32669903	chr6:32669903:T:C	HLA-DQB1	Intergenic	rs3134994	T>C	10	9.13x10-28	0.66 [0.61-0.71]	0.551	0
6	32669919	chr6:32669919:T:G	HLA-DQB1	Intergenic	rs9274727	T>G	10	6.62x10-53	1.58 [1.50-1.68]	0.363	8.65
6	32669995	chr6:32669995:A:G	HLA-DQB1	Intergenic	rs9274728	A>G	10	7.27x10-54	1.60 [1.51-1.69]	0.302	15.35
6	32670001	chr6:32670001:G:A	HLA-DQB1	Intergenic	rs3134993	G>A	10	1.06x10-21	0.57 [0.51-0.64]	0.014	56.62
6	32670040	chr6:32670040:G:A	HLA-DQB1	Intergenic	rs9274730	G>A	10	8.19x10-24	0.60 [0.55-0.66]	0.033	50.55
6	32670046	chr6:32670046:A:G	HLA-DQB1	Intergenic	rs9274731	A>G	10	8.31x10-24	0.60 [0.55-0.66]	0.033	50.58
6	32670090	chr6:32670090:G:A	HLA-DQB1	Intergenic	rs35788076	G>A	5	1.02x10-10	0.40 [0.31-0.53]	0.898	0
6	32670097	chr6:32670097:C:T	HLA-DQB1	Intergenic	rs9274733	C>T	10	5.60x10-22	0.61 [0.56-0.68]	0.036	49.71
6	32670110	chr6:32670110:T:C	HLA-DQB1	Intergenic	rs9274734	T>C	10	8.19x10-24	0.60 [0.55-0.66]	0.033	50.55
6	32670119	chr6:32670119:G:A	HLA-DQB1	Intergenic	rs9274735	G>A	10	7.27x10-54	1.60 [1.51-1.69]	0.302	15.35
6	32670128	chr6:32670128:T:C	HLA-DQB1	Intergenic	rs9274736	T>C	10	8.19x10-24	0.60 [0.55-0.66]	0.033	50.55
6	32670130	chr6:32670130:T:C	HLA-DQB1	Intergenic	rs9274737	T>C	10	7.27x10-54	1.60 [1.51-1.69]	0.302	15.35
6	32670132	chr6:32670132:A:C	HLA-DQB1	Intergenic	rs9274738	A>C	10	8.19x10-24	0.60 [0.55-0.66]	0.033	50.55
6	32670163	chr6:32670163:A:T	HLA-DQB1	Intergenic	rs9274739	A>T	10	7.27x10-54	1.60 [1.51-1.69]	0.302	15.35
6	32670191	chr6:32670191:A:T	HLA-DQB1	Intergenic	rs9274740	A>T	10	8.19x10-24	0.60 [0.55-0.66]	0.033	50.55
6	32670217	chr6:32670217:T:C	HLA-DQB1	Intergenic	rs9274741	T>C	10	5.62x10-13	0.66 [0.59-0.74]	0.006	61.04
6	32670260	chr6:32670260:T:C	HLA-DQB1	Intergenic	rs9274742	T>C	9	2.59x10-46	1.58 [1.49-1.68]	0.76	0
6	32670265	chr6:32670265:A:G	HLA-DQB1	Intergenic	rs9274743	A>G	10	8.19x10-24	0.60 [0.55-0.66]	0.033	50.55
6	32670277	chr6:32670277:G:A	HLA-DQB1	Intergenic	rs9274744	G>A	10	1.42x10-23	0.60 [0.55-0.66]	0.031	51.01
6	32670281	chr6:32670281:C:T	HLA-DQB1	Intergenic	rs9274745	C>T	10	5.54x10-13	0.66 [0.59-0.74]	0.006	61.04
6	32670283	chr6:32670283:G:A	HLA-DQB1	Intergenic	rs9274746	G>A	10	7.62x10-24	0.60 [0.55-0.66]	0.033	50.51
6	32670294	chr6:32670294:C:A	HLA-DQB1	Intergenic	rs9274747	C>A	9	6.52x10-66	2.08 [1.92-2.25]	0.952	0
6	32673848	chr6:32673848:C:T	-	Intergenic	rs9274936	C>T	9	4.48x10-46	1.58 [1.49-1.67]	0.755	0
6	32673854	chr6:32673854:G:A	-	Intergenic	rs9274937	G>A	10	7.15x10-22	0.61 [0.56-0.68]	0.036	49.77
6	32674500	chr6:32674500:T:C	-	Intergenic	rs9274978	T>C	10	2.86x10-22	0.62 [0.56-0.68]	0.043	48.3
6	32674513	chr6:32674513:G:T	-	Intergenic	rs9274980	G>T	10	4.65x10-22	0.61 [0.56-0.68]	0.037	49.48
6	32675062	chr6:32675062:C:G	-	Intergenic	rs9275004	C>G	9	2.59x10-46	1.58 [1.49-1.68]	0.76	0
6	32675068	chr6:32675068:A:G	-	Intergenic	rs1694122	A>G	9	5.89x10-53	1.65 [1.55-1.75]	0.33	12.56
6	32675075	chr6:32675075:C:T	-	Intergenic	rs9275006	C>T	9	2.59x10-46	1.58 [1.49-1.68]	0.76	0
6	32680376	chr6:32680376:T:C	-	Intergenic	rs9275051	T>C	10	5.93x10-22	0.61 [0.56-0.68]	0.036	49.8
6	32680379	chr6:32680379:G:A	-	Intergenic	rs9275052	G>A	10	5.07x10-33	0.69 [0.65-0.73]	0.13	34.77
6	32680431	chr6:32680431:A:G	-	Intergenic	rs9275054	A>G	10	6.04x10-22	0.61 [0.56-0.68]	0.036	49.86
6	32680660	chr6:32680660:A:G	-	Intergenic	rs9275069	A>G	10	5.60x10-22	0.61 [0.56-0.68]	0.036	49.71
6	32680672	chr6:32680672:C:T	-	Intergenic	rs9275070	C>T	10	5.60x10-22	0.61 [0.56-0.68]	0.036	49.71
6	32680713	chr6:32680713:G:A	-	Intergenic	rs9275071	G>A	10	5.60x10-22	0.61 [0.56-0.68]	0.036	49.71
6	32680723	chr6:32680723:G:T	-	Intergenic	rs3129781	G>T	8	2.98x10-14	0.65 [0.58-0.72]	0.923	0
6	32680725	chr6:32680725:C:T	-	Intergenic	rs9275072	C>T	9	2.59x10-46	1.58 [1.49-1.68]	0.76	0
6	32680736	chr6:32680736:C:G	-	Intergenic	rs9275073	C>G	10	3.73x10-22	0.61 [0.56-0.68]	0.038	49.43

6	32680745	chr6:32680745:T:C	-	Intergenic	rs9275074	T>C	9	2.59x10-46	1.58 [1.49-1.68]	0.76	0
6	32680773	chr6:32680773:G:A	-	Intergenic	rs9275075	G>A	9	2.59x10-46	1.58 [1.49-1.68]	0.76	0
6	32680776	chr6:32680776:C:G	-	Intergenic	rs9275076	C>G	9	2.59x10-46	1.58 [1.49-1.68]	0.76	0
6	32680788	chr6:32680788:T:C	-	Intergenic	rs9275077	T>C	9	2.59x10-46	1.58 [1.49-1.68]	0.76	0
6	32680815	chr6:32680815:A:C	-	Intergenic	rs9275078	A>C	10	4.76x10-22	0.61 [0.56-0.68]	0.038	49.46
6	32680837	chr6:32680837:A:C	-	Intergenic	rs9275079	A>C	9	3.35x10-46	1.58 [1.49-1.67]	0.762	0
6	32680850	chr6:32680850:C:T	-	Intergenic	rs9275080	C>T	9	3.35x10-46	1.58 [1.49-1.67]	0.762	0
6	32680892	chr6:32680892:C:T	-	Intergenic	rs9275081	C>T	10	4.76x10-22	0.61 [0.56-0.68]	0.038	49.46
6	32680907	chr6:32680907:A:C	-	Intergenic	rs9275082	A>C	10	4.76x10-22	0.61 [0.56-0.68]	0.038	49.46
6	32680917	chr6:32680917:T:C	-	Intergenic	rs9275083	T>C	9	1.94x10-46	1.58 [1.49-1.68]	0.771	0
6	32680942	chr6:32680942:G:A	-	Intergenic	rs3129782	G>A	10	2.10x10-27	0.66 [0.62-0.71]	0.551	0
6	32680945	chr6:32680945:A:G	-	Intergenic	rs9275084	A>G	10	4.76x10-22	0.61 [0.56-0.68]	0.038	49.46
6	32680958	chr6:32680958:G:A	-	Intergenic	rs9275085	G>A	10	4.76x10-22	0.61 [0.56-0.68]	0.038	49.46
6	32681032	chr6:32681032:A:G	-	Intergenic	rs9275086	A>G	10	4.76x10-22	0.61 [0.56-0.68]	0.038	49.46
6	32681116	chr6:32681116:T:C	-	Intergenic	rs9275087	T>C	10	4.69x10-22	0.61 [0.56-0.68]	0.038	49.44
6	32681154	chr6:32681154:T:A	-	Intergenic	rs9275088	T>A	10	4.33x10-22	0.61 [0.56-0.68]	0.038	49.37
6	32681170	chr6:32681170:A:T	-	Intergenic	rs9275089	A>T	10	2.29x10-22	0.62 [0.56-0.68]	0.042	48.48
6	32681194	chr6:32681194:T:G	-	Intergenic	rs9275090	T>G	9	1.94x10-46	1.58 [1.49-1.68]	0.771	0
6	32681195	chr6:32681195:C:T	-	Intergenic	rs9275091	C>T	10	4.76x10-22	0.61 [0.56-0.68]	0.038	49.46
6	32681210	chr6:32681210:C:T	-	Intergenic	rs9275092	C>T	9	6.52x10-66	2.08 [1.92-2.25]	0.952	0
6	32681270	chr6:32681270:C:T	-	Intergenic	rs9275093	C>T	9	1.94x10-46	1.58 [1.49-1.68]	0.771	0
6	32681285	chr6:32681285:T:C	-	Intergenic	rs9275094	T>C	9	1.94x10-46	1.58 [1.49-1.68]	0.771	0
6	32681311	chr6:32681311:C:G	-	Intergenic	rs9275095	C>G	9	6.52x10-66	2.08 [1.92-2.25]	0.952	0
6	32681319	chr6:32681319:C:T	-	Intergenic	rs9275096	C>T	9	1.94x10-46	1.58 [1.49-1.68]	0.771	0
6	32681349	chr6:32681349:A:G	-	Intergenic	rs9275097	A>G	9	6.52x10-66	2.08 [1.92-2.25]	0.952	0
6	32681384	chr6:32681384:C:T	-	Intergenic	rs9275098	C>T	9	6.52x10-66	2.08 [1.92-2.25]	0.952	0
6	32681420	chr6:32681420:C:T	-	Intergenic	rs1794496	C>T	9	1.75x10-51	1.64 [1.54-1.74]	0.444	0
6	32681531	chr6:32681531:A:G	-	Intergenic	rs9275100	A>G	9	1.94x10-46	1.58 [1.49-1.68]	0.771	0
6	32681578	chr6:32681578:G:A	-	Intergenic	rs9275101	G>A	10	4.76x10-22	0.61 [0.56-0.68]	0.038	49.46
6	32681584	chr6:32681584:G:A	-	Intergenic	rs9275102	G>A	10	4.76x10-22	0.61 [0.56-0.68]	0.038	49.46
6	32681609	chr6:32681609:T:C	-	Intergenic	rs9275103	T>C	10	4.76x10-22	0.61 [0.56-0.68]	0.038	49.46
6	32681611	chr6:32681611:T:C	-	Intergenic	rs9275104	T>C	9	1.86x10-46	1.58 [1.49-1.68]	0.771	0
6	32681639	chr6:32681639:C:T	-	Intergenic	rs9275105	C>T	10	4.76x10-22	0.61 [0.56-0.68]	0.038	49.46
6	32681698	chr6:32681698:T:C	-	Intergenic	rs9275106	T>C	10	4.76x10-22	0.61 [0.56-0.68]	0.038	49.46
6	32681731	chr6:32681731:A:G	-	Intergenic	rs9275107	A>G	9	1.94x10-46	1.58 [1.49-1.68]	0.771	0
6	32681832	chr6:32681832:G:A	-	Intergenic	rs9275108	G>A	9	1.94x10-46	1.58 [1.49-1.68]	0.771	0
6	32681899	chr6:32681899:G:T	-	Intergenic	rs9275109	G>T	10	1.34x10-08	0.74 [0.67-0.82]	0.048	47.12
6	32681946	chr6:32681946:T:C	-	Intergenic	rs9275110	T>C	9	1.94x10-46	1.58 [1.49-1.68]	0.771	0
6	32681958	chr6:32681958:G:T	-	Intergenic	rs9275111	G>T	9	1.94x10-46	1.58 [1.49-1.68]	0.771	0
6	32681996	chr6:32681996:G:A	-	Intergenic	rs9275112	G>A	10	5.06x10-22	0.62 [0.56-0.68]	0.038	49.41
6	32682048	chr6:32682048:A:G	-	Intergenic	rs9275113	A>G	9	1.94x10-46	1.58 [1.49-1.68]	0.771	0
6	32682120	chr6:32682120:G:A	-	Intergenic	rs9275114	G>A	10	4.75x10-22	0.61 [0.56-0.68]	0.038	49.45
6	32682164	chr6:32682164:T:C	-	Intergenic	rs9275115	T>C	9	1.94x10-46	1.58 [1.49-1.68]	0.771	0
6	32682175	chr6:32682175:T:C	-	Intergenic	rs9275116	T>C	10	4.75x10-22	0.61 [0.56-0.68]	0.038	49.45
6	32682188	chr6:32682188:C:T	-	Intergenic	rs9275117	C>T	9	1.72x10-46	1.58 [1.49-1.68]	0.77	0
6	32682219	chr6:32682219:A:G	-	Intergenic	rs9275118	A>G	10	4.68x10-22	0.61 [0.56-0.68]	0.038	49.43

6	32682254	chr6:32682254:G:A	-	Intergenic	rs9275119	G>A	10	4.75x10-22	0.61 [0.56-0.68]	0.038	49.45
6	32682261	chr6:32682261:C:T	-	Intergenic	rs115239177	C>T	5	8.44x10-11	0.40 [0.31-0.53]	0.898	0
6	32682401	chr6:32682401:A:T	-	Intergenic	rs3134979	A>T	10	9.66x10-28	0.66 [0.61-0.71]	0.552	0
6	32682408	chr6:32682408:A:G	-	Intergenic	rs9275121	A>G	9	1.94x10-46	1.58 [1.49-1.68]	0.771	0
6	32682456	chr6:32682456:T:C	-	Intergenic	rs9275122	T>C	9	1.94x10-46	1.58 [1.49-1.68]	0.771	0
6	32682479	chr6:32682479:G:T	-	Intergenic	rs9275123	G>T	10	4.68x10-22	0.61 [0.56-0.68]	0.038	49.42
6	32682491	chr6:32682491:T:C	-	Intergenic	rs2516006	T>C	9	5.27x10-53	1.65 [1.55-1.76]	0.345	10.77
6	32682516	chr6:32682516:A:T	-	Intergenic	rs9275125	A>T	10	4.75x10-22	0.61 [0.56-0.68]	0.038	49.45
6	32682530	chr6:32682530:A:T	-	Intergenic	rs9275126	A>T	10	1.70x10-22	0.61 [0.56-0.68]	0.042	48.5
6	32682537	chr6:32682537:G:T	-	Intergenic	rs9275127	G>T	9	1.94x10-46	1.58 [1.49-1.68]	0.771	0
6	32682610	chr6:32682610:A:G	-	Intergenic	rs115727536	A>G	5	8.44x10-11	0.40 [0.31-0.53]	0.898	0
6	32682627	chr6:32682627:C:T	-	Intergenic	rs9275128	C>T	9	1.89x10-46	1.58 [1.49-1.68]	0.767	0
6	32682628	chr6:32682628:T:C	-	Intergenic	rs9275129	T>C	9	1.89x10-46	1.58 [1.49-1.68]	0.767	0
6	32682632	chr6:32682632:T:C	-	Intergenic	rs9275130	T>C	10	4.41x10-22	0.61 [0.56-0.68]	0.038	49.34
6	32682690	chr6:32682690:C:T	-	Intergenic	rs9275131	C>T	10	5.60x10-22	0.61 [0.56-0.68]	0.036	49.7
6	32682723	chr6:32682723:G:A	-	Intergenic	rs9275132	G>A	9	2.67x10-46	1.58 [1.49-1.68]	0.78	0
6	32682811	chr6:32682811:A:G	-	Intergenic	rs9275133	A>G	10	3.99x10-22	0.61 [0.56-0.68]	0.039	49.18
6	32682835	chr6:32682835:C:G	-	Intergenic	rs9275134	C>G	9	3.13x10-46	1.58 [1.49-1.68]	0.775	0
6	32682928	chr6:32682928:T:C	-	Intergenic	rs115420259	T>C	5	1.06x10-10	0.41 [0.31-0.53]	0.904	0
6	32682959	chr6:32682959:T:C	-	Intergenic	rs9275135	T>C	10	3.99x10-22	0.61 [0.56-0.68]	0.039	49.18
6	32682980	chr6:32682980:C:T	-	Intergenic	rs9275136	C>T	10	3.99x10-22	0.61 [0.56-0.68]	0.039	49.18
6	32683103	chr6:32683103:G:C	-	Intergenic	rs9275137	G>C	9	3.40x10-46	1.58 [1.49-1.67]	0.776	0
6	32683181	chr6:32683181:A:G	-	Intergenic	rs9275138	A>G	10	3.99x10-22	0.61 [0.56-0.68]	0.039	49.18
6	32683187	chr6:32683187:T:C	-	Intergenic	rs35534346	T>C	5	1.06x10-10	0.41 [0.31-0.53]	0.904	0
6	32683228	chr6:32683228:C:A	-	Intergenic	rs9275139	C>A	10	3.99x10-22	0.61 [0.56-0.68]	0.039	49.18
6	32683241	chr6:32683241:G:A	-	Intergenic	rs9275140	G>A	9	3.13x10-46	1.58 [1.49-1.68]	0.775	0
6	32683415	chr6:32683415:A:T	-	Intergenic	rs9275143	A>T	10	5.69x10-22	0.62 [0.56-0.68]	0.038	49.42
6	32683441	chr6:32683441:T:A	-	Intergenic	rs9275144	T>A	9	3.13x10-46	1.58 [1.49-1.68]	0.775	0
6	32683513	chr6:32683513:C:A	-	Intergenic	rs114774621	C>A	5	1.06x10-10	0.41 [0.31-0.53]	0.904	0
6	32683586	chr6:32683586:C:T	-	Intergenic	rs9275145	C>T	10	3.93x10-22	0.62 [0.56-0.68]	0.039	49.15
6	32683718	chr6:32683718:G:T	-	Intergenic	rs9275146	G>T	10	3.99x10-22	0.61 [0.56-0.68]	0.039	49.18
6	32683780	chr6:32683780:C:T	-	Intergenic	rs9275147	C>T	9	3.13x10-46	1.58 [1.49-1.68]	0.775	0
6	32683808	chr6:32683808:T:C	-	Intergenic	rs9275148	T>C	10	3.99x10-22	0.61 [0.56-0.68]	0.039	49.18
6	32683839	chr6:32683839:C:T	-	Intergenic	rs9275149	C>T	9	3.13x10-46	1.58 [1.49-1.68]	0.775	0
6	32683840	chr6:32683840:A:G	-	Intergenic	rs9275150	A>G	9	3.13x10-46	1.58 [1.49-1.68]	0.775	0
6	32683930	chr6:32683930:C:G	-	Intergenic	rs7750477	C>G	9	3.13x10-46	1.58 [1.49-1.68]	0.775	0
6	32683964	chr6:32683964:C:T	-	Intergenic	rs3021062	C>T	9	3.13x10-46	1.58 [1.49-1.68]	0.775	0
6	32684062	chr6:32684062:C:G	-	Intergenic	rs3021061	C>G	9	3.13x10-46	1.58 [1.49-1.68]	0.775	0
6	32684069	chr6:32684069:G:C	-	Intergenic	rs3021060	G>C	9	3.13x10-46	1.58 [1.49-1.68]	0.775	0
6	32684117	chr6:32684117:A:G	-	Intergenic	rs2856695	A>G	10	3.86x10-22	0.61 [0.56-0.68]	0.039	49.17
6	32684159	chr6:32684159:C:T	-	Intergenic	rs9275151	C>T	9	1.17x10-65	2.07 [1.92-2.24]	0.953	0
6	32684419	chr6:32684419:T:C	-	Intergenic	rs9275152	T>C	9	3.74x10-68	2.14 [1.98-2.32]	0.924	0
6	32684528	chr6:32684528:A:C	-	Intergenic	rs3134976	A>C	10	1.26x10-27	0.66 [0.61-0.71]	0.549	0
6	32684567	chr6:32684567:C:T	-	Intergenic	rs3021059	C>T	9	3.13x10-46	1.58 [1.49-1.68]	0.775	0
6	32684582	chr6:32684582:A:C	-	Intergenic	rs3021058	A>C	10	4.29x10-22	0.61 [0.56-0.68]	0.038	49.3
6	32684586	chr6:32684586:C:T	-	Intergenic	rs3021057	C>T	10	4.29x10-22	0.61 [0.56-0.68]	0.038	49.3

6	32684658	chr6:32684658:C:T	-	Intergenic	rs9275154	C>T	10	3.99x10-22	0.61 [0.56-0.68]	0.039	49.18
6	32684707	chr6:32684707:T:A	-	Intergenic	rs9275155	T>A	10	1.71x10-22	0.61 [0.56-0.68]	0.042	48.41
6	32684709	chr6:32684709:T:C	-	Intergenic	rs9275156	T>C	9	3.13x10-46	1.58 [1.49-1.68]	0.775	0
6	32684775	chr6:32684775:G:A	-	Intergenic	rs9275157	G>A	9	2.46x10-46	1.58 [1.49-1.68]	0.773	0
6	32684804	chr6:32684804:T:C	-	Intergenic	rs3134975	T>C	10	2.99x10-27	0.66 [0.62-0.71]	0.55	0
6	32684805	chr6:32684805:G:A	-	Intergenic	rs9275158	G>A	10	3.99x10-22	0.61 [0.56-0.68]	0.039	49.18
6	32684830	chr6:32684830:C:T	-	Intergenic	rs9275159	C>T	9	3.13x10-46	1.58 [1.49-1.68]	0.775	0
6	32684843	chr6:32684843:G:A	-	Intergenic	rs9275160	G>A	10	7.05x10-13	1.59 [1.40-1.79]	0.001	68.86
6	32684859	chr6:32684859:T:C	-	Intergenic	rs9275161	T>C	10	4.05x10-22	0.61 [0.56-0.68]	0.039	49.22
6	32684877	chr6:32684877:T:C	-	Intergenic	rs35507670	T>C	5	1.06x10-10	0.41 [0.31-0.53]	0.904	0
6	32684910	chr6:32684910:T:C	-	Intergenic	rs9275162	T>C	10	3.99x10-22	0.61 [0.56-0.68]	0.039	49.18
6	32685102	chr6:32685102:C:T	-	Intergenic	rs116565195	C>T	5	1.06x10-10	0.41 [0.31-0.53]	0.904	0
6	32685103	chr6:32685103:A:C	-	Intergenic	rs9275163	A>C	9	1.17x10-65	2.07 [1.92-2.24]	0.953	0
6	32685152	chr6:32685152:T:C	-	Intergenic	rs9275164	T>C	9	3.13x10-46	1.58 [1.49-1.68]	0.775	0
6	32685179	chr6:32685179:A:G	-	Intergenic	rs9275165	A>G	9	3.13x10-46	1.58 [1.49-1.68]	0.775	0
6	32685186	chr6:32685186:T:C	-	Intergenic	rs9275166	T>C	9	3.13x10-46	1.58 [1.49-1.68]	0.775	0
6	32685293	chr6:32685293:G:A	-	Intergenic	rs4947342	G>A	9	3.13x10-46	1.58 [1.49-1.68]	0.775	0
6	32685486	chr6:32685486:A:G	-	Intergenic	rs9275167	A>G	9	1.17x10-65	2.07 [1.92-2.24]	0.953	0
6	32685506	chr6:32685506:C:T	-	Intergenic	rs2856693	C>T	10	3.91x10-22	0.62 [0.56-0.68]	0.039	49.13
6	32685608	chr6:32685608:T:G	-	Intergenic	rs2856692	T>G	9	3.13x10-46	1.58 [1.49-1.68]	0.775	0
6	32685658	chr6:32685658:G:A	-	Intergenic	rs2856691	G>A	9	2.94x10-46	1.58 [1.49-1.68]	0.778	0
6	32685899	chr6:32685899:T:C	-	Intergenic	rs9275168	T>C	10	9.35x10-13	0.66 [0.59-0.74]	0.005	61.6
6	32685944	chr6:32685944:T:A	-	Intergenic	rs9275169	T>A	10	9.35x10-13	0.66 [0.59-0.74]	0.005	61.6
6	32685949	chr6:32685949:G:A	-	Intergenic	rs9275170	G>A	10	9.42x10-13	0.66 [0.59-0.74]	0.005	61.63
6	32686015	chr6:32686015:A:G	-	Intergenic	rs34100078	A>G	8	4.38x10-10	0.61 [0.53-0.71]	0.191	29.73
6	32686195	chr6:32686195:A:G	-	Intergenic	rs9275171	A>G	9	1.05x10-29	1.42 [1.34-1.51]	0.308	15.11
6	32686220	chr6:32686220:A:G	-	Intergenic	rs9275172	A>G	10	9.49x10-13	0.66 [0.59-0.74]	0.005	61.64
6	32686235	chr6:32686235:A:C	-	Intergenic	rs191225457	A>C	5	1.06x10-10	0.41 [0.31-0.53]	0.904	0
6	32686260	chr6:32686260:T:A	-	Intergenic	rs9275173	T>A	10	9.35x10-13	0.66 [0.59-0.74]	0.005	61.6
6	32686370	chr6:32686370:G:A	-	Intergenic	rs9275175	G>A	9	1.05x10-29	1.42 [1.34-1.51]	0.308	15.11
6	32686397	chr6:32686397:C:T	-	Intergenic	rs9275177	C>T	10	1.11x10-12	0.66 [0.59-0.74]	0.005	62
6	32686404	chr6:32686404:G:A	-	Intergenic	rs9275178	G>A	10	9.55x10-13	0.66 [0.59-0.74]	0.005	61.66
6	32686498	chr6:32686498:A:G	-	Intergenic	rs9275179	A>G	9	3.13x10-46	1.58 [1.49-1.68]	0.775	0
6	32686501	chr6:32686501:C:T	-	Intergenic	rs9275180	C>T	10	4.91x10-22	0.61 [0.56-0.68]	0.037	49.55
6	32686548	chr6:32686548:A:G	-	Intergenic	rs9275181	A>G	9	3.13x10-46	1.58 [1.49-1.68]	0.775	0
6	32686613	chr6:32686613:C:T	-	Intergenic	rs9275182	C>T	9	3.13x10-46	1.58 [1.49-1.68]	0.775	0
6	32686725	chr6:32686725:A:G	-	Intergenic	rs9275183	A>G	9	1.57x10-51	1.82 [1.69-1.96]	0.812	0
6	32686759	chr6:32686759:T:A	-	Intergenic	rs2856690	T>A	10	4.24x10-22	0.61 [0.56-0.68]	0.038	49.31
6	32686774	chr6:32686774:A:G	-	Intergenic	rs2856689	A>G	10	1.77x10-21	0.62 [0.56-0.68]	0.033	50.59
6	32686863	chr6:32686863:G:T	-	Intergenic	rs2856688	G>T	9	3.13x10-46	1.58 [1.49-1.68]	0.775	0
6	32686937	chr6:32686937:T:C	-	Intergenic	rs9275184	T>C	9	1.17x10-65	2.07 [1.92-2.24]	0.953	0
6	32687045	chr6:32687045:T:C	-	Intergenic	rs2856687	T>C	10	1.61x10-34	0.68 [0.64-0.72]	0.297	15.88
6	32687092	chr6:32687092:T:C	-	Intergenic	rs2856686	T>C	10	3.99x10-22	0.61 [0.56-0.68]	0.039	49.18
6	32687295	chr6:32687295:A:T	-	Intergenic	rs2856685	A>T	10	3.99x10-22	0.61 [0.56-0.68]	0.039	49.18
6	32687318	chr6:32687318:T:C	-	Intergenic	rs2856684	T>C	10	4.04x10-22	0.61 [0.56-0.68]	0.039	49.22
6	32687441	chr6:32687441:T:G	-	Intergenic	rs2856683	T>G	9	3.39x10-46	1.58 [1.49-1.67]	0.777	0

6	32687528	chr6:32687528:G:T	-	Intergenic	rs9275185	G>T	9	1.17x10-65	2.07 [1.92-2.24]	0.953	0
6	32687838	chr6:32687838:C:A	-	Intergenic	rs2856679	C>A	10	1.14x10-22	0.62 [0.56-0.68]	0.046	47.55
6	32687953	chr6:32687953:A:G	-	Intergenic	rs3129783	A>G	10	2.57x10-09	0.84 [0.80-0.89]	0.965	0
6	32688185	chr6:32688185:T:G	-	Intergenic	rs2935909	T>G	9	3.52x10-46	1.58 [1.49-1.67]	0.702	0
6	32688831	chr6:32688831:G:T	-	Intergenic	rs3129784	G>T	10	3.97x10-22	0.61 [0.56-0.68]	0.039	49.19
6	32688903	chr6:32688903:C:T	-	Intergenic	rs9275199	C>T	10	3.99x10-22	0.61 [0.56-0.68]	0.039	49.18
6	32688914	chr6:32688914:C:T	-	Intergenic	rs116516516	C>T	5	1.06x10-10	0.41 [0.31-0.53]	0.904	0
6	32688920	chr6:32688920:T:G	-	Intergenic	rs3104362	T>G	9	3.13x10-46	1.58 [1.49-1.68]	0.775	0
6	32688945	chr6:32688945:C:T	-	Intergenic	rs149270899	C>T	5	1.06x10-10	0.41 [0.31-0.53]	0.904	0
6	32688946	chr6:32688946:G:A	-	Intergenic	rs9275201	G>A	9	1.58x10-65	2.07 [1.91-2.24]	0.952	0
6	32688947	chr6:32688947:C:T	-	Intergenic	rs3129785	C>T	9	3.13x10-46	1.58 [1.49-1.68]	0.775	0
6	32689038	chr6:32689038:C:T	-	Intergenic	rs2856677	C>T	9	3.13x10-46	1.58 [1.49-1.68]	0.775	0
6	32689056	chr6:32689056:G:A	-	Intergenic	rs2856676	G>A	10	3.27x10-22	0.61 [0.56-0.68]	0.039	49.07
6	32689092	chr6:32689092:A:G	-	Intergenic	rs9275202	A>G	10	3.99x10-22	0.61 [0.56-0.68]	0.039	49.18
6	32689113	chr6:32689113:T:C	-	Intergenic	rs2647000	T>C	10	3.99x10-22	0.61 [0.56-0.68]	0.039	49.18
6	32689170	chr6:32689170:G:A	-	Intergenic	rs9275203	G>A	9	1.17x10-65	2.07 [1.92-2.24]	0.953	0
6	32689423	chr6:32689423:T:A	-	Intergenic	rs3129712	T>A	9	3.13x10-46	1.58 [1.49-1.68]	0.775	0
6	32689766	chr6:32689766:G:T	-	Intergenic	rs4642516	G>T	10	3.83x10-22	0.62 [0.56-0.68]	0.039	49.11
6	32689783	chr6:32689783:T:C	-	Intergenic	rs9275205	T>C	9	6.56x10-53	1.65 [1.55-1.75]	0.348	10.41
6	32689788	chr6:32689788:A:G	-	Intergenic	rs9275206	A>G	9	1.17x10-65	2.07 [1.92-2.24]	0.953	0
6	32689801	chr6:32689801:T:C	-	Intergenic	rs7774434	T>C	9	6.45x10-11	1.21 [1.14-1.28]	0.634	0
6	32689933	chr6:32689933:A:G	-	Intergenic	rs9275207	A>G	9	1.17x10-65	2.07 [1.92-2.24]	0.953	0
6	32689945	chr6:32689945:G:A	-	Intergenic	rs9275208	G>A	10	1.23x10-18	0.60 [0.54-0.67]	0.006	61.19
6	32690040	chr6:32690040:C:T	-	Intergenic	rs9275210	C>T	10	8.88x10-19	0.60 [0.54-0.67]	0.006	60.88
6	32690041	chr6:32690041:A:G	-	Intergenic	rs7754464	A>G	10	3.99x10-22	0.61 [0.56-0.68]	0.039	49.18
6	32690067	chr6:32690067:T:C	-	Intergenic	rs9275211	T>C	9	6.56x10-53	1.65 [1.55-1.75]	0.348	10.41
6	32690081	chr6:32690081:G:A	-	Intergenic	rs7754885	G>A	10	3.99x10-22	0.61 [0.56-0.68]	0.039	49.18
6	32690127	chr6:32690127:G:A	-	Intergenic	rs35130600	G>A	5	1.06x10-10	0.41 [0.31-0.53]	0.904	0
6	32690550	chr6:32690550:G:T	-	Intergenic	rs9275212	G>T	10	3.01x10-34	0.68 [0.64-0.72]	0.299	15.69
6	32690558	chr6:32690558:T:C	-	Intergenic	rs9275213	T>C	9	1.17x10-65	2.07 [1.92-2.24]	0.953	0
6	32690847	chr6:32690847:G:A	-	Intergenic	rs2157051	G>A	10	4.36x10-09	0.83 [0.78-0.88]	0.199	26.61
6	32690888	chr6:32690888:G:A	-	Intergenic	rs9275214	G>A	9	1.17x10-65	2.07 [1.92-2.24]	0.953	0
6	32690900	chr6:32690900:T:C	-	Intergenic	rs9275215	T>C	10	3.44x10-13	1.58 [1.40-1.78]	0.001	67.6
6	32690971	chr6:32690971:A:T	-	Intergenic	rs9275216	A>T	10	2.76x10-13	1.58 [1.40-1.79]	0.001	67.39
6	32691090	chr6:32691090:A:G	-	Intergenic	rs9275217	A>G	10	2.70x10-22	0.75 [0.71-0.79]	0.06	44.87
6	32691134	chr6:32691134:C:T	-	Intergenic	rs2858328	C>T	10	2.88x10-34	0.68 [0.65-0.72]	0.34	11.12
6	32691300	chr6:32691300:C:T	-	Intergenic	rs9275219	C>T	10	1.55x10-14	1.28 [1.20-1.36]	0.459	0
6	32691310	chr6:32691310:T:C	-	Intergenic	rs9275220	T>C	10	1.55x10-14	1.28 [1.20-1.36]	0.459	0
6	32691322	chr6:32691322:T:C	-	Intergenic	rs9275221	T>C	9	1.17x10-65	2.07 [1.92-2.24]	0.953	0
6	32691381	chr6:32691381:G:A	-	Intergenic	rs5002702	G>A	10	2.11x10-22	0.75 [0.71-0.79]	0.061	44.72
6	32691415	chr6:32691415:C:A	-	Intergenic	rs1794523	C>A	5	6.31x10-11	0.40 [0.31-0.53]	0.899	0
6	32691538	chr6:32691538:A:G	-	Intergenic	rs3134967	A>G	10	1.12x10-27	0.66 [0.61-0.71]	0.549	0
6	32691658	chr6:32691658:G:A	-	Intergenic	rs4713589	G>A	10	1.23x10-34	0.68 [0.64-0.72]	0.343	10.8
6	32691739	chr6:32691739:A:T	-	Intergenic	rs9275222	A>T	10	4.70x10-25	1.35 [1.28-1.43]	0.058	45.36
6	32691758	chr6:32691758:G:T	-	Intergenic	rs4713587	G>T	10	2.11x10-22	0.75 [0.71-0.79]	0.061	44.72
6	32691830	chr6:32691830:G:A	-	Intergenic	rs2858326	G>A	5	1.06x10-10	0.41 [0.31-0.53]	0.904	0

6	32691966	chr6:32691966:G:C	-	Intergenic	rs4248168	G>C	10	2.35x10-22	0.75 [0.71-0.79]	0.058	45.26
6	32692061	chr6:32692061:T:C	-	Intergenic	rs3129718	T>C	8	2.43x10-13	0.66 [0.59-0.74]	0.945	0
6	32692062	chr6:32692062:G:A	-	Intergenic	rs9275223	G>A	9	1.17x10-65	2.07 [1.92-2.24]	0.953	0
6	32692101	chr6:32692101:A:G	-	Intergenic	rs9275224	A>G	10	2.35x10-22	0.75 [0.71-0.79]	0.058	45.26
6	32692217	chr6:32692217:C:T	-	Intergenic	rs4713580	C>T	10	1.84x10-22	0.75 [0.71-0.79]	0.06	44.97
6	32692246	chr6:32692246:T:C	-	Intergenic	rs4713581	T>C	10	2.35x10-22	0.75 [0.71-0.79]	0.058	45.26
6	32692274	chr6:32692274:T:C	-	Intergenic	rs4713582	T>C	10	2.03x10-22	0.75 [0.71-0.79]	0.061	44.78
6	32692376	chr6:32692376:T:G	-	Intergenic	rs4713583	T>G	10	2.35x10-22	0.75 [0.71-0.79]	0.058	45.26
6	32692393	chr6:32692393:T:C	-	Intergenic	rs4711304	T>C	10	2.11x10-22	0.75 [0.71-0.79]	0.061	44.72
6	32692460	chr6:32692460:C:T	-	Intergenic	rs4713584	C>T	10	2.35x10-22	0.75 [0.71-0.79]	0.058	45.26
6	32692485	chr6:32692485:G:A	-	Intergenic	rs9275225	G>A	10	2.35x10-22	0.75 [0.71-0.79]	0.058	45.26
6	32692534	chr6:32692534:C:T	-	Intergenic	rs9275226	C>T	10	2.29x10-22	0.75 [0.71-0.79]	0.06	45.03
6	32692560	chr6:32692560:C:G	-	Intergenic	rs9275227	C>G	10	2.35x10-22	0.75 [0.71-0.79]	0.058	45.26
6	32692570	chr6:32692570:G:A	-	Intergenic	rs9275228	G>A	10	2.35x10-22	0.75 [0.71-0.79]	0.058	45.26
6	32692598	chr6:32692598:A:G	-	Intergenic	rs2858324	A>G	10	1.61x10-34	0.68 [0.64-0.72]	0.297	15.88
6	32692665	chr6:32692665:A:G	-	Intergenic	rs9275230	A>G	10	2.22x10-22	0.75 [0.71-0.79]	0.065	44.02
6	32692728	chr6:32692728:T:C	-	Intergenic	rs9275231	T>C	10	2.11x10-22	0.75 [0.71-0.79]	0.061	44.72
6	32692797	chr6:32692797:C:A	-	Intergenic	rs9275232	C>A	10	2.12x10-22	0.75 [0.71-0.79]	0.061	44.72
6	32692805	chr6:32692805:A:C	-	Intergenic	rs2647006	A>C	10	1.61x10-34	0.68 [0.64-0.72]	0.297	15.88
6	32692809	chr6:32692809:C:T	-	Intergenic	rs9275234	C>T	10	2.35x10-22	0.75 [0.71-0.79]	0.058	45.26
6	32692810	chr6:32692810:A:G	-	Intergenic	rs9275235	A>G	10	2.32x10-22	0.75 [0.71-0.79]	0.058	45.26
6	32692824	chr6:32692824:A:G	-	Intergenic	rs9275236	A>G	10	2.35x10-22	0.75 [0.71-0.79]	0.058	45.26
6	32692835	chr6:32692835:C:A	-	Intergenic	rs9275237	C>A	10	2.11x10-22	0.75 [0.71-0.79]	0.061	44.72
6	32692912	chr6:32692912:A:G	-	Intergenic	rs9275238	A>G	10	1.75x10-22	0.75 [0.71-0.79]	0.06	45.02
6	32692940	chr6:32692940:G:A	-	Intergenic	rs9275239	G>A	10	2.35x10-22	0.75 [0.71-0.79]	0.058	45.26
6	32692945	chr6:32692945:C:T	-	Intergenic	rs9275240	C>T	10	2.35x10-22	0.75 [0.71-0.79]	0.058	45.26
6	32692964	chr6:32692964:G:T	-	Intergenic	rs9275241	G>T	10	2.24x10-22	0.75 [0.71-0.79]	0.058	45.28
6	32692997	chr6:32692997:C:T	-	Intergenic	rs9275242	C>T	10	2.02x10-22	0.75 [0.71-0.79]	0.058	45.35
6	32693007	chr6:32693007:C:G	-	Intergenic	rs9275243	C>G	10	1.83x10-22	0.75 [0.71-0.79]	0.061	44.82
6	32693104	chr6:32693104:G:T	-	Intergenic	rs9275244	G>T	10	2.35x10-22	0.75 [0.71-0.79]	0.058	45.26
6	32693166	chr6:32693166:A:G	-	Intergenic	rs9275245	A>G	10	2.35x10-22	0.75 [0.71-0.79]	0.058	45.26
6	32693226	chr6:32693226:C:A	-	Intergenic	rs9275246	C>A	10	2.11x10-22	0.75 [0.71-0.79]	0.061	44.72
6	32693238	chr6:32693238:T:C	-	Intergenic	rs9275247	T>C	10	2.11x10-22	0.75 [0.71-0.79]	0.061	44.72
6	32693358	chr6:32693358:A:G	-	Intergenic	rs9275248	A>G	10	2.35x10-22	0.75 [0.71-0.79]	0.058	45.26
6	32693364	chr6:32693364:C:T	-	Intergenic	rs9275250	C>T	10	2.26x10-22	0.75 [0.71-0.79]	0.058	45.28
6	32693369	chr6:32693369:A:C	-	Intergenic	rs9275251	A>C	10	2.26x10-22	0.75 [0.71-0.79]	0.058	45.28
6	32693427	chr6:32693427:C:T	-	Intergenic	rs9275253	C>T	10	2.35x10-22	0.75 [0.71-0.79]	0.058	45.26
6	32693432	chr6:32693432:T:G	-	Intergenic	rs9275254	T>G	10	2.35x10-22	0.75 [0.71-0.79]	0.058	45.26
6	32693469	chr6:32693469:T:A	-	Intergenic	rs9275255	T>A	10	2.35x10-22	0.75 [0.71-0.79]	0.058	45.26
6	32693516	chr6:32693516:A:G	-	Intergenic	rs2858320	A>G	10	6.92x10-37	0.68 [0.64-0.72]	0.172	29.66
6	32693517	chr6:32693517:T:C	-	Intergenic	rs2858319	T>C	10	1.61x10-34	0.68 [0.64-0.72]	0.297	15.88
6	32693525	chr6:32693525:T:G	-	Intergenic	rs2858318	T>G	10	1.61x10-34	0.68 [0.64-0.72]	0.297	15.88
6	32693795	chr6:32693795:C:A	-	Intergenic	rs9275259	C>A	10	2.35x10-22	0.75 [0.71-0.79]	0.058	45.26
6	32693798	chr6:32693798:C:T	-	Intergenic	rs9275260	C>T	10	2.35x10-22	0.75 [0.71-0.79]	0.058	45.26
6	32694002	chr6:32694002:T:G	-	Intergenic	rs3129719	T>G	10	2.86x10-27	0.66 [0.62-0.71]	0.55	0
6	32694183	chr6:32694183:A:T	-	Intergenic	rs73407347	A>T	10	2.48x10-22	0.75 [0.71-0.79]	0.058	45.33

6	32694247	chr6:32694247:A:G	-	Intergenic	rs111391378	A>G	10	2.35x10-22	0.75 [0.71-0.79]	0.058	45.26
6	32694256	chr6:32694256:A:G	-	Intergenic	rs72844346	A>G	10	2.11x10-22	0.75 [0.71-0.79]	0.061	44.72
6	32694282	chr6:32694282:A:G	-	Intergenic	rs72844347	A>G	10	2.35x10-22	0.75 [0.71-0.79]	0.058	45.26
6	32694307	chr6:32694307:A:G	-	Intergenic	rs73407351	A>G	10	2.35x10-22	0.75 [0.71-0.79]	0.058	45.26
6	32694351	chr6:32694351:A:G	-	Intergenic	rs67838634	A>G	10	4.70x10-25	1.35 [1.28-1.43]	0.058	45.36
6	32694445	chr6:32694445:C:T	-	Intergenic	rs200419915	C>T	10	2.35x10-22	0.75 [0.71-0.79]	0.058	45.26
6	32694462	chr6:32694462:T:C	-	Intergenic	rs201449386	T>C	10	2.35x10-22	0.75 [0.71-0.79]	0.058	45.26
6	32694463	chr6:32694463:G:A	-	Intergenic	rs202170080	G>A	10	2.35x10-22	0.75 [0.71-0.79]	0.058	45.26
6	32694567	chr6:32694567:A:G	-	Intergenic	rs9275263	A>G	10	2.28x10-22	0.75 [0.71-0.79]	0.058	45.27
6	32694595	chr6:32694595:A:G	-	Intergenic	rs9275264	A>G	10	2.06x10-22	0.75 [0.71-0.79]	0.064	44.3
6	32694612	chr6:32694612:C:T	-	Intergenic	rs9275265	C>T	10	2.28x10-22	0.75 [0.71-0.79]	0.058	45.27
6	32694615	chr6:32694615:G:C	-	Intergenic	rs9275266	G>C	10	2.28x10-22	0.75 [0.71-0.79]	0.058	45.27
6	32694648	chr6:32694648:A:G	-	Intergenic	rs9275267	A>G	10	2.35x10-22	0.75 [0.71-0.79]	0.058	45.26
6	32694656	chr6:32694656:A:G	-	Intergenic	rs9275268	A>G	10	2.35x10-22	0.75 [0.71-0.79]	0.058	45.26
6	32694682	chr6:32694682:A:G	-	Intergenic	rs9275269	A>G	10	1.72x10-08	0.76 [0.69-0.84]	0.049	46.98
6	32694756	chr6:32694756:T:C	-	Intergenic	rs9275271	T>C	10	2.35x10-22	0.75 [0.71-0.79]	0.058	45.26
6	32694769	chr6:32694769:G:C	-	Intergenic	rs9275272	G>C	10	5.38x10-13	1.54 [1.37-1.73]	0.003	64.48
6	32694782	chr6:32694782:C:A	-	Intergenic	rs9275273	C>A	10	5.50x10-13	1.54 [1.37-1.73]	0.003	64.53
6	32694789	chr6:32694789:G:C	-	Intergenic	rs9275274	G>C	10	5.38x10-13	1.54 [1.37-1.73]	0.003	64.48
6	32694830	chr6:32694830:G:A	-	Intergenic	rs9275275	G>A	9	1.17x10-65	2.07 [1.92-2.24]	0.953	0
6	32694899	chr6:32694899:T:C	-	Intergenic	rs9275276	T>C	10	2.35x10-22	0.75 [0.71-0.79]	0.058	45.26
6	32694900	chr6:32694900:G:A	-	Intergenic	rs9275277	G>A	10	2.35x10-22	0.75 [0.71-0.79]	0.058	45.26
6	32694922	chr6:32694922:A:T	-	Intergenic	rs114052356	A>T	5	1.06x10-10	0.41 [0.31-0.53]	0.904	0
6	32695066	chr6:32695066:G:A	-	Intergenic	rs9275279	G>A	10	2.35x10-22	0.75 [0.71-0.79]	0.058	45.26
6	32695127	chr6:32695127:G:C	-	Intergenic	rs2856670	G>C	10	1.61x10-34	0.68 [0.64-0.72]	0.297	15.88
6	32695143	chr6:32695143:G:A	-	Intergenic	rs9275281	G>A	10	2.35x10-22	0.75 [0.71-0.79]	0.058	45.26
6	32695197	chr6:32695197:C:T	-	Intergenic	rs9275282	C>T	10	1.48x10-21	0.76 [0.71-0.80]	0.065	44.04
6	32695225	chr6:32695225:C:A	-	Intergenic	rs1794517	C>A	5	1.06x10-10	0.41 [0.31-0.53]	0.904	0
6	32695227	chr6:32695227:A:G	-	Intergenic	rs2647022	A>G	10	1.61x10-34	0.68 [0.64-0.72]	0.297	15.88
6	32695296	chr6:32695296:C:T	-	Intergenic	rs9275284	C>T	10	2.16x10-22	0.75 [0.71-0.79]	0.062	44.58
6	32695303	chr6:32695303:A:G	-	Intergenic	rs9275285	A>G	10	2.34x10-22	0.75 [0.71-0.79]	0.058	45.26
6	32695366	chr6:32695366:T:C	-	Intergenic	rs9275286	T>C	10	2.35x10-22	0.75 [0.71-0.79]	0.058	45.26
6	32695374	chr6:32695374:T:C	-	Intergenic	rs2647021	T>C	10	1.61x10-34	0.68 [0.64-0.72]	0.297	15.88
6	32695426	chr6:32695426:A:G	-	Intergenic	rs9275288	A>G	10	2.35x10-22	0.75 [0.71-0.79]	0.058	45.26
6	32695431	chr6:32695431:A:C	-	Intergenic	rs9275289	A>C	10	2.94x10-13	1.54 [1.37-1.72]	0.004	63.37
6	32695479	chr6:32695479:T:C	-	Intergenic	rs2647020	T>C	10	1.61x10-34	0.68 [0.64-0.72]	0.297	15.88
6	32695483	chr6:32695483:G:A	-	Intergenic	rs9275291	G>A	10	2.93x10-13	1.54 [1.37-1.72]	0.004	63.38
6	32695512	chr6:32695512:C:A	-	Intergenic	rs9275292	C>A	10	2.35x10-22	0.75 [0.71-0.79]	0.058	45.26
6	32695531	chr6:32695531:T:C	-	Intergenic	rs9275293	T>C	9	1.17x10-65	2.07 [1.92-2.24]	0.953	0
6	32695614	chr6:32695614:A:G	-	Intergenic	rs9275295	A>G	10	2.35x10-22	0.75 [0.71-0.79]	0.058	45.26
6	32695654	chr6:32695654:T:C	-	Intergenic	rs9275296	T>C	9	1.17x10-65	2.07 [1.92-2.24]	0.953	0
6	32695669	chr6:32695669:G:A	-	Intergenic	rs9275297	G>A	10	2.92x10-13	1.54 [1.37-1.72]	0.004	63.38
6	32695670	chr6:32695670:T:C	-	Intergenic	rs2647018	T>C	10	1.55x10-34	0.68 [0.64-0.72]	0.297	15.9
6	32695787	chr6:32695787:A:G	-	Intergenic	rs5000634	A>G	10	2.94x10-13	1.54 [1.37-1.72]	0.004	63.37
6	32695829	chr6:32695829:G:C	-	Intergenic	rs2856668	G>C	10	1.61x10-34	0.68 [0.64-0.72]	0.297	15.88
6	32695833	chr6:32695833:C:A	-	Intergenic	rs5000633	C>A	10	2.72x10-22	0.75 [0.71-0.79]	0.058	45.23

6	32695854	chr6:32695854:T:C	-	Intergenic	rs3129720	T>C	10	3.11x10-27	0.66 [0.62-0.71]	0.552	0
6	32696074	chr6:32696074:C:T	-	Intergenic	rs64576174	C>T	10	2.11x10-22	0.75 [0.71-0.79]	0.061	44.72
6	32696096	chr6:32696096:A:G	-	Intergenic	rs373544996	A>G	9	1.17x10-65	2.07 [1.92-2.24]	0.953	0
6	32696148	chr6:32696148:C:A	-	Intergenic	rs2647016	C>A	10	1.61x10-34	0.68 [0.64-0.72]	0.297	15.88
6	32696209	chr6:32696209:C:G	-	Intergenic	rs6457619	C>G	10	3.64x10-13	1.54 [1.37-1.72]	0.003	63.57
6	32696222	chr6:32696222:G:C	-	Intergenic	rs6457620	G>C	10	2.58x10-22	0.75 [0.71-0.79]	0.06	44.92
6	32696349	chr6:32696349:A:G	-	Intergenic	rs9275300	A>G	9	1.17x10-65	2.07 [1.92-2.24]	0.953	0
6	32696386	chr6:32696386:A:C	-	Intergenic	rs6457622	A>C	10	2.95x10-13	1.54 [1.37-1.72]	0.004	63.37
6	32696507	chr6:32696507:C:G	-	Intergenic	rs2647013	C>G	10	1.61x10-34	0.68 [0.64-0.72]	0.297	15.88
6	32696555	chr6:32696555:T:C	-	Intergenic	rs7745040	T>C	10	3.22x10-13	1.54 [1.37-1.72]	0.003	63.49
6	32696681	chr6:32696681:T:C	-	Intergenic	rs2647012	T>C	10	1.61x10-34	0.68 [0.64-0.72]	0.297	15.88
6	32696898	chr6:32696898:A:G	-	Intergenic	rs2894380	A>G	10	2.94x10-13	1.54 [1.37-1.72]	0.004	63.37
6	32696945	chr6:32696945:A:T	-	Intergenic	rs2395522	A>T	10	2.35x10-22	0.75 [0.71-0.79]	0.058	45.26
6	32697103	chr6:32697103:T:G	-	Intergenic	rs2647003	T>G	10	1.67x10-34	0.68 [0.64-0.72]	0.297	15.87
6	32697213	chr6:32697213:A:T	-	Intergenic	rs9275307	A>T	9	9.03x10-66	2.07 [1.92-2.24]	0.953	0
6	32697302	chr6:32697302:T:C	-	Intergenic	rs2856667	T>C	10	3.60x10-22	0.62 [0.56-0.68]	0.039	49.06
6	32697309	chr6:32697309:C:T	-	Intergenic	rs2647001	C>T	10	1.61x10-34	0.68 [0.64-0.72]	0.297	15.88
6	32697478	chr6:32697478:T:A	-	Intergenic	rs9275308	T>A	9	1.17x10-65	2.07 [1.92-2.24]	0.953	0
6	32697481	chr6:32697481:G:T	-	Intergenic	rs9275309	G>T	10	1.46x10-14	1.28 [1.21-1.36]	0.462	0
6	32697508	chr6:32697508:G:T	-	Intergenic	rs2856666	G>T	10	1.61x10-34	0.68 [0.64-0.72]	0.297	15.88
6	32697542	chr6:32697542:T:G	-	Intergenic	rs9275310	T>G	9	1.17x10-65	2.07 [1.92-2.24]	0.953	0
6	32697590	chr6:32697590:T:G	-	Intergenic	rs2646998	T>G	10	1.61x10-34	0.68 [0.64-0.72]	0.297	15.88
6	32697863	chr6:32697863:G:T	-	Intergenic	rs9275311	G>T	9	1.17x10-65	2.07 [1.92-2.24]	0.953	0
6	32697951	chr6:32697951:A:G	-	Intergenic	rs9275312	A>G	9	5.39x10-50	1.79 [1.67-1.93]	0.696	0
6	32697982	chr6:32697982:G:T	-	Intergenic	rs9275313	G>T	9	1.17x10-65	2.07 [1.92-2.24]	0.953	0
6	32698132	chr6:32698132:C:A	-	Intergenic	rs9275314	C>A	9	1.17x10-65	2.07 [1.92-2.24]	0.953	0
6	32698135	chr6:32698135:C:A	-	Intergenic	rs9275315	C>A	9	1.17x10-65	2.07 [1.92-2.24]	0.953	0
6	32698139	chr6:32698139:T:C	-	Intergenic	rs9275316	T>C	10	1.46x10-14	1.28 [1.21-1.36]	0.462	0
6	32698396	chr6:32698396:A:G	-	Intergenic	rs9275318	A>G	9	1.57x10-51	1.82 [1.69-1.96]	0.812	0
6	32698518	chr6:32698518:A:G	-	Intergenic	rs9275319	A>G	9	1.57x10-51	1.82 [1.69-1.96]	0.812	0
6	32698620	chr6:32698620:C:T	-	Intergenic	rs2856727	C>T	10	2.19x10-34	0.68 [0.64-0.72]	0.295	16.07
6	32698692	chr6:32698692:A:C	-	Intergenic	rs9275320	A>C	10	1.44x10-14	1.28 [1.21-1.36]	0.463	0
6	32698783	chr6:32698783:C:G	-	Intergenic	rs9275322	C>G	10	1.64x10-14	1.28 [1.20-1.36]	0.418	2.34
6	32698832	chr6:32698832:T:C	-	Intergenic	rs9275323	T>C	10	1.64x10-14	1.28 [1.20-1.36]	0.418	2.34
6	32698858	chr6:32698858:C:T	-	Intergenic	rs9275324	C>T	9	1.17x10-65	2.07 [1.92-2.24]	0.953	0
6	32698874	chr6:32698874:G:T	-	Intergenic	rs9275325	G>T	9	1.17x10-65	2.07 [1.92-2.24]	0.953	0
6	32698883	chr6:32698883:C:T	-	Intergenic	rs9275326	C>T	9	1.17x10-65	2.07 [1.92-2.24]	0.953	0
6	32698961	chr6:32698961:C:T	-	Intergenic	rs2856725	C>T	10	1.83x10-34	0.68 [0.64-0.72]	0.299	15.61
6	32699025	chr6:32699025:C:T	-	Intergenic	rs9275327	C>T	9	5.39x10-50	1.79 [1.67-1.93]	0.696	0
6	32699040	chr6:32699040:A:G	-	Intergenic	rs1794281	A>G	5	1.06x10-10	0.41 [0.31-0.53]	0.904	0
6	32699045	chr6:32699045:C:T	-	Intergenic	rs9275328	C>T	9	5.39x10-50	1.79 [1.67-1.93]	0.696	0
6	32699054	chr6:32699054:G:T	-	Intergenic	rs9275329	G>T	10	1.46x10-14	1.28 [1.21-1.36]	0.462	0
6	32699098	chr6:32699098:A:G	-	Intergenic	rs9275330	A>G	9	5.39x10-50	1.79 [1.67-1.93]	0.696	0
6	32699122	chr6:32699122:A:G	-	Intergenic	rs9275331	A>G	9	5.95x10-50	1.79 [1.67-1.93]	0.691	0
6	32699166	chr6:32699166:G:A	-	Intergenic	rs9275332	G>A	10	1.46x10-14	1.28 [1.21-1.36]	0.462	0
6	32699191	chr6:32699191:T:C	-	Intergenic	rs9275333	T>C	9	5.39x10-50	1.79 [1.67-1.93]	0.696	0

6	32699330	chr6:32699330:T:C	-	Intergenic	rs9275334	T>C	9	1.17x10-65	2.07 [1.92-2.24]	0.953	0
6	32699342	chr6:32699342:T:C	-	Intergenic	rs3135006	T>C	10	3.54x10-27	0.66 [0.62-0.71]	0.548	0
6	32699503	chr6:32699503:A:G	-	Intergenic	rs2647040	A>G	10	8.54x10-34	0.68 [0.64-0.72]	0.29	16.59
6	32699541	chr6:32699541:G:T	-	Intergenic	rs9275337	G>T	10	1.46x10-14	1.28 [1.21-1.36]	0.462	0
6	32699566	chr6:32699566:A:T	-	Intergenic	rs9275338	A>T	9	5.39x10-50	1.79 [1.67-1.93]	0.696	0
6	32699629	chr6:32699629:G:A	-	Intergenic	rs9275339	G>A	9	1.24x10-18	1.35 [1.27-1.44]	0.701	0
6	32699985	chr6:32699985:A:G	-	Intergenic	rs2856723	A>G	10	2.29x10-33	0.69 [0.65-0.73]	0.278	17.89
6	32700030	chr6:32700030:C:T	-	Intergenic	rs9275352	C>T	10	1.46x10-14	1.28 [1.21-1.36]	0.462	0
6	32700038	chr6:32700038:T:G	-	Intergenic	rs9275353	T>G	10	1.46x10-14	1.28 [1.21-1.36]	0.462	0
6	32700043	chr6:32700043:T:G	-	Intergenic	rs9275354	T>G	10	1.46x10-14	1.28 [1.21-1.36]	0.462	0
6	32700052	chr6:32700052:T:C	-	Intergenic	rs9275355	T>C	10	1.46x10-14	1.28 [1.21-1.36]	0.462	0
6	32700073	chr6:32700073:C:G	-	Intergenic	rs9275356	C>G	9	1.17x10-65	2.07 [1.92-2.24]	0.953	0
6	32700114	chr6:32700114:A:C	-	Intergenic	rs9275357	A>C	10	1.46x10-14	1.28 [1.21-1.36]	0.462	0
6	32700118	chr6:32700118:C:G	-	Intergenic	rs9275358	C>G	9	5.39x10-50	1.79 [1.67-1.93]	0.696	0
6	32700160	chr6:32700160:A:T	-	Intergenic	rs9275360	A>T	9	5.39x10-50	1.79 [1.67-1.93]	0.696	0
6	32700169	chr6:32700169:T:G	-	Intergenic	rs3135190	T>G	10	2.41x10-26	0.66 [0.62-0.71]	0.468	0
6	32700180	chr6:32700180:C:T	-	Intergenic	rs9275362	C>T	9	5.39x10-50	1.79 [1.67-1.93]	0.696	0
6	32700181	chr6:32700181:A:G	-	Intergenic	rs2856722	A>G	10	1.72x10-33	0.68 [0.65-0.73]	0.295	16.07
6	32700250	chr6:32700250:A:G	-	Intergenic	rs9275364	A>G	10	1.46x10-14	1.28 [1.21-1.36]	0.462	0
6	32700323	chr6:32700323:G:A	-	Intergenic	rs2647045	G>A	9	5.42x10-44	1.55 [1.47-1.65]	0.323	13.36
6	32700348	chr6:32700348:G:A	-	Intergenic	rs9275365	G>A	9	5.07x10-50	1.79 [1.67-1.93]	0.695	0
6	32700394	chr6:32700394:A:C	-	Intergenic	rs9275366	A>C	10	1.92x10-14	1.28 [1.20-1.36]	0.474	0
6	32700405	chr6:32700405:A:G	-	Intergenic	rs9275367	A>G	10	1.92x10-14	1.28 [1.20-1.36]	0.474	0
6	32700406	chr6:32700406:C:G	-	Intergenic	rs9275368	C>G	10	1.92x10-14	1.28 [1.20-1.36]	0.474	0
6	32700478	chr6:32700478:C:T	-	Intergenic	rs9275369	C>T	10	1.59x10-14	1.28 [1.20-1.36]	0.46	0
6	32700481	chr6:32700481:C:T	-	Intergenic	rs9275370	C>T	10	1.59x10-14	1.28 [1.20-1.36]	0.46	0
6	32700519	chr6:32700519:T:C	-	Intergenic	rs9275371	T>C	10	1.46x10-14	1.28 [1.21-1.36]	0.462	0
6	32700546	chr6:32700546:G:A	-	Intergenic	rs2858310	G>A	10	1.46x10-33	0.68 [0.65-0.73]	0.293	16.3
6	32700559	chr6:32700559:A:C	-	Intergenic	rs2647046	A>C	10	1.90x10-33	0.68 [0.65-0.73]	0.296	15.96
6	32700611	chr6:32700611:C:T	-	Intergenic	rs9275372	C>T	10	1.46x10-14	1.28 [1.21-1.36]	0.462	0
6	32700634	chr6:32700634:G:A	-	Intergenic	rs9275373	G>A	9	1.84x10-55	1.92 [1.78-2.08]	0.846	0
6	32700662	chr6:32700662:A:C	-	Intergenic	rs3135002	A>C	10	2.63x10-19	0.69 [0.64-0.75]	0.74	0
6	32700749	chr6:32700749:C:T	-	Intergenic	rs9275374	C>T	10	1.19x10-14	1.28 [1.21-1.36]	0.481	0
6	32700855	chr6:32700855:G:A	-	Intergenic	rs9275375	G>A	10	1.19x10-14	1.28 [1.21-1.36]	0.481	0
6	32700856	chr6:32700856:G:T	-	Intergenic	rs9275376	G>T	10	7.06x10-15	1.29 [1.21-1.37]	0.497	0
6	32700890	chr6:32700890:A:G	-	Intergenic	rs9275377	A>G	10	7.06x10-15	1.29 [1.21-1.37]	0.497	0
6	32700928	chr6:32700928:A:T	-	Intergenic	rs2647048	A>T	10	1.90x10-33	0.68 [0.65-0.73]	0.296	15.96
6	32700936	chr6:32700936:C:G	-	Intergenic	rs2858309	C>G	10	1.90x10-33	0.68 [0.65-0.73]	0.296	15.96
6	32700952	chr6:32700952:C:G	-	Intergenic	rs9275378	C>G	10	7.06x10-15	1.29 [1.21-1.37]	0.497	0
6	32701027	chr6:32701027:C:T	-	Intergenic	rs9275379	C>T	10	7.06x10-15	1.29 [1.21-1.37]	0.497	0
6	32701037	chr6:32701037:A:C	-	Intergenic	rs9275380	A>C	9	2.60x10-63	2.02 [1.87-2.18]	0.801	0
6	32701042	chr6:32701042:A:C	-	Intergenic	rs9275381	A>C	10	1.19x10-14	1.28 [1.21-1.36]	0.481	0
6	32701054	chr6:32701054:T:C	-	Intergenic	rs9275382	T>C	9	1.98x10-63	2.02 [1.87-2.18]	0.795	0
6	32701069	chr6:32701069:G:T	-	Intergenic	rs9275383	G>T	9	1.86x10-63	2.02 [1.87-2.19]	0.797	0
6	32701121	chr6:32701121:T:C	-	Intergenic	rs9275384	T>C	10	1.19x10-14	1.28 [1.21-1.36]	0.481	0
6	32701140	chr6:32701140:C:T	MTCO3P1	Intergenic	rs9275385	C>T	10	1.19x10-14	1.28 [1.21-1.36]	0.481	0

6	32701183	chr6:32701183:A:T	MTCO3P1	Intergenic	rs9275386	A>T	10	1.19x10-14	1.28 [1.21-1.36]	0.481	0
6	32701236	chr6:32701236:T:C	MTCO3P1	Intergenic	rs9275387	T>C	9	1.17x10-65	2.07 [1.92-2.24]	0.953	0
6	32701241	chr6:32701241:C:A	MTCO3P1	Intergenic	rs1612904	C>A	10	5.86x10-22	0.74 [0.69-0.78]	0.053	46.3
6	32701307	chr6:32701307:T:C	MTCO3P1	Intergenic	rs9275388	T>C	10	1.19x10-14	1.28 [1.21-1.36]	0.481	0
6	32701355	chr6:32701355:C:T	MTCO3P1	Intergenic	rs9275389	C>T	10	1.83x10-33	0.68 [0.65-0.73]	0.296	15.98
6	32701379	chr6:32701379:T:C	MTCO3P1	Intergenic	rs9275390	T>C	10	1.19x10-14	1.28 [1.21-1.36]	0.481	0
6	32701392	chr6:32701392:C:T	MTCO3P1	Intergenic	rs9275391	C>T	10	2.59x10-15	1.29 [1.21-1.37]	0.488	0
6	32701648	chr6:32701648:A:T	MTCO3P1	Intergenic	rs9275392	A>T	10	1.07x10-14	1.28 [1.21-1.36]	0.481	0
6	32701662	chr6:32701662:G:A	MTCO3P1	Intergenic	rs9275393	G>A	10	1.19x10-14	1.28 [1.21-1.36]	0.481	0
6	32701677	chr6:32701677:A:C	MTCO3P1	Intergenic	rs9275394	A>C	9	1.17x10-65	2.07 [1.92-2.24]	0.953	0
6	32701706	chr6:32701706:A:G	MTCO3P1	Intergenic	rs9275395	A>G	9	1.17x10-65	2.07 [1.92-2.24]	0.953	0
6	32701751	chr6:32701751:T:A	MTCO3P1	Intergenic	rs9275396	T>A	10	7.06x10-15	1.29 [1.21-1.37]	0.497	0
6	32701791	chr6:32701791:A:C	MTCO3P1	Intergenic	rs9275398	A>C	10	1.09x10-14	1.28 [1.21-1.36]	0.483	0
6	32701935	chr6:32701935:A:G	MTCO3P1	Intergenic	rs9275399	A>G	10	7.06x10-15	1.29 [1.21-1.37]	0.497	0
6	32701984	chr6:32701984:C:G	MTCO3P1	Intergenic	rs9275400	C>G	9	1.17x10-65	2.07 [1.92-2.24]	0.953	0
6	32702050	chr6:32702050:C:G	MTCO3P1	Intergenic	rs9275401	C>G	10	1.40x10-14	1.28 [1.21-1.36]	0.478	0
6	32702060	chr6:32702060:T:C	MTCO3P1	Intergenic	rs9275402	T>C	10	1.19x10-14	1.28 [1.21-1.36]	0.481	0
6	32702061	chr6:32702061:G:T	MTCO3P1	Intergenic	rs9275403	G>T	10	1.19x10-14	1.28 [1.21-1.36]	0.481	0
6	32702101	chr6:32702101:T:C	MTCO3P1	Intergenic	rs9275404	T>C	10	1.19x10-14	1.28 [1.21-1.36]	0.481	0
6	32702106	chr6:32702106:A:G	MTCO3P1	Intergenic	rs9275405	A>G	10	1.22x10-14	1.28 [1.21-1.36]	0.484	0
6	32702178	chr6:32702178:G:T	MTCO3P1	Intergenic	rs9275406	G>T	10	1.19x10-14	1.28 [1.21-1.36]	0.481	0
6	32702260	chr6:32702260:G:T	MTCO3P1	Intergenic	rs9275407	G>T	10	1.19x10-14	1.28 [1.21-1.36]	0.481	0
6	32702333	chr6:32702333:T:C	MTCO3P1	Intergenic	rs9275408	T>C	10	1.19x10-14	1.28 [1.21-1.36]	0.481	0
6	32702359	chr6:32702359:T:C	MTCO3P1	Intergenic	rs3135001	T>C	10	2.28x10-26	0.66 [0.62-0.71]	0.462	0
6	32702368	chr6:32702368:G:A	MTCO3P1	Intergenic	rs9275410	G>A	10	1.19x10-14	1.28 [1.21-1.36]	0.481	0
6	32702380	chr6:32702380:G:T	MTCO3P1	Intergenic	rs9275411	G>T	10	1.09x10-14	1.28 [1.21-1.36]	0.483	0
6	32702419	chr6:32702419:C:T	MTCO3P1	Intergenic	rs9275416	C>T	10	2.84x10-18	1.33 [1.25-1.42]	0.524	0
6	32702437	chr6:32702437:A:C	MTCO3P1	Intergenic	rs9275417	A>C	10	1.51x10-14	1.28 [1.21-1.36]	0.497	0
6	32702467	chr6:32702467:A:G	MTCO3P1	Intergenic	rs9275418	A>G	10	1.19x10-14	1.28 [1.21-1.36]	0.481	0
6	32702531	chr6:32702531:A:G	MTCO3P1	Intergenic	rs2856717	A>G	10	1.83x10-33	0.68 [0.65-0.73]	0.296	15.98
6	32702687	chr6:32702687:G:T	MTCO3P1	Intergenic	rs2858305	G>T	10	1.83x10-33	0.68 [0.65-0.73]	0.296	15.98
6	32702700	chr6:32702700:C:A	MTCO3P1	Intergenic	rs9275421	C>A	10	1.19x10-14	1.28 [1.21-1.36]	0.481	0
6	32702771	chr6:32702771:G:A	MTCO3P1	Intergenic	rs9275422	G>A	9	1.17x10-65	2.07 [1.92-2.24]	0.953	0
6	32702787	chr6:32702787:G:T	MTCO3P1	Intergenic	rs9275423	G>T	10	1.19x10-14	1.28 [1.21-1.36]	0.481	0
6	32702799	chr6:32702799:A:G	MTCO3P1	Intergenic	rs9275424	A>G	10	1.19x10-14	1.28 [1.21-1.36]	0.481	0
6	32703097	chr6:32703097:C:A	MTCO3P1	Intergenic	rs9275425	C>A	10	1.19x10-14	1.28 [1.21-1.36]	0.481	0
6	32703120	chr6:32703120:T:C	MTCO3P1	Intergenic	rs2647051	T>C	10	1.83x10-33	0.68 [0.65-0.73]	0.296	15.98
6	32703135	chr6:32703135:T:C	MTCO3P1	Intergenic	rs9275426	T>C	9	1.17x10-65	2.07 [1.92-2.24]	0.953	0
6	32703138	chr6:32703138:C:T	MTCO3P1	Intergenic	rs9275427	C>T	10	1.19x10-14	1.28 [1.21-1.36]	0.481	0
6	32703201	chr6:32703201:A:G	MTCO3P1	Intergenic	rs9275428	A>G	10	1.10x10-14	1.28 [1.21-1.36]	0.483	0
6	32703280	chr6:32703280:G:A	MTCO3P1	Intergenic	rs9275429	G>A	9	1.17x10-65	2.07 [1.92-2.24]	0.953	0
6	32703309	chr6:32703309:C:T	MTCO3P1	Intergenic	rs9275430	C>T	9	1.07x10-65	2.07 [1.92-2.24]	0.953	0
6	32703363	chr6:32703363:G:A	MTCO3P1	Intergenic	rs9275431	G>A	10	1.19x10-14	1.28 [1.21-1.36]	0.481	0
6	32703408	chr6:32703408:G:T	MTCO3P1	Intergenic	rs9275432	G>T	10	1.19x10-14	1.28 [1.21-1.36]	0.481	0
6	32703429	chr6:32703429:G:C	MTCO3P1	Intergenic	rs9275433	G>C	10	1.19x10-14	1.28 [1.21-1.36]	0.481	0
6	32703470	chr6:32703470:C:T	MTCO3P1	Intergenic	rs9275434	C>T	9	1.17x10-65	2.07 [1.92-2.24]	0.953	0

6	32703555	chr6:32703555:G:C	MTCO3P1	Intergenic	rs9275435	G>C	9	1.17x10-65	2.07 [1.92-2.24]	0.953	0
6	32703622	chr6:32703622:C:G	MTCO3P1	Intergenic	rs9275436	C>G	10	1.19x10-14	1.28 [1.21-1.36]	0.481	0
6	32703635	chr6:32703635:C:T	MTCO3P1	Intergenic	rs9275437	C>T	9	1.17x10-65	2.07 [1.92-2.24]	0.953	0
6	32703731	chr6:32703731:C:T	MTCO3P1	Intergenic	rs9275438	C>T	10	1.19x10-14	1.28 [1.21-1.36]	0.481	0
6	32703744	chr6:32703744:T:C	MTCO3P1	Intergenic	rs9275439	T>C	10	1.19x10-14	1.28 [1.21-1.36]	0.481	0
6	32703819	chr6:32703819:C:T	MTCO3P1	Intergenic	rs9275440	C>T	10	1.19x10-14	1.28 [1.21-1.36]	0.481	0
6	32703899	chr6:32703899:C:A	MTCO3P1	Intergenic	rs9275441	C>A	10	1.20x10-14	1.28 [1.21-1.36]	0.482	0
6	32703978	chr6:32703978:G:A	MTCO3P1	Intergenic	rs9275442	G>A	9	1.17x10-65	2.07 [1.92-2.24]	0.953	0
6	32703988	chr6:32703988:A:G	MTCO3P1	Intergenic	rs2856699	A>G	10	2.29x10-33	0.69 [0.65-0.73]	0.299	15.67
6	32704305	chr6:32704305:A:G	MTCO3P1	Intergenic	rs9275464	A>G	10	7.06x10-15	1.29 [1.21-1.37]	0.497	0
6	32704312	chr6:32704312:T:C	MTCO3P1	Intergenic	rs3134995	T>C	10	9.47x10-27	0.66 [0.62-0.71]	0.47	0
6	32704469	chr6:32704469:A:C	MTCO3P1	Intergenic	rs4568495	A>C	10	7.06x10-15	1.29 [1.21-1.37]	0.497	0
6	32704606	chr6:32704606:G:C	MTCO3P1	Intergenic	rs1794529	G>C	5	1.06x10-10	0.41 [0.31-0.53]	0.904	0
6	32704635	chr6:32704635:G:A	MTCO3P1	Intergenic	rs9275468	G>A	10	7.06x10-15	1.29 [1.21-1.37]	0.497	0
6	32704653	chr6:32704653:A:G	MTCO3P1	Intergenic	rs9275469	A>G	10	7.06x10-15	1.29 [1.21-1.37]	0.497	0
6	32704714	chr6:32704714:G:A	MTCO3P1	Intergenic	rs9275471	G>A	10	7.06x10-15	1.29 [1.21-1.37]	0.497	0
6	32704717	chr6:32704717:G:A	MTCO3P1	Intergenic	rs9275472	G>A	10	7.06x10-15	1.29 [1.21-1.37]	0.497	0
6	32704746	chr6:32704746:C:T	MTCO3P1	Intergenic	rs9275473	C>T	10	7.06x10-15	1.29 [1.21-1.37]	0.497	0
6	32704752	chr6:32704752:C:T	MTCO3P1	Intergenic	rs9275474	C>T	10	7.06x10-15	1.29 [1.21-1.37]	0.497	0
6	32704779	chr6:32704779:A:G	MTCO3P1	Intergenic	rs9275475	A>G	10	7.06x10-15	1.29 [1.21-1.37]	0.497	0
6	32704847	chr6:32704847:T:C	MTCO3P1	Intergenic	rs9275476	T>C	9	1.17x10-65	2.07 [1.92-2.24]	0.953	0
6	32704864	chr6:32704864:A:C	MTCO3P1	Intergenic	rs9275477	A>C	9	1.17x10-65	2.07 [1.92-2.24]	0.953	0
6	32704877	chr6:32704877:G:T	MTCO3P1	Intergenic	rs9275478	G>T	10	7.06x10-15	1.29 [1.21-1.37]	0.497	0
6	32704882	chr6:32704882:C:T	MTCO3P1	Intergenic	rs9275479	C>T	10	8.25x10-15	1.29 [1.21-1.37]	0.512	0
6	32704891	chr6:32704891:G:A	MTCO3P1	Intergenic	rs9275480	G>A	10	7.06x10-15	1.29 [1.21-1.37]	0.497	0
6	32705136	chr6:32705136:T:A	MTCO3P1	Intergenic	rs9275481	T>A	10	7.06x10-15	1.29 [1.21-1.37]	0.497	0
6	32705155	chr6:32705155:G:T	MTCO3P1	Intergenic	rs9275482	G>T	10	7.06x10-15	1.29 [1.21-1.37]	0.497	0
6	32705274	chr6:32705274:T:C	MTCO3P1	Intergenic	rs9275483	T>C	10	6.94x10-15	1.29 [1.21-1.37]	0.495	0
6	32705275	chr6:32705275:G:T	MTCO3P1	Intergenic	rs9275484	G>T	10	6.94x10-15	1.29 [1.21-1.37]	0.495	0
6	32705300	chr6:32705300:G:A	MTCO3P1	Intergenic	rs9275485	G>A	10	7.06x10-15	1.29 [1.21-1.37]	0.497	0
6	32705322	chr6:32705322:T:A	MTCO3P1	Intergenic	rs9275486	T>A	9	1.07x10-65	2.07 [1.92-2.24]	0.954	0
6	32705397	chr6:32705397:C:T	MTCO3P1	Intergenic	rs9275487	C>T	10	6.94x10-15	1.29 [1.21-1.37]	0.498	0
6	32705492	chr6:32705492:G:A	MTCO3P1	Intergenic	rs9275488	G>A	10	7.06x10-15	1.29 [1.21-1.37]	0.497	0
6	32705495	chr6:32705495:C:T	MTCO3P1	Intergenic	rs9275489	C>T	10	7.06x10-15	1.29 [1.21-1.37]	0.497	0
6	32705608	chr6:32705608:C:G	MTCO3P1	Intergenic	rs9275490	C>G	9	1.17x10-65	2.07 [1.92-2.24]	0.953	0
6	32705647	chr6:32705647:C:T	MTCO3P1	Intergenic	rs9275491	C>T	10	7.06x10-15	1.29 [1.21-1.37]	0.497	0
6	32705669	chr6:32705669:C:T	MTCO3P1	Intergenic	rs9275492	C>T	10	7.66x10-15	1.29 [1.21-1.37]	0.495	0
6	32705702	chr6:32705702:G:C	MTCO3P1	Intergenic	rs9275494	G>C	10	7.06x10-15	1.29 [1.21-1.37]	0.497	0
6	32705797	chr6:32705797:A:T	MTCO3P1	Intergenic	rs9275495	A>T	9	1.17x10-65	2.07 [1.92-2.24]	0.953	0
6	32705937	chr6:32705937:T:C	MTCO3P1	Intergenic	rs9275496	T>C	10	7.06x10-15	1.29 [1.21-1.37]	0.497	0
6	32705949	chr6:32705949:A:G	MTCO3P1	Intergenic	rs9275497	A>G	10	7.06x10-15	1.29 [1.21-1.37]	0.497	0
6	32705972	chr6:32705972:C:T	MTCO3P1	Intergenic	rs9275498	C>T	10	7.06x10-15	1.29 [1.21-1.37]	0.497	0
6	32705985	chr6:32705985:G:C	MTCO3P1	Intergenic	rs1618221	G>C	5	1.06x10-10	0.41 [0.31-0.53]	0.904	0
6	32706011	chr6:32706011:C:T	MTCO3P1	Intergenic	rs9275500	C>T	10	2.51x10-22	0.75 [0.71-0.79]	0.06	45.03
6	32706042	chr6:32706042:C:G	MTCO3P1	Intergenic	rs1794270	C>G	5	1.06x10-10	0.41 [0.31-0.53]	0.904	0
6	32706117	chr6:32706117:C:T	MTCO3P1	Intergenic	rs1794269	C>T	10	4.77x10-11	1.21 [1.15-1.28]	0.859	0

6	32707061	chr6:32707061:G:C	MTCO3P1	Intergenic	rs9275519	G>C	10	1.22x10-33	0.68 [0.65-0.73]	0.296	15.95
6	32707062	chr6:32707062:G:C	MTCO3P1	Intergenic	rs866379110	G>C	10	6.10x10-36	0.68 [0.64-0.72]	0.149	32.37
6	32707175	chr6:32707175:T:C	MTCO3P1	Intergenic	rs9275521	T>C	10	1.10x10-33	0.68 [0.64-0.73]	0.295	16.15
6	32707193	chr6:32707193:C:T	MTCO3P1	Intergenic	rs9275522	C>T	9	2.84x10-18	1.35 [1.26-1.44]	0.671	0
6	32707217	chr6:32707217:C:A	MTCO3P1	Intergenic	rs9275523	C>A	9	2.84x10-18	1.35 [1.26-1.44]	0.671	0
6	32707332	chr6:32707332:T:C	MTCO3P1	Intergenic	rs9275524	T>C	10	6.50x10-36	0.68 [0.64-0.72]	0.151	32.13
6	32707456	chr6:32707456:G:A	MTCO3P1	Intergenic	rs9275525	G>A	9	2.84x10-18	1.35 [1.26-1.44]	0.671	0
6	32707460	chr6:32707460:G:T	MTCO3P1	Intergenic	rs9275526	G>T	10	6.50x10-36	0.68 [0.64-0.72]	0.151	32.13
6	32707601	chr6:32707601:C:G	MTCO3P1	Intergenic	rs9275527	C>G	9	1.70x10-18	1.35 [1.27-1.44]	0.715	0
6	32707630	chr6:32707630:T:C	MTCO3P1	Intergenic	rs9275528	T>C	9	2.60x10-18	1.35 [1.26-1.44]	0.673	0
6	32707653	chr6:32707653:C:T	MTCO3P1	Intergenic	rs9275529	C>T	9	2.73x10-18	1.35 [1.26-1.44]	0.67	0
6	32707746	chr6:32707746:G:C	MTCO3P1	Intergenic	rs9275530	G>C	9	1.17x10-65	2.07 [1.92-2.24]	0.953	0
6	32707815	chr6:32707815:A:G	MTCO3P1	Intergenic	rs9275531	A>G	9	1.70x10-18	1.35 [1.27-1.44]	0.715	0
6	32707857	chr6:32707857:C:G	MTCO3P1	Intergenic	rs9275532	C>G	9	1.17x10-65	2.07 [1.92-2.24]	0.953	0
6	32707868	chr6:32707868:G:A	MTCO3P1	Intergenic	rs17219281	G>A	9	1.37x10-11	1.41 [1.28-1.56]	0.387	5.78
6	32707973	chr6:32707973:G:C	MTCO3P1	Intergenic	rs9275533	G>C	10	6.50x10-36	0.68 [0.64-0.72]	0.151	32.13
6	32707990	chr6:32707990:C:T	MTCO3P1	Intergenic	rs9275534	C>T	9	2.84x10-18	1.35 [1.26-1.44]	0.671	0
6	32708012	chr6:32708012:G:A	MTCO3P1	Intergenic	rs9275535	G>A	10	6.82x10-36	0.68 [0.64-0.72]	0.148	32.52
6	32708052	chr6:32708052:A:G	MTCO3P1	Intergenic	rs9275536	A>G	10	6.50x10-36	0.68 [0.64-0.72]	0.151	32.13
6	32708125	chr6:32708125:T:G	MTCO3P1	Intergenic	rs1794524	T>G	10	4.31x10-22	0.74 [0.69-0.78]	0.052	46.45
6	32708240	chr6:32708240:T:C	MTCO3P1	Intergenic	rs9275538	T>C	10	6.75x10-36	0.68 [0.64-0.72]	0.158	31.33
6	32708271	chr6:32708271:G:A	MTCO3P1	Intergenic	rs9275539	G>A	9	2.84x10-18	1.35 [1.26-1.44]	0.671	0
6	32708362	chr6:32708362:T:A	MTCO3P1	Intergenic	rs9275540	T>A	10	5.89x10-36	0.68 [0.64-0.72]	0.151	32.13
6	32708382	chr6:32708382:C:G	MTCO3P1	Intergenic	rs9275541	C>G	9	1.17x10-65	2.07 [1.92-2.24]	0.953	0
6	32708469	chr6:32708469:C:T	MTCO3P1	Intergenic	rs9275542	C>T	9	3.08x10-18	1.35 [1.26-1.44]	0.669	0
6	32708474	chr6:32708474:C:A	MTCO3P1	Intergenic	rs3129721	C>A	10	4.05x10-22	0.74 [0.69-0.78]	0.052	46.5
6	32708566	chr6:32708566:C:A	MTCO3P1	Intergenic	rs3129722	C>A	5	1.06x10-10	0.41 [0.31-0.53]	0.904	0
6	32708576	chr6:32708576:A:G	MTCO3P1	Intergenic	rs9275544	A>G	9	2.84x10-18	1.35 [1.26-1.44]	0.671	0
6	32708578	chr6:32708578:C:T	MTCO3P1	Intergenic	rs9275545	C>T	9	2.84x10-18	1.35 [1.26-1.44]	0.671	0
6	32708668	chr6:32708668:T:G	MTCO3P1	Intergenic	rs9275546	T>G	9	2.84x10-18	1.35 [1.26-1.44]	0.671	0
6	32708748	chr6:32708748:T:G	MTCO3P1	Intergenic	rs9275547	T>G	9	1.55x10-18	1.35 [1.27-1.44]	0.708	0
6	32709311	chr6:32709311:C:T	MTCO3P1	Intergenic	rs9275555	C>T	9	3.07x10-18	1.35 [1.26-1.44]	0.68	0
6	32709413	chr6:32709413:C:T	MTCO3P1	Intergenic	rs9275556	C>T	10	7.04x10-36	0.68 [0.64-0.72]	0.151	32.14
6	32709422	chr6:32709422:C:T	MTCO3P1	Intergenic	rs9275557	C>T	10	7.04x10-36	0.68 [0.64-0.72]	0.151	32.14
6	32709650	chr6:32709650:G:T	MTCO3P1	Intergenic	rs9275558	G>T	9	3.29x10-18	1.35 [1.26-1.44]	0.678	0
6	32710012	chr6:32710012:C:T	MTCO3P1	Intergenic	rs9275561	C>T	9	3.29x10-18	1.35 [1.26-1.44]	0.678	0
6	32710069	chr6:32710069:T:C	MTCO3P1	Intergenic	rs4947344	T>C	10	1.27x10-39	0.62 [0.58-0.67]	0.633	0
6	32710135	chr6:32710135:C:T	MTCO3P1	Intergenic	rs9275563	C>T	10	2.50x10-17	0.60 [0.54-0.68]	0.003	63.7
6	32710150	chr6:32710150:C:A	MTCO3P1	Intergenic	rs9275564	C>A	9	3.29x10-18	1.35 [1.26-1.44]	0.678	0
6	32710161	chr6:32710161:T:C	MTCO3P1	Intergenic	rs9275565	T>C	10	7.04x10-36	0.68 [0.64-0.72]	0.151	32.14
6	32710310	chr6:32710310:T:A	MTCO3P1	Intergenic	rs9275566	T>A	9	2.44x10-18	1.35 [1.26-1.44]	0.714	0
6	32710354	chr6:32710354:A:G	MTCO3P1	Intergenic	rs9275567	A>G	10	1.10x10-35	0.68 [0.64-0.72]	0.156	31.57
6	32710358	chr6:32710358:A:C	MTCO3P1	Intergenic	rs9275568	A>C	9	5.41x10-18	1.34 [1.26-1.43]	0.645	0
6	32710359	chr6:32710359:C:T	MTCO3P1	Intergenic	rs9275569	C>T	10	1.19x10-35	0.68 [0.64-0.72]	0.155	31.7
6	32710405	chr6:32710405:C:G	MTCO3P1	Intergenic	rs6932517	C>G	10	9.77x10-36	0.68 [0.64-0.72]	0.153	31.94
6	32710422	chr6:32710422:G:A	MTCO3P1	Intergenic	rs9275570	G>A	10	1.27x10-35	0.68 [0.64-0.72]	0.154	31.79

6	32710429	chr6:32710429:G:A	MTCO3P1	Intergenic	rs3129726	G>A	10	1.91x10-25	0.67 [0.62-0.72]	0.528	0
6	32710483	chr6:32710483:T:A	MTCO3P1	Intergenic	rs4947345	T>A	10	1.93x10-33	0.68 [0.65-0.73]	0.302	15.37
6	32710546	chr6:32710546:G:C	MTCO3P1	Intergenic	rs4516986	G>C	9	2.44x10-18	1.35 [1.26-1.44]	0.714	0
6	32710647	chr6:32710647:G:A	MTCO3P1	Intergenic	rs2894281	G>A	9	4.31x10-18	1.34 [1.26-1.43]	0.672	0
6	32710700	chr6:32710700:C:A	MTCO3P1	Intergenic	rs3998157	C>A	10	9.77x10-36	0.68 [0.64-0.72]	0.153	31.94
6	32710714	chr6:32710714:T:C	MTCO3P1	Intergenic	rs4273728	T>C	9	2.92x10-65	2.07 [1.91-2.24]	0.953	0
6	32710820	chr6:32710820:C:G	MTCO3P1	Intergenic	rs4273729	C>G	10	9.77x10-36	0.68 [0.64-0.72]	0.153	31.94
6	32711070	chr6:32711070:T:G	MTCO3P1	Intergenic	rs9275571	T>G	9	4.31x10-18	1.34 [1.26-1.43]	0.672	0
6	32711090	chr6:32711090:G:A	MTCO3P1	Intergenic	rs4476871	G>A	10	9.77x10-36	0.68 [0.64-0.72]	0.153	31.94
6	32711119	chr6:32711119:C:A	MTCO3P1	Intergenic	rs3135191	C>A	5	1.80x10-10	0.41 [0.32-0.54]	0.906	0
6	32711369	chr6:32711369:C:G	MTCO3P1	Intergenic	rs9275573	C>G	10	1.03x10-35	0.68 [0.64-0.72]	0.155	31.72
6	32711406	chr6:32711406:T:A	MTCO3P1	Intergenic	rs9275574	T>A	9	2.44x10-18	1.35 [1.26-1.44]	0.714	0
6	32711408	chr6:32711408:T:A	MTCO3P1	Intergenic	rs9275575	T>A	9	2.44x10-18	1.35 [1.26-1.44]	0.714	0
6	32711568	chr6:32711568:A:T	MTCO3P1	Intergenic	rs9275577	A>T	10	1.12x10-35	0.68 [0.64-0.72]	0.209	25.45
6	32711607	chr6:32711607:G:T	MTCO3P1	Intergenic	rs9275578	G>T	9	2.44x10-18	1.35 [1.26-1.44]	0.714	0
6	32711682	chr6:32711682:T:A	MTCO3P1	Intergenic	rs9275579	T>A	9	4.31x10-18	1.34 [1.26-1.43]	0.672	0
6	32711685	chr6:32711685:A:G	MTCO3P1	Intergenic	rs9275580	A>G	9	4.31x10-18	1.34 [1.26-1.43]	0.672	0
6	32711913	chr6:32711913:C:T	MTCO3P1	Intergenic	rs3129727	C>T	5	1.80x10-10	0.41 [0.32-0.54]	0.906	0
6	32712137	chr6:32712137:T:C	-	Intergenic	rs9275581	T>C	9	2.19x10-18	1.35 [1.27-1.44]	0.729	0
6	32712293	chr6:32712293:C:T	-	Intergenic	rs9275582	C>T	9	2.19x10-18	1.35 [1.27-1.44]	0.729	0
6	32712345	chr6:32712345:G:A	-	Intergenic	rs3135461	G>A	10	1.01x10-22	0.69 [0.64-0.74]	0.239	22.15
6	32712372	chr6:32712372:G:A	-	Intergenic	rs3129728	G>A	5	1.06x10-10	0.41 [0.31-0.53]	0.904	0
6	32712500	chr6:32712500:A:C	-	Intergenic	rs3129729	A>C	5	1.06x10-10	0.41 [0.31-0.53]	0.904	0
6	32712522	chr6:32712522:G:A	-	Intergenic	rs9275583	G>A	9	1.47x10-65	2.07 [1.91-2.24]	0.951	0
6	32712573	chr6:32712573:T:C	-	Intergenic	rs9275585	T>C	9	3.72x10-18	1.34 [1.26-1.43]	0.684	0
6	32712599	chr6:32712599:G:T	-	Intergenic	rs9275586	G>T	9	3.45x10-18	1.35 [1.26-1.43]	0.686	0
6	32712602	chr6:32712602:T:C	-	Intergenic	rs9275587	T>C	9	2.19x10-18	0.77 [0.73-0.82]	0.11	38.69
6	32712646	chr6:32712646:G:A	-	Intergenic	rs9275588	G>A	9	2.29x10-18	0.77 [0.73-0.82]	0.149	33.66
6	32712664	chr6:32712664:T:G	-	Intergenic	rs9275589	T>G	9	2.29x10-18	0.77 [0.73-0.82]	0.149	33.66
6	32712703	chr6:32712703:T:C	-	Intergenic	rs9275590	T>C	9	2.19x10-18	1.35 [1.27-1.44]	0.729	0
6	32712785	chr6:32712785:C:T	-	Intergenic	rs9275591	C>T	9	2.15x10-18	1.35 [1.27-1.44]	0.728	0
6	32712843	chr6:32712843:G:T	-	Intergenic	rs9275592	G>T	9	2.23x10-65	2.07 [1.91-2.24]	0.952	0
6	32712863	chr6:32712863:T:A	-	Intergenic	rs7764856	T>A	10	5.17x10-28	1.40 [1.32-1.48]	0.22	24.23
6	32713066	chr6:32713066:G:A	-	Intergenic	rs9275593	G>A	9	2.19x10-18	1.35 [1.27-1.44]	0.729	0
6	32713384	chr6:32713384:G:T	-	Intergenic	rs2858332	G>T	9	3.21x10-18	0.77 [0.73-0.82]	0.123	37.01
6	32713578	chr6:32713578:T:C	-	Intergenic	rs9275595	T>C	9	2.70x10-18	1.35 [1.26-1.44]	0.715	0
6	32713706	chr6:32713706:T:C	-	Intergenic	rs7454108	T>C	9	2.82x10-65	2.07 [1.91-2.24]	0.942	0
6	32713753	chr6:32713753:T:C	-	Intergenic	rs3957146	T>C	9	2.82x10-65	2.07 [1.91-2.24]	0.942	0
6	32713854	chr6:32713854:C:T	-	Intergenic	rs9275596	C>T	10	5.50x10-22	0.74 [0.69-0.78]	0.051	46.53
6	32714215	chr6:32714215:T:C	-	Intergenic	rs3998158	T>C	9	2.94x10-19	1.37 [1.28-1.46]	0.667	0
6	32714242	chr6:32714242:A:C	-	Intergenic	rs3998159	A>C	9	7.40x10-65	2.06 [1.91-2.23]	0.939	0
6	32714360	chr6:32714360:A:G	-	Intergenic	rs3957148	A>G	10	6.69x10-72	2.09 [1.94-2.26]	0.772	0
6	32714422	chr6:32714422:T:C	-	Intergenic	rs35122968	T>C	9	1.43x10-10	1.39 [1.26-1.53]	0.34	11.43
6	32714652	chr6:32714652:C:T	-	Intergenic	rs9275599	C>T	9	8.25x10-63	2.07 [1.91-2.25]	0.965	0
6	32714675	chr6:32714675:G:A	-	Intergenic	rs3104407	G>A	10	2.25x10-21	0.62 [0.56-0.68]	0.038	49.28
6	32715138	chr6:32715138:T:G	-	Intergenic	rs3997854	T>G	9	4.38x10-29	1.59 [1.47-1.72]	0.243	22.53

6	32715188	chr6:32715188:C:T	-	Intergenic	rs3997855	C>T	10	1.15x10-12	1.28 [1.20-1.36]	0.289	16.76
6	32715233	chr6:32715233:C:T	-	Intergenic	rs3873445	C>T	9	2.37x10-28	1.60 [1.47-1.73]	0.459	0
6	32715234	chr6:32715234:A:G	-	Intergenic	rs3873446	A>G	10	3.51x10-13	1.28 [1.20-1.36]	0.392	5.3
6	32715235	chr6:32715235:G:A	-	Intergenic	rs3873447	G>A	10	3.49x10-13	1.28 [1.20-1.36]	0.391	5.38
6	32715278	chr6:32715278:C:T	-	Intergenic	rs3873448	C>T	9	2.49x10-28	1.60 [1.47-1.73]	0.457	0
6	32715868	chr6:32715868:A:G	-	Intergenic	rs9275607	A>G	10	1.18x10-26	1.51 [1.40-1.62]	0.694	0
6	32715876	chr6:32715876:G:A	-	Intergenic	rs3104411	G>A	10	1.85x10-12	0.81 [0.76-0.86]	0.215	24.85
6	32715914	chr6:32715914:A:C	-	Intergenic	rs6935940	A>C	10	7.22x10-20	1.33 [1.25-1.40]	0.382	6.48
6	32715931	chr6:32715931:A:G	-	Intergenic	rs9275608	A>G	10	1.24x10-26	1.51 [1.40-1.62]	0.609	0
6	32715936	chr6:32715936:C:G	-	Intergenic	rs9275609	C>G	10	1.24x10-26	1.51 [1.40-1.62]	0.609	0
6	32715973	chr6:32715973:T:C	-	Intergenic	rs9275610	T>C	10	1.18x10-26	1.51 [1.40-1.62]	0.607	0
6	32715986	chr6:32715986:G:A	-	Intergenic	rs9275611	G>A	10	3.11x10-31	1.62 [1.50-1.75]	0.608	0
6	32716252	chr6:32716252:C:G	-	Intergenic	rs6936863	C>G	10	1.73x10-19	1.32 [1.25-1.40]	0.404	3.88
6	32716480	chr6:32716480:A:G	-	Intergenic	rs9275614	A>G	10	5.27x10-30	1.60 [1.48-1.73]	0.697	0
6	32716495	chr6:32716495:T:A	-	Intergenic	rs9275615	T>A	10	5.27x10-30	1.60 [1.48-1.73]	0.697	0
6	32716540	chr6:32716540:T:C	-	Intergenic	rs6916779	T>C	10	9.33x10-26	1.50 [1.39-1.61]	0.644	0
6	32716567	chr6:32716567:G:A	-	Intergenic	rs5024431	G>A	10	1.48x10-19	1.32 [1.25-1.40]	0.408	3.44
6	32716610	chr6:32716610:A:G	-	Intergenic	rs9275618	A>G	10	5.27x10-30	1.60 [1.48-1.73]	0.697	0
6	32716736	chr6:32716736:G:A	-	Intergenic	rs6457636	G>A	10	1.36x10-19	1.32 [1.25-1.40]	0.397	4.69
6	32716983	chr6:32716983:C:T	-	Intergenic	rs9275638	C>T	10	5.53x10-27	1.52 [1.41-1.64]	0.528	0
6	32717054	chr6:32717054:A:T	-	Intergenic	rs9275639	A>T	10	2.57x10-18	1.31 [1.24-1.39]	0.146	32.75
6	32717142	chr6:32717142:C:T	-	Intergenic	rs9275641	C>T	10	2.23x10-18	1.31 [1.24-1.39]	0.161	30.91
6	32717158	chr6:32717158:C:T	-	Intergenic	rs9275642	C>T	10	6.09x10-19	1.32 [1.25-1.40]	0.091	39.98
6	32717180	chr6:32717180:G:A	-	Intergenic	rs7738118	G>A	10	2.49x10-18	1.31 [1.24-1.39]	0.152	32.07
6	32717182	chr6:32717182:G:A	-	Intergenic	rs7738119	G>A	10	2.49x10-18	1.31 [1.24-1.39]	0.152	32.07
6	32717773	chr6:32717773:G:A	-	Intergenic	rs3916765	G>A	10	4.22x10-30	1.60 [1.48-1.73]	0.64	0
6	32719304	chr6:32719304:A:G	-	Intergenic	rs114121446	A>G	7	6.95x10-29	2.24 [1.96-2.56]	0.647	0
6	32752654	chr6:32752654:G:T	HLA-DQB2	Intergenic	rs9276521	G>T	8	5.73x10-10	0.72 [0.65-0.80]	0.497	0
6	32774318	chr6:32774318:A:T	-	Intergenic	rs9276610	A>T	7	1.25x10-09	0.69 [0.61-0.77]	0.586	0
6	32776058	chr6:32776058:C:T	-	Intergenic	rs9276627	C>T	10	8.19x10-12	1.39 [1.27-1.52]	0.863	0
6	32779239	chr6:32779239:C:A	-	Intergenic	rs34153000	C>A	10	2.40x10-09	1.48 [1.30-1.67]	0.293	16.32
6	32780252	chr6:32780252:T:G	-	Intergenic	rs35520662	T>G	10	2.40x10-09	1.48 [1.30-1.67]	0.293	16.32
6	32780813	chr6:32780813:C:T	-	Intergenic	rs34043227	C>T	10	2.40x10-09	1.48 [1.30-1.67]	0.293	16.32
6	32787099	chr6:32787099:T:C	-	Intergenic	rs116309362	T>C	6	9.76x10-23	2.29 [1.95-2.68]	0.883	0
6	32787400	chr6:32787400:T:C	-	Intergenic	rs34408145	T>C	10	2.28x10-09	1.48 [1.30-1.67]	0.304	15.11
6	32788964	chr6:32788964:A:G	-	Intergenic	rs9276702	A>G	7	4.14x10-10	0.69 [0.61-0.77]	0.533	0
6	32794748	chr6:32794748:A:G	-	Intergenic	rs35805736	A>G	8	1.59x10-09	1.52 [1.33-1.73]	0.468	0
6	32795729	chr6:32795729:A:G	-	Intergenic	rs35486885	A>G	10	2.48x10-09	1.48 [1.30-1.67]	0.306	14.92
6	32797405	chr6:32797405:G:A	-	Intergenic	rs7767167	G>A	7	5.32x10-10	0.69 [0.62-0.77]	0.542	0
6	32800338	chr6:32800338:A:G	-	Intergenic	rs34710970	A>G	10	3.46x10-09	1.47 [1.30-1.67]	0.312	14.21
6	32805720	chr6:32805720:G:T	-	Intergenic	rs6457681	G>T	10	4.36x10-15	1.29 [1.22-1.38]	0.351	9.96
6	32806639	chr6:32806639:C:T	-	Intergenic	rs35921062	C>T	10	3.46x10-09	1.47 [1.30-1.67]	0.312	14.21
6	32809218	chr6:32809218:T:C	HLA-DOB	Intergenic	rs34422230	T>C	8	2.19x10-09	1.51 [1.32-1.73]	0.476	0
6	32819105	chr6:32819105:T:C	HLA-DOB	Intergenic	rs3763355	T>C	9	7.16x10-20	1.63 [1.47-1.80]	0.816	0
6	32820219	chr6:32820219:C:T	HLA-DOB	Intergenic	rs17213756	C>T	10	3.66x10-09	1.47 [1.30-1.67]	0.309	14.55
6	32825109	chr6:32825109:T:A	TAP2	Intergenic	rs41316544	T>A	10	4.85x10-09	1.47 [1.29-1.66]	0.316	13.86

6	32836793	chr6:32836793:G:A	PSMB8	Intergenic	rs3819720	G>A	10	1.09x10-18	1.30 [1.23-1.38]	0.274	18.35
6	32837102	chr6:32837102:C:T	PSMB8	Intergenic	rs1871665	C>T	10	6.95x10-10	0.83 [0.79-0.88]	0.209	25.47
6	32837104	chr6:32837104:G:A	PSMB8	Intergenic	rs1871664	G>A	10	6.64x10-10	0.83 [0.79-0.88]	0.245	21.51
6	32837132	chr6:32837132:A:G	PSMB8	Intergenic	rs241425	A>G	10	3.38x10-10	0.83 [0.79-0.88]	0.186	28.13
6	32843852	chr6:32843852:G:T	TAP1	Intergenic	rs2071543	G>T	10	1.21x10-14	1.36 [1.26-1.47]	0.065	44.14
6	32861364	chr6:32861364:A:T	PSMB9	Intergenic	rs28724899	A>T	10	4.15x10-15	1.37 [1.27-1.48]	0.076	42.28
6	32863884	chr6:32863884:C:T	PSMB9	Intergenic	rs4959116	C>T	10	9.81x10-09	1.25 [1.16-1.34]	0.091	40.03
6	32870762	chr6:32870762:G:A	-	Intergenic	rs115493740	G>A	10	4.15x10-26	1.69 [1.54-1.85]	0.103	38.24
6	32875198	chr6:32875198:C:T	PPP1R2P1	Intergenic	rs6903433	C>T	10	5.12x10-15	1.37 [1.27-1.48]	0.103	38.31
6	32883655	chr6:32883655:G:A	PPP1R2P1	Intergenic	rs2857219	G>A	10	4.92x10-15	1.37 [1.27-1.48]	0.105	38
6	33814551	chr6:33814551:C:T	-	Intergenic	rs116276283	C>T	7	4.37x10-08	1.46 [1.28-1.67]	0.156	35.68
6	160679306	chr6:160679306:C:T	-	Intergenic	rs60553356	C>T	10	4.67x10-08	0.85 [0.81-0.90]	0.435	0.31
6	160679480	chr6:160679480:C:T	-	Intergenic	rs62439764	C>T	10	4.93x10-08	0.85 [0.81-0.90]	0.421	1.92
6	160694213	chr6:160694213:A:T	-	Intergenic	rs7772437	A>T	10	8.52x10-11	0.83 [0.79-0.88]	0.301	15.46
6	160696881	chr6:160696881:G:A	-	Intergenic	rs1009124	G>A	10	9.39x10-11	0.83 [0.79-0.88]	0.269	18.89
6	160698869	chr6:160698869:G:A	PLG	Intergenic	rs113525646	G>A	10	1.75x10-10	0.83 [0.79-0.88]	0.23	23.18
6	160699026	chr6:160699026:A:G	PLG	Intergenic	rs7452732	A>G	10	1.82x10-10	0.83 [0.79-0.88]	0.273	18.49
6	160699616	chr6:160699616:G:A	PLG	Intergenic	rs1965091	G>A	10	1.72x10-10	0.83 [0.79-0.88]	0.263	19.6
6	160700178	chr6:160700178:G:A	PLG	Intergenic	rs4252045	G>A	10	1.42x10-10	0.83 [0.79-0.88]	0.235	22.62
6	160700358	chr6:160700358:C:T	PLG	Intergenic	rs2144723	C>T	10	1.56x10-10	0.83 [0.79-0.88]	0.263	19.59
6	160809769	chr6:160809769:T:C	-	Intergenic	rs3846778	T>C	10	1.74x10-10	1.22 [1.15-1.29]	0.074	42.59
6	160862554	chr6:160862554:C:G	-	Intergenic	rs1247514	C>G	7	4.52x10-12	1.26 [1.18-1.34]	0.074	47.86
6	160863441	chr6:160863441:C:G	-	Intergenic	rs2255792	C>G	9	9.86x10-09	1.19 [1.12-1.26]	0.533	0
6	160863747	chr6:160863747:T:C	-	Intergenic	rs1937487	T>C	9	6.45x10-09	1.19 [1.12-1.26]	0.599	0
6	160863813	chr6:160863813:G:C	-	Intergenic	rs1937486	G>C	9	6.35x10-09	1.19 [1.12-1.26]	0.606	0
6	160863863	chr6:160863863:A:G	-	Intergenic	rs1937485	A>G	8	6.76x10-09	1.20 [1.13-1.27]	0.504	0
6	160865918	chr6:160865918:T:C	-	Intergenic	rs1937484	T>C	8	2.62x10-08	1.19 [1.12-1.26]	0.548	0
6	160866301	chr6:160866301:C:T	-	Intergenic	rs2465868	C>T	8	3.13x10-08	1.19 [1.12-1.26]	0.559	0
6	160867608	chr6:160867608:C:T	-	Intergenic	rs2489943	C>T	8	2.83x10-08	1.19 [1.12-1.26]	0.49	0
6	160868863	chr6:160868863:G:A	-	Intergenic	rs2953492	G>A	8	3.04x10-08	1.19 [1.12-1.26]	0.546	0
6	160869563	chr6:160869563:G:T	-	Intergenic	rs1782624	G>T	8	3.09x10-08	1.19 [1.12-1.26]	0.537	0
6	160869780	chr6:160869780:T:C	-	Intergenic	rs1782625	T>C	8	2.86x10-08	1.19 [1.12-1.26]	0.55	0
6	31138712	chr6:31138712:G:A	PSORS1C2	Intronic	rs1265096	G>A	9	4.70x10-10	1.44 [1.28-1.60]	0.953	0
6	31145336	chr6:31145336:C:A	CCHCR1	Intronic	rs1265075	C>A	9	1.18x10-08	1.38 [1.24-1.53]	0.913	0
6	31301442	chr6:31301442:C:T	LINC02571	Intronic	rs35192021	C>T	9	1.63x10-10	1.42 [1.28-1.58]	0.608	0
6	31615389	chr6:31615389:G:A	AIF1	Intronic	rs4711274	G>A	10	1.11x10-08	1.27 [1.17-1.37]	0.634	0
6	31616154	chr6:31616154:C:T	AIF1	Intronic	rs2269475	C>T	10	1.08x10-08	1.27 [1.17-1.37]	0.62	0
6	31633743	chr6:31633743:T:C	PRRC2A	Intronic	rs2242658	T>C	10	1.58x10-08	1.26 [1.17-1.37]	0.612	0
6	31643479	chr6:31643479:T:C	BAG6	Intronic	rs12524536	T>C	10	1.50x10-08	1.26 [1.17-1.37]	0.605	0
6	31644063	chr6:31644063:C:A	BAG6	Intronic	rs2077102	C>A	10	2.13x10-08	1.26 [1.16-1.37]	0.62	0
6	31645794	chr6:31645794:A:G	BAG6	Intronic	rs114919178	A>G	6	4.44x10-08	1.69 [1.40-2.03]	0.423	0
6	31651376	chr6:31651376:T:C	BAG6	Intronic	rs138039007	T>C	6	4.44x10-08	1.69 [1.40-2.03]	0.423	0
6	31689924	chr6:31689924:C:A	ABHD16A	Intronic	rs760294	C>A	9	2.30x10-08	1.31 [1.20-1.44]	0.723	0
6	31748659	chr6:31748659:A:G	MSH5	Intronic	rs116043458	A>G	10	1.76x10-27	2.19 [1.91-2.51]	0.694	0
6	32053184	chr6:32053184:G:C	TNXB	Intronic	rs34562262	G>C	8	2.13x10-08	1.49 [1.30-1.71]	0.348	10.62
6	32074545	chr6:32074545:T:C	TNXB	Intronic	rs3130286	T>C	10	3.25x10-11	0.78 [0.73-0.84]	0.86	0

6	32087709	chr6:32087709:G:A	TNXB	Intronic	rs137893789	G>A	8	2.14x10-08	1.49 [1.30-1.71]	0.348	10.62
6	32103860	chr6:32103860:T:C	TNXB	Intronic	rs4711286	T>C	10	6.88x10-11	1.21 [1.14-1.28]	0.656	0
6	32209300	chr6:32209300:A:T	NOTCH4	Intronic	rs116753971	A>T	10	1.17x10-35	2.30 [2.03-2.60]	0.822	0
6	32261141	chr6:32261141:T:C	TSBP1-AS1	Intronic	rs3130326	T>C	8	2.84x10-08	0.59 [0.50-0.71]	0.176	31.54
6	32261854	chr6:32261854:C:T	TSBP1-AS1	Intronic	rs6457524	C>T	8	2.84x10-08	0.59 [0.50-0.71]	0.176	31.54
6	32275311	chr6:32275311:A:G	TSBP1-AS1	Intronic	rs1559870	A>G	8	1.83x10-08	0.59 [0.50-0.71]	0.269	20.24
6	32275330	chr6:32275330:A:G	TSBP1-AS1	Intronic	rs1559871	A>G	8	1.83x10-08	0.59 [0.50-0.71]	0.269	20.24
6	32283360	chr6:32283360:A:G	TSBP1-AS1	Intronic	rs2222988	A>G	8	1.83x10-08	0.59 [0.50-0.71]	0.269	20.24
6	32297901	chr6:32297901:A:T	TSBP1	Intronic	rs517630	A>T	8	1.86x10-08	0.59 [0.49-0.71]	0.197	28.93
6	32302723	chr6:32302723:A:G	TSBP1	Intronic	rs477005	A>G	10	1.82x10-12	1.23 [1.16-1.30]	0.237	22.35
6	32303673	chr6:32303673:T:A	TSBP1	Intronic	rs491870	T>A	8	6.45x10-09	0.58 [0.49-0.70]	0.236	24.28
6	32305283	chr6:32305283:A:G	TSBP1	Intronic	rs522254	A>G	10	1.67x10-42	1.60 [1.50-1.71]	0.195	27.05
6	32305381	chr6:32305381:G:C	TSBP1	Intronic	rs544358	G>C	10	4.24x10-13	1.24 [1.17-1.31]	0.273	18.45
6	32307778	chr6:32307778:G:T	TSBP1	Intronic	rs480085	G>T	10	5.21x10-13	1.24 [1.17-1.31]	0.255	20.41
6	32308392	chr6:32308392:A:G	TSBP1	Intronic	rs540926	A>G	10	4.24x10-13	1.24 [1.17-1.31]	0.273	18.45
6	32308725	chr6:32308725:A:G	TSBP1	Intronic	rs1265776	A>G	10	4.24x10-13	1.24 [1.17-1.31]	0.273	18.45
6	32310489	chr6:32310489:A:G	TSBP1	Intronic	rs502626	A>G	10	4.24x10-13	1.24 [1.17-1.31]	0.273	18.45
6	32310858	chr6:32310858:G:A	TSBP1	Intronic	rs9268199	G>A	9	4.24x10-13	0.71 [0.65-0.77]	0.185	29.24
6	32314256	chr6:32314256:G:A	TSBP1	Intronic	rs547261	G>A	10	5.41x10-13	1.24 [1.17-1.31]	0.284	17.25
6	32315077	chr6:32315077:A:G	TSBP1	Intronic	rs6910071	A>G	10	2.60x10-42	1.60 [1.50-1.70]	0.226	23.57
6	32315617	chr6:32315617:A:G	TSBP1	Intronic	rs493136	A>G	8	6.33x10-09	0.58 [0.48-0.69]	0.163	33.16
6	32316646	chr6:32316646:A:T	TSBP1	Intronic	rs495140	A>T	10	5.85x10-13	1.24 [1.17-1.31]	0.287	16.93
6	32317173	chr6:32317173:T:A	TSBP1	Intronic	rs567828	T>A	10	5.85x10-13	1.24 [1.17-1.31]	0.287	16.93
6	32317471	chr6:32317471:G:A	TSBP1	Intronic	rs543928	G>A	10	1.59x10-13	0.80 [0.75-0.85]	0.143	33.13
6	32317548	chr6:32317548:C:T	TSBP1	Intronic	rs543131	C>T	8	6.45x10-09	0.58 [0.49-0.70]	0.236	24.28
6	32318102	chr6:32318102:G:A	TSBP1	Intronic	rs538473	G>A	9	4.00x10-12	0.65 [0.58-0.73]	0.075	43.98
6	32318325	chr6:32318325:T:A	TSBP1	Intronic	rs531094	T>A	10	6.29x10-13	1.24 [1.17-1.31]	0.274	18.41
6	32318475	chr6:32318475:G:A	TSBP1	Intronic	rs503042	G>A	8	6.45x10-09	0.58 [0.49-0.70]	0.236	24.28
6	32318785	chr6:32318785:C:T	TSBP1	Intronic	rs500279	C>T	8	6.33x10-09	0.58 [0.48-0.69]	0.163	33.16
6	32318903	chr6:32318903:G:A	TSBP1	Intronic	rs557539	G>A	10	5.58x10-13	1.24 [1.17-1.31]	0.241	21.96
6	32318984	chr6:32318984:T:G	TSBP1	Intronic	rs498422	T>G	9	2.12x10-10	0.58 [0.50-0.69]	0.32	13.67
6	32319207	chr6:32319207:T:G	TSBP1	Intronic	rs560324	T>G	10	5.02x10-13	1.24 [1.17-1.31]	0.243	21.77
6	32319719	chr6:32319719:A:G	TSBP1	Intronic	rs580921	A>G	10	1.27x10-14	1.26 [1.19-1.33]	0.206	25.77
6	32320099	chr6:32320099:T:G	TSBP1	Intronic	rs513095	T>G	8	4.85x10-09	0.60 [0.51-0.71]	0.323	13.74
6	32320264	chr6:32320264:T:C	TSBP1	Intronic	rs575765	T>C	10	5.37x10-13	1.24 [1.17-1.31]	0.284	17.25
6	32320413	chr6:32320413:T:C	TSBP1	Intronic	rs574710	T>C	10	5.58x10-13	1.24 [1.17-1.31]	0.241	21.96
6	32320685	chr6:32320685:A:C	TSBP1	Intronic	rs539703	A>C	10	2.76x10-13	1.24 [1.17-1.31]	0.288	16.82
6	32320787	chr6:32320787:T:G	TSBP1	Intronic	rs540639	T>G	10	5.58x10-13	1.24 [1.17-1.31]	0.241	21.96
6	32321028	chr6:32321028:T:C	TSBP1	Intronic	rs542525	T>C	10	5.58x10-13	1.24 [1.17-1.31]	0.241	21.96
6	32321155	chr6:32321155:T:A	TSBP1	Intronic	rs544100	T>A	10	4.93x10-13	1.24 [1.17-1.31]	0.281	17.57
6	32321463	chr6:32321463:C:T	TSBP1	Intronic	rs546857	C>T	10	2.09x10-12	1.23 [1.16-1.30]	0.245	21.57
6	32321541	chr6:32321541:T:C	TSBP1	Intronic	rs547077	T>C	10	5.58x10-13	1.24 [1.17-1.31]	0.241	21.96
6	32321987	chr6:32321987:C:T	TSBP1	Intronic	rs572730	C>T	10	5.00x10-13	1.24 [1.17-1.31]	0.281	17.56
6	32322971	chr6:32322971:A:G	TSBP1	Intronic	rs487649	A>G	10	4.86x10-13	1.24 [1.17-1.31]	0.28	17.7
6	32323177	chr6:32323177:A:G	TSBP1	Intronic	rs485774	A>G	10	4.93x10-13	1.24 [1.17-1.31]	0.281	17.57
6	32323413	chr6:32323413:A:G	TSBP1	Intronic	rs4963607	A>G	8	6.45x10-09	0.58 [0.49-0.70]	0.236	24.28

6	32324060	chr6:32324060:G:T	TSBP1	Intronic	rs523627	G>T	10	4.93x10-13	1.24 [1.17-1.31]	0.281	17.57
6	32324307	chr6:32324307:C:T	TSBP1	Intronic	rs525607	C>T	10	5.58x10-13	1.24 [1.17-1.31]	0.241	21.96
6	32324794	chr6:32324794:T:A	TSBP1	Intronic	rs74839053	T>A	10	7.45x10-13	1.23 [1.17-1.31]	0.308	14.7
6	32324938	chr6:32324938:G:A	TSBP1	Intronic	rs552339	G>A	10	4.93x10-13	1.24 [1.17-1.31]	0.281	17.57
6	32325041	chr6:32325041:C:A	TSBP1	Intronic	rs536693	C>A	8	6.45x10-09	0.58 [0.49-0.70]	0.236	24.28
6	32325375	chr6:32325375:A:G	TSBP1	Intronic	rs533885	A>G	10	2.25x10-13	1.24 [1.17-1.31]	0.373	7.44
6	32325657	chr6:32325657:A:C	TSBP1	Intronic	rs510181	A>C	10	5.35x10-13	1.24 [1.17-1.31]	0.243	21.79
6	32325821	chr6:32325821:A:T	TSBP1	Intronic	rs471081	A>T	10	5.24x10-13	1.24 [1.17-1.31]	0.203	26.1
6	32325894	chr6:32325894:G:A	TSBP1	Intronic	rs471964	G>A	10	2.98x10-11	1.22 [1.15-1.29]	0.282	17.51
6	32326240	chr6:32326240:T:C	TSBP1	Intronic	rs504703	T>C	10	4.30x10-13	1.24 [1.17-1.31]	0.279	17.8
6	32326415	chr6:32326415:G:A	TSBP1	Intronic	rs476584	G>A	10	5.52x10-13	1.24 [1.17-1.31]	0.223	23.89
6	32326789	chr6:32326789:G:A	TSBP1	Intronic	rs501545	G>A	10	4.13x10-13	1.24 [1.17-1.31]	0.283	17.42
6	32326935	chr6:32326935:A:G	TSBP1	Intronic	rs476885	A>G	10	4.13x10-13	1.24 [1.17-1.31]	0.283	17.42
6	32327066	chr6:32327066:T:C	TSBP1	Intronic	rs504203	T>C	10	4.25x10-13	1.24 [1.17-1.31]	0.278	17.89
6	32327215	chr6:32327215:G:A	TSBP1	Intronic	rs505274	G>A	10	4.13x10-13	1.24 [1.17-1.31]	0.283	17.42
6	32327573	chr6:32327573:G:A	TSBP1	Intronic	rs508805	G>A	10	4.13x10-13	1.24 [1.17-1.31]	0.283	17.42
6	32327580	chr6:32327580:G:A	TSBP1	Intronic	rs524578	G>A	10	4.20x10-13	1.24 [1.17-1.31]	0.24	22.02
6	32327776	chr6:32327776:G:C	TSBP1	Intronic	rs4424116	G>C	10	4.36x10-13	1.24 [1.17-1.31]	0.284	17.26
6	32327917	chr6:32327917:A:G	TSBP1	Intronic	rs6913605	A>G	10	2.33x10-13	1.24 [1.17-1.31]	0.336	11.55
6	32327970	chr6:32327970:T:A	TSBP1	Intronic	rs11752503	T>A	10	1.39x10-13	1.24 [1.18-1.31]	0.358	9.18
6	32328051	chr6:32328051:A:G	TSBP1	Intronic	rs6457542	A>G	10	1.73x10-13	1.24 [1.17-1.31]	0.373	7.47
6	32328252	chr6:32328252:T:G	TSBP1	Intronic	rs6457543	T>G	10	2.65x10-12	1.23 [1.16-1.30]	0.231	23
6	32328280	chr6:32328280:C:T	TSBP1	Intronic	rs6457544	C>T	10	2.77x10-12	1.23 [1.16-1.30]	0.225	23.67
6	32328539	chr6:32328539:C:T	TSBP1	Intronic	rs12528615	C>T	10	4.00x10-13	1.24 [1.17-1.31]	0.276	18.16
6	32328598	chr6:32328598:G:A	TSBP1	Intronic	rs10947252	G>A	10	4.00x10-13	1.24 [1.17-1.31]	0.276	18.16
6	32328778	chr6:32328778:G:C	TSBP1	Intronic	rs10947253	G>C	10	4.00x10-13	1.24 [1.17-1.31]	0.276	18.16
6	32329003	chr6:32329003:C:T	TSBP1	Intronic	rs9348880	C>T	10	4.52x10-13	1.24 [1.17-1.31]	0.24	22.1
6	32329065	chr6:32329065:C:G	TSBP1	Intronic	rs9348881	C>G	10	4.54x10-13	1.24 [1.17-1.31]	0.241	22
6	32329347	chr6:32329347:A:T	TSBP1	Intronic	rs9394086	A>T	10	4.06x10-13	1.24 [1.17-1.31]	0.275	18.29
6	32329379	chr6:32329379:T:C	TSBP1	Intronic	rs9368711	T>C	10	3.92x10-13	1.24 [1.17-1.31]	0.282	17.48
6	32329551	chr6:32329551:G:A	TSBP1	Intronic	rs9368712	G>A	10	4.03x10-13	1.24 [1.17-1.31]	0.266	19.29
6	32329560	chr6:32329560:T:C	TSBP1	Intronic	rs9368713	T>C	10	4.00x10-13	1.24 [1.17-1.31]	0.276	18.16
6	32329564	chr6:32329564:G:A	TSBP1	Intronic	rs9368714	G>A	10	6.14x10-20	1.32 [1.24-1.39]	0.115	36.64
6	32329640	chr6:32329640:G:A	TSBP1	Intronic	rs9380289	G>A	10	3.87x10-13	1.24 [1.17-1.31]	0.279	17.88
6	32329883	chr6:32329883:C:G	TSBP1	Intronic	rs75052715	C>G	10	3.63x10-13	1.24 [1.17-1.31]	0.279	17.8
6	32329913	chr6:32329913:C:T	TSBP1	Intronic	rs9296021	C>T	10	4.00x10-13	1.24 [1.17-1.31]	0.276	18.16
6	32330178	chr6:32330178:G:A	TSBP1	Intronic	rs9296022	G>A	10	4.00x10-13	1.24 [1.17-1.31]	0.276	18.16
6	32331938	chr6:32331938:G:A	TSBP1	Intronic	rs742582	G>A	10	3.94x10-13	1.24 [1.17-1.31]	0.275	18.29
6	32332096	chr6:32332096:G:T	TSBP1	Intronic	rs910052	G>T	10	4.00x10-13	1.24 [1.17-1.31]	0.276	18.16
6	32332597	chr6:32332597:T:G	TSBP1	Intronic	rs1076711	T>G	10	3.92x10-13	1.24 [1.17-1.31]	0.276	18.15
6	32332693	chr6:32332693:T:G	TSBP1	Intronic	rs761190	T>G	10	4.03x10-13	1.24 [1.17-1.31]	0.276	18.15
6	32333071	chr6:32333071:T:C	TSBP1	Intronic	rs9394087	T>C	10	3.67x10-13	1.24 [1.17-1.31]	0.238	22.33
6	32333512	chr6:32333512:T:C	TSBP1	Intronic	rs9366793	T>C	10	4.39x10-13	1.24 [1.17-1.31]	0.237	22.35
6	32333545	chr6:32333545:C:T	TSBP1	Intronic	rs9348882	C>T	10	4.00x10-13	1.24 [1.17-1.31]	0.276	18.16
6	32333675	chr6:32333675:C:G	TSBP1	Intronic	rs9380290	C>G	10	4.68x10-13	1.24 [1.17-1.31]	0.24	22.08
6	32333737	chr6:32333737:G:A	TSBP1	Intronic	rs9357140	G>A	10	4.00x10-13	1.24 [1.17-1.31]	0.276	18.16

6	32334027	chr6:32334027:G:C	TSBP1	Intronic	rs9391777	G>C	10	4.00x10-13	1.24 [1.17-1.31]	0.276	18.16
6	32334268	chr6:32334268:A:C	TSBP1	Intronic	rs1474729	A>C	10	3.61x10-13	1.24 [1.17-1.31]	0.263	19.53
6	32334290	chr6:32334290:C:T	TSBP1	Intronic	rs1474728	C>T	10	3.36x10-13	1.24 [1.17-1.31]	0.261	19.82
6	32334762	chr6:32334762:A:G	TSBP1	Intronic	rs9405091	A>G	10	3.36x10-13	1.24 [1.17-1.31]	0.261	19.82
6	32334764	chr6:32334764:T:G	TSBP1	Intronic	rs9378257	T>G	10	3.36x10-13	1.24 [1.17-1.31]	0.261	19.82
6	32334951	chr6:32334951:T:G	TSBP1	Intronic	rs9405092	T>G	10	3.19x10-13	1.24 [1.17-1.31]	0.26	19.92
6	32334964	chr6:32334964:G:T	TSBP1	Intronic	rs9405093	G>T	10	3.36x10-13	1.24 [1.17-1.31]	0.261	19.82
6	32334980	chr6:32334980:G:A	TSBP1	Intronic	rs9378258	G>A	10	3.36x10-13	1.24 [1.17-1.31]	0.261	19.82
6	32334999	chr6:32334999:A:G	TSBP1	Intronic	rs1265755	A>G	8	6.71x10-09	0.58 [0.49-0.70]	0.239	23.91
6	32335291	chr6:32335291:G:A	TSBP1	Intronic	rs10807100	G>A	10	3.36x10-13	1.24 [1.17-1.31]	0.261	19.82
6	32335734	chr6:32335734:G:A	TSBP1	Intronic	rs6929776	G>A	10	3.75x10-13	1.24 [1.17-1.31]	0.224	23.83
6	32336071	chr6:32336071:G:A	TSBP1	Intronic	rs28361060	G>A	10	1.33x10-42	1.60 [1.50-1.71]	0.225	23.7
6	32336254	chr6:32336254:G:A	TSBP1	Intronic	rs6930681	G>A	10	3.36x10-13	1.24 [1.17-1.31]	0.261	19.82
6	32336445	chr6:32336445:C:G	TSBP1	Intronic	rs9366794	C>G	10	3.36x10-13	1.24 [1.17-1.31]	0.261	19.82
6	32337252	chr6:32337252:T:A	TSBP1	Intronic	rs12194915	T>A	10	2.49x10-13	1.24 [1.17-1.31]	0.251	20.89
6	32337348	chr6:32337348:C:T	TSBP1	Intronic	rs973037	C>T	10	2.83x10-13	1.24 [1.17-1.31]	0.255	20.43
6	32337365	chr6:32337365:C:T	TSBP1	Intronic	rs2022535	C>T	10	3.44x10-13	1.24 [1.17-1.31]	0.259	19.99
6	32337366	chr6:32337366:C:G	TSBP1	Intronic	rs973036	C>G	10	3.23x10-13	1.24 [1.17-1.31]	0.257	20.26
6	32337593	chr6:32337593:T:G	TSBP1	Intronic	rs926594	T>G	10	3.36x10-13	1.24 [1.17-1.31]	0.261	19.82
6	32337913	chr6:32337913:C:A	TSBP1	Intronic	rs926591	C>A	10	3.36x10-13	1.24 [1.17-1.31]	0.261	19.82
6	32338245	chr6:32338245:G:A	TSBP1	Intronic	rs9368715	G>A	10	2.95x10-13	1.24 [1.17-1.31]	0.258	20.14
6	32338313	chr6:32338313:G:A	TSBP1	Intronic	rs9368716	G>A	10	8.18x10-17	0.79 [0.74-0.83]	0.216	24.75
6	32338485	chr6:32338485:T:C	TSBP1	Intronic	rs3817982	T>C	10	3.36x10-13	1.24 [1.17-1.31]	0.261	19.82
6	32339360	chr6:32339360:T:C	TSBP1	Intronic	rs2022534	T>C	10	3.36x10-13	1.24 [1.17-1.31]	0.261	19.82
6	32339483	chr6:32339483:T:G	TSBP1	Intronic	rs2022533	T>G	10	3.28x10-13	1.24 [1.17-1.31]	0.228	23.35
6	32340092	chr6:32340092:T:C	TSBP1	Intronic	rs1033498	T>C	10	3.36x10-13	1.24 [1.17-1.31]	0.261	19.82
6	32340285	chr6:32340285:A:G	TSBP1	Intronic	rs1033497	A>G	10	3.31x10-13	1.24 [1.17-1.31]	0.259	19.95
6	32340329	chr6:32340329:G:C	TSBP1	Intronic	rs1033496	G>C	10	3.36x10-13	1.24 [1.17-1.31]	0.261	19.82
6	32340568	chr6:32340568:T:G	TSBP1	Intronic	rs2395144	T>G	10	3.36x10-13	1.24 [1.17-1.31]	0.261	19.82
6	32340865	chr6:32340865:G:A	TSBP1	Intronic	rs4469285	G>A	10	3.36x10-13	1.24 [1.17-1.31]	0.261	19.82
6	32340913	chr6:32340913:A:G	TSBP1	Intronic	rs7453102	A>G	10	2.98x10-13	1.24 [1.17-1.31]	0.253	20.61
6	32340957	chr6:32340957:A:G	TSBP1	Intronic	rs2179579	A>G	10	3.13x10-13	1.24 [1.17-1.31]	0.258	20.12
6	32340993	chr6:32340993:G:A	TSBP1	Intronic	rs2179578	G>A	10	3.05x10-13	1.24 [1.17-1.31]	0.264	19.48
6	32341226	chr6:32341226:A:T	TSBP1	Intronic	rs2143468	A>T	10	3.36x10-13	1.24 [1.17-1.31]	0.261	19.82
6	32341519	chr6:32341519:C:G	TSBP1	Intronic	rs2143467	C>G	10	3.36x10-13	1.24 [1.17-1.31]	0.261	19.82
6	32341546	chr6:32341546:C:T	TSBP1	Intronic	rs2143466	C>T	10	1.66x10-14	1.25 [1.18-1.32]	0.496	0
6	32341575	chr6:32341575:T:C	TSBP1	Intronic	rs2143465	T>C	10	3.36x10-13	1.24 [1.17-1.31]	0.261	19.82
6	32341657	chr6:32341657:T:C	TSBP1	Intronic	rs2143464	T>C	10	3.36x10-13	1.24 [1.17-1.31]	0.261	19.82
6	32342087	chr6:32342087:C:T	TSBP1	Intronic	rs9268256	C>T	10	3.36x10-13	1.24 [1.17-1.31]	0.261	19.82
6	32342326	chr6:32342326:G:A	TSBP1	Intronic	rs9268257	G>A	10	3.36x10-13	1.24 [1.17-1.31]	0.261	19.82
6	32342907	chr6:32342907:G:A	TSBP1	Intronic	rs9268258	G>A	10	3.36x10-13	1.24 [1.17-1.31]	0.261	19.82
6	32343026	chr6:32343026:G:A	TSBP1	Intronic	rs9268259	G>A	10	3.36x10-13	1.24 [1.17-1.31]	0.261	19.82
6	32343190	chr6:32343190:C:T	TSBP1	Intronic	rs9268260	C>T	10	3.27x10-13	1.24 [1.17-1.31]	0.268	18.97
6	32343209	chr6:32343209:C:G	TSBP1	Intronic	rs9268261	C>G	10	3.36x10-13	1.24 [1.17-1.31]	0.261	19.82
6	32343371	chr6:32343371:C:G	TSBP1	Intronic	rs1555117	C>G	10	3.36x10-13	1.24 [1.17-1.31]	0.261	19.82
6	32343682	chr6:32343682:G:A	TSBP1	Intronic	rs3132954	G>A	10	3.18x10-10	0.83 [0.78-0.88]	0.638	0

6	32344094	chr6:32344094:T:A	TSBP1	Intronic	rs2395145	T>A	10	3.08x10-13	1.24 [1.17-1.31]	0.258	20.13
6	32344098	chr6:32344098:T:A	TSBP1	Intronic	rs2395146	T>A	10	3.08x10-13	1.24 [1.17-1.31]	0.258	20.13
6	32344718	chr6:32344718:A:G	TSBP1	Intronic	rs9268267	A>G	8	7.01x10-09	0.58 [0.49-0.70]	0.238	24.05
6	32344963	chr6:32344963:C:A	TSBP1	Intronic	rs1555116	C>A	10	3.31x10-13	1.24 [1.17-1.31]	0.259	19.95
6	32345019	chr6:32345019:A:G	TSBP1	Intronic	rs4576282	A>G	10	3.36x10-13	1.24 [1.17-1.31]	0.261	19.82
6	32345106	chr6:32345106:C:T	TSBP1	Intronic	rs9268271	C>T	10	2.79x10-13	1.24 [1.17-1.31]	0.257	20.27
6	32345233	chr6:32345233:C:T	TSBP1	Intronic	rs4959024	C>T	10	3.85x10-13	1.24 [1.17-1.31]	0.265	19.37
6	32345234	chr6:32345234:T:G	TSBP1	Intronic	rs4959092	T>G	10	3.85x10-13	1.24 [1.17-1.31]	0.265	19.37
6	32345320	chr6:32345320:T:C	TSBP1	Intronic	rs4959093	T>C	10	3.31x10-13	1.24 [1.17-1.31]	0.259	19.95
6	32345359	chr6:32345359:C:T	TSBP1	Intronic	rs4959094	C>T	10	3.31x10-13	1.24 [1.17-1.31]	0.259	19.95
6	32345366	chr6:32345366:A:C	TSBP1	Intronic	rs4959025	A>C	10	3.31x10-13	1.24 [1.17-1.31]	0.259	19.95
6	32345684	chr6:32345684:G:T	TSBP1	Intronic	rs4959026	G>T	10	3.72x10-13	1.24 [1.17-1.31]	0.261	19.77
6	32346899	chr6:32346899:G:A	TSBP1	Intronic	rs4959096	G>A	10	3.08x10-13	1.24 [1.17-1.31]	0.257	20.25
6	32347396	chr6:32347396:T:C	TSBP1	Intronic	rs9268283	T>C	10	3.08x10-13	1.24 [1.17-1.31]	0.257	20.25
6	32347439	chr6:32347439:G:A	TSBP1	Intronic	rs9268284	G>A	10	2.62x10-13	1.24 [1.17-1.31]	0.25	20.96
6	32347780	chr6:32347780:T:C	TSBP1	Intronic	rs9268285	T>C	10	3.40x10-13	1.24 [1.17-1.31]	0.26	19.9
6	32347816	chr6:32347816:C:G	TSBP1	Intronic	rs9268286	C>G	10	3.17x10-13	1.24 [1.17-1.31]	0.257	20.19
6	32349350	chr6:32349350:G:A	TSBP1	Intronic	rs2076542	G>A	10	3.36x10-13	1.24 [1.17-1.31]	0.26	19.95
6	32349438	chr6:32349438:T:C	TSBP1	Intronic	rs2076541	T>C	10	3.08x10-13	1.24 [1.17-1.31]	0.257	20.25
6	32349499	chr6:32349499:T:C	TSBP1	Intronic	rs2076540	T>C	10	1.39x10-12	1.23 [1.16-1.30]	0.222	24.07
6	32349694	chr6:32349694:A:C	TSBP1	Intronic	rs2076538	A>C	10	3.28x10-13	1.24 [1.17-1.31]	0.259	19.97
6	32350259	chr6:32350259:A:G	TSBP1	Intronic	rs761187	A>G	10	1.29x10-12	1.23 [1.17-1.30]	0.219	24.34
6	32350952	chr6:32350952:A:G	TSBP1	Intronic	rs2143463	A>G	10	3.31x10-13	1.24 [1.17-1.31]	0.259	19.95
6	32351587	chr6:32351587:C:T	TSBP1	Intronic	rs1265763	C>T	10	3.83x10-16	0.73 [0.68-0.79]	0.269	18.96
6	32351860	chr6:32351860:G:A	TSBP1	Intronic	rs9268301	G>A	10	5.17x10-20	1.32 [1.24-1.39]	0.109	37.54
6	32353338	chr6:32353338:C:A	TSBP1	Intronic	rs1265762	C>A	10	3.99x10-20	0.76 [0.72-0.80]	0.238	22.32
6	32353820	chr6:32353820:A:G	TSBP1	Intronic	rs1265761	A>G	8	7.01x10-09	0.58 [0.49-0.70]	0.238	24.05
6	32354095	chr6:32354095:T:C	TSBP1	Intronic	rs1265760	T>C	10	3.78x10-20	0.76 [0.72-0.80]	0.236	22.47
6	32354616	chr6:32354616:C:T	TSBP1	Intronic	rs1265759	C>T	10	3.99x10-20	0.76 [0.72-0.80]	0.238	22.32
6	32355752	chr6:32355752:A:G	TSBP1	Intronic	rs1265758	A>G	10	2.19x10-18	0.77 [0.73-0.81]	0.223	23.95
6	32356767	chr6:32356767:A:G	TSBP1	Intronic	rs2206618	A>G	10	3.04x10-16	0.73 [0.68-0.79]	0.266	19.24
6	32356864	chr6:32356864:A:G	TSBP1	Intronic	rs2223638	A>G	10	3.04x10-16	0.73 [0.68-0.79]	0.266	19.24
6	32357040	chr6:32357040:C:T	TSBP1	Intronic	rs9268302	C>T	10	3.04x10-16	0.73 [0.68-0.79]	0.266	19.24
6	32357168	chr6:32357168:G:A	TSBP1	Intronic	rs6907322	G>A	10	1.44x10-14	0.74 [0.69-0.80]	0.225	23.73
6	32357361	chr6:32357361:A:G	TSBP1	Intronic	rs6907226	A>G	10	3.04x10-16	0.73 [0.68-0.79]	0.266	19.24
6	32357622	chr6:32357622:T:C	TSBP1	Intronic	rs6930477	T>C	10	3.04x10-16	0.73 [0.68-0.79]	0.266	19.24
6	32357930	chr6:32357930:C:A	TSBP1	Intronic	rs3129908	C>A	10	2.27x10-18	0.77 [0.73-0.81]	0.224	23.81
6	32357933	chr6:32357933:G:A	TSBP1	Intronic	rs3129909	G>A	10	2.34x10-18	0.77 [0.73-0.81]	0.225	23.71
6	32358268	chr6:32358268:G:A	TSBP1	Intronic	rs2395150	G>A	10	2.14x10-18	0.77 [0.73-0.81]	0.223	23.97
6	32358388	chr6:32358388:G:A	TSBP1	Intronic	rs9268303	G>A	10	1.44x10-14	0.74 [0.69-0.80]	0.225	23.73
6	32358463	chr6:32358463:G:A	TSBP1	Intronic	rs6457548	G>A	10	2.27x10-18	0.77 [0.73-0.81]	0.224	23.81
6	32358754	chr6:32358754:T:C	TSBP1	Intronic	rs9268307	T>C	10	3.24x10-13	1.24 [1.17-1.31]	0.259	19.99
6	32358876	chr6:32358876:C:T	TSBP1	Intronic	rs7775581	C>T	10	4.09x10-20	0.76 [0.72-0.80]	0.239	22.23
6	32359298	chr6:32359298:C:G	TSBP1	Intronic	rs6457550	C>G	10	3.11x10-16	0.73 [0.68-0.79]	0.266	19.21
6	32359950	chr6:32359950:C:T	TSBP1	Intronic	rs6904608	C>T	10	4.09x10-20	0.76 [0.72-0.80]	0.239	22.23
6	32360004	chr6:32360004:C:T	TSBP1	Intronic	rs6904636	C>T	10	4.09x10-20	0.76 [0.72-0.80]	0.239	22.23

6	32360062	chr6:32360062:A:G	TSBP1	Intronic	rs9268314	A>G	10	3.53x10-16	0.73 [0.68-0.79]	0.275	18.28
6	32360192	chr6:32360192:C:T	TSBP1	Intronic	rs9268315	C>T	10	3.11x10-16	0.73 [0.68-0.79]	0.266	19.21
6	32360460	chr6:32360460:A:T	TSBP1	Intronic	rs4389802	A>T	10	3.11x10-16	0.73 [0.68-0.79]	0.266	19.21
6	32360519	chr6:32360519:T:C	TSBP1	Intronic	rs3129910	T>C	10	4.09x10-20	0.76 [0.72-0.80]	0.239	22.23
6	32360626	chr6:32360626:C:T	TSBP1	Intronic	rs3129911	C>T	10	4.09x10-20	0.76 [0.72-0.80]	0.239	22.23
6	32361118	chr6:32361118:G:A	TSBP1	Intronic	rs7775978	G>A	10	3.65x10-20	0.76 [0.72-0.80]	0.235	22.61
6	32361187	chr6:32361187:T:C	TSBP1	Intronic	rs7776257	T>C	10	3.89x10-20	0.76 [0.72-0.80]	0.237	22.43
6	32361867	chr6:32361867:C:T	TSBP1	Intronic	rs7763071	C>T	10	3.92x10-20	0.76 [0.72-0.80]	0.237	22.41
6	32362244	chr6:32362244:G:A	TSBP1	Intronic	rs9268323	G>A	10	1.67x10-15	0.74 [0.69-0.79]	0.263	19.58
6	32362258	chr6:32362258:C:T	TSBP1	Intronic	rs9268324	C>T	10	3.85x10-16	0.73 [0.68-0.79]	0.266	19.25
6	32362315	chr6:32362315:C:T	TSBP1	Intronic	rs9268325	C>T	10	2.96x10-16	0.73 [0.68-0.79]	0.266	19.26
6	32362376	chr6:32362376:T:C	TSBP1	Intronic	rs9268326	T>C	10	3.29x10-13	1.24 [1.17-1.31]	0.26	19.86
6	32362387	chr6:32362387:C:G	TSBP1	Intronic	rs9268327	C>G	10	3.29x10-13	1.24 [1.17-1.31]	0.26	19.86
6	32362397	chr6:32362397:A:G	TSBP1	Intronic	rs9268329	A>G	10	3.29x10-13	1.24 [1.17-1.31]	0.26	19.86
6	32362529	chr6:32362529:A:G	TSBP1	Intronic	rs9268330	A>G	10	2.89x10-16	0.73 [0.68-0.79]	0.266	19.29
6	32362653	chr6:32362653:C:T	TSBP1	Intronic	rs7744155	C>T	10	2.89x10-16	0.73 [0.68-0.79]	0.266	19.29
6	32362874	chr6:32362874:C:T	TSBP1	Intronic	rs9268333	C>T	10	3.33x10-13	1.24 [1.17-1.31]	0.261	19.73
6	32363160	chr6:32363160:A:G	TSBP1	Intronic	rs6415129	A>G	10	2.92x10-16	0.73 [0.68-0.79]	0.266	19.29
6	32363292	chr6:32363292:T:C	TSBP1	Intronic	rs6934200	T>C	10	3.29x10-13	1.24 [1.17-1.31]	0.26	19.86
6	32363652	chr6:32363652:C:A	TSBP1	Intronic	rs6912095	C>A	10	3.19x10-13	1.24 [1.17-1.31]	0.258	20.08
6	32364039	chr6:32364039:C:T	TSBP1	Intronic	rs6917075	C>T	10	2.93x10-16	0.73 [0.68-0.79]	0.267	19.19
6	32364047	chr6:32364047:A:G	TSBP1	Intronic	rs9268343	A>G	10	3.29x10-13	1.24 [1.17-1.31]	0.26	19.86
6	32364060	chr6:32364060:C:T	TSBP1	Intronic	rs9268344	C>T	10	3.29x10-13	1.24 [1.17-1.31]	0.26	19.86
6	32364064	chr6:32364064:G:A	TSBP1	Intronic	rs9268345	G>A	10	3.29x10-13	1.24 [1.17-1.31]	0.26	19.86
6	32364150	chr6:32364150:C:G	TSBP1	Intronic	rs9268346	C>G	10	3.29x10-13	1.24 [1.17-1.31]	0.26	19.86
6	32364221	chr6:32364221:G:A	TSBP1	Intronic	rs9268347	G>A	10	2.82x10-16	0.73 [0.68-0.79]	0.265	19.31
6	32364787	chr6:32364787:T:C	TSBP1	Intronic	rs9268350	T>C	10	2.71x10-16	0.73 [0.68-0.79]	0.265	19.36
6	32364916	chr6:32364916:G:A	TSBP1	Intronic	rs9268351	G>A	8	7.32x10-09	0.58 [0.49-0.70]	0.241	23.66
6	32365013	chr6:32365013:C:A	TSBP1	Intronic	rs3129918	C>A	10	3.99x10-20	0.76 [0.72-0.80]	0.238	22.32
6	32365143	chr6:32365143:C:T	TSBP1	Intronic	rs9268356	C>T	10	2.77x10-16	0.73 [0.68-0.79]	0.265	19.33
6	32365219	chr6:32365219:C:G	TSBP1	Intronic	rs9268357	C>G	10	3.29x10-13	1.24 [1.17-1.31]	0.26	19.86
6	32365316	chr6:32365316:A:G	TSBP1	Intronic	rs3129921	A>G	10	3.99x10-20	0.76 [0.72-0.80]	0.238	22.32
6	32365321	chr6:32365321:C:T	TSBP1	Intronic	rs3129922	C>T	10	3.99x10-20	0.76 [0.72-0.80]	0.238	22.32
6	32365418	chr6:32365418:A:G	TSBP1	Intronic	rs3129923	A>G	10	4.09x10-20	0.76 [0.72-0.80]	0.239	22.23
6	32365466	chr6:32365466:G:A	TSBP1	Intronic	rs9268361	G>A	10	2.79x10-16	0.73 [0.68-0.79]	0.265	19.34
6	32365564	chr6:32365564:A:G	TSBP1	Intronic	rs9268362	A>G	10	5.31x10-42	1.60 [1.50-1.70]	0.244	21.59
6	32365633	chr6:32365633:A:C	TSBP1	Intronic	rs9268363	A>C	10	3.29x10-13	1.24 [1.17-1.31]	0.26	19.86
6	32365648	chr6:32365648:C:T	TSBP1	Intronic	rs9268364	C>T	10	2.85x10-16	0.73 [0.68-0.79]	0.266	19.29
6	32365662	chr6:32365662:G:T	TSBP1	Intronic	rs9268365	G>T	10	2.85x10-16	0.73 [0.68-0.79]	0.266	19.29
6	32365765	chr6:32365765:A:G	TSBP1	Intronic	rs9268367	A>G	10	3.38x10-16	0.73 [0.68-0.79]	0.265	19.4
6	32366447	chr6:32366447:C:G	TSBP1	Intronic	rs9268369	C>G	10	3.29x10-13	1.24 [1.17-1.31]	0.26	19.86
6	32366481	chr6:32366481:G:T	TSBP1	Intronic	rs9268370	G>T	10	3.29x10-13	1.24 [1.17-1.31]	0.26	19.86
6	32366625	chr6:32366625:G:C	TSBP1	Intronic	rs9268371	G>C	8	7.32x10-09	0.58 [0.49-0.70]	0.241	23.66
6	32366684	chr6:32366684:A:G	TSBP1	Intronic	rs9268372	A>G	8	8.15x10-09	0.58 [0.49-0.70]	0.244	23.22
6	32366712	chr6:32366712:G:A	TSBP1	Intronic	rs9268373	G>A	10	4.20x10-13	1.24 [1.17-1.31]	0.262	19.63
6	32366757	chr6:32366757:C:T	TSBP1	Intronic	rs7769717	C>T	10	1.71x10-16	0.73 [0.68-0.78]	0.241	21.92

6	32367851	chr6:32367851:A:T	TSBP1	Intronic	rs9268378	A>T	10	3.07x10-16	0.73 [0.68-0.79]	0.266	19.24
6	32368297	chr6:32368297:C:T	TSBP1	Intronic	rs2073046	C>T	10	3.29x10-13	1.24 [1.17-1.31]	0.26	19.86
6	32368740	chr6:32368740:A:G	TSBP1	Intronic	rs6903816	A>G	10	3.42x10-16	0.73 [0.68-0.79]	0.26	19.92
6	32369457	chr6:32369457:G:A	TSBP1	Intronic	rs9268386	G>A	10	3.24x10-13	1.24 [1.17-1.31]	0.259	19.99
6	32369590	chr6:32369590:T:G	TSBP1	Intronic	rs9268387	T>G	10	3.40x10-13	1.24 [1.17-1.31]	0.253	20.6
6	32369615	chr6:32369615:C:T	TSBP1	Intronic	rs1967437	C>T	10	2.95x10-16	0.73 [0.68-0.79]	0.266	19.25
6	32369853	chr6:32369853:G:A	TSBP1	Intronic	rs2273017	G>A	10	5.97x10-18	0.77 [0.73-0.82]	0.209	25.52
6	32370609	chr6:32370609:G:C	TSBP1	Intronic	rs9268394	G>C	10	4.97x10-13	1.24 [1.17-1.31]	0.268	18.97
6	32371209	chr6:32371209:C:T	TSBP1	Intronic	rs2073044	C>T	10	3.28x10-32	1.46 [1.38-1.55]	0.338	11.38
6	32371571	chr6:32371571:C:T	TSBP1	Intronic	rs2076536	C>T	10	1.53x10-29	0.70 [0.66-0.74]	0.079	41.75
6	32376954	chr6:32376954:A:G	TSBP1-AS1	Intronic	rs9268426	A>G	9	9.42x10-52	1.61 [1.52-1.71]	0.124	36.86
6	32377031	chr6:32377031:C:T	TSBP1-AS1	Intronic	rs9268427	C>T	9	9.42x10-52	1.61 [1.52-1.71]	0.124	36.86
6	32377196	chr6:32377196:G:T	TSBP1-AS1	Intronic	rs9268428	G>T	9	9.42x10-52	1.61 [1.52-1.71]	0.124	36.86
6	32377275	chr6:32377275:A:G	TSBP1-AS1	Intronic	rs9268429	A>G	9	9.89x10-52	1.61 [1.52-1.71]	0.123	36.99
6	32377666	chr6:32377666:G:T	TSBP1-AS1	Intronic	rs2894252	G>T	9	9.42x10-52	1.61 [1.52-1.71]	0.124	36.86
6	32377737	chr6:32377737:T:G	TSBP1-AS1	Intronic	rs9268431	T>G	9	9.42x10-52	1.61 [1.52-1.71]	0.124	36.86
6	32377818	chr6:32377818:C:G	TSBP1-AS1	Intronic	rs2395153	C>G	10	6.80x10-27	1.37 [1.29-1.44]	0.189	27.69
6	32377890	chr6:32377890:A:G	TSBP1-AS1	Intronic	rs9268432	A>G	9	9.42x10-52	1.61 [1.52-1.71]	0.124	36.86
6	32378114	chr6:32378114:T:G	TSBP1-AS1	Intronic	rs9268433	T>G	10	6.17x10-53	1.67 [1.57-1.78]	0.241	22
6	32378675	chr6:32378675:A:T	TSBP1-AS1	Intronic	rs9268434	A>T	10	3.26x10-24	1.34 [1.27-1.42]	0.12	35.96
6	32378714	chr6:32378714:G:A	TSBP1-AS1	Intronic	rs9268435	G>A	10	6.34x10-27	1.37 [1.30-1.45]	0.185	28.17
6	32378860	chr6:32378860:A:G	TSBP1-AS1	Intronic	rs9268436	A>G	9	8.71x10-52	1.61 [1.52-1.71]	0.121	37.17
6	32378885	chr6:32378885:A:T	TSBP1-AS1	Intronic	rs9268437	A>T	9	8.51x10-52	1.61 [1.52-1.71]	0.121	37.24
6	32378886	chr6:32378886:T:G	TSBP1-AS1	Intronic	rs9268438	T>G	9	8.71x10-52	1.61 [1.52-1.71]	0.121	37.17
6	32378924	chr6:32378924:G:A	TSBP1-AS1	Intronic	rs9268439	G>A	9	9.66x10-52	1.61 [1.52-1.71]	0.122	37.06
6	32379017	chr6:32379017:A:C	TSBP1-AS1	Intronic	rs1980495	A>C	9	1.33x10-51	1.61 [1.52-1.70]	0.146	34.04
6	32379239	chr6:32379239:A:G	TSBP1-AS1	Intronic	rs1980494	A>G	9	1.13x10-51	1.61 [1.52-1.71]	0.155	32.92
6	32379503	chr6:32379503:C:T	TSBP1-AS1	Intronic	rs8180659	C>T	9	1.18x10-51	1.61 [1.52-1.71]	0.153	33.07
6	32379713	chr6:32379713:C:T	TSBP1-AS1	Intronic	rs8180664	C>T	9	1.61x10-51	1.61 [1.52-1.70]	0.159	32.35
6	32379755	chr6:32379755:C:T	TSBP1-AS1	Intronic	rs8180673	C>T	9	1.53x10-51	1.61 [1.52-1.70]	0.157	32.68
6	32379756	chr6:32379756:A:G	TSBP1-AS1	Intronic	rs8180672	A>G	9	1.53x10-51	1.61 [1.52-1.70]	0.157	32.68
6	32379972	chr6:32379972:A:G	TSBP1-AS1	Intronic	rs9268446	A>G	9	1.16x10-51	1.61 [1.52-1.71]	0.155	32.84
6	32380054	chr6:32380054:T:C	TSBP1-AS1	Intronic	rs9268447	T>C	9	1.18x10-51	1.61 [1.52-1.71]	0.153	33.07
6	32380120	chr6:32380120:C:T	TSBP1-AS1	Intronic	rs2395155	C>T	9	1.16x10-51	1.61 [1.52-1.71]	0.155	32.84
6	32380321	chr6:32380321:C:A	TSBP1-AS1	Intronic	rs2395156	C>A	9	1.18x10-51	1.61 [1.52-1.71]	0.153	33.07
6	32380368	chr6:32380368:A:G	TSBP1-AS1	Intronic	rs2395157	A>G	9	1.13x10-51	1.61 [1.52-1.71]	0.155	32.93
6	32380686	chr6:32380686:C:G	TSBP1-AS1	Intronic	rs9268448	C>G	9	1.21x10-51	1.61 [1.52-1.71]	0.154	32.99
6	32380690	chr6:32380690:G:A	TSBP1-AS1	Intronic	rs9268449	G>A	9	1.21x10-51	1.61 [1.52-1.71]	0.154	32.99
6	32380913	chr6:32380913:G:A	TSBP1-AS1	Intronic	rs9268450	G>A	9	1.16x10-51	1.61 [1.52-1.71]	0.155	32.84
6	32381309	chr6:32381309:T:C	TSBP1-AS1	Intronic	rs9268451	T>C	10	9.58x10-53	1.67 [1.57-1.77]	0.246	21.38
6	32381717	chr6:32381717:A:G	TSBP1-AS1	Intronic	rs9268452	A>G	9	1.21x10-51	1.61 [1.52-1.71]	0.154	32.99
6	32381794	chr6:32381794:T:C	TSBP1-AS1	Intronic	rs9268453	T>C	9	1.21x10-51	1.61 [1.52-1.71]	0.154	32.99
6	32381934	chr6:32381934:A:G	TSBP1-AS1	Intronic	rs9268454	A>G	9	1.21x10-51	1.61 [1.52-1.71]	0.154	32.99
6	32381995	chr6:32381995:C:T	TSBP1-AS1	Intronic	rs9268455	C>T	10	9.58x10-53	1.67 [1.57-1.77]	0.246	21.38
6	32382169	chr6:32382169:C:A	TSBP1-AS1	Intronic	rs9268456	C>A	9	1.21x10-51	1.61 [1.52-1.71]	0.154	32.99
6	32382259	chr6:32382259:A:G	TSBP1-AS1	Intronic	rs9268457	A>G	9	1.21x10-51	1.61 [1.52-1.71]	0.154	32.99

6	32382607	chr6:32382607:C:A	TSBP1-AS1	Intronic	rs9268458	C>A	9	6.62x10-51	1.60 [1.51-1.70]	0.204	26.98
6	32382999	chr6:32382999:G:A	TSBP1-AS1	Intronic	rs9268459	G>A	9	1.40x10-51	1.61 [1.52-1.70]	0.146	33.96
6	32383091	chr6:32383091:A:C	TSBP1-AS1	Intronic	rs4373382	A>C	10	6.93x10-24	1.34 [1.27-1.41]	0.103	38.24
6	32383506	chr6:32383506:T:C	TSBP1-AS1	Intronic	rs9268460	T>C	10	7.02x10-24	1.34 [1.27-1.41]	0.103	38.23
6	32384124	chr6:32384124:C:A	TSBP1-AS1	Intronic	rs9268461	C>A	9	1.05x10-51	1.61 [1.52-1.71]	0.154	33.03
6	32384214	chr6:32384214:C:A	TSBP1-AS1	Intronic	rs9268462	C>A	9	1.05x10-51	1.61 [1.52-1.71]	0.154	33.03
6	32384310	chr6:32384310:C:G	TSBP1-AS1	Intronic	rs3117101	C>G	10	1.44x10-12	0.70 [0.64-0.77]	0.826	0
6	32384443	chr6:32384443:A:G	TSBP1-AS1	Intronic	rs3117100	A>G	10	8.54x10-13	0.70 [0.64-0.77]	0.811	0
6	32385389	chr6:32385389:A:G	TSBP1-AS1	Intronic	rs9268468	A>G	10	1.02x10-18	0.75 [0.70-0.80]	0.054	45.98
6	32395750	chr6:32395750:C:T	BTNL2	Intronic	rs2076533	C>T	10	1.22x10-23	1.34 [1.27-1.41]	0.107	37.74
6	32396579	chr6:32396579:A:G	BTNL2	Intronic	rs9268481	A>G	9	1.78x10-51	1.61 [1.52-1.70]	0.155	32.85
6	32396890	chr6:32396890:A:G	BTNL2	Intronic	rs3117139	A>G	10	1.28x10-12	0.70 [0.64-0.77]	0.838	0
6	32397803	chr6:32397803:A:G	BTNL2	Intronic	rs3129954	A>G	10	8.74x10-19	0.75 [0.70-0.80]	0.052	46.34
6	32398063	chr6:32398063:T:C	BTNL2	Intronic	rs3129955	T>C	10	8.83x10-19	0.75 [0.70-0.80]	0.053	46.31
6	32399240	chr6:32399240:G:A	BTNL2	Intronic	rs3117116	G>A	10	1.89x10-12	0.71 [0.65-0.78]	0.803	0
6	32399920	chr6:32399920:C:G	BTNL2	Intronic	rs116522341	C>G	6	8.33x10-36	2.46 [2.15-2.81]	0.511	0
6	32399945	chr6:32399945:A:G	BTNL2	Intronic	rs2294880	A>G	9	2.17x10-51	1.61 [1.52-1.70]	0.142	34.49
6	32400000	chr6:32400000:A:T	BTNL2	Intronic	rs9268482	A>T	9	2.09x10-51	1.61 [1.52-1.70]	0.144	34.2
6	32400018	chr6:32400018:G:T	BTNL2	Intronic	rs2294878	G>T	10	1.82x10-19	1.30 [1.23-1.37]	0.198	26.74
6	32400070	chr6:32400070:T:C	BTNL2	Intronic	rs3817966	T>C	9	1.26x10-50	1.60 [1.51-1.69]	0.19	28.69
6	32400220	chr6:32400220:T:A	BTNL2	Intronic	rs3817964	T>A	8	8.91x10-28	1.88 [1.69-2.10]	0.486	0
6	32400310	chr6:32400310:T:C	BTNL2	Intronic	rs3817963	T>C	9	8.45x10-51	1.60 [1.51-1.70]	0.197	27.87
6	32400537	chr6:32400537:C:A	BTNL2	Intronic	rs3817962	C>A	9	1.75x10-51	1.61 [1.52-1.70]	0.148	33.76
6	32400659	chr6:32400659:A:T	BTNL2	Intronic	rs28359862	A>T	10	1.24x10-19	1.30 [1.23-1.37]	0.204	26.05
6	32401711	chr6:32401711:G:A	BTNL2	Intronic	rs3763305	G>A	8	1.04x10-27	1.88 [1.69-2.10]	0.494	0
6	32402839	chr6:32402839:T:C	BTNL2	Intronic	rs2076525	T>C	9	1.80x10-51	1.61 [1.52-1.70]	0.139	34.82
6	32402907	chr6:32402907:A:G	BTNL2	Intronic	rs2076524	A>G	9	2.30x10-51	1.61 [1.51-1.70]	0.134	35.58
6	32403058	chr6:32403058:T:C	BTNL2	Intronic	rs20765230	T>C	10	4.04x10-14	1.44 [1.31-1.58]	0.05	46.91
6	32403402	chr6:32403402:G:C	BTNL2	Intronic	rs2076522	G>C	9	1.41x10-51	1.61 [1.52-1.70]	0.14	34.77
6	32403492	chr6:32403492:C:T	BTNL2	Intronic	rs2076520	C>T	9	1.80x10-51	1.61 [1.52-1.70]	0.139	34.82
6	32403842	chr6:32403842:A:G	BTNL2	Intronic	rs3793126	A>G	9	2.31x10-51	1.61 [1.51-1.70]	0.142	34.44
6	32404138	chr6:32404138:C:T	BTNL2	Intronic	rs3793127	C>T	10	1.30x10-52	1.67 [1.57-1.77]	0.242	21.87
6	32405601	chr6:32405601:A:T	BTNL2	Intronic	rs3806155	A>T	5	1.30x10-10	1.74 [1.48-2.06]	0.805	0
6	32405921	chr6:32405921:G:T	BTNL2	Intronic	rs3806156	G>T	10	4.04x10-14	1.44 [1.31-1.58]	0.05	46.91
6	32406024	chr6:32406024:T:G	BTNL2	Intronic	rs3806157	T>G	10	1.91x10-40	1.48 [1.40-1.56]	0.056	45.65
6	32406354	chr6:32406354:C:G	BTNL2	Intronic	rs9268491	C>G	9	1.73x10-51	1.61 [1.52-1.70]	0.141	34.62
6	32406845	chr6:32406845:A:T	BTNL2	Intronic	rs3763307	A>T	9	1.80x10-51	1.61 [1.52-1.70]	0.139	34.82
6	32440235	chr6:32440235:G:A	HLA-DRA	Intronic	rs3129876	G>A	10	9.91x10-12	0.80 [0.75-0.85]	0.859	0
6	32440267	chr6:32440267:A:C	HLA-DRA	Intronic	rs9268644	A>C	10	2.96x10-28	0.71 [0.67-0.76]	0.145	32.91
6	32440820	chr6:32440820:G:A	HLA-DRA	Intronic	rs3129877	G>A	10	5.49x10-12	0.80 [0.75-0.85]	0.792	0
6	32440958	chr6:32440958:A:C	HLA-DRA	Intronic	rs3129878	A>C	10	1.51x10-11	0.80 [0.76-0.85]	0.759	0
6	32441130	chr6:32441130:G:A	HLA-DRA	Intronic	rs3129879	G>A	10	1.26x10-12	0.79 [0.74-0.84]	0.879	0
6	32441269	chr6:32441269:G:A	HLA-DRA	Intronic	rs9268651	G>A	7	5.70x10-09	0.64 [0.55-0.74]	0.735	0
6	32441281	chr6:32441281:T:C	HLA-DRA	Intronic	rs9268653	T>C	9	2.67x10-12	0.68 [0.61-0.76]	0.635	0
6	32441528	chr6:32441528:A:G	HLA-DRA	Intronic	rs6926374	A>G	10	1.28x10-25	0.74 [0.70-0.78]	0.113	36.97
6	32441707	chr6:32441707:C:T	HLA-DRA	Intronic	rs3129881	C>T	10	1.29x10-11	0.80 [0.75-0.85]	0.849	0

6	32441753	chr6:32441753:G:A	HLA-DRA	Intronic	rs3129882	G>A	10	2.76x10-19	0.77 [0.72-0.81]	0.188	27.84
6	32442004	chr6:32442004:C:T	HLA-DRA	Intronic	rs6931646	C>T	10	1.16x10-25	0.74 [0.70-0.78]	0.117	36.46
6	32442010	chr6:32442010:T:C	HLA-DRA	Intronic	rs6911419	T>C	10	1.28x10-25	0.74 [0.70-0.78]	0.113	36.97
6	32442939	chr6:32442939:G:A	HLA-DRA	Intronic	rs9268658	G>A	10	1.24x10-25	0.74 [0.70-0.78]	0.112	37.04
6	32443164	chr6:32443164:T:C	HLA-DRA	Intronic	rs9268659	T>C	10	7.69x10-09	0.84 [0.79-0.89]	0.448	0
6	32443210	chr6:32443210:A:G	HLA-DRA	Intronic	rs3135391	A>G	9	2.01x10-12	0.68 [0.61-0.75]	0.629	0
6	32443258	chr6:32443258:A:C	HLA-DRA	Intronic	rs8084	A>C	10	7.45x10-09	0.84 [0.79-0.89]	0.447	0
6	32443746	chr6:32443746:T:C	HLA-DRA	Intronic	rs2239804	T>C	10	1.28x10-25	0.74 [0.70-0.78]	0.113	36.97
6	32443949	chr6:32443949:G:A	HLA-DRA	Intronic	rs3129888	G>A	10	2.20x10-10	0.77 [0.72-0.84]	0.363	8.61
6	32444056	chr6:32444056:C:T	HLA-DRA	Intronic	rs2239803	C>T	10	5.55x10-23	0.75 [0.71-0.79]	0.129	34.85
6	32444069	chr6:32444069:C:G	HLA-DRA	Intronic	rs2239802	C>G	10	2.12x10-12	0.76 [0.71-0.82]	0.447	0
6	32444618	chr6:32444618:C:A	HLA-DRA	Intronic	rs3135390	C>A	10	2.20x10-10	0.77 [0.72-0.84]	0.363	8.61
6	32444621	chr6:32444621:C:T	HLA-DRA	Intronic	rs4935354	C>T	10	5.31x10-14	0.79 [0.75-0.84]	0.776	0
6	32460285	chr6:32460285:C:T	HLA-DRB9	Intronic	rs9268833	C>T	10	9.53x10-75	1.73 [1.64-1.83]	0.217	24.65
6	32460302	chr6:32460302:C:A	HLA-DRB9	Intronic	rs9268834	C>A	10	8.46x10-75	1.73 [1.64-1.83]	0.208	25.65
6	32460338	chr6:32460338:G:A	HLA-DRB9	Intronic	rs9268835	G>A	10	8.46x10-75	1.73 [1.64-1.83]	0.208	25.65
6	32460409	chr6:32460409:G:C	HLA-DRB9	Intronic	rs6923504	G>C	10	4.76x10-35	0.66 [0.62-0.71]	0.081	41.45
6	32460508	chr6:32460508:C:T	HLA-DRB9	Intronic	rs6903608	C>T	10	5.76x10-35	0.66 [0.62-0.71]	0.08	41.65
6	32460938	chr6:32460938:G:A	HLA-DRB9	Intronic	rs9268838	G>A	10	5.34x10-75	1.73 [1.64-1.83]	0.224	23.79
6	32460995	chr6:32460995:A:G	HLA-DRB9	Intronic	rs9268839	A>G	10	4.77x10-47	1.52 [1.44-1.61]	0.529	0
6	32461234	chr6:32461234:C:T	HLA-DRB9	Intronic	rs6919855	C>T	10	4.76x10-35	0.66 [0.62-0.71]	0.081	41.45
6	32461376	chr6:32461376:A:G	HLA-DRB9	Intronic	rs9268844	A>G	10	1.77x10-77	1.88 [1.77-2.00]	0.845	0
6	32461468	chr6:32461468:C:G	HLA-DRB9	Intronic	rs9268846	C>G	10	3.29x10-76	1.73 [1.63-1.82]	0.152	32.05
6	32461500	chr6:32461500:A:G	HLA-DRB9	Intronic	rs9268847	A>G	10	2.77x10-33	0.67 [0.63-0.71]	0.089	40.34
6	32461526	chr6:32461526:G:A	HLA-DRB9	Intronic	rs9268848	G>A	9	2.34x10-24	1.70 [1.54-1.87]	0.047	49.11
6	32461550	chr6:32461550:C:T	HLA-DRB9	Intronic	rs9268849	C>T	10	1.02x10-83	1.78 [1.68-1.88]	0.255	20.4
6	32461805	chr6:32461805:A:G	HLA-DRB9	Intronic	rs9268851	A>G	9	1.96x10-13	0.66 [0.60-0.74]	0.645	0
6	32461817	chr6:32461817:A:G	HLA-DRB9	Intronic	rs9268852	A>G	10	2.32x10-16	0.61 [0.54-0.68]	0.004	62.74
6	32461866	chr6:32461866:T:C	HLA-DRB9	Intronic	rs9268853	T>C	10	5.20x10-84	1.78 [1.68-1.88]	0.27	18.76
6	32461981	chr6:32461981:T:C	HLA-DRB9	Intronic	rs9268858	T>C	10	2.62x10-83	1.77 [1.68-1.87]	0.261	19.76
6	32462048	chr6:32462048:C:T	HLA-DRB9	Intronic	rs9268860	C>T	10	3.81x10-83	1.77 [1.68-1.87]	0.267	19.09
6	32462512	chr6:32462512:A:G	HLA-DRB9	Intronic	rs9268863	A>G	10	5.36x10-16	0.61 [0.54-0.69]	0.004	63.04
6	32462549	chr6:32462549:T:A	HLA-DRB9	Intronic	rs9268864	T>A	10	2.62x10-83	1.77 [1.68-1.87]	0.261	19.76
6	32463347	chr6:32463347:G:A	HLA-DRB9	Intronic	rs7747529	G>A	10	2.85x10-83	1.77 [1.68-1.87]	0.259	19.98
6	32463370	chr6:32463370:A:G	HLA-DRB9	Intronic	rs9268877	A>G	10	5.79x10-16	0.61 [0.54-0.69]	0.004	63.11
6	32463581	chr6:32463581:T:G	HLA-DRB9	Intronic	rs9268880	T>G	10	3.85x10-34	0.67 [0.63-0.71]	0.072	42.87
6	32463767	chr6:32463767:A:G	HLA-DRB9	Intronic	rs9256933	A>G	10	2.66x10-83	1.77 [1.68-1.87]	0.258	20.07
6	32463829	chr6:32463829:T:A	HLA-DRB9	Intronic	rs9268881	T>A	10	5.79x10-16	0.61 [0.54-0.69]	0.004	63.11
6	32463846	chr6:32463846:T:C	HLA-DRB9	Intronic	rs9268882	T>C	10	3.85x10-34	0.67 [0.63-0.71]	0.072	42.87
6	32463861	chr6:32463861:T:A	HLA-DRB9	Intronic	rs9268883	T>A	10	5.79x10-16	0.61 [0.54-0.69]	0.004	63.11
6	32463863	chr6:32463863:T:A	HLA-DRB9	Intronic	rs9268884	T>A	10	2.85x10-83	1.77 [1.68-1.87]	0.259	19.98
6	32464090	chr6:32464090:G:T	HLA-DRB9	Intronic	rs9268888	G>T	10	5.55x10-16	0.61 [0.54-0.69]	0.004	63.04
6	32464109	chr6:32464109:A:G	HLA-DRB9	Intronic	rs9268889	A>G	10	3.16x10-83	1.77 [1.68-1.87]	0.261	19.76
6	32464150	chr6:32464150:C:T	HLA-DRB9	Intronic	rs9268893	C>T	10	5.55x10-16	0.61 [0.54-0.69]	0.004	63.04
6	32464300	chr6:32464300:G:C	HLA-DRB9	Intronic	rs9268905	G>C	10	3.16x10-83	1.77 [1.68-1.87]	0.261	19.76
6	32464404	chr6:32464404:A:G	HLA-DRB9	Intronic	rs9268906	A>G	10	3.16x10-83	1.77 [1.68-1.87]	0.261	19.76

6	32464563	chr6:32464563:T:C	HLA-DRB9	Intronic	rs9268909	T>C	7	2.95x10-09	0.64 [0.55-0.74]	0.482	0
6	32464732	chr6:32464732:C:A	HLA-DRB9	Intronic	rs9268912	C>A	10	5.36x10-16	0.61 [0.54-0.69]	0.004	63.08
6	32464779	chr6:32464779:C:T	HLA-DRB9	Intronic	rs9268914	C>T	10	1.85x10-10	0.70 [0.63-0.78]	0.044	48.04
6	32465058	chr6:32465058:C:T	HLA-DRB9	Intronic	rs9268923	C>T	10	2.85x10-83	1.77 [1.68-1.87]	0.259	19.98
6	32465192	chr6:32465192:A:C	HLA-DRB9	Intronic	rs9268925	A>C	9	1.79x10-13	0.66 [0.60-0.74]	0.62	0
6	32465280	chr6:32465280:A:C	HLA-DRB9	Intronic	rs9469120	A>C	10	9.90x10-10	0.60 [0.51-0.70]	0.581	0
6	32465290	chr6:32465290:A:G	HLA-DRB9	Intronic	rs9268926	A>G	10	1.51x10-75	1.87 [1.75-1.99]	0.789	0
6	32465308	chr6:32465308:A:T	HLA-DRB9	Intronic	rs9268927	A>T	9	2.04x10-13	0.66 [0.60-0.74]	0.648	0
6	32465390	chr6:32465390:G:T	HLA-DRB9	Intronic	rs2395185	G>T	10	2.47x10-83	1.77 [1.68-1.87]	0.257	20.21
6	32465415	chr6:32465415:C:G	HLA-DRB9	Intronic	rs2395186	C>G	10	2.85x10-83	1.77 [1.68-1.87]	0.259	19.98
6	32465513	chr6:32465513:T:G	HLA-DRB9	Intronic	rs9268932	T>G	10	5.79x10-16	0.61 [0.54-0.69]	0.004	63.11
6	32465622	chr6:32465622:A:G	HLA-DRB9	Intronic	rs9268937	A>G	10	5.79x10-16	0.61 [0.54-0.69]	0.004	63.11
6	32465689	chr6:32465689:G:A	HLA-DRB9	Intronic	rs9469121	G>A	10	9.67x10-10	0.60 [0.51-0.70]	0.577	0
6	32466021	chr6:32466021:C:T	HLA-DRB9	Intronic	rs9268949	C>T	10	1.78x10-22	1.61 [1.47-1.77]	0.045	47.78
6	32466130	chr6:32466130:G:A	HLA-DRB9	Intronic	rs9268951	G>A	10	2.78x10-22	1.61 [1.47-1.77]	0.043	48.18
6	32466234	chr6:32466234:G:A	HLA-DRB9	Intronic	rs9268954	G>A	10	3.55x10-83	1.77 [1.68-1.87]	0.249	21.12
6	32466353	chr6:32466353:T:C	HLA-DRB9	Intronic	rs9268960	T>C	10	3.80x10-83	1.77 [1.68-1.87]	0.263	19.53
6	32466358	chr6:32466358:G:A	HLA-DRB9	Intronic	rs9268961	G>A	10	1.34x10-83	1.77 [1.68-1.87]	0.241	21.94
6	32466372	chr6:32466372:C:T	HLA-DRB9	Intronic	rs9268963	C>T	10	2.85x10-83	1.77 [1.68-1.87]	0.259	19.98
6	32466463	chr6:32466463:G:A	HLA-DRB9	Intronic	rs9268965	G>A	10	2.85x10-83	1.77 [1.68-1.87]	0.259	19.98
6	32466572	chr6:32466572:C:T	HLA-DRB9	Intronic	rs9268969	C>T	10	2.85x10-83	1.77 [1.68-1.87]	0.259	19.98
6	32466584	chr6:32466584:C:A	HLA-DRB9	Intronic	rs9268970	C>A	10	2.85x10-83	1.77 [1.68-1.87]	0.259	19.98
6	32467073	chr6:32467073:G:C	HLA-DRB9	Intronic	rs9391781	G>C	10	2.85x10-83	1.77 [1.68-1.87]	0.259	19.98
6	32467201	chr6:32467201:A:G	HLA-DRB9	Intronic	rs9268978	A>G	9	2.06x10-13	0.66 [0.60-0.74]	0.647	0
6	32467267	chr6:32467267:T:C	HLA-DRB9	Intronic	rs9268979	T>C	10	5.79x10-16	0.61 [0.54-0.69]	0.004	63.11
6	32467561	chr6:32467561:C:T	HLA-DRB9	Intronic	rs4947340	C>T	10	1.01x10-73	1.70 [1.61-1.79]	0.204	25.99
6	32467773	chr6:32467773:G:A	HLA-DRB9	Intronic	rs28895223	G>A	10	3.93x10-83	1.77 [1.68-1.87]	0.251	20.85
6	32469273	chr6:32469273:A:G	HLA-DRB9	Intronic	rs28895234	A>G	10	3.96x10-83	1.77 [1.68-1.87]	0.263	19.52
6	32469383	chr6:32469383:T:A	HLA-DRB9	Intronic	rs28895235	T>A	10	1.96x10-80	1.75 [1.66-1.85]	0.23	23.13
6	32469562	chr6:32469562:C:T	HLA-DRB9	Intronic	rs374532386	C>T	5	4.00x10-31	2.53 [2.18-2.94]	0.281	20.95
6	32469689	chr6:32469689:A:T	HLA-DRB9	Intronic	rs9269027	A>T	10	2.04x10-10	0.70 [0.63-0.78]	0.044	48.09
6	32469837	chr6:32469837:G:T	HLA-DRB9	Intronic	rs9405038	G>T	10	2.85x10-83	1.77 [1.68-1.87]	0.259	19.98
6	32470181	chr6:32470181:T:C	HLA-DRB9	Intronic	rs9269037	T>C	10	2.67x10-10	0.70 [0.63-0.78]	0.039	49.12
6	32470382	chr6:32470382:C:T	HLA-DRB9	Intronic	rs9368725	C>T	10	2.85x10-83	1.77 [1.68-1.87]	0.259	19.98
6	32470466	chr6:32470466:G:A	HLA-DRB9	Intronic	rs9269041	G>A	10	2.08x10-09	0.66 [0.58-0.75]	0	70.49
6	32470521	chr6:32470521:A:G	HLA-DRB9	Intronic	rs9380306	A>G	10	2.52x10-83	1.77 [1.68-1.87]	0.26	19.92
6	32470610	chr6:32470610:C:T	HLA-DRB9	Intronic	rs9269042	C>T	10	5.79x10-16	0.61 [0.54-0.69]	0.004	63.11
6	32470765	chr6:32470765:T:C	HLA-DRB9	Intronic	rs9368726	T>C	10	2.85x10-83	1.77 [1.68-1.87]	0.259	19.98
6	32470827	chr6:32470827:A:T	HLA-DRB9	Intronic	rs6924760	A>T	10	1.01x10-09	0.60 [0.51-0.70]	0.587	0
6	32470871	chr6:32470871:C:T	HLA-DRB9	Intronic	rs9405108	C>T	10	2.85x10-83	1.77 [1.68-1.87]	0.259	19.98
6	32471006	chr6:32471006:C:G	HLA-DRB9	Intronic	rs9269047	C>G	9	2.06x10-13	0.66 [0.60-0.74]	0.647	0
6	32471090	chr6:32471090:G:T	HLA-DRB9	Intronic	rs9391877	G>T	10	2.85x10-83	1.77 [1.68-1.87]	0.259	19.98
6	32471252	chr6:32471252:T:C	HLA-DRB9	Intronic	rs9405109	T>C	10	2.39x10-83	1.77 [1.68-1.87]	0.253	20.6
6	32471300	chr6:32471300:T:C	HLA-DRB9	Intronic	rs7452864	T>C	10	1.21x10-10	0.70 [0.63-0.78]	0.046	47.66
6	32471331	chr6:32471331:A:C	HLA-DRB9	Intronic	rs9405110	A>C	10	5.82x10-83	1.77 [1.68-1.87]	0.256	20.36
6	32471919	chr6:32471919:T:G	HLA-DRB9	Intronic	rs9286789	T>G	10	1.01x10-09	0.60 [0.51-0.70]	0.587	0

6	32472051	chr6:32472051:G:A	HLA-DRB9	Intronic	rs9286790	G>A	10	2.85x10-83	1.77 [1.68-1.87]	0.259	19.98
6	32472187	chr6:32472187:G:T	HLA-DRB9	Intronic	rs5026743	G>T	10	5.79x10-16	0.61 [0.54-0.69]	0.004	63.11
6	32473269	chr6:32473269:G:A	HLA-DRB9	Intronic	rs9461755	G>A	10	9.40x10-10	0.60 [0.51-0.70]	0.584	0
6	32473323	chr6:32473323:A:C	HLA-DRB9	Intronic	rs9269081	A>C	10	1.21x10-10	0.70 [0.63-0.78]	0.046	47.66
6	32473352	chr6:32473352:G:A	HLA-DRB9	Intronic	rs9461756	G>A	10	1.03x10-09	0.60 [0.51-0.70]	0.59	0
6	32528393	chr6:32528393:G:A	HLA-DRB5	Intronic	rs998861334	G>A	9	5.09x10-09	0.62 [0.53-0.73]	0.839	0
6	32528403	chr6:32528403:G:C	HLA-DRB5	Intronic	rs67456882	G>C	10	5.61x10-15	0.70 [0.64-0.76]	0.211	25.26
6	32529172	chr6:32529172:A:G	HLA-DRB5	Intronic	rs115003145	A>G	10	7.63x10-16	0.69 [0.63-0.75]	0.157	31.47
6	32529242	chr6:32529242:T:G	HLA-DRB5	Intronic	rs112875478	T>G	10	1.65x10-15	0.69 [0.63-0.76]	0.245	21.49
6	32553707	chr6:32553707:G:A	HLA-DRB6	Intronic	rs71536528	G>A	10	5.69x10-16	0.69 [0.63-0.75]	0.264	19.46
6	32554331	chr6:32554331:G:A	HLA-DRB6	Intronic	rs554350950	G>A	10	5.45x10-81	1.77 [1.67-1.87]	0.183	28.38
6	32558619	chr6:32558619:A:G	HLA-DRB6	Intronic	rs28534491	A>G	10	1.81x10-13	0.70 [0.64-0.77]	0.427	1.19
6	32558713	chr6:32558713:T:G	HLA-DRB6	Intronic	rs68176300	T>G	10	1.03x10-15	0.69 [0.63-0.75]	0.194	27.2
6	32558749	chr6:32558749:A:G	HLA-DRB6	Intronic	rs35083819	A>G	10	1.03x10-15	0.69 [0.63-0.75]	0.194	27.2
6	32582954	chr6:32582954:G:T	HLA-DRB1	Intronic	rs9269870	G>T	10	1.08x10-15	0.69 [0.63-0.75]	0.213	25
6	32628271	chr6:32628271:G:A	HLA-DQA1	Intronic	rs9271906	G>A	10	1.68x10-33	1.42 [1.34-1.50]	0.859	0
6	32628346	chr6:32628346:C:T	HLA-DQA1	Intronic	rs9271907	C>T	10	1.68x10-33	1.42 [1.34-1.50]	0.859	0
6	32628354	chr6:32628354:C:T	HLA-DQA1	Intronic	rs9271908	C>T	10	1.68x10-33	1.42 [1.34-1.50]	0.859	0
6	32628400	chr6:32628400:A:T	HLA-DQA1	Intronic	rs9271909	A>T	10	1.74x10-33	1.42 [1.34-1.50]	0.858	0
6	32628406	chr6:32628406:T:C	HLA-DQA1	Intronic	rs9271910	T>C	10	1.74x10-33	1.42 [1.34-1.50]	0.858	0
6	32628426	chr6:32628426:C:T	HLA-DQA1	Intronic	rs2133036	C>T	10	2.64x10-85	2.02 [1.89-2.16]	0.939	0
6	32628430	chr6:32628430:C:T	HLA-DQA1	Intronic	rs9271912	C>T	10	1.68x10-33	1.42 [1.34-1.50]	0.859	0
6	32628445	chr6:32628445:T:A	HLA-DQA1	Intronic	rs9271913	T>A	10	1.68x10-33	1.42 [1.34-1.50]	0.859	0
6	32628461	chr6:32628461:T:C	HLA-DQA1	Intronic	rs9271914	T>C	10	1.68x10-33	1.42 [1.34-1.50]	0.859	0
6	32628470	chr6:32628470:G:T	HLA-DQA1	Intronic	rs13207922	G>T	8	3.13x10-14	0.46 [0.38-0.56]	0.855	0
6	32628517	chr6:32628517:T:C	HLA-DQA1	Intronic	rs9271915	T>C	10	1.69x10-33	1.42 [1.34-1.50]	0.86	0
6	32628532	chr6:32628532:C:T	HLA-DQA1	Intronic	rs2036202	C>T	10	8.05x10-18	0.69 [0.64-0.75]	0.215	24.87
6	32628559	chr6:32628559:T:G	HLA-DQA1	Intronic	rs9271917	T>G	10	1.97x10-32	1.41 [1.34-1.49]	0.788	0
6	32628575	chr6:32628575:C:T	HLA-DQA1	Intronic	rs9271918	C>T	10	1.68x10-33	1.42 [1.34-1.50]	0.859	0
6	32628593	chr6:32628593:C:T	HLA-DQA1	Intronic	rs73728888	C>T	9	1.68x10-08	0.61 [0.52-0.72]	0.779	0
6	32628646	chr6:32628646:A:G	HLA-DQA1	Intronic	rs9271919	A>G	10	1.61x10-33	1.42 [1.34-1.50]	0.86	0
6	32628652	chr6:32628652:C:T	HLA-DQA1	Intronic	rs202092176	C>T	10	9.33x10-12	0.70 [0.63-0.77]	0.927	0
6	32628653	chr6:32628653:T:G	HLA-DQA1	Intronic	rs9271920	T>G	10	1.68x10-33	1.42 [1.34-1.50]	0.859	0
6	32628654	chr6:32628654:C:G	HLA-DQA1	Intronic	rs9271921	C>G	10	1.68x10-33	1.42 [1.34-1.50]	0.859	0
6	32628667	chr6:32628667:G:A	HLA-DQA1	Intronic	rs9271922	G>A	10	1.68x10-33	1.42 [1.34-1.50]	0.859	0
6	32628870	chr6:32628870:T:C	HLA-DQA1	Intronic	rs9271927	T>C	10	1.68x10-33	1.42 [1.34-1.50]	0.859	0
6	32628871	chr6:32628871:G:A	HLA-DQA1	Intronic	rs9271928	G>A	10	1.68x10-33	1.42 [1.34-1.50]	0.859	0
6	32628873	chr6:32628873:A:C	HLA-DQA1	Intronic	rs9271929	A>C	10	8.41x10-35	1.43 [1.36-1.51]	0.802	0
6	32628900	chr6:32628900:A:G	HLA-DQA1	Intronic	rs9271930	A>G	10	1.68x10-33	1.42 [1.34-1.50]	0.859	0
6	32628937	chr6:32628937:T:C	HLA-DQA1	Intronic	rs9271932	T>C	10	1.68x10-33	1.42 [1.34-1.50]	0.859	0
6	32628940	chr6:32628940:T:A	HLA-DQA1	Intronic	rs9271933	T>A	10	1.68x10-33	1.42 [1.34-1.50]	0.859	0
6	32628996	chr6:32628996:A:T	HLA-DQA1	Intronic	rs9271934	A>T	10	1.44x10-33	1.42 [1.34-1.50]	0.856	0
6	32629054	chr6:32629054:T:C	HLA-DQA1	Intronic	rs9271935	T>C	10	1.68x10-33	1.42 [1.34-1.50]	0.859	0
6	32629079	chr6:32629079:C:G	HLA-DQA1	Intronic	rs73728889	C>G	10	8.48x10-12	0.70 [0.63-0.77]	0.925	0
6	32629093	chr6:32629093:A:G	HLA-DQA1	Intronic	rs9271937	A>G	10	1.52x10-33	1.42 [1.34-1.50]	0.858	0
6	32629101	chr6:32629101:A:G	HLA-DQA1	Intronic	rs114786106	A>G	10	7.34x10-12	0.70 [0.63-0.77]	0.915	0

6	32629131	chr6:32629131:G:C	HLA-DQA1	Intronic	rs6929020	G>C	10	5.10x10-09	0.84 [0.79-0.89]	0.162	30.82
6	32629144	chr6:32629144:T:C	HLA-DQA1	Intronic	rs9271939	T>C	10	1.68x10-33	1.42 [1.34-1.50]	0.859	0
6	32629160	chr6:32629160:G:T	HLA-DQA1	Intronic	rs3104386	G>T	10	6.14x10-15	0.70 [0.64-0.76]	0.158	31.36
6	32629169	chr6:32629169:G:A	HLA-DQA1	Intronic	rs6928873	G>A	10	4.87x10-09	0.84 [0.79-0.89]	0.165	30.48
6	32629228	chr6:32629228:A:C	HLA-DQA1	Intronic	rs6929057	A>C	10	3.78x10-33	0.65 [0.61-0.70]	0.137	33.88
6	32629245	chr6:32629245:G:A	HLA-DQA1	Intronic	rs3129769	G>A	10	1.19x10-86	2.03 [1.90-2.16]	0.926	0
6	32629257	chr6:32629257:G:A	HLA-DQA1	Intronic	rs9271945	G>A	10	1.97x10-33	1.42 [1.34-1.50]	0.855	0
6	32629287	chr6:32629287:G:A	HLA-DQA1	Intronic	rs3129770	G>A	10	9.04x10-16	0.79 [0.74-0.83]	0.163	30.75
6	32629328	chr6:32629328:C:A	HLA-DQA1	Intronic	rs9271948	C>A	10	1.68x10-33	1.42 [1.34-1.50]	0.859	0
6	32629439	chr6:32629439:T:C	HLA-DQA1	Intronic	rs9271954	T>C	10	1.97x10-33	1.42 [1.34-1.50]	0.858	0
6	32629504	chr6:32629504:G:A	HLA-DQA1	Intronic	rs9271955	G>A	10	1.68x10-33	1.42 [1.34-1.50]	0.859	0
6	32629536	chr6:32629536:T:C	HLA-DQA1	Intronic	rs9271956	T>C	10	7.86x10-15	0.70 [0.64-0.76]	0.166	30.43
6	32629558	chr6:32629558:T:C	HLA-DQA1	Intronic	rs9271957	T>C	10	2.04x10-33	1.42 [1.34-1.50]	0.864	0
6	32629564	chr6:32629564:G:A	HLA-DQA1	Intronic	rs114798579	G>A	8	3.13x10-14	0.46 [0.38-0.56]	0.855	0
6	32630137	chr6:32630137:G:A	HLA-DQA1	Intronic	rs11754858	G>A	9	1.68x10-08	0.61 [0.52-0.72]	0.779	0
6	32630207	chr6:32630207:G:A	HLA-DQA1	Intronic	rs11754883	G>A	9	1.67x10-08	0.61 [0.52-0.72]	0.778	0
6	32630261	chr6:32630261:C:T	HLA-DQA1	Intronic	rs9272007	C>T	10	1.85x10-33	1.42 [1.34-1.50]	0.862	0
6	32630265	chr6:32630265:G:A	HLA-DQA1	Intronic	rs9272008	G>A	10	1.85x10-33	1.42 [1.34-1.50]	0.862	0
6	32630286	chr6:32630286:C:T	HLA-DQA1	Intronic	rs9272009	C>T	10	4.45x10-33	1.42 [1.34-1.49]	0.852	0
6	32630290	chr6:32630290:A:C	HLA-DQA1	Intronic	rs2395519	A>C	10	8.79x10-87	2.03 [1.90-2.16]	0.926	0
6	32630327	chr6:32630327:T:C	HLA-DQA1	Intronic	rs115266533	T>C	10	1.68x10-33	1.42 [1.34-1.50]	0.859	0
6	32630379	chr6:32630379:G:A	HLA-DQA1	Intronic	rs9272012	G>A	10	1.68x10-33	1.42 [1.34-1.50]	0.859	0
6	32630447	chr6:32630447:C:T	HLA-DQA1	Intronic	rs9272013	C>T	10	1.68x10-33	1.42 [1.34-1.50]	0.859	0
6	32630514	chr6:32630514:A:G	HLA-DQA1	Intronic	rs11970000	A>G	10	3.53x10-36	0.63 [0.59-0.68]	0.243	21.7
6	32630522	chr6:32630522:G:A	HLA-DQA1	Intronic	rs9272015	G>A	10	1.68x10-33	1.42 [1.34-1.50]	0.859	0
6	32630569	chr6:32630569:C:T	HLA-DQA1	Intronic	rs9272016	C>T	10	1.68x10-33	1.42 [1.34-1.50]	0.859	0
6	32630804	chr6:32630804:C:A	HLA-DQA1	Intronic	rs9272025	C>A	10	8.76x10-83	1.78 [1.69-1.88]	0.177	29.05
6	32630840	chr6:32630840:G:C	HLA-DQA1	Intronic	rs192408808	G>C	9	1.68x10-08	0.61 [0.52-0.72]	0.779	0
6	32630841	chr6:32630841:A:G	HLA-DQA1	Intronic	rs9272026	A>G	10	1.68x10-33	1.42 [1.34-1.50]	0.859	0
6	32630845	chr6:32630845:C:G	HLA-DQA1	Intronic	rs9272027	C>G	10	1.68x10-33	1.42 [1.34-1.50]	0.859	0
6	32630929	chr6:32630929:C:T	HLA-DQA1	Intronic	rs9272028	C>T	10	1.68x10-33	1.42 [1.34-1.50]	0.859	0
6	32630930	chr6:32630930:C:G	HLA-DQA1	Intronic	rs9272029	C>G	10	1.68x10-33	1.42 [1.34-1.50]	0.859	0
6	32630987	chr6:32630987:C:G	HLA-DQA1	Intronic	rs114354029	C>G	10	1.68x10-33	1.42 [1.34-1.50]	0.859	0
6	32631007	chr6:32631007:G:C	HLA-DQA1	Intronic	rs3104383	G>C	10	6.05x10-15	0.70 [0.64-0.76]	0.158	31.3
6	32631077	chr6:32631077:G:A	HLA-DQA1	Intronic	rs3104381	G>A	10	1.03x10-86	2.03 [1.90-2.16]	0.924	0
6	32631140	chr6:32631140:A:C	HLA-DQA1	Intronic	rs9272042	A>C	10	1.25x10-38	1.46 [1.38-1.54]	0.776	0
6	32631169	chr6:32631169:T:G	HLA-DQA1	Intronic	rs9272043	T>G	10	1.25x10-38	1.46 [1.38-1.54]	0.776	0
6	32631194	chr6:32631194:C:A	HLA-DQA1	Intronic	rs9272044	C>A	10	1.68x10-33	1.42 [1.34-1.50]	0.859	0
6	32631229	chr6:32631229:C:T	HLA-DQA1	Intronic	rs9272045	C>T	10	1.57x10-38	1.46 [1.38-1.54]	0.74	0
6	32631265	chr6:32631265:G:T	HLA-DQA1	Intronic	rs3104380	G>T	10	6.05x10-15	0.70 [0.64-0.76]	0.158	31.3
6	32631268	chr6:32631268:T:G	HLA-DQA1	Intronic	rs9272049	T>G	10	1.68x10-33	1.42 [1.34-1.50]	0.859	0
6	32631294	chr6:32631294:G:A	HLA-DQA1	Intronic	rs9272050	G>A	10	2.47x10-20	1.32 [1.25-1.40]	0.987	0
6	32631337	chr6:32631337:G:T	HLA-DQA1	Intronic	rs3104379	G>T	10	6.05x10-15	0.70 [0.64-0.76]	0.158	31.3
6	32631354	chr6:32631354:G:A	HLA-DQA1	Intronic	rs76434237	G>A	9	1.68x10-08	0.61 [0.52-0.72]	0.779	0
6	32631386	chr6:32631386:C:T	HLA-DQA1	Intronic	rs3104378	C>T	10	8.88x10-87	2.03 [1.90-2.16]	0.928	0
6	32631387	chr6:32631387:C:A	HLA-DQA1	Intronic	rs9272054	C>A	10	1.68x10-33	1.42 [1.34-1.50]	0.859	0

6	32631399	chr6:32631399:T:A	HLA-DQA1	Intronic	rs9272055	T>A	10	1.68x10-33	1.42 [1.34-1.50]	0.859	0
6	32631403	chr6:32631403:G:A	HLA-DQA1	Intronic	rs9272056	G>A	10	1.25x10-38	1.46 [1.38-1.54]	0.776	0
6	32631413	chr6:32631413:T:A	HLA-DQA1	Intronic	rs9272057	T>A	10	1.25x10-38	1.46 [1.38-1.54]	0.776	0
6	32631435	chr6:32631435:T:C	HLA-DQA1	Intronic	rs73728894	T>C	9	1.68x10-08	0.61 [0.52-0.72]	0.779	0
6	32631446	chr6:32631446:T:C	HLA-DQA1	Intronic	rs9272059	T>C	10	1.68x10-33	1.42 [1.34-1.50]	0.859	0
6	32631473	chr6:32631473:G:A	HLA-DQA1	Intronic	rs9272061	G>A	10	1.68x10-33	1.42 [1.34-1.50]	0.859	0
6	32631488	chr6:32631488:G:A	HLA-DQA1	Intronic	rs9272062	G>A	10	1.25x10-38	1.46 [1.38-1.54]	0.776	0
6	32631495	chr6:32631495:A:G	HLA-DQA1	Intronic	rs35779452	A>G	10	3.53x10-36	0.63 [0.59-0.68]	0.243	21.7
6	32631560	chr6:32631560:C:T	HLA-DQA1	Intronic	rs34061722	C>T	10	3.53x10-36	0.63 [0.59-0.68]	0.243	21.7
6	32631561	chr6:32631561:C:T	HLA-DQA1	Intronic	rs9272064	C>T	10	1.68x10-33	1.42 [1.34-1.50]	0.859	0
6	32631624	chr6:32631624:C:A	HLA-DQA1	Intronic	rs9272067	C>A	10	1.68x10-33	1.42 [1.34-1.50]	0.859	0
6	32631658	chr6:32631658:G:C	HLA-DQA1	Intronic	rs35360625	G>C	10	3.53x10-36	0.63 [0.59-0.68]	0.243	21.7
6	32631708	chr6:32631708:T:G	HLA-DQA1	Intronic	rs9272070	T>G	10	1.25x10-38	1.46 [1.38-1.54]	0.776	0
6	32631710	chr6:32631710:G:C	HLA-DQA1	Intronic	rs9272071	G>C	10	1.47x10-21	1.32 [1.25-1.40]	0.422	1.8
6	32632119	chr6:32632119:C:T	HLA-DQA1	Intronic	rs9272103	C>T	10	1.68x10-33	1.42 [1.34-1.50]	0.859	0
6	32632127	chr6:32632127:G:A	HLA-DQA1	Intronic	rs34680078	G>A	8	2.74x10-14	0.46 [0.38-0.56]	0.852	0
6	32632141	chr6:32632141:T:C	HLA-DQA1	Intronic	rs9272104	T>C	10	1.25x10-38	1.46 [1.38-1.54]	0.776	0
6	32632222	chr6:32632222:G:A	HLA-DQA1	Intronic	rs9272105	G>A	10	1.25x10-38	1.46 [1.38-1.54]	0.776	0
6	32632226	chr6:32632226:T:C	HLA-DQA1	Intronic	rs3104376	T>C	10	1.99x10-18	0.71 [0.66-0.76]	0.311	14.4
6	32632269	chr6:32632269:G:A	HLA-DQA1	Intronic	rs35585151	G>A	10	7.21x10-40	0.63 [0.59-0.67]	0.116	36.57
6	32632280	chr6:32632280:A:G	HLA-DQA1	Intronic	rs35242582	A>G	10	3.41x10-36	0.63 [0.59-0.68]	0.243	21.74
6	32632289	chr6:32632289:T:G	HLA-DQA1	Intronic	rs34180045	T>G	10	3.41x10-36	0.63 [0.59-0.68]	0.243	21.74
6	32632297	chr6:32632297:A:G	HLA-DQA1	Intronic	rs34107231	A>G	10	3.41x10-36	0.63 [0.59-0.68]	0.243	21.74
6	32632302	chr6:32632302:T:C	HLA-DQA1	Intronic	rs35635298	T>C	10	3.41x10-36	0.63 [0.59-0.68]	0.243	21.74
6	32632307	chr6:32632307:A:T	HLA-DQA1	Intronic	rs35744886	A>T	10	3.41x10-36	0.63 [0.59-0.68]	0.243	21.74
6	32632322	chr6:32632322:T:C	HLA-DQA1	Intronic	rs35828545	T>C	10	3.41x10-36	0.63 [0.59-0.68]	0.243	21.74
6	32632324	chr6:32632324:G:C	HLA-DQA1	Intronic	rs3104375	G>C	10	8.88x10-87	2.03 [1.90-2.16]	0.928	0
6	32632358	chr6:32632358:T:C	HLA-DQA1	Intronic	rs35426875	T>C	10	3.41x10-36	0.63 [0.59-0.68]	0.243	21.74
6	32632365	chr6:32632365:C:T	HLA-DQA1	Intronic	rs34700194	C>T	9	1.68x10-08	0.61 [0.52-0.72]	0.779	0
6	32632376	chr6:32632376:C:T	HLA-DQA1	Intronic	rs35656734	C>T	10	7.21x10-40	0.63 [0.59-0.67]	0.116	36.57
6	32660321	chr6:32660321:G:C	HLA-DQB1	Intronic	rs28703037	G>C	9	2.72x10-08	0.61 [0.52-0.73]	0.833	0
6	32660350	chr6:32660350:T:C	HLA-DQB1	Intronic	rs9273482	T>C	10	5.30x10-20	0.57 [0.51-0.64]	0.007	60.41
6	32660829	chr6:32660829:C:A	HLA-DQB1	Intronic	rs201049473	C>A	9	3.36x10-08	0.62 [0.52-0.73]	0.843	0
6	32660883	chr6:32660883:T:C	HLA-DQB1	Intronic	rs28688207	T>C	8	4.23x10-10	0.53 [0.44-0.64]	0.289	17.87
6	32660935	chr6:32660935:C:T	HLA-DQB1	Intronic	rs9273531	C>T	9	7.37x10-66	2.07 [1.92-2.24]	0.951	0
6	32661805	chr6:32661805:C:T	HLA-DQB1	Intronic	rs9273841	C>T	10	1.20x10-26	0.67 [0.62-0.71]	0.572	0
6	32661862	chr6:32661862:T:C	HLA-DQB1	Intronic	rs9273866	T>C	10	2.30x10-55	1.73 [1.62-1.84]	0.329	12.37
6	32663139	chr6:32663139:G:T	HLA-DQB1	Intronic	rs9274212	G>T	10	1.21x10-26	0.67 [0.62-0.71]	0.573	0
6	32663153	chr6:32663153:G:C	HLA-DQB1	Intronic	rs9274214	G>C	10	1.20x10-26	0.67 [0.62-0.71]	0.573	0
6	32663190	chr6:32663190:C:A	HLA-DQB1	Intronic	rs2854267	C>A	10	9.37x10-27	0.66 [0.62-0.71]	0.568	0
6	32663214	chr6:32663214:G:A	HLA-DQB1	Intronic	rs35986240	G>A	7	2.21x10-11	0.54 [0.46-0.65]	0.096	44.32
6	32663252	chr6:32663252:T:C	HLA-DQB1	Intronic	rs58770498	T>C	9	2.72x10-08	0.61 [0.52-0.73]	0.833	0
6	32663262	chr6:32663262:A:G	HLA-DQB1	Intronic	rs9274218	A>G	10	1.07x10-26	0.66 [0.62-0.71]	0.569	0
6	32663281	chr6:32663281:A:G	HLA-DQB1	Intronic	rs2856703	A>G	10	1.44x10-55	1.73 [1.62-1.84]	0.354	9.6
6	32663555	chr6:32663555:A:G	HLA-DQB1	Intronic	rs9274250	A>G	10	1.21x10-26	0.67 [0.62-0.71]	0.573	0
6	32663651	chr6:32663651:T:C	HLA-DQB1	Intronic	rs9274256	T>C	10	2.09x10-14	1.59 [1.42-1.78]	0.002	65.15

6	32663653	chr6:32663653:G:A	HLA-DQB1	Intronic	rs9274257	G>A	10	1.21x10-26	0.67 [0.62-0.71]	0.573	0
6	32663671	chr6:32663671:A:G	HLA-DQB1	Intronic	rs9274258	A>G	10	4.27x10-44	1.50 [1.42-1.58]	0.052	46.4
6	32663675	chr6:32663675:C:T	HLA-DQB1	Intronic	rs200501154	C>T	9	2.41x10-08	0.61 [0.52-0.73]	0.833	0
6	32663681	chr6:32663681:T:C	HLA-DQB1	Intronic	rs2300825	T>C	10	5.27x10-67	1.88 [1.76-2.01]	0.49	0
6	32663696	chr6:32663696:C:T	HLA-DQB1	Intronic	rs9274260	C>T	10	2.46x10-55	1.73 [1.62-1.84]	0.328	12.44
6	32663777	chr6:32663777:G:A	HLA-DQB1	Intronic	rs56260471	G>A	9	2.64x10-08	0.61 [0.52-0.73]	0.834	0
6	32663793	chr6:32663793:G:A	HLA-DQB1	Intronic	rs9274265	G>A	10	3.22x10-55	1.73 [1.62-1.84]	0.326	12.76
6	32665621	chr6:32665621:T:C	HLA-DQB1	Intronic	rs28746824	T>C	10	7.38x10-56	1.73 [1.62-1.85]	0.285	17.21
6	32666182	chr6:32666182:T:C	HLA-DQB1	Intronic	rs28746835	T>C	10	3.18x10-55	1.73 [1.62-1.84]	0.325	12.86
6	32666194	chr6:32666194:A:G	HLA-DQB1	Intronic	rs3830060	A>G	10	5.27x10-67	1.88 [1.76-2.01]	0.49	0
6	32666230	chr6:32666230:A:G	HLA-DQB1	Intronic	rs3830059	A>G	10	3.18x10-55	1.73 [1.62-1.84]	0.325	12.86
6	32666233	chr6:32666233:G:A	HLA-DQB1	Intronic	rs3830058	G>A	10	1.21x10-26	0.67 [0.62-0.71]	0.573	0
6	32666255	chr6:32666255:A:G	HLA-DQB1	Intronic	rs9274501	A>G	10	6.29x10-20	0.57 [0.51-0.64]	0.006	60.9
6	32666280	chr6:32666280:C:T	HLA-DQB1	Intronic	rs28746838	C>T	10	3.18x10-55	1.73 [1.62-1.84]	0.325	12.86
6	32666313	chr6:32666313:T:C	HLA-DQB1	Intronic	rs28746839	T>C	10	3.18x10-55	1.73 [1.62-1.84]	0.325	12.86
6	32666317	chr6:32666317:T:C	HLA-DQB1	Intronic	rs28746841	T>C	10	3.18x10-55	1.73 [1.62-1.84]	0.325	12.86
6	32666327	chr6:32666327:C:T	HLA-DQB1	Intronic	rs9274503	C>T	10	1.26x10-09	0.83 [0.79-0.88]	0.293	16.3
6	32666362	chr6:32666362:C:T	HLA-DQB1	Intronic	rs28746844	C>T	10	3.18x10-55	1.73 [1.62-1.84]	0.325	12.86
6	32666373	chr6:32666373:A:C	HLA-DQB1	Intronic	rs28746845	A>C	10	3.18x10-55	1.73 [1.62-1.84]	0.325	12.86
6	32666375	chr6:32666375:G:A	HLA-DQB1	Intronic	rs9274507	G>A	10	1.21x10-26	0.67 [0.62-0.71]	0.573	0
6	32666407	chr6:32666407:A:G	HLA-DQB1	Intronic	rs9274510	A>G	10	8.99x10-22	0.57 [0.51-0.64]	0.014	56.5
6	32666484	chr6:32666484:C:T	HLA-DQB1	Intronic	rs28746846	C>T	10	3.79x10-55	1.73 [1.62-1.84]	0.308	14.68
6	32666485	chr6:32666485:T:G	HLA-DQB1	Intronic	rs28746847	T>G	10	3.79x10-55	1.73 [1.62-1.84]	0.308	14.68
6	32744889	chr6:32744889:C:T	HLA-DQA2	Intronic	rs9276434	C>T	8	8.45x10-11	0.71 [0.64-0.78]	0.506	0
6	32815018	chr6:32815018:C:A	HLA-DOB	Intronic	rs41316241	C>A	10	3.53x10-09	1.47 [1.30-1.67]	0.308	14.64
6	32829390	chr6:32829390:C:T	TAP2	Intronic	rs41316548	C>T	10	4.34x10-09	1.47 [1.29-1.66]	0.266	19.26
6	32834577	chr6:32834577:C:A	TAP2	Intronic	rs41317090	C>A	8	3.94x10-10	1.54 [1.35-1.76]	0.717	0
6	32846350	chr6:32846350:G:A	TAP1	Intronic	rs1053752	G>A	10	3.52x10-09	1.19 [1.12-1.25]	0.055	45.86
6	32846670	chr6:32846670:T:C	TAP1	Intronic	rs6457684	T>C	10	4.21x10-09	1.18 [1.12-1.25]	0.054	46.01
6	32851740	chr6:32851740:T:C	TAP1	Intronic	rs2284190	T>C	10	4.82x10-15	1.37 [1.27-1.48]	0.051	46.67
6	160587614	chr6:160587614:G:A	LPA	Intronic	rs41272086	G>A	10	4.04x10-08	0.74 [0.67-0.82]	0.292	16.37
6	160671406	chr6:160671406:C:T	-	Intronic	rs11751347	C>T	10	2.64x10-08	0.73 [0.66-0.82]	0.226	23.57
6	160702381	chr6:160702381:G:T	PLG	Intronic	rs2314851	G>T	10	4.41x10-10	0.84 [0.79-0.88]	0.215	24.83
6	160716958	chr6:160716958:G:A	PLG	Intronic	rs783147	G>A	10	6.53x10-09	1.18 [1.12-1.25]	0.06	44.96
6	160722158	chr6:160722158:T:C	PLG	Intronic	rs4252114	T>C	10	1.38x10-13	1.25 [1.18-1.32]	0.063	44.42
6	160724144	chr6:160724144:A:T	PLG	Intronic	rs1321197	A>T	10	2.62x10-13	1.24 [1.18-1.32]	0.059	45.11
6	160725672	chr6:160725672:A:G	PLG	Intronic	rs1897108	A>G	10	2.57x10-13	1.24 [1.18-1.32]	0.059	45.16
8	27726817	chr8:27726817:A:G	-	Intergenic	rs4323431	A>G	10	2.19x10-08	1.17 [1.11-1.24]	0.539	0
8	27754871	chr8:27754871:T:C	CCDC25	Intronic	rs4146307	T>C	10	3.78x10-08	1.17 [1.11-1.24]	0.461	0
8	27755309	chr8:27755309:C:T	CCDC25	Intronic	rs10866862	C>T	10	3.76x10-08	1.17 [1.11-1.24]	0.46	0
8	27755771	chr8:27755771:G:A	CCDC25	Intronic	rs11782593	G>A	10	3.75x10-08	1.17 [1.11-1.24]	0.459	0
8	27755870	chr8:27755870:G:T	CCDC25	Intronic	rs11782624	G>T	10	1.28x10-08	1.18 [1.12-1.25]	0.424	1.54
15	88906856	chr15:88906856:C:T	MFGE8	Intronic	rs8029053	C>T	10	4.96x10-08	1.19 [1.12-1.26]	0.533	0
17	28367840	chr17:28367840:G:A	VTN	xonic (missens	rs704	G>A	9	2.75x10-09	0.84 [0.79-0.89]	0.248	21.97
17	28366210	chr17:28366210:G:C	VTN	Intergenic	rs2227736	G>C	9	1.01x10-08	1.19 [1.12-1.26]	0.695	0
17	28366237	chr17:28366237:C:T	VTN	Intergenic	rs2227735	C>T	9	1.05x10-08	1.19 [1.12-1.26]	0.69	0

17	28398249	chr17:28398249:G:A	SARM1	Intergenic	rs2239908	G>A	8	1.38x10-08	1.19 [1.12-1.26]	0.718	0
17	28398728	chr17:28398728:T:C	SARM1	Intergenic	rs2239907	T>C	8	4.49x10-08	1.18 [1.11-1.25]	0.726	0
17	28337780	chr17:28337780:T:C	TNFAIP1	Intronic	rs3093680	T>C	9	6.17x10-09	1.19 [1.12-1.26]	0.799	0
17	28338622	chr17:28338622:A:G	TNFAIP1	Intronic	rs1007398	A>G	9	7.25x10-09	1.19 [1.12-1.26]	0.8	0
17	28388929	chr17:28388929:G:C	SARM1	Intronic	rs6505077	G>C	9	1.49x10-08	1.19 [1.12-1.26]	0.738	0
17	28393545	chr17:28393545:G:A	SARM1	Intronic	rs4795435	G>A	8	7.26x10-09	1.19 [1.12-1.26]	0.773	0
17	28394285	chr17:28394285:A:G	SARM1	Intronic	rs10853128	A>G	8	1.08x10-08	1.19 [1.12-1.26]	0.76	0
17	28394772	chr17:28394772:C:A	SARM1	Intronic	rs1128162	C>A	8	8.21x10-09	1.19 [1.12-1.26]	0.768	0
17	28394876	chr17:28394876:G:A	SARM1	Intronic	rs1128161	G>A	8	9.01x10-09	1.19 [1.12-1.26]	0.756	0
17	28395626	chr17:28395626:G:A	SARM1	Intronic	rs8079943	G>A	8	1.67x10-08	1.18 [1.12-1.26]	0.748	0
17	28395709	chr17:28395709:C:T	SARM1	Intronic	rs8081240	C>T	8	1.57x10-08	1.19 [1.12-1.26]	0.774	0
17	28396594	chr17:28396594:G:T	SARM1	Intronic	rs2239911	G>T	8	1.94x10-08	1.18 [1.12-1.25]	0.705	0

SNP, single nucleotide polymorphism; OR, odds ratio; CI, confidence interval
Only significant p-values are shown. P-values were adjusted when the Cochran's Q was significant.

Supplementary Table 7. Lead variants showing genome-wide significant association with GCA after meta-analysis.

CHR	BP (hg38)	SNP	Nearest gene	Variant type	ID	Change	Meta-analysis				
							N	P	OR	Q	I
6	32652425	chr6:32652425:G:A	*HLA-DQA1*	Intergenic	rs41269974	G>A	10	1.60x10-87	2.03 [1.90-2.17]	0.932	0
6	1.61E+08	chr6:160722158:T:C	*PLG*	Intronic	rs4252114	T>C	10	1.38x10-13	1.25 [1.18-1.32]	0.063	44.42
8	27755870	chr8:27755870:G:T	*CCDC25*	Intronic	rs11782624	G>T	10	1.28x10-08	1.18 [1.12-1.25]	0.424	1.54
15	88906856	chr15:88906856:C:T	*MFGE8*	Intronic	rs8029053	C>T	10	4.96x10-08	1.19 [1.12-1.26]	0.533	0
17	28367840	chr17:28367840:G:A	*VTN*	Exonic (missense)	rs704	G>A	9	2.75x10-09	0.84 [0.79-0.89]	0.248	21.97

ID	Spain		UK		Netherlands		Germany		North America	
	P	OR	P	OR	P	OR	P	OR	P	OR
rs41269974	2.06x10-15	2.01 [1.69-2.38]	8.03x10-46	2.09 [1.89-2.31]	1.13x10-06	2.13 [1.57-2.88]	1.58x10-08	1.97 [1.56-2.50]	3.58x10-11	2.15 [1.72-2.70]
rs4252114	1.40x10-02	1.17 [1.03-1.32]	2.23x10-08	1.30 [1.19-1.42]	1.72x10-02	1.38 [1.06-1.81]	8.26x10-02	1.20 [0.98-1.48]	3.49x10-01	1.11 [0.89-1.37]
rs11782624	3.89x10-05	1.29 [1.14-1.45]	8.81x10-04	1.16 [1.06-1.27]	5.34x10-01	1.08 [0.84-1.40]	1.71x10-01	1.15 [0.94-1.40]	8.31x10-01	1.02 [0.84-1.25]
rs8029053	3.58x10-05	1.31 [1.15-1.50]	3.12x10-03	1.16 [1.05-1.27]	8.59x10-02	1.27 [0.97-1.66]	2.66x10-01	1.13 [0.91-1.40]	1.49x10-01	1.18 [0.94-1.47]
rs704	5.78x10-03	0.85 [0.75-0.95]	4.32x10-05	0.83 [0.76-0.91]	1.39x10-01	0.82 [0.64-1.06]	6.83x10-05	0.67 [0.55-0.81]	8.51x10-01	1.02 [0.84-1.24]

ID	France		Italy		Norway		Ireland		Switzerland	
	P	OR	P	OR	P	OR	P	OR	P	OR
rs41269974	1.88x10-04	2.12 [1.43-3.15]	2.82x10-03	1.71 [1.20-2.43]	5.02x10-02	1.57 [1.00-2.46]	1.04x10-01	1.76 [0.89-3.49]	7.19x10-02	1.66 [0.96-2.88]
rs4252114	8.95x10-01	0.98 [0.70-1.36]	1.45x10-01	1.17 [0.95-1.43]	1.24x10-03	2.04 [1.32-3.15]	1.65x10-01	1.44 [0.86-2.39]	1.92x10-03	2.08 [1.31-3.31]
rs11782624	9.32x10-02	1.31 [0.96-1.81]	7.77x10-03	1.33 [1.08-1.64]	7.55x10-01	0.94 [0.63-1.40]	5.33x10-01	0.85 [0.51-1.42]	3.92x10-01	1.21 [0.78-1.89]
rs8029053	1.85x10-01	1.25 [0.90-1.73]	3.90x10-01	1.10 [0.88-1.38]	8.87x10-01	0.97 [0.64-1.47]	1.95x10-01	0.65 [0.34-1.25]	2.29x10-01	1.34 [0.83-2.14]
rs704	6.15x10-01	0.92 [0.68-1.26]	NA	NA	8.65x10-01	0.97 [0.64-1.45]	4.46x10-01	0.81 [0.47-1.4]	1.28x10-01	0.70 [0.45-1.11]

SNP, single nucleotide polymorphism; OR, odds ratio; CI, confidence interval

Only significant p-values are shown. P-values were adjusted when the Cochran's Q was significant.

Supplementary Table 8. Genomic association results of the HLA region with giant cell arteritis.

CHR	BP (hg18)	SNP	A1	Meta-analysis				
				N	P	OR	Q	I
6	30185208	rs2523990	T	10	5.78x10-10	0.84 [0.79-0.88]	0.72	0.00
6	30198799	rs9261440	C	10	1.18x10-08	1.22 [1.14-1.30]	0.42	1.89
6	30205899	rs9261451	G	10	1.20x10-08	1.22 [1.14-1.30]	0.45	0.00
6	30206302	rs757263	T	10	1.20x10-08	1.22 [1.14-1.30]	0.45	0.00
6	30207927	rs9261453	G	10	2.72x10-08	1.21 [1.13-1.29]	0.52	0.00
6	30207975	rs9261455	G	10	2.76x10-08	1.21 [1.13-1.29]	0.52	0.00
6	30217810	rs9261490	C	10	4.60x10-08	1.20 [1.13-1.28]	0.79	0.00
6	30217840	rs9261491	C	10	4.53x10-08	1.20 [1.13-1.28]	0.79	0.00
6	30218316	rs9261493	A	10	4.60x10-08	1.20 [1.13-1.28]	0.79	0.00
6	30218993	rs9261496	T	10	4.60x10-08	1.20 [1.13-1.28]	0.79	0.00
6	30219075	rs9261497	T	10	4.60x10-08	1.20 [1.13-1.28]	0.79	0.00
6	30219505	rs9261501	T	10	4.33x10-08	1.20 [1.13-1.28]	0.78	0.00
6	30219589	rs9261502	T	10	4.33x10-08	1.20 [1.13-1.28]	0.78	0.00
6	30220606	rs9261507	A	10	4.33x10-08	1.20 [1.13-1.28]	0.78	0.00
6	30220683	rs9261508	C	10	4.33x10-08	1.20 [1.13-1.28]	0.78	0.00
6	30220885	rs9261509	C	10	4.33x10-08	1.20 [1.13-1.28]	0.78	0.00
6	30221323	rs3815086	T	10	4.33x10-08	1.20 [1.13-1.28]	0.78	0.00
6	30221396	rs3815085	T	10	3.12x10-08	1.20 [1.13-1.28]	0.76	0.00
6	30221406	rs3815084	T	10	4.33x10-08	1.20 [1.13-1.28]	0.78	0.00
6	30221450	rs1541324	T	10	4.33x10-08	1.20 [1.13-1.28]	0.78	0.00
6	30221934	rs3815082	G	10	4.33x10-08	1.20 [1.13-1.28]	0.78	0.00
6	30225080	rs9261519	C	10	4.11x10-08	1.20 [1.13-1.28]	0.80	0.00
6	30225892	rs9261523	A	10	4.06x10-08	1.20 [1.13-1.28]	0.79	0.00
6	30226107	rs9261525	C	10	4.06x10-08	1.20 [1.13-1.28]	0.79	0.00
6	30226468	rs1557609	C	10	4.41x10-08	1.20 [1.13-1.28]	0.79	0.00
6	30227371	rs9261527	A	10	4.15x10-08	1.20 [1.13-1.28]	0.83	0.00
6	30227408	rs9261528	G	10	3.99x10-08	1.20 [1.13-1.28]	0.83	0.00
6	30393503	rs34171662	T	10	1.16x10-08	1.42 [1.26-1.60]	0.94	0.00
6	30424959	rs34266569	C	10	8.24x10-09	1.43 [1.27-1.60]	0.92	0.00
6	30523640	rs34982703	T	10	1.33x10-08	1.42 [1.26-1.60]	0.93	0.00
6	30682173	rs12174151	T	10	3.24x10-10	1.27 [1.18-1.37]	0.95	0.00
6	30709211	rs12665339	G	10	4.55x10-10	1.27 [1.18-1.37]	0.95	0.00
6	30778271	rs9405048	T	10	3.32x10-10	1.28 [1.18-1.38]	0.96	0.00
6	31321881	rs3095245	G	10	1.45x10-08	1.18 [1.11-1.25]	0.99	0.00
6	31346171	HLA_C_0304	P	10	8.75x10-11	1.41 [1.27-1.56]	0.54	0.00
6	31347731	SNP_C_31347731_A	P	10	4.56x10-08	1.26 [1.16-1.36]	0.33	11.81
6	31429190	rs2596501	G	10	2.79x10-10	1.20 [1.14-1.27]	0.96	0.00
6	31432578	AA_B_46_31432578	A	10	1.79x10-08	1.27 [1.17-1.37]	0.92	0.00
6	31432578	SNP_B_31432578	G	10	1.79x10-08	1.27 [1.17-1.37]	0.92	0.00
6	31432581	AA_B_45_31432581_M	P	10	1.79x10-08	1.27 [1.17-1.37]	0.92	0.00
6	31432581	AA_B_45_31432581_MG	P	10	1.79x10-08	1.27 [1.17-1.37]	0.92	0.00
6	31432581	AA_B_45_31432581_T	P	10	4.99x10-10	0.80 [0.75-0.86]	0.67	0.00
6	31432581	AA_B_45_31432581_TG	P	10	4.99x10-10	0.80 [0.75-0.86]	0.67	0.00
6	31432581	SNP_B_31432581_A	P	10	1.79x10-08	1.27 [1.17-1.37]	0.92	0.00
6	31432581	SNP_B_31432581_G	P	10	4.99x10-10	0.80 [0.75-0.86]	0.67	0.00
6	31432581	SNP_B_31432581_TA	A	10	4.46x10-10	0.80 [0.74-0.86]	0.67	0.00
6	31432581	SNP_B_31432581_TG	A	10	2.06x10-08	1.27 [1.17-1.37]	0.91	0.00
6	31691910	rs2269475	A	10	1.20x10-08	1.27 [1.17-1.37]	0.62	0.00
6	31695917	rs3763295	G	10	9.27x10-09	1.27 [1.17-1.38]	0.66	0.00
6	31710468	rs2242657	G	10	1.72x10-08	1.26 [1.17-1.37]	0.61	0.00
6	31719819	rs2077102	T	10	2.24x10-08	1.26 [1.16-1.36]	0.62	0.00
6	31735428	rs2242655	C	10	1.97x10-08	1.26 [1.17-1.37]	0.62	0.00

6	32022914	rs1048709	A	10	5.68x10-10	0.77 [0.71-0.84]	0.19	27.62
6	32150300	rs3130286	T	10	3.31x10-11	0.78 [0.73-0.84]	0.86	0.00
6	32222493	rs9296009	T	10	2.93x10-12	1.28 [1.20-1.37]	0.32	13.33
6	32227876	rs3131283	A	10	1.18x10-09	0.74 [0.68-0.82]	0.95	0.00
6	32233980	rs3134603	T	10	2.77x10-08	0.77 [0.71-0.84]	0.88	0.00
6	32246523	rs3130283	A	10	1.70x10-09	0.75 [0.69-0.82]	0.87	0.00
6	32259972	rs1800684	T	10	6.54x10-10	0.74 [0.67-0.81]	0.94	0.00
6	32263559	rs204993	C	10	5.20x10-10	1.22 [1.15-1.30]	0.27	19.08
6	32269374	rs3132940	A	10	1.93x10-09	0.75 [0.68-0.82]	0.90	0.00
6	32280043	rs1044506	A	10	7.87x10-10	0.74 [0.68-0.81]	0.92	0.00
6	32288124	rs3131294	T	10	1.11x10-09	0.74 [0.68-0.82]	0.92	0.00
6	32298006	rs3132946	A	10	2.42x10-09	0.75 [0.68-0.82]	0.89	0.00
6	32298368	rs915894	C	10	3.71x10-10	1.20 [1.14-1.27]	0.42	1.53
6	32306926	rs6457499	C	10	1.41x10-08	0.84 [0.79-0.89]	0.10	39.02
6	32308125	rs3134926	C	10	2.34x10-08	0.84 [0.79-0.89]	0.11	36.86
6	32315371	rs416352	A	10	4.60x10-08	0.85 [0.80-0.90]	0.62	0.00
6	32317839	rs507778	A	10	3.65x10-09	0.84 [0.79-0.89]	0.55	0.00
6	32319063	rs412657	C	10	1.13x10-11	0.82 [0.77-0.87]	0.93	0.00
6	32321128	rs9267955	A	10	3.55x10-13	0.71 [0.65-0.77]	0.82	0.00
6	32321616	rs9267956	G	10	5.77x10-10	1.20 [1.13-1.27]	0.18	28.75
6	32323747	rs1559876	G	10	4.57x10-10	1.20 [1.14-1.27]	0.19	27.88
6	32328375	rs9267992	G	10	6.06x10-13	0.71 [0.65-0.78]	0.77	0.00
6	32341792	rs3115562	A	10	1.38x10-09	0.67 [0.59-0.76]	0.16	31.47
6	32343362	rs2114436	C	10	1.65x10-09	0.67 [0.59-0.76]	0.13	34.97
6	32344032	rs3132933	T	10	1.13x10-09	0.66 [0.58-0.75]	0.10	38.55
6	32362632	rs9268132	G	10	1.20x10-09	1.20 [1.13-1.26]	0.47	0.00
6	32365315	rs4713518	G	10	8.53x10-10	1.20 [1.13-1.27]	0.47	0.00
6	32368537	rs482194	C	10	4.02x10-10	1.20 [1.14-1.27]	0.45	0.00
6	32369749	rs560505	C	10	1.10x10-09	1.20 [1.13-1.27]	0.41	3.38
6	32376479	rs537757	A	10	4.30x10-10	1.20 [1.14-1.27]	0.48	0.00
6	32378478	rs477005	C	10	1.68x10-12	1.23 [1.16-1.30]	0.23	22.64
6	32386244	rs502626	C	10	4.24x10-13	1.24 [1.17-1.31]	0.27	18.45
6	32386613	rs9268199	G	10	1.74x10-16	0.70 [0.65-0.76]	0.25	21.15
6	32390011	rs547261	T	10	5.37x10-13	1.24 [1.17-1.31]	0.28	17.25
6	32394080	rs531094	A	10	3.73x10-13	1.24 [1.17-1.31]	0.27	18.79
6	32394739	rs498422	C	10	1.51x10-11	0.65 [0.58-0.74]	0.16	31.43
6	32396067	rs6920338	C	10	5.26x10-11	0.74 [0.67-0.81]	0.87	0.00
6	32396440	rs539703	C	10	2.55x10-13	1.24 [1.17-1.31]	0.29	17.13
6	32397218	rs546857	T	10	1.94x10-12	1.23 [1.16-1.30]	0.24	21.85
6	32397296	rs547077	C	10	5.58x10-13	1.24 [1.17-1.31]	0.24	21.96
6	32398932	rs485774	C	10	4.56x10-13	1.24 [1.17-1.31]	0.28	17.89
6	32399168	rs496360	G	10	6.67x10-10	0.66 [0.58-0.75]	0.12	36.59
6	32399815	rs523627	T	10	4.56x10-13	1.24 [1.17-1.31]	0.28	17.89
6	32400062	rs525607	A	10	5.58x10-13	1.24 [1.17-1.31]	0.24	21.96
6	32400693	rs552339	T	10	4.56x10-13	1.24 [1.17-1.31]	0.28	17.89
6	32402821	rs504203	C	10	3.93x10-13	1.24 [1.17-1.31]	0.28	18.20
6	32402970	rs505274	A	10	3.82x10-13	1.24 [1.17-1.31]	0.28	17.74
6	32403328	rs508805	A	10	3.82x10-13	1.24 [1.17-1.31]	0.28	17.74
6	32403335	rs524578	T	10	4.20x10-13	1.24 [1.17-1.31]	0.24	22.02
6	32404758	rs9348880	T	10	4.52x10-13	1.24 [1.17-1.31]	0.24	22.10
6	32404820	rs9348881	G	10	4.42x10-13	1.24 [1.17-1.31]	0.23	22.92
6	32405315	rs9368713	C	10	3.69x10-13	1.24 [1.17-1.31]	0.27	18.47
6	32406350	rs9405090	G	10	3.69x10-13	1.24 [1.17-1.31]	0.27	18.47
6	32407851	rs910052	A	10	3.69x10-13	1.24 [1.17-1.31]	0.27	18.47
6	32409267	rs9366793	C	10	4.06x10-13	1.24 [1.17-1.31]	0.23	22.64
6	32409300	rs9348882	T	10	3.69x10-13	1.24 [1.17-1.31]	0.27	18.47

6	32409430	rs9380290	G	10	4.58x10-13	1.24 [1.17-1.31]	0.23	22.67
6	32409492	rs9357140	A	10	3.69x10-13	1.24 [1.17-1.31]	0.27	18.47
6	32410023	rs1474729	G	10	3.31x10-13	1.24 [1.17-1.31]	0.26	19.83
6	32410045	rs1474728	A	10	3.10x10-13	1.24 [1.17-1.31]	0.26	20.12
6	32411046	rs10807100	A	10	3.10x10-13	1.24 [1.17-1.31]	0.26	20.12
6	32411489	rs6929776	A	10	3.47x10-13	1.24 [1.17-1.31]	0.22	24.10
6	32412009	rs6930681	A	10	3.10x10-13	1.24 [1.17-1.31]	0.26	20.12
6	32413103	rs973037	A	10	2.83x10-13	1.24 [1.17-1.31]	0.26	20.43
6	32413348	rs926594	C	10	3.10x10-13	1.24 [1.17-1.31]	0.26	20.12
6	32414068	rs9368716	G	10	8.36x10-17	0.79 [0.74-0.83]	0.22	24.65
6	32415115	rs2022534	G	10	3.10x10-13	1.24 [1.17-1.31]	0.26	20.12
6	32415238	rs2022533	C	10	3.03x10-13	1.24 [1.17-1.31]	0.23	23.62
6	32415360	rs1033500	T	10	3.10x10-13	1.24 [1.17-1.31]	0.26	20.12
6	32416981	rs2143468	A	10	2.65x10-13	1.24 [1.17-1.31]	0.26	20.41
6	32417301	rs2143466	A	10	1.53x10-14	1.25 [1.18-1.32]	0.49	0.00
6	32417330	rs2143465	G	10	3.10x10-13	1.24 [1.17-1.31]	0.26	20.12
6	32418945	rs9268260	T	10	3.03x10-13	1.24 [1.17-1.31]	0.27	19.28
6	32419437	rs3132954	T	10	3.43x10-10	0.83 [0.78-0.88]	0.64	0.00
6	32421114	rs4959094	T	10	3.06x10-13	1.24 [1.17-1.31]	0.26	20.25
6	32421121	rs4959025	C	10	3.06x10-13	1.24 [1.17-1.31]	0.26	20.25
6	32421439	rs4959026	T	10	3.44x10-13	1.24 [1.17-1.31]	0.26	20.07
6	32422654	rs4959096	A	10	2.83x10-13	1.24 [1.17-1.31]	0.25	20.55
6	32423151	rs9268283	C	10	2.83x10-13	1.24 [1.17-1.31]	0.25	20.55
6	32423194	rs9268284	A	10	2.40x10-13	1.24 [1.17-1.31]	0.25	21.26
6	32423535	rs9268285	C	10	3.12x10-13	1.24 [1.17-1.31]	0.26	20.20
6	32425105	rs2076542	T	10	3.08x10-13	1.24 [1.17-1.31]	0.26	20.25
6	32425193	rs2076541	G	10	2.83x10-13	1.24 [1.17-1.31]	0.25	20.55
6	32425254	rs2076540	G	10	1.28x10-12	1.23 [1.17-1.30]	0.22	24.34
6	32425449	rs2076538	G	10	3.03x10-13	1.24 [1.17-1.31]	0.26	20.27
6	32426014	rs761187	C	10	1.29x10-12	1.23 [1.17-1.30]	0.22	24.34
6	32429093	rs1265762	G	10	3.74x10-20	0.76 [0.72-0.80]	0.24	22.47
6	32429575	rs1265761	C	10	4.19x10-10	0.66 [0.58-0.75]	0.11	37.49
6	32430371	rs1265759	G	10	3.74x10-20	0.76 [0.72-0.80]	0.24	22.47
6	32431507	rs1265758	T	10	2.12x10-18	0.77 [0.73-0.81]	0.22	24.05
6	32432522	rs2206618	C	10	3.04x10-16	0.73 [0.68-0.79]	0.27	19.24
6	32432923	rs6907322	A	10	1.44x10-14	0.74 [0.69-0.80]	0.22	23.73
6	32433685	rs3129908	C	10	2.19x10-18	0.77 [0.73-0.81]	0.22	23.91
6	32434023	rs2395150	G	10	2.07x10-18	0.77 [0.73-0.81]	0.22	24.07
6	32434143	rs9268303	A	10	1.44x10-14	0.74 [0.69-0.80]	0.22	23.73
6	32435759	rs6904636	C	10	3.94x10-20	0.76 [0.72-0.80]	0.24	22.33
6	32438131	rs9268326	C	10	3.04x10-13	1.24 [1.17-1.31]	0.26	20.16
6	32439802	rs9268343	G	10	3.04x10-13	1.24 [1.17-1.31]	0.26	20.16
6	32439819	rs9268345	A	10	3.04x10-13	1.24 [1.17-1.31]	0.26	20.16
6	32439976	rs9268347	A	10	2.82x10-16	0.73 [0.68-0.79]	0.27	19.31
6	32441071	rs3129921	A	10	3.74x10-20	0.76 [0.72-0.80]	0.24	22.47
6	32441076	rs3129922	C	10	3.74x10-20	0.76 [0.72-0.80]	0.24	22.47
6	32441173	rs3129923	A	10	2.81x10-20	0.76 [0.72-0.80]	0.25	20.61
6	32441417	rs9268365	T	10	2.85x10-16	0.73 [0.68-0.79]	0.27	19.29
6	32441933	rs9268368	C	10	3.04x10-13	1.24 [1.17-1.31]	0.26	20.16
6	32444052	rs2073046	A	10	3.04x10-13	1.24 [1.17-1.31]	0.26	20.16
6	32444564	rs9268384	G	10	3.04x10-13	1.24 [1.17-1.31]	0.26	20.16
6	32445608	rs2273017	C	10	4.77x10-18	0.77 [0.73-0.82]	0.21	24.91
6	32446364	rs9268394	C	10	2.74x10-13	1.24 [1.17-1.31]	0.25	20.99
6	32446964	rs2073044	A	10	3.28x10-32	1.46 [1.38-1.55]	0.34	11.38
6	32449296	rs9268401	G	10	4.65x10-51	1.58 [1.49-1.67]	0.07	44.04
6	32449451	rs9268403	C	10	4.75x10-51	1.58 [1.49-1.67]	0.07	43.98

6	32449697	rs9268404	T	10	4.65x10-51	1.58 [1.49-1.67]	0.07	44.04
6	32452951	rs9268428	T	10	1.11x10-51	1.59 [1.50-1.68]	0.07	43.92
6	32453030	rs9268429	G	10	1.31x10-14	1.53 [1.38-1.70]	0.02	55.48
6	32453573	rs2395153	G	10	4.04x10-27	1.37 [1.30-1.45]	0.20	26.21
6	32455468	rs8180664	T	10	9.30x10-52	1.59 [1.50-1.68]	0.10	38.14
6	32457924	rs9268456	A	10	1.19x10-51	1.58 [1.50-1.68]	0.09	40.57
6	32458014	rs9268457	G	10	5.23x10-52	1.59 [1.50-1.68]	0.11	37.60
6	32458754	rs9268459	A	10	1.41x10-51	1.58 [1.50-1.68]	0.08	41.21
6	32458846	rs4373382	C	10	7.44x10-24	1.34 [1.27-1.41]	0.10	38.05
6	32462406	rs4424066	G	10	9.30x10-24	1.34 [1.27-1.41]	0.11	37.77
6	32462498	rs1555115	C	10	4.40x10-08	0.78 [0.72-0.85]	0.11	37.57
6	32462622	rs3129948	C	10	8.35x10-19	0.75 [0.70-0.80]	0.05	46.47
6	32463583	rs9268472	A	10	8.54x10-24	1.34 [1.27-1.41]	0.11	37.96
6	32465111	rs17423649	T	10	4.40x10-08	0.78 [0.72-0.85]	0.11	37.57
6	32465143	rs9268474	C	10	8.07x10-52	1.59 [1.50-1.68]	0.09	40.21
6	32465693	rs12529049	T	10	4.33x10-08	0.78 [0.72-0.85]	0.11	37.71
6	32466209	rs9268475	T	10	8.07x10-52	1.59 [1.50-1.68]	0.09	40.21
6	32466491	rs3117098	C	10	7.07x10-19	0.75 [0.70-0.80]	0.06	45.36
6	32466511	rs17495592	G	10	4.21x10-08	0.79 [0.72-0.86]	0.05	46.67
6	32467741	rs3129952	G	10	7.07x10-19	0.75 [0.70-0.80]	0.06	45.36
6	32468981	rs3817976	A	10	4.42x10-08	0.78 [0.72-0.85]	0.11	37.54
6	32469089	rs3817973	A	10	7.34x10-24	1.34 [1.27-1.41]	0.11	36.98
6	32469366	rs3817969	A	10	4.48x10-08	0.79 [0.72-0.86]	0.05	46.58
6	32470723	rs28362678	T	10	4.34x10-08	0.79 [0.72-0.86]	0.05	46.59
6	32471794	rs2076530	G	10	1.11x10-22	1.33 [1.26-1.40]	0.09	40.74
6	32471822	rs9268480	T	10	1.79x10-51	1.58 [1.50-1.68]	0.09	40.50
6	32471933	rs2076529	G	10	1.19x10-23	1.34 [1.27-1.41]	0.11	37.75
6	32472334	rs9268481	G	10	1.22x10-51	1.58 [1.50-1.68]	0.10	39.09
6	32475700	rs2294880	C	10	4.86x10-51	1.58 [1.49-1.67]	0.06	44.91
6	32475755	rs9268482	T	10	2.07x10-51	1.58 [1.50-1.68]	0.08	41.27
6	32475773	rs2294878	A	10	1.95x10-19	1.30 [1.23-1.37]	0.20	26.48
6	32475825	rs3817966	G	10	3.87x10-50	1.57 [1.48-1.66]	0.08	42.33
6	32475975	rs3817964	T	10	1.23x10-28	1.86 [1.67-2.06]	0.56	0.00
6	32476065	rs3817963	G	10	2.97x10-50	1.57 [1.49-1.66]	0.08	41.89
6	32476292	rs3817962	T	10	1.79x10-51	1.58 [1.50-1.68]	0.08	41.19
6	32477466	rs3763305	A	10	1.33x10-28	1.86 [1.67-2.06]	0.57	0.00
6	32478594	rs2076525	G	10	1.83x10-51	1.58 [1.50-1.68]	0.08	41.91
6	32478813	rs2076523	G	10	1.54x10-39	1.47 [1.39-1.55]	0.05	46.55
6	32479157	rs2076522	G	10	1.63x10-51	1.58 [1.50-1.68]	0.08	41.76
6	32479893	rs3793127	T	10	1.30x10-52	1.67 [1.57-1.77]	0.24	21.87
6	32481676	rs3806156	T	10	1.54x10-39	1.47 [1.39-1.55]	0.05	46.55
6	32481779	rs3806157	G	10	2.28x10-40	1.48 [1.40-1.56]	0.06	45.27
6	32482600	rs3763307	T	10	1.83x10-51	1.58 [1.50-1.68]	0.08	41.91
6	32483330	rs9268494	C	10	7.08x10-14	1.47 [1.34-1.63]	0.03	50.81
6	32483402	rs9268497	A	10	7.08x10-14	1.47 [1.34-1.63]	0.03	50.81
6	32483673	rs9268499	A	10	3.21x10-48	1.55 [1.47-1.64]	0.05	46.66
6	32483723	rs6926737	G	10	2.17x10-24	0.74 [0.70-0.78]	0.21	25.35
6	32484154	rs3763311	T	10	2.45x10-49	1.56 [1.48-1.65]	0.05	46.45
6	32484326	rs3763312	A	10	2.62x10-52	1.66 [1.56-1.77]	0.23	22.67
6	32484495	rs9268500	T	10	5.12x10-28	1.86 [1.67-2.06]	0.63	0.00
6	32485517	rs9268507	A	10	2.43x10-24	0.74 [0.70-0.78]	0.21	25.35
6	32486844	rs5007265	T	10	2.41x10-24	0.74 [0.70-0.78]	0.21	25.25
6	32486960	rs5007263	A	10	2.41x10-24	0.74 [0.70-0.78]	0.21	25.25
6	32487079	rs5007259	T	10	2.41x10-24	0.74 [0.70-0.78]	0.21	25.25
6	32487273	rs9268515	C	10	1.15x10-55	1.76 [1.65-1.89]	0.28	18.24
6	32487467	rs9268516	T	10	4.71x10-51	1.58 [1.49-1.67]	0.07	42.45

6	32488240	rs6932542	A	10	2.11x10-24	0.74 [0.70-0.78]	0.21	25.44
6	32488760	rs4502931	T	10	4.09x10-24	0.74 [0.70-0.79]	0.19	27.23
6	32489352	rs9268521	C	10	3.69x10-53	1.66 [1.56-1.77]	0.25	20.57
6	32491086	rs9268528	G	10	3.36x10-08	1.35 [1.21-1.49]	0.01	57.03
6	32491116	rs4959028	G	10	4.53x10-08	0.68 [0.59-0.78]	0.55	0.00
6	32492779	rs9268543	T	10	9.90x10-61	1.84 [1.72-1.97]	0.51	0.00
6	32495787	rs2395163	C	10	9.15x10-58	1.71 [1.61-1.82]	0.43	0.32
6	32504593	rs3135342	A	10	1.80x10-11	0.80 [0.75-0.85]	0.84	0.00
6	32505027	rs3129848	A	10	1.73x10-11	0.80 [0.75-0.85]	0.83	0.00
6	32506626	rs3129853	A	10	1.73x10-11	0.80 [0.75-0.85]	0.83	0.00
6	32507239	rs3135339	C	10	1.75x10-11	0.80 [0.75-0.85]	0.84	0.00
6	32507820	rs2395172	C	10	1.68x10-11	0.80 [0.75-0.85]	0.83	0.00
6	32508498	rs3129858	A	10	1.72x10-11	0.80 [0.75-0.85]	0.83	0.00
6	32508917	rs3129859	C	10	6.89x10-09	0.83 [0.78-0.88]	0.46	0.00
6	32510756	rs9268614	G	10	3.81x10-62	1.74 [1.63-1.85]	0.50	0.00
6	32511633	rs983561	C	10	1.98x10-11	0.80 [0.75-0.85]	0.83	0.00
6	32512355	rs3129868	A	10	1.80x10-12	0.71 [0.65-0.78]	0.56	0.00
6	32513022	rs9268626	C	10	4.63x10-10	0.63 [0.54-0.72]	0.24	21.75
6	32513054	rs2395177	C	10	2.08x10-11	0.80 [0.75-0.85]	0.83	0.00
6	32515131	rs3129872	T	10	2.43x10-11	0.80 [0.75-0.85]	0.83	0.00
6	32515288	rs2395180	G	10	6.26x10-12	0.80 [0.75-0.85]	0.88	0.00
6	32515382	rs2395181	C	10	6.26x10-12	0.80 [0.75-0.85]	0.88	0.00
6	32515446	rs3129875	C	10	6.26x10-12	0.80 [0.75-0.85]	0.88	0.00
6	32515687	rs14004	A	10	2.26x10-19	1.30 [1.23-1.38]	0.07	43.06
6	32515990	rs3129876	A	10	9.62x10-12	0.80 [0.75-0.85]	0.86	0.00
6	32516022	rs9268644	A	10	2.13x10-27	0.72 [0.68-0.76]	0.30	15.33
6	32516575	rs3129877	A	10	5.34x10-12	0.80 [0.75-0.85]	0.79	0.00
6	32516713	rs3129878	C	10	1.52x10-11	0.80 [0.76-0.85]	0.76	0.00
6	32517462	rs3129881	T	10	1.25x10-11	0.80 [0.75-0.85]	0.85	0.00
6	32517508	rs3129882	G	10	4.46x10-19	0.77 [0.73-0.81]	0.17	29.74
6	32517759	rs6931646	C	10	1.29x10-25	0.74 [0.70-0.78]	0.12	36.25
6	32517765	rs6911419	T	10	1.41x10-25	0.74 [0.70-0.78]	0.11	36.76
6	32518694	rs9268658	G	10	7.72x10-26	0.74 [0.70-0.78]	0.08	41.49
6	32518965	rs3135391	T	10	3.10x10-13	0.70 [0.63-0.76]	0.60	0.00
6	32519013	rs8084	A	10	7.57x10-09	0.84 [0.79-0.89]	0.45	0.00
6	32519501	rs2239804	A	10	1.41x10-25	0.74 [0.70-0.78]	0.11	36.76
6	32519624	rs7192	T	10	5.02x10-14	0.79 [0.75-0.84]	0.77	0.00
6	32519704	rs3129888	G	10	2.05x10-10	0.77 [0.72-0.84]	0.36	9.01
6	32519811	rs2239803	G	10	4.42x10-23	0.75 [0.71-0.79]	0.12	35.95
6	32519824	rs2239802	G	10	2.00x10-12	0.76 [0.71-0.82]	0.44	0.00
6	32520517	rs7195	A	10	5.27x10-14	0.79 [0.75-0.84]	0.78	0.00
6	32520558	rs7197	T	10	9.91x10-12	0.74 [0.69-0.81]	0.17	30.23
6	32521029	rs3135388	T	10	1.00x10-12	0.70 [0.64-0.77]	0.57	0.00
6	32521072	rs2213586	T	10	2.71x10-14	0.79 [0.75-0.84]	0.78	0.00
6	32521128	rs2213585	C	10	4.50x10-14	0.79 [0.75-0.84]	0.78	0.00
6	32521295	rs2395182	G	10	2.00x10-12	0.76 [0.71-0.82]	0.44	0.00
6	32521437	rs2227139	C	10	3.95x10-14	0.79 [0.75-0.84]	0.77	0.00
6	32521523	rs3129889	G	10	8.73x10-13	0.70 [0.64-0.77]	0.56	0.00
6	32521808	rs3763327	G	10	4.26x10-14	0.79 [0.75-0.84]	0.74	0.00
6	32528157	rs7754768	C	10	1.48x10-16	0.78 [0.73-0.82]	0.58	0.00
6	32535726	rs9268831	C	10	1.05x10-10	0.72 [0.65-0.79]	0.02	53.14
6	32535767	rs9268832	T	10	5.72x10-18	0.77 [0.72-0.81]	0.51	0.00
6	32536093	rs9268835	A	10	1.87x10-74	1.73 [1.64-1.83]	0.20	26.60
6	32536164	rs6923504	G	10	5.56x10-35	0.66 [0.62-0.71]	0.08	42.17
6	32536263	rs6903608	C	10	6.08x10-35	0.66 [0.62-0.71]	0.08	41.89
6	32536693	rs9268838	A	10	7.66x10-75	1.73 [1.64-1.83]	0.23	23.71

6	32537131	rs9268844	G	10	5.04x10-77	1.88 [1.76-2.00]	0.82	0.00
6	32537621	rs9268853	C	10	6.66x10-84	1.78 [1.68-1.88]	0.27	18.30
6	32539125	rs9268877	A	10	5.79x10-16	0.61 [0.54-0.69]	0.00	63.11
6	32539601	rs9268882	T	10	3.85x10-34	0.67 [0.63-0.71]	0.07	42.87
6	32540813	rs9268923	T	10	3.16x10-83	1.77 [1.68-1.87]	0.26	19.76
6	32541145	rs2395185	T	10	3.03x10-83	1.77 [1.68-1.87]	0.26	19.77
6	32542327	rs9268969	T	10	3.16x10-83	1.77 [1.68-1.87]	0.26	19.76
6	32546520	rs9368726	C	10	3.16x10-83	1.77 [1.68-1.87]	0.26	19.76
6	32546626	rs9405108	T	10	3.16x10-83	1.77 [1.68-1.87]	0.26	19.76
6	32547942	rs5026743	G	10	4.20x10-16	0.61 [0.54-0.69]	0.00	62.83
6	32549078	rs9269081	A	10	6.93x10-31	0.68 [0.64-0.72]	0.05	46.23
6	32551247	rs9269110	A	10	2.85x10-34	0.67 [0.63-0.71]	0.08	42.28
6	32556741	rs7748472	G	10	9.95x10-10	0.60 [0.51-0.70]	0.80	0.00
6	32557389	rs1964995	G	10	1.83x10-46	1.52 [1.44-1.60]	0.54	0.00
6	32656004	AA_DRB1_233_32656004_R	P	10	5.74x10-17	0.78 [0.73-0.82]	0.28	17.52
6	32656004	AA_DRB1_233_32656004_T	A	10	3.05x10-19	0.77 [0.72-0.81]	0.39	5.58
6	32656004	SNP_DRB1_32656004	C	10	9.25x10-18	0.77 [0.73-0.82]	0.20	26.01
6	32656559	SNP_DRB1_32656559	G	10	3.70x10-37	1.49 [1.40-1.58]	0.34	11.65
6	32657334	SNP_DRB1_32657334	C	10	7.14x10-48	1.53 [1.45-1.61]	0.59	0.00
6	32657338	AA_DRB1_180_32657338_L	P	10	6.32x10-87	2.02 [1.89-2.15]	0.92	0.00
6	32657338	AA_DRB1_180_32657338_V	A	10	9.27x10-75	1.87 [1.76-1.99]	0.85	0.00
6	32657339	SNP_DRB1_32657339	G	10	6.43x10-87	2.02 [1.89-2.15]	0.92	0.00
6	32657379	SNP_DRB1_32657379	T	10	1.69x10-15	0.69 [0.63-0.76]	0.16	30.75
6	32657430	SNP_DRB1_32657430	G	10	3.90x10-20	0.76 [0.72-0.80]	0.37	7.76
6	32657431	AA_DRB1_149_32657431_H	P	10	3.85x10-20	0.76 [0.72-0.80]	0.37	7.57
6	32657431	AA_DRB1_149_32657431_Q	A	10	4.41x10-20	0.76 [0.72-0.80]	0.37	8.09
6	32657452	AA_DRB1_142_32657452_M	P	10	1.69x10-15	0.69 [0.63-0.76]	0.16	30.75
6	32657452	AA_DRB1_142_32657452_V	A	10	2.75x10-15	0.69 [0.64-0.76]	0.13	34.97
6	32657453	SNP_DRB1_32657453	T	10	1.69x10-15	0.69 [0.63-0.76]	0.16	30.75
6	32657475	SNP_DRB1_32657475	A	10	3.90x10-20	0.76 [0.72-0.80]	0.37	7.76
6	32657479	AA_DRB1_133_32657479_L	P	10	1.69x10-15	0.69 [0.63-0.76]	0.16	30.75
6	32657479	AA_DRB1_133_32657479_R	A	10	2.75x10-15	0.69 [0.64-0.76]	0.13	34.97
6	32657479	SNP_DRB1_32657479	A	10	1.69x10-15	0.69 [0.63-0.76]	0.16	30.75
6	32657518	AA_DRB1_120_32657518_N	P	10	7.02x10-82	1.96 [1.84-2.10]	0.93	0.00
6	32657518	AA_DRB1_120_32657518_S	A	10	1.55x10-81	1.96 [1.84-2.09]	0.90	0.00
6	32657518	SNP_DRB1_32657518	T	10	7.21x10-82	1.96 [1.84-2.10]	0.92	0.00
6	32657526	SNP_DRB1_32657526	A	10	2.13x10-21	0.75 [0.71-0.80]	0.54	0.00
6	32657535	SNP_DRB1_32657535	C	9	1.84x10-13	0.42 [0.34-0.53]	0.96	0.00
6	32657542	AA_DRB1_112_32657542_H	A	9	2.24x10-12	0.43 [0.34-0.54]	0.99	0.00
6	32657542	AA_DRB1_112_32657542_Y	P	9	7.60x10-13	0.40 [0.32-0.51]	0.98	0.00
6	32657543	SNP_DRB1_32657543	A	9	7.60x10-13	0.40 [0.32-0.51]	0.98	0.00
6	32657566	AA_DRB1_104_32657566_A	P	10	1.27x10-84	1.79 [1.70-1.89]	0.27	18.49
6	32657566	AA_DRB1_104_32657566_S	A	10	1.50x10-84	1.79 [1.69-1.89]	0.26	19.84
6	32657567	SNP_DRB1_32657567	C	10	1.21x10-84	1.79 [1.70-1.89]	0.27	18.52
6	32657574	SNP_DRB1_32657574	T	10	1.69x10-15	0.69 [0.63-0.76]	0.16	30.75
6	32657584	AA_DRB1_98_32657584_E	P	10	1.27x10-84	1.79 [1.70-1.89]	0.27	18.49
6	32657584	AA_DRB1_98_32657584_K	A	10	1.26x10-84	1.79 [1.69-1.89]	0.26	20.22
6	32657585	SNP_DRB1_32657585	C	10	1.21x10-84	1.79 [1.70-1.89]	0.27	18.52
6	32657589	SNP_DRB1_32657589_T	P	10	1.21x10-16	0.68 [0.63-0.75]	0.08	42.16
6	32657590	AA_DRB1_96_32657590_H	A	10	6.88x10-09	1.19 [1.12-1.25]	0.09	40.37
6	32657590	AA_DRB1_96_32657590_HE	A	10	8.31x10-25	1.37 [1.29-1.45]	0.90	0.00
6	32657590	AA_DRB1_96_32657590_HQ	A	10	3.75x10-37	1.49 [1.40-1.58]	0.34	11.40
6	32657590	AA_DRB1_96_32657590_Hx	A	10	7.37x10-09	1.19 [1.12-1.25]	0.09	40.62
6	32657590	AA_DRB1_96_32657590_Q	P	10	1.21x10-16	0.68 [0.63-0.75]	0.08	42.16
6	32657590	AA_DRB1_96_32657590_Qx	P	10	1.98x10-16	0.69 [0.63-0.75]	0.06	44.60
6	32657590	AA_DRB1_96_32657590_QY	P	10	9.41x10-25	1.37 [1.29-1.45]	0.91	0.00

6	32657590	AA_DRB1_96_32657590_Y	P	10	6.32x10-87	2.02 [1.89-2.15]	0.92	0.00
6	32657590	AA_DRB1_96_32657590_YE	P	10	3.85x10-37	1.49 [1.40-1.58]	0.33	11.79
6	32657590	AA_DRB1_96_32657590_Yx	P	10	1.29x10-86	2.01 [1.88-2.14]	0.88	0.00
6	32657591	SNP_DRB1_32657591_A	P	10	4.34x10-87	2.02 [1.89-2.15]	0.92	0.00
6	32657591	SNP_DRB1_32657591_G	A	10	3.36x10-37	1.49 [1.41-1.58]	0.35	9.95
6	32659935	AA_DRB1_71_32659935_A	P	10	3.72x10-13	0.71 [0.65-0.77]	0.28	17.87
6	32659935	AA_DRB1_71_32659935_E	P	10	1.27x10-11	0.74 [0.68-0.80]	0.98	0.00
6	32659935	AA_DRB1_71_32659935_K	P	10	3.05x10-15	1.30 [1.22-1.38]	0.24	22.40
6	32659935	AA_DRB1_71_32659935_KR	A	10	1.20x10-27	0.68 [0.64-0.73]	0.46	0.00
6	32659935	SNP_DRB1_32659935_G	P	10	6.68x10-14	0.70 [0.64-0.76]	0.49	0.00
6	32659936	SNP_DRB1_32659936	C	10	1.09x10-27	0.68 [0.64-0.73]	0.46	0.00
6	32659938	AA_DRB1_70_32659938_Q	A	10	5.02x10-10	0.83 [0.79-0.88]	0.80	0.00
6	32659938	AA_DRB1_70_32659938_R	P	9	3.37x10-11	0.56 [0.48-0.67]	0.35	10.71
6	32659938	SNP_DRB1_32659938	C	9	3.37x10-11	0.56 [0.48-0.67]	0.35	10.71
6	32659940	SNP_DRB1_32659940	T	10	1.33x10-13	0.78 [0.74-0.83]	0.27	19.17
6	32659947	AA_DRB1_67_32659947_I	P	10	2.43x10-08	0.85 [0.80-0.90]	0.41	2.70
6	32659947	AA_DRB1_67_32659947_L	P	10	3.01x10-17	1.28 [1.21-1.35]	0.20	26.13
6	32659948	SNP_DRB1_32659948_G	P	10	3.01x10-17	1.28 [1.21-1.35]	0.20	26.13
6	32659948	SNP_DRB1_32659948_T	P	10	2.43x10-08	0.85 [0.80-0.90]	0.41	2.70
6	32659968	AA_DRB1_60_32659968_H	P	9	1.29x10-13	0.42 [0.34-0.52]	0.95	0.00
6	32659969	SNP_DRB1_32659969	G	9	1.14x10-13	0.42 [0.33-0.52]	0.95	0.00
6	32659973	SNP_DRB1_32659973_A	P	10	1.39x10-15	0.69 [0.63-0.75]	0.18	29.32
6	32659973	SNP_DRB1_32659973_C	P	10	6.20x10-10	0.75 [0.69-0.82]	0.27	19.04
6	32659973	SNP_DRB1_32659973_G	A	10	4.47x10-10	0.70 [0.63-0.78]	0.04	48.14
6	32659977	AA_DRB1_57_32659977_A	P	9	1.09x10-13	0.42 [0.33-0.52]	0.95	0.00
6	32659977	SNP_DRB1_32659977_G	P	9	1.09x10-13	0.42 [0.33-0.52]	0.95	0.00
6	32660007	AA_DRB1_47_32660007	F	10	4.89x10-27	0.73 [0.69-0.77]	0.15	32.00
6	32660007	SNP_DRB1_32660007	A	10	4.89x10-27	0.73 [0.69-0.77]	0.15	32.00
6	32660037	AA_DRB1_37_32660037_NS	P	10	1.74x10-15	0.66 [0.60-0.73]	0.03	52.60
6	32660037	AA_DRB1_37_32660037_Y	P	10	5.02x10-42	1.51 [1.43-1.60]	0.09	40.10
6	32660037	AA_DRB1_37_32660037_YF	A	10	1.06x10-15	0.68 [0.62-0.74]	0.05	47.61
6	32660037	AA_DRB1_37_32660037_YL	P	10	1.75x10-14	1.50 [1.36-1.66]	0.02	53.55
6	32660042	HLA_DRB1_04	P	10	1.40x10-86	2.02 [1.89-2.15]	0.88	0.00
6	32660042	HLA_DRB1_0401	P	10	4.04x10-33	1.72 [1.58-1.88]	0.89	0.00
6	32660042	HLA_DRB1_0404	P	10	2.37x10-57	2.51 [2.26-2.80]	0.57	0.00
6	32660042	HLA_DRB1_13	P	10	5.68x10-10	0.74 [0.67-0.81]	0.96	0.00
6	32660042	HLA_DRB1_14	P	9	1.78x10-13	0.43 [0.34-0.53]	0.95	0.00
6	32660042	HLA_DRB1_15	P	10	5.02x10-14	0.70 [0.64-0.76]	0.50	0.00
6	32660042	HLA_DRB1_1302	P	10	3.26x10-10	0.58 [0.49-0.68]	0.76	0.00
6	32660042	HLA_DRB1_1401	P	9	6.17x10-13	0.40 [0.32-0.51]	0.98	0.00
6	32660042	HLA_DRB1_1501	P	10	1.96x10-14	0.69 [0.62-0.75]	0.69	0.00
6	32660045	SNP_DRB1_32660045	T	10	5.58x10-20	1.31 [1.24-1.38]	0.35	9.98
6	32660049	AA_DRB1_33_32660049	H	10	2.10x10-86	2.00 [1.87-2.13]	0.71	0.00
6	32660050	SNP_DRB1_32660050	G	10	2.10x10-86	2.00 [1.87-2.13]	0.71	0.00
6	32660052	AA_DRB1_32_32660052	H	10	1.25x10-14	0.78 [0.73-0.83]	0.23	23.44
6	32660053	SNP_DRB1_32660053	G	10	1.25x10-14	0.78 [0.73-0.83]	0.23	23.44
6	32660090	SNP_DRB1_32660090	G	10	6.91x10-81	1.77 [1.67-1.87]	0.31	14.88
6	32660108	SNP_DRB1_32660108	C	10	1.20x10-15	0.69 [0.63-0.75]	0.17	29.99
6	32660109	AA_DRB1_13_32660109_H	P	10	2.23x10-86	2.00 [1.87-2.13]	0.72	0.00
6	32660109	AA_DRB1_13_32660109_HF	P	10	2.68x10-35	1.46 [1.38-1.55]	0.33	12.56
6	32660109	AA_DRB1_13_32660109_HG	P	10	3.77x10-68	1.77 [1.67-1.89]	0.07	42.49
6	32660109	AA_DRB1_13_32660109_HY	P	10	4.62x10-85	1.80 [1.70-1.90]	0.33	12.65
6	32660109	AA_DRB1_13_32660109_R	P	10	1.20x10-15	0.69 [0.63-0.75]	0.17	29.99
6	32660109	AA_DRB1_13_32660109_RG	P	10	8.04x10-16	0.73 [0.67-0.78]	0.16	30.89
6	32660109	AA_DRB1_13_32660109_RH	P	10	9.73x10-27	1.39 [1.31-1.47]	0.96	0.00
6	32660109	AA_DRB1_13_32660109_S	P	10	4.55x10-18	0.77 [0.72-0.81]	0.50	0.00

6	32660109	AA_DRB1_13_32660109_SF	A	10	4.04x10-35	1.43 [1.36-1.52]	0.76	0.00
6	32660109	AA_DRB1_13_32660109_SFG	A	10	3.90x10-39	1.46 [1.38-1.54]	0.73	0.00
6	32660109	AA_DRB1_13_32660109_SG	P	10	3.59x10-20	0.76 [0.72-0.80]	0.35	10.37
6	32660109	AA_DRB1_13_32660109_SR	P	10	1.90x10-43	0.67 [0.63-0.70]	0.35	9.88
6	32660109	AA_DRB1_13_32660109_SRR	A	10	4.33x10-75	1.72 [1.63-1.82]	0.10	39.38
6	32660109	AA_DRB1_13_32660109_SRG	A	10	4.15x10-48	1.53 [1.45-1.61]	0.58	0.00
6	32660109	AA_DRB1_13_32660109_SRY	A	10	2.16x10-29	1.40 [1.32-1.48]	0.09	40.55
6	32660109	AA_DRB1_13_32660109_SYF	A	10	5.31x10-22	1.33 [1.26-1.41]	0.77	0.00
6	32660109	AA_DRB1_13_32660109_SYG	A	10	2.21x10-09	1.19 [1.13-1.26]	0.15	32.20
6	32660109	SNP_DRB1_32660109_C	P	10	8.04x10-16	0.73 [0.67-0.78]	0.16	30.89
6	32660109	SNP_DRB1_32660109_G	P	10	4.55x10-18	0.77 [0.72-0.81]	0.50	0.00
6	32660109	SNP_DRB1_32660109_GA	A	10	4.04x10-35	1.43 [1.36-1.52]	0.76	0.00
6	32660109	SNP_DRB1_32660109_GC	A	10	4.15x10-48	1.53 [1.45-1.61]	0.58	0.00
6	32660109	SNP_DRB1_32660109_T	P	10	4.62x10-85	1.80 [1.70-1.90]	0.33	12.65
6	32660110	SNP_DRB1_32660110_A	A	10	5.31x10-22	1.33 [1.26-1.41]	0.77	0.00
6	32660110	SNP_DRB1_32660110_AC	A	10	8.42x10-27	1.39 [1.31-1.47]	0.96	0.00
6	32660110	SNP_DRB1_32660110_AG	A	10	9.61x10-16	0.73 [0.67-0.78]	0.17	30.34
6	32660110	SNP_DRB1_32660110_AT	A	10	2.67x10-68	1.78 [1.67-1.89]	0.07	42.83
6	32660110	SNP_DRB1_32660110_G	P	10	2.23x10-86	2.00 [1.87-2.13]	0.72	0.00
6	32660110	SNP_DRB1_32660110_T	P	10	1.20x10-15	0.69 [0.63-0.75]	0.17	29.99
6	32660111	SNP_DRB1_32660111	T	10	2.23x10-86	2.00 [1.87-2.13]	0.72	0.00
6	32660112	AA_DRB1_12_32660112	T	10	3.59x10-20	0.76 [0.72-0.80]	0.35	10.37
6	32660112	SNP_DRB1_32660112	G	10	3.59x10-20	0.76 [0.72-0.80]	0.35	10.37
6	32660115	AA_DRB1_11_32660115_P	P	10	1.20x10-15	0.69 [0.63-0.75]	0.17	29.99
6	32660115	AA_DRB1_11_32660115_PD	P	10	5.05x10-15	0.71 [0.65-0.77]	0.41	2.98
6	32660115	AA_DRB1_11_32660115_PV	P	10	5.64x10-25	1.37 [1.29-1.45]	0.90	0.00
6	32660115	AA_DRB1_11_32660115_S	P	10	3.59x10-20	0.76 [0.72-0.80]	0.35	10.37
6	32660115	AA_DRB1_11_32660115_SD	P	10	5.86x10-20	0.76 [0.72-0.81]	0.19	27.20
6	32660115	AA_DRB1_11_32660115_SG	A	10	2.21x10-09	1.19 [1.13-1.26]	0.15	32.20
6	32660115	AA_DRB1_11_32660115_SGD	A	10	2.37x10-09	1.19 [1.13-1.26]	0.09	40.45
6	32660115	AA_DRB1_11_32660115_SGL	A	10	8.79x10-25	1.36 [1.29-1.44]	0.98	0.00
6	32660115	AA_DRB1_11_32660115_SL	A	10	2.40x10-37	1.45 [1.37-1.53]	0.79	0.00
6	32660115	AA_DRB1_11_32660115_SLD	A	10	2.52x10-37	1.45 [1.37-1.53]	0.63	0.00
6	32660115	AA_DRB1_11_32660115_SP	A	10	4.15x10-48	1.53 [1.45-1.61]	0.58	0.00
6	32660115	AA_DRB1_11_32660115_SPD	A	10	3.89x10-48	1.53 [1.45-1.61]	0.59	0.00
6	32660115	AA_DRB1_11_32660115_SPG	A	10	2.25x10-35	1.47 [1.38-1.55]	0.33	12.79
6	32660115	AA_DRB1_11_32660115_SPL	A	10	4.51x10-81	1.77 [1.67-1.87]	0.30	15.87
6	32660115	AA_DRB1_11_32660115_V	P	10	2.17x10-81	1.95 [1.83-2.08]	0.76	0.00
6	32660115	AA_DRB1_11_32660115_VD	P	10	5.75x10-79	1.91 [1.79-2.03]	0.75	0.00
6	32660115	AA_DRB1_11_32660115_VG	P	10	1.06x10-81	1.78 [1.68-1.88]	0.37	8.13
6	32660115	AA_DRB1_11_32660115_VL	P	10	1.08x10-35	1.47 [1.39-1.56]	0.32	13.83
6	32660115	SNP_DRB1_32660115_A	P	10	1.08x10-35	1.47 [1.39-1.56]	0.32	13.83
6	32660115	SNP_DRB1_32660115_G	A	10	4.15x10-48	1.53 [1.45-1.61]	0.58	0.00
6	32660115	SNP_DRB1_32660115_GC	A	10	2.25x10-35	1.47 [1.38-1.55]	0.33	12.79
6	32660115	SNP_DRB1_32660115_GT	A	10	3.89x10-48	1.53 [1.45-1.61]	0.59	0.00
6	32660116	SNP_DRB1_32660116_A	P	10	3.59x10-20	0.76 [0.72-0.80]	0.35	10.37
6	32660116	SNP_DRB1_32660116_C	P	10	6.91x10-81	1.77 [1.67-1.87]	0.31	14.88
6	32660117	SNP_DRB1_32660117	G	10	3.59x10-20	0.76 [0.72-0.80]	0.35	10.37
6	32660118	AA_DRB1_10_32660118_Q	A	10	2.26x10-21	0.75 [0.71-0.80]	0.51	0.00
6	32660118	AA_DRB1_10_32660118_Y	P	10	3.59x10-20	0.76 [0.72-0.80]	0.35	10.37
6	32660119	SNP_DRB1_32660119_A	P	10	3.59x10-20	0.76 [0.72-0.80]	0.35	10.37
6	32660119	SNP_DRB1_32660119_G	A	10	2.26x10-21	0.75 [0.71-0.80]	0.51	0.00
6	32665401	SNP_DRB1_32665401	T	10	5.67x10-19	0.77 [0.72-0.81]	0.58	0.00
6	32665412	AA_DRB1_-1_32665412_A	A	10	5.03x10-15	0.72 [0.66-0.78]	0.26	20.03
6	32665412	AA_DRB1_-1_32665412_S	P	10	4.73x10-14	0.70 [0.64-0.76]	0.51	0.00
6	32665413	SNP_DRB1_32665413	A	10	5.00x10-14	0.70 [0.64-0.76]	0.51	0.00

6	32665457	AA_DRB1_-16_32665457_A	A	10	2.70x10-21	0.76 [0.71-0.80]	0.50	0.00
6	32665457	AA_DRB1_-16_32665457_V	P	10	1.54x10-18	0.77 [0.72-0.81]	0.32	12.95
6	32665457	SNP_DRB1_32665457	A	10	2.26x10-21	0.75 [0.71-0.80]	0.51	0.00
6	32665481	AA_DRB1_-24_32665481_F	P	10	1.81x10-86	2.00 [1.87-2.13]	0.71	0.00
6	32665481	AA_DRB1_-24_32665481_L	A	10	5.49x10-68	1.78 [1.67-1.89]	0.37	8.16
6	32665482	SNP_DRB1_32665482	A	10	1.22x10-86	2.00 [1.88-2.13]	0.71	0.00
6	32665484	AA_DRB1_-25_32665484_K	A	10	2.70x10-21	0.76 [0.71-0.80]	0.50	0.00
6	32665484	AA_DRB1_-25_32665484_R	P	10	1.54x10-18	0.77 [0.72-0.81]	0.32	12.95
6	32665484	SNP_DRB1_32665484	C	10	2.18x10-21	0.75 [0.71-0.80]	0.52	0.00
6	32677669	rs477515	T	10	3.74x10-85	1.80 [1.70-1.90]	0.28	17.70
6	32682038	rs9270986	A	10	1.26x10-15	0.69 [0.63-0.75]	0.17	29.87
6	32683303	rs2858867	C	10	5.33x10-17	0.77 [0.73-0.82]	0.50	0.00
6	32683961	rs12660719	G	10	5.69x10-10	0.59 [0.50-0.69]	0.57	0.00
6	32684042	rs482044	G	10	4.00x10-14	1.48 [1.34-1.63]	0.02	55.13
6	32685358	rs660895	G	10	5.08x10-71	1.82 [1.71-1.94]	0.44	0.00
6	32686030	rs532098	T	10	4.02x10-14	1.48 [1.34-1.64]	0.02	54.82
6	32687686	rs13207945	C	10	4.02x10-14	1.48 [1.34-1.64]	0.02	54.82
6	32689074	rs35759989	G	10	5.69x10-10	0.59 [0.50-0.69]	0.57	0.00
6	32689900	rs522308	A	10	1.12x10-83	1.79 [1.69-1.89]	0.19	27.52
6	32691335	rs6931277	T	10	1.57x10-86	2.02 [1.89-2.16]	0.94	0.00
6	32694832	rs9271366	G	10	1.56x10-13	0.70 [0.64-0.77]	0.40	4.00
6	32696978	rs9271488	T	10	7.05x10-83	1.78 [1.69-1.88]	0.18	28.85
6	32698811	rs2097431	T	10	1.20x10-09	0.60 [0.51-0.71]	0.00	77.56
6	32698931	rs9271588	C	10	1.28x10-32	1.41 [1.34-1.49]	0.19	27.50
6	32700178	rs9271640	T	10	7.68x10-18	0.69 [0.64-0.75]	0.21	25.59
6	32702306	rs9271775	C	10	1.67x10-15	0.70 [0.65-0.76]	0.18	28.47
6	32703061	rs3129768	G	10	8.19x10-18	0.69 [0.64-0.75]	0.21	25.26
6	32703075	rs3104389	T	10	8.72x10-83	1.78 [1.69-1.88]	0.18	28.60
6	32703201	rs9271858	A	10	1.65x10-33	1.42 [1.34-1.50]	0.86	0.00
6	32707977	rs9272105	G	10	1.79x10-38	1.46 [1.38-1.54]	0.76	0.00
6	32708781	rs9272143	T	10	4.79x10-34	1.42 [1.35-1.50]	0.88	0.00
6	32713235	SNP_DQA1_32713235	C	10	7.40x10-21	0.57 [0.50-0.63]	0.00	61.97
6	32713236	AA_DQA1_-16_32713236_L	P	10	7.40x10-21	0.57 [0.50-0.63]	0.00	61.97
6	32713236	AA_DQA1_-16_32713236_M	A	10	6.96x10-21	0.57 [0.50-0.63]	0.00	61.90
6	32713252	SNP_DQA1_32713252	T	10	8.04x10-20	0.57 [0.51-0.64]	0.01	61.14
6	32713273	SNP_DQA1_32713273	T	10	8.07x10-83	1.78 [1.69-1.88]	0.17	29.88
6	32716284	HLA_DQA1_01	P	10	7.14x10-20	0.57 [0.51-0.64]	0.01	61.08
6	32716284	HLA_DQA1_03	P	10	6.66x10-83	1.97 [1.84-2.10]	0.83	0.00
6	32716284	HLA_DQA1_0102	P	10	2.77x10-26	0.64 [0.59-0.69]	0.36	9.01
6	32716284	HLA_DQA1_0301	P	10	6.66x10-83	1.97 [1.84-2.10]	0.83	0.00
6	32717072	SNP_DQA1_32717072	T	10	6.91x10-46	1.56 [1.47-1.65]	0.07	43.45
6	32717083	AA_DQA1_11_32717083_C	P	10	8.04x10-20	0.57 [0.51-0.64]	0.01	61.14
6	32717083	AA_DQA1_11_32717083_Y	A	10	7.50x10-20	0.57 [0.51-0.64]	0.01	61.05
6	32717083	SNP_DQA1_32717083	G	10	8.04x10-20	0.57 [0.51-0.64]	0.01	61.14
6	32717084	SNP_DQA1_32717084	C	10	8.07x10-83	1.78 [1.69-1.88]	0.17	29.88
6	32717104	AA_DQA1_18_32717104	F	10	8.04x10-20	0.57 [0.51-0.64]	0.01	61.14
6	32717104	SNP_DQA1_32717104	T	10	8.04x10-20	0.57 [0.51-0.64]	0.01	61.14
6	32717108	SNP_DQA1_32717108	T	10	6.66x10-83	1.97 [1.84-2.10]	0.83	0.00
6	32717120	SNP_DQA1_32717120	G	10	6.66x10-83	1.97 [1.84-2.10]	0.83	0.00
6	32717128	AA_DQA1_26_32717128	S	10	6.66x10-83	1.97 [1.84-2.10]	0.83	0.00
6	32717128	SNP_DQA1_32717128	G	10	6.66x10-83	1.97 [1.84-2.10]	0.83	0.00
6	32717147	SNP_DQA1_32717147	C	10	8.09x10-73	1.71 [1.61-1.80]	0.11	37.19
6	32717151	SNP_DQA1_32717151	G	10	1.24x10-31	1.40 [1.33-1.48]	0.24	21.94
6	32717152	AA_DQA1_34_32717152	E	10	1.24x10-31	1.40 [1.33-1.48]	0.24	21.94
6	32717159	SNP_DQA1_32717159	T	10	8.07x10-83	1.78 [1.69-1.88]	0.17	29.88
6	32717185	AA_DQA1_45_32717185	A	10	8.04x10-20	0.57 [0.51-0.64]	0.01	61.14

6	32717185	SNP_DQA1_32717185	C	10	8.04x10-20	0.57 [0.51-0.64]	0.01	61.14
6	32717191	AA_DQA1_47_32717191_Q	P	10	6.66x10-83	1.97 [1.84-2.10]	0.83	0.00
6	32717191	AA_DQA1_47_32717191_R	P	10	8.04x10-20	0.57 [0.51-0.64]	0.01	61.14
6	32717191	AA_DQA1_47_32717191_RC	A	10	7.74x10-83	1.78 [1.69-1.88]	0.17	29.88
6	32717191	AA_DQA1_47_32717191_RK	A	10	2.42x10-12	1.48 [1.33-1.64]	0.01	60.06
6	32717191	SNP_DQA1_32717191	A	10	7.74x10-83	1.78 [1.69-1.88]	0.17	29.88
6	32717194	AA_DQA1_48_32717194	W	10	7.50x10-20	0.57 [0.51-0.64]	0.01	61.05
6	32717194	SNP_DQA1_32717194	G	10	7.50x10-20	0.57 [0.51-0.64]	0.01	61.05
6	32717199	SNP_DQA1_32717199	C	10	8.07x10-83	1.78 [1.69-1.88]	0.17	29.88
6	32717200	AA_DQA1_50_32717200_E	P	10	7.50x10-20	0.57 [0.51-0.64]	0.01	61.05
6	32717200	AA_DQA1_50_32717200_L	P	10	8.07x10-83	1.78 [1.69-1.88]	0.17	29.88
6	32717200	SNP_DQA1_32717200	A	10	7.50x10-20	0.57 [0.51-0.64]	0.01	61.05
6	32717205	SNP_DQA1_32717205	C	10	8.07x10-83	1.78 [1.69-1.88]	0.17	29.88
6	32717206	AA_DQA1_52_32717206_R	P	10	2.35x10-12	1.48 [1.33-1.64]	0.01	59.99
6	32717206	AA_DQA1_52_32717206_S	P	10	7.50x10-20	0.57 [0.51-0.64]	0.01	61.05
6	32717209	AA_DQA1_53_32717209_K	P	10	7.50x10-20	0.57 [0.51-0.64]	0.01	61.05
6	32717209	AA_DQA1_53_32717209_R	P	10	8.07x10-83	1.78 [1.69-1.88]	0.17	29.88
6	32717209	SNP_DQA1_32717209	G	10	8.07x10-83	1.78 [1.69-1.88]	0.17	29.88
6	32717214	SNP_DQA1_32717214	G	10	7.50x10-20	0.57 [0.51-0.64]	0.01	61.05
6	32717215	AA_DQA1_55_32717215	G	10	7.50x10-20	0.57 [0.51-0.64]	0.01	61.05
6	32717217	SNP_DQA1_32717217_A	P	10	6.66x10-83	1.97 [1.84-2.10]	0.83	0.00
6	32717217	SNP_DQA1_32717217_G	P	10	7.50x10-20	0.57 [0.51-0.64]	0.01	61.05
6	32717218	AA_DQA1_56_32717218_G	P	10	7.50x10-20	0.57 [0.51-0.64]	0.01	61.05
6	32717218	AA_DQA1_56_32717218_R	P	10	6.66x10-83	1.97 [1.84-2.10]	0.83	0.00
6	32717219	SNP_DQA1_32717219_A	P	10	6.66x10-83	1.97 [1.84-2.10]	0.83	0.00
6	32717219	SNP_DQA1_32717219_T	P	10	7.50x10-20	0.57 [0.51-0.64]	0.01	61.05
6	32717231	SNP_DQA1_32717231	G	10	7.50x10-20	0.57 [0.51-0.64]	0.01	61.05
6	32717232	SNP_DQA1_32717232	G	10	7.50x10-20	0.57 [0.51-0.64]	0.01	61.05
6	32717233	AA_DQA1_61_32717233	G	10	7.50x10-20	0.57 [0.51-0.64]	0.01	61.05
6	32717233	SNP_DQA1_32717233	G	10	7.50x10-20	0.57 [0.51-0.64]	0.01	61.05
6	32717242	AA_DQA1_64_32717242	R	10	7.50x10-20	0.57 [0.51-0.64]	0.01	61.05
6	32717242	SNP_DQA1_32717242	G	10	7.50x10-20	0.57 [0.51-0.64]	0.01	61.05
6	32717248	AA_DQA1_66_32717248	M	10	7.50x10-20	0.57 [0.51-0.64]	0.01	61.05
6	32717249	SNP_DQA1_32717249	G	10	7.50x10-20	0.57 [0.51-0.64]	0.01	61.05
6	32717256	SNP_DQA1_32717256_C	A	10	6.96x10-21	0.57 [0.50-0.63]	0.00	61.90
6	32717256	SNP_DQA1_32717256_G	P	10	7.50x10-20	0.57 [0.51-0.64]	0.01	61.05
6	32717257	AA_DQA1_69_32717257_A	P	10	7.50x10-20	0.57 [0.51-0.64]	0.01	61.05
6	32717257	AA_DQA1_69_32717257_L	A	10	6.96x10-21	0.57 [0.50-0.63]	0.00	61.90
6	32717257	SNP_DQA1_32717257	C	10	6.96x10-21	0.57 [0.50-0.63]	0.00	61.90
6	32717264	rs1048087	C	10	6.96x10-21	0.57 [0.50-0.63]	0.00	61.90
6	32717264	SNP_DQA1_32717264	C	10	6.96x10-21	0.57 [0.50-0.63]	0.00	61.90
6	32717277	SNP_DQA1_32717277_A	P	10	7.50x10-20	0.57 [0.51-0.64]	0.01	61.05
6	32717277	SNP_DQA1_32717277_G	P	10	6.66x10-83	1.97 [1.84-2.10]	0.83	0.00
6	32717278	AA_DQA1_76_32717278_M	P	10	7.50x10-20	0.57 [0.51-0.64]	0.01	61.05
6	32717278	AA_DQA1_76_32717278_V	P	10	6.66x10-83	1.97 [1.84-2.10]	0.83	0.00
6	32717290	AA_DQA1_80_32717290	Y	10	7.50x10-20	0.57 [0.51-0.64]	0.01	61.05
6	32717290	SNP_DQA1_32717290	A	10	7.50x10-20	0.57 [0.51-0.64]	0.01	61.05
6	32717791	SNP_DQA1_32717791	T	10	7.17x10-15	0.56 [0.49-0.65]	0.00	69.66
6	32717833	SNP_DQA1_32717833	C	10	7.87x10-83	1.78 [1.69-1.88]	0.17	29.95
6	32717850	AA_DQA1_129_32717850_H	A	10	6.88x10-15	0.56 [0.49-0.65]	0.00	69.60
6	32717850	AA_DQA1_129_32717850_Q	P	10	7.17x10-15	0.56 [0.49-0.65]	0.00	69.66
6	32717851	SNP_DQA1_32717851	G	10	7.17x10-15	0.56 [0.49-0.65]	0.00	69.66
6	32717986	SNP_DQA1_32717986	T	10	7.87x10-83	1.78 [1.69-1.88]	0.17	29.95
6	32717987	SNP_DQA1_32717987_C	P	10	7.50x10-20	0.57 [0.51-0.64]	0.01	61.05
6	32717987	SNP_DQA1_32717987_G	P	10	8.09x10-73	1.71 [1.61-1.80]	0.11	37.19
6	32717988	AA_DQA1_175_32717988_E	P	10	5.53x10-73	1.71 [1.62-1.80]	0.12	36.29

6	32717988	AA_DQA1_175_32717988_Q	P	10	7.50x10-20	0.57 [0.51-0.64]	0.01	61.05
6	32717988	AA_DQA1_175_32717988_QK	A	10	5.53x10-73	1.71 [1.62-1.80]	0.12	36.29
6	32717988	AA_DQA1_175_32717988_Qx	P	10	4.88x10-20	0.57 [0.51-0.64]	0.01	60.68
6	32718379	SNP_DQA1_32718379	A	10	6.66x10-83	1.97 [1.84-2.10]	0.83	0.00
6	32718380	AA_DQA1_187_32718380_A	A	10	8.14x10-83	1.97 [1.84-2.10]	0.83	0.00
6	32718380	AA_DQA1_187_32718380_T	P	10	6.66x10-83	1.97 [1.84-2.10]	0.83	0.00
6	32718381	SNP_DQA1_32718381	A	10	6.47x10-83	1.78 [1.69-1.88]	0.18	28.39
6	32718414	SNP_DQA1_32718414	T	10	1.05x10-10	0.82 [0.78-0.87]	0.09	39.85
6	32718439	SNP_DQA1_32718439	A	10	2.96x10-26	0.64 [0.59-0.69]	0.36	8.95
6	32718440	AA_DQA1_207_32718440_M	P	10	3.04x10-26	0.64 [0.59-0.69]	0.36	8.75
6	32718440	AA_DQA1_207_32718440_V	A	10	3.04x10-26	0.64 [0.59-0.69]	0.36	8.75
6	32718456	SNP_DQA1_32718456	G	10	6.47x10-83	1.78 [1.69-1.88]	0.18	28.39
6	32718459	SNP_DQA1_32718459	C	10	6.47x10-83	1.78 [1.69-1.88]	0.18	28.39
6	32718464	AA_DQA1_215_32718464_F	A	10	6.81x10-83	1.78 [1.69-1.88]	0.18	28.23
6	32718464	AA_DQA1_215_32718464_L	P	10	6.47x10-83	1.78 [1.69-1.88]	0.18	28.39
6	32718465	SNP_DQA1_32718465	G	10	6.47x10-83	1.78 [1.69-1.88]	0.18	28.39
6	32718473	AA_DQA1_218_32718473_Q	P	10	4.68x10-20	0.57 [0.51-0.64]	0.01	60.63
6	32718473	AA_DQA1_218_32718473_R	A	10	4.68x10-20	0.57 [0.51-0.64]	0.01	60.63
6	32718473	SNP_DQA1_32718473	A	10	4.68x10-20	0.57 [0.51-0.64]	0.01	60.63
6	32718513	SNP_DQA1_32718513	A	10	4.69x10-15	0.56 [0.49-0.65]	0.00	69.23
6	32736000	SNP_DQB1_32736000	A	10	6.98x10-20	0.57 [0.51-0.64]	0.01	61.06
6	32736002	)B1_226x227_32736002_PQG	P	10	2.01x10-11	0.52 [0.43-0.62]	0.36	8.45
6	32737115	SNP_DQB1_32737115	T	10	5.62x10-20	0.57 [0.51-0.64]	0.01	60.87
6	32737116	AA_DQB1_221_32737116_H	A	10	2.33x10-19	0.58 [0.51-0.65]	0.01	61.68
6	32737116	AA_DQB1_221_32737116_Q	P	10	3.58x10-20	0.58 [0.51-0.64]	0.01	59.84
6	32737119	AA_DQB1_220_32737119_H	A	10	3.41x10-19	0.57 [0.51-0.64]	0.00	62.64
6	32737119	AA_DQB1_220_32737119_R	P	10	3.56x10-20	0.58 [0.51-0.64]	0.01	59.83
6	32737119	SNP_DQB1_32737119	C	10	5.52x10-20	0.57 [0.51-0.64]	0.01	60.90
6	32737124	SNP_DQB1_32737124	A	10	3.31x10-14	1.59 [1.41-1.78]	0.00	65.44
6	32737139	SNP_DQB1_32737139	A	10	7.09x10-22	0.57 [0.51-0.64]	0.01	56.40
6	32737170	AA_DQB1_203_32737170_I	A	10	4.43x10-21	0.57 [0.51-0.64]	0.01	58.29
6	32737170	AA_DQB1_203_32737170_V	P	10	2.72x10-22	0.58 [0.52-0.64]	0.02	54.87
6	32737171	SNP_DQB1_32737171	C	10	7.05x10-22	0.57 [0.51-0.64]	0.01	56.39
6	32737733	AA_DQB1_185_32737733_I	P	10	3.68x10-55	1.73 [1.62-1.85]	0.32	13.21
6	32737733	AA_DQB1_185_32737733_T	A	10	2.64x10-54	1.72 [1.61-1.84]	0.35	10.29
6	32737733	SNP_DQB1_32737733	A	10	3.68x10-55	1.73 [1.62-1.85]	0.32	13.21
6	32737742	AA_DQB1_182_32737742_N	P	10	3.30x10-14	1.59 [1.41-1.78]	0.00	65.61
6	32737742	AA_DQB1_182_32737742_S	A	10	2.87x10-14	1.58 [1.41-1.77]	0.00	64.88
6	32737742	SNP_DQB1_32737742	T	10	3.45x10-14	1.59 [1.41-1.78]	0.00	65.68
6	32737780	SNP_DQB1_32737780	A	10	7.63x10-22	0.57 [0.51-0.64]	0.01	56.44
6	32737825	SNP_DQB1_32737825	A	10	1.01x10-26	0.66 [0.62-0.71]	0.52	0.00
6	32737867	SNP_DQB1_32737867	A	10	7.70x10-44	1.49 [1.42-1.58]	0.12	35.93
6	32737868	AA_DQB1_140_32737868_A	A	10	2.70x10-14	1.58 [1.41-1.77]	0.00	64.84
6	32737868	AA_DQB1_140_32737868_T	P	10	3.45x10-14	1.59 [1.41-1.78]	0.00	65.68
6	32737869	SNP_DQB1_32737869	T	10	3.45x10-14	1.59 [1.41-1.78]	0.00	65.68
6	32737882	SNP_DQB1_32737882	A	10	7.63x10-22	0.57 [0.51-0.64]	0.01	56.44
6	32737898	AA_DQB1_130_32737898_Q	P	10	1.23x10-09	0.60 [0.51-0.70]	0.58	0.00
6	32737898	AA_DQB1_130_32737898_R	A	10	5.39x10-10	0.59 [0.50-0.69]	0.61	0.00
6	32737898	SNP_DQB1_32737898	T	10	1.23x10-09	0.60 [0.51-0.70]	0.58	0.00
6	32737913	AA_DQB1_125_32737913_A	A	10	5.86x10-20	0.57 [0.51-0.64]	0.01	60.93
6	32737913	AA_DQB1_125_32737913_AS	A	10	1.28x10-26	0.67 [0.62-0.72]	0.50	0.00
6	32737913	AA_DQB1_125_32737913_Ax	A	10	2.83x10-20	0.57 [0.51-0.64]	0.01	59.70
6	32737913	AA_DQB1_125_32737913_G	P	10	2.91x10-26	0.67 [0.63-0.72]	0.53	0.00
6	32737913	SNP_DQB1_32737913	C	10	5.86x10-20	0.57 [0.51-0.64]	0.01	60.93
6	32737933	SNP_DQB1_32737933	T	10	3.38x10-14	1.59 [1.41-1.78]	0.00	65.63
6	32739039	HLA_DQB1_03	P	10	1.36x10-15	1.64 [1.46-1.85]	0.00	66.20

6	32739039	HLA_DQB1_06	P	10	4.09x10-26	0.67 [0.63-0.72]	0.48	0.00
6	32739039	HLA_DQB1_0302	P	10	1.45x10-73	2.11 [1.96-2.28]	0.79	0.00
6	32739039	HLA_DQB1_0503	P	9	7.23x10-14	0.41 [0.33-0.51]	0.91	0.00
6	32739039	HLA_DQB1_0602	P	10	5.52x10-14	0.68 [0.62-0.75]	0.13	34.75
6	32740556	SNP_DQB1_32740556	T	10	3.63x10-20	0.57 [0.51-0.64]	0.01	59.98
6	32740559	SNP_DQB1_32740559	T	10	8.28x10-20	0.57 [0.51-0.64]	0.01	61.11
6	32740565	SNP_DQB1_32740565	G	10	2.21x10-12	0.57 [0.49-0.66]	0.00	63.95
6	32740567	AA_DQB1_90_32740567_I	P	10	3.97x10-20	0.58 [0.51-0.64]	0.01	59.86
6	32740567	AA_DQB1_90_32740567_T	A	10	5.79x10-20	0.57 [0.51-0.64]	0.01	60.91
6	32740567	SNP_DQB1_32740567	A	10	8.28x10-20	0.57 [0.51-0.64]	0.01	61.11
6	32740570	AA_DQB1_89_32740570_G	P	10	3.97x10-20	0.58 [0.51-0.64]	0.01	59.86
6	32740570	AA_DQB1_89_32740570_T	A	10	5.79x10-20	0.57 [0.51-0.64]	0.01	60.91
6	32740570	SNP_DQB1_32740570	C	10	8.28x10-20	0.57 [0.51-0.64]	0.01	61.11
6	32740571	SNP_DQB1_32740571	C	10	8.28x10-20	0.57 [0.51-0.64]	0.01	61.11
6	32740576	AA_DQB1_87_32740576_F	P	10	3.26x10-16	0.72 [0.67-0.78]	0.40	4.31
6	32740576	AA_DQB1_87_32740576_L	A	10	5.79x10-20	0.57 [0.51-0.64]	0.01	60.91
6	32740576	AA_DQB1_87_32740576_LF	A	10	2.15x10-12	0.57 [0.48-0.66]	0.00	64.16
6	32740576	AA_DQB1_87_32740576_Lx	A	10	2.75x10-20	0.57 [0.51-0.64]	0.01	59.66
6	32740576	AA_DQB1_87_32740576_LY	A	10	1.27x10-16	0.72 [0.66-0.77]	0.41	3.01
6	32740576	AA_DQB1_87_32740576_Y	P	10	1.78x10-12	0.57 [0.49-0.66]	0.00	62.93
6	32740576	SNP_DQB1_32740576	T	10	2.21x10-12	0.57 [0.49-0.66]	0.00	63.95
6	32740577	SNP_DQB1_32740577	A	10	8.28x10-20	0.57 [0.51-0.64]	0.01	61.11
6	32740579	AA_DQB1_86_32740579_A	P	10	1.17x10-13	0.60 [0.53-0.69]	0.00	66.75
6	32740579	AA_DQB1_86_32740579_E	A	10	5.79x10-20	0.57 [0.51-0.64]	0.01	60.91
6	32740579	AA_DQB1_86_32740579_EA	A	10	5.39x10-10	0.59 [0.50-0.69]	0.61	0.00
6	32740579	AA_DQB1_86_32740579_EG	A	10	1.40x10-13	0.60 [0.53-0.68]	0.00	67.69
6	32740579	AA_DQB1_86_32740579_Ex	A	10	2.75x10-20	0.57 [0.51-0.64]	0.01	59.66
6	32740579	AA_DQB1_86_32740579_G	P	10	1.94x10-09	0.60 [0.51-0.71]	0.52	0.00
6	32740579	SNP_DQB1_32740579_C	P	10	1.94x10-09	0.60 [0.51-0.71]	0.52	0.00
6	32740579	SNP_DQB1_32740579_G	P	10	1.64x10-13	0.60 [0.53-0.69]	0.00	67.73
6	32740579	SNP_DQB1_32740579_T	A	10	5.79x10-20	0.57 [0.51-0.64]	0.01	60.91
6	32740582	AA_DQB1_85_32740582_L	A	10	5.79x10-20	0.57 [0.51-0.64]	0.01	60.91
6	32740582	AA_DQB1_85_32740582_V	P	10	3.97x10-20	0.58 [0.51-0.64]	0.01	59.86
6	32740583	SNP_DQB1_32740583	C	10	8.28x10-20	0.57 [0.51-0.64]	0.01	61.11
6	32740585	AA_DQB1_84_32740585_E	P	10	3.97x10-20	0.58 [0.51-0.64]	0.01	59.86
6	32740585	AA_DQB1_84_32740585_Q	A	10	5.79x10-20	0.57 [0.51-0.64]	0.01	60.91
6	32740586	SNP_DQB1_32740586	C	10	8.28x10-20	0.57 [0.51-0.64]	0.01	61.11
6	32740605	SNP_DQB1_32740605_T	P	9	9.47x10-14	0.52 [0.44-0.61]	0.10	40.84
6	32740606	AA_DQB1_77_32740606	R	10	1.37x10-09	0.83 [0.79-0.88]	0.30	16.08
6	32740606	SNP_DQB1_32740606	C	10	1.37x10-09	0.83 [0.79-0.88]	0.30	16.08
6	32740612	AA_DQB1_75_32740612	V	10	1.94x10-11	0.82 [0.77-0.87]	0.26	19.47
6	32740613	SNP_DQB1_32740613	C	10	1.94x10-11	0.82 [0.77-0.87]	0.26	19.47
6	32740615	AA_DQB1_74_32740615_E	A	10	1.66x10-11	0.82 [0.77-0.87]	0.27	18.82
6	32740615	SNP_DQB1_32740615	G	10	1.94x10-11	0.82 [0.77-0.87]	0.26	19.47
6	32740624	AA_DQB1_71_32740624_KA	P	10	2.09x10-09	0.84 [0.79-0.89]	0.33	12.77
6	32740624	AA_DQB1_71_32740624_T	A	10	1.66x10-11	0.82 [0.77-0.87]	0.27	18.82
6	32740627	AA_DQB1_70_32740627_G	P	10	6.91x10-15	0.60 [0.53-0.68]	0.00	62.87
6	32740627	AA_DQB1_70_32740627_R	A	10	4.84x10-14	0.59 [0.52-0.68]	0.00	68.85
6	32740628	SNP_DQB1_32740628	C	10	5.70x10-14	0.59 [0.52-0.68]	0.00	68.89
6	32740665	SNP_DQB1_32740665	A	10	6.26x10-14	0.63 [0.56-0.71]	0.01	59.42
6	32740666	AA_DQB1_57_32740666_A	P	10	6.67x10-44	1.50 [1.42-1.59]	0.06	44.81
6	32740666	AA_DQB1_57_32740666_AD	A	10	1.34x10-08	0.62 [0.52-0.73]	0.00	65.14
6	32740666	AA_DQB1_57_32740666_AS	P	10	3.01x10-38	1.46 [1.38-1.54]	0.09	40.37
6	32740666	AA_DQB1_57_32740666_AV	A	10	6.12x10-11	0.83 [0.78-0.88]	0.56	0.00
6	32740666	AA_DQB1_57_32740666_D	P	10	1.13x10-08	0.85 [0.80-0.90]	0.58	0.00
6	32740666	SNP_DQB1_32740666_G	P	10	6.67x10-44	1.50 [1.42-1.59]	0.06	44.81

6	32740666	SNP_DQB1_32740666_GA	A	10	6.12x10-11	0.83 [0.78-0.88]	0.56	0.00
6	32740666	SNP_DQB1_32740666_GC	P	10	3.01x10-38	1.46 [1.38-1.54]	0.09	40.37
6	32740666	SNP_DQB1_32740666_GT	A	10	1.34x10-08	0.62 [0.52-0.73]	0.00	65.14
6	32740666	SNP_DQB1_32740666_T	P	10	1.13x10-08	0.85 [0.80-0.90]	0.58	0.00
6	32740672	AA_DQB1_55_32740672_P	P	10	7.31x10-16	1.64 [1.46-1.85]	0.00	65.62
6	32740672	AA_DQB1_55_32740672_R	P	10	9.33x10-19	0.56 [0.50-0.64]	0.00	66.18
6	32740672	SNP_DQB1_32740672_C	P	10	9.33x10-19	0.56 [0.50-0.64]	0.00	66.18
6	32740672	SNP_DQB1_32740672_G	P	10	7.31x10-16	1.64 [1.46-1.85]	0.00	65.62
6	32740678	AA_DQB1_53_32740678	Q	10	8.28x10-20	0.57 [0.51-0.64]	0.01	61.11
6	32740678	SNP_DQB1_32740678	T	10	8.28x10-20	0.57 [0.51-0.64]	0.01	61.11
6	32740692	SNP_DQB1_32740692	G	10	1.12x10-16	0.71 [0.66-0.77]	0.43	0.39
6	32740695	SNP_DQB1_32740695	A	10	5.93x10-12	1.49 [1.33-1.66]	0.03	51.50
6	32740722	SNP_DQB1_32740722	T	10	7.31x10-16	1.64 [1.46-1.85]	0.00	65.62
6	32740723	AA_DQB1_38_32740723	V	10	1.04x10-09	0.83 [0.79-0.88]	0.31	14.88
6	32740723	SNP_DQB1_32740723	A	10	1.04x10-09	0.83 [0.79-0.88]	0.31	14.88
6	32740747	AA_DQB1_30_32740747_H	P	10	4.99x10-36	0.64 [0.59-0.68]	0.08	42.43
6	32740747	AA_DQB1_30_32740747_Y	A	10	1.78x10-20	0.76 [0.72-0.81]	0.30	15.53
6	32740748	SNP_DQB1_32740748_A	A	10	1.78x10-20	0.76 [0.72-0.81]	0.30	15.53
6	32740748	SNP_DQB1_32740748_G	P	10	4.99x10-36	0.64 [0.59-0.68]	0.08	42.43
6	32740755	SNP_DQB1_32740755	T	10	6.70x10-11	0.71 [0.65-0.79]	0.90	0.00
6	32740810	AA_DQB1_9_32740810_F	P	10	1.61x10-15	0.69 [0.63-0.75]	0.16	31.43
6	32740810	AA_DQB1_9_32740810_Y	A	10	3.81x10-15	0.70 [0.64-0.76]	0.11	37.74
6	32740810	AA_DQB1_9_32740810_YL	A	10	1.14x10-15	0.69 [0.63-0.75]	0.20	26.55
6	32740810	AA_DQB1_9_32740810_Yx	A	10	3.81x10-15	0.70 [0.64-0.76]	0.11	37.74
6	32740810	SNP_DQB1_32740810	A	10	5.22x10-15	0.70 [0.64-0.76]	0.09	40.15
6	32742277	AA_DQB1_-4_32742277_L	P	10	5.03x10-17	0.60 [0.53-0.67]	0.01	58.53
6	32742277	AA_DQB1_-4_32742277_V	A	10	4.54x10-21	0.57 [0.51-0.64]	0.01	58.29
6	32742278	SNP_DQB1_32742278_C	A	10	5.62x10-20	0.57 [0.51-0.64]	0.01	60.89
6	32742278	SNP_DQB1_32742278_x	P	10	2.37x10-21	0.64 [0.59-0.70]	0.95	0.00
6	32742279	SNP_DQB1_32742279_C	P	10	2.87x10-55	1.73 [1.62-1.85]	0.34	11.63
6	32742279	SNP_DQB1_32742279_x	P	10	2.37x10-21	0.64 [0.59-0.70]	0.95	0.00
6	32742280	AA_DQB1_-5_32742280_L	P	10	4.63x10-19	0.70 [0.65-0.75]	0.61	0.00
6	32742280	AA_DQB1_-5_32742280_P	A	10	4.54x10-21	0.57 [0.51-0.64]	0.01	58.29
6	32742280	AA_DQB1_-5_32742280_PL	A	10	8.21x10-11	0.56 [0.48-0.67]	0.00	68.69
6	32742280	AA_DQB1_-5_32742280_PS	A	10	5.82x10-27	0.66 [0.62-0.71]	0.50	0.00
6	32742280	AA_DQB1_-5_32742280_Px	A	10	3.98x10-17	0.60 [0.53-0.67]	0.01	58.45
6	32742280	SNP_DQB1_32742280_A	P	10	5.52x10-14	0.68 [0.62-0.75]	0.13	34.75
6	32742280	SNP_DQB1_32742280_G	A	10	5.07x10-39	0.62 [0.58-0.66]	0.85	0.00
6	32742280	SNP_DQB1_32742280_x	P	10	2.37x10-21	0.64 [0.59-0.70]	0.95	0.00
6	32742281	SNP_DQB1_32742281_G	A	10	3.41x10-39	0.63 [0.59-0.67]	0.06	45.08
6	32742281	SNP_DQB1_32742281_x	P	10	2.37x10-21	0.64 [0.59-0.70]	0.95	0.00
6	32742283	AA_DQB1_-6_32742283_S	P	10	5.03x10-17	0.60 [0.53-0.67]	0.01	58.53
6	32742283	AA_DQB1_-6_32742283_T	A	10	4.54x10-21	0.57 [0.51-0.64]	0.01	58.29
6	32742283	AA_DQB1_-6_32742283_TA	A	10	4.54x10-21	0.57 [0.51-0.64]	0.01	58.29
6	32742283	AA_DQB1_-6_32742283_Tx	A	10	3.98x10-17	0.60 [0.53-0.67]	0.01	58.45
6	32742284	SNP_DQB1_32742284_T	A	10	5.62x10-20	0.57 [0.51-0.64]	0.01	60.89
6	32742284	SNP_DQB1_32742284_TA	A	10	1.74x10-21	0.64 [0.59-0.70]	0.95	0.00
6	32742284	SNP_DQB1_32742284_TC	A	10	5.62x10-20	0.57 [0.51-0.64]	0.01	60.89
6	32742284	SNP_DQB1_32742284_x	P	10	2.37x10-21	0.64 [0.59-0.70]	0.95	0.00
6	32742291	SNP_DQB1_32742291_C	A	10	3.49x10-39	0.63 [0.59-0.67]	0.06	45.11
6	32742291	SNP_DQB1_32742291_x	P	10	2.37x10-21	0.64 [0.59-0.70]	0.95	0.00
6	32742292	AA_DQB1_-9_32742292_M	A	10	8.38x10-11	0.56 [0.48-0.67]	0.00	68.71
6	32742296	SNP_DQB1_32742296_x	P	10	2.37x10-21	0.64 [0.59-0.70]	0.95	0.00
6	32742309	SNP_DQB1_32742309_C	P	10	3.74x10-44	1.50 [1.42-1.58]	0.06	45.81
6	32742309	SNP_DQB1_32742309_CA	A	10	5.62x10-20	0.57 [0.51-0.64]	0.01	60.89
6	32742309	SNP_DQB1_32742309_x	P	10	2.37x10-21	0.64 [0.59-0.70]	0.95	0.00

6	32742319	SNP_DQB1_32742319_x	P	10	1.74x10-21	0.64 [0.59-0.70]	0.95	0.00
6	32742328	AA_DQB1_-21_32742328_D	P	10	3.98x10-17	0.60 [0.53-0.67]	0.01	58.45
6	32742328	AA_DQB1_-21_32742328_G	A	10	4.54x10-21	0.57 [0.51-0.64]	0.01	58.29
6	32742328	SNP_DQB1_32742328_C	A	10	5.62x10-20	0.57 [0.51-0.64]	0.01	60.89
6	32742328	SNP_DQB1_32742328_x	P	10	1.74x10-21	0.64 [0.59-0.70]	0.95	0.00
6	32742333	SNP_DQB1_32742333_A	P	10	5.89x10-55	1.73 [1.62-1.84]	0.32	13.43
6	32742333	SNP_DQB1_32742333_x	P	10	1.74x10-21	0.64 [0.59-0.70]	0.95	0.00
6	32742346	AA_DQB1_-27_32742346_A	A	10	8.32x10-11	0.56 [0.48-0.67]	0.00	68.69
6	32742347	SNP_DQB1_32742347_C	A	10	3.65x10-39	0.63 [0.59-0.67]	0.06	45.07
6	32742347	SNP_DQB1_32742347_x	P	10	1.75x10-21	0.64 [0.59-0.70]	0.95	0.00
6	32742351	SNP_DQB1_32742351_x	P	10	1.75x10-21	0.64 [0.59-0.70]	0.95	0.00
6	32744041	rs3828800	A	10	1.98x10-53	1.70 [1.60-1.81]	0.29	16.84
6	32759095	rs9275141	T	10	4.48x10-22	0.61 [0.56-0.68]	0.04	49.36
6	32759817	rs3021061	C	10	9.54x10-50	1.59 [1.50-1.68]	0.76	0.00
6	32761048	rs4947342	A	10	9.78x10-50	1.59 [1.50-1.68]	0.76	0.00
6	32762692	rs9275184	C	10	4.72x10-73	2.11 [1.95-2.28]	0.78	0.00
6	32765521	rs4642516	G	10	3.71x10-22	0.62 [0.56-0.68]	0.04	49.07
6	32765543	rs9275206	G	10	4.72x10-73	2.11 [1.95-2.28]	0.78	0.00
6	32765556	rs7774434	C	10	8.50x10-11	1.21 [1.14-1.28]	0.67	0.00
6	32767856	rs9275224	A	10	2.20x10-22	0.75 [0.71-0.79]	0.06	45.29
6	32768353	rs2858324	T	10	1.51x10-34	0.68 [0.64-0.72]	0.30	15.90
6	32771542	rs5000634	C	10	3.01x10-13	1.54 [1.37-1.72]	0.00	63.42
6	32771829	rs6457617	C	10	1.83x10-22	0.75 [0.71-0.79]	0.06	44.80
6	32771977	rs6457620	G	10	3.47x10-22	0.75 [0.71-0.80]	0.06	44.56
6	32772310	rs7745040	C	10	3.30x10-13	1.54 [1.37-1.72]	0.00	63.54
6	32772436	rs2647012	A	10	1.51x10-34	0.68 [0.64-0.72]	0.30	15.90
6	32774853	rs9275330	G	10	1.80x10-55	1.82 [1.69-1.95]	0.51	0.00
6	32774921	rs9275332	A	10	1.40x10-14	1.28 [1.21-1.36]	0.46	0.00
6	32775085	rs9275334	C	10	5.94x10-73	2.11 [1.95-2.27]	0.78	0.00
6	32775321	rs9275338	T	10	1.41x10-55	1.82 [1.70-1.95]	0.51	0.00
6	32776274	rs9275371	C	10	1.40x10-14	1.28 [1.21-1.36]	0.46	0.00
6	32776996	rs1612904	G	10	5.60x10-22	0.74 [0.69-0.78]	0.05	46.32
6	32777134	rs9275390	C	10	1.40x10-14	1.28 [1.21-1.36]	0.46	0.00
6	32777417	rs9275393	A	10	1.40x10-14	1.28 [1.21-1.36]	0.46	0.00
6	32778015	rs9275407	T	10	1.40x10-14	1.28 [1.21-1.36]	0.46	0.00
6	32778286	rs2856717	T	10	1.72x10-33	0.68 [0.65-0.73]	0.30	16.00
6	32778554	rs9275424	G	10	1.48x10-14	1.28 [1.21-1.36]	0.45	0.00
6	32778852	rs9275425	A	10	1.40x10-14	1.28 [1.21-1.36]	0.46	0.00
6	32778956	rs9275428	G	10	1.40x10-14	1.28 [1.21-1.36]	0.46	0.00
6	32779499	rs9275439	C	10	1.09x10-14	1.28 [1.21-1.36]	0.48	0.00
6	32780910	rs9275482	T	10	6.52x10-15	1.29 [1.21-1.37]	0.50	0.00
6	32781552	rs9275495	T	10	4.94x10-73	2.11 [1.95-2.28]	0.79	0.00
6	32782621	rs9275516	A	10	6.24x10-36	0.68 [0.64-0.72]	0.15	32.14
6	32783501	rs9275530	C	10	4.72x10-73	2.11 [1.95-2.28]	0.78	0.00
6	32783612	rs9275532	G	10	4.72x10-73	2.11 [1.95-2.28]	0.78	0.00
6	32785066	rs9275555	T	10	4.02x10-18	1.33 [1.25-1.42]	0.63	0.00
6	32786160	rs6932517	C	10	8.45x10-36	0.68 [0.64-0.72]	0.15	32.78
6	32787362	rs9275578	T	10	2.66x10-18	1.34 [1.26-1.42]	0.67	0.00
6	32787440	rs9275580	G	10	4.35x10-18	1.33 [1.25-1.42]	0.65	0.00
6	32787668	rs3129727	T	9	9.14x10-14	0.41 [0.33-0.52]	0.89	0.00
6	32788048	rs9275582	T	10	2.39x10-18	1.34 [1.26-1.43]	0.69	0.00
6	32788618	rs7764856	A	10	5.17x10-28	1.40 [1.32-1.48]	0.22	24.23
6	32789139	rs2858332	C	10	5.15x10-18	0.78 [0.73-0.82]	0.13	34.42
6	32789461	rs7454108	C	10	1.13x10-72	2.11 [1.95-2.27]	0.76	0.00
6	32789508	rs3957146	C	10	1.13x10-72	2.11 [1.95-2.27]	0.76	0.00
6	32789609	rs9275596	C	10	5.26x10-22	0.74 [0.69-0.78]	0.05	46.54

6	32789970	rs3998158	C	10	1.12x10-18	1.35 [1.27-1.44]	0.46	0.00
6	32789997	rs3998159	C	10	2.85x10-72	2.10 [1.95-2.27]	0.79	0.00
6	32790407	rs9275599	T	10	2.60x10-70	2.11 [1.95-2.28]	0.87	0.00
6	32790430	rs3104407	G	10	2.34x10-21	0.62 [0.56-0.68]	0.04	49.35
6	32792007	rs6936863	G	10	1.73x10-19	1.32 [1.25-1.40]	0.40	3.88
6	32792235	rs9275614	G	10	5.27x10-30	1.60 [1.48-1.73]	0.70	0.00
6	32792295	rs6916779	C	10	8.65x10-26	1.50 [1.39-1.61]	0.64	0.00
6	32792322	rs5024431	A	10	1.48x10-19	1.32 [1.25-1.40]	0.41	3.44
6	32793528	rs3916765	A	10	4.22x10-30	1.60 [1.48-1.73]	0.64	0.00
6	32807303	rs9276189	A	10	5.03x10-10	0.72 [0.65-0.80]	0.41	2.80
6	32850073	rs9276610	A	10	2.89x10-09	0.74 [0.67-0.81]	0.40	4.55
6	32873160	rs7767167	G	10	1.70x10-10	0.74 [0.67-0.81]	0.43	1.06
6	32894860	rs3763355	G	10	6.98x10-23	1.66 [1.51-1.83]	0.52	0.00
6	32912548	rs3819720	A	10	1.09x10-18	1.30 [1.23-1.38]	0.27	18.35
6	32912887	rs241425	T	10	3.66x10-10	0.83 [0.79-0.88]	0.20	26.69
6	32919607	rs2071543	A	10	1.21x10-14	1.36 [1.26-1.47]	0.06	44.14
6	32927495	rs2284190	C	10	4.50x10-15	1.37 [1.27-1.48]	0.05	46.69
6	32950953	rs6903433	T	10	5.12x10-15	1.37 [1.27-1.48]	0.10	38.31
6	33157346	HLA_DPB1_06	P	9	3.29x10-10	1.81 [1.51-2.17]	0.95	0.00
6	33157346	HLA_DPB1_0601	P	9	1.59x10-10	1.83 [1.52-2.19]	0.93	0.00

SNP, single nucleotide polymorphism; OR, odds ratio; CI, confidence interval

Only suggestive and significant p-values are shown. P-values were adjusted when the Cochran's Q was significant.

Supplementary Table 9. Regulatory role of genetic variants in the HLA region independently associated with GCA.

		pQTL	eQTL		sQTL	
**Variant ID**	**Nearest gene**	**Protein**	**Gene**	**Tissue**	**Gene**	**Tissue**
rs17882084	*HLA-DRB1*	-	-	-	-	-
rs1049087	*HLA-DQB1*	*C2, AGER*	*DQA1*	Blood, B cells, CD4+ T cells, CD8+ T cells, monocytes	-	-
			DQA2	Blood, B cells, CD4+ T cells, CD8+ T cells, monocytes		
			DQB1	Blood, B cells, CD4+ T cells, CD8+ T cells, monocytes, neutrophils, NK cells		
			DQB2	Blood, B cells, CD4+ T cells, monocytes		
			DRB1	Blood, B cells, CD4+ T cells, monocytes, neutrophils		
			DRB5	Blood, CD4+ T cells, CD8+ T cells, monocytes		
			C4B	Blood		
rs2856726	(intergenic)	-	*DQA1*	Blood, CD4+ T cells, monocytes	*DQA1*	Artery
			DQA2	Blood, monocytes, neutrophils	*DQA2*	-
			DQB1	Blood, CD4+ T cells, monocytes	*DQB1*	Artery, blood
			DQB2	Blood, monocytes	*DQB2*	Artery, blood
			DRB1	Blood, CD4+ T cells, CD8+ T cells, monocytes, neutrophils	*DRB1*	Blood
			DRB5	Blood, monocytes, neutrophils	*DRB5*	Artery, blood
			DRA	Blood	*DRA*	-
			NOTCH4	Blood	*NOTCH4*	-
			TAP2	Blood	*TAP2*	-
			TNXB	Blood	*TNXB*	-
rs2596501	*HLA-B*	*MICA, MICB*	*HLA-B*	Artery, blood, CD4+ T cells, monocytes	*HLA-B*	Artery, blood
			HLA-C	Artery, blood, B cells, CD4+ T cells, CD8+ T cells, monocytes	*HLA-C*	Artery, blood
			MICA	Artery, blood, monocytes	*MICA*	Blood
			MICB	Blood	*MICB*	-
			TCF19	Blood	*TCF19*	-
			NCR3	Blood	*NCR3*	-
			SKIV2L	Blood	*SKIV2L*	-
			DDAH2	Blood	*DDAH2*	-
			HCG22	Blood	*HCG22*	-
			HLA-E	-	*HLA-E*	Artery, blood
			CCHCR1	-	*CCHCR1*	Artery
			AIF1	-	*AIF1*	Blood

pQTL, protein quantitative trait loci; eQTL, expression quantitative trait loci; sQTL, splicing quantitative trait loci

Supp Table 10. Fine mapping results from PAINTOR

Chromosome	rsID	Z Score	Posterior probability
chr6	rs3127573	4.111	1.19x10-10
chr6	rs7756836	4.083	1.06x10-10
chr6	rs7757336	4.134	1.30x10-10
chr6	rs3119311	4.095	1.11x10-10
chr6	rs3120137	4.196	1.19x10-08
chr6	rs12209391	4.393	2.74x10-08
chr6	rs11751605	4.702	1.10x10-07
chr6	rs9365172	4.877	1.05x10-07
chr6	rs9355291	4.909	1.23x10-07
chr6	rs77009508	5.355	2.84x10-06
chr6	rs41272086	5.489	2.42x10-06
chr6	rs13198987	4.932	1.37x10-07
chr6	rs9355296	4.664	3.84x10-08
chr6	rs7770628	4.098	7.94x10-09
chr6	rs35499210	4.621	3.16x10-08
chr6	rs4708876	4.157	1.01x10-08
chr6	rs12212507	4.749	1.37x10-07
chr6	rs3011437	-4.677	4.08x10-08
chr6	rs1652507	4.666	3.87x10-08
chr6	rs2315129	4.745	1.90x10-09
chr6	rs1800769	4.719	4.96x10-08
chr6	rs783149	4.783	6.68x10-08
chr6	rs11751347	5.564	3.65x10-06
chr6	rs1897106	5.358	1.20x10-06
chr6	rs1590183	5.424	1.70x10-06
chr6	rs60553356	5.464	2.11x10-06
chr6	rs62439764	5.454	2.00x10-06
chr6	rs60171130	5.440	1.86x10-06
chr6	rs9347439	5.429	1.75x10-06
chr6	rs10455873	5.380	1.35x10-06
chr6	rs7772437	6.491	9.20x10-04
chr6	rs1009124	6.476	8.37x10-04
chr6	rs783144	4.225	5.57x10-09
chr6	rs113525646	6.382	4.58x10-04
chr6	rs7452732	6.376	4.42x10-04
chr6	rs1965091	6.385	4.67x10-04
chr6	rs4252045	6.414	1.35x10-03
chr6	rs2144723	6.400	5.13x10-04
chr6	rs2314851	6.239	1.88x10-04
chr6	rs9458010	-4.577	2.58x10-08
chr6	rs12212146	4.780	1.58x10-07
chr6	rs4252066	-4.378	1.07x10-08
chr6	rs4252072	-4.339	9.02x10-09
chr6	rs4252076	-4.330	8.70x10-09

chr6	rs59614420	-4.319	8.28x10-09
chr6	rs34126283	-4.279	6.99x10-09
chr6	rs9458012	-4.316	8.17x10-09
chr6	rs57539649	-4.420	1.28x10-08
chr6	rs4252078	-4.326	8.53x10-09
chr6	rs4757	-4.327	8.56x10-09
chr6	rs4252082	-4.320	8.33x10-09
chr6	rs4252086	-4.334	8.84x10-09
chr6	rs1853018	-4.337	8.96x10-09
chr6	rs1853017	-4.378	1.07x10-08
chr6	rs1853016	-4.359	9.81x10-09
chr6	rs4252087	-4.358	9.80x10-09
chr6	rs3798903	4.638	3.41x10-08
chr6	rs4252090	-4.403	1.19x10-08
chr6	rs4252092	4.610	3.00x10-08
chr6	rs4252093	-4.403	1.19x10-08
chr6	rs4252096	-4.341	9.09x10-09
chr6	rs35513104	-4.376	1.06x10-08
chr6	rs62439804	-4.341	9.09x10-09
chr6	rs62439805	-4.352	9.53x10-09
chr6	rs3757019	4.580	2.62x10-08
chr6	rs4252105	4.575	2.56x10-08
chr6	rs4252107	-4.308	7.89x10-09
chr6	rs4252109	-4.318	8.24x10-09
chr6	rs14224	4.637	3.38x10-08
chr6	rs783147	-5.803	1.40x10-05
chr6	rs1321201	4.611	3.01x10-08
chr6	rs3778217	4.642	3.47x10-08
chr6	rs1130656	4.611	3.00x10-08
chr6	rs2295368	4.595	2.80x10-08
chr6	rs13231	-4.379	1.07x10-08
chr6	rs4709458	4.624	3.19x10-08
chr6	rs4252114	-7.399	4.71x10-01
chr6	rs4252117	-4.655	3.68x10-08
chr6	rs4252120	-4.653	3.65x10-08
chr6	rs9458016	-4.614	3.04x10-08
chr6	rs1321197	-7.313	2.51x10-01
chr6	rs1897108	-7.315	2.56x10-01
chr6	rs9456578	-4.560	2.39x10-08
chr6	rs9458017	-4.650	3.60x10-08
chr6	rs4252125	-4.569	2.48x10-08
chr6	rs4252126	-4.590	2.73x10-08
chr6	rs783145	-4.822	8.04x10-08
chr6	rs4252130	-4.609	2.98x10-08
chr6	rs4252134	-4.605	2.93x10-08
chr6	rs9458019	-4.580	2.61x10-08
chr6	rs4252135	-4.673	4.00x10-08
chr6	rs1972748	-4.539	2.17x10-08

chr6	rs9458020	-4.655	3.68x10-08
chr6	rs56093624	-4.560	2.39x10-08
chr6	rs4252150	-4.481	1.68x10-08
chr6	rs4252151	-4.480	1.67x10-08
chr6	rs4252165	-4.496	1.79x10-08
chr6	rs783182	-4.445	1.43x10-08
chr6	rs28402939	-4.613	3.03x10-08
chr6	rs783180	-4.425	1.31x10-08
chr6	rs783178	-4.443	1.42x10-08
chr6	rs783177	-4.443	1.42x10-08
chr6	rs9458022	-4.560	2.38x10-08
chr6	rs783174	-4.219	5.43x10-09
chr6	rs783173	-4.205	5.11x10-09
chr6	rs783171	-4.217	5.37x10-09
chr6	rs6690	-4.235	5.81x10-09
chr6	rs783169	-4.248	6.13x10-09
chr6	rs783168	-4.208	5.17x10-09
chr6	rs11902	-4.214	5.32x10-09
chr6	rs4252181	-4.553	2.31x10-08
chr6	rs783165	-4.234	5.77x10-09
chr6	rs1084658	-4.263	6.52x10-09
chr6	rs1084655	-4.323	8.44x10-09
chr6	rs60198722	-4.514	1.94x10-08
chr6	rs1084654	-4.298	7.57x10-09
chr6	rs783162	-4.337	8.93x10-09
chr6	rs56262039	-4.527	2.06x10-08
chr6	rs783159	-4.333	8.78x10-09
chr6	rs9456580	-4.535	2.13x10-08
chr6	rs1406891	-4.347	9.32x10-09
chr6	rs1406890	-4.345	9.25x10-09
chr6	rs1247551	-4.354	9.63x10-09
chr6	rs1247552	-4.344	9.23x10-09
chr6	rs59754710	-4.425	1.31x10-08
chr6	rs1247553	-4.409	2.46x10-10
chr6	rs1247555	-4.455	1.50x10-08
chr6	rs1247556	-4.408	1.21x10-08
chr6	rs1247557	-4.420	1.28x10-08
chr6	rs1247558	-4.397	1.16x10-08
chr6	rs1740446	-4.444	1.42x10-08
chr6	rs62436701	-4.572	2.52x10-08
chr6	rs1652502	-4.264	6.56x10-09
chr6	rs1782629	-5.335	1.06x10-06
chr6	rs1611873	-5.315	9.53x10-07
chr6	rs1740445	-5.312	9.38x10-07
chr6	rs62436702	-4.602	2.89x10-08
chr6	rs1782627	-5.319	9.74x10-07
chr6	rs1621801	-4.391	1.13x10-08
chr6	rs1740443	-4.405	1.20x10-08

chr6	rs1620921	-4.416	1.26x10-08
chr6	rs1740442	-4.395	1.15x10-08
chr6	rs1614840	-4.415	1.25x10-08
chr6	rs1652482	-4.304	7.76x10-09
chr6	rs62436703	-4.559	2.38x10-08
chr6	rs62436704	-4.502	1.84x10-08
chr6	rs1652488	-5.217	5.74x10-07
chr6	rs62436705	-4.520	1.99x10-08
chr6	rs1247570	-4.433	1.36x10-08
chr6	rs1652455	-4.153	4.13x10-09
chr6	rs1740432	-4.448	1.45x10-08
chr6	rs3846778	-6.383	4.62x10-04
chr6	rs1652463	-4.201	5.03x10-09
chr6	rs45475797	-4.647	3.55x10-08
chr6	rs45485198	-4.741	5.49x10-08
chr6	rs2802356	-4.263	6.53x10-09
chr6	rs2565724	-4.177	4.56x10-09
chr6	rs2802357	-4.171	4.44x10-09
chr6	rs2802358	-4.173	4.48x10-09
chr6	rs2565725	-4.199	4.99x10-09
chr6	rs62436736	-4.532	2.11x10-08
chr6	rs1629794	-4.269	6.69x10-09
chr6	rs9346838	4.187	4.76x10-09
chr6	rs1652465	-4.256	6.33x10-09
chr6	rs1782632	-4.256	6.33x10-09
chr6	rs1652466	-4.258	6.39x10-09
chr6	rs765351	4.255	6.31x10-09
chr6	rs1247541	-4.300	7.65x10-09
chr6	rs1652471	-4.349	9.40x10-09
chr6	rs9346839	-4.247	6.10x10-09
chr6	rs1937479	-4.292	7.38x10-09
chr6	rs2489942	-4.310	7.95x10-09
chr6	rs2465846	-4.310	7.96x10-09
chr6	rs2489948	-4.489	1.74x10-08
chr6	rs2489949	-4.352	9.55x10-09
chr6	rs2489951	-4.344	9.22x10-09
chr6	rs2465863	-4.349	2.27x10-08
chr6	rs2489958	-4.386	1.11x10-08
chr6	rs2465862	-4.378	1.07x10-08
chr6	rs2489960	-4.376	1.06x10-08
chr6	rs2489961	-4.376	1.06x10-08
chr6	rs2465861	-4.376	1.06x10-08
chr6	rs9458045	-4.353	9.60x10-09
chr6	rs9458046	-4.364	2.41x10-08
chr6	rs9458047	-4.363	1.00x10-08
chr6	rs2489965	-4.354	9.63x10-09
chr6	rs2489966	-4.376	1.06x10-08
chr6	rs59942091	-4.383	1.09x10-08

chr6	rs12215233	-4.387	1.11x10-08
chr6	rs12194479	-4.372	1.04x10-08
chr6	rs12189741	-4.376	1.06x10-08
chr6	rs12191314	-4.323	8.41x10-09
chr6	rs12209829	-4.375	1.06x10-08
chr6	rs12196112	-4.397	2.79x10-08
chr6	rs67368974	-4.376	1.06x10-08
chr6	rs59536962	-4.399	1.17x10-08
chr6	rs2489968	-4.354	9.62x10-09
chr6	rs2489969	-4.376	2.54x10-08
chr6	rs11753819	-4.381	2.60x10-08
chr6	rs55968870	-4.340	9.06x10-09
chr6	rs56238146	-4.376	1.06x10-08
chr6	rs55900705	-4.371	2.49x10-08
chr6	rs66561051	-4.362	9.96x10-09
chr6	rs67613705	-4.388	1.12x10-08
chr6	rs66940842	-4.369	1.03x10-08
chr6	rs67721200	-4.354	9.63x10-09
chr6	rs62435278	-4.376	1.06x10-08
chr6	rs62435280	-4.390	2.70x10-08
chr6	rs28698869	-4.376	1.06x10-08
chr6	rs75211522	-4.384	1.10x10-08
chr6	rs28488549	-4.357	2.34x10-08
chr6	rs28715036	-4.357	9.75x10-09
chr6	rs12176296	-4.376	1.06x10-08
chr6	rs12176297	-4.436	1.37x10-08
chr6	rs12173853	-4.375	1.06x10-08
chr6	rs12173859	-4.361	2.39x10-08
chr6	rs10945699	-4.376	2.54x10-08
chr6	rs10945700	-4.350	9.47x10-09
chr6	rs10945701	-4.376	1.06x10-08
chr6	rs11753537	-4.354	9.62x10-09
chr6	rs11753103	-4.376	1.06x10-08
chr6	rs11753560	-4.351	9.49x10-09
chr6	rs10945702	-4.345	9.27x10-09
chr6	rs9355841	-4.337	8.94x10-09
chr6	rs9355842	-4.350	9.47x10-09
chr6	rs9766397	-4.334	8.82x10-09
chr6	rs13208565	-4.399	1.17x10-08
chr6	rs9766412	-4.375	1.06x10-08
chr6	rs67876803	-4.375	1.05x10-08
chr6	rs73029748	-4.348	9.37x10-09
chr6	rs73029749	-4.429	1.33x10-08
chr6	rs67591610	-4.345	9.25x10-09
chr6	rs62435284	-4.353	9.58x10-09
chr6	rs28515783	4.286	7.21x10-09
chr6	rs28533078	-4.331	8.71x10-09
chr6	rs28670241	-4.331	8.71x10-09

chr6	rs9365215	-4.325	8.49x10-09
chr6	rs9355843	-4.325	8.49x10-09
chr6	rs9365216	-4.325	2.04x10-08
chr6	rs9355844	-4.327	8.58x10-09
chr6	rs9355845	-4.325	8.49x10-09
chr6	rs9355846	-4.321	8.36x10-09
chr6	rs9355320	-4.325	8.49x10-09
chr6	rs9347447	-4.309	1.91x10-08
chr6	rs9347448	-4.325	8.49x10-09
chr6	rs9347449	-4.325	8.49x10-09
chr6	rs3949208	-4.325	8.49x10-09
chr6	rs2186128	-4.325	8.49x10-09
chr6	rs9365217	-4.338	8.97x10-09
chr6	rs9347450	-4.338	8.97x10-09
chr6	rs9365218	-4.338	8.97x10-09
chr6	rs13217026	-4.338	8.97x10-09
chr6	rs13220254	-4.324	8.46x10-09
chr6	rs13201433	-4.338	8.97x10-09
chr6	rs9365219	-4.325	2.04x10-08
chr6	rs9347451	-4.325	8.49x10-09
chr6	rs2465855	-4.314	8.11x10-09
chr6	rs9347452	-4.327	8.55x10-09
chr6	rs2489930	-4.327	8.55x10-09
chr6	rs2465856	-4.327	8.55x10-09
chr6	rs28597670	-4.317	1.97x10-08
chr6	rs28594774	-4.320	8.32x10-09
chr6	rs28448569	-4.337	8.95x10-09
chr6	rs28544460	-4.371	1.04x10-08
chr6	rs28655734	-4.327	8.57x10-09
chr6	rs28711930	-4.327	8.55x10-09
chr6	rs9458048	-4.327	8.55x10-09
chr6	rs9456599	-4.327	8.55x10-09
chr6	rs6926947	-4.508	1.89x10-08
chr6	rs9456600	-4.346	9.30x10-09
chr6	rs12196838	-4.327	8.55x10-09
chr6	rs68027339	-4.341	9.11x10-09
chr6	rs57127570	-4.327	8.55x10-09
chr6	rs60507137	-4.320	8.33x10-09
chr6	rs66542006	-4.320	8.33x10-09
chr6	rs68142848	-4.327	2.06x10-08
chr6	rs67998011	-4.224	5.53x10-09
chr6	rs67136969	-4.327	8.55x10-09
chr6	rs2465866	-4.272	1.63x10-08
chr6	rs2465865	-4.299	7.60x10-09
chr6	rs2489931	-4.376	1.06x10-08
chr6	rs2489932	-4.323	8.42x10-09
chr6	rs2465864	-4.325	8.49x10-09
chr6	rs12153973	-4.299	7.60x10-09

chr6	rs12153845	-4.341	9.09x10-09
chr6	rs7383363	-4.341	9.10x10-09
chr6	rs7381975	-4.341	9.10x10-09
chr6	rs13216274	-4.332	8.74x10-09
chr6	rs13200798	-4.338	2.16x10-08
chr6	rs13219765	-4.338	2.16x10-08
chr6	rs13192981	-4.342	9.15x10-09
chr6	rs13203412	-4.341	9.10x10-09
chr6	rs13203427	-4.341	9.10x10-09
chr6	rs7383738	-4.314	8.11x10-09
chr6	rs7382318	-4.365	1.01x10-08
chr6	rs7381442	-4.341	2.19x10-08
chr6	rs7382435	-4.316	8.18x10-09
chr6	rs7382456	-4.345	9.26x10-09
chr6	rs9365221	-4.422	1.29x10-08
chr6	rs9347455	-4.389	1.12x10-08
chr6	rs9347456	-4.404	1.19x10-08
chr6	rs9689453	-4.523	2.02x10-08
chr6	rs4709473	-4.523	2.02x10-08
chr6	rs9689560	-4.492	1.76x10-08
chr6	rs58508475	-4.521	2.01x10-08
chr6	rs13206496	-4.543	5.31x10-08
chr6	rs9365224	-4.548	2.26x10-08
chr6	rs9347462	-4.548	2.26x10-08
chr6	rs9347463	-4.553	2.31x10-08
chr6	rs9365226	-4.565	2.44x10-08
chr6	rs2465848	-4.312	8.03x10-09
chr6	rs2465849	-4.317	8.21x10-09
chr6	rs2489936	-4.204	5.10x10-09
chr6	rs2243944	-4.162	4.29x10-09
chr6	rs1806449	-4.174	4.49x10-09
chr6	rs2465850	-4.162	4.29x10-09
chr6	rs1892347	-4.160	4.24x10-09
chr6	rs1892348	-4.160	4.24x10-09
chr6	rs2489938	-4.223	5.52x10-09
chr6	rs2465857	-4.088	3.17x10-09
chr6	rs2465859	-4.091	3.20x10-09
chr6	rs5013438	-4.081	3.08x10-09
chr6	rs5013439	-4.083	3.11x10-09
chr6	rs5013440	-4.081	3.08x10-09
chr6	rs5013441	-4.081	3.08x10-09
chr6	rs2997093	-4.064	2.88x10-09
chr6	rs2997094	-4.064	2.88x10-09
chr6	rs2953494	-4.064	2.88x10-09
chr6	rs2953495	-4.064	2.88x10-09
chr6	rs2997095	-4.077	3.03x10-09
chr6	rs1247514	-6.920	1.58x10-02
chr6	rs62435345	-4.085	3.12x10-09

chr6	rs2255792	-5.733	9.39x10-06
chr6	rs1937487	-5.805	1.41x10-05
chr6	rs1937486	-5.807	1.43x10-05
chr6	rs1937485	-5.797	1.35x10-05
chr6	rs4709474	4.959	1.56x10-07
chr6	rs2186129	4.945	1.46x10-07
chr6	rs1937484	-5.565	3.67x10-06
chr6	rs2465868	-5.534	3.09x10-06
chr6	rs2489943	-5.552	3.41x10-06
chr6	rs2953492	-5.539	3.18x10-06
chr6	rs9347465	4.751	5.77x10-08
chr6	rs1782624	-5.536	3.14x10-06
chr6	rs1782625	-5.550	3.37x10-06
chr6	rs2953493	-5.431	1.77x10-06
chr6	rs1781518	4.805	7.43x10-08
chr8	rs553014	-4.795	1.21x10-08
chr8	rs10216990	-4.565	4.25x10-09
chr8	rs11778467	-5.367	2.02x10-07
chr8	rs4352802	-5.394	2.32x10-07
chr8	rs4323431	-5.597	6.81x10-07
chr8	rs2004538	-5.376	2.11x10-07
chr8	rs4585703	-5.396	2.34x10-07
chr8	rs898477	-5.396	2.34x10-07
chr8	rs13273575	-5.376	2.11x10-07
chr8	rs13249159	-5.371	2.06x10-07
chr8	rs1809285	-5.364	1.98x10-07
chr8	rs2010937	-5.364	1.98x10-07
chr8	rs898475	-5.364	1.98x10-07
chr8	rs2010903	-5.349	1.83x10-07
chr8	rs898476	-5.349	1.83x10-07
chr8	rs2010763	-5.349	1.83x10-07
chr8	rs7830136	-5.349	1.83x10-07
chr8	rs7833799	-5.349	1.83x10-07
chr8	rs3735751	-5.349	1.83x10-07
chr8	rs7109	-5.348	1.82x10-07
chr8	rs8445	-5.348	1.82x10-07
chr8	rs2010525	-5.348	1.82x10-07
chr8	rs7465	-5.326	1.62x10-07
chr8	rs1048887	-5.313	1.52x10-07
chr8	rs3735747	-5.326	1.63x10-07
chr8	rs11775492	-5.329	1.66x10-07
chr8	rs11782418	-5.358	1.92x10-07
chr8	rs11784867	-5.364	1.98x10-07
chr8	rs11136006	-5.333	1.68x10-07
chr8	rs10866860	-5.350	1.84x10-07
chr8	rs10866861	-5.350	1.84x10-07
chr8	rs12681592	-5.349	7.63x10-08
chr8	rs34283399	-5.350	1.84x10-07

chr8	rs7831555	-5.339	1.74x10-07
chr8	rs7016934	-5.370	2.04x10-07
chr8	rs2294028	-5.323	1.61x10-07
chr8	rs7013429	-5.381	2.17x10-07
chr8	rs7013435	-5.381	2.17x10-07
chr8	rs10216777	-5.387	2.23x10-07
chr8	rs1073235	-5.385	2.21x10-07
chr8	rs718422	-5.384	2.19x10-07
chr8	rs725360	-5.411	2.53x10-07
chr8	rs10100165	-5.388	2.24x10-07
chr8	rs4146307	-5.501	4.08x10-07
chr8	rs10866862	-5.502	4.10x10-07
chr8	rs11782593	-5.502	4.10x10-07
chr8	rs11782624	-5.689	1.13x10-06
chr8	rs2280884	-5.391	2.28x10-07
chr8	rs1832	-5.350	1.84x10-07
chr8	rs10101568	-5.340	1.75x10-07
chr8	rs10091801	-5.310	1.50x10-07
chr8	rs10101588	-5.348	1.83x10-07
chr8	rs12545651	-5.392	2.29x10-07
chr8	rs7831769	-5.260	1.16x10-07
chr8	rs7818903	-5.382	2.18x10-07
chr8	rs12679973	-5.387	2.24x10-07
chr8	rs3937606	-5.389	9.39x10-08
chr8	rs9693289	-5.389	2.26x10-07
chr8	rs35951849	-5.416	2.60x10-07
chr8	rs9692909	-5.396	2.34x10-07
chr8	rs10092493	-5.362	1.97x10-07
chr8	rs11787128	-5.279	8.39x10-13
chr8	rs9644049	-5.235	1.00x10+00
chr8	rs10105720	-5.235	6.48x10-13
chr8	rs2272730	-5.248	5.57x10-18
chr8	rs3802154	-5.286	5.70x10-19
chr8	rs4470991	-5.181	7.77x10-08
chr8	rs7842005	-5.168	7.27x10-08
chr8	rs9773688	-5.159	6.97x10-08
chr8	rs6558015	-5.178	7.67x10-08
chr8	rs4732754	4.426	2.32x10-09
chr8	rs10086140	-5.184	7.88x10-08
chr8	rs62498042	4.561	1.73x10-09
chr8	rs2294092	4.406	2.13x10-09
chr8	rs6997451	-5.165	7.16x10-08
chr8	rs17388370	-5.010	3.35x10-08
chr8	rs11136011	-5.055	4.17x10-08
chr8	rs10503818	-5.106	5.34x10-08
chr8	rs727813	4.211	9.39x10-10
chr8	rs3824095	4.382	1.92x10-09
chr8	rs6558018	-5.019	3.49x10-08

chr15	rs939590	-4.523	3.67x10-09
chr15	rs17791430	-4.546	4.05x10-09
chr15	rs8025485	-4.525	3.71x10-09
chr15	rs7170845	-4.547	4.07x10-09
chr15	rs7170063	-4.522	3.66x10-09
chr15	rs12917052	-4.555	4.22x10-09
chr15	rs3743395	-4.541	3.98x10-09
chr15	rs8043470	-4.655	6.58x10-09
chr15	rs8024551	-4.696	7.89x10-09
chr15	rs8029546	-4.699	9.77x10-08
chr15	rs8029559	-4.696	7.89x10-09
chr15	rs6496531	-4.737	9.49x10-09
chr15	rs6496532	-4.718	8.72x10-09
chr15	rs6496533	-4.807	1.31x10-08
chr15	rs1064085	-5.068	4.50x10-08
chr15	rs12148547	-5.083	9.81x10-10
chr15	rs10852114	-5.082	3.06x10-13
chr15	rs35729705	-5.202	1.76x10-09
chr15	rs11073821	-5.233	1.00x10+00
chr15	rs8029053	-5.453	3.14x10-07
chr17	rs241773	-4.471	2.43x10-06
chr17	rs241772	-4.541	6.77x10-05
chr17	rs58901	-4.628	2.41x10-04
chr17	rs614877	-5.362	2.91x10-04
chr17	rs241791	-4.346	7.02x10-05
chr17	rs241780	-5.299	2.10x10-04
chr17	rs241779	-4.932	4.13x10-04
chr17	rs241777	-4.981	4.26x10-05
chr17	rs171360	-4.165	3.31x10-05
chr17	rs241776	-5.367	2.98x10-04
chr17	rs241775	-5.125	8.68x10-05
chr17	rs11080054	-5.082	1.45x10-13
chr17	rs11080055	-5.253	1.66x10-04
chr17	rs3093680	-5.812	9.89x10-02
chr17	rs1007398	-5.785	8.49x10-02
chr17	rs3093692	-5.324	2.39x10-04
chr17	rs733914	-5.387	3.31x10-04
chr17	rs13469	-5.271	1.81x10-04
chr17	rs2073867	-5.252	1.65x10-04
chr17	rs708100	-5.238	1.53x10-04
chr17	rs2227736	-5.729	6.18x10-02
chr17	rs2227735	-5.722	5.97x10-02
chr17	rs704	5.946	2.13x10-01
chr17	rs2071379	-5.155	2.95x10-03
chr17	rs7212510	-5.204	1.29x10-04
chr17	rs2027993	-5.144	9.55x10-05
chr17	rs967645	-5.213	1.35x10-04
chr17	rs6505077	-5.662	4.29x10-02

chr17	rs4795433	-5.238	1.54x10-04
chr17	rs4795434	-5.395	3.45x10-04
chr17	rs4794828	-5.321	2.35x10-04
chr17	rs6505079	-5.292	2.02x10-04
chr17	rs9913833	-5.242	1.56x10-04
chr17	rs4795435	-5.785	8.48x10-02
chr17	rs10853128	-5.717	5.81x10-02
chr17	rs1128162	-5.764	7.54x10-02
chr17	rs1128161	-5.748	6.91x10-02
chr17	rs8079943	-5.643	3.85x10-02
chr17	rs8081240	-5.653	4.08x10-02
chr17	rs2239911	-5.617	1.14x10-03
chr17	rs2239910	-5.023	1.54x10-03
chr17	rs2239908	-5.675	4.61x10-02
chr17	rs2239907	-5.470	1.51x10-02
chr17	rs7212349	-4.660	5.61x10-06
chr17	rs6505081	-4.377	7.99x10-05
chr17	rs12947270	-4.118	2.74x10-05
chr17	rs11869677	-4.405	9.00x10-05
chr17	rs4795439	-4.180	3.51x10-05
chr17	rs62065287	-4.437	1.03x10-04
chr17	rs62065289	-4.484	1.27x10-04
chr17	rs62065290	-4.383	8.21x10-05
chr17	rs55828523	-4.428	9.94x10-05
chr17	rs56163914	-4.429	1.00x10-04
chr17	rs11871289	-4.410	9.19x10-05
chr17	rs55746797	-4.642	2.56x10-04

Supp Table 11. High confidence protein-protein interaction networks from STRING

Loci	GWAS proposed gene	PPi network proteins
6q23	*PLG*	C3
6q23	*PLG*	F12
6q23	*PLG*	FGA
6q23	*PLG*	FGB
6q23	*PLG*	HRG
6q23	*PLG*	KLKB1
6q23	*PLG*	LOC102723407
6q23	*PLG*	PLAT
6q23	*PLG*	PLAU
6q23	*PLG*	PLG
6q23	*PLG*	SERPINF2
8p21.1	*CCDC25*	CCDC25
8p21.1	*CLU*	APOA1
8p21.1	*CLU*	APOE
8p21.1	*CLU*	APP
8p21.1	*CLU*	BAX
8p21.1	*CLU*	CETP
8p21.1	*CLU*	CLU
8p21.1	*CLU*	HPR
8p21.1	*CLU*	PCYOX1
8p21.1	*CLU*	PON1
8p21.1	*CLU*	SELENOS
8p21.1	*CLU*	XRCC6
8p21.1	*ELP3*	ELP1
8p21.1	*ELP3*	ELP2
8p21.1	*ELP3*	ELP3
8p21.1	*ELP3*	ELP4
8p21.1	*ELP3*	ELP5
8p21.1	*ELP3*	GRWD1
8p21.1	*ELP3*	NMD3
8p21.1	*ELP3*	WDR36
8p21.1	*ELP3*	ELP6
8p21.1	*ELP3*	WDR43
8p21.1	*ESCO2*	CCNA2
8p21.1	*ESCO2*	DLGAP5
8p21.1	*ESCO2*	ESCO2
8p21.1	*ESCO2*	SMC2
8p21.1	*ESCO2*	PCNA
8p21.1	*ESCO2*	SMC3
8p21.1	*LEPROTL1*	LEPROTL1
8p21.1	*LEPROTL1*	TMEM50A
8p21.1	*LEPROTL1*	TMEM50B
8p21.1	*PBK*	BIRC5
8p21.1	*PBK*	CDCA3
8p21.1	*PBK*	CDK1

8p21.1	*PBK*	DLGAP5
8p21.1	*PBK*	KIF11
8p21.1	*PBK*	MAD2L1
8p21.1	*PBK*	MELK
8p21.1	*PBK*	NDC80
8p21.1	*PBK*	NUF2
8p21.1	*PBK*	PBK
8p21.1	*PBK*	TTK
8p21.1	*SCARA3*	SCARA3
8p21.1	*SCARA5*	FTH1
8p21.1	*SCARA5*	FTHL
8p21.1	*SCARA5*	SCARA5
8p21.1	*TMEM66*	TMEM66
15q26.1	*ACAN*	ACAN
15q26.1	*ACAN*	HLA-DRA
15q26.1	*ACAN*	TNC
15q26.1	*ACAN*	HAPLN1
15q26.1	*ACAN*	TNR
15q26.1	*ACAN*	HTRA1
15q26.1	*ACAN*	HLA-DRB5
15q26.1	*HAPLN3*	HAPLN3
15q26.1	*MFGE8*	FAU
15q26.1	*MFGE8*	HEPHL1
15q26.1	*MFGE8*	MFGE8
17q11.2	*FOXN1*	FOXN1
17q11.2	*IFT20*	CLUAP1
17q11.2	*IFT20*	HSPB11
17q11.2	*IFT20*	IFT172
17q11.2	*IFT20*	IFT20
17q11.2	*IFT20*	IFT46
17q11.2	*IFT20*	IFT57
17q11.2	*IFT20*	IFT80
17q11.2	*IFT20*	TRAF3IP1
17q11.2	*IFT20*	TTC30A
17q11.2	*IFT20*	TTC30B
17q11.2	*IFT20*	TRIP11
17q11.2	*LGALS9*	HAVCR2
17q11.2	*LGALS9*	LGALS9
17q11.2	*LGALS9*	LGALS9B
17q11.2	*LGALS9*	LGALS9C
17q11.2	*NUFIP2*	ATXN2
17q11.2	*NUFIP2*	ATXN2L
17q11.2	*NUFIP2*	DDX6
17q11.2	*NUFIP2*	FAM98A
17q11.2	*NUFIP2*	G3BP1
17q11.2	*NUFIP2*	G3BP2
17q11.2	*NUFIP2*	LSM12
17q11.2	*NUFIP2*	NUFIP2

17q11.2	*NUFIP2*	RPL11
17q11.2	*NUFIP2*	RPL19
17q11.2	*NUFIP2*	RPL8
17q11.2	*POLDIP2*	POLDIP2
17q11.2	*SARM1*	SARM1
17q11.2	*SARM1*	TICAM2
17q11.2	*SEBOX*	SEBOX
17q11.2	*SLC13A2*	SLC13A2
17q11.2	*SLC46A1*	SLC46A1
17q11.2	*TMEM199*	ATP6AP2
17q11.2	*TMEM199*	ATP6V0A2
17q11.2	*TMEM199*	ATP6V0C
17q11.2	*TMEM199*	ATP6V0D1
17q11.2	*TMEM199*	ATP6V0D2
17q11.2	*TMEM199*	ATP6V1D
17q11.2	*TMEM199*	ATP6V1E1
17q11.2	*TMEM199*	ATP6V1F
17q11.2	*TMEM199*	ATP6V1G1
17q11.2	*TMEM199*	CCDC115
17q11.2	*TMEM199*	TMEM199
17q11.2	*TMEM97*	TMEM97
17q11.2	*TNFAIP1*	CUL3
17q11.2	*TNFAIP1*	FAF1
17q11.2	*TNFAIP1*	KCTD10
17q11.2	*TNFAIP1*	KCTD13
17q11.2	*TNFAIP1*	KLHL12
17q11.2	*TNFAIP1*	KLHL20
17q11.2	*TNFAIP1*	RBX1
17q11.2	*TNFAIP1*	TNFAIP1
17q11.2	*TNFAIP1*	UBXN1
17q11.2	*TNFAIP1*	UBXN7
17q11.2	*TNFAIP1*	ZSWIM8
17q11.2	*VTN*	ITGA2B
17q11.2	*VTN*	ITGA8
17q11.2	*VTN*	ITGAV
17q11.2	*VTN*	ITGB1
17q11.2	*VTN*	ITGB3
17q11.2	*VTN*	ITGB5
17q11.2	*VTN*	ITGB8
17q11.2	*VTN*	PLAU
17q11.2	*VTN*	PLAUR
17q11.2	*VTN*	SERPINE1
17q11.2	*VTN*	VTN

Supplementary Table 12. Results of the drug repurposing analysis.

Drug ID	Name	Status	Targets	Identified target
DB01593	Zinc	approved, investigational	*BDKRB1, MGMT, ALDOA, EEF1A1, ENO1, GAPDHS, NME1, P4HB, PDIA3, PRDX1, PSPH, TPI1, TUFM, ESR1, IL3, MT2A, CCS, HDAC1, HDAC4, MPG, SEMG1, SOD1, HDAC8, SIVA1, GLRA1, MDM2, INS, UTRN, ASPA, S100A8, S100A9, MMP9, TP73, S100A2, TP53, MT3, PDCD6, DAND5, MT1A, A1BG, A2M, AGT, AHSG, APCS, APOA1, APOA2, APOA4, APOBR, APOE, APOL1, C1QB, C1QC, C1R, C1S, C3, C4B, C4BPA, C4BPB, C5, BRCC3, C8A, C8B, C8G, CFB, CFH, CFI, CLU, CP, CPN1, CPN2, DCD, DSP, F12, F13B, F2, FCN3, FGA, FN1, GSN, HBA1, HBB, HPR, HRNR, IGFALS, IGHA1, IGHM, IGKV1-17, IGLV3-21, ITIH1, ITIH2, ITIH3, ITIH4, JCHAIN, JUP, KLKB1, KNG1, KRT1, KRT10, KRT14, KRT16, KRT2, KRT5, KRT6A, KRT9, ORM2, PGLYRP2, PON1, PZP, S100A7, SEPP1, SERPINA1, SERPINA3, SERPINA4, SERPINA6, SERPIND1, SHBG, TF, TTR, VTN, APLP1, APLP2, APP, PARP1*	F12
DB06404	Human C1-esterase inhibitor	approved	*C1R, C1S, KLKB1, F12, F2, F11, PLAT*	F12
DB06689	Ethanolamine oleate	approved	*F12*	F12
DB09228	Conestat alfa	approved, investigational	*C1R, C1S, KLKB1, F12, F2, F11, PLAT*	F12
DB12598	Nafamostat	investigational	*F2, F10, F12, PRSS1, KLK1, ICAM1*	F12
DB14487	Zinc acetate	approved, investigational	*BDKRB1, MGMT, ALDOA, EEF1A1, ENO1, GAPDHS, NME1, P4HB, PDIA3, PRDX1, PSPH, TPI1, TUFM, ESR1, IL3, MT2A, CCS, HDAC1, HDAC4, MPG, SEMG1, SOD1, HDAC8, SIVA1, GLRA1, MDM2, INS, UTRN, ASPA, S100A8, S100A9, MMP9, TP73, S100A2, TP53, MT3, PDCD6, DAND5, MT1A, A1BG, A2M, AGT, AHSG, APCS, APOA1, APOA2, APOA4, APOBR, APOE, APOL1, C1QB, C1QC, C1R, C1S, C3, C4B, C4BPA, C4BPB, C5, BRCC3, C8A, C8B, C8G, CFB, CFH, CFI, CLU, CP, CPN1, CPN2, DCD, DSP, F12, F13B, F2, FCN3, FGA, FN1, GSN, HBA1, HBB, HPR, HRNR, IGFALS, IGHA1, IGHM, IGKV1-17, IGLV3-21, ITIH1, ITIH2, ITIH3, ITIH4, JCHAIN, JUP, KLKB1, KNG1, KRT1, KRT10, KRT14, KRT16, KRT2, KRT5, KRT6A, KRT9, ORM2, PGLYRP2, PON1, PZP, S100A7, SEPP1, SERPINA1, SERPINA3, SERPINA4, SERPINA6, SERPIND1, SHBG, TF, TTR, VTN, APLP1, APLP2, APP, PARP1*	F12
DB00009	Alteplase	approved	*PLG, FGA, PLAUR, SERPINE1*	FGA
DB00015	Reteplase	approved, investigational	*PLG, FGA, SERPINE1*	FGA
DB00029	Anistreplase	approved	*PLG, FGA, SERPINE1*	FGA
DB00031	Tenecteplase	approved	*PLG, FGA, PLAUR, SERPINE1, SERPINB2, CLEC3B, KRT8, ANXA2, CALR, CANX, LRP1*	FGA
DB00364	Sucralfate	approved	*PGA5, FGF2, EGF, FGA*	FGA
DB01593	Zinc	approved, investigational	*BDKRB1, MGMT, ALDOA, EEF1A1, ENO1, GAPDHS, NME1, P4HB, PDIA3, PRDX1, PSPH, TPI1, TUFM, ESR1, IL3, MT2A, CCS, HDAC1, HDAC4, MPG, SEMG1, SOD1, HDAC8, SIVA1, GLRA1, MDM2, INS, UTRN, ASPA, S100A8, S100A9, MMP9, TP73, S100A2, TP53, MT3, PDCD6, DAND5, MT1A, A1BG, A2M, AGT, AHSG, APCS, APOA1, APOA2, APOA4, APOBR, APOE, APOL1, C1QB, C1QC, C1R, C1S, C3, C4B, C4BPA, C4BPB, C5, BRCC3, C8A, C8B, C8G, CFB, CFH, CFI, CLU, CP, CPN1, CPN2, DCD, DSP, F12, F13B, F2, FCN3, FGA, FN1, GSN, HBA1, HBB, HPR, HRNR, IGFALS, IGHA1, IGHM, IGKV1-17, IGLV3-21, ITIH1, ITIH2, ITIH3, ITIH4, JCHAIN, JUP, KLKB1, KNG1, KRT1, KRT10, KRT14, KRT16, KRT2, KRT5, KRT6A, KRT9, ORM2, PGLYRP2, PON1, PZP, S100A7, SEPP1, SERPINA1, SERPINA3, SERPINA4, SERPINA6, SERPIND1, SHBG, TF, TTR, VTN, APLP1, APLP2, APP, PARP1*	FGA
DB04919	Alfimeprase	investigational	*FGA, FGB*	FGA
DB05099	Ancrod	approved, investigational	*FGA*	FGA

DB05675	EP-2104R	investigational	*FGA*	FGA
DB06245	Lanoteplase	investigational	*PLAUR, FGA, KLK1, LAMA5, F10, FN1, SERPINB2, CLEC3B, KRT8, ANXA2, LAMB1, LAMC1, LAMA1, CALR, CANX, LRP1, LAMA3*	FGA
DB11300	Thrombin	approved, investigational	*F2R, F2RL3, F11, F13A1, F13B, FGA, FGB, FGG, F5, F8*	FGA
DB11311	Prothrombin	approved	*FGA, FGB, F13A1, CPB2*	FGA
DB11571	Human thrombin	approved	*F5, F8, F11, F13A1, F13B, FGA, FGB, FGG*	FGA
DB11572	Thrombin alfa	approved	*F5, F8, FGA, FGB, FGG*	FGA
DB13151	Anti-inhibitor coagulant complex	approved, investigational	*F10, F2, FGA, FGB, F13A1, F5, F8, F7*	FGA
DB14487	Zinc acetate	approved, investigational	*BDKRB1, MGMT, ALDOA, EEF1A1, ENO1, GAPDHS, NME1, P4HB, PDIA3, PRDX1, PSPH, TPI1, TUFM, ESR1, IL3, MT2A, CCS, HDAC1, HDAC4, MPG, SEMG1, SOD1, HDAC8, SIVA1, GLRA1, MDM2, INS, UTRN, ASPA, S100A8, S100A9, MMP9, TP73, S100A2, TP53, MT3, PDCD6, DAND5, MT1A, A1BG, A2M, AGT, AHSG, APCS, APOA1, APOA2, APOA4, APOBR, APOE, APOL1, C1QB, C1QC, C1R, C1S, C3, C4B, C4BPA, C4BPB, C5, BRCC3, C8A, C8B, C8G, CFB, CFH, CFI, CLU, CP, CPN1, CPN2, DCD, DSP, F12, F13B, F2, FCN3, FGA, FN1, GSN, HBA1, HBB, HPR, HRNR, IGFALS, IGHA1, IGHM, IGKV1-17, IGLV3-21, ITIH1, ITIH2, ITIH3, ITIH4, JCHAIN, JUP, KLKB1, KNG1, KRT1, KRT10, KRT14, KRT16, KRT2, KRT5, KRT6A, KRT9, ORM2, PGLYRP2, PON1, PZP, S100A7, SEPP1, SERPINA1, SERPINA3, SERPINA4, SERPINA6, SERPIND1, SHBG, TF, TTR, VTN, APLP1, APLP2, APP, PARP1*	FGA
DB14533	Zinc chloride	approved, investigational	*BDKRB1, MGMT, ALDOA, EEF1A1, ENO1, GAPDHS, NME1, P4HB, PDIA3, PRDX1, PSPH, TPI1, TUFM, ESR1, IL3, MT2A, CCS, HDAC1, HDAC4, MPG, SEMG1, SOD1, HDAC8, SIVA1, GLRA1, MDM2, INS, UTRN, ASPA, S100A8, S100A9, MMP9, TP73, S100A2, TP53, MT3, MT1A, A2M, AHSG, APOA1, APOBR, APOE, APOL1, C1QB, C1QC, C1R, C1S, C3, C4B, C4BPA, C4BPB, C5, BRCC3, CFH, CLU, CP, CPN1, CPN2, DCD, FGA, FN1, HBA1, HBB, HP, IGFALS, ITIH1, ITIH2, ITIH3, ITIH4, JCHAIN, KLKB1, KNG1, KRT9, ORM2, PGLYRP2, PON1, PZP, S100A7, SEPP1, SERPINA1, SERPINA3, SERPINA6, SERPIND1, SHBG, TF, TTR, VTN, APLP1, APLP2, APP, PARP1, APOB, HPX, HRG, F11, APOBEC1, SEMG2*	FGA
DB14548	Zinc sulfate, unspecified form	approved, experimental	*BDKRB1, MGMT, ALDOA, EEF1A1, ENO1, GAPDHS, NME1, P4HB, PDIA3, PRDX1, PSPH, TPI1, TUFM, ESR1, IL3, MT2A, CCS, HDAC1, HDAC4, MPG, SEMG1, SOD1, HDAC8, SIVA1, GLRA1, MDM2, INS, UTRN, ASPA, S100A8, S100A9, MMP9, TP73, S100A2, TP53, MT3, MT1A, A2M, AHSG, APOA1, APOBR, APOE, APOL1, C1QB, C1QC, C1R, C1S, C3, C4B, C4BPA, C4BPB, C5, BRCC3, CFH, CLU, CP, CPN1, CPN2, DCD, FGA, FN1, HBA1, HBB, HP, IGFALS, ITIH1, ITIH2, ITIH3, ITIH4, JCHAIN, KLKB1, KNG1, KRT9, ORM2, PGLYRP2, PON1, PZP, S100A7, SEPP1, SERPINA1, SERPINA3, SERPINA6, SERPIND1, SHBG, TF, TTR, VTN, APLP1, APLP2, APP, PARP1, APOB, HPX, HRG, F11, APOBEC1, SEMG2*	FGA
DB04919	Alfimeprase	investigational	*FGA, FGB*	FGB
DB11300	Thrombin	approved, investigational	*F2R, F2RL3, F11, F13A1, F13B, FGA, FGB, FGG, F5, F8*	FGB
DB11311	Prothrombin	approved	*FGA, FGB, F13A1, CPB2*	FGB
DB11571	Human thrombin	approved	*F5, F8, F11, F13A1, F13B, FGA, FGB, FGG*	FGB
DB11572	Thrombin alfa	approved	*F5, F8, FGA, FGB, FGG*	FGB
DB13151	Anti-inhibitor coagulant complex	approved, investigational	*F10, F2, FGA, FGB, F13A1, F5, F8, F7*	FGB

DB14533	Zinc chloride	approved, investigational	*BDKRB1, MGMT, ALDOA, EEF1A1, ENO1, GAPDHS, NME1, P4HB, PDIA3, PRDX1, PSPH, TPI1, TUFM, ESR1, IL3, MT2A, CCS, HDAC1, HDAC4, MPG, SEMG1, SOD1, HDAC8, SIVA1, GLRA1, MDM2, INS, UTRN, ASPA, S100A8, S100A9, MMP9, TP73, S100A2, TP53, MT3, MT1A, A2M, AHSG, APOA1, APOBR, APOE, APOL1, C1QB, C1QC, C1R, C1S, C3, C4B, C4BPA, C4BPB, C5, BRCC3, CFH, CLU, CP, CPN1, CPN2, DCD, FGA, FN1, HBA1, HBB, HP, IGFALS, ITIH1, ITIH2, ITIH3, ITIH4, JCHAIN, KLKB1, KNG1, KRT9, ORM2, PGLYRP2, PON1, PZP, S100A7, SEPP1, SERPINA1, SERPINA3, SERPINA6, SERPIND1, SHBG, TF, TTR, VTN, APLP1, APLP2, APP, PARP1, APOB, HPX, HRG, F11, APOBEC1, SEMG2*	HRG
DB14548	Zinc sulfate, unspecified form	approved, experimental	*BDKRB1, MGMT, ALDOA, EEF1A1, ENO1, GAPDHS, NME1, P4HB, PDIA3, PRDX1, PSPH, TPI1, TUFM, ESR1, IL3, MT2A, CCS, HDAC1, HDAC4, MPG, SEMG1, SOD1, HDAC8, SIVA1, GLRA1, MDM2, INS, UTRN, ASPA, S100A8, S100A9, MMP9, TP73, S100A2, TP53, MT3, MT1A, A2M, AHSG, APOA1, APOBR, APOE, APOL1, C1QB, C1QC, C1R, C1S, C3, C4B, C4BPA, C4BPB, C5, BRCC3, CFH, CLU, CP, CPN1, CPN2, DCD, FGA, FN1, HBA1, HBB, HP, IGFALS, ITIH1, ITIH2, ITIH3, ITIH4, JCHAIN, KLKB1, KNG1, KRT9, ORM2, PGLYRP2, PON1, PZP, S100A7, SEPP1, SERPINA1, SERPINA3, SERPINA6, SERPIND1, SHBG, TF, TTR, VTN, APLP1, APLP2, APP, PARP1, APOB, HPX, HRG, F11, APOBEC1, SEMG2*	HRG
DB01593	Zinc	approved, investigational	*BDKRB1, MGMT, ALDOA, EEF1A1, ENO1, GAPDHS, NME1, P4HB, PDIA3, PRDX1, PSPH, TPI1, TUFM, ESR1, IL3, MT2A, CCS, HDAC1, HDAC4, MPG, SEMG1, SOD1, HDAC8, SIVA1, GLRA1, MDM2, INS, UTRN, ASPA, S100A8, S100A9, MMP9, TP73, S100A2, TP53, MT3, PDCD6, DAND5, MT1A, A1BG, A2M, AGT, AHSG, APCS, APOA1, APOA2, APOA4, APOBR, APOE, APOL1, C1QB, C1QC, C1R, C1S, C3, C4B, C4BPA, C4BPB, C5, BRCC3, C8A, C8B, C8G, CFB, CFH, CFI, CLU, CP, CPN1, CPN2, DCD, DSP, F12, F13B, F2, FCN3, FGA, FN1, GSN, HBA1, HBB, HPR, HRNR, IGFALS, IGHA1, IGHM, IGKV1-17, IGLV3-21, ITIH1, ITIH2, ITIH3, ITIH4, JCHAIN, JUP, KLKB1, KNG1, KRT1, KRT10, KRT14, KRT16, KRT2, KRT5, KRT6A, KRT9, ORM2, PGLYRP2, PON1, PZP, S100A7, SEPP1, SERPINA1, SERPINA3, SERPINA4, SERPINA6, SERPIND1, SHBG, TF, TTR, VTN, APLP1, APLP2, APP, PARP1*	KLKB1
DB05311	Ecallantide	approved, investigational	*KLKB1*	KLKB1
DB06404	Human C1-esterase inhibitor	approved	*C1R, C1S, KLKB1, F12, F2, F11, PLAT*	KLKB1
DB09228	Conestat alfa	approved, investigational	*C1R, C1S, KLKB1, F12, F2, F11, PLAT*	KLKB1
DB12831	Gabexate	investigational	*KLKB1, F2, PLG*	KLKB1
DB14487	Zinc acetate	approved, investigational	*BDKRB1, MGMT, ALDOA, EEF1A1, ENO1, GAPDHS, NME1, P4HB, PDIA3, PRDX1, PSPH, TPI1, TUFM, ESR1, IL3, MT2A, CCS, HDAC1, HDAC4, MPG, SEMG1, SOD1, HDAC8, SIVA1, GLRA1, MDM2, INS, UTRN, ASPA, S100A8, S100A9, MMP9, TP73, S100A2, TP53, MT3, PDCD6, DAND5, MT1A, A1BG, A2M, AGT, AHSG, APCS, APOA1, APOA2, APOA4, APOBR, APOE, APOL1, C1QB, C1QC, C1R, C1S, C3, C4B, C4BPA, C4BPB, C5, BRCC3, C8A, C8B, C8G, CFB, CFH, CFI, CLU, CP, CPN1, CPN2, DCD, DSP, F12, F13B, F2, FCN3, FGA, FN1, GSN, HBA1, HBB, HPR, HRNR, IGFALS, IGHA1, IGHM, IGKV1-17, IGLV3-21, ITIH1, ITIH2, ITIH3, ITIH4, JCHAIN, JUP, KLKB1, KNG1, KRT1, KRT10, KRT14, KRT16, KRT2, KRT5, KRT6A, KRT9, ORM2, PGLYRP2, PON1, PZP, S100A7, SEPP1, SERPINA1, SERPINA3, SERPINA4, SERPINA6, SERPIND1, SHBG, TF, TTR, VTN, APLP1, APLP2, APP, PARP1*	KLKB1
DB14533	Zinc chloride	approved, investigational	*BDKRB1, MGMT, ALDOA, EEF1A1, ENO1, GAPDHS, NME1, P4HB, PDIA3, PRDX1, PSPH, TPI1, TUFM, ESR1, IL3, MT2A, CCS, HDAC1, HDAC4, MPG, SEMG1, SOD1, HDAC8, SIVA1, GLRA1, MDM2, INS, UTRN, ASPA, S100A8, S100A9, MMP9, TP73, S100A2, TP53, MT3, MT1A, A2M, AHSG, APOA1, APOBR, APOE, APOL1, C1QB, C1QC, C1R, C1S, C3, C4B, C4BPA, C4BPB, C5, BRCC3, CFH, CLU, CP, CPN1, CPN2, DCD, FGA, FN1, HBA1, HBB, HP, IGFALS, ITIH1, ITIH2, ITIH3, ITIH4, JCHAIN, KLKB1, KNG1, KRT9, ORM2, PGLYRP2, PON1, PZP, S100A7, SEPP1, SERPINA1, SERPINA3, SERPINA6, SERPIND1, SHBG, TF, TTR, VTN, APLP1, APLP2, APP, PARP1, APOB, HPX, HRG, F11, APOBEC1, SEMG2*	KLKB1

DB14548	Zinc sulfate, unspecified form	approved, experimental	*BDKRB1, MGMT, ALDOA, EEF1A1, ENO1, GAPDHS, NME1, P4HB, PDIA3, PRDX1, PSPH, TPI1, TUFM, ESR1, IL3, MT2A, CCS, HDAC1, HDAC4, MPG, SEMG1, SOD1, HDAC8, SIVA1, GLRA1, MDM2, INS, UTRN, ASPA, S100A8, S100A9, MMP9, TP73, S100A2, TP53, MT3, MT1A, A2M, AHSG, APOA1, APOBR, APOE, APOL1, C1QB, C1QC, C1R, C1S, C3, C4B, C4BPA, C4BPB, C5, BRCC3, CFH, CLU, CP, CPN1, CPN2, DCD, FGA, FN1, HBA1, HBB, HP, IGFALS, ITIH1, ITIH2, ITIH3, ITIH4, JCHAIN, KLKB1, KNG1, KRT9, ORM2, PGLYRP2, PON1, PZP, S100A7, SEPP1, SERPINA1, SERPINA3, SERPINA6, SERPIND1, SHBG, TF, TTR, VTN, APLP1, APLP2, APP, PARP1, APOB, HPX, HRG, F11, APOBEC1, SEMG2*	KLKB1
DB14597	Lanadelumab	approved, investigational	*KLKB1*	KLKB1
DB15982	Berotralstat	approved	*KLKB1*	KLKB1
DB00513	Aminocaproic acid	approved, investigational	*PLG, PLAT, LPA*	PLAT
DB01088	Iloprost	approved, investigational	*PTGIR, PTGER1, PDE4A, PDE4B, PDE4C, PDE4D, PLAT, PTGDR2*	PLAT
DB06404	Human C1-esterase inhibitor	approved	*C1R, C1S, KLKB1, F12, F2, F11, PLAT*	PLAT
DB07684	5-(DIMETHYLAMINO)-2-NAPHTHALENESULFONIC ACID	experimental	*PLAT*	PLAT
DB09213	Dexibuprofen	approved, investigational	*PTGS2, PTGS1, BCL2, THBD, PLAT, FABP2, PPARG, CFTR, SLC15A1, PPARA, GP1BA, S100A7*	PLAT
DB09228	Conestat alfa	approved, investigational	*C1R, C1S, KLKB1, F12, F2, F11, PLAT*	PLAT
DB00594	Amiloride	approved	*SCNN1A, SCNN1B, SCNN1G, SCNN1D, AOC1, ASIC2, ASIC1, SLC9A1, PLAU*	PLAU
DB01725	2-{2-hydroxy-[1,1-biphenyl]-3-yl}-1H-1,3-benzodiazole-5-carboximidamide	experimental	*PRSS1, F2, PLAU*	PLAU
DB01905	2-(2-Hydroxy-5-Methoxy-Phenyl)-1h-Benzoimidazole-5-Carboxamidine	experimental	*PRSS1, PLAU*	PLAU
DB01977	6-(N-Phenylcarbamyl)-2-Naphthalenecarboxamidine	experimental	*PLAU*	PLAU
DB02287	2-(2-hydroxy-phenyl)-3H-benzoimidazole-5-carboxamidine	experimental	*F2, PRSS1, PLAU*	PLAU
DB02398	6-[N-(4-(Aminomethyl)Phenyl)Carbamyl]-2-Naphthalenecarboxamidine	experimental	*PLAU*	PLAU
DB02473	6-[N-(1-Isopropyl-3,4-Dihydro-7-Isoquinolinyl)Carbamyl]-2-Naphthalenecarboxamidine	experimental	*PLAU*	PLAU
DB02526	CRA_10655	experimental	*PRSS1, PLAU*	PLAU
DB02551	6-[N-(4-Ethyl-1,2,3,4-Tetrahydro-6-Isoquinolinyl)Carbamyl]-2-Naphthalenecarboxamidine	experimental	*PLAU*	PLAU
DB02705	6-[N-(1-Isopropyl-1,2,3,4-Tetrahydro-7-Isoquinolinyl)Carbamyl]-2-Naphthalenecarboxamidine	experimental	*PLAU*	PLAU
DB03046	7-Methoxy-8-[1-(Methylsulfonyl)-1h-Pyrazol-4-Yl]Naphthalene-2-Carboximidamide	experimental	*PLAU*	PLAU
DB03082	6-[(Z)-Amino(Imino)Methyl]-N-[4-(Aminomethyl)Phenyl]-4-(Pyrimidin-2-Ylamino)-2-Naphthamide	experimental	*PLAU*	PLAU
DB03127	Benzamidine	experimental	*KLK6, PRSS2, PRSS3, ATOX1, ECI1, sprT, ST14, PLAU, PRSS1, CSNK2A1, KLK1*	PLAU

DB03136	4-Iodobenzo[B]Thiophene-2-Carboxamidine	experimental	*F2, PRSS1, PLAU*	PLAU
DB03159	CRA_8696	experimental	*F2, PRSS1, PLAU*	PLAU
DB03476	Trans-6-(2-Phenylcyclopropyl)-Naphthalene-2-Carboxamidine	experimental	*PLAU*	PLAU
DB03729	2-Amino-1H-benzimidazol-5-ol	experimental	*PLAU*	PLAU
DB03782	N-(1-adamantyl)-N-(4-guanidinobenzyl)urea	experimental	*PLAU*	PLAU
DB03865	6-Chloro-2-(2-Hydroxy-Biphenyl-3-Yl)-1h-Indole-5-Carboxamidine	experimental	*F2, PRSS1, PLAU, HPN*	PLAU
DB03876	Thieno[2,3-B]Pyridine-2-Carboxamidine	experimental	*PRSS1, PLAU*	PLAU
DB04059	8-(Pyrimidin-2-Ylamino)Naphthalene-2-Carboximidamide	experimental	*PLAU*	PLAU
DB04172	[2,4,6-Triisopropyl-Phenylsulfonyl-L-[3-Amidino-Phenylalanine]]-Piperazine-N-Beta-Alanine	experimental	*PLAU*	PLAU
DB05254	Fibrinolysin	investigational	*SERPINE1, PLAU*	PLAU
DB06855	6-fluoro-2-(2-hydroxy-3-isobutoxy-phenyl)-1H-benzoimidazole-5-carboxamidine	experimental	*PLAU, PRSS1*	PLAU
DB06856	6-FLUORO-2-[2-HYDROXY-3-(2-METHYL-CYCLOHEXYLOXY)-PHENYL]-1H-INDOLE-5-CARBOXAMIDINE	experimental	*PLAU*	PLAU
DB06857	N-(4-CARBAMIMIDOYL-3-CHORO-PHENYL)-2-HYDROXY-3-IODO-5-METHYL-BENZAMIDE	experimental	*PLAU*	PLAU
DB07076	6-[(Z)-AMINO(IMINO)METHYL]-N-[3-(CYCLOPENTYLOXY)PHENYL]-2-NAPHTHAMIDE	experimental	*PLAU*	PLAU
DB07122	1-[4-(2-oxo-2-phenylethyl)phenyl]guanidine	experimental	*PLAU*	PLAU
DB07129	(2R)-1-(2,6-dimethylphenoxy)propan-2-amine	experimental	*PLAU*	PLAU
DB07625	4-(2-aminoethoxy)-N-(2,5-diethoxyphenyl)-3,5-dimethylbenzamide	experimental	*PLAU*	PLAU
DB07626	4-(2-aminoethoxy)-N-(3-chloro-2-ethoxy-5-piperidin-1-ylphenyl)-3,5-dimethylbenzamide	experimental	*PLAU*	PLAU
DB08072	4-(2-AMINOETHOXY)-3,5-DICHLORO-N-[3-(1-METHYLETHOXY)PHENYL]BENZAMIDE	experimental	*PLAU*	PLAU
DB08697	4-(2-aminoethoxy)-N-(3-chloro-5-piperidin-1-ylphenyl)-3,5-dimethylbenzamide	experimental	*PLAU*	PLAU

DB00009	Alteplase	approved	*PLG, FGA, PLAUR, SERPINE1*	PLG
DB00013	Urokinase	approved, investigational, withdrawn	*PLG, PLAUR, SERPINE1, SERPINB2, SERPINA5, LRP2, ST14, NID1*	PLG
DB00015	Reteplase	approved, investigational	*PLG, FGA, SERPINE1*	PLG
DB00029	Anistreplase	approved	*PLG, FGA, SERPINE1*	PLG
DB00031	Tenecteplase	approved	*PLG, FGA, PLAUR, SERPINE1, SERPINB2, CLEC3B, KRT8, ANXA2, CALR, CANX, LRP1*	PLG
DB00086	Streptokinase	approved, investigational	*PLG, F2R*	PLG
DB00302	Tranexamic acid	approved	*PLG*	PLG
DB00513	Aminocaproic acid	approved, investigational	*PLG, PLAT, LPA*	PLG
DB03709	Bicine	experimental	*glf, PLG, mltB*	PLG
DB04925	Desmoteplase	investigational	*PLG*	PLG
DB06692	Aprotinin	approved, investigational, withdrawn	*PRSS1, CTRB1, PLG, KLK1*	PLG
DB09130	Copper	approved, investigational	*APP, AHCY, HIST1H2BC, GAPDH, NME1, HIST1H1E, PRDX1, S100A8, RPSA, ACTG1, ENO1, EEF1A1, KRT8, P4HB, PDIA3, HSPD1, HSPA13, HSPA5, HSP90B1, TF, Lyar, RPS2, SRSF1, HNRNPA2B1, HNRNPH1, HNRNPH3, cbiD, HNRNPL, SFPQ, SF3A2, RACK1, ACTN1, ACY1, ANXA4, ANXA5, CALR, PKM, AKR1A1, CYB5R3, GSR, TKT, PRDX2, PRDX6, PPIA, HSPA8, HSP90AA1, PTGES3, STIP1, EEF1A1L14, EIF6, EIF4A1, GPI, LDHA, PGK1, TUBA3C, TUBB, CFL1, YWHAB, GOT1, GSS, HDGF, IDH3A, CLIC1, PSME1, PEBP1, PGAM1, RANBP1, UGDH, B2M, SCO1, PRNP, GLRA1, HTT, NEIL1, NEIL2, HEPHL1, SERPINE1, S100A2, S100A4, SNCA, BDNF, PARK7, IAPP, TAC3, A1BG, AFM, AGT, AHSG, APCS, APOA1, APOA2, APOA4, APOBR, APOC2, APOC3, APOD, APOE, APOH, AZGP1, C1QC, C1S, C3, C4B, C4BPA, C5, C8B, C9, CFH, CFI, CLEC3B, CLU, F2, C1QBP, GSN, HBA1, HBB, CBX5, HPR, IGFALS, IGHG1, IGHG4, IGLL1, ITIH2, KNG1, KRT1, KRT10, KRT2, KRT9, LRG1, LUM, PGLYRP2, PLG, PON1, PPBP, SERPINA1, SERPINA4, SERPINA6, SERPINA7, SERPINC1, SERPIND1, SERPINF1, SERPINF2, SERPING1, TTR, VTN, APLP1*	PLG
DB12831	Gabexate	investigational	*KLKB1, F2, PLG*	PLG
DB08888	Ocriplasmin	approved	*FN1, A2M, SERPINF2*	SERPINF2
DB09130	Copper	approved, investigational	*APP, AHCY, HIST1H2BC, GAPDH, NME1, HIST1H1E, PRDX1, S100A8, RPSA, ACTG1, ENO1, EEF1A1, KRT8, P4HB, PDIA3, HSPD1, HSPA13, HSPA5, HSP90B1, TF, Lyar, RPS2, SRSF1, HNRNPA2B1, HNRNPH1, HNRNPH3, cbiD, HNRNPL, SFPQ, SF3A2, RACK1, ACTN1, ACY1, ANXA4, ANXA5, CALR, PKM, AKR1A1, CYB5R3, GSR, TKT, PRDX2, PRDX6, PPIA, HSPA8, HSP90AA1, PTGES3, STIP1, EEF1A1L14, EIF6, EIF4A1, GPI, LDHA, PGK1, TUBA3C, TUBB, CFL1, YWHAB, GOT1, GSS, HDGF, IDH3A, CLIC1, PSME1, PEBP1, PGAM1, RANBP1, UGDH, B2M, SCO1, PRNP, GLRA1, HTT, NEIL1, NEIL2, HEPHL1, SERPINE1, S100A2, S100A4, SNCA, BDNF, PARK7, IAPP, TAC3, A1BG, AFM, AGT, AHSG, APCS, APOA1, APOA2, APOA4, APOBR, APOC2, APOC3, APOD, APOE, APOH, AZGP1, C1QC, C1S, C3, C4B, C4BPA, C5, C8B, C9, CFH, CFI, CLEC3B, CLU, F2, C1QBP, GSN, HBA1, HBB, CBX5, HPR, IGFALS, IGHG1, IGHG4, IGLL1, ITIH2, KNG1, KRT1, KRT10, KRT2, KRT9, LRG1, LUM, PGLYRP2, PLG, PON1, PPBP, SERPINA1, SERPINA4, SERPINA6, SERPINA7, SERPINC1, SERPIND1, SERPINF1, SERPINF2, SERPING1, TTR, VTN, APLP1*	SERPINF2

DB01593	Zinc	approved, investigational	*BDKRB1, MGMT, ALDOA, EEF1A1, ENO1, GAPDHS, NME1, P4HB, PDIA3, PRDX1, PSPH, TPI1, TUFM, ESR1, IL3, MT2A, CCS, HDAC1, HDAC4, MPG, SEMG1, SOD1, HDAC8, SIVA1, GLRA1, MDM2, INS, UTRN, ASPA, S100A8, S100A9, MMP9, TP73, S100A2, TP53, MT3, PDCD6, DAND5, MT1A, A1BG, A2M, AGT, AHSG, APCS, APOA1, APOA2, APOA4, APOBR, APOE, APOL1, C1QB, C1QC, C1R, C1S, C3, C4B, C4BPA, C4BPB, C5, BRCC3, C8A, C8B, C8G, CFB, CFH, CFI, CLU, CP, CPN1, CPN2, DCD, DSP, F12, F13B, F2, FCN3, FGA, FN1, GSN, HBA1, HBB, HPR, HRNR, IGFALS, IGHA1, IGHM, IGKV1-17, IGLV3-21, ITIH1, ITIH2, ITIH3, ITIH4, JCHAIN, JUP, KLKB1, KNG1, KRT1, KRT10, KRT14, KRT16, KRT2, KRT5, KRT6A, KRT9, ORM2, PGLYRP2, PON1, PZP, S100A7, SEPP1, SERPINA1, SERPINA3, SERPINA4, SERPINA6, SERPIND1, SHBG, TF, TTR, VTN, APLP1, APLP2, APP, PARP1*	APOA1
DB09130	Copper	approved, investigational	*APP, AHCY, HIST1H2BC, GAPDH, NME1, HIST1H1E, PRDX1, S100A8, RPSA, ACTG1, ENO1, EEF1A1, KRT8, P4HB, PDIA3, HSPD1, HSPA13, HSPA5, HSP90B1, TF, Lyar, RPS2, SRSF1, HNRNPA2B1, HNRNPH1, HNRNPH3, cbiD, HNRNPL, SFPQ, SF3A2, RACK1, ACTN1, ACY1, ANXA4, ANXA5, CALR, PKM, AKR1A1, CYB5R3, GSR, TKT, PRDX2, PRDX6, PPIA, HSPA8, HSP90AA1, PTGES3, STIP1, EEF1A1L14, EIF6, EIF4A1, GPI, LDHA, PGK1, TUBA3C, TUBB, CFL1, YWHAB, GOT1, GSS, HDGF, IDH3A, CLIC1, PSME1, PEBP1, PGAM1, RANBP1, UGDH, B2M, SCO1, PRNP, GLRA1, HTT, NEIL1, NEIL2, HEPHL1, SERPINE1, S100A2, S100A4, SNCA, BDNF, PARK7, IAPP, TAC3, A1BG, AFM, AGT, AHSG, APCS, APOA1, APOA2, APOA4, APOBR, APOC2, APOC3, APOD, APOE, APOH, AZGP1, C1QC, C1S, C3, C4B, C4BPA, C5, C8B, C9, CFH, CFI, CLEC3B, CLU, F2, C1QBP, GSN, HBA1, HBB, CBX5, HPR, IGFALS, IGHG1, IGHG4, IGLL1, ITIH2, KNG1, KRT1, KRT10, KRT2, KRT9, LRG1, LUM, PGLYRP2, PLG, PON1, PPBP, SERPINA1, SERPINA4, SERPINA6, SERPINA7, SERPINC1, SERPIND1, SERPINF1, SERPINF2, SERPING1, TTR, VTN, APLP1*	APOA1
DB14487	Zinc acetate	approved, investigational	*BDKRB1, MGMT, ALDOA, EEF1A1, ENO1, GAPDHS, NME1, P4HB, PDIA3, PRDX1, PSPH, TPI1, TUFM, ESR1, IL3, MT2A, CCS, HDAC1, HDAC4, MPG, SEMG1, SOD1, HDAC8, SIVA1, GLRA1, MDM2, INS, UTRN, ASPA, S100A8, S100A9, MMP9, TP73, S100A2, TP53, MT3, PDCD6, DAND5, MT1A, A1BG, A2M, AGT, AHSG, APCS, APOA1, APOA2, APOA4, APOBR, APOE, APOL1, C1QB, C1QC, C1R, C1S, C3, C4B, C4BPA, C4BPB, C5, BRCC3, C8A, C8B, C8G, CFB, CFH, CFI, CLU, CP, CPN1, CPN2, DCD, DSP, F12, F13B, F2, FCN3, FGA, FN1, GSN, HBA1, HBB, HPR, HRNR, IGFALS, IGHA1, IGHM, IGKV1-17, IGLV3-21, ITIH1, ITIH2, ITIH3, ITIH4, JCHAIN, JUP, KLKB1, KNG1, KRT1, KRT10, KRT14, KRT16, KRT2, KRT5, KRT6A, KRT9, ORM2, PGLYRP2, PON1, PZP, S100A7, SEPP1, SERPINA1, SERPINA3, SERPINA4, SERPINA6, SERPIND1, SHBG, TF, TTR, VTN, APLP1, APLP2, APP, PARP1*	APOA1
DB14533	Zinc chloride	approved, investigational	*BDKRB1, MGMT, ALDOA, EEF1A1, ENO1, GAPDHS, NME1, P4HB, PDIA3, PRDX1, PSPH, TPI1, TUFM, ESR1, IL3, MT2A, CCS, HDAC1, HDAC4, MPG, SEMG1, SOD1, HDAC8, SIVA1, GLRA1, MDM2, INS, UTRN, ASPA, S100A8, S100A9, MMP9, TP73, S100A2, TP53, MT3, MT1A, A2M, AHSG, APOA1, APOBR, APOE, APOL1, C1QB, C1QC, C1R, C1S, C3, C4B, C4BPA, C4BPB, C5, BRCC3, CFH, CLU, CP, CPN1, CPN2, DCD, FGA, FN1, HBA1, HBB, HP, IGFALS, ITIH1, ITIH2, ITIH3, ITIH4, JCHAIN, KLKB1, KNG1, KRT9, ORM2, PGLYRP2, PON1, PZP, S100A7, SEPP1, SERPINA1, SERPINA3, SERPINA6, SERPIND1, SHBG, TF, TTR, VTN, APLP1, APLP2, APP, PARP1, APOB, HPX, HRG, F11, APOBEC1, SEMG2*	APOA1

DB14548	Zinc sulfate, unspecified form	approved, experimental	*BDKRB1, MGMT, ALDOA, EEF1A1, ENO1, GAPDHS, NME1, P4HB, PDIA3, PRDX1, PSPH, TPI1, TUFM, ESR1, IL3, MT2A, CCS, HDAC1, HDAC4, MPG, SEMG1, SOD1, HDAC8, SIVA1, GLRA1, MDM2, INS, UTRN, ASPA, S100A8, S100A9, MMP9, TP73, S100A2, TP53, MT3, MT1A, A2M, AHSG, APOA1, APOBR, APOE, APOL1, C1QB, C1QC, C1R, C1S, C3, C4B, C4BPA, C4BPB, C5, BRCC3, CFH, CLU, CP, CPN1, CPN2, DCD, FGA, FN1, HBA1, HBB, HP, IGFALS, ITIH1, ITIH2, ITIH3, ITIH4, JCHAIN, KLKB1, KNG1, KRT9, ORM2, PGLYRP2, PON1, PZP, S100A7, SEPP1, SERPINA1, SERPINA3, SERPINA6, SERPIND1, SHBG, TF, TTR, VTN, APLP1, APLP2, APP, PARP1, APOB, HPX, HRG, F11, APOBEC1, SEMG2*	APOA1
DB01593	Zinc	approved, investigational	*BDKRB1, MGMT, ALDOA, EEF1A1, ENO1, GAPDHS, NME1, P4HB, PDIA3, PRDX1, PSPH, TPI1, TUFM, ESR1, IL3, MT2A, CCS, HDAC1, HDAC4, MPG, SEMG1, SOD1, HDAC8, SIVA1, GLRA1, MDM2, INS, UTRN, ASPA, S100A8, S100A9, MMP9, TP73, S100A2, TP53, MT3, PDCD6, DAND5, MT1A, A1BG, A2M, AGT, AHSG, APCS, APOA1, APOA2, APOA4, APOBR, APOE, APOL1, C1QB, C1QC, C1R, C1S, C3, C4B, C4BPA, C4BPB, C5, BRCC3, C8A, C8B, C8G, CFB, CFH, CFI, CLU, CP, CPN1, CPN2, DCD, DSP, F12, F13B, F2, FCN3, FGA, FN1, GSN, HBA1, HBB, HPR, HRNR, IGFALS, IGHA1, IGHM, IGKV1-17, IGLV3-21, ITIH1, ITIH2, ITIH3, ITIH4, JCHAIN, JUP, KLKB1, KNG1, KRT1, KRT10, KRT14, KRT16, KRT2, KRT5, KRT6A, KRT9, ORM2, PGLYRP2, PON1, PZP, S100A7, SEPP1, SERPINA1, SERPINA3, SERPINA4, SERPINA6, SERPIND1, SHBG, TF, TTR, VTN, APLP1, APLP2, APP, PARP1*	APOE
DB09130	Copper	approved, investigational	*APP, AHCY, HIST1H2BC, GAPDH, NME1, HIST1H1E, PRDX1, S100A8, RPSA, ACTG1, ENO1, EEF1A1, KRT8, P4HB, PDIA3, HSPD1, HSPA13, HSPA5, HSP90B1, TF, Lyar, RPS2, SRSF1, HNRNPA2B1, HNRNPH1, HNRNPH3, cbiD, HNRNPL, SFPQ, SF3A2, RACK1, ACTN1, ACY1, ANXA4, ANXA5, CALR, PKM, AKR1A1, CYB5R3, GSR, TKT, PRDX2, PRDX6, PPIA, HSPA8, HSP90AA1, PTGES3, STIP1, EEF1A1L14, EIF6, EIF4A1, GPI, LDHA, PGK1, TUBA3C, TUBB, CFL1, YWHAB, GOT1, GSS, HDGF, IDH3A, CLIC1, PSME1, PEBP1, PGAM1, RANBP1, UGDH, B2M, SCO1, PRNP, GLRA1, HTT, NEIL1, NEIL2, HEPHL1, SERPINE1, S100A2, S100A4, SNCA, BDNF, PARK7, IAPP, TAC3, A1BG, AFM, AGT, AHSG, APCS, APOA1, APOA2, APOA4, APOBR, APOC2, APOC3, APOD, APOE, APOH, AZGP1, C1QC, C1S, C3, C4B, C4BPA, C5, C8B, C9, CFH, CFI, CLEC3B, CLU, F2, C1QBP, GSN, HBA1, HBB, CBX5, HPR, IGFALS, IGHG1, IGHG4, IGLL1, ITIH2, KNG1, KRT1, KRT10, KRT2, KRT9, LRG1, LUM, PGLYRP2, PLG, PON1, PPBP, SERPINA1, SERPINA4, SERPINA6, SERPINA7, SERPINC1, SERPIND1, SERPINF1, SERPINF2, SERPING1, TTR, VTN, APLP1*	APOE
DB14487	Zinc acetate	approved, investigational	*BDKRB1, MGMT, ALDOA, EEF1A1, ENO1, GAPDHS, NME1, P4HB, PDIA3, PRDX1, PSPH, TPI1, TUFM, ESR1, IL3, MT2A, CCS, HDAC1, HDAC4, MPG, SEMG1, SOD1, HDAC8, SIVA1, GLRA1, MDM2, INS, UTRN, ASPA, S100A8, S100A9, MMP9, TP73, S100A2, TP53, MT3, PDCD6, DAND5, MT1A, A1BG, A2M, AGT, AHSG, APCS, APOA1, APOA2, APOA4, APOBR, APOE, APOL1, C1QB, C1QC, C1R, C1S, C3, C4B, C4BPA, C4BPB, C5, BRCC3, C8A, C8B, C8G, CFB, CFH, CFI, CLU, CP, CPN1, CPN2, DCD, DSP, F12, F13B, F2, FCN3, FGA, FN1, GSN, HBA1, HBB, HPR, HRNR, IGFALS, IGHA1, IGHM, IGKV1-17, IGLV3-21, ITIH1, ITIH2, ITIH3, ITIH4, JCHAIN, JUP, KLKB1, KNG1, KRT1, KRT10, KRT14, KRT16, KRT2, KRT5, KRT6A, KRT9, ORM2, PGLYRP2, PON1, PZP, S100A7, SEPP1, SERPINA1, SERPINA3, SERPINA4, SERPINA6, SERPIND1, SHBG, TF, TTR, VTN, APLP1, APLP2, APP, PARP1*	APOE

DB14533	Zinc chloride	approved, investigational	*BDKRB1, MGMT, ALDOA, EEF1A1, ENO1, GAPDHS, NME1, P4HB, PDIA3, PRDX1, PSPH, TPI1, TUFM, ESR1, IL3, MT2A, CCS, HDAC1, HDAC4, MPG, SEMG1, SOD1, HDAC8, SIVA1, GLRA1, MDM2, INS, UTRN, ASPA, S100A8, S100A9, MMP9, TP73, S100A2, TP53, MT3, MT1A, A2M, AHSG, APOA1, APOBR, APOE, APOL1, C1QB, C1QC, C1R, C1S, C3, C4B, C4BPA, C4BPB, C5, BRCC3, CFH, CLU, CP, CPN1, CPN2, DCD, FGA, FN1, HBA1, HBB, HP, IGFALS, ITIH1, ITIH2, ITIH3, ITIH4, JCHAIN, KLKB1, KNG1, KRT9, ORM2, PGLYRP2, PON1, PZP, S100A7, SEPP1, SERPINA1, SERPINA3, SERPINA6, SERPIND1, SHBG, TF, TTR, VTN, APLP1, APLP2, APP, PARP1, APOB, HPX, HRG, F11, APOBEC1, SEMG2*	APOE
DB14548	Zinc sulfate, unspecified form	approved, experimental	*BDKRB1, MGMT, ALDOA, EEF1A1, ENO1, GAPDHS, NME1, P4HB, PDIA3, PRDX1, PSPH, TPI1, TUFM, ESR1, IL3, MT2A, CCS, HDAC1, HDAC4, MPG, SEMG1, SOD1, HDAC8, SIVA1, GLRA1, MDM2, INS, UTRN, ASPA, S100A8, S100A9, MMP9, TP73, S100A2, TP53, MT3, MT1A, A2M, AHSG, APOA1, APOBR, APOE, APOL1, C1QB, C1QC, C1R, C1S, C3, C4B, C4BPA, C4BPB, C5, BRCC3, CFH, CLU, CP, CPN1, CPN2, DCD, FGA, FN1, HBA1, HBB, HP, IGFALS, ITIH1, ITIH2, ITIH3, ITIH4, JCHAIN, KLKB1, KNG1, KRT9, ORM2, PGLYRP2, PON1, PZP, S100A7, SEPP1, SERPINA1, SERPINA3, SERPINA6, SERPIND1, SHBG, TF, TTR, VTN, APLP1, APLP2, APP, PARP1, APOB, HPX, HRG, F11, APOBEC1, SEMG2*	APOE
DB00746	Deferoxamine	approved, investigational	*APP*	APP
DB01370	Aluminium	approved, investigational	*TF, ATP1A1, KLK1, APP*	APP
DB01593	Zinc	approved, investigational	*BDKRB1, MGMT, ALDOA, EEF1A1, ENO1, GAPDHS, NME1, P4HB, PDIA3, PRDX1, PSPH, TPI1, TUFM, ESR1, IL3, MT2A, CCS, HDAC1, HDAC4, MPG, SEMG1, SOD1, HDAC8, SIVA1, GLRA1, MDM2, INS, UTRN, ASPA, S100A8, S100A9, MMP9, TP73, S100A2, TP53, MT3, PDCD6, DAND5, MT1A, A1BG, A2M, AGT, AHSG, APCS, APOA1, APOA2, APOA4, APOBR, APOE, APOL1, C1QB, C1QC, C1R, C1S, C3, C4B, C4BPA, C4BPB, C5, BRCC3, C8A, C8B, C8G, CFB, CFH, CFI, CLU, CP, CPN1, CPN2, DCD, DSP, F12, F13B, F2, FCN3, FGA, FN1, GSN, HBA1, HBB, HPR, HRNR, IGFALS, IGHA1, IGHM, IGKV1-17, IGLV3-21, ITIH1, ITIH2, ITIH3, ITIH4, JCHAIN, JUP, KLKB1, KNG1, KRT1, KRT10, KRT14, KRT16, KRT2, KRT5, KRT6A, KRT9, ORM2, PGLYRP2, PON1, PZP, S100A7, SEPP1, SERPINA1, SERPINA3, SERPINA4, SERPINA6, SERPIND1, SHBG, TF, TTR, VTN, APLP1, APLP2, APP, PARP1*	APP
DB02235	L-methionine (R)-S-oxide	experimental	*PROF, APP, katA*	APP
DB02709	Resveratrol	investigational	*NQO2, CSNK2A1, PTGS1, PTGS2, ALOX15, ALOX5, AHR, PI4K2B, ITGA5, ITGB3, APP, SNCA, SIRT1, ESR1, MTNR1A, MTNR1B, CLEC14A, NR1I2, NR1I3, SLC2A1, CBR1, PPARA, PPARG, AKT1, KHSRP, YARS*	APP
DB03754	Tromethamine	approved	*APP*	APP
DB04892	Phenserine	investigational	*ACHE, BCHE, APP*	APP
DB05088	Tetrathiomolybdate	investigational	*APP*	APP
DB05150	CAD106	investigational	*APP*	APP
DB05846	Mito-4509	investigational	*APP*	APP
DB05938	Edonerpic	investigational	*APP*	APP
DB06782	Dimercaprol	approved	*APP*	APP

DB09130	Copper	approved, investigational	*APP, AHCY, HIST1H2BC, GAPDH, NME1, HIST1H1E, PRDX1, S100A8, RPSA, ACTG1, ENO1, EEF1A1, KRT8, P4HB, PDIA3, HSPD1, HSPA13, HSPA5, HSP90B1, TF, Lyar, RPS2, SRSF1, HNRNPA2B1, HNRNPH1, HNRNPH3, cbiD, HNRNPL, SFPQ, SF3A2, RACK1, ACTN1, ACY1, ANXA4, ANXA5, CALR, PKM, AKR1A1, CYB5R3, GSR, TKT, PRDX2, PRDX6, PPIA, HSPA8, HSP90AA1, PTGES3, STIP1, EEF1A1L14, EIF6, EIF4A1, GPI, LDHA, PGK1, TUBA3C, TUBB, CFL1, YWHAB, GOT1, GSS, HDGF, IDH3A, CLIC1, PSME1, PEBP1, PGAM1, RANBP1, UGDH, B2M, SCO1, PRNP, GLRA1, HTT, NEIL1, NEIL2, HEPHL1, SERPINE1, S100A2, S100A4, SNCA, BDNF, PARK7, IAPP, TAC3, A1BG, AFM, AGT, AHSG, APCS, APOA1, APOA2, APOA4, APOBR, APOC2, APOC3, APOD, APOE, APOH, AZGP1, C1QC, C1S, C3, C4B, C4BPA, C5, C8B, C9, CFH, CFI, CLEC3B, CLU, F2, C1QBP, GSN, HBA1, HBB, CBX5, HPR, IGFALS, IGHG1, IGHG4, IGLL1, ITIH2, KNG1, KRT1, KRT10, KRT2, KRT9, LRG1, LUM, PGLYRP2, PLG, PON1, PPBP, SERPINA1, SERPINA4, SERPINA6, SERPINA7, SERPINC1, SERPIND1, SERPINF1, SERPINF2, SERPING1, TTR, VTN, APLP1*	APP
DB09148	Florbetaben (18F)	approved	*APP*	APP
DB09149	Florbetapir (18F)	approved, investigational	*APP*	APP
DB09151	Flutemetamol (18F)	approved, investigational	*APP*	APP
DB12274	Aducanumab	approved, investigational	*APP*	APP
DB14487	Zinc acetate	approved, investigational	*BDKRB1, MGMT, ALDOA, EEF1A1, ENO1, GAPDHS, NME1, P4HB, PDIA3, PRDX1, PSPH, TPI1, TUFM, ESR1, IL3, MT2A, CCS, HDAC1, HDAC4, MPG, SEMG1, SOD1, HDAC8, SIVA1, GLRA1, MDM2, INS, UTRN, ASPA, S100A8, S100A9, MMP9, TP73, S100A2, TP53, MT3, PDCD6, DAND5, MT1A, A1BG, A2M, AGT, AHSG, APCS, APOA1, APOA2, APOA4, APOBR, APOE, APOL1, C1QB, C1QC, C1R, C1S, C3, C4B, C4BPA, C4BPB, C5, BRCC3, C8A, C8B, C8G, CFB, CFH, CFI, CLU, CP, CPN1, CPN2, DCD, DSP, F12, F13B, F2, FCN3, FGA, FN1, GSN, HBA1, HBB, HPR, HRNR, IGFALS, IGHA1, IGHM, IGKV1-17, IGLV3-21, ITIH1, ITIH2, ITIH3, ITIH4, JCHAIN, JUP, KLKB1, KNG1, KRT1, KRT10, KRT14, KRT16, KRT2, KRT5, KRT6A, KRT9, ORM2, PGLYRP2, PON1, PZP, S100A7, SEPP1, SERPINA1, SERPINA3, SERPINA4, SERPINA6, SERPIND1, SHBG, TF, TTR, VTN, APLP1, APLP2, APP, PARP1*	APP
DB14517	Aluminium phosphate	approved, investigational	*TF, ATP1A1, KLK1, APP*	APP
DB14518	Aluminum acetate	approved, investigational	*TF, ATP1A1, KLK1, APP*	APP
DB14533	Zinc chloride	approved, investigational	*BDKRB1, MGMT, ALDOA, EEF1A1, ENO1, GAPDHS, NME1, P4HB, PDIA3, PRDX1, PSPH, TPI1, TUFM, ESR1, IL3, MT2A, CCS, HDAC1, HDAC4, MPG, SEMG1, SOD1, HDAC8, SIVA1, GLRA1, MDM2, INS, UTRN, ASPA, S100A8, S100A9, MMP9, TP73, S100A2, TP53, MT3, MT1A, A2M, AHSG, APOA1, APOBR, APOE, APOL1, C1QB, C1QC, C1R, C1S, C3, C4B, C4BPA, C4BPB, C5, BRCC3, CFH, CLU, CP, CPN1, CPN2, DCD, FGA, FN1, HBA1, HBB, HP, IGFALS, ITIH1, ITIH2, ITIH3, ITIH4, JCHAIN, KLKB1, KNG1, KRT9, ORM2, PGLYRP2, PON1, PZP, S100A7, SEPP1, SERPINA1, SERPINA3, SERPINA6, SERPIND1, SHBG, TF, TTR, VTN, APLP1, APLP2, APP, PARP1, APOB, HPX, HRG, F11, APOBEC1, SEMG2*	APP
DB14548	Zinc sulfate, unspecified form	approved, experimental	*BDKRB1, MGMT, ALDOA, EEF1A1, ENO1, GAPDHS, NME1, P4HB, PDIA3, PRDX1, PSPH, TPI1, TUFM, ESR1, IL3, MT2A, CCS, HDAC1, HDAC4, MPG, SEMG1, SOD1, HDAC8, SIVA1, GLRA1, MDM2, INS, UTRN, ASPA, S100A8, S100A9, MMP9, TP73, S100A2, TP53, MT3, MT1A, A2M, AHSG, APOA1, APOBR, APOE, APOL1, C1QB, C1QC, C1R, C1S, C3, C4B, C4BPA, C4BPB, C5, BRCC3, CFH, CLU, CP, CPN1, CPN2, DCD, FGA, FN1, HBA1, HBB, HP, IGFALS, ITIH1, ITIH2, ITIH3, ITIH4, JCHAIN, KLKB1, KNG1, KRT9, ORM2, PGLYRP2, PON1, PZP, S100A7, SEPP1, SERPINA1, SERPINA3, SERPINA6, SERPIND1, SHBG, TF, TTR, VTN, APLP1, APLP2, APP, PARP1, APOB, HPX, HRG, F11, APOBEC1, SEMG2*	APP
DB06281	Torcetrapib	investigational	*CETP*	CETP

DB01593	Zinc	approved, investigational	*BDKRB1, MGMT, ALDOA, EEF1A1, ENO1, GAPDHS, NME1, P4HB, PDIA3, PRDX1, PSPH, TPI1, TUFM, ESR1, IL3, MT2A, CCS, HDAC1, HDAC4, MPG, SEMG1, SOD1, HDAC8, SIVA1, GLRA1, MDM2, INS, UTRN, ASPA, S100A8, S100A9, MMP9, TP73, S100A2, TP53, MT3, PDCD6, DAND5, MT1A, A1BG, A2M, AGT, AHSG, APCS, APOA1, APOA2, APOA4, APOBR, APOE, APOL1, C1QB, C1QC, C1R, C1S, C3, C4B, C4BPA, C4BPB, C5, BRCC3, C8A, C8B, C8G, CFB, CFH, CFI, CLU, CP, CPN1, CPN2, DCD, DSP, F12, F13B, F2, FCN3, FGA, FN1, GSN, HBA1, HBB, HPR, HRNR, IGFALS, IGHA1, IGHM, IGKV1-17, IGLV3-21, ITIH1, ITIH2, ITIH3, ITIH4, JCHAIN, JUP, KLKB1, KNG1, KRT1, KRT10, KRT14, KRT16, KRT2, KRT5, KRT6A, KRT9, ORM2, PGLYRP2, PON1, PZP, S100A7, SEPP1, SERPINA1, SERPINA3, SERPINA4, SERPINA6, SERPIND1, SHBG, TF, TTR, VTN, APLP1, APLP2, APP, PARP1*	CLU
DB09130	Copper	approved, investigational	*APP, AHCY, HIST1H2BC, GAPDH, NME1, HIST1H1E, PRDX1, S100A8, RPSA, ACTG1, ENO1, EEF1A1, KRT8, P4HB, PDIA3, HSPD1, HSPA13, HSPA5, HSP90B1, TF, Lyar, RPS2, SRSF1, HNRNPA2B1, HNRNPH1, HNRNPH3, cbiD, HNRNPL, SFPQ, SF3A2, RACK1, ACTN1, ACY1, ANXA4, ANXA5, CALR, PKM, AKR1A1, CYB5R3, GSR, TKT, PRDX2, PRDX6, PPIA, HSPA8, HSP90AA1, PTGES3, STIP1, EEF1A1L14, EIF6, EIF4A1, GPI, LDHA, PGK1, TUBA3C, TUBB, CFL1, YWHAB, GOT1, GSS, HDGF, IDH3A, CLIC1, PSME1, PEBP1, PGAM1, RANBP1, UGDH, B2M, SCO1, PRNP, GLRA1, HTT, NEIL1, NEIL2, HEPHL1, SERPINE1, S100A2, S100A4, SNCA, BDNF, PARK7, IAPP, TAC3, A1BG, AFM, AGT, AHSG, APCS, APOA1, APOA2, APOA4, APOBR, APOC2, APOC3, APOD, APOE, APOH, AZGP1, C1QC, C1S, C3, C4B, C4BPA, C5, C8B, C9, CFH, CFI, CLEC3B, CLU, F2, C1QBP, GSN, HBA1, HBB, CBX5, HPR, IGFALS, IGHG1, IGHG4, IGLL1, ITIH2, KNG1, KRT1, KRT10, KRT2, KRT9, LRG1, LUM, PGLYRP2, PLG, PON1, PPBP, SERPINA1, SERPINA4, SERPINA6, SERPINA7, SERPINC1, SERPIND1, SERPINF1, SERPINF2, SERPING1, TTR, VTN, APLP1*	CLU
DB14487	Zinc acetate	approved, investigational	*BDKRB1, MGMT, ALDOA, EEF1A1, ENO1, GAPDHS, NME1, P4HB, PDIA3, PRDX1, PSPH, TPI1, TUFM, ESR1, IL3, MT2A, CCS, HDAC1, HDAC4, MPG, SEMG1, SOD1, HDAC8, SIVA1, GLRA1, MDM2, INS, UTRN, ASPA, S100A8, S100A9, MMP9, TP73, S100A2, TP53, MT3, PDCD6, DAND5, MT1A, A1BG, A2M, AGT, AHSG, APCS, APOA1, APOA2, APOA4, APOBR, APOE, APOL1, C1QB, C1QC, C1R, C1S, C3, C4B, C4BPA, C4BPB, C5, BRCC3, C8A, C8B, C8G, CFB, CFH, CFI, CLU, CP, CPN1, CPN2, DCD, DSP, F12, F13B, F2, FCN3, FGA, FN1, GSN, HBA1, HBB, HPR, HRNR, IGFALS, IGHA1, IGHM, IGKV1-17, IGLV3-21, ITIH1, ITIH2, ITIH3, ITIH4, JCHAIN, JUP, KLKB1, KNG1, KRT1, KRT10, KRT14, KRT16, KRT2, KRT5, KRT6A, KRT9, ORM2, PGLYRP2, PON1, PZP, S100A7, SEPP1, SERPINA1, SERPINA3, SERPINA4, SERPINA6, SERPIND1, SHBG, TF, TTR, VTN, APLP1, APLP2, APP, PARP1*	CLU
DB14533	Zinc chloride	approved, investigational	*BDKRB1, MGMT, ALDOA, EEF1A1, ENO1, GAPDHS, NME1, P4HB, PDIA3, PRDX1, PSPH, TPI1, TUFM, ESR1, IL3, MT2A, CCS, HDAC1, HDAC4, MPG, SEMG1, SOD1, HDAC8, SIVA1, GLRA1, MDM2, INS, UTRN, ASPA, S100A8, S100A9, MMP9, TP73, S100A2, TP53, MT3, MT1A, A2M, AHSG, APOA1, APOBR, APOE, APOL1, C1QB, C1QC, C1R, C1S, C3, C4B, C4BPA, C4BPB, C5, BRCC3, CFH, CLU, CP, CPN1, CPN2, DCD, FGA, FN1, HBA1, HBB, HP, IGFALS, ITIH1, ITIH2, ITIH3, ITIH4, JCHAIN, KLKB1, KNG1, KRT9, ORM2, PGLYRP2, PON1, PZP, S100A7, SEPP1, SERPINA1, SERPINA3, SERPINA6, SERPIND1, SHBG, TF, TTR, VTN, APLP1, APLP2, APP, PARP1, APOB, HPX, HRG, F11, APOBEC1, SEMG2*	CLU

DB14548	Zinc sulfate, unspecified form	approved, experimental	*BDKRB1, MGMT, ALDOA, EEF1A1, ENO1, GAPDHS, NME1, P4HB, PDIA3, PRDX1, PSPH, TPI1, TUFM, ESR1, IL3, MT2A, CCS, HDAC1, HDAC4, MPG, SEMG1, SOD1, HDAC8, SIVA1, GLRA1, MDM2, INS, UTRN, ASPA, S100A8, S100A9, MMP9, TP73, S100A2, TP53, MT3, MT1A, A2M, AHSG, APOA1, APOBR, APOE, APOL1, C1QB, C1QC, C1R, C1S, C3, C4B, C4BPA, C4BPB, C5, BRCC3, CFH, CLU, CP, CPN1, CPN2, DCD, FGA, FN1, HBA1, HBB, HP, IGFALS, ITIH1, ITIH2, ITIH3, ITIH4, JCHAIN, KLKB1, KNG1, KRT9, ORM2, PGLYRP2, PON1, PZP, S100A7, SEPP1, SERPINA1, SERPINA3, SERPINA6, SERPIND1, SHBG, TF, TTR, VTN, APLP1, APLP2, APP, PARP1, APOB, HPX, HRG, F11, APOBEC1, SEMG2*	CLU
DB01593	Zinc	approved, investigational	*BDKRB1, MGMT, ALDOA, EEF1A1, ENO1, GAPDHS, NME1, P4HB, PDIA3, PRDX1, PSPH, TPI1, TUFM, ESR1, IL3, MT2A, CCS, HDAC1, HDAC4, MPG, SEMG1, SOD1, HDAC8, SIVA1, GLRA1, MDM2, INS, UTRN, ASPA, S100A8, S100A9, MMP9, TP73, S100A2, TP53, MT3, PDCD6, DAND5, MT1A, A1BG, A2M, AGT, AHSG, APCS, APOA1, APOA2, APOA4, APOBR, APOE, APOL1, C1QB, C1QC, C1R, C1S, C3, C4B, C4BPA, C4BPB, C5, BRCC3, C8A, C8B, C8G, CFB, CFH, CFI, CLU, CP, CPN1, CPN2, DCD, DSP, F12, F13B, F2, FCN3, FGA, FN1, GSN, HBA1, HBB, HPR, HRNR, IGFALS, IGHA1, IGHM, IGKV1-17, IGLV3-21, ITIH1, ITIH2, ITIH3, ITIH4, JCHAIN, JUP, KLKB1, KNG1, KRT1, KRT10, KRT14, KRT16, KRT2, KRT5, KRT6A, KRT9, ORM2, PGLYRP2, PON1, PZP, S100A7, SEPP1, SERPINA1, SERPINA3, SERPINA4, SERPINA6, SERPIND1, SHBG, TF, TTR, VTN, APLP1, APLP2, APP, PARP1*	HPR
DB09130	Copper	approved, investigational	*APP, AHCY, HIST1H2BC, GAPDH, NME1, HIST1H1E, PRDX1, S100A8, RPSA, ACTG1, ENO1, EEF1A1, KRT8, P4HB, PDIA3, HSPD1, HSPA13, HSPA5, HSP90B1, TF, Lyar, RPS2, SRSF1, HNRNPA2B1, HNRNPH1, HNRNPH3, cbiD, HNRNPL, SFPQ, SF3A2, RACK1, ACTN1, ACY1, ANXA4, ANXA5, CALR, PKM, AKR1A1, CYB5R3, GSR, TKT, PRDX2, PRDX6, PPIA, HSPA8, HSP90AA1, PTGES3, STIP1, EEF1A1L14, EIF6, EIF4A1, GPI, LDHA, PGK1, TUBA3C, TUBB, CFL1, YWHAB, GOT1, GSS, HDGF, IDH3A, CLIC1, PSME1, PEBP1, PGAM1, RANBP1, UGDH, B2M, SCO1, PRNP, GLRA1, HTT, NEIL1, NEIL2, HEPHL1, SERPINE1, S100A2, S100A4, SNCA, BDNF, PARK7, IAPP, TAC3, A1BG, AFM, AGT, AHSG, APCS, APOA1, APOA2, APOA4, APOBR, APOC2, APOC3, APOD, APOE, APOH, AZGP1, C1QC, C1S, C3, C4B, C4BPA, C5, C8B, C9, CFH, CFI, CLEC3B, CLU, F2, C1QBP, GSN, HBA1, HBB, CBX5, HPR, IGFALS, IGHG1, IGHG4, IGLL1, ITIH2, KNG1, KRT1, KRT10, KRT2, KRT9, LRG1, LUM, PGLYRP2, PLG, PON1, PPBP, SERPINA1, SERPINA4, SERPINA6, SERPINA7, SERPINC1, SERPIND1, SERPINF1, SERPINF2, SERPING1, TTR, VTN, APLP1*	HPR
DB14487	Zinc acetate	approved, investigational	*BDKRB1, MGMT, ALDOA, EEF1A1, ENO1, GAPDHS, NME1, P4HB, PDIA3, PRDX1, PSPH, TPI1, TUFM, ESR1, IL3, MT2A, CCS, HDAC1, HDAC4, MPG, SEMG1, SOD1, HDAC8, SIVA1, GLRA1, MDM2, INS, UTRN, ASPA, S100A8, S100A9, MMP9, TP73, S100A2, TP53, MT3, PDCD6, DAND5, MT1A, A1BG, A2M, AGT, AHSG, APCS, APOA1, APOA2, APOA4, APOBR, APOE, APOL1, C1QB, C1QC, C1R, C1S, C3, C4B, C4BPA, C4BPB, C5, BRCC3, C8A, C8B, C8G, CFB, CFH, CFI, CLU, CP, CPN1, CPN2, DCD, DSP, F12, F13B, F2, FCN3, FGA, FN1, GSN, HBA1, HBB, HPR, HRNR, IGFALS, IGHA1, IGHM, IGKV1-17, IGLV3-21, ITIH1, ITIH2, ITIH3, ITIH4, JCHAIN, JUP, KLKB1, KNG1, KRT1, KRT10, KRT14, KRT16, KRT2, KRT5, KRT6A, KRT9, ORM2, PGLYRP2, PON1, PZP, S100A7, SEPP1, SERPINA1, SERPINA3, SERPINA4, SERPINA6, SERPIND1, SHBG, TF, TTR, VTN, APLP1, APLP2, APP, PARP1*	HPR
DB01327	Cefazolin	approved	*mrcA, mrcB, pbpC, mrdA, ftsI, PON1, IL15, IL2*	PON1

DB01593	Zinc	approved, investigational	*BDKRB1, MGMT, ALDOA, EEF1A1, ENO1, GAPDHS, NME1, P4HB, PDIA3, PRDX1, PSPH, TPI1, TUFM, ESR1, IL3, MT2A, CCS, HDAC1, HDAC4, MPG, SEMG1, SOD1, HDAC8, SIVA1, GLRA1, MDM2, INS, UTRN, ASPA, S100A8, S100A9, MMP9, TP73, S100A2, TP53, MT3, PDCD6, DAND5, MT1A, A1BG, A2M, AGT, AHSG, APCS, APOA1, APOA2, APOA4, APOBR, APOE, APOL1, C1QB, C1QC, C1R, C1S, C3, C4B, C4BPA, C4BPB, C5, BRCC3, C8A, C8B, C8G, CFB, CFH, CFI, CLU, CP, CPN1, CPN2, DCD, DSP, F12, F13B, F2, FCN3, FGA, FN1, GSN, HBA1, HBB, HPR, HRNR, IGFALS, IGHA1, IGHM, IGKV1-17, IGLV3-21, ITIH1, ITIH2, ITIH3, ITIH4, JCHAIN, JUP, KLKB1, KNG1, KRT1, KRT10, KRT14, KRT16, KRT2, KRT5, KRT6A, KRT9, ORM2, PGLYRP2, PON1, PZP, S100A7, SEPP1, SERPINA1, SERPINA3, SERPINA4, SERPINA6, SERPIND1, SHBG, TF, TTR, VTN, APLP1, APLP2, APP, PARP1*	PON1
DB09130	Copper	approved, investigational	*APP, AHCY, HIST1H2BC, GAPDH, NME1, HIST1H1E, PRDX1, S100A8, RPSA, ACTG1, ENO1, EEF1A1, KRT8, P4HB, PDIA3, HSPD1, HSPA13, HSPA5, HSP90B1, TF, Lyar, RPS2, SRSF1, HNRNPA2B1, HNRNPH1, HNRNPH3, cbiD, HNRNPL, SFPQ, SF3A2, RACK1, ACTN1, ACY1, ANXA4, ANXA5, CALR, PKM, AKR1A1, CYB5R3, GSR, TKT, PRDX2, PRDX6, PPIA, HSPA8, HSP90AA1, PTGES3, STIP1, EEF1A1L14, EIF6, EIF4A1, GPI, LDHA, PGK1, TUBA3C, TUBB, CFL1, YWHAB, GOT1, GSS, HDGF, IDH3A, CLIC1, PSME1, PEBP1, PGAM1, RANBP1, UGDH, B2M, SCO1, PRNP, GLRA1, HTT, NEIL1, NEIL2, HEPHL1, SERPINE1, S100A2, S100A4, SNCA, BDNF, PARK7, IAPP, TAC3, A1BG, AFM, AGT, AHSG, APCS, APOA1, APOA2, APOA4, APOBR, APOC2, APOC3, APOD, APOE, APOH, AZGP1, C1QC, C1S, C3, C4B, C4BPA, C5, C8B, C9, CFH, CFI, CLEC3B, CLU, F2, C1QBP, GSN, HBA1, HBB, CBX5, HPR, IGFALS, IGHG1, IGHG4, IGLL1, ITIH2, KNG1, KRT1, KRT10, KRT2, KRT9, LRG1, LUM, PGLYRP2, PLG, PON1, PPBP, SERPINA1, SERPINA4, SERPINA6, SERPINA7, SERPINC1, SERPIND1, SERPINF1, SERPINF2, SERPING1, TTR, VTN, APLP1*	PON1
DB14487	Zinc acetate	approved, investigational	*BDKRB1, MGMT, ALDOA, EEF1A1, ENO1, GAPDHS, NME1, P4HB, PDIA3, PRDX1, PSPH, TPI1, TUFM, ESR1, IL3, MT2A, CCS, HDAC1, HDAC4, MPG, SEMG1, SOD1, HDAC8, SIVA1, GLRA1, MDM2, INS, UTRN, ASPA, S100A8, S100A9, MMP9, TP73, S100A2, TP53, MT3, PDCD6, DAND5, MT1A, A1BG, A2M, AGT, AHSG, APCS, APOA1, APOA2, APOA4, APOBR, APOE, APOL1, C1QB, C1QC, C1R, C1S, C3, C4B, C4BPA, C4BPB, C5, BRCC3, C8A, C8B, C8G, CFB, CFH, CFI, CLU, CP, CPN1, CPN2, DCD, DSP, F12, F13B, F2, FCN3, FGA, FN1, GSN, HBA1, HBB, HPR, HRNR, IGFALS, IGHA1, IGHM, IGKV1-17, IGLV3-21, ITIH1, ITIH2, ITIH3, ITIH4, JCHAIN, JUP, KLKB1, KNG1, KRT1, KRT10, KRT14, KRT16, KRT2, KRT5, KRT6A, KRT9, ORM2, PGLYRP2, PON1, PZP, S100A7, SEPP1, SERPINA1, SERPINA3, SERPINA4, SERPINA6, SERPIND1, SHBG, TF, TTR, VTN, APLP1, APLP2, APP, PARP1*	PON1
DB14533	Zinc chloride	approved, investigational	*BDKRB1, MGMT, ALDOA, EEF1A1, ENO1, GAPDHS, NME1, P4HB, PDIA3, PRDX1, PSPH, TPI1, TUFM, ESR1, IL3, MT2A, CCS, HDAC1, HDAC4, MPG, SEMG1, SOD1, HDAC8, SIVA1, GLRA1, MDM2, INS, UTRN, ASPA, S100A8, S100A9, MMP9, TP73, S100A2, TP53, MT3, MT1A, A2M, AHSG, APOA1, APOBR, APOE, APOL1, C1QB, C1QC, C1R, C1S, C3, C4B, C4BPA, C4BPB, C5, BRCC3, CFH, CLU, CP, CPN1, CPN2, DCD, FGA, FN1, HBA1, HBB, HP, IGFALS, ITIH1, ITIH2, ITIH3, ITIH4, JCHAIN, KLKB1, KNG1, KRT9, ORM2, PGLYRP2, PON1, PZP, S100A7, SEPP1, SERPINA1, SERPINA3, SERPINA6, SERPIND1, SHBG, TF, TTR, VTN, APLP1, APLP2, APP, PARP1, APOB, HPX, HRG, F11, APOBEC1, SEMG2*	PON1

DB14548	Zinc sulfate, unspecified form	approved, experimental	*BDKRB1, MGMT, ALDOA, EEF1A1, ENO1, GAPDHS, NME1, P4HB, PDIA3, PRDX1, PSPH, TPI1, TUFM, ESR1, IL3, MT2A, CCS, HDAC1, HDAC4, MPG, SEMG1, SOD1, HDAC8, SIVA1, GLRA1, MDM2, INS, UTRN, ASPA, S100A8, S100A9, MMP9, TP73, S100A2, TP53, MT3, MT1A, A2M, AHSG, APOA1, APOBR, APOE, APOL1, C1QB, C1QC, C1R, C1S, C3, C4B, C4BPA, C4BPB, C5, BRCC3, CFH, CLU, CP, CPN1, CPN2, DCD, FGA, FN1, HBA1, HBB, HP, IGFALS, ITIH1, ITIH2, ITIH3, ITIH4, JCHAIN, KLKB1, KNG1, KRT9, ORM2, PGLYRP2, PON1, PZP, S100A7, SEPP1, SERPINA1, SERPINA3, SERPINA6, SERPIND1, SHBG, TF, TTR, VTN, APLP1, APLP2, APP, PARP1, APOB, HPX, HRG, F11, APOBEC1, SEMG2*	PON1
DB02091	4-(2,4-Dimethyl-Thiazol-5-Yl)-Pyrimidin-2-Ylamine	experimental	*CDK2, CCNA2*	CCNA2
DB02407	6-O-Cyclohexylmethyl Guanine	experimental	*CDK2, CCNA2*	CCNA2
DB02833	[4-(2-Amino-4-Methyl-Thiazol-5-Yl)-Pyrimidin-2-Yl]-(3-Nitro-Phenyl)-Amine	experimental	*CDK2, CCNA2*	CCNA2
DB02915	4-(2,4-Dimethyl-1,3-thiazol-5-yl)-N-[4-(trifluoromethyl)phenyl]-2-pyrimidinamine	experimental	*CDK2, CCNA2*	CCNA2
DB06844	4-[(7-OXO-7H-THIAZOLO[5,4-E]INDOL-8-YLMETHYL)-AMINO]-N-PYRIDIN-2-YL-BENZENESULFONAMIDE	experimental	*CCNA2, CDK2*	CCNA2
DB06944	N-(3-cyclopropyl-1H-pyrazol-5-yl)-2-(2-naphthyl)acetamide	experimental	*CCNA2, CDK2*	CCNA2
DB06948	2-ANILINO-6-CYCLOHEXYLMETHOXYPURINE	experimental	*CCNA2, CDK2*	CCNA2
DB07126	O6-CYCLOHEXYLMETHOXY-2-(4-SULPHAMOYLANILINO) PURINE	experimental	*CCNA2, CDK2*	CCNA2
DB07137	(2S)-N-[(3E)-5-Cyclopropyl-3H-pyrazol-3-ylidene]-2-[4-(2-oxo-1-imidazolidinyl)phenyl]propanamide	experimental	*CCNA2, CDK2*	CCNA2
DB07164	N-cyclopropyl-4-pyrazolo[1,5-b]pyridazin-3-ylpyrimidin-2-amine	experimental	*CCNA2, CDK2*	CCNA2
DB07203	6-CYCLOHEXYLMETHOXY-2-(3-CHLOROANILINO) PURINE	experimental	*CCNA2, CDK2*	CCNA2
DB07471	5-[5,6-BIS(METHYLOXY)-1H-BENZIMIDAZOL-1-YL]-3-{[1-(2-CHLOROPHENYL)ETHYL]OXY}-2-THIOPHENECARBOXAMIDE	experimental	*CCNA2, CDK2*	CCNA2
DB07533	4-{5-[(Z)-(2-IMINO-4-OXO-1,3-THIAZOLIDIN-5-YLIDENE)METHYL]-2-FURYL}-N-METHYLBENZENESULFONAMIDE	experimental	*CCNA2, CDK2*	CCNA2
DB07534	4-{5-[(Z)-(2-IMINO-4-OXO-1,3-THIAZOLIDIN-5-YLIDENE)METHYL]FURAN-2-YL}BENZENESULFONAMIDE	experimental	*CCNA2, CDK2*	CCNA2

DB07538	4-{5-[(Z)-(2-IMINO-4-OXO-1,3-THIAZOLIDIN-5-YLIDENE)METHYL]FURAN-2-YL}-2-(TRIFLUOROMETHYL)BENZENESULFONAMIDE	experimental	*CCNA2, CDK2*	CCNA2
DB07539	4-{5-[(Z)-(2-IMINO-4-OXO-1,3-THIAZOLIDIN-5-YLIDENE)METHYL]FURAN-2-YL}BENZOIC ACID	experimental	*CCNA2, CDK2*	CCNA2
DB07562	N-[4-(2,4-DIMETHYL-THIAZOL-5-YL)-PYRIMIDIN-2-YL]-N,N-DIMETHYL-BENZENE-1,4-DIAMINE	experimental	*CDK2, CCNA2*	CCNA2
DB07688	4-{[5-(CYCLOHEXYLOXY)[1,2,4]TRIAZOLO[1,5-A]PYRIMIDIN-7-YL]AMINO}BENZENESULFONAMIDE	experimental	*CDK2, CCNA2*	CCNA2
DB07852	1-(3,5-DICHLOROPHENYL)-5-METHYL-1H-1,2,4-TRIAZOLE-3-CARBOXYLIC ACID	experimental	*CCNA2, CDK2*	CCNA2
DB08178	4-(4-methoxy-1H-pyrrolo[2,3-b]pyridin-3-yl)pyrimidin-2-amine	experimental	*CDK2, CCNA2*	CCNA2
DB08182	4-(4-propoxy-1H-pyrrolo[2,3-b]pyridin-3-yl)pyrimidin-2-amine	experimental	*CDK2, CCNA2*	CCNA2
DB08218	HYDROXY(OXO)(3-{[(2Z)-4-[3-(1H-1,2,4-TRIAZOL-1-YLMETHYL)PHENYL]PYRIMIDIN-2(5H)-YLIDENE]AMINO}PHENYL)AMMONIUM	experimental	*CCNA2, CDK2*	CCNA2
DB08219	4-Methyl-5-[(2Z)-2-{[4-(4-morpholinyl)phenyl]imino}-2,5-dihydro-4-pyrimidinyl]-1,3-thiazol-2-amine	experimental	*CCNA2, CDK2*	CCNA2
DB08233	6-CYCLOHEXYLMETHYLOXY-2-(4-HYDROXYANILINO)PURINE	experimental	*CCNA2, CDK2*	CCNA2
DB08241	4-(6-CYCLOHEXYLMETHOXY-9H-PURIN-2-YLAMINO)--BENZAMIDE	experimental	*CCNA2, CDK2*	CCNA2
DB08248	3-(6-CYCLOHEXYLMETHOXY-9H-PURIN-2-YLAMINO)-BENZENESULFONAMIDE	experimental	*CCNA2, CDK2*	CCNA2
DB08285	(2R)-2-{[4-(benzylamino)-8-(1-methylethyl)pyrazolo[1,5-a][1,3,5]triazin-2-yl]amino}butan-1-ol	experimental	*CDK2, CCNA2*	CCNA2
DB08309	3-({2-[(4-{[6-(CYCLOHEXYLMETHOXY)-9H-PURIN-2-YL]AMINO}PHENYL)SULFONYL]ETHYL}AMINO)PROPAN-1-OL	experimental	*CDK2, CCNA2*	CCNA2

DB08355	1-methyl-8-(phenylamino)-4,5-dihydro-1H-pyrazolo[4,3-h]quinazoline-3-carboxylic acid	experimental	*CCNA2, CDK2*	CCNA2
DB08463	(2R)-2-({9-(1-methylethyl)-6-[(4-pyridin-2-ylbenzyl)amino]-9H-purin-2-yl}amino)butan-1-ol	experimental	*CDK2, CCNA2*	CCNA2
DB08527	1-[4-(AMINOSULFONYL)PHENYL]-1,6-DIHYDROPYRAZOLO[3,4-E]INDAZOLE-3-CARBOXAMIDE	experimental	*CCNA2, CDK2*	CCNA2
DB08572	4-{[4-AMINO-6-(CYCLOHEXYLMETHOXY)-5-NITROSOPYRIMIDIN-2-YL]AMINO}BENZAMIDE	experimental	*CCNA2, CDK2*	CCNA2
DB08694	Variolin B	experimental	*CDK2, CCNA2*	CCNA2
DB00279	Liothyronine	approved, vet_approved	*THRA, THRB, PCNA*	PCNA
DB00945	Acetylsalicylic acid	approved, vet_approved	*PTGS1, PTGS2, AKR1C1, PRKAA1, EDNRA, TP53, HSPA5, RPS6KA3, NFKBIA, TNFAIP6, CASP1, CASP3, IKBKB, MAPK1, CCND1, MYC, PCNA, NEU1*	PCNA
DB00206	Reserpine	approved, investigational	*SLC18A2, SLC18A1, BIRC5*	BIRC5
DB04115	Berberine	approved, investigational	*qacR, BIRC5*	BIRC5
DB05141	LY2181308	investigational	*BIRC5*	BIRC5
DB02052	Indirubin-3-monoxime	experimental	*CDK5, CDK1, GSK3B, CDK5R1, CDK2, AHR*	CDK1
DB02116	Olomoucine	experimental	*CDK2, CDK5, CDK1, MAPK1*	CDK1
DB02950	Hymenialdisine	experimental	*CDK5, CDK1, CDK2*	CDK1
DB03428	SU9516	experimental	*CDK5, CDK1, CDK2*	CDK1
DB03496	Alvocidib	experimental, investigational	*CDK2, CDK5, CDK9, CDK1, CDK6, EGFR, CDK4, CDK8, CDK7, PYGM, PYGB*	CDK1
DB04014	Alsterpaullone	experimental	*CDK5, CDK1, GSK3B*	CDK1
DB06195	Seliciclib	investigational	*CDK1, CDK2, MAPK3, MAPK1, CDK7, CDK9, CSNK1E*	CDK1
DB08142	AT-7519	investigational	*CDK2, CDK1*	CDK1

DB12010	Fostamatinib	approved, investigational	*SYK, ADORA3, VMAT2, FAAH, SLC29A1, UGT1A1, PDE5A, ALOX5, CTSS, CTSL, ABL1, CPK1, pknB, RPS6KA6, TYK2, COQ8A, JAK1, MET, NIM1K, STK26, PRKACA, STK24, STK3, TEC, STK33, STK35, STK36, STK38, STK38L, STK39, TAOK1, TAOK2, TAOK3, TBK1, TEK, TESK1, TGFBR1, TGFBR2, TIE1, TLK1, TLK2, TNIK, TNK1, TNK2, TNNI3K, TSSK1B, TTK, TXK, TYRO3, ULK1, ULK2, ULK3, WEE1, YES1, MAP3K19, ZAK, ZAP70, AAK1, ABL2, ACVR1, ACVR1B, COQ8B, ALK, ANKK1, AURKA, AURKB, AURKC, AXL, BLK, BMP2K, BMPR1B, BMPR2, BMX, BRAF, BTK, CAMK1, CAMK1D, CAMK1G, CAMK2A, CAMK2B, CAMK2D, CAMK2G, CAMKK1, CAMKK2, CASK, CSN3, CDK1, CDC42BPG, CDK4, CDKL1, CDKL2, CHEK1, CHEK2, CIT, CLK1, CLK2, CLK3, CLK4, CSF1R, CSK, CSNK1A1, CSNK2A1, CSNK2A2, DAPK1, DAPK2, DAPK3, DCLK1, DCLK2, DCLK3, DDR1, DDR2, DYRK1A, DYRK1B, EGFR, EIF2AK1, EIF2AK2, EIF2AK4, EPHA1, EPHA2, EPHA3, EPHA4, EPHA5, EPHA6, EPHA7, EPHA8, EPHB1, EPHB2, EPHB4, EPHB6, ERBB2, ERBB4, ERN1, FER, FES, FGFR1, FGFR2, FGFR3, FGR, FLT1, FLT3, FLT4, MTOR, FRK, FYN, GAK, GSK3A, GSK3B, HCK, HIPK2, HIPK3, ICK, IKBKB, IKBKE, INSR, INSRR, IRAK1, IRAK3, IRAK4, ITK, JAK2, JAK3, KDR, SIK3, KIT, LATS1, LCK, LIMK1, LIMK2, LRRK2, LTK, LYN, MAP2K2, MAP2K3, MAP2K5, MAP2K6, MAP3K1, MAP3K10, MAP3K11, MAP3K12, MAP3K13, MAP3K15, MAP3K2, MAP3K3, MAP3K4, MAP3K6, MAP3K9, MAP4K1, MAP4K2, MAP4K3, MAP4K4, MAP4K5, MAPK10, MAPK13, MAPK14, MAPK15, MAPK4, MAPK7, MAPK9, MAPKAPK5, MARK1, MARK2, MARK3, MARK4, CLASP1, MAST1, MATK, MELK, MERTK, MINK1, MKNK1, MKNK2, MST1R, MUSK, MYLK, MYLK2, MYLK3, MYLK4, MYO3A, NEK1, NEK11, NEK2, NEK3, NEK4, NEK5, NEK9, NTRK1, NTRK2, NTRK3, NUAK1, NUAK2, OXSR1, PAK1, PAK2, PAK3, PAK4, PAK6, PAK5, CDK16, CDK17, PDGFRA, PDGFRB, PDPK1, CDK15, PHKG1, PI4KB, PIK3C2B, PIK3C2G, PIK3CD, PIK3CG, PIM1, PIM3, PIP4K2B, PIP4K2C, PKMYT1, PKN1, PKN2, PLK1, PLK2, PLK3, PLK4, PRKAA1, PRKAA1, PRKACB, PRKCD, PRKCE, PRKCG, PRKCI, PRKCQ, PRKD1, PRKG2, PRPF4B, PTK2, PTK2B, PTK6, RAF1, RET, RIOK1, RIOK2, RIOK3, RIPK1, RIPK2, RIPK4, ROCK2, ROS1, RPS6KA1, RPS6KA3, SBK1, SBK3, SGK3, SIK1, SIK2, SLK, SNRK, SRC, SRMS, STK10, STK16, STK17A, STK17B, STK32A*	CDK1
DB16652	Avotaciclib	investigational	*CDK1*	CDK1
DB03996	3-[(5s)-1-Acetyl-3-(2-Chlorophenyl)-4,5-Dihydro-1h-Pyrazol-5-Yl]Phenol	experimental	*KIF11*	KIF11
DB04331	Monastrol	experimental	*KIF11*	KIF11
DB06040	Filanesib	investigational	*KIF11*	KIF11
DB07064	(4R)-4-(3-HYDROXYPHENYL)-N,N,7,8-TETRAMETHYL-3,4-DIHYDROISOQUINOLINE-2(1H)-CARBOXAMIDE	experimental	*KIF11*	KIF11
DB08032	N,N-DIETHYL-2-[(2-THIENYLCARBONYL)AMINO]-4,5,6,7-TETRAHYDRO-1-BENZOTHIOPHENE-3-CARBOXAMIDE	experimental	*KIF11*	KIF11
DB08033	(5R)-N,N-DIETHYL-5-METHYL-2-[(THIOPHEN-2-YLCARBONYL)AMINO]-4,5,6,7-TETRAHYDRO-1-BENZOTHIOPHENE-3-CARBOXAMIDE	experimental	*KIF11*	KIF11

DB08037	MK-0731	experimental	*KIF11*	KIF11
DB08198	[(4R)-4-(3-HYDROXYPHENYL)-1,6-DIMETHYL-2-THIOXO-1,2,3,4-TETRAHYDROPYRIMIDIN-5-YL](PHENYL)METHANONE	experimental	*KIF11*	KIF11
DB08239	(2S)-4-(2,5-DIFLUOROPHENYL)-N-METHYL-2-PHENYL-N-PIPERIDIN-4-YL-2,5-DIHYDRO-1H-PYRROLE-1-CARBOXAMIDE	experimental	*KIF11*	KIF11
DB08244	(1S)-1-CYCLOPROPYL-2-[(2S)-4-(2,5-DIFLUOROPHENYL)-2-PHENYL-2,5-DIHYDRO-1H-PYRROL-1-YL]-2-OXOETHANAMINE	experimental	*KIF11*	KIF11
DB08246	(2S)-4-(2,5-DIFLUOROPHENYL)-N,N-DIMETHYL-2-PHENYL-2,5-DIHYDRO-1H-PYRROLE-1-CARBOXAMIDE	experimental	*KIF11*	KIF11
DB08250	(5S)-5-(3-AMINOPROPYL)-3-(2,5-DIFLUOROPHENYL)-N-ETHYL-5-PHENYL-4,5-DIHYDRO-1H-PYRAZOLE-1-CARBOXAMIDE	experimental	*KIF11*	KIF11

DB12010	Fostamatinib	approved, investigational	*SYK, ADORA3, VMAT2, FAAH, SLC29A1, UGT1A1, PDE5A, ALOX5, CTSS, CTSL, ABL1, CPK1, pknB, RPS6KA6, TYK2, COQ8A, JAK1, MET, NIM1K, STK26, PRKACA, STK24, STK3, TEC, STK33, STK35, STK36, STK38, STK38L, STK39, TAOK1, TAOK2, TAOK3, TBK1, TEK, TESK1, TGFBR1, TGFBR2, TIE1, TLK1, TLK2, TNIK, TNK1, TNK2, TNNI3K, TSSK1B, TTK, TXK, TYRO3, ULK1, ULK2, ULK3, WEE1, YES1, MAP3K19, ZAK, ZAP70, AAK1, ABL2, ACVR1, ACVR1B, COQ8B, ALK, ANKK1, AURKA, AURKB, AURKC, AXL, BLK, BMP2K, BMPR1B, BMPR2, BMX, BRAF, BTK, CAMK1, CAMK1D, CAMK1G, CAMK2A, CAMK2B, CAMK2D, CAMK2G, CAMKK1, CAMKK2, CASK, CSN3, CDK1, CDC42BPG, CDK4, CDKL1, CDKL2, CHEK1, CHEK2, CIT, CLK1, CLK2, CLK3, CLK4, CSF1R, CSK, CSNK1A1, CSNK2A1, CSNK2A2, DAPK1, DAPK2, DAPK3, DCLK1, DCLK2, DCLK3, DDR1, DDR2, DYRK1A, DYRK1B, EGFR, EIF2AK1, EIF2AK2, EIF2AK4, EPHA1, EPHA2, EPHA3, EPHA4, EPHA5, EPHA6, EPHA7, EPHA8, EPHB1, EPHB2, EPHB4, EPHB6, ERBB2, ERBB4, ERN1, FER, FES, FGFR1, FGFR2, FGFR3, FGR, FLT1, FLT3, FLT4, MTOR, FRK, FYN, GAK, GSK3A, GSK3B, HCK, HIPK2, HIPK3, ICK, IKBKB, IKBKE, INSR, INSRR, IRAK1, IRAK3, IRAK4, ITK, JAK2, JAK3, KDR, SIK3, KIT, LATS1, LCK, LIMK1, LIMK2, LRRK2, LTK, LYN, MAP2K2, MAP2K3, MAP2K5, MAP2K6, MAP3K1, MAP3K10, MAP3K11, MAP3K12, MAP3K13, MAP3K15, MAP3K2, MAP3K3, MAP3K4, MAP3K6, MAP3K9, MAP4K1, MAP4K2, MAP4K3, MAP4K4, MAP4K5, MAPK10, MAPK13, MAPK14, MAPK15, MAPK4, MAPK7, MAPK9, MAPKAPK5, MARK1, MARK2, MARK3, MARK4, CLASP1, MAST1, MATK, MELK, MERTK, MINK1, MKNK1, MKNK2, MST1R, MUSK, MYLK, MYLK2, MYLK3, MYLK4, MYO3A, NEK1, NEK11, NEK2, NEK3, NEK4, NEK5, NEK9, NTRK1, NTRK2, NTRK3, NUAK1, NUAK2, OXSR1, PAK1, PAK2, PAK3, PAK4, PAK6, PAK5, CDK16, CDK17, PDGFRA, PDGFRB, PDPK1, CDK15, PHKG1, PI4KB, PIK3C2B, PIK3C2G, PIK3CD, PIK3CG, PIM1, PIM3, PIP4K2B, PIP4K2C, PKMYT1, PKN1, PKN2, PLK1, PLK2, PLK3, PLK4, PRKAA1, PRKAA1, PRKACB, PRKCD, PRKCE, PRKCG, PRKCI, PRKCQ, PRKD1, PRKG2, PRPF4B, PTK2, PTK2B, PTK6, RAF1, RET, RIOK1, RIOK2, RIOK3, RIPK1, RIPK2, RIPK4, ROCK2, ROS1, RPS6KA1, RPS6KA3, SBK1, SBK3, SGK3, SIK1, SIK2, SLK, SNRK, SRC, SRMS, STK10, STK16, STK17A, STK17B, STK32A*	MELK
DB01782	Pyrazolanthrone	experimental	*MAPK10, MAPK8IP1, MAPK8, TTK*	TTK

DB12010	Fostamatinib	approved, investigational	*SYK, ADORA3, VMAT2, FAAH, SLC29A1, UGT1A1, PDE5A, ALOX5, CTSS, CTSL, ABL1, CPK1, pknB, RPS6KA6, TYK2, COQ8A, JAK1, MET, NIM1K, STK26, PRKACA, STK24, STK3, TEC, STK33, STK35, STK36, STK38, STK38L, STK39, TAOK1, TAOK2, TAOK3, TBK1, TEK, TESK1, TGFBR1, TGFBR2, TIE1, TLK1, TLK2, TNIK, TNK1, TNK2, TNNI3K, TSSK1B, TTK, TXK, TYRO3, ULK1, ULK2, ULK3, WEE1, YES1, MAP3K19, ZAK, ZAP70, AAK1, ABL2, ACVR1, ACVR1B, COQ8B, ALK, ANKK1, AURKA, AURKB, AURKC, AXL, BLK, BMP2K, BMPR1B, BMPR2, BMX, BRAF, BTK, CAMK1, CAMK1D, CAMK1G, CAMK2A, CAMK2B, CAMK2D, CAMK2G, CAMKK1, CAMKK2, CASK, CSN3, CDK1, CDC42BPG, CDK4, CDKL1, CDKL2, CHEK1, CHEK2, CIT, CLK1, CLK2, CLK3, CLK4, CSF1R, CSK, CSNK1A1, CSNK2A1, CSNK2A2, DAPK1, DAPK2, DAPK3, DCLK1, DCLK2, DCLK3, DDR1, DDR2, DYRK1A, DYRK1B, EGFR, EIF2AK1, EIF2AK2, EIF2AK4, EPHA1, EPHA2, EPHA3, EPHA4, EPHA5, EPHA6, EPHA7, EPHA8, EPHB1, EPHB2, EPHB4, EPHB6, ERBB2, ERBB4, ERN1, FER, FES, FGFR1, FGFR2, FGFR3, FGR, FLT1, FLT3, FLT4, MTOR, FRK, FYN, GAK, GSK3A, GSK3B, HCK, HIPK2, HIPK3, ICK, IKBKB, IKBKE, INSR, INSRR, IRAK1, IRAK3, IRAK4, ITK, JAK2, JAK3, KDR, SIK3, KIT, LATS1, LCK, LIMK1, LIMK2, LRRK2, LTK, LYN, MAP2K2, MAP2K3, MAP2K5, MAP2K6, MAP3K1, MAP3K10, MAP3K11, MAP3K12, MAP3K13, MAP3K15, MAP3K2, MAP3K3, MAP3K4, MAP3K6, MAP3K9, MAP4K1, MAP4K2, MAP4K3, MAP4K4, MAP4K5, MAPK10, MAPK13, MAPK14, MAPK15, MAPK4, MAPK7, MAPK9, MAPKAPK5, MARK1, MARK2, MARK3, MARK4, CLASP1, MAST1, MATK, MELK, MERTK, MINK1, MKNK1, MKNK2, MST1R, MUSK, MYLK, MYLK2, MYLK3, MYLK4, MYO3A, NEK1, NEK11, NEK2, NEK3, NEK4, NEK5, NEK9, NTRK1, NTRK2, NTRK3, NUAK1, NUAK2, OXSR1, PAK1, PAK2, PAK3, PAK4, PAK6, PAK5, CDK16, CDK17, PDGFRA, PDGFRB, PDPK1, CDK15, PHKG1, PI4KB, PIK3C2B, PIK3C2G, PIK3CD, PIK3CG, PIM1, PIM3, PIP4K2B, PIP4K2C, PKMYT1, PKN1, PKN2, PLK1, PLK2, PLK3, PLK4, PRKAA1, PRKAA1, PRKACB, PRKCD, PRKCE, PRKCG, PRKCI, PRKCQ, PRKD1, PRKG2, PRPF4B, PTK2, PTK2B, PTK6, RAF1, RET, RIOK1, RIOK2, RIOK3, RIPK1, RIPK2, RIPK4, ROCK2, ROS1, RPS6KA1, RPS6KA3, SBK1, SBK3, SGK3, SIK1, SIK2, SLK, SNRK, SRC, SRMS, STK10, STK16, STK17A, STK17B, STK32A*	TTK
DB15498	BOS172722	experimental	*TTK*	TTK
DB00893	Iron Dextran	approved, vet_approved	*HBB, HBA1, FTH1, FTL*	FTH1
DB01592	Iron	approved	*TFRC, EGLN1, HDAC8, AHSP, HBA1, FXN, FTH1, FEN1, NEIL1, NEIL2, POLB, CP, TF*	FTH1
DB13995	Ferric pyrophosphate citrate	approved, investigational	*FTL, FTH1, HBA1, HBB*	FTH1
DB14488	Ferrous gluconate	approved	*TFRC, EGLN1, HDAC8, AHSP, HBA1, FXN, FTH1, FEN1, NEIL1, NEIL2, POLB, CP, TF*	FTH1
DB14489	Ferrous succinate	approved	*TFRC, EGLN1, HDAC8, AHSP, HBA1, FXN, FTH1, FEN1, NEIL1, NEIL2, POLB, CP, TF*	FTH1
DB14490	Ferrous ascorbate	approved	*TFRC, EGLN1, HDAC8, AHSP, HBA1, FXN, FTH1, FEN1, NEIL1, NEIL2, POLB, CP, TF*	FTH1
DB14491	Ferrous fumarate	approved	*TFRC, EGLN1, HDAC8, AHSP, HBA1, FXN, FTH1, FEN1, NEIL1, NEIL2, POLB, CP, TF*	FTH1
DB14501	Ferrous glycine sulfate	approved	*TFRC, EGLN1, HDAC8, AHSP, HBA1, FXN, FTH1, FEN1, NEIL1, NEIL2, POLB, CP, TF*	FTH1
DB02255	Ilomastat	experimental	*lef, ADAM28, ACAN*	ACAN
DB05121	1D09C3	investigational	*HLA-DRA*	HLA-DRA
DB11294	Coccidioides immitis spherule	approved	*HLA-A, HLA-DRA, HLA-A, HLA-B*	HLA-DRA
DB08818	Hyaluronic acid	approved, vet_approved	*CD44, ICAM1, HMMR, NCAN, VCAN, C1QBP, HAPLN1, HAPLN3, HABP2, LAYN, STAB2, TNFAIP6, IMPG2, HABP4, LYVE1*	HAPLN1
DB08818	Hyaluronic acid	approved, vet_approved	*CD44, ICAM1, HMMR, NCAN, VCAN, C1QBP, HAPLN1, HAPLN3, HABP2, LAYN, STAB2, TNFAIP6, IMPG2, HABP4, LYVE1*	HAPLN3

DB09130	Copper	approved, investigational	*APP, AHCY, HIST1H2BC, GAPDH, NME1, HIST1H1E, PRDX1, S100A8, RPSA, ACTG1, ENO1, EEF1A1, KRT8, P4HB, PDIA3, HSPD1, HSPA13, HSPA5, HSP90B1, TF, Lyar, RPS2, SRSF1, HNRNPA2B1, HNRNPH1, HNRNPH3, cbiD, HNRNPL, SFPQ, SF3A2, RACK1, ACTN1, ACY1, ANXA4, ANXA5, CALR, PKM, AKR1A1, CYB5R3, GSR, TKT, PRDX2, PRDX6, PPIA, HSPA8, HSP90AA1, PTGES3, STIP1, EEF1A1L14, EIF6, EIF4A1, GPI, LDHA, PGK1, TUBA3C, TUBB, CFL1, YWHAB, GOT1, GSS, HDGF, IDH3A, CLIC1, PSME1, PEBP1, PGAM1, RANBP1, UGDH, B2M, SCO1, PRNP, GLRA1, HTT, NEIL1, NEIL2, HEPHL1, SERPINE1, S100A2, S100A4, SNCA, BDNF, PARK7, IAPP, TAC3, A1BG, AFM, AGT, AHSG, APCS, APOA1, APOA2, APOA4, APOBR, APOC2, APOC3, APOD, APOE, APOH, AZGP1, C1QC, C1S, C3, C4B, C4BPA, C5, C8B, C9, CFH, CFI, CLEC3B, CLU, F2, C1QBP, GSN, HBA1, HBB, CBX5, HPR, IGFALS, IGHG1, IGHG4, IGLL1, ITIH2, KNG1, KRT1, KRT10, KRT2, KRT9, LRG1, LUM, PGLYRP2, PLG, PON1, PPBP, SERPINA1, SERPINA4, SERPINA6, SERPINA7, SERPINC1, SERPIND1, SERPINF1, SERPINF2, SERPING1, TTR, VTN, APLP1*	HEPHL1
DB04472	(R)-1-Para-Nitro-Phenyl-2-Azido-Ethanol	experimental	*hheC, LGALS9*	LGALS9
DB01694	D-tartaric acid	experimental	*dlgD, B3GAT1, TREM1, DDX6, HIF1AN, serA, lpxA, ATP6V1C1, rmlC, phnA*	DDX6
DB02494	(S)-3-phenyllactic acid	experimental	*pdh, RPL10L, RPL13A, RPL23, RPL15, RPL19, RPL23A, RSL24D1, RPL26L1, RPL8, RPL37, RPL3, RPL11, SNU13, CPA1*	RPL11
DB07374	Anisomycin	experimental	*RPL10L, RPL13A, RPL23, RPL15, RPL19, RPL23A, RSL24D1, RPL26L1, RPL8, RPL37, RPL3, RPL11, SNU13*	RPL11
DB08437	Puromycin	experimental	*RPL10L, RPL13A, RPL23, RPL15, RPL19, RPL23A, RSL24D1, RPL26L1, RPL8, RPL37, RPL3, RPL11, aat*	RPL11
DB02494	(S)-3-phenyllactic acid	experimental	*pdh, RPL10L, RPL13A, RPL23, RPL15, RPL19, RPL23A, RSL24D1, RPL26L1, RPL8, RPL37, RPL3, RPL11, SNU13, CPA1*	RPL19
DB07374	Anisomycin	experimental	*RPL10L, RPL13A, RPL23, RPL15, RPL19, RPL23A, RSL24D1, RPL26L1, RPL8, RPL37, RPL3, RPL11, SNU13*	RPL19
DB08437	Puromycin	experimental	*RPL10L, RPL13A, RPL23, RPL15, RPL19, RPL23A, RSL24D1, RPL26L1, RPL8, RPL37, RPL3, RPL11, aat*	RPL19
DB02494	(S)-3-phenyllactic acid	experimental	*pdh, RPL10L, RPL13A, RPL23, RPL15, RPL19, RPL23A, RSL24D1, RPL26L1, RPL8, RPL37, RPL3, RPL11, SNU13, CPA1*	RPL8
DB07374	Anisomycin	experimental	*RPL10L, RPL13A, RPL23, RPL15, RPL19, RPL23A, RSL24D1, RPL26L1, RPL8, RPL37, RPL3, RPL11, SNU13*	RPL8
DB08437	Puromycin	experimental	*RPL10L, RPL13A, RPL23, RPL15, RPL19, RPL23A, RSL24D1, RPL26L1, RPL8, RPL37, RPL3, RPL11, aat*	RPL8
DB00054	Abciximab	approved	*ITGB3, ITGA2B, FCGR2A, FCGR2B, VTN*	ITGA2B
DB00775	Tirofiban	approved	*ITGA2B, ITGB3*	ITGA2B
DB04863	Lefradafiban	investigational	*ITGA2B, ITGB3*	ITGA2B
DB06472	Fradafiban	investigational	*ITGA2B, ITGB3*	ITGA2B
DB00098	Antithymocyte immunoglobulin (rabbit)	approved	*CD1A, MR1, ITGAL, CD86, FCGR2B, CD4, ITGB1, ITGAV, ITGB3*	ITGAV
DB00451	Levothyroxine	approved	*THRA, THRB, ITGAV, ITGB3*	ITGAV
DB00098	Antithymocyte immunoglobulin (rabbit)	approved	*CD1A, MR1, ITGAL, CD86, FCGR2B, CD4, ITGB1, ITGAV, ITGB3*	ITGB1
DB00054	Abciximab	approved	*ITGB3, ITGA2B, FCGR2A, FCGR2B, VTN*	ITGB3
DB00063	Eptifibatide	approved, investigational	*ITGB3*	ITGB3

DB00098	Antithymocyte immunoglobulin (rabbit)	approved	*CD1A, MR1, ITGAL, CD86, FCGR2B, CD4, ITGB1, ITGAV, ITGB3*	ITGB3
DB00451	Levothyroxine	approved	*THRA, THRB, ITGAV, ITGB3*	ITGB3
DB00775	Tirofiban	approved	*ITGA2B, ITGB3*	ITGB3
DB02709	Resveratrol	investigational	*NQO2, CSNK2A1, PTGS1, PTGS2, ALOX15, ALOX5, AHR, PI4K2B, ITGA5, ITGB3, APP, SNCA, SIRT1, ESR1, MTNR1A, MTNR1B, CLEC14A, NR1I2, NR1I3, SLC2A1, CBR1, PPARA, PPARG, AKT1, KHSRP, YARS*	ITGB3
DB04863	Lefradafiban	investigational	*ITGA2B, ITGB3*	ITGB3
DB05787	LM-609	investigational	*ITGB3*	ITGB3
DB06472	Fradafiban	investigational	*ITGA2B, ITGB3*	ITGB3
DB15598	Ferric maltol	approved	*ITGB3, SLC11A2*	ITGB3
DB00594	Amiloride	approved	*SCNN1A, SCNN1B, SCNN1G, SCNN1D, AOC1, ASIC2, ASIC1, SLC9A1, PLAU*	PLAU
DB01725	2-{2-hydroxy-[1,1-biphenyl]-3-yl}-1H-1,3-benzodiazole-5-carboximidamide	experimental	*PRSS1, F2, PLAU*	PLAU
DB01905	2-(2-Hydroxy-5-Methoxy-Phenyl)-1h-Benzoimidazole-5-Carboxamidine	experimental	*PRSS1, PLAU*	PLAU
DB01977	6-(N-Phenylcarbamyl)-2-Naphthalenecarboxamidine	experimental	*PLAU*	PLAU
DB02287	2-(2-hydroxy-phenyl)-3H-benzoimidazole-5-carboxamidine	experimental	*F2, PRSS1, PLAU*	PLAU
DB02398	6-[N-(4-(Aminomethyl)Phenyl)Carbamyl]-2-Naphthalenecarboxamidine	experimental	*PLAU*	PLAU
DB02473	6-[N-(1-Isopropyl-3,4-Dihydro-7-Isoquinolinyl)Carbamyl]-2-Naphthalenecarboxamidine	experimental	*PLAU*	PLAU
DB02526	CRA_10655	experimental	*PRSS1, PLAU*	PLAU
DB02551	6-[N-(4-Ethyl-1,2,3,4-Tetrahydro-6-Isoquinolinyl)Carbamyl]-2-Naphthalenecarboxamidine	experimental	*PLAU*	PLAU
DB02705	6-[N-(1-Isopropyl-1,2,3,4-Tetrahydro-7-Isoquinolinyl)Carbamyl]-2-Naphthalenecarboxamidine	experimental	*PLAU*	PLAU
DB03046	7-Methoxy-8-[1-(Methylsulfonyl)-1h-Pyrazol-4-Yl]Naphthalene-2-Carboximidamide	experimental	*PLAU*	PLAU
DB03082	6-[(Z)-Amino(Imino)Methyl]-N-[4-(Aminomethyl)Phenyl]-4-(Pyrimidin-2-Ylamino)-2-Naphthamide	experimental	*PLAU*	PLAU
DB03127	Benzamidine	experimental	*KLK6, PRSS2, PRSS3, ATOX1, ECI1, sprT, ST14, PLAU, PRSS1, CSNK2A1, KLK1*	PLAU
DB03136	4-Iodobenzo[B]Thiophene-2-Carboxamidine	experimental	*F2, PRSS1, PLAU*	PLAU
DB03159	CRA_8696	experimental	*F2, PRSS1, PLAU*	PLAU
DB03476	Trans-6-(2-Phenylcyclopropyl)-Naphthalene-2-Carboxamidine	experimental	*PLAU*	PLAU
DB03729	2-Amino-1H-benzimidazol-5-ol	experimental	*PLAU*	PLAU

DB03782	N-(1-adamantyl)-N-(4-guanidinobenzyl)urea	experimental	*PLAU*	PLAU
DB03865	6-Chloro-2-(2-Hydroxy-Biphenyl-3-Yl)-1h-Indole-5-Carboxamidine	experimental	*F2, PRSS1, PLAU, HPN*	PLAU
DB03876	Thieno[2,3-B]Pyridine-2-Carboxamidine	experimental	*PRSS1, PLAU*	PLAU
DB04059	8-(Pyrimidin-2-Ylamino)Naphthalene-2-Carboximidamide	experimental	*PLAU*	PLAU
DB04172	[2,4,6-Triisopropyl-Phenylsulfonyl-L-[3-Amidino-Phenylalanine]]-Piperazine-N-Beta-Alanine	experimental	*PLAU*	PLAU
DB05254	Fibrinolysin	investigational	*SERPINE1, PLAU*	PLAU
DB06855	6-fluoro-2-(2-hydroxy-3-isobutoxy-phenyl)-1H-benzoimidazole-5-carboxamidine	experimental	*PLAU, PRSS1*	PLAU
DB06856	6-FLUORO-2-[2-HYDROXY-3-(2-METHYL-CYCLOHEXYLOXY)-PHENYL]-1H-INDOLE-5-CARBOXAMIDINE	experimental	*PLAU*	PLAU
DB06857	N-(4-CARBAMIMIDOYL-3-CHORO-PHENYL)-2-HYDROXY-3-IODO-5-METHYL-BENZAMIDE	experimental	*PLAU*	PLAU
DB07076	6-[(Z)-AMINO(IMINO)METHYL]-N-[3-(CYCLOPENTYLOXY)PHENYL]-2-NAPHTHAMIDE	experimental	*PLAU*	PLAU
DB07122	1-[4-(2-oxo-2-phenylethyl)phenyl]guanidine	experimental	*PLAU*	PLAU
DB07129	(2R)-1-(2,6-dimethylphenoxy)propan-2-amine	experimental	*PLAU*	PLAU
DB07625	4-(2-aminoethoxy)-N-(2,5-diethoxyphenyl)-3,5-dimethylbenzamide	experimental	*PLAU*	PLAU
DB07626	4-(2-aminoethoxy)-N-(3-chloro-2-ethoxy-5-piperidin-1-ylphenyl)-3,5-dimethylbenzamide	experimental	*PLAU*	PLAU
DB08072	4-(2-AMINOETHOXY)-3,5-DICHLORO-N-[3-(1-METHYLETHOXY)PHENYL]BENZAMIDE	experimental	*PLAU*	PLAU
DB08697	4-(2-aminoethoxy)-N-(3-chloro-5-piperidin-1-ylphenyl)-3,5-dimethylbenzamide	experimental	*PLAU*	PLAU
DB00009	Alteplase	approved	*PLG, FGA, PLAUR, SERPINE1*	PLAUR
DB00013	Urokinase	approved, investigational, withdrawn	*PLG, PLAUR, SERPINE1, SERPINB2, SERPINA5, LRP2, ST14, NID1*	PLAUR
DB00031	Tenecteplase	approved	*PLG, FGA, PLAUR, SERPINE1, SERPINB2, CLEC3B, KRT8, ANXA2, CALR, CANX, LRP1*	PLAUR
DB05476	WX-UK1	investigational	*PLAUR*	PLAUR

DB06245	Lanoteplase	investigational	*PLAUR, FGA, KLK1, LAMA5, F10, FN1, SERPINB2, CLEC3B, KRT8, ANXA2, LAMB1, LAMC1, LAMA1, CALR, CANX, LRP1, LAMA3*	PLAUR
DB00009	Alteplase	approved	*PLG, FGA, PLAUR, SERPINE1*	SERPINE1
DB00013	Urokinase	approved, investigational, withdrawn	*PLG, PLAUR, SERPINE1, SERPINB2, SERPINA5, LRP2, ST14, NID1*	SERPINE1
DB00015	Reteplase	approved, investigational	*PLG, FGA, SERPINE1*	SERPINE1
DB00029	Anistreplase	approved	*PLG, FGA, SERPINE1*	SERPINE1
DB00031	Tenecteplase	approved	*PLG, FGA, PLAUR, SERPINE1, SERPINB2, CLEC3B, KRT8, ANXA2, CALR, CANX, LRP1*	SERPINE1
DB00055	Drotrecogin alfa	approved, investigational, withdrawn	*F8, F5, SERPINE1, THBD, PROS1, F2, PF4, SERPINA5, SERPINB6, PROCR*	SERPINE1
DB00197	Troglitazone	approved, investigational, withdrawn	*PPARG, ACSL4, SERPINE1, SLC29A1, ESRRG, ESRRA, PPARD, PPARA, GSTP1*	SERPINE1
DB05254	Fibrinolysin	investigational	*SERPINE1, PLAU*	SERPINE1
DB09130	Copper	approved, investigational	*APP, AHCY, HIST1H2BC, GAPDH, NME1, HIST1H1E, PRDX1, S100A8, RPSA, ACTG1, ENO1, EEF1A1, KRT8, P4HB, PDIA3, HSPD1, HSPA13, HSPA5, HSP90B1, TF, Lyar, RPS2, SRSF1, HNRNPA2B1, HNRNPH1, HNRNPH3, cbiD, HNRNPL, SFPQ, SF3A2, RACK1, ACTN1, ACY1, ANXA4, ANXA5, CALR, PKM, AKR1A1, CYB5R3, GSR, TKT, PRDX2, PRDX6, PPIA, HSPA8, HSP90AA1, PTGES3, STIP1, EEF1A1L14, EIF6, EIF4A1, GPI, LDHA, PGK1, TUBA3C, TUBB, CFL1, YWHAB, GOT1, GSS, HDGF, IDH3A, CLIC1, PSME1, PEBP1, PGAM1, RANBP1, UGDH, B2M, SCO1, PRNP, GLRA1, HTT, NEIL1, NEIL2, HEPHL1, SERPINE1, S100A2, S100A4, SNCA, BDNF, PARK7, IAPP, TAC3, A1BG, AFM, AGT, AHSG, APCS, APOA1, APOA2, APOA4, APOBR, APOC2, APOC3, APOD, APOE, APOH, AZGP1, C1QC, C1S, C3, C4B, C4BPA, C5, C8B, C9, CFH, CFI, CLEC3B, CLU, F2, C1QBP, GSN, HBA1, HBB, CBX5, HPR, IGFALS, IGHG1, IGHG4, IGLL1, ITIH2, KNG1, KRT1, KRT10, KRT2, KRT9, LRG1, LUM, PGLYRP2, PLG, PON1, PPBP, SERPINA1, SERPINA4, SERPINA6, SERPINA7, SERPINC1, SERPIND1, SERPINF1, SERPINF2, SERPING1, TTR, VTN, APLP1*	SERPINE1
DB00054	Abciximab	approved	*ITGB3, ITGA2B, FCGR2A, FCGR2B, VTN*	VTN
DB01593	Zinc	approved, investigational	*BDKRB1, MGMT, ALDOA, EEF1A1, ENO1, GAPDHS, NME1, P4HB, PDIA3, PRDX1, PSPH, TPI1, TUFM, ESR1, IL3, MT2A, CCS, HDAC1, HDAC4, MPG, SEMG1, SOD1, HDAC8, SIVA1, GLRA1, MDM2, INS, UTRN, ASPA, S100A8, S100A9, MMP9, TP73, S100A2, TP53, MT3, PDCD6, DAND5, MT1A, A1BG, A2M, AGT, AHSG, APCS, APOA1, APOA2, APOA4, APOBR, APOE, APOL1, C1QB, C1QC, C1R, C1S, C3, C4B, C4BPA, C4BPB, C5, BRCC3, C8A, C8B, C8G, CFB, CFH, CFI, CLU, CP, CPN1, CPN2, DCD, DSP, F12, F13B, F2, FCN3, FGA, FN1, GSN, HBA1, HBB, HPR, HRNR, IGFALS, IGHA1, IGHM, IGKV1-17, IGLV3-21, ITIH1, ITIH2, ITIH3, ITIH4, JCHAIN, JUP, KLKB1, KNG1, KRT1, KRT10, KRT14, KRT16, KRT2, KRT5, KRT6A, KRT9, ORM2, PGLYRP2, PON1, PZP, S100A7, SEPP1, SERPINA1, SERPINA3, SERPINA4, SERPINA6, SERPIND1, SHBG, TF, TTR, VTN, APLP1, APLP2, APP, PARP1*	VTN

DB09130	Copper	approved, investigational	*APP, AHCY, HIST1H2BC, GAPDH, NME1, HIST1H1E, PRDX1, S100A8, RPSA, ACTG1, ENO1, EEF1A1, KRT8, P4HB, PDIA3, HSPD1, HSPA13, HSPA5, HSP90B1, TF, Lyar, RPS2, SRSF1, HNRNPA2B1, HNRNPH1, HNRNPH3, cbiD, HNRNPL, SFPQ, SF3A2, RACK1, ACTN1, ACY1, ANXA4, ANXA5, CALR, PKM, AKR1A1, CYB5R3, GSR, TKT, PRDX2, PRDX6, PPIA, HSPA8, HSP90AA1, PTGES3, STIP1, EEF1A1L14, EIF6, EIF4A1, GPI, LDHA, PGK1, TUBA3C, TUBB, CFL1, YWHAB, GOT1, GSS, HDGF, IDH3A, CLIC1, PSME1, PEBP1, PGAM1, RANBP1, UGDH, B2M, SCO1, PRNP, GLRA1, HTT, NEIL1, NEIL2, HEPHL1, SERPINE1, S100A2, S100A4, SNCA, BDNF, PARK7, IAPP, TAC3, A1BG, AFM, AGT, AHSG, APCS, APOA1, APOA2, APOA4, APOBR, APOC2, APOC3, APOD, APOE, APOH, AZGP1, C1QC, C1S, C3, C4B, C4BPA, C5, C8B, C9, CFH, CFI, CLEC3B, CLU, F2, C1QBP, GSN, HBA1, HBB, CBX5, HPR, IGFALS, IGHG1, IGHG4, IGLL1, ITIH2, KNG1, KRT1, KRT10, KRT2, KRT9, LRG1, LUM, PGLYRP2, PLG, PON1, PPBP, SERPINA1, SERPINA4, SERPINA6, SERPINA7, SERPINC1, SERPIND1, SERPINF1, SERPINF2, SERPING1, TTR, VTN, APLP1*	VTN
DB14487	Zinc acetate	approved, investigational	*BDKRB1, MGMT, ALDOA, EEF1A1, ENO1, GAPDHS, NME1, P4HB, PDIA3, PRDX1, PSPH, TPI1, TUFM, ESR1, IL3, MT2A, CCS, HDAC1, HDAC4, MPG, SEMG1, SOD1, HDAC8, SIVA1, GLRA1, MDM2, INS, UTRN, ASPA, S100A8, S100A9, MMP9, TP73, S100A2, TP53, MT3, PDCD6, DAND5, MT1A, A1BG, A2M, AGT, AHSG, APCS, APOA1, APOA2, APOA4, APOBR, APOE, APOL1, C1QB, C1QC, C1R, C1S, C3, C4B, C4BPA, C4BPB, C5, BRCC3, C8A, C8B, C8G, CFB, CFH, CFI, CLU, CP, CPN1, CPN2, DCD, DSP, F12, F13B, F2, FCN3, FGA, FN1, GSN, HBA1, HBB, HPR, HRNR, IGFALS, IGHA1, IGHM, IGKV1-17, IGLV3-21, ITIH1, ITIH2, ITIH3, ITIH4, JCHAIN, JUP, KLKB1, KNG1, KRT1, KRT10, KRT14, KRT16, KRT2, KRT5, KRT6A, KRT9, ORM2, PGLYRP2, PON1, PZP, S100A7, SEPP1, SERPINA1, SERPINA3, SERPINA4, SERPINA6, SERPIND1, SHBG, TF, TTR, VTN, APLP1, APLP2, APP, PARP1*	VTN
DB14533	Zinc chloride	approved, investigational	*BDKRB1, MGMT, ALDOA, EEF1A1, ENO1, GAPDHS, NME1, P4HB, PDIA3, PRDX1, PSPH, TPI1, TUFM, ESR1, IL3, MT2A, CCS, HDAC1, HDAC4, MPG, SEMG1, SOD1, HDAC8, SIVA1, GLRA1, MDM2, INS, UTRN, ASPA, S100A8, S100A9, MMP9, TP73, S100A2, TP53, MT3, MT1A, A2M, AHSG, APOA1, APOBR, APOE, APOL1, C1QB, C1QC, C1R, C1S, C3, C4B, C4BPA, C4BPB, C5, BRCC3, CFH, CLU, CP, CPN1, CPN2, DCD, FGA, FN1, HBA1, HBB, HP, IGFALS, ITIH1, ITIH2, ITIH3, ITIH4, JCHAIN, KLKB1, KNG1, KRT9, ORM2, PGLYRP2, PON1, PZP, S100A7, SEPP1, SERPINA1, SERPINA3, SERPINA6, SERPIND1, SHBG, TF, TTR, VTN, APLP1, APLP2, APP, PARP1, APOB, HPX, HRG, F11, APOBEC1, SEMG2*	VTN
DB14548	Zinc sulfate, unspecified form	approved, experimental	*BDKRB1, MGMT, ALDOA, EEF1A1, ENO1, GAPDHS, NME1, P4HB, PDIA3, PRDX1, PSPH, TPI1, TUFM, ESR1, IL3, MT2A, CCS, HDAC1, HDAC4, MPG, SEMG1, SOD1, HDAC8, SIVA1, GLRA1, MDM2, INS, UTRN, ASPA, S100A8, S100A9, MMP9, TP73, S100A2, TP53, MT3, MT1A, A2M, AHSG, APOA1, APOBR, APOE, APOL1, C1QB, C1QC, C1R, C1S, C3, C4B, C4BPA, C4BPB, C5, BRCC3, CFH, CLU, CP, CPN1, CPN2, DCD, FGA, FN1, HBA1, HBB, HP, IGFALS, ITIH1, ITIH2, ITIH3, ITIH4, JCHAIN, KLKB1, KNG1, KRT9, ORM2, PGLYRP2, PON1, PZP, S100A7, SEPP1, SERPINA1, SERPINA3, SERPINA6, SERPIND1, SHBG, TF, TTR, VTN, APLP1, APLP2, APP, PARP1, APOB, HPX, HRG, F11, APOBEC1, SEMG2*	VTN

Supplementary Table 13. PRS models tested and their prediction ability.

Model	Approach	SNP subset	N. SNPs candidates	N. SNPs in the model	AUC	AUC CI 95%	Sensitivity at 0.9 specificity	Sensitivity CI 95 %	Sensitivity at 0.8 specificity	Sensitivity CI 95%
Non-HLA PRS	Clumpling and p-value thresholding	Non-HLA suggestive or significant independent SNPs	78	39	0.56	0.53-0.58	0.13	0.11 - 0.15	0.23	0.21 - 0.26
HLA PRS	Conditional analysis and p-value thresholding	Identified HLA independent variants	5	5	0.61	0.59-0.63	0.21	0.19 - 0.24	0.34	0.32 - 0.37
Complete PRS	HLA variants + Clumpling and p-value thresholding for non-HLA SNPs	HLA independent variants and non-HLA suggestive or significant independent SNPs	5+78	5+6	0.62	0.59-0.64	0.23	0.20 - 0.25	0.38	0.35 - 0.41
Complete PRS + Sex	Complete PRS model using sex as covariate	Selected SNPs in the complete PRS model	-	5+6	0.62	0.59-0.64	0.22	0.20 - 0.24	0.38	0.35 - 0.41

PRS, polygenic risk score; SNP, single nucleotide polymorphism; AUC, area under the curve; CI, Confidence Interval

Supplementary Table 14. HLA+non-HLA PRS model and variant weights

Variant ID	A1	Log(OR)	OR [CI 95]% *
rs17882084	A	0.70	2.02 [1.88- 2.17]
rs1049087	A	0.40	1.56 [1.47- 1.66]
rs2596501	G	0.18	1.19 [1.12- 1.27]
rs2856726	A	0.16	1.19 [1.12- 1.27]
HLA-DPB1*03	P	-0.26	0.77 [0.69- 0.86]
rs45485198	C	0.26	1.30 [1.21- 1.39]
rs7772437	A	-0.18	0.83 [0.78- 0.89]
rs7827332	G	-0.23	0.79 [0.73- 0.86]
rs704	A	-0.18	0.83 [0.78- 0.89]
rs11751347	T	-0.31	0.73 [0.65- 0.83]
rs7090203	T	0.34	1.40 [1.23- 1.60]

*ORs and their respective CIs are not parameters used in the PRS model, they are shown here for comprehensive representation of the selected variants
PRS, Polygenic risk score; OR, odds ratio; CI, confidence interval

Supplementary Table 15. GCA risk stratification based on PRS results.

Risk percentile threshold	Corresponding risk score threshold	OR [95% CI]	p-value	Cases identified as high-risk (true positives)	Controls identified as high-risk (false positives)	Controls identified as low-risk (true negatives)	Cases identified as low-risk (false negatives)
50	0.0198	1.88 [1.59-2.22]	8.24E-14	607	557	737	427
55	0.0240	1.94 [1.64-2.29]	6.23E-15	559	489	805	475
60	0.0283	1.99 [1.68-2.35]	1.21E-15	508	423	871	526
65	0.0326	2.19 [1.84-2.60]	6.87E-19	464	351	943	570
70	0.0369	2.40 [2.01-2.88]	1.20E-21	416	283	1011	618
75	0.0411	2.51 [2.07-3.04]	3.71E-21	357	225	1069	677
80	0.0482	2.53 [2.05-3.12]	1.24E-18	292	174	1120	742
85	0.0545	2.60 [2.05-3.29]	7.75E-16	225	125	1169	809
90	0.0628	2.87 [2.15-3.82]	1.73E-13	157	76	1218	877
95	0.0740	2.85 [1.91-4.24]	1.47E-07	80	37	1257	954

P-values represent the significance level of the Chi-squared test in relation to the null hypothesis, which assumes no association between the variables.
GCA, Giant cell arteritis; OR, Odds ratio; CI, Confidence interval.

Members of the GCA consortia

Spanish GCA Group

First name	Surname
José Luis	Callejas
Luis	Caminal-Montero
Marc	Corbera-Bellalta
Eugenio	de Miguel
J. Bernardino	Díaz-López
María Jesús	García-Villanueva
Carmen	Gómez-Vaquero
Mercedes	Guijarro-Rojas
Ana	Hidalgo-Conde
Begoña	Marí-Alfonso
Agustín	Martínez-Berriochoa
Inmaculada C.	Morado
Javier	Narváez
Marc	Ramentol-Sintas
Aleida	Martínez-Zapico
Víctor Manuel	Martínez-Taboada
José A.	Miranda-Filloy
Jordi	Monfort
Mercedes	Pérez-Conesa
Sergio	Prieto-González
Enrique	Raya
Raquel	Ríos-Fenández
Julio	Sánchez-Martín
Bernardo	Sopeña
Laura	Tío
Ainhoa	Unzurrunzaga

UK GCA Consortium

First name	Surname
Oliver	Wordsworth
Isobel	Whitwell
Jessica	Brock
Victoria	Douglas
Chamila	Hettiarachchi
Jacqui	Bartholomew
Stephen	Jarrett
Gayle	Smithson
Michael	Green
Pearl Clark	Brown
Cathy	Lawson
Esther	Gordon
Suzanne	Lane
Rebecca	Francis
Bhaskar	Dasgupta
Bridgett	Masunda
Jo	Calver
Yusuf	Patel
Charlotte	Thompson
Louise	Gregory
Sarah	Levy
Ajit	Menon
Amy	Thompson
Lisa	Dyche
Michael	Martin
Charles	Li
Ramasharan	Laxminarayan
Louise	Wilcox
Ralph	de Guzman
John	Isaacs
Alice	Lorenzi
Ross	Farley
Helain	Hinchcliffe-Hume
Victoria	Bejarano
Susan	Hope
Pradip	Nandi
Lynne	Stockham
Catherine	Wilde

Donna	Durrant
Mark	Lloyd
Chee-Seng	Ye
Rob	Stevens
Amjad	Jilani
David	Collins
Suzannah	Pegler
Ali	Rivett
Liz	Price
Neil	McHugh
Sarah	Skeoch
Diana	O’Kane
Sue	Kirkwood
Saravanan	Vadivelu
Susan	Pugmire
Shabina	Sultan
Emma	Dooks
Lisa	Armstrong
Hala	Sadik
Anupama	Nandagudi
Tolu	Abioye
Angelo	Ramos
Steph	Gumus
Nidhi	Sofat
Abiola	Harrison
Abi	Seward
Susan	Mollan
Ray	Rahan
Helen	Hawkins
Hedley	Emsley
Anna	Bhargava
Vicki	Fleming
Marianne	Hare
Sonia	Raj
Emmanuel	George
Nicola	Allen
Karl	Hunter
Eoin	O’Sullivan
Georgina	Bird
Malgorzata	Magliano
Katarina	Manzo

Bobbie	Sanghera
David	Hutchinson
Fiona	Hammonds
Poonam	Sharma
Richard	Cooper
Graeme	McLintock
Zaid S.	Al-Saffar
Mike	Green
Kerry	Elliott
Tania	Neale
Janine	Mallinson
Peter	Lanyon
Marie-Josephe	Pradere
Natasha	Jordan
Ei Phyu	Htut
Thelma	Mushapaidzi
Donna	Abercrombie
Sam	Wright
Jane	Rowlands
Chetan	Mukhtyar
James	Kennedy
Damodar	Makkuni
Elva	Wilhelmsen
Michael	Kouroupis
Lily	John
Rod	Hughes
Margaret	Walsh
Marie	Buckley
Kirsten	Mackay
Tracey	Camden-Woodley
Joan	Redome
Kirsty	Pearce
Thiraupathy	Marianayagam
Carina	Cruz
Elizabeth	Warner
Ishmael	Atchia
Claire	Walker
Karen	Black
Stacey	Duffy
Lynda	Fothergill
Rebecca	Jefferey

Jackie	Toomey
Ceril	Rhys-Dillon
Carla	Pothecary
Lauren	Green
Tracey	Toms
Linda	Maher
Diana	Davis
Amrinder	Sayan
Mini	Thankachen
Mahdi	Abusalameh
Jessica	Record
Asad	Khan
Sam	Stafford
Azza	Hussein
Clare	Williams
Alison	Fletcher
Laura	Johson
Richard	Burnett
Robert	Moots
Helen	Frankland
James	Dale
Karen	Black
Kirsten	Moar
Carol	Hollas
Ben	Parker
Derek	Ridings
Sandhya	Eapen
Sindhu	John
Jo	Robson
Lucy Belle	Guthrie
Rose	Fyfe
Moira	Tait
Jonathan	Marks
Emma	Gunter
Rochelle	Hernandez
Smita	Bhat
Paul	Johnston
Muhammad	Khurshid
Charlotte	Barclay
Deepti	Kapur
Helen	Jeffrey

Anna	Hughes
Lauren	Slack
Eleri	Thomas
Anna	Royon
Angela	Hall
Jon	King
Sindi	Nyathi
Vanessa	Morris
Madhura	Castelino
Ellie	Hawkins
Linda	Tomson
Animesh	Singh
Annalyn	Nunag
Stella	O'Connor
Nathan	Rushby
Nicola	Hewitson
Kenny	O'Sunmboye
Adam	Lewszuk
Louise	Boyles
Martin	Perry
Emma	Williams
Christine	Graver
Emmanuel	Defever
Sanjeet	Kamanth
Dominic	Kay
Joe	Ogor
Louise	Winter
Sarah	Horton
Gillian	Welch
Kath	Hollinshead
James	Peters
Julius	Labao
Andrea	Dmello
Julie	Dawson
Denise	Graham
Denise	De Lord
Jo	Deery
Tracy	Hazelton

Vasculitis Clinical Research Consortium

First name	Surname
Simon	Carette
Sharon	Chung
David	Cuthbertson
Lindsy J.	Forbess
Ora	Gewurz-Singer
Gary S.	Hoffman
Curry L.	Koening
Kathleen M.	Maksimowicz-McKinnon
Carol A.	McAlear
Larry W.	Moreland
Christian	Pagnoux
Philip	Seo
Ulrich	Specks
Robert F.	Spiera
Antoine	Sreih
Kenneth J.	Warrington
Paul A.	Monach
Michael	Weisman
